# Use of deep neural network ensembles to identify embryonic-fetal transition markers: repression of *COX7A1* in embryonic and cancer cells

**DOI:** 10.18632/oncotarget.23748

**Published:** 2017-12-28

**Authors:** Michael D. West, Ivan Labat, Hal Sternberg, Dana Larocca, Igor Nasonkin, Karen B. Chapman, Ratnesh Singh, Eugene Makarev, Alex Aliper, Andrey Kazennov, Andrey Alekseenko, Nikolai Shuvalov, Evgenia Cheskidova, Aleksandr Alekseev, Artem Artemov, Evgeny Putin, Polina Mamoshina, Nikita Pryanichnikov, Jacob Larocca, Karen Copeland, Evgeny Izumchenko, Mikhail Korzinkin, Alex Zhavoronkov

**Affiliations:** ^1^ AgeX Therapeutics, Inc., Alameda, CA, USA; ^2^ BioTime, Inc., Alameda, CA, USA; ^3^ Johns Hopkins University, Baltimore, MD, USA; ^4^ Pharmaceutical Artificial Intelligence Department, Insilico Medicine, Inc., Emerging Technology Centers, Johns Hopkins University at Eastern, Baltimore, MD, USA; ^5^ Moscow Institute of Physics and Technology, Dolgoprudny, Russia; ^6^ Computer Technologies Lab, ITMO University, St. Petersburg, Russia; ^7^ Boulder Statistics, Boulder, CO, USA; ^8^ Johns Hopkins University, School of Medicine, Department of Otolaryngology-Head and Neck Cancer Research, Baltimore, MD, USA; ^9^ The Biogerontology Research Foundation, Trevissome Park, Truro, UK; ^10^ Innopolis University, Innoplis, Russia

**Keywords:** cancer marker, Warburg effect, embryonic-fetal transition, deep neural network, stem cells

## Abstract

Here we present the application of deep neural network (DNN) ensembles trained on transcriptomic data to identify the novel markers associated with the mammalian embryonic-fetal transition (EFT). Molecular markers of this process could provide important insights into regulatory mechanisms of normal development, epimorphic tissue regeneration and cancer. Subsequent analysis of the most significant genes behind the DNNs classifier on an independent dataset of adult-derived and human embryonic stem cell (hESC)-derived progenitor cell lines led to the identification of *COX7A1* gene as a potential EFT marker. *COX7A1*, encoding a cytochrome C oxidase subunit, was up-regulated in post-EFT murine and human cells including adult stem cells, but was not expressed in pre-EFT pluripotent embryonic stem cells or their *in vitro*-derived progeny. *COX7A1* expression level was observed to be undetectable or low in multiple sarcoma and carcinoma cell lines as compared to normal controls. The knockout of the gene in mice led to a marked glycolytic shift reminiscent of the Warburg effect that occurs in cancer cells. The DNN approach facilitated the elucidation of a potentially new biomarker of cancer and pre-EFT cells, the embryo-onco phenotype, which may potentially be used as a target for controlling the embryonic-fetal transition.

## INTRODUCTION

In contrast to embryonic cells, fetal and adult-derived (F/A) cells often show reduced potential for organogenesis *in vitro* and epimorphic regeneration following injury *in vivo* [1]. The developmental timing of loss of epimorphosis potential cannot be fixed precisely, and likely varies with tissue type, however, one specific event - the embryonic-fetal transition (EFT) which occurs at eight weeks of human development (Carnegie Stage 23) [2], marks a loss of scarless skin regeneration in placental mammals [3]. Marsupial species also show scarring as opposed to regeneration beginning at about pouch day 9, which corresponds to approximately eight weeks of human development [4]. The observation that many species show increased regenerative potential in the embryonic or larval state [5], suggests that tissue regeneration, as opposed to scarring, may reflect the presence of an embryonic, as opposed to F/A phenotype. However, there are few molecular markers of the EFT to test its role in repressing epimorphic regeneration or the re-emergence of an embryonic phenotype in cancer [1, 6]. In this research, we undertook the first attempt to identify these markers utilizing deep learning algorithms and to analyze their expression in adult, malignant and embryonic states. To identify gene expression markers of the EFT, we analyzed large datasets for global patterns distinguishing cells prior to and following the transition. While being a daunting task due to data size and complexity, pattern recognition is a perfect fit for machine learning algorithms which have the potential to markedly enhance efficiency and accuracy. Deep learning is a type of machine learning in which high-level representations are constructed from input data via a series of hierarchical, multilayer feature extractions in deep neural networks (DNNs) [7, 8].

To date, deep learning has been utilized in a variety of biological applications [9], from prediction of alternate splicing code [10] to the analysis of protein secondary structure [11], drug-induced hepatotoxicity [12], and long non-coding RNAs [13]. The number of potential applications are, however, more diverse, from basic classification to prediction [14–16], modeling [14], image processing [15], and even text mining. Moreover, the complex, noisy, high-dimensional, multi-platform data collated in many biological databases are well suited to deep learning. In a recent example, a deep learning algorithm succeeded in integrating otherwise incompatible multi-platform genomic data to cluster cancer patients by cancer subtype [16].

Transcriptional data has many challenging features, including high dimensionality, noise, and multiple, often incompatible, platforms. One of the problems with deep learning is that high dimensional data, such as that associated with gene expression, must have the dimensionality reduced prior to training of a deep network. We recently developed an algorithm, iPANDA [17], to calculate pathway activation strength (PAS) for signaling pathways activated or repressed in a given condition. PAS calculation reduces dimensionality by reducing thousands of changes in gene expression down to a relatively small number of biologically meaningful changes in signaling pathway activity [18, 19].

In the present study, human gene expression data from Affymetrix and Illumina platforms of pluripotent stem cells, their derivative embryonic progenitor cell lines (as described in [20]), adult stem cells and adult derived cell lines are used to train platform-specific DNN ensembles (available online at www.Embryonic.AI). We demonstrate the accuracy of DNN ensembles in classifying embryonic vs. adult cells and use them to generate a list of statistically-significant gene expression markers that can differentiate between the two states. Here we validate these candidate markers using transcriptomic data from an independent collection of embryonic progenitor and F/A cells, focusing on the salient marker, *COX7A1*.

The human *COX7A1* gene encodes cytochrome c oxidase subunit 7A1 protein [21, 22]. This protein plays a role in the super-assembly of the multi-unit heteromeric complexes of the mitochondrial respiratory chain such as complex IV, which consists of three catalytic subunits encoded by mitochondrial genes and multiple structural/regulatory subunits encoded by nuclear genes [23].

We report here the development of a DNN ensemble that reveals *COX7A1* as a marker of the embryo-onco phenotype. We demonstrate its down-regulation in multiple embryonic and cancer cell lines and show that its expression in adult cell lines is associated with hypomethylation. Our findings also demonstrate that *COX7A1* repression in embryonic and tumor cell lines is associated with a metabolic shift toward glycolysis reminiscent of Warburg effect observed in cancer [24].

## RESULTS

### DNN ensemble classifier demonstrates best performance among all machine learning techniques

We gathered and preprocessed transcriptomic profiles of 12,404 healthy untreated tissue samples from Affymetrix (4,822 samples) and Illumina (7,582 samples) microarray platforms to train the classifiers. The collected samples were assigned to the following five categories: embryonic stem cells (ESCs), induced pluripotent stem cells (iPSCs), embryonic progenitor cells (EPCs), adult stem cells (ASCs) and adult cells (ACs).

We separately trained six different classifiers on each microarray platform as follows: K-nearest neighbors (kNN), logistic regression with PCA-based dimensionality reduction (LR), support vector machines (SVM), gradient boosting machines (GBM), and multiclass deep neural network (DNN). We also developed a more computationally demanding but more accurate method, which employs an ensemble of 20 two-class deep neural networks (DNN ensemble) because usage of single multiclass DNN has multiple drawbacks. We performed a hyperparameter search for all classifiers except for the DNN ensemble. In the case of the DNN ensemble, we used optimal network hyperparameters obtained for the single DNN (final hyperparameters are shown in Supplementary Table 1). The performance of the classifiers is shown in Supplementary Figure 1. The DNN achieved a mean 0.99 F1 score (probability that the guesses are correct) on the Affymetrix microarray training dataset, and 0.75 on the external validation dataset, while other methods achieved a 0.50–0.64 F1 score on the external validation dataset. The DNN achieved a mean 0.99 F1 score on the llumina microarray training dataset, and 0.83 on the external validation dataset, while other methods achieved 0.52–0.58 F1 score on the external validation dataset. Classical methods, such as kNN and LR performed noticeably worse than SVM, XGB and DNN methods. Our DNN ensemble performed substantially better with about 12% relative improvement for Affymetrix and 36% relative improvement for Illumina (Supplementary Figure 1A and 1B).

**Figure 1:**
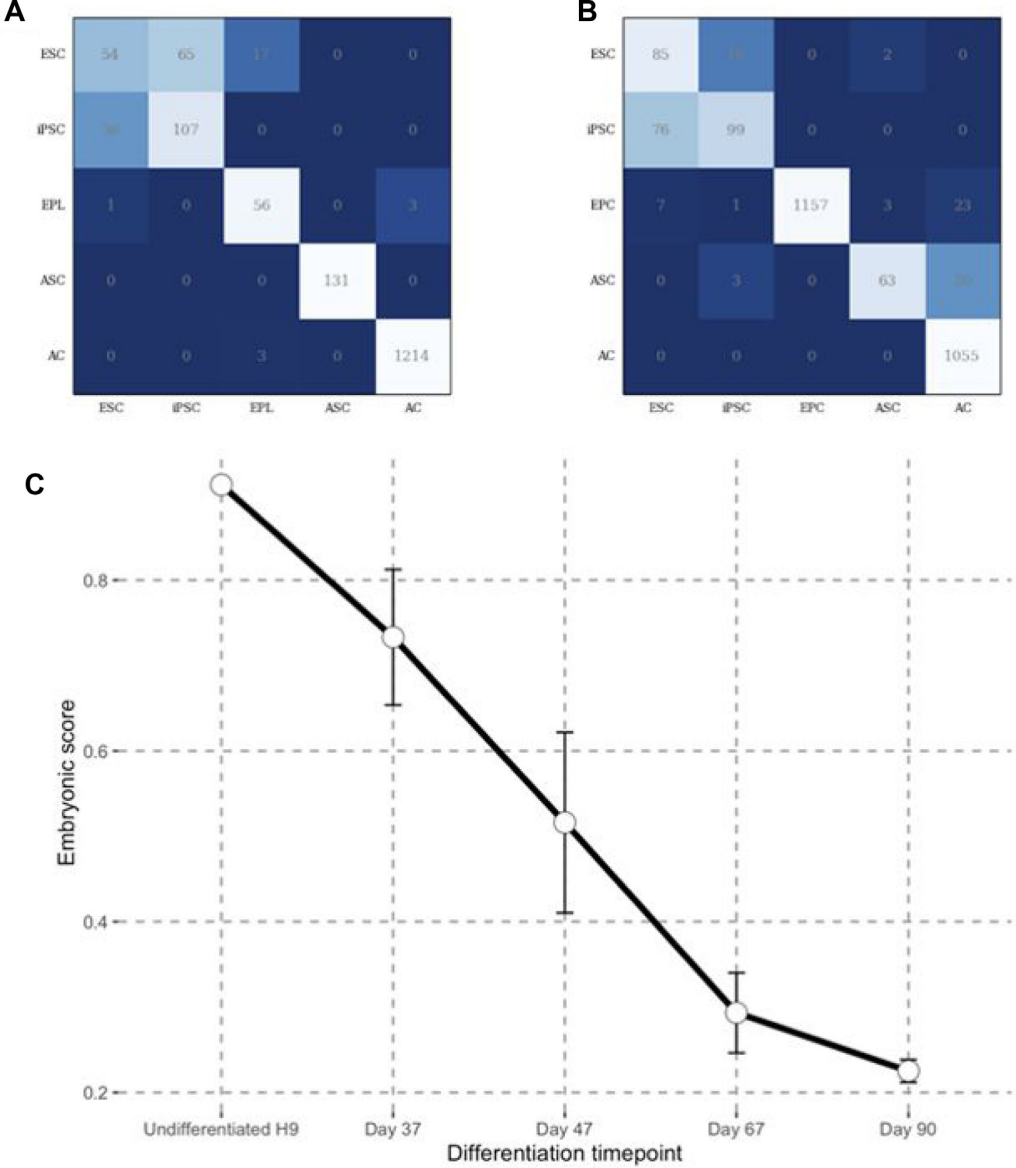
Predicting embryonic state through DNN ensemble (**A**) Validation confusion matrix performance for DNN ensemble trained on Illumina data. (**B**) Validation confusion matrix performance for DNN ensemble trained on Affymetrix data. (**C**) Embryonic scores obtained through Affymetrix DNN ensembles for GEO next generation data set GSE62193 consisting of samples representing different stages of human photoreceptor development from ES cells.

We reasoned that dimensionality reduction might improve the accuracy of our methods. We therefore tried a pathway level analysis approach that we had previously established called iPanda. This gene aggregation method allows preservation of biological function while dramatically reducing dimensionality. Using pathway level analysis (Supplementary Figure 1C and 1D), we demonstrated that despite the lower accuracy on the training set, the DNN ensemble performance on the validation set is similar to what was achieved at the gene level with F1 scores 0.74 and 0.81 for Affymetrix and Illumina platforms, respectively. In order to prove that our DNN ensemble could successfully distinguish between the five classes, we utilized a validation confusion matrix for samples from each platform (Figure 1A and 1B). Our DNN clearly resolved all class comparisons except for ESC vs. iPSC. The reason is likely because fully reprogrammed “high quality” iPSC lines are largely indistinguishable from ESC lines at the transcriptional level [25].

Having shown that our DNN ensemble could distinguish between the five different classes of samples, we next used it to position any sample on a differentiation axis. We developed an integrative Embryonic Score (E Score, see Materials and Methods) based on the DNN ensemble output to determine how close a sample is to the embryonic state. We assigned an E Score of 1 to represent an embryonic state and E Score of 0 to represent the adult state. Thus, any intermediate state has an E Score between 1 and 0. The E Score can be computed either with Affymetrix or Illumina based DNN ensembles. We tested the performance of the E score using our Affymetrix-based DNN ensemble for an RNA-Seq data set consisting of samples from various stages of human photoreceptor development [26]. We compared undifferentiated H9, ESCs and RNA samples taken at days 37, 47, 67 and 90 of photoreceptor development. Effective gene counts corrected for bias were used as an input. We observed a clear decrease in the E score upon photoreceptor differentiation (Figure 1C). Considering progenitors as embryonic tissues with E score of 0.5 and adult stem cells as fetal tissues with E score of 0.7, the E score for EFT spans in the 0.5–0.7 range for the photoreceptor development dataset. The above example shows that even though DNN ensemble was trained on samples from microarray platforms it is also suitable for RNA-Seq data analysis.

### Identification *COX7A1* gene as embryonic - fetal transition biomarker

We attempted to identify individual genes as potential expression markers of the mammalian EFT by assessing the most highly ranked genes behind the DNN ensemble classifier that was trained on the Illumina dataset because Illumina datasets were more homogenous than Affymetrix. Our analysis of the classifier revealed that *COX7A1* was among the genes with the highest ranking in the Illumina DNN ensemble (Supplementary Table 2). Gene expression analysis of an additional panel of transcriptomic profiles (RNA-seq proprietary dataset; BioTime, Inc.) from 15 diverse adult-derived cell types representing derivatives of endoderm, mesoderm, ectoderm, and neural crest cell types (adult group) compared to 17 diverse clonal embryonic progenitor cell lines (embryonic group) independently confirmed the DNN results. Our *t*-test analysis identified several genes with statistically significant difference in level of expression between the embryonic and adult groups (Supplementary Table 3). The most significantly (*p* < 0.0001) dysregulated genes between the adult and embryonic groups included *COX7A1* which again showed increased expression in the adult-derived cell lines. Thus, we were able to identify *COX7A1* expression as highly associated with the F/A state compared to the embryonic state using two independent analytical methods on two different data sets. We therefore selected *COX7A1* for further analysis as a novel biomarker of the EFT.

**Figure 2:**
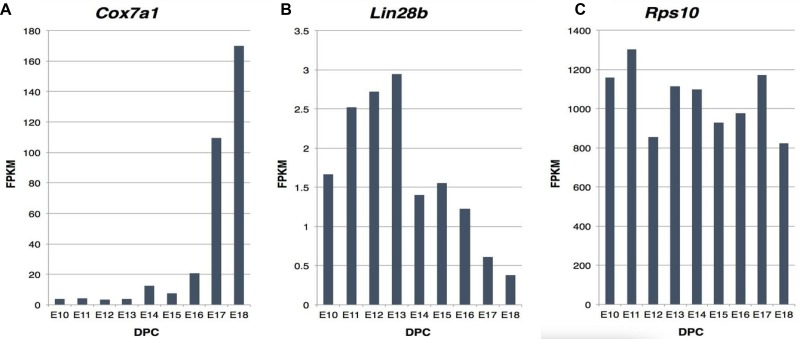
Expression analysis of *COX7A1*, *Lin28b* and *Rps10* transcripts in mouse development measured by RNA-seq (**A**, **B**) Analysis of expression of key embryonic-fetal makers had been conducted in mouse to demonstrate gradient upregulation of *COX7A1* along with gradient downregulation of *Lin28b* during mouse embryonal development as measured by NGS, where FPKM is relative RNA expression units and DPC (days post coitum) reflects embryonic stage. (**C**) *Rps10* expression was used to ensure equal amount of RNA was used across all samples.

We next examined the expression of *COX7A1* during mouse and human embryonic development to directly determine if its temporal expression pattern during development was consistent with its role as an EFT marker as indicated by our bioinformatic findings. We first assessed *COX7A1* expression in total mouse embryo RNA that was sampled during embryonic time points spanning the murine EFT (stage E10 to E18 which correspond to Theiler stages TS16–TS26 and represent days 10–18 post coitem). We included analysis of *Lin28b* expression as a control for the embryonic state because it is known to be associated with pluripotency and embryonic development [27–29]. As shown in Figure 2, *COX7A1* showed a marked up-regulation at the time point approximating the murine EFT (E16) while the expression *Lin28b* decreased during the same period (Figure 2). The constitutive marker *RPS10* was used as a control for normalization of RNA levels across all samples.

**Figure 3:**
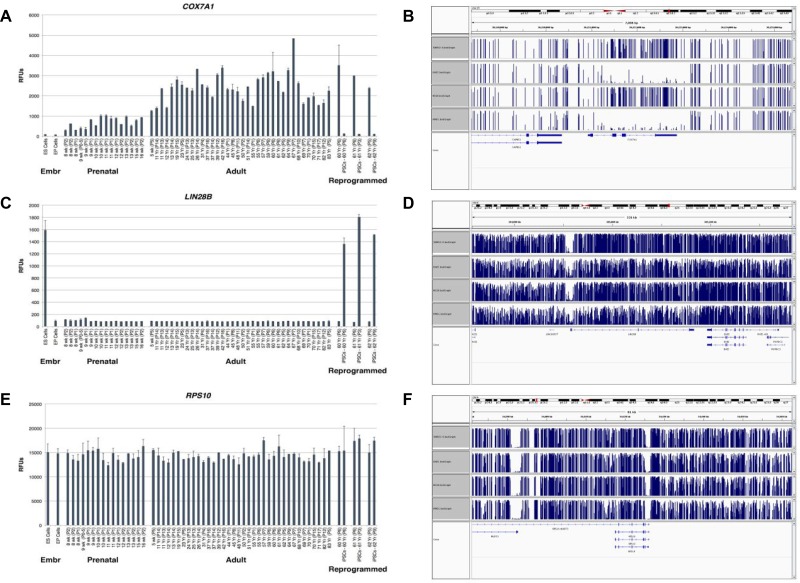
Expression analysis of *COX7A1*, *LIN28B* and *RPS10* transcripts in human tissues at different stages of development along with methylation analysis of *COX7A1*, *LIN28B* and *RPS10* genes in human cell lines (**A**, **C**) Dermal fibroblasts of the upper arm from developmental stages spanning the onset of fetal development (eight weeks of gestation) through adulthood were synchronized in quiescence *in vitro* and RNA subjected to analysis on Illumina gene expression bead arrays. *COX7A1* had been upregulated in adult stages while *LIN28B* displayed the opposite pattern. It should be noted that in iPSCs generated from matching adult tissues the level of expression of these genes demonstrated the reverse pattern compared to adult tissues. (**B**, **D**) Four human cell lines were used for methylation analysis by bisulfite sequencing. In two embryonic derived cell lines, 4D20.8 and 30-MV2-6, genomic DNA appears to be methylated at *COX7A1* region, while in two adult derived cell lines where *COX7A1* expression had been detected its genomic region appears to be relatively unmethylated. *LIN28B* methylation pattern seems to be unchanged in embryonic and adult derived cell lines. Blue bars represent levels of methylation, one bar for every methylated C. The height of the bar corresponds to the fraction of reads covering that C that are methylated (the highest bars = 1–meaning Cs in all reads are methylated). (**E**, **F**) *RPS10* was used as a housekeeping control for methylation and expression analysis.

We next assessed *COX7A1* expression during human development using RNA from early passage dermal fibroblasts of the upper arm from developmental stages spanning the onset of human fetal development (eight weeks of gestation) through adulthood for analysis on Illumina gene expression bead arrays. As shown at Figure 3A, *COX7A1* gene expression was induced at eight weeks of gestation and appeared to progressively increase throughout fetal and postnatal development, reaching its maximum level in adulthood. Interestingly, *COX7A1* expression was also dramatically decreased in iPSC that were reprogrammed from adult fibroblasts. In contrast, the expression of *LIN28B* is markedly upregulated in hESC and iPSC compared to F/A cells, and expression was inversely correlated *COX7A1* (Figure 3C). *RPS10* was used to normalize RNA levels (Figure 3E). We estimated methylation levels of the genomic region encoding this gene in order to elucidate a potential mechanism of *COX7A1* silencing in embryonic tissues. We found that *COX7A1* was significantly methylated in human embryonic stem cell-derived progenitor cell lines corresponding to mesenchymal and endothelial phenotypes (4D20.8 and 30-MV2-6 respectively), as compared to human adult cell counterparts (HMSC and HAEC (Figure 3B)). The same assay applied to *LIN28B* and *RPS10* did not reveal noticeable changes in their methylation pattern (Figure 3D, 3F).

Further evidence of the utility of *COX7A1* as a marker of the F/A state and *LIN28B* as a marker of the embryonic state, expression of this pair of genes was consistently inversely correlated in human cell lines (BioTime proprietary data). The correlation plot (Supplementary Figure 4) demonstrated an inverse correlation between *LIN28B* and *COX7A1* of 83.3% (95% CI: 66.4–92.7).

### *COX7A1* is downregulated in cancer and embryonic-derived cell lines

**Figure 4:**
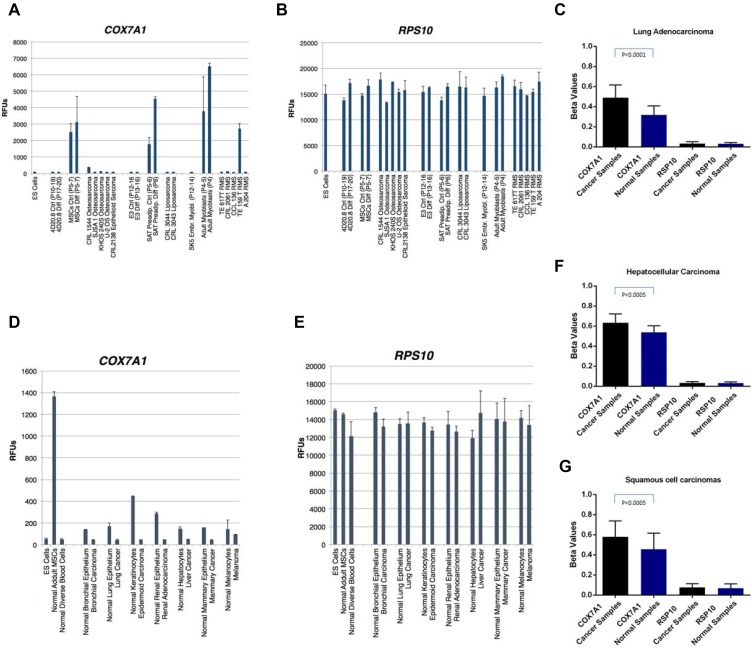
Expression analysis of *COX7A1* and *RPS10* transcripts in cancer, embryonic and adult cell lines along methylation analysis of *COX7A1* and *RPS10* genes in cancer and healthy samples (**A**) Embryonic progenitors capable of osteochondral differentiation such as the line 4D20.8 showed no evidence of *COX7A1* expression either in the progenitor state or in the differentiated state despite expressing high levels of osteochondral markers. Similarly, embryonic progenitor adipocytes E3 and myoblasts SK5 did not express *COX7A1*. In contrast, adult-derived MSCs expressed *COX7A1* before and after differentiation. The same situation was observed with adult-derived preadipocytes and myoblasts. When expression levels of *COX7A1* were measured in osteosarcomas, liposarcomas and rhabdomyosarcoma all lines except one showed an embryonic pattern of *COX7A1* expression. (**D**) Several cancer cell lines demonstrated decreased level of *COX7A1* expression compared to healthy tissue controls; ESCs and adult MSCs were used as internal controls for *COX7A1* expression. (**C**, **F**, **G**) Methylation analysis of cancer samples obtained from lung, liver and oral carcinomas demonstrated statistically significant increase of methylation of *COX7A1* compare to healthy controls. (**B**, C, **E**–G) *RPS10* gene used as a housekeeping control for methylation and expression analysis.

Since pre-EFT cell lines and cancer cell lines share many of morphological, proliferative and metabolic features, we reasoned that *COX7A1* repression might also be a marker of this hallmark of cancer. We therefore examined the expression of human *COX7A1* in three types of sarcomas (osteosarcoma, liposarcoma, and rhabdomyosarcoma) and compared them to corresponding normal embryonic progenitors (osteochondral (4D20.8), adipocyte (E3) and myogenic (SK5)), as well as corresponding adult-derived cells (normal bone marrow derived MSCs (hMSC-BM), subcutaneous adipose tissue (SAT)-derived preadipocytes, and myoblasts). Embryonic progenitors capable of osteochondral differentiation (4D20.8) showed no evidence of *COX7A1* expression in either the progenitor state or in the differentiated state despite expressing high levels of osteochondral markers (Figure 4A). In contrast, adult-derived MSCs expressed *COX7A1* before and after differentiation. All four osteosarcoma lines showed evidence of an embryonic pattern of low or absent expression of *COX7A1*, including the epithelioid sarcoma cell line (CRL21380). Similarly, an embryonic progenitor cell line capable of lipogenic differentiation (E3) did not express *COX7A1* despite expressing robust markers of adipocyte differentiation (data not shown), while adult-derived subcutaneous adipose tissue (SAT) preadipocytes expressed *COX7A1* both as relatively undifferentiated cells and as fully differentiated adipocytes. As in the case of the osteosarcomas, the two liposarcoma cell lines studied, also displayed an embryonic pattern of undetectable *COX7A1* gene expression. Lastly, five rhabdomyosarcoma cell lines were similarly studied in comparison to an embryonic myoblast progenitor cell line SK5, and adult-derived myoblasts. *COX7A1*, previously described as being highly expressed in skeletal and cardiac myocytes [30], was expressed at high levels in the adult-derived myoblasts, but was not expressed in the embryonic myoblast progenitor line, SK5, nor was it expressed in 4 out of 5 of the rhabdomyosarcoma cell lines (Figure 4A).

Extending our analysis of *COX7A1* expression to other forms of cancer, we examined cancer cell lines obtained from lung, liver, kidney, breast and skin (Figure 4D). Collectively in all cancer cell lines examined we observed downregulation of *COX7A1* expression compared to matching control normal counterparts. Blood cell cancers were excluded from our analysis due to the observation that *COX7A1* was not expressed in any differentiated blood cell types tested (data not shown).

To elucidate the possible mechanism behind *COX7A1* suppression in cancer, we analyzed the methylation landscape of the several different cancer cell types that demonstrated downregulation of *COX7A1* compared to normal tissues (Figure 4D). We observed a statistically significant increase in methylation of the *COX7A1* gene in adenocarcinoma, hepatocellular carcinoma and squamous cell carcinoma using publicly available lung methylation data (Figure 4C, 4F, 4G) supporting a potential role for DNA methylation in regulating *COX7A1* expression in cancer. RPS10 was used to normalize RNA and methylation levels (Figure 4E–4GE). However, since these findings cannot resolve whether methylation is the cause or effect of repressed gene expression, we investigated the possible effect of demethylation treatment on *COX7A1* expression. The methylation level of the *COX7A1* gene decreased upon 5-aza treatment but the gene expression level remained the same implying that methylation may not be the sole point of regulation of *COX7A1* gene repression (Supplementary Figure 5).

**Figure 5:**
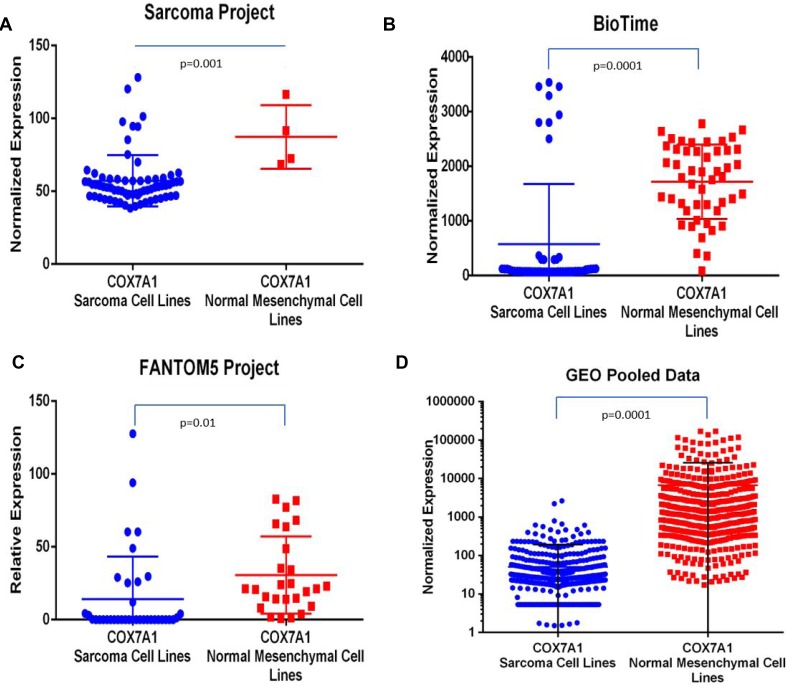
Comparative analysis of *COX7A1* expression in sarcoma cell lines *vs.* normal mesenchymal cell lines Analysis by *t*-test demonstrated statistically significant decrease of *COX7A1* expression in cancer cell lines comparing to matching controls. Normalized expression or relative expression values of *COX7A1* were calculated using transcriptomic data from (**A**) Sarcoma Project–67 samples, (**B**) BioTime internal data–103 samples, (**C**) Fantom5 Project–71 samples, (**D**) GEO pooled sarcoma and mesenchymal cell lines–over 1000 samples.

Lastly, we performed an investigation of *COX7A1* expression in sarcoma cancer lines utilizing all available transcriptomic data collected from the public domain combined with internal (BioTime, Inc.) data. We used datasets from the following four independent sources (Figure 5): Sarcoma Project (https://sarcoma.cancer.gov/sarcoma/), BioTime, Inc. (proprietary data), FANTOM5 Project (http://fantom.gsc.riken.jp/5/) and pooled cross platform normalized data from multiple GEO repository datasets (https://www.ncbi.nlm.nih.gov/gds/?term=sarcoma). In all four groups, we observed a statistically significant downregulation of *COX7A1* expression in sarcoma cell lines compared to normal adult mesenchymal cells (Figure 5). Finally, we extended our analysis to all available cancer cell lines from the highly representative FANTOM5 dataset (over 500 samples) to further test the hypothesis that *COX7A1* is generally under-expressed in cancer. As shown in Supplementary Figure 6, the level of *COX7A1* gene expression was reduced in cancer lines compared to normal cell lines.

### *COX7A1* control of energy production mode in mouse knock-out model

**Figure 6:**
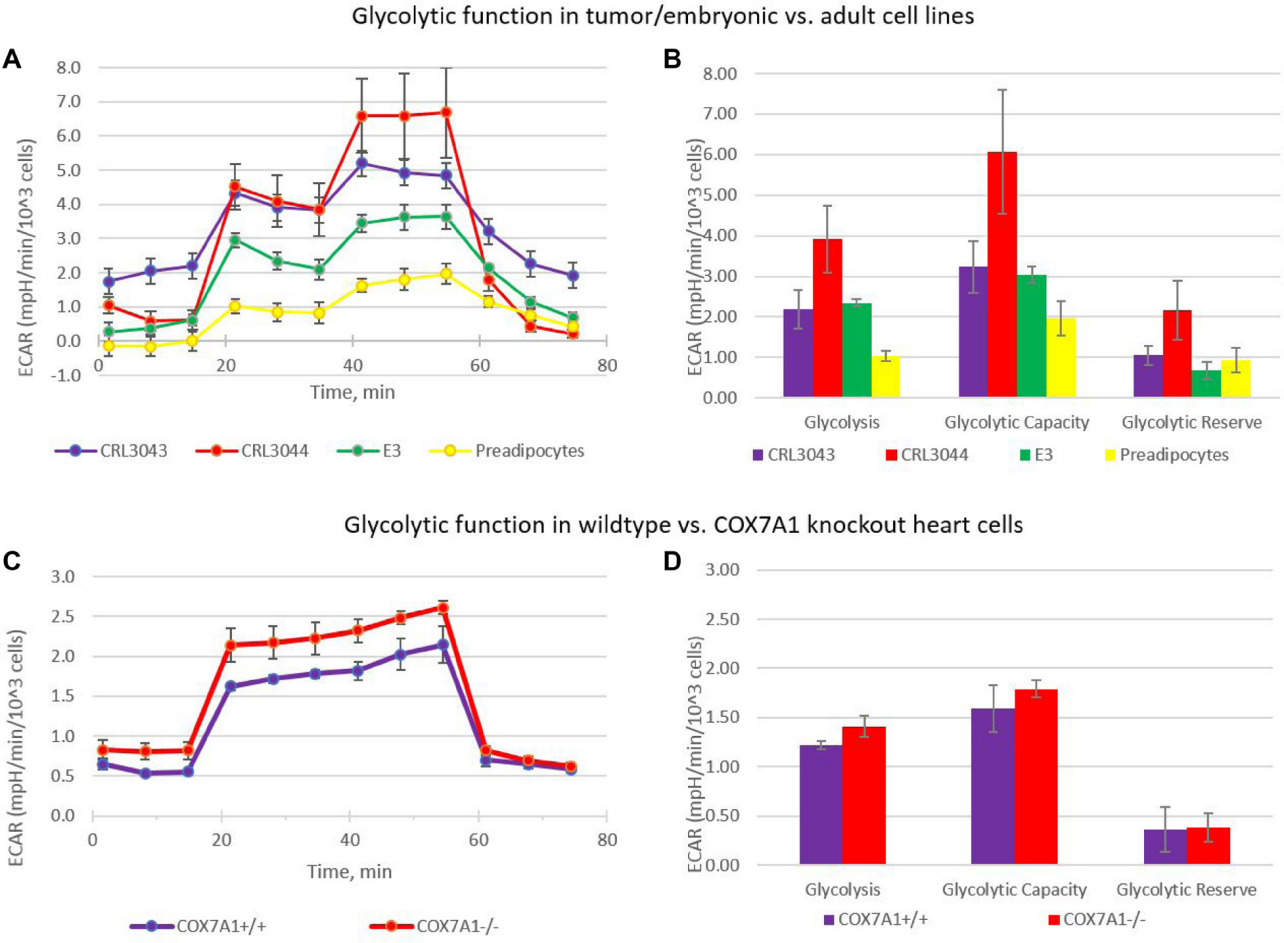
Warburg effect in cells with *COX7A1* deletion (**A**, **B**) The figure shows that a glycolytic shift, normally observed in cancer cell lines (CRL3042, CRL3044) and E3 (hESC derived) progenitor cell line, but not adult primary preadipocyte cells, (**C, D**) Glycolytic shift is also observed in cells, lacking COX7A1 gene: heart cells derived from *COX7A1* –/− mouse; heart cells from a *COX7A1* +/+ littermate mouse, age 2 months. Just as in cancer and E3 progenitor cells, glycolysis level was higher in cells with *COX7A1* deletion.

Many cancer cell lines demonstrate a metabolic shift from oxidative phosphorylation to aerobic glycolysis [24, 31, 32] called the Warburg Effect as well as a heterogeneity corresponding to clinical outcomes. This effect is also observed in some embryonic pluripotent cell lines [33] but not in normal adult cells [24]. We first compared extracellular acidification rates (ECAR) in a lipogenic series of cell lines consisting of two liposarcoma cancer lines (CRL3034 and CRL3044), an embryonic adipocyte progenitor line (E3) and adult-derived subcutaneous preadipocytes (primary culture). Cancer and embryonic cell lines showed an increased ECAR while the adult-derived preadipocyte cell line showed a statistically significant decreased ECAR (Figure 6A, 6B) that is typical of adult cell lines with upregulated *COX7A1* expression. We reasoned that *COX7A1* expression may provide increased capacity for OXPHOS, and therefore decided to test the influence of *COX7A1* expression on energy production mode using a mouse *COX7A1* knock-out model (Supplementary Figure 7) to determine whether the absence of *COX7A1* would result in a glycolytic shift. Primary cultures of cardiomyocytes obtained from *COX7A1* –/− mouse displayed elevated ECAR levels compared to the equivalent control cell culture derived from *COX7A1* +/+ littermate mouse (Figure 6C, 6D). We therefore conclude that the down-regulation of *COX7A1* gene expression may be sufficient to decrease OXPHOS capacity relative to glycolysis correlating with the long-noted trend toward anaerobic glycolysis in embryonic development re-emerging in cancer.

## DISCUSSION

To date, it has been a daunting task to identify markers of EFT [34]. For example, a comparison of mouse embryonic stem cells with committed adult stem cells led to identification over 200 differentially expressed genes [35, 36]. Moreover, although similar experiments with human embryonic stem cells pointed to the importance of signaling-based regulation in determining the embryonic state, including an increased expression of genes in the Wnt, Hh, and Notch signaling pathways [37], they were unable to identify definitive EFT markers.

In the present study, we have developed an ensemble of deep neural networks that is able to recognize different developmental stages using transcriptomic data. To the best of our knowledge, this is the first DNN-based tool designed to identify cell phenotypes spanning the EFT. The constructed DNN ensemble convincingly outperformed other powerful classification algorithms on both Affymetrix and Illumina platforms (Supplementary Figure 1). When the nested cross validation is organized in such a way that the test set is comprised of the samples coming from datasets that weren't used during the training, all standard classifiers fall short compared to DNNs. We observed that cross validation was important, especially in cases where samples from the same dataset are present in both training and validation sets. In that case, the classifier performance is greatly overestimated (validation performance above 0.9 for all methods; data not shown). This emphasizes the degree to which batch effects [38, 39] affect transcriptomic data and the need for careful selection of the cross-validation procedure to obtain unbiased estimation of classifier behavior on new data sets. Looking at the confusion matrices coming from both DNN ensembles, one can clearly see that iPSCs are often confused with ESCs (Figure 1A, 1B). This supports the fact that properly reprogrammed iPSCs are almost identical to ESCs on the transcriptome level [40, 41].

In contrast to the CellNet approach [42], our system does not distinguish between different types of differentiated cells (liver, muscle, kidney, etc.) but instead is aimed at recognizing different states of early embryonic development and identifying whether cells have traversed the EFT. Accordingly, we introduced an Embryonic Score (E Score) which acts as an integrative measure of development stage (Figure 1C). We created a public website www.Embryonic.AI (online implementation of trained DNN ensemble) to enable an E Score calculation for the broad scientific community with user provided transcriptomic data and to collect data to facilitate precise EFT identification based on additional transcriptomic data.

Identification of *COX7A1* as a marker of cells that have traversed EFT demonstrates the utility of our DNN ensemble. *COX7A1* was originally reported as a marker of cardiac and skeletal muscle suggesting a potential role in increased ATP production in these tissues. Indeed, the gene is expressed at relatively high levels in muscle cells, consistent with increased demand for OXPHOS, and *COX7A1* knockout mice develop a dilated cardiomyopathy [30]. However, paradoxically ATP levels in the knockout model were reported to be higher than normal [30] reflecting the complex role of OXPHOS in multiple metabolic pathways. *COX7A1* is also expressed in skeletal muscle and its expression is reported to be reduced during inflammation potentially consistent with a reversion to a regenerative state [43]. In another report, *COX7A1* was identified as an indirect target of HIF1A via Activin/Nodal signaling providing intriguing indications of potential *COX7A1* involvement in key phenotypic and metabolic differences between embryonic and adult cells [44]. Consistent with its role in metabolism, *COX7A1* regulation has been implicated in diabetes [45] and is induced during browning of white adipose [46, 47].

In the present study, we have demonstrated that *COX7A1* is downregulated in embryonic compared to adult cell lines using two independent cohorts of embryonic and adult cell lines. This result led us to investigate *COX7A1* expression in whole mouse embryos collected at different time points spanning the EFT event (Figure 2). The up-regulation of the gene following EFT coincided with the downregulation of *Lin28b* (a biomarker of pre-EFT cells) in the same embryo samples. Cancer cell lines which share many properties with embryonic cells showed a significant reduction in *COX7A1* compared to normal cells (Figures 4, 5). Interestingly, cytochrome C oxidase (COX) subunits are shown to be involved in prostate cancer [48] and in tumor cell survival under hypoxic conditions [49]. Whether changes in *COX7A1* expression play a causal role in regulating cellular metabolism or are the downstream result of changes in cellular metabolism remains to be determined. In addition, further studies are warranted to define potential linkage between *COX7A1* expression and various manifestations of the metabolic state such as the propensity for apoptosis. Although the exact role of *COX7A1* in the diverse metabolic pathways regulating malignancy is not yet clear, recent research reported by Mishra demonstrating that *COX7A1* overexpression in the cancer cell line, A549, can induce apoptosis via an intrinsic mechanism (Caspase 9, 3 activation) [50] supports its potential role as a tumor suppressor.

We hypothesize that variation of *COX7A1* expression level in different cancer lines could correlate with the magnitude of glycolytic shift or OXPHOS capacity in these lines and consequently with the varying degrees of tumor aggression, invasiveness, and sensitivity to chemotherapeutic regimens. Accordingly, lack of *COX7A1* expression may be responsible for the embryonic-like glycolytic phenotype of tumors and a sensitivity to anoikis, whereas malignant cells expressing the gene may correlate with a more oxidative and anoikis-resistant phenotype. In addition, unravelling the role of *COX7A1* and the mechanisms regulating its expression could provide an *in vitro* model of the EFT that could allow, for the first time, a robust system for the analysis of the down-regulation of a regenerative phenotype in many tissues at the EFT. The most intriguing question remaining to be determined that could benefit from such model systems is whether maintaining cells in a pre-EFT pattern of gene expression facilitates an embryonic-like regenerative ability without malignant transformation. One explanation for the evolutionary selection for the repression of scarless regenerative potential after EFT is that for most vertebrates, repression of regenerative potential once organogenesis is complete functions as a tumor suppression mechanism. Consistent with this hypothesis is the well-known observation that many cancers show markers of embryonic reversion including the reactivation of telomerase activity [51], oncofetal protein expression, and the Warburg effect [52]. As a result, the repression of epimorphic potential at the EFT, evidenced by the onset of *COX7A1* expression, may provide an important role in tumor suppression. Thus, the loss of epimorphic potential may allow for a limited degree of fibrotic tissue repair while simultaneously reducing the risk of malignant transformation much in the same way that repression of the telomerase catalytic component (TERT) early in development may decrease malignancy risk. Our work provides a novel *in vitro* tool for characterization of embryonic cellular states. We demonstrate the potential of the DNNs to deconvolute complex data and therefore facilitate the discovery of new cellular markers, such as *COX7A1*, which are connected to the transition between embryonic and adult/fetal states. Expression and metabolic profiling of embryonic and adult states clearly demonstrates that *COX7A1* discriminates between two distinct phenotypes: 1.) the embryo-onco phenotype comprising of highly glycolytic/OXPHOS impaired *COX7A1* negative cancer cells, iPSCs, embryonic and partially differentiated embryonic derived cell lines and 2.) the F/A phenotype comprising of all other *COX7A1* positive cells including adult stem cells. These insights provide at least one robust marker for the switch between these states. Further research is warranted to determine the extent to which highly glycolytic/OXPHOS-compromised *COX7A1* deficient cells have increased regenerative ability, and the role of the phenotype in diverse aspects of tumor cell biology. The use of robust markers such as *COX7A1* may facilitate these studies as well as a detailed examination of the epigenetic regulation of the EFT, thereby advancing our understanding of induced tissue regeneration (iTR) and oncogenesis.

## METHODS

### Human cell lines and samples microarray analysis

BioTime's clonal embryonic progenitor cell (EPC) lines 4D20.8, E3, and SK5 were generated by partial differentiation of hESCs (PTA8172, ATCC) followed by clonal expansion. Osteochondral lines 4D20.8 and MSC were differentiated by exposure to serum free medium containing TGFb3 10 ng/ml, lipogenic lines E3 and SAT were differentiated in serum free medium containing rosiglitazone 1 uM and T3 2 nM. The MSCs (hMSC-BM) were obtained from PromoCell, Heidelberg Germany. The subcutaneous preadipocytes and adult myoblasts were obtained from Zenbio, Triangle Park, NC and the sarcoma lines were obtained from ATCC. Cells were lysed with RLT buffer (Qiagen) containing b-mercaptoethanol and RNA was prepared using Qiagen mini kits following manufacturer's instructions, RNA concentrations were measured using a Nanodrop spectrophotometer and RNA integrity was determined by denaturing agarose gel electrophoresis or by an Agilent 2100 bioanalyzer. RNA expression was obtained using Illumina Human HT-12 v4 BeadArrays, and RNA expression magnitudes for certain genes were verified by qRT-PCR. In preparation for Illumina BeadArrays, total RNA was linearly amplified and biotin-labeled using Illumina TotalPrep kits (Life Technologies, Temecula, CA, USA). The cRNA quality was measured using an Agilent 2100 Bioanalyzer before being hybridized to Illumina BeadChips, processed, and read by an iScan microarray scanner according to the manufacturer's instructions (Illumina, San Diego, CA, USA). Values under 130 relative fluorescence units (RFUs) were considered as nonspecific background signal. Raw microarray data were normalized with the R BeadArray library. Analysis of microarray data was performed using the R lumi library.

### Data collection and integration for machine learning

We used data from public databases Gene Expression Omnibus (GEO) [53, 54] and ArrayExpress [55]. Each sample belongs to one of the following classes: embryonic stem cell (ESC), induced pluripotent stem cell (iPSC), embryonic progenitor cell (EPC), adult stem cell (ASC) and adult cell (AC). Samples in this study were obtained from the following microarray platforms: Illumina HumanHT-12 V4.0 (GPL10558), Illumina HumanHT-12 V3.0 (GPL6947), Affymetrix HT Human Genome U133A Array (GPL3921), Affymetrix GeneChip Human Genome U133 Array Set HG-U133A (GPL4557), Affymetrix Human Exon 1.0 ST Array (GPL5188), Affymetrix Human Genome U133 Plus 2.0 Array (GPL570), Affymetrix Human Genome U133A 2.0 Array (GPL571), Affymetrix Human Gene 1.0 ST Array (GPL6244), Affymetrix Human Genome U133A Array (GPL96), Affymetrix Human Genome U133 Plus 2.0 Array (GPL11670). The final number of samples used for training and validation were grouped by platform vendor and cell type and shown in Supplementary Table 4.

### Data processing for machine learning

We employed separate processing pipelines for Affymetrix and Illumina data. For the processing of Affymetrix data sets we utilized Frozen RMA (fRMA)[56] method, which allows the analysis of microarrays individually or in small batches and then combined the data for analysis. After obtaining probe expression data, we converted it to gene expression using annotation tables, available from GEO for Illumina platforms and ‘AnnotationDbi’ package from Bioconductor for Affymetrix platforms. Such tables contain probe-gene mapping for particular microarray platform. If multiple probes were mapped to the same gene, we used geometric mean to average their signals. After converting to genes, non-normalized datasets (separately for Affymetrix and Illumina platforms) were processed with quantile normalization algorithm. The samples to be classified were normalized using same set of quantiles as were determined for training dataset. We used gene expression values as input features for each Affymetrix and Illumina classifiers.

### Pathway analysis

For pathway activation analysis, we used iPANDA algorithm [17]. For each investigated sample group, we performed a case-reference comparison using Student's *t*-test and generate the list of significantly differentially expressed genes and calculate the Pathway Activation Strength (PAS) score for 367 pathways currently annotated, a value which serves as a qualitative measure of pathway activation. Positive and negative PAS values indicate pathway up and downregulation, respectively. In this study, we used 50 randomly chosen ESC samples as a reference group and the genes with FDR-adjusted *p*-value < 0.05 were considered significantly differentially expressed. After PAS values had been calculated for each sample they were used as an input for machine learning algorithm training and validation.

### K-nearest neighbors algorithm (kNN)

K-nearest neighbors algorithm is a simple non-parametric method, that can be applied to regression. The underlying idea of the method is to predict a value of a given object as an average of the values of its *k* nearest neighbors. The choice of optimal k is defined by the properties of the data. In the current study, we used the scikit-learn implementation of the method [57]. Hyperparameters tuned were the number of neighbors to use (5–20), the neighbor weighting (uniform of inversely proportional to their distance), and metric (Manhattan, Euclidean, or Minkowski with *p* = 3).

### Logistic regression (LR)

Logistic regression is a widely used straightforward approach to model the dependence of a given variable *Y* on a set of independent variables *X_i_*. In the current study we used scikit-learn implementation [57]. First, we reduced data dimensionality using Principal Component Analysis with whitening, and then trained multiclass classifier with L_2_-regularization. Hyperparameters tuned were the number of principal components (100–500), and regularization strength (0.1–100).

### Support vector machines (SVM)

SVM is another classical machine learning algorithm, which, in its basic form, constructs a set of hyperplanes separating multidimensional data into classes. The use of non-linear kernels allows SVM to perform non-linear classification. In the current study we used the scikit-learn implementation of the method [57]. Hyperparameters tuned were the type of kernel (linear, sigmoid, 3rd-degree polynomial, and radial basis function [Gaussian] kernels), and regularization strength (0.1–100).

### Gradient boosting machines (GBM)

Gradient boosting is a machine learning method used for classification and regression problems. This method uses an ensemble of weak models, like classification trees in this case, to generate predictions. We used XGBoost library [53] to implement gradient boosting classifier. Hyperparameters tuned were the number of trees grows (10–100), maximal depth of each tree (3–8), subsampling ratio (0.5–1.0), regularization parameters gamma (further partitioning threshold, 0.5–1) and minimal child weight (1–5), and step size shrinkage (0.005–0.05).

### Multiclass deep neural network (DNN)

The number of input layer neurons was equal to the number of genes used. Hyperparameters tuned were the number of hidden layers (2–4), the number of neurons in each hidden layer (100–500), activation function for all layers except output one (ReLU, sigmoid, or tanh), L_2_ weight-regularization strength (0.01 to 0.05), and dropout value (0.0 to 0.5). The output layer used softmax activation. The neural network was trained for 200 epochs using Adam optimizer^51^.

### Ensemble of deep neural networks (DNN ens)

The design of each network was similar to multiclass network, except the output layer had only one neuron with sigmoid activation. Since running hyperparameter optimization for DNN ensemble is very computationally expensive, each network used the set of hyperparameters identified as optimal for multiclass network: 2 layers of 200 neurons, ReLU activation, 0.2 dropout, and 0.03 L_2_ weight regularization strength. We trained 20 binary networks for each target platform set (Affymetrix and Illumina) to perform pairwise (one-vs-one) classification. Then we evaluated the overall ensemble vote for each class as the sum of four one-vs-one networks, which perform pairwise distinction of this class from four other classes.

### Training classifiers

The majority of the deep learning experiments presented in this research paper were performed in 2015 and utilized the state of the deep learning techniques available at that time. To train the deep neural networks on the chosen datasets, we employed the following scheme (Supplementary Figures 1 and 2). First, we preprocessed the datasets (gathered from public data repositories, as well as the one provided by BioTime, Inc.) to convert the probe data into genes, and apply quantile normalization. Afterwards, we employed a nested cross validation approach to tune hyperparameters and obtain an unbiased estimation of classifier performance. Both outer and inner loops used stratified labeled 3-fold cross validation, with samples from same dataset belonging to either training or validation set, but not both. In outer loop, we held out a part of the data, and used the remaining samples to optimize the classifier hyperparameters. We then verified that hyperparameters were not overfit by the training classifier with found optimal hyperparameters, and tested it on the held out data. The hyperparameter tuning was repeated for each fold. This result was designated “Ext. validation”. We used Tree of Parzen Estimators (TPE) algorithm (as implemented in hyperopt package [55]) to optimize hyperparameters. For each parameter set attempted, we ran 3-fold cross validation, and used mean validation score as optimization target. For best hyperparameter set, we presented its mean performance on training (“Training”) and validation (“Int. validation”) sets in internal cross validation loop. We only presented training and validation scores for the DNN ensemble, since we did not run hyperparameter estimation for it due to the high computational cost.

### Determining sample's embryonic score

To determine how close the sample is to the embryonic state we used an ensemble of deep neural network predictors, built upon one of the proposed approaches (Supplementary Figure 3). The sample to be classified was subjected to same preprocessing protocol as training samples from appropriate platform. The genes were supplied to trained deep neural network predictors’ input. Our DNN ensemble produced five ensemble votes - one for each class - which we used to calculate the Embryonic Score (ES) as follows:

$$\frac{\sum_{i=1}^{5}Class{\;}_{i}w_{i}}{\sum_{i=1}^{5}Class{\;}_{i}w}$$

where *Class*_1–5_ is the ensemble vote for each class, and w_1–5_ - are arbitrary degrees of embryonic development for chosen classes (we assign w_ESC_ = 1.0, w_iPSC_ = 0.9, w_EPC_ = 0.7, w_ASC_ = 0.5, w_AC_ = 0.0). As a result, the system outputs calculated embryonic score for each sample. In order to find out what genes were good markers of each stage of cell development, we used a previously developed method [58] that allows estimation of the importance of each feature directly from DNN's weight matrices. This method measures the magnitude by which every input feature was propagated all the way to the output layer. For verification, we measured gene importance from trained multiclass GBM classifier by measuring how many times a particular feature is used to split a tree (f-score).

### Human cell line RNA-seq analysis

All human embryonic cell lines were derived from Human embryonic stem cell lines H9 (WA09; WiCell Research Institute, Inc.), ESI-017 (ESI BIO, Singapore) and MA03 (International Stem Cell Registry, UMASS Medical School). RNA extraction performed using Qiagen's RNeasy Mini Kit. RNA sequencing performed by on Illumina Hiseq 2500 machines, 100 bp paired end sequencing, and the resulting data analyzed by TUXEDO suite [59].

### Mouse embryo RNA-seq analysis

Total mouse embryo RNA was procured from Zyagen, Inc., San Diego, CA. RNA sequencing performed by on Illumina Hiseq 2500 machines, 100 bp paired end sequencing, and the resulting data analyzed by TUXEDO suite [59]. All samples were sequenced to the depth of 25 million 100 bp, paired end reads).

### *COX7A1* methylation analysis of EPCs and ACs

DNA extraction, bisulfite library construction and 90G (150 bp paired end) per sample sequencing on Illumina Hyseq 2500 machines performed by Beijing Genome Institute (BGI). Analysis performed using Bismark suite [60].

### Gene expression analysis of human tissues and cell lines

Human samples were obtained from Advanced Bioscence Resources, Alameda, CA (fetal samples) and Coriell Institute for Medical Research, Camden, NJ (adult samples). RNA extraction performed using Qiagen's RNeasy Mini Kit. All microarray expression profiling analyses performed on Illumina's HumanHT-12_V4_0_R2 microarray chips. Primary analysis performed using Illumina's GenomeStudio version 1.9.0. Data normalized and expression profiles determined using lumi package in R [61].

### Gene expression analysis of normal and cancer cell lines

Normal controls and the cancer cell tissues were purchased from Asterand Biosciences, Detroit, MI. RNA extraction performed using Qiagen's RNeasy Mini Kit. All microarray expression profiling analyses performed on Illumina's HumanHT-12_V4_0_R2 microarray chips. Primary analysis performed using Illumina's GenomeStudio version 1.9.0. Data normalized and expression profiles determined using lumi package in R [61].

### *COX7A1* methylation analysis using publicly available data

We utilized the following datasets found at GEO database: GSE49996 Lung adenocarcinoma, GSE58272 Squamous cell carcinoma, GSE73003 Hepatocellular carcinoma, and GSE35242 Prostate cancer cells. All datasets were obtained on Illumina HumanMethylation27 BeadChip platform. *COX7A1* and *RSP10* (housekeeping control) average beta values were calculated using Genome Studio software v2010.3 and plotted using Prizm 6 software.

### *COX7A1* expression analysis using publicly available data

The following large data sources had been used to evaluate *COX7A1* expression in cancer cell lines: Sarcoma project https://sarcoma.cancer.gov/sarcoma, FANTOM5 project http://fantom.gsc.riken.jp/5, GEO database https://www.ncbi.nlm.nih.gov/geo, referred as “Pooled GEO data”. Relative expression level of *COX7A1* for each data source was downloaded and plotted using Prizm 6 software.

### *COX7A1* expression analysis using BioTime proprietary sarcoma data

All sarcoma lines were obtained from ATCC. RNA extraction performed using Qiagen's RNeasy Mini Kit. All microarray expression profiling analyses performed on Illumina's HumanHT-12_V4_0_R2 microarray chips. Primary analysis performed using Illumina's GenomeStudio version 1.9.0. Data normalized and expression profiles determined using lumi package in R [61].

### Energy process: OCR and ECAR

OCR and ECAR of the various cell lines were obtained using a Seahorse XFp bioanalyzer (Agilent). Seahorse XF glycolysis stress test kits and mito stress test kits and reagents were used according to manufacturer's instructions. Data from cell lines were normalized by cell number which was obtained for each well of the 8 well XFp plates. Wave desktop 2.3 software (Agilent Technologies) was used to analyze the normalized results.

## SUPPLEMENTARY MATERIALS FIGURES AND TABLES






