# Supplementary material for: Use of deep neural network ensembles to identify embryonic-fetal transition markers: repression of COX7A1 in embryonic and cancer cells
Source: Oncotarget. 2017 Dec 28;9(8):7796–811. doi: 10.18632/oncotarget.23748 (PMC5814259; doi:10.18632/oncotarget.23748)
Supplement: Supplementary file 1 [file oncotarget-09-7796-s001.pdf]

Use of deep neural network ensembles to identify embryonic-fetal transition markers: repression of *COX7A1* in embryonic and cancer cells

SUPPLEMENTARY MATERIALS

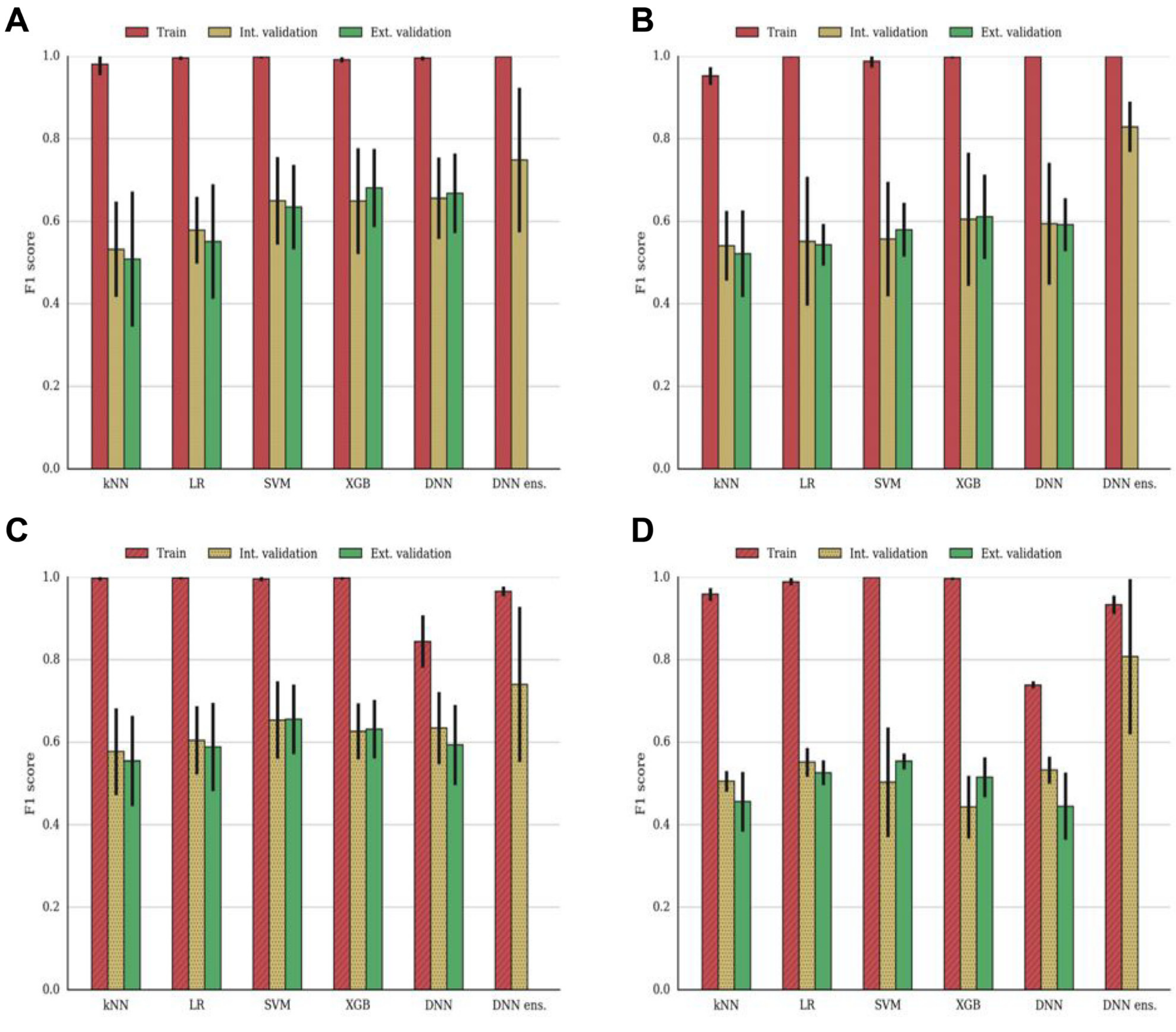

Supplementary Figure 1: Comparison of classifier performances. We compared F1 scores obtained on training, internal validation and external validation sets (see Methods section for detailed description of nested cross-validation protocol employed) of gene expression (**A**, **B**) and pathway activation score (**C**, **D**) input data from Affymetrix (A, C) and Illumina (B, D) platforms.

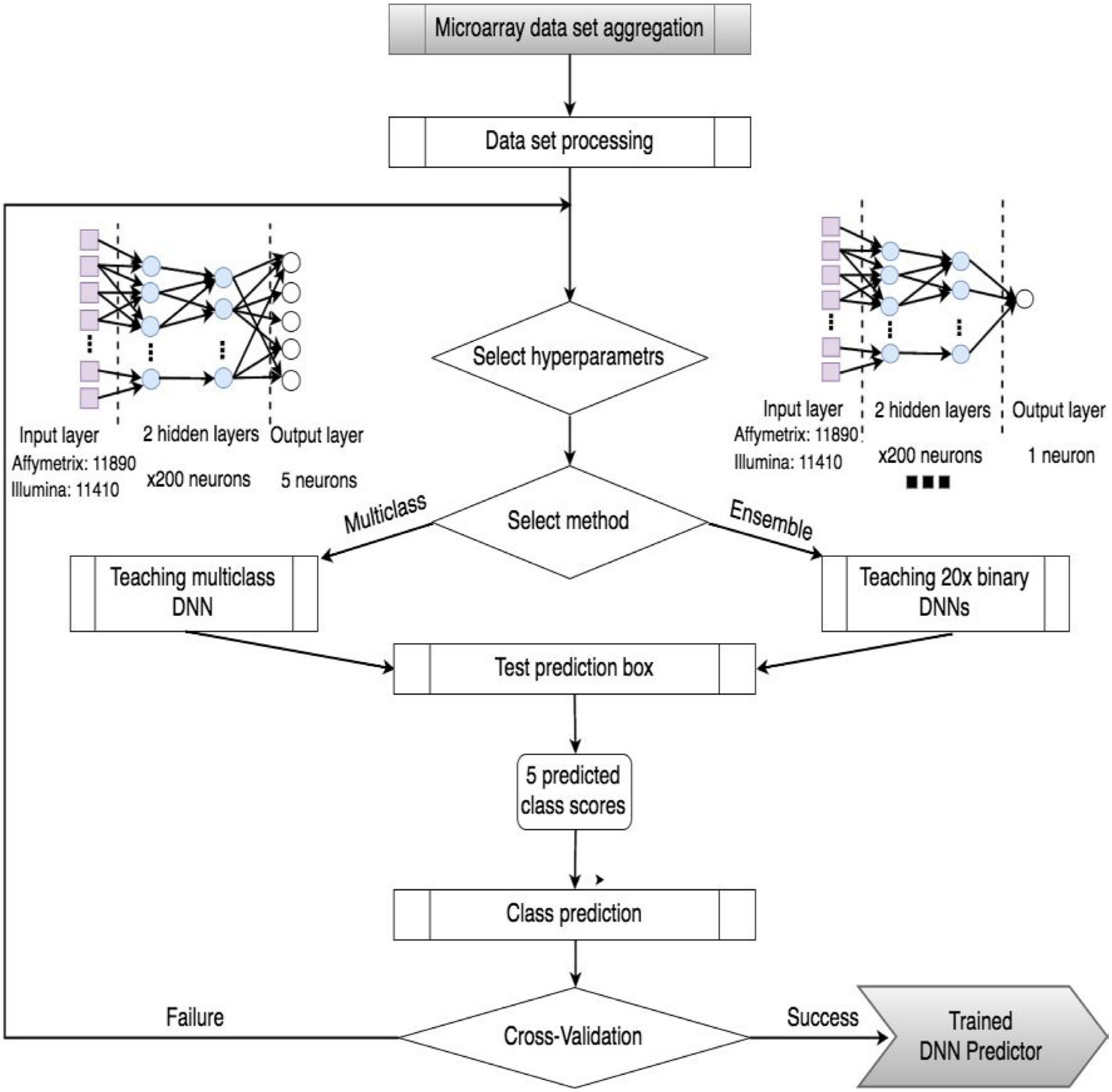

Supplementary Figure 2: The scheme for training of DNN predictor. Labelled transcriptomic data is used to train an ensemble of 20 deep neural networks with a single output neuron and a multiclass deep neural network with five neurons, one for each of five classes.

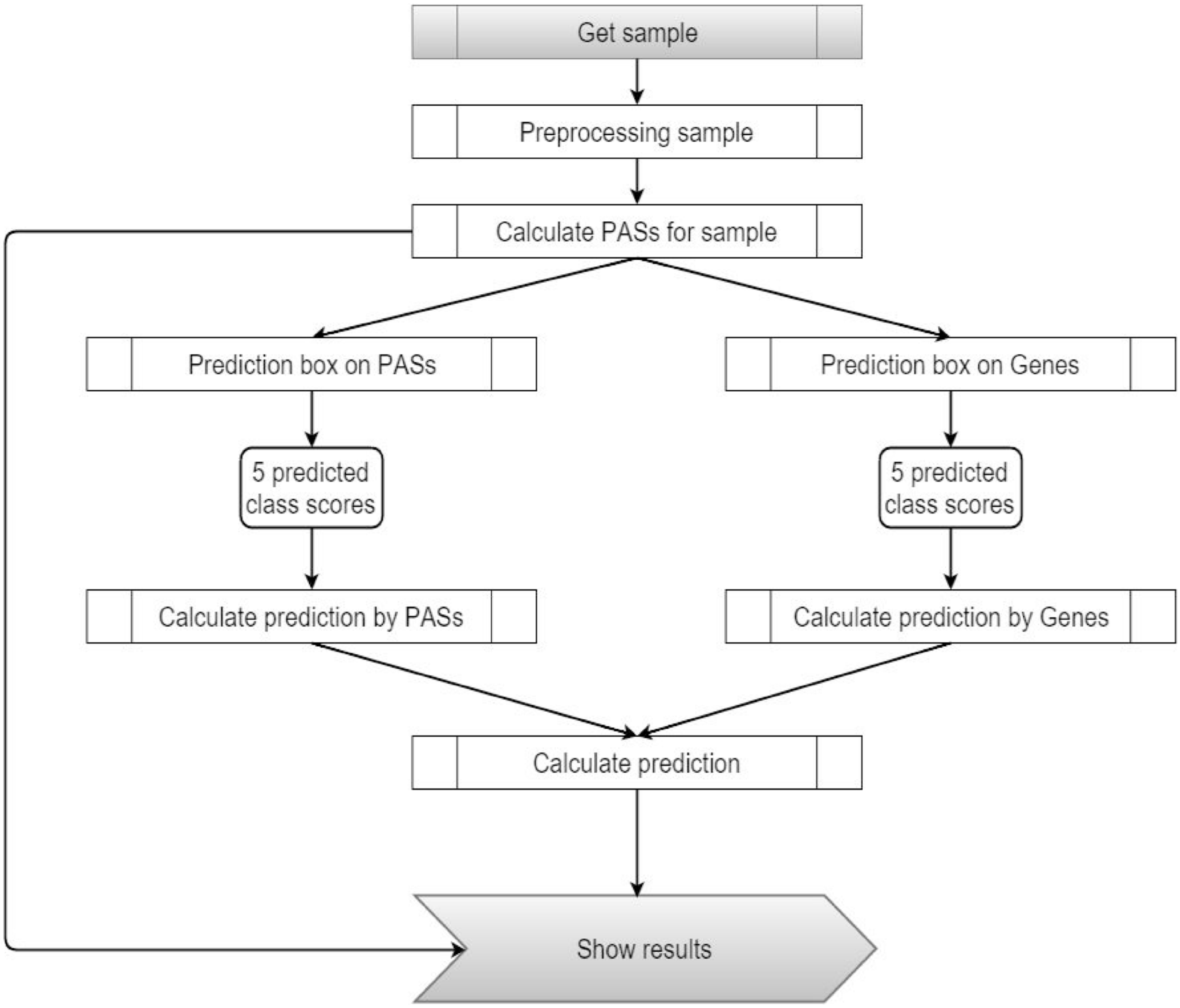

Supplementary Figure 3: The scheme for computing sample's embryonic score. For each input sample, the prediction is formed based on the results of gene- and pathway-level DNN ensembles. The final E score was calculated as an average of gene- and pathway-level embryonic score.

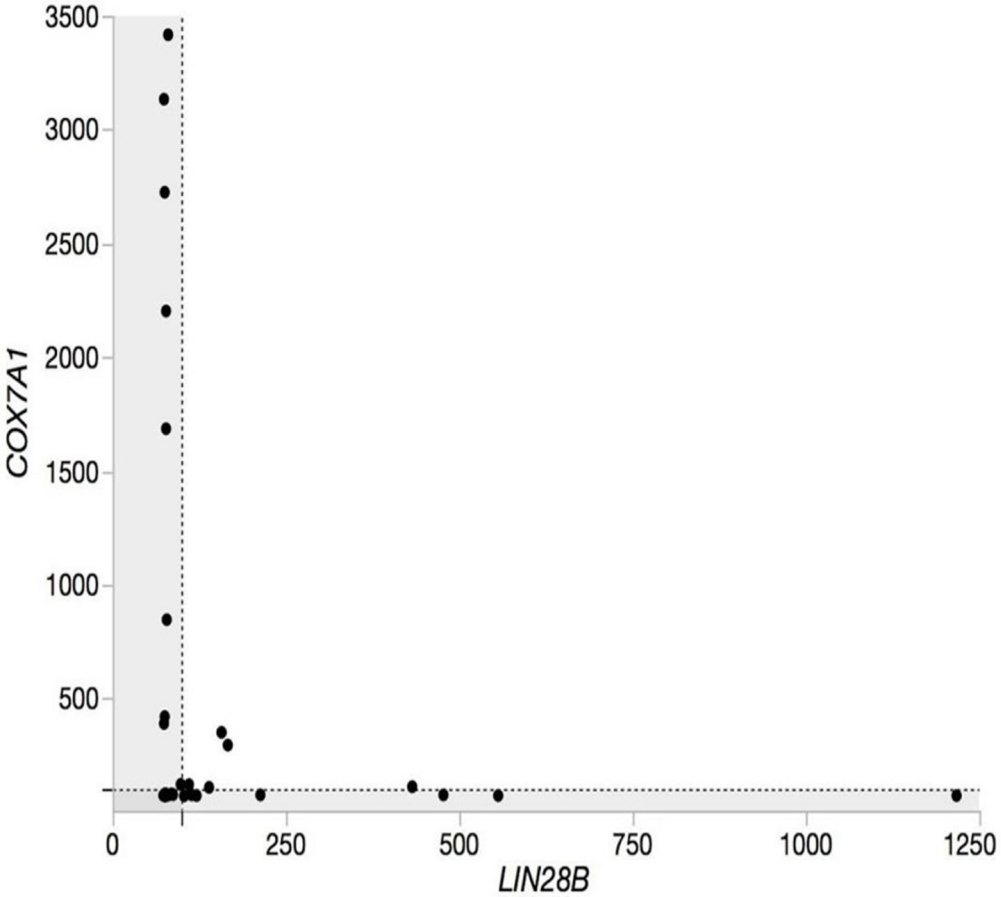

Supplementary Figure 4: Negative correlation between expression level of LIN28B vs COX7A1. Strong inverse agreement had been identified between expression level of LIN28B vs COX7A1, that is, when one gene was expressed the other gene was not, or both genes were not expressed. Expression was defined as a microarray hybridization signal greater than 100 RFUs, where the shaded regions highlight the strong inverse correlation of gene expression.

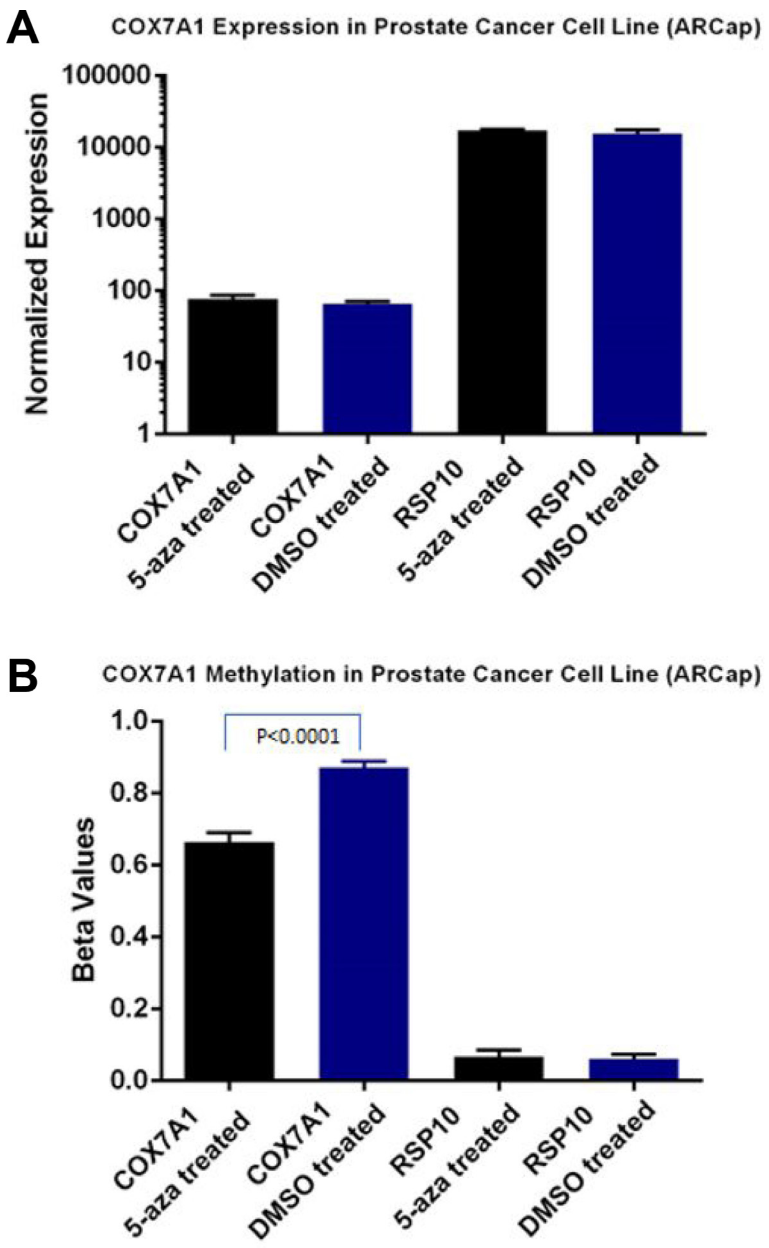

Supplementary Figure 5: COX7A1 methylation may not be involved in its regulation. Samples of prostate cancer cell line ARCap had been treated with 5-aza, and simultaneously subjected to expression and methylation analysis. (**A**) expression analysis did not reveal any differences in COX7A1 expression between demethylated (5-aza treated) and methylated cells. (**B**) Demethylation of COX7A1 genome region in 5-aza treated cells had been confirmed to be statistically significant. RPS10 gene had been utilized in both assays as housekeeping control.

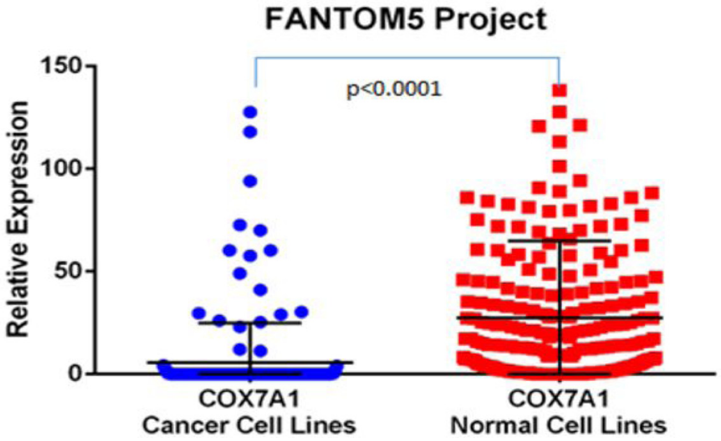

Supplementary Figure 6: Comparative analysis of COX7A1 expression in all cancer cell lines vs. all normal cell lines. Analysis by *t*-test demonstrated statistically significant decrease of COX7A1 expression in all cancer cell lines comparing to all normal cell lines. Normalized expression values of COX7A1 were calculated using transcriptomic data from Fantom5 Project – 564 samples total.

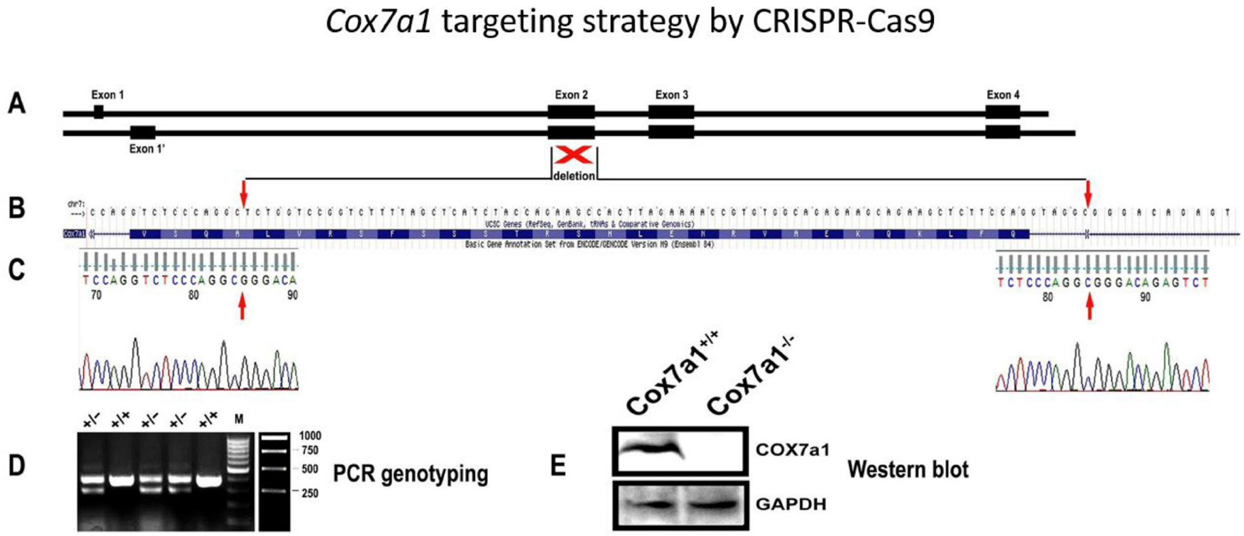

Supplementary Figure 7: Cox7a1 targeting strategy by CRISPR-Cas9. (**A**) Mouse Cox7a1 genomic locus. Exon2 was selected for CRISPR-Cas9-mediated deletion. (**B**) Nucleotide sequence around the Exon2 of Cox7a1, single base-pair resolution. Red arrows point to positions in Exon2, where CRISPR-Cas9-mediated excision took place. (**C**) Confirmation of CRISPR-Cas9 engineered deletion by sequencing. (**D**) PCR-genotyping of F1 animals. (**E**) Confirmation of Cox7a1 targeting by Western blot analysis. COX7A1 protein was not found in heart tissue of Cox7a1 −/− mice, whereas it is abundantly expressed in heart tissue of wild type (+/+) littermate mice.

Supplementary Table 1: Final hyperparameters

Method	Affymetrix	Illumina
kNN	k = 10, distance weighting, p = 3	k = 5, distance weighting, p = 2
LR	200 components, C = 0.34	200 components, C = 0.24
SVM	Linear kernel, C = 0.48	RBF kernel, C = 99.94
GBM	30 trees, depth = 5, subsample 0.8, gamma = 0.6, min_child_weight = 2, eta = 0.005	80 trees, depth = 6, subsample 0.5, gamma = 1.0, min_child_weight = 3, eta = 0.05
DNN	2 hidden layers, 100 neurons per layer, ReLU activation, dropout 0.2, L_2 0.03	2 hidden layers, 200 neurons per layer, ReLU activation, dropout 0.0, L_2 0.04

Supplementary Table 2: Gene ranking

Rank	Gene
1	***TSPYL5***
2	*ZNF280D*
3	***COMT***
4	***COX7A1***
5	*BMS1P5*
6	***TRIM4***
7	*PDXDC1*
8	*PLD6*
9	*ITPRIPL2*
10	***CAT***
11	***NAALADL1***
12	***ADIRF***
13	***PCDHB2***
14	*CTSF*
15	*FRG1B*
16	*FARSB*
17	*HSP90B3P*
18	*HNRNPH1*
19	*FOXD2*
20	*UBA1*
21	*QRFPR*
22	*ITGA8*
23	*TAF9B*
24	*ECHDC3*
25	*FOXN3*
26	*KIF18B*
27	*PPAPDC3*
28	*D2HGDH*
29	*MGMT*
30	*VPS26A*
31	*PKIB*
32	*RRP7A*
33	*BHMT2*
34	*DYNLT3*
35	*GSTM5*
36	*DNAJC28*
37	*SLC24A3*
38	*AHNAK2*
39	*KAT2B*
40	*ZNF738*
41	*RFX7*
42	*DENND1B*
43	*PIP4K2A*
44	*UNK*
45	*EPDR1*
46	*PSG3*
47	*SPESP1*
48	*POLA1*
49	***CYTH2***
50	*FAM175A*

Supplementary Table 3: Differential gene expression results. See Supplementary_Table_3

Supplementary Table 4: A list of samples used for training and validation. See Supplementary_Table_4
