# Supplementary material for: Use of deep neural network ensembles to identify embryonic-fetal transition markers: repression of COX7A1 in embryonic and cancer cells
Source: Oncotarget. 2017 Dec 28;9(8):7796–811. doi: 10.18632/oncotarget.23748 (PMC5814259; doi:10.18632/oncotarget.23748)
Supplement: Supplementary file 2 [file oncotarget-09-7796-s002.doc]

entrez_id,gene_symbol,4D20.8 P15 ctrl RS_44,7PEND24 P21 ctrl RS_49,7SMOO32 P11 ctrl RS_46,30-MV2-6 P6 RS_70,30-MV2-7 P6 RS_77,C4ELS5.1 P14 ctrl RS_53,E3 P12 ctrl RS_54,E15 P19 ctrl RS_47,E69 P16 ctrl RS_52,EN13 P14 ctrl RS_43,MEL2 P17 ctrl RS_50,ESI004 NP88 SM P12 ctrl RS_56,ESI004 NP110 SM P12 ctrl RS_55,RP1-SKEL-8 P6 RS_84,SK11 P16 ctrl RS_48,SM30 P15 ctrl RS_45,T42 P17 ctrl RS_51,NHA (normal human astrocytes Lonza) P3 ctrl RS_35,NHAC P6 RS_18,Normal human arm skin fibroblast 82 yr (Coriell GM01706 A) P12 RS_85,"HAEC tRNA (Human Aortic Endothelial Cell total RNA), 10 μg RS_13",HMVEC P6 RS_19,Normal human hepatocyte P5 64 yr old (Zenbio-HPNP2 lot ZBH2134) RS_86,NHEM (Lonza neonatal human epidermal melanocytes) P7 ctrl RS_37,MSC P5 RS_27,Skeletal myoblasts Zenbio P5 ctrl RS_30,NHOST Lonza normal human osteoblasts) P5 ctrl RS_39,Brain Pericytes P6 RS_25,Fetal brown preadipocytes (Zenbio) P4 ctrl RS_29,Subcutaneous preadipocytes Zenbio (Lot SLOO54) P6 ctrl RS_32,HSC human schwann cell P2 RS_22,HAoSMC (aortic smooth muscle PromoCell) lot 4002012.2 P5 ctrl RS_42,meanProgenitors,meanAdult,pvalue,FDR-corrected p-value

,,Progenitors,Progenitors,Progenitors,Progenitors,Progenitors,Progenitors,Progenitors,Progenitors,Progenitors,Progenitors,Progenitors,Progenitors,Progenitors,Progenitors,Progenitors,Progenitors,Progenitors,Adult,Adult,Adult,Adult,Adult,Adult,Adult,Adult,Adult,Adult,Adult,Adult,Adult,Adult,Adult,,,,

338707,B4GALNT4,22.59,43.04,19.34,25.03,18.93,14.96,6.96,28.48,18.88,28.93,11.86,27.41,20.52,24.25,20.22,23.53,10.1,13.62,1.23,0.36,0.16,0.23,0.04,1.49,1.78,0.42,0.48,10.39,2.23,0.85,8.89,0.68,21.47235294,2.856666667,1.06E-07,0.001154117

153020,RASGEF1B,0,0,0,0,0,0,0,0,0,0,0,0,0,0,0,0,0,0.55,0.03,0.11,0.34,0.04,6.2,0.46,0.09,0.36,0.11,0.09,0.09,0.06,0.06,0.04,0,0.575333333,1.95E-07,0.001154117

246777,SPESP1,0,0,0,0,0,0,0,0,0,0,0,0,0,0,0,0,0,0.5,2.62,0.79,1.49,1.49,0.2,3.31,0.07,1.15,0.29,1.09,1.48,1.54,1.21,1.24,0,1.231333333,1.98E-07,0.001154117

84418,CYSTM1,10.43,1.34,5.59,5.99,8.44,8.27,16.59,6.95,5.16,4.81,19.46,19.05,7.82,5.04,5.58,9.36,5.96,19.71,13.42,25.07,31.85,47.48,30.86,113.56,25.05,20.56,30.27,13.54,18.11,24.33,21.57,43.5,8.578823529,31.92533333,2.37E-07,0.001154117

100506243,KRBOX1,0,0.09,0,0,0,0,0,0,0,0,0,0,0,0,0,0,0,2.61,3.07,2.29,0.43,0.13,0.42,0.39,1.51,2.85,3.39,4.25,4.23,2.83,2.79,0.53,0.005294118,2.114666667,2.96E-07,0.001154117

1346,COX7A1,0,0.08,0,0,0,0,0.32,0,0,0.26,0,0,0,0,0,0,0,2.42,35.44,21.12,13.52,42.45,0.56,5.84,28.04,106.74,1.9,33.76,21.8,23.36,16.83,35.99,0.038823529,25.98466667,5.46E-07,0.001774618

100130418,CECR7,0,0,0,0,0,0,0,0,0,0,0,0,0,0,0,0,0,0.06,1.4,2.44,0,0.06,0.05,0.57,0.72,0.15,0.09,0.1,1.37,0.74,0.97,0.06,0,0.585333333,7.95E-07,0.002099937

10069,RWDD2B,6.36,4.02,4.49,0.05,0,6.68,5.75,8.27,5.36,4.31,6.85,4.69,3.26,0.06,2.98,4.04,8.07,9.32,8.14,11.27,15.5,6.31,10.68,13.04,11.23,12.3,8.12,6.06,11.18,10.35,9.55,8.81,4.425882353,10.124,9.62E-07,0.002099937

10004,NAALADL1,0,0,0.02,0,0,0,0,0,0,0.42,0.11,0,0,0.04,0,0,0,0.75,4.14,2.58,1.52,0.52,0.05,3.87,3.23,0.66,1.68,3.31,3.6,3.41,4.45,4,0.034705882,2.518,1.03E-06,0.002099937

120892,LRRK2,0.02,0,0,0,0,0.02,0.12,0,0.01,0,0.2,0,0.04,0,0,0.01,0,0.23,0.53,4.93,0.56,0.98,0.6,0.71,2.32,0.65,1.17,2.64,0.36,2.89,1.07,2.02,0.024705882,1.444,1.11E-06,0.002099937

5178,PEG3,0,0,0,0,0,0,0.11,0,0.01,0,0,0,0,0,0.01,0,0,1.45,0.23,0.65,0.13,0.53,1.13,0.04,0.07,0.24,0.01,0.88,1.29,0.74,2.5,0.41,0.007647059,0.686666667,1.21E-06,0.002099937

100132288,TEKT4P2,0,0,0,0.36,0.28,0,0,0,0,0.15,0,0,0,0,0,0,0,0.95,3.02,8.02,0.25,2.46,1.29,1.67,1.97,1.46,2.57,2.85,0.37,3.15,0.06,4.12,0.046470588,2.280666667,1.49E-06,0.002099937

26025,PCDHGA12,0.06,0.05,0.05,0.08,0.07,0.03,0.03,0.03,0,0.11,0.12,0.03,0.25,0.15,0,0,0,7.46,16.1,12.32,4.38,2.83,0.39,16.04,43.21,4.19,19.38,16.3,8.02,13.74,27.68,11.87,0.062352941,13.594,1.55E-06,0.002099937

56144,PCDHA4,3.37,1.1,2.85,2.94,4.04,3.83,1.57,0.9,3.69,0.88,9,7.19,2.76,3.55,0.7,2.43,2.08,0.34,0.11,0,0.2,0.2,0.07,0.1,0.47,0.07,0,0.16,0.03,0.11,0.15,0.07,3.110588235,0.138666667,1.60E-06,0.002099937

268,AMH,7.68,5.66,6.33,8.18,13.08,5.77,2.42,7.61,6.81,6.46,4.81,3.03,2.03,13.94,18.07,6.84,10.54,0.46,0.16,0.66,0.31,0.13,0.2,0.34,1.01,1.27,0.77,0.63,0.51,0.36,0.31,0.93,7.603529412,0.536666667,1.62E-06,0.002099937

93517,SDR42E1,0,0,0,0.06,0,0,0.03,0,0,0,0,0,0,0,0,0,0,0.11,1.41,0.11,2.49,0.28,2.96,0,0.88,0.09,0.77,0.16,0.42,0.48,0.54,0.47,0.005294118,0.744666667,2.11E-06,0.002427982

83938,C10orf11,0,0.76,0,0,0,0.17,0.18,0,0,0.33,0,0,0,0.11,0,0,0.11,0.46,4.48,0.83,5.8,3.59,3.91,14.24,4.5,0.57,1.35,0.99,0.69,1.56,0.74,1.93,0.097647059,3.042666667,2.12E-06,0.002427982

847,CAT,0,0.03,0.03,0.03,0,0,9.93,11.69,6.46,0.21,0.07,0,0,18.04,0,0,0,11.71,25.13,39.12,29.65,18.2,25.79,46.34,32.84,23.51,20.5,48.18,11.28,32.04,34.8,33.41,2.734705882,28.83333333,2.35E-06,0.002511652

150763,GPAT2,0,0,0,0.08,0.14,0.02,0.08,0.09,0,0.43,0.08,0,0,0.1,0.03,0.13,0.03,0.27,2.64,1.67,1.84,0.4,0.25,3.31,2.97,0.97,0.84,0.89,1.69,4.23,0.66,10.94,0.071176471,2.238,2.60E-06,0.002511652

220594,USP32P2,0.05,0.02,0.13,0.26,0.07,0.06,0.08,0,0.09,0.05,0.18,0.12,0.04,0.24,0.05,0.04,0.07,0.46,3.67,0.15,0.62,0.37,1.04,1.84,3.58,1.16,1.38,2.81,2.12,1.51,3.2,4.17,0.091176471,1.872,2.80E-06,0.002511652

389813,C9orf172,3.11,3.65,1.64,1.65,1.75,2.37,1.88,4.31,3.6,2.19,2.7,2.61,2.19,2.75,4.15,4.83,3.46,0.72,1.85,1.24,0.12,0.68,0.29,0.71,1.04,1.55,1.44,1.37,1.3,0.91,1.07,1.33,2.872941176,1.041333333,2.83E-06,0.002511652

284123,FAM27E5,0,0,0,0,0,0,0,0,0,0,0,0,0,0,0,0,0,0.14,0.48,1.41,0.5,0,0,0.46,1.06,0.58,0.29,0.58,0.71,0.83,0.23,1.54,0,0.587333333,3.00E-06,0.002511652

122402,TDRD9,0,0,0,0,0,0,0,0,0,0,0,0,0,0,0,0,0,0.08,1.26,0,0.45,0,0.12,1.07,1.14,1.39,0.34,0.04,0.31,0.47,1.08,0.25,0,0.533333333,3.01E-06,0.002511652

150381,PRR34-AS1,0,0,0,0,0,0,0.67,0,0,0.06,0,0,0,0,0,0,0,0.38,0.49,0.49,1.28,1.93,1.2,1.33,0.42,1.9,0.71,0.04,0.24,0.29,0.31,0.37,0.042941176,0.758666667,3.09E-06,0.002511652

729857,RGPD2,1.98,5.24,2.57,4.73,2.8,2.73,0.84,1.43,1.25,3.74,3.1,1.88,1.13,4.7,2.51,3.46,2.14,0,0.04,0.9,0.37,1.42,0.1,0.14,0.38,0,0.08,0.3,0,0.04,0,0.17,2.719411765,0.262666667,3.33E-06,0.002546261

8641,PCDHGB4,53.15,12.43,21.78,35.64,14.25,36.53,18.9,61.3,12.89,15.16,114.78,31.11,24.14,19.31,43.51,37.91,9.89,4.01,5.72,5.69,2.23,6.7,1.43,2.86,14.54,1.47,7.25,2.23,2.98,3.62,7.95,6.24,33.09882353,4.994666667,3.40E-06,0.002546261

56146,PCDHA2,2.17,0.3,0.46,1.15,0.74,0.85,0.61,0.79,0.85,0.07,5.63,0.16,0.28,0.78,0.8,1.87,1.13,0.03,0,0,0,0.37,0,0,0.27,0,0,0,0,0.05,0.02,0.03,1.096470588,0.051333333,3.81E-06,0.002659619

57683,ZDBF2,4.84,4.19,3.88,3.73,6.4,2.63,5.32,3.04,2.01,4.84,4.26,3.26,4.07,2.28,4.3,4.32,3.54,1.42,2.38,1.9,1.82,1.38,0.56,1.67,1.71,2.24,1.6,1.47,1.64,1.71,1.85,2.36,3.935882353,1.714,4.07E-06,0.002659619

23743,BHMT2,0.46,0.57,0.53,0.85,0.57,0.43,0.35,0.97,0.71,0.65,0.49,0.6,0.33,0.95,0.58,0.49,0.54,1.48,0.74,9.28,1.27,1.65,53.36,0.92,2.96,1.46,1.1,1.54,1.98,6.4,1.55,1.72,0.592352941,5.827333333,4.07E-06,0.002659619

134429,STARD4,13.77,16.41,21.94,9.53,9.72,12.39,17.05,11.26,18.69,12.16,9.22,9.19,23.98,12.51,14.36,29.37,15.56,6.88,2.71,4.05,10.74,10.58,7.89,3.1,7.54,11.77,4.7,7.53,9.1,5.13,10.32,7.15,15.12411765,7.279333333,4.27E-06,0.002659619

222698,NKAPL,0.04,0,0,0,0,0,0.61,0.4,0,0,0,0,0,0,0,0,0,0.61,0.57,0.62,0.71,0.47,0.25,0.89,0.65,0.29,0.6,1.09,0.99,0.52,1.59,0.1,0.061764706,0.663333333,4.32E-06,0.002659619

202243,CCDC125,0.29,0.29,0.34,0.82,0.78,0.24,0.2,0.32,0.21,0.44,0.4,0.17,0.22,1.31,0.51,0.33,0.35,2.63,1.31,1.07,2.42,0.84,3.23,0.65,3.13,1.61,1.69,1.92,1.86,1.67,2.61,1.38,0.424705882,1.868,4.45E-06,0.002659619

8653,DDX3Y,0,0,0,0,0,0,0.09,0,0,0.1,0,0,0,0,0,0,0,0.05,26.77,0.02,0.03,0.02,12.41,17.09,0.15,11.85,0.02,29.55,15.72,0.03,16.87,19.38,0.011176471,9.997333333,4.50E-06,0.002659619

6192,RPS4Y1,0,0,0,0,0,0,0.81,0,0,0.33,0,0,0,0.11,0,0,0,0.81,174.12,0.09,0.16,0.2,78.75,223.06,0.57,297.93,0.47,405.17,211.13,0.23,250.95,365.72,0.073529412,133.9573333,5.21E-06,0.002688307

56112,PCDHGA3,0.16,0.09,0,0.03,0.04,0.03,0.04,0.07,0,0.16,0.34,0.13,0.45,0.08,0.07,0.19,0,3.01,1.81,1.08,0.62,0.84,0.61,0.74,0.13,0.44,2.04,1.14,0.94,0.92,3.03,1.38,0.110588235,1.248666667,5.26E-06,0.002688307

1375,CPT1B,12.69,7.55,7.86,25.12,30.35,8.94,3.57,9.61,9.49,7.97,9.75,6.18,4.95,9.24,8.51,12.42,13.83,1.22,1.77,2.58,0.99,0.24,7.02,20.08,2.29,1.08,1.79,2.56,1.54,1.41,2.19,2.38,11.06058824,3.276,5.62E-06,0.002688307

23467,NPTXR,1.85,5.11,6.36,1.57,1.04,5.65,3.85,3.38,9.85,9.15,14.16,6.55,26.4,10.61,6.24,3.83,5.74,3.04,3.05,2.27,0.47,0.87,0.19,1.3,2.44,1.5,1.28,2.16,0.69,0.26,1.02,0.61,7.137647059,1.41,5.62E-06,0.002688307

112752,IFT43,15.04,12.92,9.97,10.24,15.43,14.13,14.96,15.78,8.99,15.06,11.05,12.12,12.03,11.82,9.88,14.55,11.27,17.65,19.8,14.09,22.82,16.25,34.15,17.21,18.12,22.24,16.24,15.3,18.79,17.57,15.75,12.88,12.66117647,18.59066667,5.62E-06,0.002688307

4255,MGMT,5.19,0,5.24,0,4.74,2.02,0.19,4.87,6.73,6.6,8.6,0,0,10.01,2.54,0,4.06,9.43,12.47,5.79,13.64,6.75,18.02,32.23,16.69,13.38,12.73,20.31,12.62,16.22,24.45,24.83,3.575882353,15.97066667,5.63E-06,0.002688307

5443,POMC,0,0,0,0,0.06,0,0,0,0,0,0,0,0,0,0,0,0,0.64,0.22,0.52,1.84,0.36,0.12,1.35,0.65,0,0.14,0.37,0.43,0.11,0.93,0,0.003529412,0.512,5.66E-06,0.002688307

2996,GYPE,0,0,0,0,0,0,0,0,0,0,0,0,0,0,0,0.04,0,0,0.68,2.61,0.2,0.05,0,0.1,0.56,0.6,0.09,1.08,0.78,1.86,0.39,1.83,0.002352941,0.722,5.66E-06,0.002688307

643529,LINC00865,0,0,0,0,0,0,0,0,0,0,0,0,0.02,0,0,0,0,0.68,0.14,0.07,0.18,0.02,0,1.23,1.76,0,0.34,1.16,0.42,0.26,2.26,0.12,0.001176471,0.576,6.23E-06,0.002889123

388697,HRNR,0.01,0,0,0,0,0,0,0.02,0.03,0,0,0,0,0,0,0,0,0.03,0.09,0.09,0.04,0,0.01,0.3,0.07,0.03,0.03,0.05,0.22,0.08,0.15,0.11,0.003529412,0.086666667,6.48E-06,0.002936219

149837,LINC00654,0,0,0.02,0,0,0.02,0,0.09,0,0,0,0,0,0.4,0.07,0,0,1.12,1.43,1.65,1.43,0.34,0,2.04,2.35,2.57,1.51,1.54,2.04,2.05,0.99,1.22,0.035294118,1.485333333,6.91E-06,0.003058827

7026,NR2F2,103.21,96.28,46.09,80.25,125.45,130.2,43.47,104.33,55.72,72.21,61.29,74.86,47.96,51.52,78.91,135.12,110.58,10.51,0.81,7.57,36.27,32.3,4.06,7.43,33.25,8.33,10.32,73.01,21.44,18.95,69.87,61.58,83.37941176,26.38,7.32E-06,0.003100636

9643,MORF4L2,127.76,214.01,243.23,186.38,170.63,233.79,143.34,277.65,222.26,220.67,165.28,207.26,163.91,332.13,135.42,86.1,141.8,134.08,91.09,76.49,113.47,101.56,145.02,82.85,83.37,144.38,93.77,93.23,110.81,78.91,113.29,85.32,192.4482353,103.176,7.32E-06,0.003100636

56127,PCDHB9,3.32,2.7,1.41,2.42,2.92,1.18,1.35,3.09,1.31,1.88,2.09,2.87,4.26,2.07,3.26,1.44,1.8,1.38,1.08,1.19,0.58,0.77,0.58,0.87,0.57,1.46,0.96,0.64,0.77,0.58,0.98,1.08,2.315882353,0.899333333,8.26E-06,0.003387942

55384,MEG3,0,0.08,0,0,0.16,0,223.82,0,0,0.2,0,0,0,0,0,0,0,99.23,367.52,31.88,19.5,40.67,2.34,0.39,170.97,28.76,78.29,196.5,50.94,50.05,412.15,26.06,13.19176471,105.0166667,8.34E-06,0.003387942

205251,LINC00116,0,0,0,1.89,1.77,0,2.34,4.49,3.64,0,0,1.99,2.28,2.09,0,0,0,4.07,2.97,6.14,5.73,4.24,4.9,4.93,4.77,8.78,5.48,2.27,5.05,4.18,2.7,6.36,1.205294118,4.838,9.45E-06,0.003485452

79899,PRR5L,16.38,11.62,10.35,17.06,19.62,7.52,4.56,9.5,15.88,14.8,2.45,6.92,1.32,4.23,7.67,25.61,13.8,5.47,0.77,1.15,2.95,5.66,0.49,0.42,4.71,0.87,2.63,1.54,4.03,1.07,2.37,0.57,11.13470588,2.313333333,9.48E-06,0.003485452

100,ADA,20.45,25.7,13.51,17.09,16.04,171.76,6,13.23,15.22,11.17,69.8,14.31,18.31,18.55,130.51,152.26,13.57,2.94,0.83,5.97,11.9,14.65,2.18,1.91,7.92,2.95,3,5.39,4.4,8.1,16.19,13.16,42.79294118,6.766,9.48E-06,0.003485452

157378,TMEM65,8.45,10.93,9.73,20.46,21.51,3.59,5.95,10.21,6.43,9.55,9.09,15.37,15.91,8.29,4.7,9.43,5.02,3.33,2.43,4.06,5.7,5.41,2.09,3.95,5.63,4.1,3.23,3.21,3.5,3.73,6.66,4.72,10.27176471,4.116666667,9.48E-06,0.003485452

84514,GHDC,1.98,4.7,2.69,0.59,5.82,0.15,1.7,12.14,5.1,5.18,12.77,0.24,0.33,4.41,0.52,0.18,0.29,5.55,17.77,10.09,9.71,6.07,6.13,28.29,16.95,5.94,10.85,16.23,6.27,12.41,7.91,13.49,3.458235294,11.57733333,9.48E-06,0.003485452

3226,HOXC10,0,0,0,0,0,0,0,0,0,0,0,0,0,0,0,0,0,0.04,15.94,0.48,0.1,0,0,10.14,46.04,19.22,10.49,0.03,0.03,21.48,10.22,0,0,8.947333333,1.04E-05,0.003763192

8722,CTSF,0,0,0,10.92,8.39,0,14.52,0.04,0,0.52,0,0,0,66.05,0,0,0,39.78,78.14,96.2,30.89,9.06,8.88,322.14,80.42,22.41,44.85,57.88,26.16,109.87,47.76,105.87,5.908235294,72.02066667,1.11E-05,0.003935105

56133,PCDHB2,19.96,13.65,19.23,27.49,24.03,5.12,7.25,0.82,4.12,9.56,42.28,1.32,1.21,16.84,15.19,12.85,20.65,3.69,1.75,2.61,0.25,3.94,0.15,0.11,0.68,2.47,0.65,0.89,0.7,1.36,0.72,1.26,14.21,1.415333333,1.22E-05,0.004126775

51063,CALHM2,0.24,2.71,2.75,0.88,0.6,0.85,0.25,30.32,10.16,0.26,5.6,0.09,0.49,0.48,0.28,0.32,0,4.24,25.01,41.95,10.49,8.28,1.86,15.34,19.66,7.79,19.68,9.78,22.34,46.38,15.36,35.77,3.310588235,18.92866667,1.22E-05,0.004126775

2312,FLG,0.02,0,0,0,0,0,0,0.17,0.04,0.01,0,0,0.01,0,0.01,0,0,1.97,0.22,0.99,0.33,0.41,0,2.26,0.76,0.1,8.5,0.06,1.05,0.71,0.49,0.11,0.015294118,1.197333333,1.27E-05,0.004126775

56102,PCDHGB3,0.15,0,0.05,0.38,0,0,0.07,0.14,0,0.08,0.22,0.37,0.74,0.2,0,0,0,1.56,2.43,1.87,0.31,0.68,0.12,0.83,2.52,0.96,2.72,1.98,1.33,1.18,2.96,0.73,0.141176471,1.478666667,1.27E-05,0.004126775

339834,CCDC36,0,0.02,0.02,0.03,0,0.03,0,0,0,0.04,0,0,0,0.4,0,0,0,0.64,0.67,1,0.26,0.06,0,1.03,1.13,0.3,1.26,0.58,1.02,1.24,1.37,0.9,0.031764706,0.764,1.27E-05,0.004126775

729085,FAM198A,0,0,0,0,0.02,0,0,0,0,0,0,0,0,0.03,0,0,0,0.13,0.52,0.21,0,0,13.38,0.05,0.14,0.02,0.19,1.31,0.68,0.42,0.27,0.85,0.002941176,1.211333333,1.29E-05,0.004126775

5646,PRSS3,0,0,0.08,0.1,0.2,0,0.1,0.22,0.11,0,0.1,0,0,0,0.23,0,0,0.41,0.35,0.1,4.69,37.24,1.47,0.23,3.75,0.65,2.5,0.09,0.61,1.66,1.4,4.33,0.067058824,3.965333333,1.32E-05,0.004133952

78989,COLEC11,0.05,0,0,0,0,0,0.19,0,0,0,0.05,0,0,0,0,0,0,0,0.11,0.13,0.06,0.11,2.13,0.07,0.24,0.05,0.22,2.16,0.12,0.73,0.42,2.02,0.017058824,0.571333333,1.38E-05,0.004187568

728819,C1GALT1C1L,0,0,0,0.14,0,0,0.21,0,0,0,0.2,0,0,0,0,0,0,0.55,1.83,0.49,0.25,0.08,0,0.99,0.35,0.64,0.44,0.34,0.17,0.82,0.11,1.38,0.032352941,0.562666667,1.39E-05,0.004187568

100506334,LINC00649,1.65,5.81,2.99,4.36,3.62,2.74,0.7,1.12,1.53,1.73,4.16,6.02,5.87,14.2,2.7,3.79,2.86,1.01,0.87,0.57,0.82,2.01,1.11,0.78,0.48,0.48,0.51,0.92,0.45,0.63,0.97,0.54,3.873529412,0.81,1.40E-05,0.004187568

100169890,PEG3-AS1,0,0,0,0,0,0,0.06,0,0,0,0,0,0,0,0,0.06,0,1.16,0.21,0.93,0.05,0.27,0.65,0,0.15,0.57,0,0.91,1.34,0.31,2.3,0.07,0.007058824,0.594666667,1.42E-05,0.004187568

56135,PCDHAC1,0.29,0.04,0.04,0.3,0.14,0.03,0.38,0.06,0.03,0.04,0.46,0.15,0.13,0.14,0.09,0.11,0.12,0.03,0,0,0,0,0.07,0,0,0.03,0.03,0,0.05,0.02,0,0,0.15,0.015333333,1.52E-05,0.004436097

4782,NFIC,12.38,14.49,9.21,7.38,9.04,12.78,12.32,12.83,8.21,10.72,12.45,10.3,9.11,13.62,11.08,6.77,10.66,13.71,25.33,25.4,13.53,10.76,12.71,11.59,26.76,29.95,18.98,20.05,18.21,26.99,15.6,19.13,10.78529412,19.24666667,1.56E-05,0.004465523

84267,C9orf64,0,2.23,0.99,5,4.24,0.03,1.41,5.17,2.66,0.13,0,0.1,0.4,5.57,0,0,0.04,5.25,5.92,7.78,5.47,2.54,3.61,7.28,6.77,6.7,5.67,6.93,8.67,7.84,5.18,5.44,1.645294118,6.07,1.64E-05,0.00458147

1131,CHRM3,3.05,2.43,1.41,3.6,2.08,1.62,0.72,1.06,1.14,1.43,4.34,1.59,1.15,5.74,1.59,1.66,1.36,1.28,0.9,0.28,0.55,1.29,0.86,0.45,0.89,0.47,0.44,0.87,0.67,0.42,0.88,0.51,2.115882353,0.717333333,1.67E-05,0.00458147

9051,PSTPIP1,0,0.03,0.03,0,0,0.04,0,0,0,0,0,0,0,0.11,0,0,0,0.21,2.35,0.39,0.11,0.09,1.44,0.05,0.05,0.04,0.04,0,0.39,0.07,0.2,0.24,0.012352941,0.378,1.69E-05,0.00458147

59084,ENPP5,0,0,0,0,0,0,0,0,0,0,1.29,0,0,0,0,0,0,1.16,0.09,1.13,0.02,0,0.14,0.15,0.33,8.77,0.22,0.04,0.02,0.84,0.11,0.35,0.075882353,0.891333333,1.71E-05,0.00458147

134466,ZNF300P1,0,0,0,0,0,0,0,0.24,0,0,0,0,0.04,0,0,0.03,0,1.03,1,0.66,0.86,0,0,0.75,0.24,0.34,0.58,0.99,1.8,2.16,1.9,1.62,0.018235294,0.928666667,1.72E-05,0.00458147

79957,PAQR6,7.06,2.01,3.3,5.48,9.76,4.13,0.71,4.48,5.48,7.78,9.49,4.78,2.51,3.6,8.99,8.22,4.81,1.62,1.13,0.75,0.5,0.27,0.12,1.69,1.5,1.18,0.84,3.47,0.48,0.71,1.71,0.96,5.446470588,1.128666667,1.81E-05,0.004772904

284047,CCDC144B,0.05,0.07,0.04,3.37,0.14,0.07,0.05,0.07,0.08,0.01,0.13,0.07,0.05,0.32,0.05,0.06,0.03,0.36,4.6,1.3,1.03,0.67,0.46,0.37,1.13,1.38,1.82,2.25,0.79,1.26,1.08,1.19,0.274117647,1.312666667,1.91E-05,0.004966287

84814,PLPP7,0,0.43,0.15,0.07,0,0.07,0.07,0.12,0.12,1.18,0.29,0,0.04,0.14,0.08,0.1,0,1.61,5.35,3.09,0.2,1.01,0.07,3.74,8.97,11.11,7.39,2.65,4.52,7.47,2.74,8.45,0.168235294,4.558,2.08E-05,0.005324983

3316,HSPB2,0.27,0,0,0,0,0.17,0.67,0,0.31,0.23,0,0,0,2.49,0.21,0.36,0,4.11,14.88,39.05,0.17,0.21,0.51,43.04,16.2,199.42,19.5,7.47,22.08,31.02,12.91,32.62,0.277058824,29.546,2.24E-05,0.005559009

4600,MX2,0,0.06,0,0,0,0,0,0,0.34,0.03,0,0,0,0,0,0,0,0.08,0,0.59,0,0.62,2.08,6.23,2.22,0.55,0.14,0.55,1.24,5.32,1.52,0.88,0.025294118,1.468,2.25E-05,0.005559009

56101,PCDHGB5,0.11,0.06,0.06,0.04,0,0.07,0.1,0.08,0,0.09,0.04,0.09,21.37,0.25,0.13,0.18,0,12.76,14.22,9.23,5.39,8.06,1.13,10.99,13.9,3.44,11.39,11.03,7.69,9.5,15.5,10.73,1.333529412,9.664,2.31E-05,0.005559009

8764,TNFRSF14,0.21,1.05,0,8.43,9.9,0.08,1,0,0.05,2.88,0.61,0,0.26,0.17,0.15,0.13,0.05,0.82,9.7,9.85,12.23,18.02,12.2,366.12,22.27,2.09,6.73,12.26,2.35,10.04,13.49,22.26,1.468823529,34.69533333,2.32E-05,0.005559009

1312,COMT,0.68,0.56,0.74,26.24,34.27,5.02,0.68,1.22,0.13,1.45,3.59,0.72,1.16,68.12,1.33,0.4,0.31,48.99,68.95,63.92,76.81,68.8,31.72,62.38,62.43,64.48,60.65,26.04,52.55,55.58,53.92,66.58,8.624705882,57.58666667,2.34E-05,0.005559009

79132,DHX58,0.06,0.05,0.1,0.44,0.09,1.54,1.42,0.16,0.13,0.43,1.48,1.72,0,0.48,0.03,0.14,0.03,0.18,2.7,3.47,1.19,2.13,3.57,7.06,4.52,1.2,2.64,6.53,2.9,3.79,3.5,5.37,0.488235294,3.383333333,2.34E-05,0.005559009

55510,DDX43,0,0,0,0.06,0,0,0,0,0,0,0,0,0,0,0,0,0,0.55,3.76,0,0.39,0.26,0,0,1.34,0.06,0.24,0.24,0.27,0.2,2.27,0.23,0.003529412,0.654,2.38E-05,0.00559698

390213,DOC2GP,2.87,2.46,1.8,0.6,1.22,1.5,0,0,1.33,1.73,0.97,1.1,2.32,5.03,2.02,2.86,0.95,0,0.18,0,0,0,0,0,0.52,0,0,0.17,0,0,0.33,0,1.691764706,0.08,2.43E-05,0.005627519

100750247,HIF1A-AS2,46.88,25.42,16.44,27.37,36.02,55.93,45.58,33.13,53.88,117.53,60.34,42.55,42.44,25.12,29.22,21.81,47.07,26,30.39,27.89,15.15,10.52,16.42,7.97,33.05,14.62,21.52,10.73,12.31,21.48,17.16,16.2,42.74882353,18.76066667,2.50E-05,0.005669276

2157,F8,0.51,0.41,0.77,0.47,0.81,1.61,1.19,0.19,1.23,0.84,2.51,1.04,1.36,0.59,1.14,0.31,0.66,1.28,1.8,3.19,0.72,1.39,2.78,1.35,5.94,2.96,2.99,1.4,1.84,4.58,1.57,4.67,0.92,2.564,2.50E-05,0.005669276

653390,RRN3P2,0.03,0.03,0.06,0.11,0,0.1,0.05,0,0.04,0.09,0.16,0.04,0.08,0.05,0.04,0.02,0.08,0.11,0.03,0.11,1.01,0.44,0.26,0.35,0.39,0.19,0.31,0.25,0.18,0.21,0.31,0.28,0.057647059,0.295333333,2.69E-05,0.006037227

56107,PCDHGA9,0.03,0.22,0.18,0.06,0.13,0.15,0.03,0.2,0.1,0.35,0.16,2.93,0.16,0.04,0,0.18,0.23,1.66,1.25,1.64,1.12,0.21,0.3,2.29,1.79,0.63,0.83,4.36,1.43,1.98,3.09,4.65,0.302941176,1.815333333,2.76E-05,0.006053864

55285,RBM41,4.25,7.33,5.44,9.54,8.84,5.57,4.61,10.35,7.19,6.8,4.08,5.87,6.64,6.51,3.21,4.27,4.73,2.23,2.72,3.08,4.32,3.19,1.97,5.81,3.65,3.72,2.56,3.64,3.07,3.19,3.28,3.09,6.19,3.301333333,2.76E-05,0.006053864

7499,XG,0.05,0,0,0.05,0.03,0.22,0,0,0.03,0,0,0.09,0.25,0.03,0,0,0,0.03,3.4,14.77,2.88,0.27,0.24,1.31,2.27,10.63,0.31,0.04,0.69,14.27,0.04,2.51,0.044117647,3.577333333,2.84E-05,0.006149184

197370,NSMCE1,10.88,15,12.09,8.05,15.99,18.89,17.3,29.63,20.96,21.06,14.4,21.43,18.59,27.47,9.32,13.12,10.56,29.92,24.54,24.5,28.19,22.94,21.43,41.43,40.84,57.24,21.07,46.72,25.18,26.83,49.43,40.25,16.74941176,33.36733333,3.00E-05,0.006433759

10765,KDM5B,29.52,32.99,22.59,26.28,28.83,23.07,22.71,21.27,24.95,25.58,33.8,29.08,18.97,23.58,26.76,22.77,22.21,25.38,13.46,13.07,15.18,13.36,1.9,22.27,18.35,11.04,17.19,21.96,22.37,17.45,24.58,12.87,25.58588235,16.69533333,3.14E-05,0.006588017

8776,MTMR1,5.89,8.9,9.84,6.11,4.42,8.61,5.26,6.68,5.54,5.95,6.02,11.58,10.97,9.19,3.82,4.81,5.15,4.14,3.02,3.31,5.22,4.44,1.64,3.41,4.31,5.37,4.36,5.19,4.64,3.18,5.51,3.47,6.984705882,4.080666667,3.14E-05,0.006588017

55267,PRR34,0,0,0,0,0,0,0.13,0,0,0,0,0,0.05,0.05,0,0,0,0.26,0.04,0.22,0.19,0,0.08,0.29,0.27,0.22,0,0.21,0.47,0.85,0.21,0.62,0.013529412,0.262,3.20E-05,0.006640487

1241,LTB4R,5.94,1.76,6.12,8.55,8.37,2.25,2.26,7.9,9.05,3.31,3.08,2.15,2.26,5.35,8.11,5.47,3.86,0.72,4.45,1.23,1.95,1.11,2.06,1.32,1.61,0.41,1.84,2.64,0.95,1.33,1.36,1.9,5.046470588,1.658666667,3.26E-05,0.006694497

4486,MST1R,0.02,0.04,0.08,0.08,0.05,0.02,0.02,0.07,0.07,0.02,0.03,0,0,0.06,0.06,0.06,0.16,0.1,0.57,0.71,0.14,0.37,0.23,0.48,0.55,0.09,0.07,0.08,0.06,0.11,0.14,0.2,0.049411765,0.26,3.38E-05,0.00685582

89122,TRIM4,0.02,0,0,0,0.02,0.02,3.27,16.75,10.98,0,0.02,0.13,0,0,0,0,0.08,8.93,12.43,13.27,10.98,11.68,7.46,21.06,14.04,4.85,9.36,12.88,11.71,13.32,14.13,14.14,1.840588235,12.016,3.57E-05,0.00716205

387647,PTCHD3P1,0,0,0,0,0,0.03,11.08,3.36,8.33,0.05,0.04,0,0,0,0,0,0,11.09,5.64,8.31,11.67,12.51,5.24,8.51,7.88,8.3,9.45,7.04,9.76,7.67,9.65,6.57,1.346470588,8.619333333,3.71E-05,0.00716205

137209,ZNF572,0,0,0,0.02,0,0,0.12,0.47,0.85,0,0,0,0,0,0,0,0,0.12,0.61,0.51,0.38,0.05,0.26,1.42,0.72,0.22,0.33,1.06,0.64,0.39,0.57,0.58,0.085882353,0.524,3.72E-05,0.00716205

9104,RGN,0,0.04,0.08,0.03,0.1,0.13,1.51,0,0,0.06,0,0,0,0.24,0,0,0,2.23,7.05,5.74,0.99,0.49,3.3,0,4.29,1.11,0.92,0.31,0.86,1.87,1.56,1.43,0.128823529,2.143333333,3.75E-05,0.00716205

574,BAGE,0.3,0.55,0.57,1.27,0.4,0.52,0,0,0.09,0.39,0.08,0.44,0.47,0.8,0.45,0.6,1.13,0.08,0,0,0,0,0,0,0,0,0,0.13,0.2,0,0,0,0.474117647,0.027333333,3.75E-05,0.00716205

2331,FMOD,0.02,2.41,0.1,0,0.08,0,5.08,0,0.03,0.03,0.02,0.34,0.72,0.13,0,0.07,0.03,1.58,247.31,70.39,0.79,0.23,2.51,0.06,14.44,4.17,8.68,13.96,72.57,153.95,14.07,98.33,0.532941176,46.86933333,3.76E-05,0.00716205

1287,COL4A5,16.47,142.09,39.27,61.62,36.93,86.57,44.4,75.7,95.44,57.47,36.07,50.81,26.34,69.73,13,8.27,47.63,39.44,1.8,0.49,11.51,20.85,0.59,1.95,3.6,3.73,0.33,68.31,3.11,0.37,15.86,10.06,53.40058824,12.13333333,3.93E-05,0.00716205

79868,ALG13,7.67,11.08,11.93,20.04,20.65,10.58,7.06,19.51,12.93,12.32,6.19,10.4,8.39,19.6,5.94,7.03,7.46,5.52,8.29,5.93,4.32,3.47,5.42,8.13,6.45,6.16,5.91,6.69,6.51,6.09,7.13,6.41,11.69294118,6.162,3.93E-05,0.00716205

331,XIAP,8.55,11.93,11.6,18.96,18.26,12.09,6.8,8.86,7.6,12.39,9.06,12.52,13.48,14.28,7.06,7.14,8.72,6.94,6.61,6.89,7.08,6.28,4.88,9.01,6.57,7.39,7.2,6.43,6.96,8.49,8.52,6.78,11.13529412,7.068666667,3.93E-05,0.00716205

94056,SYAP1,15.34,16.58,20.54,19.44,19.32,21.48,15.89,23.52,19.56,22.92,14.93,21.61,21.79,25.37,9.29,11.21,16.83,14.6,7.73,13.82,15.83,15.96,12.31,13.45,15.01,8.61,13.79,8.78,8.66,14.06,12.54,8.69,18.56588235,12.256,3.93E-05,0.00716205

389741,GLIDR,0.51,0.28,0.17,0.72,1.09,0.22,1.75,3.51,2.26,0.18,0.2,1.39,1.41,1.92,0.57,0.23,0.63,2.93,2.08,1.51,3.89,1.05,4.07,5.03,3.62,4.44,1.76,3.67,1.53,2.18,2.99,2.16,1.002352941,2.860666667,3.93E-05,0.00716205

23704,KCNE4,0.18,0.47,0.14,0.19,0.24,0.04,13,0.23,0.08,4.63,0.21,0.21,0.18,0.39,0.14,0.13,0.14,16.93,43.12,4.15,0.24,0.3,0.48,22.06,10.3,12.11,5.08,7.51,19.28,5.79,6.93,26.69,1.211764706,12.06466667,4.13E-05,0.007459413

51496,CTDSPL2,8.12,6.99,7.36,10.62,8.99,8.37,5.3,9.78,7.3,6.7,6.53,9.4,6.65,10.26,8.46,8.25,8.79,4.86,3.69,5.59,7.57,2.85,1.37,4.54,4.62,5.02,5.02,5.84,6.96,5.66,7,4.83,8.11,5.028,4.53E-05,0.008096116

56140,PCDHA8,0.2,0.15,0.1,0.61,0.29,0.13,0.03,0,0,0.04,0.27,0.39,0.36,0.24,0.43,0.35,0.13,0,0,0,0,0,0,0.04,0.12,0,0,0,0,0,0,0.04,0.218823529,0.013333333,4.81E-05,0.008109926

9317,PTER,0.51,0.04,0.67,0.47,0.48,0,1.84,0,1.08,0.13,0,0.69,0,2.23,0,0,0,0.78,2.89,1.94,2.08,0.84,2.78,5.22,2.19,4.16,4.61,0.38,4.05,4.54,0.89,3.66,0.478823529,2.734,4.88E-05,0.008109926

142684,RAB40A,0.88,1.17,2.13,0.65,0.47,1.45,0.28,1.79,0.82,1.14,1.14,0.82,0.95,1.25,0.91,1.15,0.69,0.47,0.35,0.54,0.05,0.13,0,0.39,0.67,0.25,0.31,0.78,0.5,0.35,0.67,0.26,1.040588235,0.381333333,4.88E-05,0.008109926

56113,PCDHGA2,0.25,0.76,0.29,0.12,0.07,0.17,0.12,0.28,0.06,0.11,0.9,0.2,0.47,0.3,0.2,0.26,0.07,2,3.55,2.02,1.07,1.45,0.11,0.27,3.15,1.93,1.56,1.24,1.81,1.47,2.63,1.28,0.272352941,1.702666667,4.89E-05,0.008109926

90843,TCEAL8,53.66,119.68,91.39,107.58,108.11,110.69,42.12,132.32,71.75,110.18,60.72,122.69,120.95,93.63,46.81,51.34,50.47,51.94,39.08,34.85,38.41,31.93,8.16,91.22,54.33,44.32,40.55,48.89,38.19,37.44,70.38,48.77,87.88764706,45.23066667,4.89E-05,0.008109926

84295,PHF6,10.16,12.26,12.35,27.59,21.49,14.78,7.37,9.21,10.13,16.77,12.51,11.88,10.22,18.23,6.14,9.6,10.63,6.5,4.03,4.58,13.04,7.5,2.31,10.41,6.48,5.16,4.86,6.72,5.6,5.03,9.32,4.96,13.01882353,6.433333333,4.89E-05,0.008109926

84321,THOC3,19.01,17.83,12.6,18.91,17.25,18.48,7.93,14.09,11.15,18.46,13.49,12.81,13.92,17.94,15.19,12.68,25.33,6.83,6.84,11.26,15.42,12.82,4.91,12.66,11.34,7.17,5.41,12.61,8.64,9.75,7.34,8.85,15.71,9.456666667,4.89E-05,0.008109926

25821,MTO1,3.63,1.9,2.86,4.94,4.7,4.51,5.67,5.63,6.83,2.22,2.41,3.55,4.05,4.91,2.13,2.4,3.58,5.4,6.2,5.54,6.54,5.11,6.21,7.87,5.78,5.17,5.51,6.87,6.47,6.74,4.76,6.62,3.877647059,6.052666667,4.89E-05,0.008109926

378884,NHLRC1,0.96,0.7,0.12,0.33,0.22,0.84,0.51,0.85,0.68,0.8,1.27,0,0.04,1.19,0.21,0.55,0.81,1.4,1.33,1.31,1.67,0.99,0.73,2.24,2.18,2.1,1.17,0.95,0.96,1.26,1.3,0.98,0.592941176,1.371333333,4.91E-05,0.008109926

386593,CHKB-CPT1B,1.26,1.17,0.23,2.27,3.44,2.08,0.86,2.65,2.34,2.04,1.36,0.92,0.85,1.55,1.11,1.23,2.43,0.03,0.22,1.16,0,0.51,1.39,0.55,0.65,0,0,0.32,0,0.67,0,0.25,1.634705882,0.383333333,5.16E-05,0.008459039

441869,ANKRD65,0,0.44,0.06,0.13,0,0.13,0.14,0.3,0.04,0.18,0.64,0,0.06,0.06,0.12,0.13,0,1.39,1.61,1.76,0.26,0,0.41,1,6.65,1.89,1.21,1.12,2.68,3.38,2.52,0.64,0.142941176,1.768,5.52E-05,0.00896462

3201,HOXA4,0,0,0,0,0,0,0.14,0,0,0,0,0,4.72,3.19,0,0,0,1.1,1.49,1.07,4.7,4.92,0.57,0.76,1.67,1.33,0.87,5.21,2.85,1.17,3.31,0.05,0.473529412,2.071333333,5.74E-05,0.009021227

7757,ZNF208,0,0,0,0,0,0,0.02,0,0.29,0,0,0,0,0,0,0,0,0.03,0.06,0.05,0.01,0.02,0.05,0,0.08,0.04,0.04,0.01,0.04,0.24,0.01,0.02,0.018235294,0.046666667,5.78E-05,0.009021227

9024,BRSK2,0.19,0.04,0.25,0.19,0.18,0.13,0.18,0.16,0.19,0.16,0.13,0.09,0.31,1.3,0.1,0.15,0.29,0.11,0,0.08,0.03,0,0.07,0.19,0.07,0.08,0.02,0.08,0.03,0.06,0.03,0.04,0.237647059,0.059333333,6.03E-05,0.009021227

54749,EPDR1,0.06,0,0.03,3.56,1.38,0.03,1.61,0,0,0.27,0.22,6.08,0.38,0.51,0,0,8.09,43.52,5.16,2.27,4.79,0.18,3.62,9.3,47.36,74.03,21.5,2.54,19.2,27.06,15.92,22.68,1.307058824,19.942,6.06E-05,0.009021227

3177,SLC29A2,2.64,4.39,4.67,2.65,2.5,0.98,0.92,5.34,1.67,7.23,1.87,5.13,7.78,2.54,1.69,4.35,1.26,0.8,0.13,0.19,0.41,0,0.55,0.58,0.74,1.48,0.16,2.67,0.39,0.21,2.71,1.72,3.388823529,0.849333333,6.06E-05,0.009021227

79948,PLPPR3,5.33,11.41,8.32,2.76,2.33,1.62,0.74,1,3.22,14.23,26.02,0.26,0.67,12.24,3.16,4.68,2.95,1.24,0.99,0.31,0.15,0.49,0.18,0.04,0.21,0.11,0.07,6.36,0.28,0.23,1.81,0.45,5.937647059,0.861333333,6.06E-05,0.009021227

2956,MSH6,11.95,14.15,14.61,14.24,12.23,18.39,8.62,19.03,14.08,14.83,15.63,17.11,14.68,25.37,14.27,14.53,23.7,7.95,9.34,9.02,13.21,6.89,7.11,13.53,11.35,8.32,7.3,13.63,11.96,9.37,15.97,9.56,15.73058824,10.30066667,6.06E-05,0.009021227

6050,RNH1,88.78,52,63.34,72.4,103.1,112.31,118.56,84.59,76.71,78.51,41.08,72.08,69.06,75.1,79.21,64.69,104.59,105.9,117.68,241.52,87.43,146.99,59.86,158.46,139.7,150.33,212.23,96.33,131.38,175.81,117.24,138.43,79.77117647,138.6193333,6.06E-05,0.009021227

7587,ZNF37A,2.38,1.03,1.42,0.92,1.36,1.97,4.43,2.35,1.84,1.22,2.21,0.04,0.05,2.03,2.29,1.48,2.19,2.7,2.15,3.21,2.91,2.45,1.43,4.44,3.92,3.8,2.78,2.59,3.57,4.08,3.4,4.06,1.718235294,3.166,6.06E-05,0.009021227

113802,HENMT1,0,0,0.56,0.6,0.29,0,1.88,2.3,1.86,0,0,0,0.19,0.75,0,0,0,3.09,1.31,2.1,2.31,0.65,0.25,1.69,2.57,2.99,1.85,3.07,4.08,2.72,4.67,2.19,0.495882353,2.369333333,6.09E-05,0.009021227

387914,SHISA2,32.02,1.98,6.3,2.1,6.17,11.63,3.7,46.61,31.86,5.75,5.4,11.7,0.66,1.51,12.91,21.9,72.44,1.27,0.07,0,0.14,0.57,0.05,0,0.47,19.82,0.11,0.13,2.98,0,3.47,0,16.15529412,1.938666667,6.16E-05,0.009021227

400966,RGPD1,2.06,1.67,1.96,2.5,2.59,2.07,0.72,1.53,1.47,1.76,2.12,1.76,1.02,2.62,1.12,1.55,2.59,0.21,0.2,1.14,0.33,0.59,22.37,0.17,0.84,0.33,0.36,0.28,0.22,0.39,0.5,0.3,1.83,1.882,6.23E-05,0.009021227

3429,IFI27,0.6,0.19,0.2,0.13,0.88,2.9,0.89,0.13,0,0.78,0.6,1.52,0.29,0,0.42,0.71,1.18,4.53,4.56,2.31,26.47,779.53,35.09,5.19,2.56,3.03,0.67,0.52,1.71,22.41,1.34,9.65,0.671764706,59.97133333,6.23E-05,0.009021227

57631,LRCH2,2.45,2,3.19,3.31,5.58,3.19,2.61,4.03,1.86,3.31,2.77,3.66,5.11,6.26,1.68,2.47,1.25,1.62,0.91,0.68,1.7,1.22,0.06,0.69,1.67,0.31,0.66,4.18,1.49,1.07,1.74,1.19,3.219411765,1.279333333,6.24E-05,0.009021227

120103,SLC36A4,4.37,4.05,4.01,5.42,3.6,5.95,3.37,5.35,4.01,6.95,3.73,2.88,4.08,3.95,6.89,7.07,7.74,3.7,3.64,2.87,3.02,2.04,3.77,2.4,3.31,2.93,2.79,3.33,2.38,3.36,3.98,2.97,4.907058824,3.099333333,6.25E-05,0.009021227

929,CD14,0,0,0,0.1,0.77,0.05,0.1,0.11,0.11,0,0.15,0.11,0.06,1.35,0,0,0.06,0.1,1.24,2.83,0.23,0.4,196.65,0.51,6.11,0.64,8.35,0.21,0.17,2.34,0.19,2.24,0.174705882,14.814,6.86E-05,0.00982751

116444,GRIN3B,0.55,0.85,0.86,2.04,1.54,0.22,0.19,1.2,0.33,0.43,2.78,1.27,2.21,1.27,1.01,2.41,0.25,0.14,0.18,0.34,0.17,0.16,0.04,0.11,0.15,0.27,0.2,0.51,0.14,0.26,0.31,0.32,1.141764706,0.22,7.31E-05,0.010346236

56126,PCDHB10,6.25,4.3,5.08,3.37,6.46,1.52,1.97,3.81,1.98,3.06,2.68,6.47,5.51,3.01,1.78,1.69,1.03,3.58,1.84,1.34,0.5,0.49,0.08,0.06,0.69,1.99,1.38,0.81,0.44,0.56,0.14,0.49,3.527647059,0.959333333,7.33E-05,0.010346236

54884,RETSAT,13.29,10.9,9.26,8.41,6.93,13.44,17.63,14.78,11.97,13.27,23.94,12.53,14.51,8.36,8.23,9.35,8.42,21,15.82,24.44,16.6,35.46,23.68,79.71,22.2,17.57,15.52,15.09,11.68,17.94,15.71,13.63,12.07176471,23.07,7.48E-05,0.010406324

8091,HMGA2,40.58,21.62,39.98,18.6,10.18,32.81,10.43,67.7,12.25,72.84,38.86,68.9,46.95,125.67,27,58.87,22.45,1.64,1.02,1.16,26.56,8.42,0.54,0.06,14.43,4.96,5.3,20.7,3.61,1.69,75.08,2.6,42.09941176,11.18466667,7.48E-05,0.010406324

119587,CPXM2,0.02,0.02,0.05,4.86,0.08,0.08,0.12,0,0,0.1,0.04,0,0.02,0.13,0,0,0,0.81,61.6,6.73,4.57,0.21,0.33,26.73,45.7,0.41,0.33,0,0.66,2.55,0.17,13.62,0.324705882,10.96133333,7.82E-05,0.010799893

317761,C14orf39,0.55,0.69,0.59,0.8,0.36,1.02,0.22,0.8,0.3,1.56,1.1,0.34,0.16,1.22,0.88,1.7,0.23,0,0.17,0,0,0.03,0,0,0.21,0.79,0.18,0.35,0,0,0.41,0.03,0.736470588,0.144666667,7.87E-05,0.010799893

85012,TCEAL3,34.64,45.2,59.89,40.49,31.2,71.38,34,82.33,59.79,24.75,66.89,60.72,46.71,54.97,28.77,23.02,29,34.44,26.31,20.87,20.51,14.19,2.78,19.7,30.53,25.93,27.23,10.87,13.83,17.34,33.78,24.75,46.69117647,21.53733333,7.93E-05,0.010805258

8284,KDM5D,0,0,0,0,0,0,0.01,0,0,0.05,0,0,0,0,0,0,0,0.01,7.65,0,0,0,3.41,16.56,0.05,6.47,0.03,8.5,5.88,0.02,9.62,6.5,0.003529412,4.313333333,8.29E-05,0.011215241

256764,WDR72,6.22,12.46,11.31,0.3,0.41,9.89,0.16,0.27,0.21,0.32,4.21,1.82,1.12,2.41,0.44,6.73,1.53,0.16,0.08,0.09,0.03,0.44,7.44,0.18,0.13,0.13,0.04,0.09,0.1,0.14,0.19,0.1,3.518235294,0.622666667,8.53E-05,0.011294621

54739,XAF1,0.04,5.32,0.07,0.06,0.18,0.22,0.02,0.39,0.02,0.56,0.04,0.5,0.29,3.85,0.1,0.04,0.21,0.16,3.71,6.06,1.37,4.65,27.12,2.78,5.06,3.16,3.64,1.69,6.75,7.62,5.74,9.12,0.700588235,5.908666667,8.53E-05,0.011294621

1122,CHML,2.8,2.88,3.62,4.48,3.57,6.35,2.69,4.62,6.41,2.65,3.41,4.25,2.73,9.99,2.63,1.84,4.74,2.9,0.76,0.52,2.84,1.43,0.41,2.41,1.39,2.6,1.43,1.82,1.57,0.94,3.55,1.31,4.097647059,1.725333333,8.58E-05,0.011294621

493861,EID3,0.15,0.57,0.43,0.74,0.75,1.27,0.43,0.17,0.35,0.91,1.02,0.12,0,1.4,0.18,0.4,0.37,0.48,2.3,2.45,1.62,1.38,0.38,2.07,1.48,0.78,1.3,2.18,1.63,2.79,2.71,1.49,0.544705882,1.669333333,8.58E-05,0.011294621

2925,GRPR,0.11,0.78,0.65,0.29,0.23,0.19,0.06,1.13,0.63,3.17,0.14,1.4,0.37,1.05,0,0.19,0.91,0.32,0,0.09,0.05,0,0,0.03,0.07,0,0,0.05,0.12,0.05,0.02,0.03,0.664705882,0.055333333,8.74E-05,0.011429239

728262,FAM157A,0.33,0.29,0.52,0.62,0.47,0.07,0.16,0.4,0.26,0.15,0.12,0,0.07,0.45,0.38,0.35,0.81,0,0,0,0,0,0,0.21,0.24,0,0.07,0.16,0,0,0.05,0,0.320588235,0.048666667,9.06E-05,0.011694598

56137,PCDHA12,4.15,0.07,0.18,1.26,0.29,0.27,0.67,0,0,0.08,6,0.38,0.35,0.78,0.69,1.15,3.4,0.09,0,0,0,0.1,0.07,0,0.04,0.06,0,0.03,0,0,0,0,1.16,0.026,9.12E-05,0.011694598

51186,TCEAL9,64.25,135.64,154.55,269.1,162.56,185.86,81.84,144.43,89.23,119.99,126.8,175.81,150.04,172.58,59.13,80.48,61.48,81.9,47.2,48.33,85.46,59.01,20.69,64.62,70.84,88.13,53.96,52.43,51.16,42.31,96.01,75.74,131.3982353,62.51933333,9.18E-05,0.011694598

23189,KANK1,3.76,0.85,4.58,3.67,3.14,2.62,9.87,2.24,0.62,1.82,0.33,6.41,6.07,5.07,0.45,0.34,1.64,7.29,19.39,9.33,11.79,18.3,13.51,20.43,7.66,33.65,3.45,1.02,6.99,16.66,10.68,3.19,3.145882353,12.22266667,9.18E-05,0.011694598

3224,HOXC8,0,0,0,0,0,0,0,0,0,0,0,0,0.16,9.08,0,0,0.08,0.03,2.26,9.63,0.03,0.04,0.06,2.54,10.94,4.73,2.39,0.09,7.81,14.35,15.92,0,0.548235294,4.721333333,9.34E-05,0.011823672

220388,CCDC89,0,0,0,0,0,0,0,0,0,0,0,0.04,0.11,0.08,0,0.09,0.08,0.1,0.17,0.98,0.03,0,0,0.46,1.5,0.5,0.61,0.8,0.38,0.94,0.85,0.72,0.023529412,0.536,9.41E-05,0.011834918

349334,FOXD4L4,0.07,0.15,0.09,1.03,1.18,0.11,0.09,0.02,0.01,0.27,0.06,0.17,0.08,0.19,0.28,0.21,0.17,0.08,0,0.05,0,0,0.01,0.02,0.02,0,0.03,0.08,0.07,0.04,0.05,0,0.245882353,0.03,9.56E-05,0.011921904

56120,PCDHGB8P,0,0,0,0,0.04,0,0,0,0,0.05,0.08,0,0.05,0,0,0,0,0.08,0.17,0,0.04,0.04,0.21,0.04,0.3,0.04,0.04,0.1,0.13,0.17,0.13,0.09,0.012941176,0.105333333,9.60E-05,0.011921904

79961,DENND2D,0.07,0,0.21,0.1,0.07,0,0.34,0.38,0.05,0.15,0.05,0.03,0.06,3,0,0,0,0.46,1.3,0.91,1.09,0.16,6.76,0.51,2.04,0.3,0.31,3.14,0.24,1.51,0.23,2.55,0.265294118,1.434,9.72E-05,0.01199246

10642,IGF2BP1,13.75,7.08,9.3,14.48,15.09,5.59,3.72,7.56,7.37,8.36,4.38,4.51,4.03,28.55,8.3,7.13,8.03,4.28,0.11,0.67,9.44,2.2,0.24,0.03,0.9,1.69,1.8,5.22,3.31,0.11,5.65,1.3,9.248823529,2.463333333,0.000100278,0.012291885

729264,TP53TG3D,0,0,0,0,0,0,0,0,0,0,0,0,0,0,0,0,0,0,0,0.06,0,0.06,0,0.5,0.85,0.28,0.12,0,0.28,0.19,0.05,0.25,0,0.176,0.000102838,0.012426016

4353,MPO,0,0,0,0,0,0,0,0,0,0,0,0,0,0,0,0,0,0,0.12,0,0.02,0.03,0.26,0.03,0.06,0.15,0,0.14,0.1,0.02,0,0,0,0.062,0.000102838,0.012426016

84690,SPATA22,0,0,0,0,0,0,0,0,0,0,0,0,0,0,0,0,0,0,0.37,0.13,0.14,0,0,0,1.21,0.06,0,0.23,0.24,0.11,0.25,0.07,0,0.187333333,0.000103284,0.012426016

3198,HOXA1,0,0,0,0,0,0,0.1,0.42,0,0.48,0,0,0.89,0.25,0,0,0,0.13,0.2,1.12,0.9,0.99,0.06,0.55,2.13,0.59,0.86,0.13,1.29,0.87,0.74,0.04,0.125882353,0.706666667,0.000104588,0.012505638

4056,LTC4S,0.23,0,0.1,0,0.12,0,0.12,0.52,0,0.3,0,0,0.14,0.31,0.14,0.46,0.14,1.97,1.06,1.13,0.77,1.8,0.44,0.14,0.94,0,3.25,0.31,1.25,2.3,0.8,0.96,0.151764706,1.141333333,0.000106175,0.012617948

8237,USP11,34.4,62.69,46.34,43.97,52.94,38.76,15.53,33.53,25.62,72.16,39.65,21.26,19.2,53.51,33.48,28.9,37.62,25.23,24.69,13.36,17.59,8.86,4.69,27.94,25.32,23.03,13.81,38.59,17.23,14.48,28.29,15.35,38.79764706,19.89733333,0.000112256,0.013179984

4734,NEDD4,16.14,13.48,10.67,16.24,17.13,15.69,6.91,15.95,12.2,11.56,6.75,6.52,11.26,23.75,9.7,10.81,13.01,5.03,2.59,5.89,10.7,15.35,3.73,4.89,10.71,8.84,10.05,9.08,6.2,4.03,6.12,3.93,12.81,7.142666667,0.000112256,0.013179984

162968,ZNF497,4.27,3.02,2.63,4.09,4.35,4.27,3.24,3.31,2.97,3.57,5.92,3.88,1.97,3.34,3.01,4.15,5.09,1.47,0.78,2.38,0.46,2.05,0.8,4.32,1.91,1.16,0.57,1.85,0.9,1.49,2.44,3.44,3.710588235,1.734666667,0.000117084,0.013583113

23643,LY96,0.73,4.1,0.21,6.45,7.6,0.12,8.21,0,0.34,0.68,0,1.73,3.23,1.46,0.58,0.49,0.36,0.52,4.86,9.91,15.39,19.73,8.91,10.17,16.49,5.76,9.27,10.24,3.45,15.08,2.26,18.44,2.134705882,10.032,0.000117084,0.013583113

337875,HIST2H2BA,0,0,0,0,0,0,0,0.88,2,0,0,0,0,0.41,0,0,0,1.59,1.19,1.36,0.3,0,1,1.37,1.92,0.71,0.68,1.53,0.43,0.98,0.41,1.11,0.193529412,0.972,0.000119445,0.013741521

10752,CHL1,0,0.02,0,0,0,0,0,0,0,0,0,0,0,0,0,0,0,1.95,0.01,0.01,0,0,0.09,1.11,0.19,0.04,0,2.17,0.02,0.14,0.08,0,0.001176471,0.387333333,0.000121039,0.013741521

56311,ANKRD7,0,0,0,0,0,0,0,0.12,0,0,0,0,0,0,0,0,0,0.65,0,0.12,0.21,0,0,0.78,0.3,0.18,0,0.1,0.25,0.05,0.43,0.13,0.007058824,0.213333333,0.000121269,0.013741521

100128385,FAM225B,0,0,0,0,0,0,0,0,0,0.33,0,0,0,0,0,0,0,0.33,0.96,0.5,0,0.15,0,0,7.9,1.23,3.01,0.38,0,2.1,0.22,0.56,0.019411765,1.156,0.000121269,0.013741521

151254,ALS2CR11,0.04,0.01,0.05,0.01,0.04,0,0.09,0.01,0.15,0.02,0.03,0.01,0.07,0.05,0.03,0,0.02,0.07,0.49,0.32,0.12,0.04,0,0.28,3.17,0.47,0.51,0.73,0.19,0.32,0.48,0.94,0.037058824,0.542,0.000122523,0.013751781

100505738,MIR4458HG,0,0.02,0,0.73,0.76,0,0,0,1.09,0.03,0,0,0,0,0,0,0,1.02,0.8,0.32,0.96,0.24,0.68,0.58,0.93,1.61,0.45,2.87,0.28,0.47,1.4,0.72,0.154705882,0.888666667,0.000122771,0.013751781

162632,USP32P1,0.34,0.05,0.14,13.72,0.11,0.04,0.22,0.16,0.23,0.09,0.21,0.07,0.1,0.46,0.04,0.14,0.19,1.49,14.04,10.8,2.49,2.16,0.12,0.34,0.86,3.56,6.34,4.69,0.89,2.97,0.58,0.41,0.959411765,3.449333333,0.00012609,0.014042829

54768,HYDIN,0,0,0,0,0,0,0,0,0,0,0.02,0.05,0,0.22,0,0.05,0,0.46,0.01,0.13,0,0.01,0,0.83,0.75,0.19,0.24,0.52,0.46,0.49,0.7,0.5,0.02,0.352666667,0.000129131,0.014299816

56108,PCDHGA7,0.06,0.02,0,0.08,0,0,6.01,0.18,3.35,0,0.19,0.22,0.27,0,0,0.06,0,4.04,5.42,3.06,0.7,0.64,0.18,1.83,7.68,2.05,3.38,5.71,1.62,2.78,4.12,1.85,0.614117647,3.004,0.000135913,0.014643405

8741,TNFSF13,0.69,0.33,0.63,2.12,1.87,0.69,0.72,0.49,0.35,0.4,0.58,1.83,0.52,0.4,0.1,0,0.43,1.25,3.96,2.25,3.39,0.92,26.18,1.43,4.09,0.55,2.06,2.28,1.77,2.91,1.62,1.86,0.714705882,3.768,0.000136328,0.014643405

23028,KDM1A,44.79,33.28,33.96,43.35,32.32,30.73,19.11,38.99,33.53,33.06,38.03,29.79,24.03,40.23,33.04,31.05,33.06,27.05,17.23,18.02,36.17,23.81,6,21.5,22.65,26.04,17.69,26.54,24.89,19.93,28.47,16.02,33.66764706,22.134,0.000136521,0.014643405

25825,BACE2,28.47,0.1,3.26,0,0,0.28,2.82,9.89,12.46,0.58,19.15,6.91,0.36,0.03,0.2,1.73,6.72,61.86,21.19,18.86,96.28,61.06,2.05,69.57,13.75,19.99,45.64,5.31,22.03,13.83,14.43,25.03,5.468235294,32.72533333,0.000136521,0.014643405

11190,CEP250,10.77,7.26,4.39,6.83,5.47,11.79,4.44,13.21,6.29,10.27,5.19,9.87,7.85,11.45,8.62,10.33,7.91,4.07,6.51,3.71,4.63,4.84,1.01,5.72,7.92,3.08,4.93,5.82,3.77,2.68,5.51,4.33,8.349411765,4.568666667,0.000136742,0.014643405

2000,ELF4,10.62,18.36,21.18,20.8,15.4,19.33,6.75,15.7,15.52,16.4,12.45,22.7,12.57,18.18,9.93,10.12,15.72,4.67,7.1,9.26,16.55,10.18,2.05,6.37,7.07,6.64,6.48,7.54,13.18,10.46,11.44,6.82,15.39588235,8.387333333,0.000136742,0.014643405

1066,CES1,0,0.18,0,0,0,0,0,0,0,0.57,0,0,0.05,0.1,0.04,0,0,13.23,0.2,3.83,0,0.04,336.41,0,7.33,6.18,0.08,4.26,1.11,0.96,4.25,13.3,0.055294118,26.07866667,0.000140861,0.015002117

10974,ADIRF,2.52,1.4,2,0.49,0.75,0.89,2.24,5.38,2.17,2.71,2.14,0,0,1.19,1.12,4.26,1.41,2.24,215.24,48.12,2.35,93.92,1.56,1.65,42.16,11.06,14.87,23.31,3.17,11.13,11.42,61.41,1.804117647,36.24066667,0.000147137,0.015585372

8789,FBP2,0.5,0.23,0.19,0.3,0.12,0.16,0,0,0,0,0.57,0.13,0.28,0.3,0.07,0.45,0.07,0,0,0,0,0,0,0,0,0,0,0,0.15,0,0,0,0.198235294,0.01,0.000150532,0.015858798

29887,SNX10,0,0.05,0.03,0,0,0,0.03,0,0,0,0,0,0,0.04,0,0,0.08,1.08,0.06,0.03,0.51,0.37,47.51,2.55,0.04,0.07,0,0,0.08,0.14,0.1,0.15,0.013529412,3.512666667,0.000151363,0.015860581

79400,NOX5,0.05,0,0.05,0,0,0,0.06,0.09,0,0.1,0,0,0,0.14,0,0,0.03,0.03,0.29,0.16,0.07,0.1,0,0.19,0.29,0.06,0.09,0.31,0.44,0.34,0.28,0.21,0.030588235,0.190666667,0.000156915,0.016354417

23137,SMC5,9.64,7.83,8.51,9.21,6.83,9.17,7.16,11.56,7.61,8.14,8.75,7.69,6.28,12.89,9.44,10.99,14.81,6.54,11.54,5.31,6.77,4.4,2.58,5.54,6.53,6.81,6.37,8.43,5.89,5.8,8.49,6.18,9.206470588,6.478666667,0.000165905,0.016929218

51142,CHCHD2,71.9,75.47,80.17,186.98,153.91,103.77,135.81,81.3,62.51,107.61,76.6,153.93,179.41,207.53,79.3,56.38,87.73,200.36,162.03,149.82,231.65,186.63,246.84,200.01,174.72,334.04,137.5,180.18,170.76,144.12,185.96,187.4,111.7829412,192.8013333,0.000165905,0.016929218

6231,RPS26,124.98,100.64,125.63,176.45,134.06,144.85,282.74,412.55,333.3,121.6,88.12,119,124.58,154.14,102.01,83.08,165.7,400.53,509.63,286.63,682.34,250.48,169.65,508.94,496.18,247.55,111.92,848.91,268.82,289.54,309.49,471.76,164.3194118,390.158,0.000165905,0.016929218

4642,MYO1D,7.59,0.91,3.68,9.54,8.26,3.51,3.59,1.54,6.18,0.87,17.31,0.18,0.78,0.95,8.61,0.47,7.94,6.92,13.28,117.26,19.4,20.85,2.09,182.23,9.04,15.95,53.77,0.93,20.53,88.16,10.03,25.33,4.818235294,39.05133333,0.000165905,0.016929218

8233,ZRSR2,7.51,8.21,8.55,13.98,12.9,8.36,4.82,11.43,8.35,7.83,6.78,6.87,6.5,8.81,7.2,7.49,8.56,4.93,2.61,5.92,6.08,7.48,3.48,6.5,9.27,3.51,5.54,5.19,2.71,3.98,4.87,4.91,8.479411765,5.132,0.000171457,0.017404627

6579,SLCO1A2,0.2,0.29,0.18,0.22,0.02,0.01,0.06,0.08,0.15,0.03,0.16,0.08,0.08,0.12,0.01,0.02,0.23,0.01,0,0.01,0.02,0.08,0,0,0,0.03,0.03,0.07,0,0,0.01,0,0.114117647,0.017333333,0.000175365,0.017709092

198437,LKAAEAR1,0.17,0.49,0.59,0.54,0.37,0.33,0,0.19,0.1,0.55,0.09,0.1,0.32,0.45,0.3,0.51,0.53,0.09,0,0.19,0.08,0,0,0,0.35,0,0.29,0,0,0.08,0.07,0,0.331176471,0.076666667,0.00018145,0.018229202

85453,TSPYL5,0,0,0.01,5.59,9.6,0,1.24,5.24,3.37,0,0,0,0,2,0,0,0.02,8.35,9.26,5.16,4.69,3.65,0.4,4.3,9.96,7.18,7.44,8.17,9.72,5.14,14.22,4.55,1.592352941,6.812666667,0.000182467,0.018237326

2069,EREG,11.92,1.54,0.28,0.13,0.9,7.3,3.72,23.29,0.9,12.42,0.53,1.57,0.76,4.56,0.88,0.89,3.34,0.23,0,0.08,0.01,0.22,0.78,0.06,1.2,0.19,0.09,3.89,0.03,0.04,0.11,0.21,4.407647059,0.476,0.000184861,0.018382322

80039,FAM106A,0.06,0,0.03,0.33,0.07,0,0.07,0,0.04,0,0.03,0.11,0,0.09,0,0,0,0.05,1.16,0.23,0,0.06,0.25,0.49,0.81,0.25,0.15,1.09,0.68,0.21,1.03,0.57,0.048823529,0.468666667,0.000195754,0.019251479

3200,HOXA3,0,0,0,0,0,0,0.05,0.1,0,0.26,0,0,3.77,6.02,0,0,0,1.29,4.46,0.3,2.31,2,0.04,0.96,2.94,0.45,0.99,4.9,3.09,0.82,7.11,0.08,0.6,2.116,0.000195977,0.019251479

644962,TNRC18P1,0.08,0.06,0.07,0.12,0,0,0.04,0,0.05,0.05,0.24,0.05,0.05,0.1,0.09,0.16,0.05,0,0,0.04,0,0,0,0,0.05,0,0,0.03,0.04,0,0,0,0.071176471,0.010666667,0.000197491,0.019251479

26287,ANKRD2,0.05,0,0.04,0.06,0,0.05,0,0.12,0,0,0.05,0.06,0,0.2,0,0,0.07,0.11,0.37,0.22,0.34,0.58,0.1,0,2.26,14.78,0,1.18,0.37,0.51,0.22,0.42,0.041176471,1.430666667,0.000197552,0.019251479

91948,LINC00923,4.31,5.48,3.15,4.39,4.62,2.86,3.19,3.75,3.35,5.33,6.82,2.59,7.35,5.47,4.63,4.37,2.74,2.43,3.47,2.17,2.55,3.2,16.69,2.56,3.37,1.79,2.14,2.42,1.86,2.23,2.34,1.72,4.376470588,3.396,0.000200561,0.019351172

11187,PKP3,1.91,4.02,2.18,2.65,0.41,5.62,0.71,7.24,3.3,1.33,0.87,0.45,0.83,6.59,0.79,0.33,3.25,0.74,0.31,0.72,0.15,0.55,0.34,0.18,0.62,0.6,0.73,0.16,0.46,1.57,0.35,0.24,2.498823529,0.514666667,0.000200561,0.019351172

85409,NKD2,0,0,0,0,0.12,0,0.04,0,0,0,0,0.82,0,0,0,0,0,0.71,0.03,0.25,0.11,0.05,0.75,0,1.24,0.09,0,2.85,0.14,0.14,0.63,1.67,0.057647059,0.577333333,0.000204285,0.019547248

3221,HOXC4,0,0,0,0,0,0,0,0,0,0,0,0,1.57,9.14,0,0.04,0,0.42,3.5,4.18,0.08,0,0,12.85,7.93,3.17,3.09,0.43,8.85,7.51,15.99,0.26,0.632352941,4.550666667,0.000204599,0.019547248

388182,SPATA41,0,0.02,0,0,0,0,0,0.1,0,0,0,0.03,0,0,0,0,0,0.1,0.08,0,0.11,0.11,2.01,0.03,0.03,0,0.16,0,0.17,0.17,0.08,0.06,0.008823529,0.207333333,0.000208022,0.019777291

1962,EHHADH,0.13,0.58,0.29,0.04,1.72,0.56,0.94,0.36,1.87,0.32,1.34,0,0,2.17,0.45,0.54,0.84,3.46,1.58,1.1,1.62,3.14,8.34,4.32,2.23,1.71,1.23,1.46,1.54,1.46,1.91,1.77,0.714705882,2.458,0.00021439,0.02001938

55609,ZNF280C,0.97,3.11,2.05,1.89,1.58,2.09,0.84,1.51,1.25,3.27,2.04,3.04,2.95,1.65,0.76,1.75,1.38,1.22,0.92,0.54,1.15,0.39,0.27,1.13,1.29,0.79,0.66,1.69,0.65,0.87,1.16,0.66,1.89,0.892666667,0.000214677,0.02001938

5366,PMAIP1,15.32,13.5,13.5,5.37,3.38,13.25,9.14,19.05,12.01,11.13,6.48,14.07,28.26,25.75,7.23,8.71,17.92,5.8,0.93,2.05,5.79,32.45,1.52,0.36,1.93,1.8,2.13,4.52,3.06,0.52,11.15,1.57,13.18058824,5.038666667,0.000214677,0.02001938

1593,CYP27A1,4.93,4.93,2.98,3.62,8.51,4.94,1.24,5.97,2.59,8.22,9.83,0.18,0.23,6.86,2.34,1.34,0.27,4.29,27.16,9.33,10.35,7.85,41.04,232.4,23.8,8.11,8.16,40.59,5.63,5.95,13.41,8.7,4.057647059,29.78466667,0.000214677,0.02001938

554203,JPX,1.52,3.13,2.5,2.61,3.5,1.64,1.56,1.95,1.8,2.45,1.55,1.46,1.74,4.21,1.91,1.93,1.91,1.77,1.52,1.17,1.13,1.75,0.76,1.45,1.31,1.36,2.35,1.37,1.26,1.65,1.38,1.17,2.198235294,1.426666667,0.000230613,0.02137591

273,AMPH,0.09,0,0.02,0.02,0.05,0,0.02,0.02,0.08,0.11,3.63,0.03,0.33,0,0.03,0,0.14,7.16,0.26,3.42,9.43,0.03,0.17,0.03,1.26,0.66,0.1,1.04,6.12,2.06,6.67,1.79,0.268823529,2.68,0.000239627,0.02137591

100133311,HOXA-AS3,0,0,0,0,0,0,0.3,0,0,0,0,0,0.25,5.57,0,0,0,0.02,1.78,1.13,3.24,0.58,0,1.84,3.16,2.88,2.58,1.19,0.23,1.29,7.13,0,0.36,1.803333333,0.000239806,0.02137591

3204,HOXA7,0,0,0,0,0,0,0.74,0,0,0,0,0,0.81,9.71,0,0,0,0,4.95,3.76,7.86,1,0.07,3.97,5.69,6.73,5.37,2.95,0.39,3.13,19.4,0,0.662352941,4.351333333,0.000239806,0.02137591

4330,MN1,102.8,25.79,41.45,11.46,11.18,54.88,9.39,72.61,60.5,32.66,17.23,18.02,3.34,11.05,50.31,51.7,63.24,33.94,7.84,17.63,2.52,3.4,0.31,0.09,2.5,0.91,2.33,2.14,36.82,17.92,5.45,10.23,37.50647059,9.602,0.000241556,0.02137591

56271,BEX4,41.77,50.98,36.19,95.81,101.87,37.21,26.18,79.05,20.86,22.91,55.39,72.91,72.29,85.23,28,40.55,15.93,35.23,13.44,2.73,25.22,6.79,4.25,90.3,13.31,11.22,12.18,21.37,14.89,14.45,37.9,23.6,51.94882353,21.792,0.000241556,0.02137591

8718,TNFRSF25,11.25,6.41,7.99,39.84,32.36,5.35,7.39,14.3,8.71,9.71,20.13,7.03,7.05,7.16,10.51,12.41,12.1,6.13,1.09,3.08,4.48,10.28,1.14,0.32,11.1,10.47,4.29,2.57,4.65,1.04,7.65,2.36,12.92352941,4.71,0.000241556,0.02137591

286527,TMSB15B,5.83,11.16,5.32,11.69,7.81,6.37,1.34,9.4,9.31,4.45,11.46,9.28,2.95,12,5.16,3.08,6.29,3.3,2.22,0.87,3.95,1.24,0,7.31,3.26,0.77,0.65,4.89,4.05,1.87,5.96,1.49,7.229411765,2.788666667,0.000241556,0.02137591

22921,MSRB2,5.63,7.41,7.07,3.52,7.01,8.75,6.38,8.58,5.1,8.29,13.64,8.94,9.39,13.52,6.55,6.33,2.89,8.71,9.66,12.49,8.79,9.91,11.8,21.7,12.07,14.82,8.54,13.61,9.88,10.92,9.08,11.71,7.588235294,11.57933333,0.000241556,0.02137591

735301,SNHG9,4.27,6.03,3.87,2.36,1.06,2.95,1.34,3.08,5.24,2.59,4.06,4.85,1.81,5.26,3.81,0.75,3.44,11.99,3.7,7.05,7.01,4.84,6.6,5.61,4.45,7.28,5.04,9.78,2.51,4.94,9.45,9.7,3.339411765,6.663333333,0.000241556,0.02137591

100129396,FAM106CP,0,0,0,0.63,0.04,0.04,0,0,0.05,0,0.04,0,0,0,0,0,0,0.29,0.96,0.34,0.28,0.32,0,0,0.19,0.46,0.61,0.18,0.33,0.19,0.14,0.15,0.047058824,0.296,0.000242499,0.02137591

23209,MLC1,0,0,0,0,0.02,0,0,0,0,0.13,0,0,0,0,0,0,0,17.74,0,0,0,0.07,0.08,0.02,0.81,0.04,0.09,0.11,0.02,0.02,0.09,0.79,0.008823529,1.325333333,0.000243576,0.02137591

84215,ZNF541,0,0,0,0.07,0.03,0,0,0,0,0,0,0,0,0,0,0,0,0,0.1,0.02,0.01,0.04,0.03,0.15,0.04,0,0.09,0.17,0,0.12,0.01,0.04,0.005882353,0.054666667,0.000245534,0.02137591

22824,HSPA4L,0.57,0.07,0.17,1.59,0.5,0.56,2.24,0.87,0.87,0.17,1.27,0.16,0.47,1.36,0.13,0.42,0.8,4.78,3.25,3.27,5.21,2.36,7.43,0.29,4.32,2.36,1.81,2.36,0.82,1.8,0.49,5.71,0.718823529,3.084,0.000247337,0.02137591

96610,BMS1P20,6.51,5.8,7.51,15.13,13.03,5.21,6.42,7.24,5.53,8.93,7.98,2.9,4.17,5.49,6.33,6.92,9.1,4.3,4.06,4.17,4.63,4.13,3.86,4.17,5.1,5.74,4.16,3.37,5,5.05,5.17,5.63,7.305882353,4.569333333,0.000247987,0.02137591

56109,PCDHGA6,0.18,0.76,0.24,0.16,0.03,0.06,0.36,9.26,0.03,0.12,0.2,0.69,0.81,0.37,0.14,0.68,0,2.21,2.79,2.04,0.76,0.44,0.36,2.06,6.99,0.46,1.81,3.84,1.86,2.32,4.81,1.82,0.828823529,2.304666667,0.000248313,0.02137591

8637,EIF4EBP3,5.52,1.43,0.96,1.82,2.49,7.59,1.25,3.49,0.56,2.8,0.64,4.28,4.28,1.31,2.59,0.8,0,1.9,6.65,9.44,1.9,3.66,11.88,42.97,10.75,5.82,7.04,11.39,4.78,13.1,3.22,11.68,2.459411765,9.745333333,0.000248639,0.02137591

141,ADPRH,0.56,0.03,0.4,1.2,1.59,0.14,2.95,0.54,0.58,0.11,0.21,0.05,0.16,0.9,0.05,0.08,0.18,3.23,1.16,0.91,3.48,4.22,1.29,4.47,3.41,6.81,2.45,0.07,2.1,2.52,1.28,1.08,0.572352941,2.565333333,0.000248965,0.02137591

6444,SGCD,0,0,0,0,0.02,0,3.45,0,0,0.04,0.35,1.59,12.81,0,0,0,0,2.97,12.46,26.83,0,0.02,0.27,37.24,4.83,40.08,9.31,5,13.2,7.36,2.87,0.91,1.074117647,10.89,0.00026541,0.022687929

285148,IAH1,0,0.07,0.07,21.44,15.21,0.08,25.17,36.22,32.99,0.21,0,0,0,41.18,0,0,0,37.81,45.1,39.59,17.55,24.13,16.53,41.78,33.76,39.26,31.69,38.5,31.14,33.51,38.05,35.81,10.15529412,33.614,0.00026811,0.022818629

1272,CNTN1,0,0,0,0,0.01,0,0,0,0,0.02,0.02,0.06,0,0,0,0,0,0.84,0,0.19,0.03,0.08,0,5.46,0.15,0.01,0.05,0.65,0.53,0.31,0.66,0,0.006470588,0.597333333,0.000279812,0.023351858

3225,HOXC9,0,0,0,0,0,0,0,0,0,0,0,0,0,2.04,0,0.04,0,0,3.2,2.95,0.52,0,0,20.95,6.75,4.37,4.14,0.04,1.96,9.78,11.16,0,0.122352941,4.388,0.000282781,0.023351858

57188,ADAMTSL3,0.12,0,0,0.41,0.3,0,0.01,0,0,0,0,0,0.02,0.03,0,0,0,6.61,4.55,0.03,0.01,0.07,1.28,0,0.16,0.37,0.03,3.43,1.24,0.82,0.04,0.41,0.052352941,1.27,0.000284224,0.023351858

339926,EHHADH-AS1,0,0.09,0,0,0.11,0.1,0,0,0.06,0,0.27,0,0,0.07,0,0,0,0.17,0,0.17,0.35,0.13,0.81,0.44,0.28,0.23,0.18,0.09,0.14,0.19,0.05,0.06,0.041176471,0.219333333,0.000285387,0.023351858

390927,ZNF793,3.09,2.32,2.65,3.31,2.64,1.99,1.29,2.67,2.14,1.98,2.99,2.02,2.84,3.78,2.52,2.7,2.79,2.35,2.31,1.41,1.1,1.77,1.06,1.97,1.78,1.3,1.46,2.85,1.27,1.28,1.77,1.21,2.571764706,1.659333333,0.000288341,0.023351858

23036,ZNF292,4.99,6.14,4.78,6.5,5.74,4.96,3.64,5.24,4.93,5.26,5.28,5.98,4.28,7.73,5.59,5.76,3.57,3.01,3.98,2.95,2.95,1.79,1.82,4.37,6.1,2.78,3.43,4.95,4.01,3.17,4.75,3.91,5.315882353,3.598,0.000288341,0.023351858

727851,RGPD8,0.38,0.12,0,0.96,1.19,0.39,2.42,1.86,1.72,0.23,0.63,0.88,0.96,2.23,0.3,0.4,0.59,1.25,3.74,3.02,3.49,0.97,1.23,1.97,2.39,1.2,2.94,1.57,3.81,4.4,2.18,1.06,0.897647059,2.348,0.000288341,0.023351858

26013,L3MBTL1,3.78,1.63,2.9,4.28,3.92,1.68,1.67,3.01,3.46,2.58,5.2,1.26,1.14,6.71,2.87,3.96,1.48,0.82,1.96,0.91,0.86,0.88,0.48,5,1.8,1.03,0.72,1.37,0.33,0.37,2.77,1.08,3.031176471,1.358666667,0.000289951,0.023351858

57826,RAP2C,9.03,20.4,22.13,29.19,27.75,17.26,7.01,18.61,14.66,17.92,10.1,17.99,17.81,35.71,7.08,9.44,9.53,9.16,14.12,8.67,10.32,5.51,7.57,6.59,8.54,8.92,7.98,9.09,8.24,5.54,10.75,6.08,17.15411765,8.472,0.000289951,0.023351858

9266,CYTH2,30.37,18.25,15.56,28.17,32.33,13.99,12.44,39.73,38.29,16.98,17.18,11.98,8.45,18.4,23.18,16.95,20.27,13.25,7.36,8.87,17.8,11.95,5.2,17.23,11.68,8.07,9.47,14.86,9.67,7.31,17.59,8.29,21.32470588,11.24,0.000289951,0.023351858

10579,TACC2,7.17,8.03,6.59,13.56,7.16,6.79,3.37,18.76,9.98,9.32,4.79,12.29,4.39,6.62,5.55,21.86,10.65,2.07,4.06,3.48,6.27,6.63,2.95,1.78,1.15,12.95,1.84,1.7,2.92,1.95,7.55,3.45,9.228235294,4.05,0.000289951,0.023351858

388152,GOLGA2P7,9.15,6.57,10.76,5.45,4,3.22,1.93,6.06,5.42,7.98,11.39,3.65,4.15,9.18,10.22,10.08,6.99,3.17,1.55,1.63,3.37,2.07,3.08,4.9,3.36,1.02,3.55,5.35,4.38,2.74,7.52,2.14,6.835294118,3.322,0.000289951,0.023351858

10723,SLC12A7,0.89,0.06,0.29,0.47,6.66,1.61,2.93,0.38,0.17,0.04,3.9,5.23,0.69,6.25,0.19,2.03,0.32,3.68,6.04,1.27,11.95,12.05,15.57,20.17,4.43,11.69,1.77,1.69,8.83,5,2.19,6.99,1.888823529,7.554666667,0.000289951,0.023351858

63950,DMRTA2,0,0,0,0,0,0,0,0,0,0,0,0,0,0,0,0,0,3.96,0.04,0,0,0,0,0.06,0.03,0.08,0,0,0.02,0.04,0.02,0.08,0,0.288666667,0.000294394,0.023467222

10942,PRSS21,0,0,0,0,0,0,0,0,0,0,0,0,0,0,0,0,0,0.14,0.13,0,0.43,0,0.27,0.24,0.1,0,0,0,0.12,0.12,0,0.48,0,0.135333333,0.000295597,0.023467222

28954,REM1,0,0,0,0,0,0,0,0,0,0,0,0,0,0,0,0,0,0,3.25,0.29,0,0.11,0.08,0,0,0,0.05,0,0.32,0.24,0.04,1.63,0,0.400666667,0.0002962,0.023467222

149954,BPIFB4,0,0,0,0,0,0,0,0,0,0,0,0,0,0,0,0,0,0.22,0,0.07,0,0,0,0,56.11,0.11,0.04,0,0.27,0.12,0.03,1.69,0,3.910666667,0.0002962,0.023467222

8808,IL1RL2,0,0,0.1,0,0.08,0,0.12,0.25,0,0,0.08,0,0,0.05,0,0,0,0.08,0.24,1.02,0.21,0,0.65,0,1.46,0.46,0.25,1.3,0.3,1.34,0.06,1.52,0.04,0.592666667,0.000297433,0.023469509

164684,WBP2NL,0,0,0,0,0,0.03,0,0,0,0,0,0,0,0,0,0,0,0.03,0.17,0.03,0,0.04,0,0.3,0,0.07,0,0.08,0.08,0.09,0.3,0,0.001764706,0.079333333,0.000302389,0.02376436

9752,PCDHA9,0.37,0.25,0.55,1.3,0.32,0.26,0.45,0.28,0.49,0.48,0.29,0.17,0.48,0.31,0.85,0.45,1.02,0.1,0.23,0.25,0.31,0.11,0.08,0.27,0.34,0.16,0.29,0.29,0.14,0.28,0.18,0.16,0.489411765,0.212666667,0.000306608,0.02399919

9420,CYP7B1,0,0,0.03,0,0,0,0,0,0,0,0,0,0,0,0,0.03,0.04,0.82,0.51,1,0,0,0.64,0,0.04,0.82,0.24,0.08,0.88,1.12,1.94,0,0.005882353,0.539333333,0.000314367,0.024508039

8287,USP9Y,0,0,0,0,0,0,0.01,0,0,0.02,0,0,0,0,0,0,0,0.01,3.36,0,0,0,0.88,4.38,0.01,2.28,0,4.35,2.75,0.01,5.75,2.7,0.001764706,1.765333333,0.000328315,0.024903379

9720,CCDC144A,0.01,0,0,1.06,0,0,0.03,0,0,0,0.02,0,0.02,0,0,0,0,0.1,1.09,0.52,0.22,0.14,0,0,0.05,0.32,0.75,0.34,0.13,0.24,0.2,0.02,0.067058824,0.274666667,0.000329704,0.024903379

445328,ARHGEF35,1.54,0.05,0.01,0.12,0.27,0.25,1.62,0.05,1.42,0.04,2.6,0.04,0.05,1.98,0.07,0.2,0.16,2.18,1.86,1.73,0.2,0.37,2.6,5.46,3.58,0.7,2.2,0.56,3.85,2.5,2.55,2.02,0.615882353,2.157333333,0.000330977,0.024903379

389114,ZNF662,0.2,0.2,0.16,0.91,0.6,0.17,0.79,1.09,0.27,0.2,0.07,0.21,0.26,3.93,0.27,0.15,0.17,0.97,2.97,0.88,0.94,0.95,0.4,0.58,1.23,0.38,1.1,2.64,1.45,1.81,2.76,1.4,0.567647059,1.364,0.000331395,0.024903379

148741,ANKRD35,0.13,1.78,0.28,0.07,0.02,0.52,0.07,0.49,0.03,1.96,0.2,0,0.62,1.9,0.41,0,0.03,0.48,1.9,2.63,0.41,3.23,0.66,0.08,5.84,2.11,2.08,4.95,0.64,1.84,2.86,2.63,0.500588235,2.156,0.000331395,0.024903379

2053,EPHX2,0.25,0.34,0.22,0.07,0.37,0.53,0.31,0.11,0.24,0.36,0.16,3.07,0.04,4.05,0.04,0.86,0.04,1.44,8.62,0.97,1.4,0.33,11.35,6.44,1.21,0.57,0.51,1.21,1.39,3.77,0.89,0.7,0.650588235,2.72,0.000331814,0.024903379

3223,HOXC6,0,0,0,0,0,0.04,0,0,0,0,0,0,3.35,24.17,0,0.04,0,0,5.85,17.65,0.08,0,0.14,16.48,20.31,10.45,9.49,0.59,8.87,29.58,22.82,0.63,1.623529412,9.529333333,0.000333072,0.024903379

56122,PCDHB14,2.51,4.95,6.07,1.86,1.95,2.78,2.91,6.11,5.06,4.69,6.27,5.48,5.12,3.38,4.32,4.32,1.32,5.9,3.12,1.99,0.72,1.33,0.91,0.32,0.77,2.58,2.39,0.99,0.87,1.36,0.32,0.81,4.064705882,1.625333333,0.000333073,0.024903379

7621,ZNF70,2.27,2.77,2.14,2.34,2.77,1.39,1.11,2.7,2.24,2.46,2.63,1.61,2.23,2.2,2.72,2.97,3.86,1.36,1.88,0.93,1.14,0.6,0.69,2.02,1.74,0.96,1.24,1.4,1.55,1.82,1.83,2.55,2.377058824,1.447333333,0.000333493,0.024903379

64901,RANBP17,1.94,1.39,3.2,2.12,1.11,1.36,1.65,1.37,2.18,1.64,2.41,0.78,1.47,4.05,2.17,0.79,2.46,2.23,0.91,0.14,1.07,0.42,0.27,0.6,0.02,0.63,1.34,1.05,1.34,0.35,1.48,0.57,1.887647059,0.828,0.000333493,0.024903379

1522,CTSZ,51.54,48.56,129.22,49.12,53.87,50.82,47.55,11.96,51.28,66.45,173.91,0.06,0.06,141.23,30.38,29.18,29.64,176.8,138.36,167.82,185.59,315.79,99.07,272.87,182.89,75.14,248.13,41.07,96.63,85.89,82.19,120.88,56.75470588,152.608,0.000333493,0.024903379

3992,FADS1,28.56,24.94,26.12,27.22,22.92,19.18,19.74,25.65,24.2,23.13,52.32,17.11,52.72,19.94,33.66,67.07,22.53,16.22,10.8,14.16,20.01,12.18,15.17,9.99,19.37,49.05,8.92,11.63,15.49,14.8,29.68,21.3,29.82411765,17.918,0.000346827,0.02541226

22859,ADGRL1,6.9,5.34,4.49,6.31,6.82,6.16,5.03,8.53,9.86,2.93,10.3,3.95,3.28,6.37,2.92,3.5,5.64,5.1,1.02,3.62,1.39,0.71,0.25,4.85,1.79,0.66,1.18,4.94,5.58,3.56,2.3,2.95,5.784117647,2.66,0.000346827,0.02541226

5426,POLE,7.19,5.74,7.09,7.84,9.81,9.01,1.85,6.33,5.24,5.95,6.09,3.35,2.94,10.56,7.1,8.04,12.72,1.36,3.16,4.1,6.12,2.51,1.99,3.04,3.63,1.55,2.56,6.34,3,3.39,4.24,2.47,6.873529412,3.297333333,0.000346827,0.02541226

6611,SMS,43.04,48.66,31.06,57.41,35.56,68.92,45.92,116.9,79.82,70.13,33.58,56.24,72.69,71.56,56.18,38.65,66.53,29.69,16.35,15.23,63.45,58.67,9.38,36.58,32.68,35.62,27.14,38.91,30.78,17.29,48.17,29.6,58.40294118,32.636,0.000346827,0.02541226

9249,DHRS3,19.61,6.7,7.05,1.32,2.16,17.76,8.39,6.42,2.25,1,4.8,11.7,18.94,6.03,1.25,0.12,0.6,22.55,64,20.39,14.04,18.21,74.01,1.39,53.08,8.54,8.84,26.32,34.04,67.55,4.49,18.32,6.829411765,29.05133333,0.000346827,0.02541226

22829,NLGN4Y,0,0,0,0,0,0,0.09,0,0,0.02,0,0,0,0,0,0,0,0,4.61,0,0,0,1.32,0.1,0.02,0.84,0.02,3.57,2.23,0.01,7.09,1.93,0.006470588,1.449333333,0.000359538,0.026174855

3222,HOXC5,0,0,0,0,0,0,0,0,0,0,0,0,0.08,1.97,0,0,0,0,1.56,2.27,0.17,0,0,3.4,2.19,1.07,0.88,0.2,3.14,4.17,5.52,0,0.120588235,1.638,0.000361814,0.026174855

3952,LEP,0,0,0,0,0,0,0,0,0,0.03,0,0,0,0,0,0,0,0.02,0.04,0.94,0,0,0,0.02,0.06,5.46,0.41,0,0.11,1.16,0,6.09,0.001764706,0.954,0.000361978,0.026174855

9086,EIF1AY,0,0,0,0,0,0,0,0,0,0.14,0,0,0,0,0,0,0,0,8.43,0,0,0,16.33,10.91,0.07,21.47,0.06,6.34,6.16,0,8.63,8.06,0.008235294,5.764,0.000362607,0.026174855

3205,HOXA9,0,0,0,0,0,0,5.76,0,0,0,0,0,0,29.19,0,0,0,0,11.85,8.58,20.7,1.31,0.2,3.85,10.15,3.97,5.73,0.09,0.1,3.56,14.73,0,2.055882353,5.654666667,0.000367269,0.026413589

23316,CUX2,0.01,0,0,0,0,0,0,0,0,0,0,0,0,0,0,0.01,0,0,0.02,0,0,0.01,3.67,0.01,0.04,0.08,0.01,0.03,0.01,0.01,0.01,0,0.001176471,0.26,0.000372525,0.026573705

7849,PAX8,0,0.03,0,0.86,0.72,0.92,0.14,0.09,0.08,0.74,0,0.28,0.31,0.97,0,0,0.14,1.49,0.95,1.51,0.19,0.36,0.42,1.07,2.98,4.98,0.72,0.18,1.19,1.35,3.4,0.74,0.310588235,1.435333333,0.000374823,0.026573705

84898,PLXDC2,1.57,0.02,0,0,0.03,1.45,0.45,0,0.2,0,0.17,0.03,0,4.62,0.17,0.34,0.14,24.43,26.5,1.68,0.56,0.1,1.89,0.07,2.92,5.01,26.52,36.49,23.69,7.11,7.28,0.07,0.540588235,10.95466667,0.000374823,0.026573705

727764,MAFIP,0,0,0,19.87,20.84,0,0.03,0,0,0,0,0,0,6.75,0,0,0,1.25,7.18,14.32,1.81,7.55,4.38,19.32,11.01,9.29,11.76,4.03,1.47,10.15,0.49,10.52,2.793529412,7.635333333,0.00037495,0.026573705

375748,ERCC6L2,1.54,1.85,1.8,2.76,1.97,2,1.07,1.85,1.97,1.59,1.57,1.98,1.75,1.86,1.87,2.62,2.27,0.91,1.45,0.89,1.4,0.91,0.65,1.78,1.39,1.53,1.79,2.39,0.95,0.95,1.74,1.05,1.901176471,1.318666667,0.00038377,0.026908555

767811,H2BFXP,0.65,1.89,1.44,3.13,3.56,0.72,0.49,2.19,1.94,1.8,2.56,2.37,2.63,2.59,1.43,1.11,1.16,0.79,0.17,0.4,0.09,0.22,0,1.65,0.38,0.1,0.1,1.73,1.34,0.17,2.17,0.55,1.862352941,0.657333333,0.000384721,0.026908555

4325,MMP16,7.41,2.49,2.97,5.21,5.29,0.64,3.84,14.13,9.27,1.75,23.35,1.93,1.72,2.12,1.8,4.19,20.43,1.65,0.69,0.75,7.35,1.06,0,0.69,3.78,0.08,1.24,0.81,1.78,0.34,1.79,0.09,6.384705882,1.473333333,0.000385197,0.026908555

65991,FUNDC2,7.04,8.28,7.26,10.39,10.83,12.26,8.58,8.83,6.54,10.25,8.38,13.27,10.22,8.57,5.97,7.78,8.33,12.19,14.47,12.19,11.78,9.85,33.1,18.56,11.5,23.67,10.33,8.5,11.86,12.62,10.8,57.18,8.987058824,17.24,0.000385197,0.026908555

165631,PARP15,0,0,0,0,0,0,0.08,0.37,0.09,0,0,0,0.09,0,0,0.04,0,0.04,0.07,0.06,0.25,1.34,0.14,0.4,0.41,0.59,0.13,0.07,0.15,0.27,0.06,0,0.039411765,0.265333333,0.000401705,0.027961555

3990,LIPC,0,0.11,0,0,0,0,0,0,0,0,0.09,0,0.06,0.06,0,0,0,0,1.65,8.33,0,0,34.67,0.05,0.25,0.82,0.05,0.33,0.29,3.99,0.2,2.24,0.018823529,3.524666667,0.000411835,0.028082659

126326,GIPC3,3.58,13.08,1.51,15.83,11.03,1.11,0.11,2.49,2.85,15.24,2.59,1.24,0.92,2.01,2.16,3.24,0.83,0.11,0.14,0.04,0.5,0.22,0.32,6.57,0.02,0.04,0.02,0.54,0.26,0.05,6.64,0.38,4.695294118,1.056666667,0.00041276,0.028082659

586,BCAT1,49.56,26.4,64.31,20.4,10.83,34.31,39.87,33.85,31.96,26.3,21.56,11.82,22.04,61.93,61.77,46.66,19.61,22.96,10.76,4.53,20.17,16.06,0.69,0.13,11.11,17.41,21.88,12.55,37.08,19.34,16.34,13.26,34.30470588,14.95133333,0.000413531,0.028082659

5339,PLEC,82.9,40.41,68.18,94.61,66.97,94.96,74.98,127.91,157.64,85.3,129.3,98.34,58.59,111.31,105.43,88.65,176.2,65.82,76.63,61.62,26.37,57.42,7.26,45,48.32,72.62,128.32,46.6,52.3,39.79,76.15,40.51,97.74588235,56.31533333,0.000413531,0.028082659

1314,COPA,61,61.09,63.62,50.19,46.4,57.43,50.45,58.85,47.89,70.67,55.27,57.03,69.07,66.23,73.89,47.46,53.39,49.35,49.73,39.95,53.75,45.94,26.48,44.14,50.14,45.74,44.08,53.04,47.77,44.55,71.4,44.32,58.23117647,47.35866667,0.000413531,0.028082659

23543,RBFOX2,34.8,47.65,31.61,33.02,24.63,50.09,31.11,45.47,37.67,31.15,33.13,36.31,32.85,40.23,38.95,44.49,31.64,27.41,23.07,27.81,27.68,22.79,4.61,18.89,31.4,16.68,27.39,45.87,31.03,29.39,43.35,26.68,36.75294118,26.93666667,0.000413531,0.028082659

6277,S100A6,1.83,2.05,375.45,1204.31,242.13,1022.28,11.36,984.64,468.74,5.79,375.65,0.66,0.7,433.95,0.81,413.05,2.86,407.26,1515.65,2056.74,772.25,1739.12,63.3,304.47,952.74,1306.26,1400.1,766.9,844.46,1350.55,960.54,1497.39,326.2505882,1062.515333,0.000413531,0.028082659

284757,MIR646HG,0,0,0,0,0,0,0,0,0,0.23,0,0,0,0,0,0,0,0,0.17,0.08,0.03,0,0.14,0,0.29,0.04,0,0.19,0.1,0.1,0.19,0.09,0.013529412,0.094666667,0.000434406,0.02936354

2949,GSTM5,0,0.04,0,0.15,0.1,0.05,0.05,0,0,0.06,0.19,0,0,0.06,0,0,0.12,0.05,16.05,24.27,0,0.11,0.27,0.06,31.96,0.42,0.05,23.46,1.9,9.76,7.5,19.6,0.048235294,9.030666667,0.000435406,0.02936354

84856,LINC00839,0.53,0.19,0.19,0.24,0.31,0.28,0.97,0.46,0.34,0.31,0.13,0.15,0.2,0.75,0.16,0.38,0.68,0.29,1.7,0.69,2.44,2.27,0.43,6.03,7.51,1.68,1.83,0.19,1.93,1.22,0.44,2.51,0.368823529,2.077333333,0.000442168,0.02955547

56474,CTPS2,4.72,9.42,6.69,16.77,14,9.42,2.13,8.35,9.92,10.92,8.52,7.88,6.03,16.24,3.67,7.31,5.08,2.69,1.78,2.86,6.05,3.2,1.83,8.53,3.87,1.62,2.32,6.6,2.81,2.48,6.08,2.43,8.651176471,3.676666667,0.000444318,0.02955547

1773,DNASE1,1.73,2.22,1.53,3.29,2.53,2.41,1.77,2.06,1.83,2.15,2.02,1.75,1.81,2.65,1.57,2.31,1.98,1.22,0.82,0.92,1.06,0.62,0.41,1.79,1.46,0.86,1.2,2.36,1.23,1.1,2.83,1.22,2.094705882,1.273333333,0.000444318,0.02955547

2690,GHR,0.5,0.18,0.82,0.02,0.11,0.31,1.41,0.21,0.66,0.33,2.27,0.13,0.94,0.31,0.86,0.17,0.41,1.24,1.56,0.77,0.9,0.68,5.19,0.52,1.06,3.23,0.51,2.37,1.27,2.07,2.72,2.26,0.567058824,1.756666667,0.000444318,0.02955547

150,ADRA2A,0,0,0,0,0,0.02,0.04,0,0.02,0.12,0.04,0,0.05,0.17,0,0,0.05,1.83,0.89,42.84,0.27,0,0.79,0,0.25,0.41,0.06,0.03,1.88,8.76,0.05,1.45,0.03,3.967333333,0.000448322,0.029665698

346528,OR2A1,0.08,0,0,0,0.04,0.08,0.09,0,0,0,0,0,0.1,0,0,0,0,0.09,0.18,0.18,0,0,0.16,0.97,0,0.05,0.28,0.15,0.3,0.22,0.42,0.1,0.022941176,0.206666667,0.000449019,0.029665698

9023,CH25H,0,0,0,0.57,0.06,0.26,0,0,0,0,0.5,0,0,0,0,0,0,0.29,0.05,13.36,1.86,0,0.41,0,4.6,0.3,1.29,0.05,7.52,4.75,0.47,0.39,0.081764706,2.356,0.000450839,0.02968528

339965,CCDC158,0,0,0,0,0,0,0.02,0,0,0,0.02,0,0,0,0.02,0,0,0.32,0.7,0.07,0,0,0.12,0,0.48,0,0.14,0.02,0.16,0.02,0.15,0.43,0.003529412,0.174,0.000455445,0.029887648

10566,AKAP3,0.07,0,0.02,0.03,0.08,0.07,0.05,0.08,0.06,0,0.17,0.06,0.06,0,0,0.12,0.03,0.08,0.4,0.03,0.48,0.23,0.05,0.74,0.29,0.19,0.03,0.36,0.19,0.26,0.21,0.32,0.052941176,0.257333333,0.000460015,0.030086221

55000,TUG1,32.52,35.78,35.31,49.41,44.72,27.65,25.92,36.68,26.21,31.63,42.95,35.06,24.09,43.83,28.72,34.13,23.58,25.61,32.07,23.32,25.88,17.41,4.18,20.1,26.72,22.28,23.62,40.35,19.28,23.25,34.01,25.25,34.01117647,24.222,0.00049146,0.031717061

133619,PRRC1,23.63,26.06,26.12,20.34,19.05,24.03,26.84,24.94,19.76,32.43,16.27,23.11,22.44,30.53,30.03,18.32,28.01,18.39,26.7,13.77,18.13,15.18,15.21,18,19.28,15.17,16.55,21.15,14.86,15.51,27.18,18.2,24.23,18.21866667,0.00049146,0.031717061

59338,PLEKHA1,22.12,11.32,12.33,20.66,19.37,12.44,15.55,28.73,18.77,16.84,13.95,15.97,11.02,6.6,9.26,9.34,10.59,7.86,6.8,9.89,10.2,13.4,8.77,14.62,5.18,8.35,7.95,6.37,7.94,7.15,12.74,7.6,14.99176471,8.988,0.00049146,0.031717061

51643,TMBIM4,10.5,9.27,7.99,27.26,11.42,17.17,17.89,21.4,13.92,8.3,12.3,24.73,0.14,19.91,7.32,7.75,8.63,12.44,21.91,26.79,23.63,21.13,17.3,51.04,30.42,22.18,16.66,23.08,15.96,26.78,22.77,38.11,13.28823529,24.68,0.00049146,0.031717061

64127,NOD2,0,0,0,0,0,0,0,0,0,0,0.03,0,0,0,0,0,0,0.02,0.07,0,0.23,0.1,0.81,0.02,0,0.45,0,0,0.07,0,0.01,0.02,0.001764706,0.12,0.000503334,0.031830616

23639,LRRC6,0.23,0.1,0,1.84,1.24,0.27,0,0.44,0,0,0.71,0.14,0.34,0.21,0.14,0.23,0.19,0.63,0.65,1.48,2.16,0.84,0.52,1.77,0.93,0.48,0.43,0.69,0.77,1.21,1.07,0.41,0.357647059,0.936,0.000505171,0.031830616

56110,PCDHGA5,0.09,0.02,0.03,0.19,0.06,0.12,0.26,0.03,0.14,0.12,0.19,0.22,0.9,0.2,0.14,0.15,0.04,2.69,3.36,0.67,0.11,0.14,0.24,0.07,1.44,0.5,1.28,2.42,1.49,1.44,1.47,1.86,0.170588235,1.278666667,0.000507583,0.031830616

146664,MGAT5B,5.21,0.77,1.41,1.72,0.48,3.14,0.11,0.65,0.27,2.01,2.35,3.77,1.33,5.53,0.73,1.51,0.71,0.81,0.08,0.06,0.16,0.55,0.03,13.92,0.27,0.14,0.1,0.08,0.25,0.11,0.53,0.04,1.864705882,1.142,0.000510609,0.031830616

84996,URB1-AS1,0.09,2.27,0.96,0.88,0.1,0.18,0.99,2.4,3.13,0,0.28,0.11,0.46,1.97,0.55,0.09,0,1.59,2.14,1.01,4.54,1.12,1.77,10.33,2.76,3.31,2.1,3.3,0.5,2.12,4.18,2.76,0.850588235,2.902,0.000510609,0.031830616

401010,NOC2LP2,0,0,0,0,0,0,0,0,0,0,0,0,0,1.3,0,0,0,0.03,0.1,0,0.08,0.32,0.1,0,0,0.06,0.12,0.02,0.05,0.05,0.11,0,0.076470588,0.069333333,0.00051121,0.031830616

375341,C3orf62,2.29,1.6,1.27,2.89,4.15,2.96,1.48,1.87,1.64,1.36,1.86,2.02,1.59,0.89,1.14,1.07,1.49,0.67,0.97,1.05,1.14,0.61,0.58,3.22,1.31,1.1,0.78,1.27,1,1.04,1.15,0.7,1.857058824,1.106,0.000511216,0.031830616

219855,SLC37A2,1.98,1.86,0.84,2.28,2.01,1.21,0.81,2.85,1.38,6.83,1.62,1,2.07,2.87,3.69,7.19,2.82,0.64,0.84,0.53,0.57,1.66,1.12,2.28,1.04,0.56,0.67,1.54,0.51,0.85,1.37,0.65,2.547647059,0.988666667,0.000511216,0.031830616

79446,WDR25,6.03,5.83,9.15,4.17,5.69,5.96,4.72,7.29,7.46,9.49,5.22,4.8,4.35,6.6,6.92,6.89,8.37,4.9,2.17,4.18,6.36,3.81,1.35,5.96,2.99,4.17,4.65,4.27,5.16,4.23,5.34,3.32,6.408235294,4.190666667,0.000511216,0.031830616

57534,MIB1,8.76,10.99,10.5,11.75,11.39,9.79,7.95,9.69,11.98,9.53,8.8,7.99,9.7,8.75,7.6,10.14,13.19,7.78,4.23,5.45,9.11,5.34,1.72,12.81,5.36,9.1,7.28,8.63,6.81,5.38,8.32,5.34,9.911764706,6.844,0.000511823,0.031830616

23186,RCOR1,6.94,8.17,7.06,17.89,12.98,8.55,6.43,7.41,6.61,6.66,7.71,6.79,7.86,8.58,6.72,9.57,7.24,5.44,4.81,5.32,10.86,6.18,2.76,8.53,6.02,6.49,5.3,7.01,6.48,4.51,6.24,5.3,8.421764706,6.083333333,0.000511823,0.031830616

79104,MEG8,0,0,0,0,0,0,3.65,0,0,0,0,0,0,0,0,0,0,0.52,1.65,0.71,0,0,0,0,0.89,0.36,0.55,0.14,0.59,0.29,1.42,0.19,0.214705882,0.487333333,0.000512818,0.031830616

85376,RIMBP3,0.09,0.18,0.43,0.12,0.48,0.35,0.19,0.31,0.5,0.34,0.45,0.1,0.11,0.17,0.31,0.39,1.63,0,0,0.07,0,0,0,0.06,0.25,0.2,0,0.04,0.29,0.15,0.22,0.12,0.361764706,0.093333333,0.00052617,0.032555742

10580,SORBS1,0.07,0,0.02,0.89,3.78,0.55,0.08,0.06,0.15,0.05,0.15,0.05,0.01,0.73,0.05,0.06,0.28,26.19,1.05,0.38,0.22,0.97,14.63,42.98,0.18,24.58,0.07,1.35,0.99,0.21,1.06,0.58,0.410588235,7.696,0.000545192,0.033537097

1149,CIDEA,0.14,0.11,0.04,1.95,0.59,0,0.15,0.15,0.24,0,0.14,0.08,0.17,0,0,0.54,0,0,0,0,0.07,0,0,0,0,0,0,0,0.04,0,0,0,0.252941176,0.007333333,0.000545473,0.033537097

644353,ZCCHC18,1.47,1.15,1.75,4.52,3.1,1.08,0.31,2.47,3.65,0.68,2.92,3.78,1.52,2.02,1.2,0.64,0.67,0.9,0.02,0.17,1.52,0.23,0.1,1.56,0.44,0.05,0.18,0.88,0.63,0.36,1.29,0.2,1.937058824,0.568666667,0.000549053,0.0336511

5616,PRKY,0,0,0,0,0,0,0.01,0,0,0,0,0,0,0,0,0.01,0.01,0,3.87,0.01,0.01,0,0.4,4.49,0,1.25,0,2.51,2.08,0.01,2.97,4.18,0.001764706,1.452,0.000562933,0.03408092

375295,LINC01116,0.14,0,0,0,0,0,11.2,0,0,0.09,0,0,2.83,11.88,0,0,0,1.45,5.46,21.99,10.12,0.26,0.41,9.56,5.31,10.19,4.37,0.32,10.27,16.31,4.75,0,1.537647059,6.718,0.000582042,0.03408092

139231,FAM199X,4.43,6,6.97,15.62,14.72,6.69,7.01,10.01,12.76,13.06,7.15,7.35,6.92,17.16,3.45,4.05,5.83,8.45,3.39,2.62,5.47,6.27,2.28,8.99,3.61,5,3.27,4.77,2.99,3,5.53,2.49,8.775294118,4.542,0.000582296,0.03408092

92344,GORAB,6.03,4.5,5.61,4.64,5.26,6.06,5.71,9.57,5.48,4.24,6.62,6.54,5.51,4.85,5.28,5.31,9.2,3.63,5.37,4.33,2.75,2.2,4.77,2.98,7.65,2.7,4.58,3.31,3.54,4.12,5.7,4.62,5.906470588,4.15,0.000582296,0.03408092

57730,ANKRD36B,1.78,2.1,2.29,5.38,3.74,1.49,1.23,2.07,1.72,2.71,2.08,2.16,2,4.42,3.2,3.66,2.04,0.79,1.64,1,1.32,0.99,0.33,2.59,1.26,0.54,1.14,2.75,0.88,1.16,3.58,0.97,2.592352941,1.396,0.000582296,0.03408092

9406,ZRANB2,30.06,31.78,37.39,41.34,33.69,24.66,25.85,30.04,22.77,35.65,29.46,22.22,25.23,52.22,37.4,29.86,31.75,25.2,31.06,16.21,22.18,20.42,13.72,26.71,29.07,23.42,19.38,29.29,16.57,14.39,32.09,21.87,31.84529412,22.772,0.000582296,0.03408092

55818,KDM3A,13.61,15.09,15.84,22.74,28.83,13.67,10.96,12.9,14.27,12.28,13.51,16.63,15.95,14.31,17.34,16.55,6.75,12.39,9.11,9.2,8.26,7.59,4.91,23.41,12.32,9.75,9.72,12.68,13.28,9.18,12.4,10.7,15.36647059,10.99333333,0.000582296,0.03408092

35,ACADS,1.25,4.23,1.93,3.33,7.04,3.52,3.32,2.14,3.8,1.97,3.67,3.29,4.86,6.34,2.5,1.14,2.28,4.56,9.2,4.39,5.41,4.83,27.08,29.47,5.33,6.86,3.19,12.11,3.09,5.15,4.45,6.63,3.33,8.783333333,0.000582296,0.03408092

647979,NORAD,110.27,86.28,87.31,106.26,114.83,108.19,72.33,113.25,94.49,100.41,169.23,99.4,88.6,108.04,76.89,69.62,77.67,97.73,64.04,80.94,92.88,71.99,26.7,63.74,74.89,80.27,72.38,96.99,57.86,65.55,81.64,67.23,99.00411765,72.98866667,0.000582296,0.03408092

27245,AHDC1,9.67,11.72,9.21,11.91,13.8,12.96,5.58,10.65,16.31,9.44,13.63,12.31,8.71,16.78,12.44,14.75,15.65,8.92,6.01,6.96,7.31,6.32,0.98,7,10.13,14.33,7.64,11.11,8.87,6.26,8.54,9.55,12.08941176,7.995333333,0.000582296,0.03408092

10643,IGF2BP3,22.54,15.3,19.26,27.04,20.86,39.84,9.46,41.39,20.75,19.58,13.76,11.8,9.41,24.4,13.06,17.5,30.34,11.6,0.21,2,28.33,34.38,0.37,5.51,4.2,3.94,2.13,10.7,8.11,0.13,18.8,2.66,20.95823529,8.871333333,0.000582296,0.03408092

4735,2-Sep,203.77,147.48,152.35,258.85,221.02,196.93,163.35,167.75,157.61,152.91,264.04,144.94,150.26,183.57,143.05,152.79,201.83,170.55,101.99,131.91,182.54,147.5,20.01,124.39,126.61,123.42,106.55,145.54,143.93,132.16,214.99,134.53,180.1470588,133.7746667,0.000582296,0.03408092

114799,ESCO1,4.36,3.27,4.27,4.89,5.69,4.31,3.83,4.15,4.19,3.35,4.63,3.63,2.96,4.14,3.78,3.86,3.6,2.92,2.52,3.15,3.92,2,2.16,8.22,2.81,3.7,2.57,2.78,3.5,3.36,3.57,2.53,4.053529412,3.314,0.000582296,0.03408092

3856,KRT8,97.56,40.43,142.05,55.87,32.36,127.46,23.47,164.56,410.8,103.14,4.05,24.07,25.12,878.47,61.74,49.76,90.15,89.96,8.52,1.21,6.6,8.07,145.42,1.16,2.67,1.51,9.12,28.75,26.31,8.66,24.99,4.92,137.1211765,24.52466667,0.000582296,0.03408092

9077,DIRAS3,0.04,0.22,0.16,0.43,0.67,0.44,0.38,0.36,0.47,0.06,0.6,0.32,0,5.47,0.21,0.18,0.34,3.57,0.08,1.76,0.69,3.62,0.17,0.53,9.24,1.61,1.23,16.87,2.73,1.07,1.01,1.18,0.608823529,3.024,0.000582296,0.03408092

5961,PRPH2,0.05,0.55,0,0.05,0.03,0,0.69,0.03,0,0.25,0.02,0,0,0.06,0,0.02,0,0.15,1.19,3.16,0.32,0.09,0,0,3.75,1.58,1.36,5.17,1.56,1.96,4.28,0.4,0.102941176,1.664666667,0.000586858,0.034245073

84218,TBC1D3F,24.12,11.42,17.26,19.33,20.69,10.85,7.02,38.88,11.95,15.72,10.85,14.25,10.16,33.41,20.14,17.94,11.76,6.7,9.63,8.46,5.6,6.83,2.81,15.45,15.43,4.56,8.04,15.91,6.42,6.1,12.05,7.58,17.39705882,8.771333333,0.000588793,0.034255478

123036,TC2N,0,0,0,0.02,0,0.03,0.03,0,0,0,0.06,0,0,0.02,0,0,0,0.11,0.07,0.11,0,0,1.52,0,0.04,0.05,0.02,0.15,0.12,0.16,0.03,0.09,0.009411765,0.164666667,0.000600358,0.034824318

3202,HOXA5,0,0,0,0,0,0,0.19,0,0,0,0.05,0,13.62,12.79,0,0,0,0.24,6.12,1.31,15.18,9.74,0,2.86,7.04,0.65,1.62,23.42,2.14,1.67,17.38,0,1.567647059,5.958,0.00060738,0.035127092

59283,CACNG8,1.73,3.3,1.81,4.13,2.01,0.32,0.03,0.74,0.12,1.61,0.34,0.15,1.39,1.74,1.2,0.6,0.16,0.25,0.01,0.02,1.05,0.96,0.03,0.05,0.09,0.07,0.03,0.2,0.07,0.06,0.07,0.34,1.257647059,0.22,0.000625405,0.035555058

2018,EMX2,0,0,0,0,0,0,0,0,0,0.03,0,0.35,0.96,0,0,0,0,4.16,0.44,35.9,0,0,0.05,0,0.61,0.28,0.88,0.09,4.8,13.97,0.05,0.03,0.078823529,4.084,0.000626289,0.035555058

10630,PDPN,0.03,0,0,0,0,0.03,0.22,0,1.59,0.25,0,0.15,2.34,2.58,0.07,0.03,0,14.4,27.2,3.7,0.1,0.59,0.05,0.03,10.24,21.95,1.55,0.98,13.67,6.29,3.77,0.07,0.428823529,6.972666667,0.000626881,0.035555058

55784,MCTP2,0,0,0,0,0.39,0.04,0.08,0.11,0,0,0,0,0.09,3.72,0.01,0,0.01,0.16,6.18,0.37,0.07,0.01,0.38,0.13,1.44,0.24,0.12,0.09,0.26,0.54,1.67,0.06,0.261764706,0.781333333,0.000628148,0.035555058

286827,TRIM59,8.74,4.29,3.59,3.87,3.58,3.53,3.73,10.54,15.24,4.35,8.68,4.21,4.34,5.79,7.43,9.15,6.78,2.81,2.77,3.53,5.6,1.3,0.31,2.47,3.83,3.21,3.21,3.31,6.74,3.66,6.17,2.56,6.343529412,3.432,0.000630472,0.035555058

112970,KTI12,3.4,2.99,2.35,3.37,2.27,3.74,3.66,6.27,5.18,2.38,3.38,6.65,5.31,3.26,3.01,2.88,3.77,6.55,4.55,6.85,8.29,4.09,3.31,10.9,6.46,5.41,5.06,4.99,5.3,6.55,7.53,5.31,3.757058824,6.076666667,0.000630472,0.035555058

652968,GATSL3,6.81,15.47,5.92,15.11,14.24,10.2,5.18,8.93,7.63,14.77,15.54,7.83,6.42,13.5,7.67,4.08,8.92,5.92,2.61,4.36,6.75,4.86,2.59,5.01,3.76,2.2,4.29,13.56,7.27,3.62,8.18,5.16,9.895294118,5.342666667,0.000631198,0.035555058

200424,TET3,3.42,3.74,4.06,6.18,6.8,2.04,2.3,3.57,5.42,1.74,3.64,2.18,2.32,3.02,3.11,3.15,3.48,3.74,0.46,1.32,3.99,1.11,1.04,1.46,1.39,1.57,2.44,1.69,2.47,1.08,1.97,1.38,3.539411765,1.807333333,0.000631198,0.035555058

100101267,POM121C,15.04,13.26,12.61,20.24,21.81,13.01,12.42,15.16,13.31,12.02,15.73,14.17,11.82,24.19,15.95,11.96,18.14,10.26,12.02,11.39,15.28,11.52,4.41,13.09,12.19,9.68,10.71,13.66,12.66,11.94,10.5,10.15,15.34352941,11.29733333,0.000631198,0.035555058

2099,ESR1,0,0.01,0,0,0,0,0.01,0,0,0.01,0,0,0.06,0.02,0,0,0,0.01,0.22,0.11,0,0.01,0.15,0.34,0.85,0.22,0.29,0,0.02,0.32,0,0.6,0.006470588,0.209333333,0.000635368,0.035686811

124221,PRSS30P,0,0,0,0,0,0,0,0,0,1.21,1.18,0,0,0.03,0,0,0,0.32,0.63,1.26,0.05,0,0,1.28,0.5,0.03,0.22,0.57,0.2,0.63,0.55,0.32,0.142352941,0.437333333,0.000645761,0.036166301

153478,PLEKHG4B,3.12,1.79,0.69,0.06,0.04,0.03,0.03,2.15,0.87,1.9,1.59,1.5,0.1,1.28,2.29,2.51,0.82,2.01,0.01,0.01,0.02,0.01,0,0.06,0.05,0.01,0.02,0.3,0.11,0,0.37,0.04,1.221764706,0.201333333,0.000665702,0.037176283

873,CBR1,0.06,0,13.35,11.87,13.82,0.12,25.65,41.75,23.05,0.08,0.06,60.94,46.53,23.55,0,0,0,37.76,40.53,57.52,23.88,38.27,48.75,101.9,47.68,15.35,32.64,29.37,35.72,48.55,39.45,44.32,15.34294118,42.77933333,0.000668755,0.037240079

339761,CYP27C1,3.6,1.6,2.31,3.71,0.56,2.67,2.38,9.62,5.88,2.14,1.17,1.82,0.41,0.14,0.88,4.44,8.48,0.81,7.6,1,0.03,0.2,0,0.27,1.43,0.03,0.89,0.03,0.26,0.35,0.42,0.02,3.047647059,0.889333333,0.000674121,0.037272741

441317,FAM90A7P,0,0,0,0,0,0,0.04,0,0.05,0,0,0,0.1,0.11,0,0,0,0.13,0.37,0.13,0,0,0,0,0.17,0.6,0.23,0.31,0.11,0.28,0.21,0.1,0.017647059,0.176,0.000674218,0.037272741

56111,PCDHGA4,0.55,0.37,0.24,0.72,0.13,0.21,1.8,0.72,0.07,0.2,1.38,1.27,3.2,0.37,0.5,1.94,0.19,3.91,2.13,5.11,1.35,6.77,0.12,0.86,9.85,2.58,4.13,1.13,1.41,2.49,3.63,3.02,0.815294118,3.232666667,0.000675659,0.037272741

100132403,FAM157B,0.66,0.19,0.44,0.25,0.47,0.67,0.15,0,0.42,0.23,0.12,0,0,0.96,1.01,0.88,0.43,0,0.16,0,0,0,0,0.07,0,0,0.07,0.21,0,0.21,0,0.07,0.404705882,0.052666667,0.000683667,0.037272741

80727,TTYH3,171.76,128.36,69.71,163.8,147.55,68.98,51.82,91,91.78,93.3,148.19,98.7,52.78,116.9,59.15,61.87,62.29,48.77,8.88,44.17,113.08,115.63,15.14,72.57,68.35,13.6,25.57,47.62,50.9,30.6,76.99,24.42,98.70235294,50.41933333,0.000687796,0.037272741

80351,TNKS2,15.99,11.38,12.52,22.98,21.11,15.28,13.95,19.92,20.11,12.88,18.28,14.42,12.45,14.05,13.27,17.32,19.18,14.99,10.11,11.42,11.76,11.5,4.63,16.88,13.31,6.22,11.94,12.26,12.29,12.1,16.16,11.88,16.18176471,11.83,0.000687796,0.037272741

8939,FUBP3,24.32,14.48,18.12,26.74,26.9,20.64,16.87,24.04,22.51,19.32,21.59,18.96,16.61,24.35,21.56,26.39,27.56,17.77,12.51,15.84,19.71,17.11,8.09,22.91,17.19,14.38,16.8,17.64,16.44,15.06,20.83,15.12,21.82117647,16.49333333,0.000687796,0.037272741

10587,TXNRD2,2.98,0.73,0.74,14.04,11.17,6.45,0.44,1.31,1.3,0.1,0.96,0.17,0.09,15.93,0.45,0.26,2.57,5.69,5.92,8.96,43.26,27.07,15.59,9.54,5.9,12.71,7.34,6.79,6.22,7.27,10.86,5.18,3.511176471,11.88666667,0.000687796,0.037272741

10247,RIDA,5.53,3.54,5.22,3.48,3.92,8.99,7.59,6.33,5.05,5.45,8.52,10.47,9.82,2.49,3.57,3.66,5.8,7.93,15.81,13.62,8.32,7.74,228.94,9.14,9.63,12.12,6.11,8.73,7.73,7.46,6.41,9.73,5.848823529,23.96133333,0.000687796,0.037272741

4626,MYH8,0,0,0,0,0,0,0,0,0,0.11,0,0,0,7.37,0,0,0,0.28,0,0.01,0,0,0.02,0.18,0.08,197.19,0.22,0.57,1.73,0.08,0.06,0.1,0.44,13.368,0.000688465,0.037272741

283383,ADGRD1,0.57,0,0,0,0,0,0.61,0.08,0,0.02,0.04,0,0,0,0.02,0,0,0.04,0.04,31.03,0,0,1.47,1.82,0.04,1.28,0.06,0.21,5.27,39.93,1.39,0.29,0.078823529,5.524666667,0.000712114,0.038446291

129642,MBOAT2,24.77,8.9,11.13,8.7,9.14,7.57,11.15,20.79,14.72,16.58,13.66,9.02,9.63,8.88,12,9.24,15.26,8.11,5.96,9.15,1.11,3.88,0.11,7.16,5.05,6.05,6.29,7.89,13.79,13.01,9.24,3.37,12.42,6.678,0.000724659,0.039015491

8638,OASL,0,0,0,0,0,0,0,0,0.05,0.11,0.09,0,0,0,0,0,0,0.14,0.64,0,0.21,0.17,2.61,0.56,0.94,0.1,0,0,0,0.08,0.18,0.04,0.014705882,0.378,0.00074372,0.039931423

3356,HTR2A,0.01,0.03,0.03,0,0,0,4.39,0,0,0,0,0,0,0,0,0,0,0.67,0.18,1.24,0,0,0.06,0,0.07,0.07,3.74,1.09,0.2,0.58,0.19,0.33,0.262352941,0.561333333,0.000749227,0.040092108

1463,NCAN,0,0,0,0,0,0,0,0,0,0,0,0,0.01,0,0,0.01,0,5.08,0.01,0,0,0,0.04,0,0.11,0.11,0,0.04,0.02,0.03,0.05,0.01,0.001176471,0.366666667,0.000750827,0.040092108

123103,KLHL33,0,0.03,0,0,0,0,0,0,0,0.05,0,0,0,0,0,0,0,0,0.15,0.04,0.04,0,0,0,0.16,0,0.22,1.02,0.11,0.51,0.54,0.19,0.004705882,0.198666667,0.000763174,0.040600955

10279,PRSS16,0.18,0.33,0.46,0,0,0.1,0.03,0.14,0.7,0.26,0.03,0.06,0.22,0.24,0.27,0.13,0.41,0.11,0.02,0,0,0.06,0.24,0,0,0.03,0,0,0.02,0.02,0.02,0,0.209411765,0.034666667,0.000764523,0.040600955

130162,CLHC1,1.53,1.01,0.9,1.46,1.09,1.44,0.99,1.87,1.11,1.14,1.72,0.9,0.9,1.3,1.05,1.45,1.63,1.46,0.67,0.58,0.35,0.7,0.11,1.01,1.19,0.7,0.57,0.91,0.7,0.46,1.24,0.65,1.264117647,0.753333333,0.000768303,0.040690823

2647,BLOC1S1,42.4,46.5,28.57,41.89,46.84,77.46,26.7,52.82,32.77,39.56,43.46,45.36,37.65,43.76,38.67,40.54,35.97,41.01,43.3,69.19,52.88,50.58,49.85,44.57,63.36,83.62,49.22,47.96,51.75,53.24,53.69,49.85,42.40705882,53.60466667,0.000776067,0.040879877

4774,NFIA,0.46,0.83,0.14,0.58,1.22,1.56,1.09,1.44,0.73,0.81,0.93,1.23,1.38,0.15,0.52,0.41,0.39,0.78,1.07,1.76,2.73,1.49,1.15,1.26,1.32,1.49,0.61,1.8,2.27,3.63,1.79,2.39,0.815882353,1.702666667,0.000776067,0.040879877

729177,NBAT1,0,0,0,0,0,0,0,0,0,0,0,0,0,0,0,0,0,0.1,0.03,0,0,0.04,0,1.01,0,0.14,0,0.03,0.03,0,0.03,0,0,0.094,0.000788508,0.041330444

8856,NR1I2,0,0,0,0,0,0,0,0,0,0,0,0,0,0,0,0,0,0.03,0.05,0,0.08,0.08,1,0.12,0.14,0.07,0,0,0,0,0,0,0,0.104666667,0.000802282,0.041330444

83869,TTTY14,0,0,0,0,0,0,0,0,0,0,0,0,0,0,0,0,0,0,2.02,0,0,0,1.49,0.74,0,2.28,0,0.56,1.57,0,0.27,1.48,0,0.694,0.000803822,0.041330444

93426,SYCE1,0,0,0,0,0,0,0,0,0,0,0,0,0,0,0,0,0,0,0.11,0,0,0.07,0.87,0,0,0.25,0,0,0.05,0.36,0.37,3.21,0,0.352666667,0.000803822,0.041330444

100379345,MIR181A2HG,1.92,0.73,1.1,0.81,0.14,0.5,1.09,0.58,0.89,1.49,1.18,1.04,0.63,1.02,1.82,2.68,0.48,0.96,0,0.14,0,0.31,0,0.76,1.22,0.29,0.14,0.23,0.81,0,0.33,0,1.064705882,0.346,0.000809032,0.041330444

2535,FZD2,123.23,53.61,51.13,0.06,0.24,52.78,18.28,73.91,62.76,37.47,47.06,54.16,37.96,15.76,38.56,47.9,47.21,14.97,0.31,8.9,0.95,0.29,0.62,2.57,19.44,5.04,7.78,21.21,30.41,15.35,47.11,12.03,44.82823529,12.46533333,0.000810068,0.041330444

9583,ENTPD4,20.9,19.69,18.99,30.24,17.9,14.99,14.58,20.01,19.36,21.71,16.29,14.68,16.42,32,14.85,16.79,22.7,17.95,18.88,10.03,13.7,18.35,6.85,18.91,14.61,11.06,12.2,14.64,8.41,7.69,18.94,8.19,19.53529412,13.36066667,0.000810068,0.041330444

8045,RASSF7,13.22,5.37,12.91,10.13,12.28,7.19,14.69,12.93,15.8,8.68,5.28,9.97,3.76,33.19,17.81,6.94,10.18,12.09,2.4,3.15,5.39,6,25.16,2.99,1.18,1.64,7.27,2.66,3.46,1.88,9.38,2.06,11.78411765,5.780666667,0.000810068,0.041330444

3091,HIF1A,148.2,96.21,51.88,83.7,98.28,159.61,130.9,114.23,184,398.42,196.09,131.53,120.03,63.95,131.21,68.05,163.85,71.23,146.89,62.9,71.13,41.02,22.39,25.79,154.61,78.04,67.95,48.78,54.22,79.99,79.45,42.4,137.6552941,69.786,0.000810068,0.041330444

64403,CDH24,7.16,5.36,4.81,8.05,4.02,4.36,2.46,3.97,8.81,8.49,7.24,3.77,2.89,15.66,6.64,6.49,9.52,3.27,1.22,1.02,10.62,4.67,0.16,2.04,1.67,0.4,0.88,3.74,1.61,0.9,12.93,1.21,6.452941176,3.089333333,0.000810068,0.041330444

94081,SFXN1,16.66,17.12,16.41,13.05,11.65,18.99,14.22,16.59,11.16,25.02,17.61,10.16,9.85,16.94,13.51,11.61,22.48,14.8,15.89,9.34,15.55,8.45,10.19,6.82,10.74,8.07,9.61,13.75,8.7,7.49,13.83,9.62,15.47235294,10.85666667,0.000810068,0.041330444

514,ATP5E,144.49,149.37,189.3,397.59,359.32,220.59,276.27,290.51,263.68,168.89,141.64,347.76,320.15,381.77,124.07,114.04,144.02,509.09,226.37,348.29,524.55,524.28,286.68,484.22,318.03,432.13,365.98,292.25,373.23,264.61,419.34,330.66,237.2623529,379.9806667,0.000810068,0.041330444

26166,RGS22,0,0,0,0,0,0,0.02,0.04,0.02,0,0.21,0.02,0,0,0,0,0,0.02,0.3,0.08,0.03,0.02,0,0,0.09,0.02,0.16,0.18,0.08,0.13,0.09,0.17,0.018235294,0.091333333,0.000821788,0.041818917

6450,SH3BGR,0.89,1.09,2.11,0.16,0.64,2.69,1.35,1.17,0.87,1.93,4.76,1.82,1.2,0.89,1.3,1.04,1.39,8.33,2.4,1.45,0.64,1.97,3.69,3.09,1.91,25.99,3.45,4.04,5.76,4.14,1.87,9.04,1.488235294,5.184666667,0.000829928,0.042013733

27010,TPK1,0.51,0.1,0.03,0.98,1.45,0.22,0.29,0.24,0.63,0,1.93,0.45,0.12,0.47,0.11,0.06,0.41,0.51,0.69,0.58,1.46,3.98,1.92,1.13,1.13,1,0.94,1.27,0.75,0.49,0.86,0.68,0.470588235,1.159333333,0.000829928,0.042013733

2747,GLUD2,0.03,0,0,0.4,0.04,0,0,0.08,0,0,0.7,0,0.01,0,0,0,0,0.65,0.06,0.04,0.75,0,0.83,0.36,0.09,0.12,0.07,0.03,0.09,0.19,0.53,0.04,0.074117647,0.256666667,0.000843377,0.042583968

54361,WNT4,0.13,0,0,0,0.08,0,0.04,0,0.6,0,0.02,0.02,0,0.1,0.07,0,0.16,0.3,0.02,1.33,0.09,0.02,0.32,1.12,0.15,0.39,0.02,23.93,0.65,3.23,1.91,0.04,0.071764706,2.234666667,0.0008484,0.042726904

148811,PM20D1,0,0,0,0,0,0,0,0,0,0,0,0,0,0,0,0.03,0,0,0,0.11,0.03,0.04,0.13,0.04,0,0.04,0.08,0.06,0,0.09,0,0,0.001764706,0.041333333,0.000857266,0.043062174

284348,LYPD5,0.03,0,0,0.28,0.44,0,0.22,0,0.1,0,0.03,0.14,0,0.51,0.04,0.03,0,0.43,0.7,0.41,0.06,0.39,0,0.73,0.2,0.29,0.33,1.7,0.34,0.7,0.33,0.39,0.107058824,0.466666667,0.000861792,0.043120978

79190,IRX6,0,0,0,0,0,0,0,0,0,0,0.03,0,0,0,0,0,0,0.03,0.06,0.34,0,0,0,0.15,0.42,0,0.07,0.08,0.37,0.6,0,0,0.001764706,0.141333333,0.000862862,0.043120978

1745,DLX1,27.38,22.1,20.56,0,0.07,9.2,1.04,23.64,9.64,0.87,4.74,10.74,5.18,0,4.47,5.58,20.09,0.14,0.03,0.36,0.06,0,0,0.39,3.32,0.26,1.17,0,0.03,0.06,0.09,0.6,9.723529412,0.434,0.000868953,0.043314329

619279,ZNF704,0.12,0,0.06,1.76,1.18,0.08,0.16,0.03,0.05,0.05,1.21,0.02,0.01,0.03,0.01,0.07,0.04,1.35,0.67,1.09,1.19,0.17,0.18,16.61,0.04,2.46,0.35,0.08,2.3,4.76,1.19,0.61,0.287058824,2.203333333,0.000884343,0.043325758

5610,EIF2AK2,7.01,5.76,6.26,9.76,7.41,9.31,7.32,8.21,7.92,8.25,7.81,8.33,8.09,7.67,4.79,8.09,8.57,5.36,4.79,5.66,8.44,5.71,1.79,7.31,7.32,5.22,4.71,5.08,6.31,6.81,7.2,5.32,7.68,5.802,0.000887249,0.043325758

79755,ZNF750,0,0.02,0,0.07,0.12,0,0,0,0,0,0,0,0,0,0,0,0,0.02,0,0.02,0.04,0.03,0,0.03,0.03,0.13,0.03,0.02,0.08,0.06,0.02,0.03,0.012352941,0.036,0.000888636,0.043325758

54328,GPR173,7.26,3.19,5.36,8.83,7.62,2.31,3.12,4.7,4.95,4.79,8.71,2.66,3.65,6.11,8.93,5.83,4.98,3.21,2.06,1.62,2.02,1.89,0.03,5.8,2.96,1.75,2.06,4.93,2.67,1.32,7.63,2.13,5.470588235,2.805333333,0.000889189,0.043325758

57622,LRFN1,1.89,0.46,1.28,1.91,2.78,0.22,0.1,0.79,0.6,1.29,3.06,2.83,2.25,0.91,0.73,0.65,0.51,0.75,0.02,0.07,0.47,0.08,0.17,0.72,0.49,0.18,0.31,0.2,0.47,0.06,1.4,0.14,1.309411765,0.368666667,0.000889189,0.043325758

1121,CHM,7.09,10.33,8.31,9.12,9.29,9.19,7.09,6.85,5.04,10.82,9.05,10.16,12.75,7.14,5.5,6.48,6.91,5.48,5.7,4.99,7.91,6.22,2.35,10.28,5.92,5.39,5.94,6.39,4.83,5.57,6.18,5.06,8.301176471,5.880666667,0.000889189,0.043325758

80143,SIKE1,8.16,8.04,6.13,7.88,7.55,9.43,6.04,9.86,7.55,7.02,11.13,7.51,7.23,8.5,7.27,7.37,8.9,6.36,6.07,6.45,5.39,4.01,3.57,5.32,5.53,5.59,5.76,9.07,7.3,7.09,8.36,5.89,7.974705882,6.117333333,0.000889189,0.043325758

254263,CNIH2,3.13,6.59,3.93,0.47,0.83,1.71,1.7,1.23,1.47,2.5,7.94,3.47,3.07,3.44,4.13,1.54,7.61,5.76,0.05,0.18,0.11,0.2,0,0.07,0.82,1.16,0.5,2.25,0.84,0.2,3.73,0.39,3.221176471,1.084,0.000889189,0.043325758

7691,ZNF132,0.92,0.74,1.11,0.2,0.23,1.3,1.17,1.24,1.94,0.62,1.79,0.2,0.18,0.13,0.6,1.02,1.13,2.17,0.68,1.64,2,1.03,0.55,2.43,2.57,2.18,1.76,1.72,1.31,1.77,1.99,2.31,0.854117647,1.740666667,0.000889189,0.043325758

56139,PCDHA10,4.63,0.17,5.25,9.22,1.84,1.11,0.86,0,0.31,0.08,24.29,0.03,0.07,3.5,5.78,4.49,8.85,0.36,0.1,0.03,0.07,0.83,0,0,0.06,0.33,0.1,0,0.03,0.08,0,0,4.145882353,0.132666667,0.000909792,0.043936257

8618,CADPS,0.01,0.01,0,0,0,0.01,0,0,0,0,0,0,0,0,0,0.01,0.02,3.12,0,0.5,0,0,0,0.59,0.9,0.67,0.02,0.83,4.73,0.04,0.02,0.18,0.003529412,0.773333333,0.000918025,0.043936257

3242,HPD,0,0,0,0,0,0,0,0,0,0.07,0,0,0,2,0,0,0,1.55,31.25,0.39,0,0.06,720.56,0,0.28,0.12,0,0.09,0,0.14,0.1,0.06,0.121764706,50.30666667,0.000925946,0.043936257

246126,TXLNGY,0,0,0,0,0,0,0,0,0,0.02,0,0,0,0,0,0,0,0,4.29,0,0,0,1.55,9.57,0,3.49,0,4.34,2.33,0.01,5.89,2.3,0.001176471,2.251333333,0.000938397,0.043936257

57576,KIF17,0.28,0.2,0.1,0.54,0.35,0.09,0.18,0.13,0.18,0.69,0.39,0.2,0.45,0.53,0.27,0.87,0.36,0.1,0.05,0.19,0.24,0.36,0.04,0.02,0.05,0.13,0,0.2,0.02,0.07,0.25,0.07,0.341764706,0.119333333,0.000941071,0.043936257

56145,PCDHA3,0.47,0.22,0.2,1.59,1.43,0.32,1.2,0.37,0.03,0.08,1.18,0.25,0.15,0.67,0.46,0.85,0.44,0.1,0.14,0.07,0.25,0.14,0,0.07,0.28,0,0.27,0.29,0.14,0.11,0.18,0.07,0.582941176,0.140666667,0.000942092,0.043936257

22881,ANKRD6,0.79,0.49,0.24,1.9,0.87,0.47,1.62,0.44,0.39,0.51,5.68,0.48,0.65,0.47,0.41,0.44,0.77,6.92,1.22,0.85,2.04,0.61,3.49,4.47,0.78,0.99,2.05,0.49,3.29,5.03,1.77,2.85,0.977647059,2.456666667,0.000949259,0.043936257

11123,RCAN3,3.28,4.05,2.8,3.04,1.1,3.09,1.71,3.84,4.77,2.86,2.72,2.89,1.66,4.16,2.32,4.65,4.94,1.72,2.42,2.48,0.12,2.55,0.47,8.91,0.57,0.49,0.33,0.99,0.99,0.95,2.86,1.16,3.169411765,1.800666667,0.000950286,0.043936257

25759,SHC2,13,7.92,9.16,42.21,38.38,3.23,1.52,7.09,11.78,11.4,29.1,9.56,12.26,9.2,6.32,21.79,10.59,3.64,0.29,0.03,4.14,3.94,23.98,11.83,1.88,1.36,0.17,10.74,1.84,0.32,2.48,0.28,14.38294118,4.461333333,0.000951289,0.043936257

2151,F2RL2,104.88,2.34,11.41,2.01,0.52,3.44,5.68,33.52,11.99,3.41,11.43,86.17,104.17,3.98,8.39,3.23,27.94,1.46,0.04,0.02,2.86,2.85,0.08,0.1,1.56,0.34,2.32,2.88,11.69,3.59,0.91,26.37,24.97117647,3.804666667,0.000951289,0.043936257

1410,CRYAB,7.11,0.28,0.19,1.19,85.6,0.44,44.73,5.21,56.19,0.59,22.63,6.02,4.31,3.3,10.85,0.34,20.18,127.03,1214.1,141.38,2.28,5.3,4.53,91.2,58.2,911.43,244.79,10.25,42.42,66.92,29.41,67.36,15.83294118,201.1066667,0.000951289,0.043936257

57473,ZNF512B,21.58,13.66,12.4,22.35,23.36,13.5,9.23,22.6,19.29,9.65,12.62,13.92,8.53,18.79,13.78,15.21,23.51,10.47,7.81,9.37,12.61,12.6,2.1,10.36,12.79,8.41,8.26,16.31,11,9.03,12.99,10.35,16.11647059,10.29733333,0.000951289,0.043936257

79692,ZNF322,7.47,7.27,7.57,8.82,8.7,7.06,5.36,7.12,8.39,7.14,7.48,6.66,7.16,7.64,6.05,7.1,7.58,5.59,4.32,4.97,5.99,3.5,2.74,7.74,4.92,4.33,4.58,7.67,5.86,6.08,7.28,5.57,7.327647059,5.409333333,0.000951289,0.043936257

57688,ZSWIM6,5.77,3.51,5.96,12.65,9.04,4.48,4.53,5.05,4.41,5.38,4.68,10.38,8.39,7.48,4.62,4.54,6.55,3.38,5.11,1.17,5.84,10.71,1.33,5.17,3.07,3.28,3.14,4.03,2.69,1.8,3.48,1.73,6.318823529,3.728666667,0.000951289,0.043936257

2055,CLN8,7.56,4.88,4.81,5.71,6.09,4.42,4.72,6.78,5.27,7.16,4.3,4.91,3.82,4.26,3.71,6.8,4.51,4.43,2.12,3.02,3.96,7.36,1.29,3.66,2.54,5.12,3.38,3.01,3.16,2.92,4.94,2.87,5.277058824,3.585333333,0.000951289,0.043936257

4256,MGP,1.11,1.91,4.12,0.06,0.11,7.57,34.75,3.84,0.25,0.28,551.81,6.59,1.19,0,1.71,0.43,0.33,65.12,152.2,1.85,301.48,11.64,26.86,0.19,29.48,13.29,4.23,8.48,54.91,82.05,12.54,498.03,36.23882353,84.15666667,0.000951289,0.043936257

10127,ZNF263,8.41,9.03,8.22,9.93,11.56,9.22,7.23,9.39,9.33,7.98,10.82,10.33,9.31,11.23,8.87,7.84,9.06,6.2,5.25,5.81,7.71,5.66,4.85,8.43,9.55,6.33,6.51,10.23,7.56,8.04,9.18,7.06,9.28,7.224666667,0.000951289,0.043936257

9919,SEC16A,19.2,16.78,18.59,17.7,19.44,19.8,15.6,16.9,15.42,16.72,16.48,16.83,15.69,23.23,25.79,19.57,20.14,16.07,13.31,11.95,19.05,17.85,16.41,14.43,16.28,12.49,16.24,16.14,14.54,13.67,18.26,11.34,18.46352941,15.202,0.000951289,0.043936257

79858,NEK11,0.52,0.54,0.4,1.17,2,0.67,1.13,2.5,1.77,1.38,1.86,2.62,0.82,2.4,0.32,0.68,1.37,0.95,2.38,4.7,2.17,2.91,0.07,3.08,4.9,1.49,3.1,3.01,2.28,3.83,2.53,3.67,1.302941176,2.738,0.000951289,0.043936257

4055,LTBR,8.01,24.81,11.76,39.39,74.48,53.15,16.12,9.69,15.33,15.35,41.31,0.15,0.09,53.4,1.19,1.68,25.41,8.69,60.54,68.49,58.25,66.23,68.43,34.65,80.74,35.65,55.61,38.74,38.58,63.83,43.78,67.71,23.01882353,52.66133333,0.000951289,0.043936257

57711,ZNF529,3.73,2.61,3.01,5.4,5.02,3.24,2.84,4.09,4.14,4.63,3.69,3,2.73,4.9,2.51,3.53,3.03,2.38,3.53,2.59,1.84,1.53,0.67,2.97,2.63,2.23,1.85,5.32,2.47,2.1,3.46,2.04,3.652941176,2.507333333,0.000951314,0.043936257

388335,TMEM220,0.75,0.62,0.63,1.32,1.53,0.8,0.7,0.98,0.87,1.07,0.31,0.58,0.09,1.69,0.25,0.52,0.88,0.88,3.5,1.61,0.94,0.85,6.46,4.12,1.65,1.6,0.67,1.5,1.31,2.05,1.11,1.57,0.799411765,1.988,0.000951314,0.043936257

3234,HOXD8,0,0,0,0.1,0,0,5.31,0,0,0.07,0,1.22,3.98,3.41,0,0,0.06,0.05,5.84,10.75,5.82,0.05,0.09,8.95,2.99,2.44,4.53,0.08,7.83,8.22,3.24,0.05,0.832352941,4.062,0.000969978,0.044692376

93166,PRDM6,0.02,0,0,0.1,0,0.09,0,0,0.03,0.09,0.17,0,0.03,0.06,0.03,0.02,0.09,0.41,0.71,0.16,0.05,0,0,0.06,0.58,0.19,0.27,5.08,0.91,0.42,1.4,0.37,0.042941176,0.707333333,0.000984269,0.045067854

51302,CYP39A1,0,0,0,0.27,0.03,1.03,0,0,0,0,4.4,0,0,0,0,0,0,0.68,0.24,0.66,0.64,0,1.84,0.76,1.08,0.21,0.04,0.03,0.03,0.47,0.28,0.08,0.337058824,0.469333333,0.000984825,0.045067854

23498,HAAO,1.09,0.14,0.89,0,0,0.06,0.06,1.16,0,0,0.12,0.07,0.5,0.15,0,0,0,0.61,1.37,0.5,0.88,0,5.94,0.88,2.4,0.58,0.07,5.41,2.91,0.21,1.93,2.88,0.249411765,1.771333333,0.000990741,0.045067854

219770,GJD4,0,0,0,0,0,0,0,0,0,0,0,0.05,0,0.25,0,0,0.12,0,0.22,0,0.09,0.23,0,0,1.01,1.36,0.11,0.42,0.76,0.13,0.53,0.17,0.024705882,0.335333333,0.001003118,0.045067854

1746,DLX2,17.19,11.06,13.77,0,0.07,6.07,0.27,14.26,6.11,0.08,1.63,7.71,1.46,0.04,3.29,3.67,18.33,0.17,0,0.07,0.06,0.08,0,0.19,0.64,0.39,0.25,0.06,0.09,0.03,0,0.04,6.177058824,0.138,0.001003363,0.045067854

29895,MYLPF,0.11,0,0.2,0,0,0,0,0,0,0.59,0,0,0,39.17,0,0,0,2.45,0,0.37,0,0.14,0,1.9,0.93,2611.38,0.78,5.19,83.35,0.94,0.7,0.27,2.357058824,180.56,0.001003755,0.045067854

93664,CADPS2,0.06,0,0,1.27,0.9,0.95,0.13,0.01,0.01,0,0,0.06,0.05,2.21,0.09,0.17,0.26,1.66,0.86,0.07,10.64,2.89,4.66,0.63,1.87,0.48,0.38,0.47,0.41,0.88,1.92,2,0.362941176,1.988,0.00100552,0.045067854

50636,ANO7,0.38,0.27,0.24,0.61,0.69,0.22,0.65,0.24,0.18,0.57,0.5,0.57,0.61,0.51,0.31,0.44,0.35,0.21,0.14,0.25,0.18,0.13,0.13,0.08,0.12,0.08,0.3,0.42,0.39,0.37,0.35,0.1,0.431764706,0.216666667,0.001010925,0.045067854

26045,LRRTM2,0,0.13,0.03,0,0.31,0.01,0,0.17,0,0.17,0,0,0,0.02,0.05,0,0,2.12,0.33,0.03,0.49,0.18,0.05,0.08,1.08,0.03,0.55,0.31,0.15,0.12,0.02,0.02,0.052352941,0.370666667,0.001011529,0.045067854

219833,C11orf45,1.56,0.54,0.33,0.83,0.02,0.42,0.86,0.67,0.6,0.28,0.92,0.39,1.31,0.5,0.78,0.58,0.84,0.28,0.09,0.48,0.41,0.1,0.08,0.59,0.56,0,0.36,0.02,0.21,0.44,0.22,0.24,0.672352941,0.272,0.00101635,0.045067854

29964,PRICKLE4,7.07,8.51,4.25,24.06,32.82,4.64,3.45,12.76,7.15,4.55,9.12,0.84,0.72,10.61,3.7,3.99,2.69,1.63,1.07,0.68,5.63,2.86,1.12,2.34,1.76,0.8,0.82,5.95,0.85,2.55,2.67,0.8,8.29,2.102,0.001017437,0.045067854

728841,NBPF8,15.89,15.83,16.26,19.35,23.1,14.66,9.55,15.02,12.51,22.58,13.23,6.08,9.43,23.86,18.73,23.56,18.9,5.62,8.88,6.84,7.2,9.46,4.63,9.06,20.6,7.97,10,15.7,9.06,9.24,10.96,13.65,16.38470588,9.924666667,0.001017437,0.045067854

80312,TET1,0.95,0.65,0.96,0.8,0.62,0.72,0.31,0.91,0.76,0.57,1.53,0.47,0.51,0.94,1.3,1.93,1.19,0.46,0.52,0.33,0.14,0.1,0.07,0.35,0.2,0.43,0.58,0.9,0.93,0.46,0.84,0.19,0.889411765,0.433333333,0.001017437,0.045067854

80055,PGAP1,3.93,3.13,4.22,1.48,1.59,2.52,2.41,4.56,3.21,2.89,3.06,3.67,3.93,1.39,2.96,6.47,2.86,2.81,1,2.5,1.7,1.52,0.19,2.31,2.54,1.35,1.25,2.32,2.48,2.26,2.33,2.83,3.192941176,1.959333333,0.001017437,0.045067854

440823,MIAT,11.93,6.99,25.91,0.08,0.11,1.04,2.98,4.35,3.98,1.28,6.74,13.71,5.73,0.68,4.11,29.17,1.68,0.62,0.03,0.23,0.03,0.02,0.26,0.04,4.72,0.15,0.22,3.1,0.36,0.18,3.88,0.57,7.086470588,0.960666667,0.001017437,0.045067854

167691,LCA5,1.37,1.95,1.14,0.75,0.87,1.73,1.19,2.05,0.73,0.92,1.88,1.09,1.1,1.27,1.34,1.24,1.12,2.25,1.35,1.43,1.49,1.49,0.09,3.84,2.4,1.97,2.03,1.71,2.54,2.35,2.63,2.5,1.278823529,2.004666667,0.001017437,0.045067854

400931,MIRLET7BHG,0.61,0.58,0.57,1.05,1.33,0.65,1.84,0.94,0.63,0.68,1.08,0.49,0.46,1.05,0.73,0.64,0.91,2.24,12.6,1.66,0.59,1.06,0.31,1.2,7.28,2.21,3.26,1.21,1.36,1.68,2.63,3.23,0.837647059,2.834666667,0.001017437,0.045067854

161198,CLEC14A,0.03,0.15,0,8.96,1.32,0,0.99,0.03,0.07,0.16,0.03,0,0,0.08,0,0.03,0.12,0,0.26,0.44,225.54,84.44,2.55,0.04,0.46,2.88,0.28,3.26,13.07,3.19,4.78,3.65,0.704117647,22.98933333,0.001037953,0.045872346

4620,MYH2,0,0,0,0,0,0,0,0,0,0.06,0,0,0,0.25,0,0,0,0.05,0,1.7,0,0,0,0.01,0.06,43.24,0.05,0.1,1.01,10.74,0.05,0.06,0.018235294,3.804666667,0.001066749,0.046916682

159963,SLC5A12,2.66,3.03,4.87,0.24,0.06,0,0.06,0.06,0.05,0.34,0.12,0.2,0.06,0.05,0.28,0.06,0.07,0,0,0.04,0,0,0.02,0,0.17,0.03,0.03,0.05,0.17,0.22,0.01,0,0.718235294,0.049333333,0.001066841,0.046916682

5241,PGR,0,0,0,0,0.01,0,0,0,0,0,0,0,0,0,0,0,0,0,0.02,0.01,0.01,0.01,0,0,0.01,0,0,0.1,0,0.02,0.18,0.13,0.000588235,0.032666667,0.001068805,0.046916682

401190,RGS7BP,0,0,0,0,0.07,0,0.79,0,0,0,0,0,0,0,0,0,0,0.28,0,0,3.55,0.58,0.12,0,0,0.02,0.02,0.29,0.26,1.75,0.03,0.29,0.050588235,0.479333333,0.00107537,0.046998983

3203,HOXA6,0,0,0,0,0,0,0,0,0,0,0,0,0.71,6.89,0,0,0,0,5.75,0.84,2.86,0.12,0,1.25,5.07,0.32,2.61,2.05,0,1.49,6.91,0,0.447058824,1.951333333,0.001078255,0.046998983

153364,MBLAC2,2.61,2.04,2.44,2.95,2.81,2.89,2.95,3.03,2.94,3.53,2.48,2.81,3.35,2.27,1.75,2.23,4.1,0.58,1.85,2.36,2.13,2.07,0.78,4.8,1.88,1.12,1.85,2.67,1.08,2.19,2.04,2.15,2.775294118,1.97,0.001084346,0.046998983

116064,LRRC58,8.87,6.27,7.09,7.39,6.52,10.59,7.11,10.59,8.67,10.66,12.15,9.72,10.78,8.44,8.3,10.36,9.42,5.46,5.61,6.96,6.07,5,2.43,10.65,7.13,4.69,6.39,8.88,6.27,8.18,7.38,6.51,8.995882353,6.507333333,0.001086644,0.046998983

64426,SUDS3,11.32,9.97,8.93,10.33,9.54,9.36,6.91,10.97,9.16,7.92,9.34,9.33,7.79,9.56,8.36,11.21,8.82,6.99,5.93,6.99,8.95,7.19,3.79,16.14,7.66,8.22,6.36,7.79,7.74,7.4,9.27,6.32,9.342352941,7.782666667,0.001086644,0.046998983

55365,TMEM176A,0,0,0,0,0,0,0,0,0,0,0,0,0,0.29,0,0,0,0.15,0,1.18,0,0,310.08,0,0,12.96,0,15.03,1.18,3.17,0.62,0.09,0.017058824,22.964,0.001102915,0.046998983

28959,TMEM176B,0,0,0,0,0,0,0,0,0,0,0,0,0,0.15,0,0,0,0.06,0,3.47,0.06,0,329.47,0,0,16.1,0,9.35,1.18,2.45,0.45,0,0.008823529,24.17266667,0.001102915,0.046998983

3206,HOXA10,0,0,0,0,0,0,9.05,0,0,0.04,0.1,0.04,1.75,8.47,0,0.06,0.04,0,20.2,16.39,7.3,1.35,0.11,2.76,28.88,7.15,18.88,0.03,0.12,11.17,10.47,0.04,1.15,8.323333333,0.001104018,0.046998983

139065,SLITRK4,0,0,0,2.22,4.08,0.01,0.03,0,0,0,0.01,0,0,0.02,0,0,0,0.09,0.17,0.01,7.12,1.06,0.05,0,0.14,4.34,0.02,0.01,0.62,0.12,1.61,0.23,0.374705882,1.039333333,0.001105901,0.046998983

56100,PCDHGB6,0.79,48.4,28.92,12.98,57.93,3.82,9.22,0.27,19.22,29.36,30.69,96.42,7.07,18.05,0.64,36.7,20.18,2.32,2.26,3.03,1.86,0.25,1.97,3.22,5.35,0.66,2.25,4.56,1.69,3.46,4.07,1.2,24.74470588,2.543333333,0.001114051,0.046998983

79710,MORC4,7.26,11.26,23.99,12.75,12.6,13.44,13.22,21.89,16.92,28.24,13.55,14.18,24.5,18.56,14.44,7.39,11.29,16.47,7.32,8.46,5.16,5.58,2.98,5.72,8.45,14.46,9.64,7.12,12.57,9.43,13.37,10.5,15.61647059,9.148666667,0.001114051,0.046998983

54874,FNBP1L,26.42,13.39,10.31,54.43,43.71,15.38,7.3,22.53,20.4,10.23,22.77,12.43,11.33,17.17,19.95,16.74,14.99,19,3.3,6.36,20.34,19.56,3.75,12.81,6.23,6.6,4.47,8.14,9.29,4.5,14.22,6.93,19.96941176,9.7,0.001114051,0.046998983

205564,SENP5,7.08,6.53,6.45,9.34,7.36,6.81,6.16,7.48,6.31,5.53,8.51,6,5.51,8.37,5.72,4.58,6.19,6.27,4.83,4.71,5.93,5.67,2.95,7.52,4.51,5.65,5.19,5.76,5.03,4.3,6.11,3.87,6.701764706,5.22,0.001114051,0.046998983

3150,HMGN1,126.27,97.19,121.54,116.67,106.89,108.27,91.39,128.26,116.96,106.42,103.9,109.19,96.57,179.23,109.04,106.44,107.84,113.41,60.05,69.56,154.17,93.41,35.32,82.69,63.59,54.45,88.88,93.54,95.97,60.28,125.15,81.13,113.6511765,84.77333333,0.001114051,0.046998983

56105,PCDHGA11,0.45,9.36,0.09,6.3,2.64,11.55,0.56,0.19,0.03,0.31,0.48,0.07,0.14,4.28,1.98,0.59,0.35,8,9.03,11.91,5.28,1.79,0.4,4.68,20.38,1.91,7.52,8.11,4.23,5.07,18.25,9.56,2.315882353,7.741333333,0.001114051,0.046998983

100271722,LINC00899,0.82,0.74,0.12,0.58,0.68,0.8,0.34,0.98,0.16,0.18,0.84,0.21,0,1.52,0.22,0.32,0.23,0.39,2.89,1.19,0.96,0.44,0.26,1.08,1.47,0.71,1.03,3.16,0.78,1.63,1.1,2.83,0.514117647,1.328,0.001114051,0.046998983

5010,CLDN11,0.96,0.32,0.13,11.46,8.67,0.7,19.99,9.04,0.37,0.38,0.43,3.37,2.35,30.08,0.28,0.18,0,2.29,1.77,167.72,7.5,372.33,1.01,0.59,172.79,32.34,2.63,153.77,10.99,152.41,145.84,114.25,5.218235294,89.21533333,0.001114051,0.046998983

54754,NUTM2F,0.11,0.34,0,0,0,0.15,0,0,0,0.04,0.37,0.04,0.06,0.04,0.17,0.06,0.09,0,0,0,0,0,0,0,0,0,0,0.06,0,0,0,0,0.086470588,0.004,0.001114086,0.046998983

6424,SFRP4,0.12,0,0,0,0,0,0.39,0,0,0.06,0,2.38,0.06,0,0,0,0.34,27.84,0,0.48,0,0.03,0.23,0,51.64,6.59,36.97,3.63,73.63,111.35,13.4,10.58,0.197058824,22.42466667,0.001136359,0.04783506

441459,ANKRD18B,0.34,0.88,0.7,0.37,0.02,1.24,0.23,0.39,0.13,0.38,0.14,0.97,0.48,0.54,0.67,0.77,1.2,0,0,0.08,1.1,0.21,0.19,0.02,0.13,0.86,0.02,0.12,0.03,0,0.02,0.07,0.555882353,0.19,0.00114568,0.047906117

60385,TSKS,0,0.07,0,0,0.04,0,0,0,0,0.53,0,0,0,0,0,0.08,0,0.04,0.3,1.37,0.04,0.1,0,0,0.05,0.14,0,0.36,0.7,1.75,0.11,0.63,0.042352941,0.372666667,0.001152137,0.047906117

643401,LINC01021,0.67,1.1,1.1,7.9,3.02,10.03,0.15,0.23,0.05,1.2,12.9,0.27,6.62,12.52,13.03,2.71,1.5,0.86,0,2.85,0,0.33,0,0.09,0.18,0.31,0.22,0.18,0.18,0.36,0.06,0.58,4.411764706,0.413333333,0.001152922,0.047906117

80128,TRIM46,5.32,3.04,4.36,6.09,2.32,2.9,3.19,5.67,4.29,4.94,5.23,4.78,3.84,4.44,4.39,4.06,4.78,3.76,1.56,1.56,2.09,3.1,0.09,2.75,5.58,0.43,1.88,2.09,2.61,1.81,6.26,1.56,4.331764706,2.475333333,0.001156555,0.047906117

29103,DNAJC15,0,0,0.13,4.88,6.22,0.03,8.59,12.68,13.83,0.17,0.11,0.34,0.06,16.37,0.03,0.03,0.23,5.69,9.69,7.75,14.71,14.51,6.01,18.21,16.58,9.38,10.69,5.01,10.61,9.66,8.84,13.22,3.747058824,10.704,0.001157768,0.047906117

63910,SLC17A9,0,20.07,0,1.21,6.81,1.36,0.03,3.81,2.56,30.21,2.21,0.07,0,0.04,0.5,1.25,0.14,3.73,23.49,6.23,8.98,12.15,47.37,1.42,11.21,5.19,8.24,9.86,2.52,2.23,9.46,7.61,4.133529412,10.646,0.001158981,0.047906117

150356,CHADL,0.6,0.12,0.47,1.19,0.62,0.11,0.12,0.1,0.1,0.15,1.48,0.07,0.11,0.46,0.45,0.26,0,0.86,1.54,1.03,0.55,0.52,0.22,4.03,0.74,0.9,0.69,0.28,0.7,1.16,0.79,0.65,0.377058824,0.977333333,0.001160196,0.047906117

100507321,ERVK13-1,2.32,1.42,2.26,3.81,3.1,1.74,1.86,2.87,1.98,2.27,2.23,1.57,1.85,2.91,1.41,2.3,2.31,1.27,2.24,1.11,0.78,1.08,0.71,2.26,2.24,1.34,0.92,1.61,1.01,1.23,2.69,1.41,2.247647059,1.46,0.001160196,0.047906117

26240,FAM50B,0.18,2.99,2.09,2.37,1.03,3.61,0.63,11.19,1.54,1.48,5.35,0.05,0,8.29,1.85,0.05,0,6.41,4,5.28,7.3,4.12,2.19,6.99,8.64,3.92,5.91,4.88,7.6,5.23,4.89,3.71,2.511764706,5.404666667,0.001161411,0.047906117

26272,FBXO4,2.81,2.62,1.66,1.57,2.28,4.02,3.77,2.09,2.6,2.62,5.43,0.11,0,5.07,6.33,2.56,1.15,3.65,4.7,6.48,4.89,2.48,4.39,4.68,5.44,4.38,4.91,6.11,5.39,6.01,5.16,6.07,2.746470588,4.982666667,0.001162627,0.047906117

11005,SPINK5,1.26,1.69,0,4.36,9.21,1.55,0.92,2.93,1.76,0.48,1.34,0.49,0,1.43,0.43,1.19,0,0.06,0,0,0.24,0.07,0.15,0.06,0.13,0.44,0,0.07,0.11,0.07,0.05,0.03,1.708235294,0.098666667,0.001192719,0.048990502

4917,NTN3,0.7,0.82,0.7,0,0.04,0.14,0.16,0.75,0.82,0.62,1.14,1.3,0.69,0.84,1.24,0.96,0.28,0.59,0.14,0.36,0,0,0,0,0.25,0,0.13,0.43,0.2,0.24,0.64,0.04,0.658823529,0.201333333,0.001193971,0.048990502

375316,RBM44,0.13,0.28,0.1,0.46,0.25,0.07,0.09,0.11,0.25,0.11,0.07,0.24,0.19,0.2,0.16,0.1,0.21,0.07,0.14,0.07,0.03,0.06,0.03,0.08,0.05,0.24,0.08,0.09,0.08,0.03,0.16,0.08,0.177647059,0.086,0.001205278,0.049350566

3232,HOXD3,0,0,0,0,0,0,0.37,0,0,0,0,0.04,0.12,0.88,0,0,0,0.95,2.44,1.75,2.59,0,0,2.06,0.34,0.18,0.21,0,3.02,1.21,2.31,0,0.082941176,1.137333333,0.0012166,0.049656535

100874323,HOXA10-AS,0,0,0,0.07,0,0,10.35,0,0,0.08,0,0,1.2,13.79,0,0,0,0,16.75,16.13,6.81,1.87,0.12,4.52,25.19,6.93,15.03,0.06,0.12,8.34,8.89,0,1.499411765,7.384,0.001217846,0.049656535

60468,BACH2,0.05,0,0.01,0.41,0.48,0.04,0.03,0.01,0.02,0,0.51,0.18,0.14,0.16,0.07,0.04,0.01,1.94,0.15,0.16,0.52,0.39,0.92,0.57,0.15,0.17,0.47,2.34,1.85,0.47,2.39,0,0.127058824,0.832666667,0.001228104,0.049970251

55676,SLC30A6,10.47,6.19,6.47,8.04,8.46,8.61,9.19,10.4,7.34,6.91,8.52,7.82,6.8,8.09,6.64,6.45,9.67,6.73,5.93,6.26,6.61,5.17,3.97,6.09,7.02,6.8,6.37,6.52,6.59,6.98,7.75,6.56,8.004117647,6.356666667,0.001242193,0.050438194

50624,CUZD1,0,0,0,0.35,0.65,0,1.3,0,0,0,0,0,0,0.13,0.04,0,0,0.15,0.33,0.33,0.59,0.19,0.07,0.25,0.33,0.38,0.23,0.67,0.03,0.26,0.18,0.27,0.145294118,0.284,0.001277081,0.051644703

2444,FRK,0.08,0,0,0,0,0.07,0.11,0,0.03,0,0.08,0,0,0.2,0,0,0,0.08,0,0.14,0,0,0.73,0.33,0.44,0.25,0.09,0.14,0.14,0.14,0.09,0.21,0.033529412,0.185333333,0.00128435,0.051644703

728377,ARHGEF34P,0.97,0,0,0.13,0.18,0.43,1.33,0.49,1.27,0.02,2.22,0.04,0,0.36,0.04,0,0,0.39,1.66,0.71,0.53,0.19,1.51,4.11,1.5,0.14,2.15,0.73,1.99,2.42,2.08,1.06,0.44,1.411333333,0.001299779,0.051644703

23015,GOLGA8A,49.05,22.73,54.79,44.62,22.1,23.79,58.29,42.73,58.84,45.66,46.06,16.11,40.83,72.4,67.19,46.74,40.81,16.52,52.08,6.49,8.47,12.68,2.67,9.96,41.89,28.68,17.8,52.1,21.83,19.22,44.17,15.22,44.27882353,23.31866667,0.001301054,0.051644703

163859,SDE2,6.13,4.79,4.55,8.63,7.36,5.55,3.76,6.01,5.4,6,6.24,4.2,5.24,7.15,5.68,4.22,7.59,5.43,3.25,3.34,4.82,3.41,4.75,5.99,3.33,4.46,4.03,4.6,3.65,4.27,4.8,4.56,5.794117647,4.312666667,0.001301054,0.051644703

54916,TMEM260,5.42,5.54,5.29,7.28,5.68,6.27,3.98,5.45,4,5.22,6.54,5.1,5.13,5.6,4.99,7.11,4.06,3.67,4.75,3.6,3.82,3.62,2.7,6.47,4.64,2.33,3.45,6.16,3.96,4.12,5.15,4.63,5.450588235,4.204666667,0.001301054,0.051644703

372,ARCN1,61.85,64.74,67.89,82.44,63.14,74.5,61.92,64.58,55.61,79.97,64.47,49.56,56.28,84.16,75.04,51.48,82.34,56.34,61.02,47.04,63.42,49.94,29.34,39.52,58.41,42.69,54.31,58.98,58.86,54.08,75.34,49.19,67.05705882,53.232,0.001301054,0.051644703

54908,SPDL1,16.81,4.75,19.42,6.26,6.31,9.99,6.98,10.54,7.06,7.19,8.22,13.25,7.16,10.08,6.49,6.2,34.53,5.14,1.62,11.95,8.3,9.28,0.47,4.54,4.65,4.78,3.21,4.06,5.51,6.33,6.71,4.74,10.66117647,5.419333333,0.001301054,0.051644703

84179,MFSD7,0.57,0.84,0.52,2.57,8.21,2.48,3.08,0.43,0.13,1.48,5.07,0.09,0.05,0.81,0.27,0.72,0.66,1.22,5.8,6.37,3.01,1.87,0.88,1.53,6.87,2.84,3.71,2.54,7.75,7.66,2.91,2.86,1.645882353,3.854666667,0.001301054,0.051644703

388789,LINC00493,18.23,19.73,15.06,22.47,16.84,17.13,15.5,26.08,16.71,15.32,10.45,22.2,22.84,20.35,18.51,15.91,12.45,18.09,25.08,25.05,23.8,16.63,29.79,33.67,23,29.9,17.17,25.06,17.33,20.4,23.89,28.67,17.98705882,23.83533333,0.001301054,0.051644703

51303,FKBP11,18.45,42.89,40.16,6.32,5.8,10.38,39.44,19.87,10.41,30.87,4.12,0.82,0.94,33.43,61.06,14.57,2.79,37.71,160.03,27.4,43.17,34.51,97.6,44.36,50.08,36.01,28.44,35.95,32.31,32.37,37.66,67.27,20.13647059,50.99133333,0.001301054,0.051644703

51599,LSR,0.71,0.46,0.19,0.12,0.24,0.18,0.2,0.76,0.35,0.1,0.27,0.09,0.14,0.3,0.09,0.19,0.84,0.76,0.31,0.77,0.54,1.57,38.07,0.71,0.55,0.67,0.3,0.37,0.71,0.62,0.42,0.45,0.307647059,3.121333333,0.001317276,0.051717134

54922,RASIP1,0.33,0.38,0.19,91.54,109.31,0.26,0.35,0.1,0.2,0.45,0.2,0.08,0.03,0.26,0.1,0.15,0.08,0.33,0.42,0.38,41.44,27.69,1.31,2.78,5.55,0.24,2.21,3.57,1.58,1.51,2.32,1.94,12.00058824,6.218,0.001319981,0.051717134

140886,PABPC5,0.17,0,0.65,0.04,0.34,0.02,0.16,0.02,0,0.19,2.7,0.22,0.03,0.11,0.03,0.04,1.07,0.74,0.91,0.6,0.78,0.08,0.08,2.89,0.85,1.12,0.72,0.21,1.18,1.29,2.03,1.06,0.340588235,0.969333333,0.001321335,0.051717134

7404,UTY,0.02,0.01,0,0.01,0.05,0.04,0.05,0,0.04,0.03,0.05,0.01,0.02,0.04,0.04,0.02,0.02,0.02,2.61,0.02,0.05,0.07,1.5,4.33,0.08,2.62,0.01,3.45,1.98,0.03,3.11,1.9,0.026470588,1.452,0.001323997,0.051717134

10350,ABCA9,0.23,0.16,0.05,0.29,0.5,1.69,10.4,0.92,0.34,0.02,0.13,0.07,0.17,0.21,0.1,0.07,0.09,0.09,0.18,4.47,4.62,0.92,0.57,0.38,4.69,0.4,1.53,5.83,6.98,44.97,7.27,10.57,0.908235294,6.231333333,0.001324046,0.051717134

126868,MAB21L3,1.32,0.8,1.12,2,1.36,0.74,1.18,0.99,1.15,0.7,1.16,0.85,1.43,2.2,0.94,0.97,0.9,0.7,0.54,0.66,0.34,1.16,1.6,0.83,0.84,0.55,0.76,0.47,0.33,1.11,0.65,0.62,1.165294118,0.744,0.001325403,0.051717134

9481,SLC25A27,0.27,0.1,0.25,1.58,0.52,1.94,0.18,0.33,0.25,0.07,4.65,1,0.2,0.1,0.24,0.26,0.17,0.57,3.72,2.53,0.56,0.34,1.23,7.16,4.37,0.87,0.79,1.1,0.53,1.44,1.52,1.63,0.712352941,1.890666667,0.001325403,0.051717134

7784,ZP3,0.33,0.37,1.45,1.9,0.62,0.06,0.07,0.29,0.32,0.58,0.5,0,0.07,2.83,0.36,0.37,0.67,1.76,0.97,2.3,0.53,1.39,0.87,0.38,1.37,1.31,2.12,1.83,1.35,1.77,2.45,1.74,0.634705882,1.476,0.001325403,0.051717134

374383,NCR3LG1,1.05,0.18,1.82,1.43,0.7,1.31,0.66,1.36,1.11,0.73,0.53,1.44,2.04,1.82,2.61,3.33,1.53,0.28,0.56,0.2,1.85,0.84,0.41,0.05,0.91,0.63,0.61,0.75,0.49,0.52,0.51,0.39,1.391176471,0.6,0.001326761,0.051717134

5446,PON3,0,0,0,0.07,0.4,0,0,0,0,0,0,0,0,0.17,0,0,0,0,0.63,0.2,1.14,0,67.5,0,1.18,0,0.07,0.06,0.17,1.2,0.11,0.97,0.037647059,4.882,0.001340881,0.052163233

9229,DLGAP1,0.03,0.21,0,0.01,0,0,12.54,0.02,0,0,0.01,0.38,0.02,0,0,0,0,3.5,0.22,0.14,0.19,0,0.15,2.18,0.07,0.58,0.02,0.23,0.39,0.33,0.62,0.04,0.777647059,0.577333333,0.001357718,0.052713006

9844,ELMO1,0,0,0,3.85,2.14,0,0.09,0,0.02,0,0,0,0,0.13,0,0,0.02,1.12,0.95,0.02,10.52,5.9,1.66,0.07,0,8.27,0.03,0.04,0.35,0.07,0.2,0.5,0.367647059,1.98,0.001364833,0.052883879

6252,RTN1,0.04,0.04,0,0.14,0.16,0.02,0,0,0.03,0.06,0.02,0.05,0.11,0.06,0.1,0.07,0.08,8.42,0.1,0,0.21,3.58,0.17,0.1,0.06,1.72,0.02,0.69,0.18,0.31,0.88,0.46,0.057647059,1.126666667,0.001385287,0.053518432

126820,WDR63,0,0.06,0.04,0,0.08,0.33,0.31,0.11,0.03,0.19,0.25,0,0.06,0.1,0.06,0.02,0.06,1.47,0.2,0.94,0.02,0.32,0,0.88,1.13,0.61,0.3,0.36,0.09,0.31,1.22,0.69,0.1,0.569333333,0.001386701,0.053518432

64080,RBKS,0.94,0.56,0.92,0.13,0.47,2.26,0.67,0.71,0.07,0.98,1.67,0.95,1.32,0.92,0.9,0.19,0.55,1.81,2.95,1.3,0.84,1.43,11.54,2.68,1.95,1.33,1.21,1.4,0.96,1.26,1.57,0.67,0.835882353,2.193333333,0.00141518,0.05424322

4253,CTAGE5,9.4,8.2,9.5,5.92,6.08,6.24,7.4,9.07,6.51,9.58,6.32,6.22,7.11,7.55,9.09,7.09,6.58,5.87,6.78,4.41,6.05,5.75,19.29,6.45,6.22,5.36,5.81,4.66,6.37,4.52,6.44,4.38,7.521176471,6.557333333,0.001416614,0.05424322

55209,SETD5,23.27,24.24,17.18,22.11,21.55,16.36,13.1,22.73,19.35,20.8,24,16.14,14.67,23.26,25.07,24.16,18.4,14.43,17.65,14.27,15.73,11.19,7.41,17.84,17,12.61,11.98,20.71,17.55,14.67,21.73,14.98,20.37588235,15.31666667,0.001416614,0.05424322

3176,HNMT,0.32,1.61,0.45,4.45,5.81,16.05,0.02,2.21,1.7,2.11,0.4,0.2,4.36,7.67,1.39,0.24,0.03,3.74,3,5.81,4.41,1.73,4.48,46.23,12.38,6.28,4.68,5.76,9.76,13.12,2.39,18.35,2.883529412,9.474666667,0.001416614,0.05424322

150221,RIMBP3C,0.08,0.12,0.1,0,0,0.06,0.02,0.04,0,0,0,0,0,0,0.12,0.07,0.18,0,0,0,0,0,0,0,0,0,0,0,0,0,0,0,0.046470588,0,0.001420036,0.054267648

2327,FMO2,0.01,3.59,0,0,0,2.61,0,0,0,0.05,0.06,0.03,0.02,0,0,0.01,0,0.37,0.09,0.08,0,0.02,0.03,0.02,3.84,0.91,0.24,18.97,0.59,0.89,1.4,33.35,0.375294118,4.053333333,0.001449489,0.055145046

5031,P2RY6,0,0.05,0.08,0,0,0,0.62,0.03,0.04,0.04,0.03,0.04,0.08,0,0.04,0,0,0.13,4.06,0.6,0.18,0.04,0.28,0,3.13,0.33,1.38,0,0.08,0.14,0.08,0.11,0.061764706,0.702666667,0.001479872,0.055145046

100505624,GTF3C2-AS1,1.09,0.97,0.63,0.58,1.07,0.89,0.68,0.62,0.74,0.71,0.75,0.21,1.35,1.09,0.87,1.09,0.91,0.78,0.5,0.3,0.44,0.44,0.52,0.76,0.25,0.61,0,0.89,0.74,0.42,0.47,0.22,0.838235294,0.489333333,0.00150298,0.055145046

360132,FKBP9P1,0.06,0.54,0.2,0.1,0.31,0.09,0.74,0.93,0.62,0.31,0.48,0.57,0.72,0.34,0.04,0.21,0.04,0.96,2.24,1.74,0.18,1.76,0.07,1.49,0.57,0.57,0.87,0.82,2.44,4.44,0.51,2.39,0.370588235,1.403333333,0.001504489,0.055145046

2657,GDF1,0.83,0.49,1.15,1.33,2.84,1.62,0.03,0.84,0.76,0.34,3.26,1.02,1.23,0.74,0.96,3.49,1.15,0.34,0.26,0.43,0.04,0.03,0,3.35,0.12,1.44,0.21,0.39,0.21,0.13,0.11,0.11,1.298823529,0.478,0.001505999,0.055145046

100289019,SLC25A25-AS1,2.2,1.01,1.73,3.97,2.79,1.42,1.13,1.99,2.19,1.48,1.42,0.85,0.56,2.8,2.67,3.05,1.47,1.08,1.4,0.41,0.7,1.13,1.39,2.04,0.77,1.17,0.84,0.99,0.68,0.57,1.34,1.13,1.925294118,1.042666667,0.001505999,0.055145046

79689,STEAP4,0,0,0.02,0.02,0.04,0.04,0,0,0,0,0,0.02,0,0,0,0,0,0.02,3.35,0.37,0,0,60.45,0,2.92,2.45,0.58,0,0.04,1.67,0.46,1.67,0.008235294,4.932,0.001506781,0.055145046

5789,PTPRD,0,0.01,0.01,3.49,1.08,0.02,0.04,0,0,0.01,1.42,0,0,4.98,0,0,0.01,3.8,2.88,0.03,3.39,0.03,0.3,0.02,1.64,0.73,0.3,1.37,9.05,3.93,3.85,0.15,0.651176471,2.098,0.001507068,0.055145046

440295,GOLGA6L9,3.04,1.71,2.38,6,5.47,1.08,1.76,3.06,3.07,2.07,2.76,0.94,1.75,4.54,3.21,2.68,2.04,2.43,1.06,0.56,1.27,1.11,0.98,3.03,1.26,0.59,1.97,1.06,1.27,0.76,2.15,1.33,2.797647059,1.388666667,0.001510534,0.055145046

59343,SENP2,10.81,11.3,12.15,15.98,13.2,11.71,10.83,12.15,11.03,12.1,16.66,12.88,10.76,12.22,9.6,10.93,10.81,9.21,7.93,9.07,12.04,9.06,7.01,12.64,8.69,12.72,8.79,11.07,7.17,7.16,9.79,8.09,12.06588235,9.362666667,0.001510534,0.055145046

25979,DHRS7B,3.07,1.37,1.76,7.4,9.54,3.7,5.67,6.18,3.81,2.7,3.69,3.51,3.58,9.23,2.67,2.35,4.39,10.02,6,7.84,6,5.38,5.61,14.94,5.4,8.11,7.9,5.84,5.82,10.11,5.79,9.03,4.389411765,7.586,0.001512047,0.055145046

7681,MKRN3,0,0,0,0,0,0,0,0,0,0,0,0,0,0,0,0,0.07,0.85,0.02,0.06,0,0,0,1.23,0,0.06,0.06,0.47,0.24,0,0.69,0,0.004117647,0.245333333,0.001512646,0.055145046

29116,MYLIP,5.08,10.27,10.19,27.27,18.19,19.55,0.5,8.34,3.99,2.69,14.28,16.18,3.05,28.05,4.16,17.26,4.58,1.56,3.16,2.18,10.97,1.55,1.72,7.51,1.11,1.86,0.67,5.4,3.52,1.77,3.56,4.53,11.39,3.404666667,0.001515435,0.055145046

23262,PPIP5K2,9.11,7.84,8.56,9.78,10.14,7.97,9.96,10.01,8.73,8.78,8.83,6.49,7.56,9.21,8.2,8.17,11.78,7.83,7.25,7.81,6.85,5.78,4.08,10.08,8.94,3.38,7.12,7.76,6.13,5.96,9.63,5.74,8.889411765,6.956,0.001515435,0.055145046

10802,SEC24A,11.41,12.8,13.63,9.88,8.52,9.97,15.37,11.7,11.3,15.99,11.57,9.87,11.47,15.33,15.43,8.7,17.31,9.15,11.79,7.69,9.7,10.04,13.77,6.44,7.99,7.36,8.96,11.09,8.14,8,11.52,6.18,12.36764706,9.188,0.001515435,0.055145046

10513,APPBP2,16.49,10.12,8.85,10.14,9.82,11.49,8.24,16.35,8.91,8.37,10.55,10.62,8.86,12.17,7.38,8.57,7.67,7.09,6.67,6.94,9.45,6.45,2.01,11.35,8.66,6.31,5.75,9.36,8.38,7.19,9.35,6.98,10.27058824,7.462666667,0.001515435,0.055145046

200845,KCTD6,4.48,6.27,4.89,4.91,4.5,6.97,4.1,8.49,5.13,5.87,4.8,6.21,6.17,4.6,7.06,4.09,5.31,4.11,2.87,3.82,2.71,2.5,3.09,5.98,5.29,2.21,2.43,6.7,3.67,3.65,5.32,4.07,5.520588235,3.894666667,0.001515435,0.055145046

3720,JARID2,5.05,4.32,3.58,10.17,10.42,2.67,2.3,9.19,4.43,3.7,3.56,6.68,2.64,11.15,4.58,6.17,5.31,2.05,1.6,2.03,4.14,1.4,1.28,14.93,1.99,2.99,1.69,6.11,2.17,2.52,4.46,1.87,5.642352941,3.415333333,0.001515435,0.055145046

84128,WDR75,14.22,10.34,12.97,22.28,17.93,20.22,9.39,17.39,11.81,17.59,25.16,12.73,12.13,16.63,13.28,13.68,20.13,7.44,9.98,7.94,17.2,14.43,4.99,11.78,11.37,16.26,7.53,15.65,9.01,9.09,11.17,11.75,15.75764706,11.03933333,0.001515435,0.055145046

23518,R3HDM1,8.1,5.91,9.38,8.78,7.15,6.81,6.41,7.05,8.44,6.5,6.84,5.35,5.49,8.06,5.83,6.65,7.65,5.81,4.86,4.93,9.3,5.5,2.59,6.55,4.58,8.05,4.87,5.09,6.32,4.84,6.38,4.48,7.082352941,5.61,0.001515435,0.055145046

8802,SUCLG1,20.02,15.58,18.54,22.28,19.12,28.05,17.77,25.15,17.54,21.96,21.01,19.52,18.66,20.21,15.19,18.79,18.22,26.24,22.02,25.16,30.82,28.17,39.99,49.59,22.63,30.41,17.7,22.13,20.07,19.95,26.97,19.41,19.85941176,26.75066667,0.001515435,0.055145046

653784,MZT2A,44.2,52.32,53.01,56.64,58.65,67.13,29.86,49.25,33.04,67.66,57.94,81.49,72.84,68.98,58.59,34.5,38.71,14.46,34.76,32.66,58.72,27.08,17.52,37.12,34.79,10.42,34.07,39.42,52.06,28.52,64.15,28.98,54.40058824,34.31533333,0.001515435,0.055145046

23593,HEBP2,18.07,21.79,15.75,35.83,25.44,33.86,33.52,26.36,20.11,22.63,24.8,28.43,29.25,22.14,17.23,9.83,16.95,31.32,108.12,40.55,39,42.5,41.58,25.98,35.52,41.34,35.88,17.12,24.41,25.28,44.34,25.18,23.64647059,38.54133333,0.001515435,0.055145046

57804,POLD4,29.6,38.66,28.65,13.01,18.22,36.68,28.68,35.19,24.61,29.75,16.13,22.5,27.18,27.89,25.41,17.19,21.84,34.02,26.5,29.39,33.04,46.96,69.36,37.55,32.3,33.55,36.24,29.69,32.95,41.32,34.64,28.37,25.95235294,36.392,0.001515435,0.055145046

5587,PRKD1,2.56,6.36,0.08,3.56,1.73,1.59,8.18,4.4,3.18,1.23,9.11,0.51,4.07,2.47,2.02,0.41,2.01,8.01,5.05,10.42,3.68,2.85,0.83,13.89,12.14,6.22,10.1,7.81,8.09,7.51,4.33,6.5,3.145294118,7.162,0.001515435,0.055145046

133584,EGFLAM,0,0.13,0.4,0,0.03,0.16,0.18,0,0.1,0.37,0,0.04,0,0.09,0.27,0.19,0,0.41,3.14,0.07,0.37,0.04,0,0.04,8.24,3.71,1.46,3.91,2.24,1.44,20.9,1.3,0.115294118,3.151333333,0.001519333,0.055145046

146439,CCDC64B,0,0,0,0,0,0,0,0,0,0.11,0,0,0,0,0,0,0,0.13,0.07,0,0,0,0.55,0,0.22,0.18,0.05,0,0.15,0.07,0.04,0,0.006470588,0.097333333,0.001519389,0.055145046

443,ASPA,0,0,0,0,0,0,0,0,0,0.54,0,0,0.2,0,0,0,0,0,0.2,3.61,0,0.07,0,73.51,0.49,0,0,1.38,0.25,6.94,0.27,3.05,0.043529412,5.984666667,0.001522385,0.055151093

83851,SYT16,0.18,0.15,0.06,0.07,0.02,0.05,0.22,0,0.38,0.18,0.21,0.73,0.03,0,0.14,0.07,0.06,0.02,0,0,0,0.03,0,0,0,0.05,0,0.02,0.1,0.02,0.18,0,0.15,0.028,0.001529507,0.055278077

374308,PTCHD3,0,0,0,0,0,0,0.12,0,0,0.04,0,0,0,0,0,0,0,0.03,0.03,0.06,0.03,0.07,0,0,0.04,0,0,0.03,0.05,0.05,0.05,0.17,0.009411765,0.040666667,0.001531563,0.055278077

342918,C19orf81,1.11,0.41,0.78,2.56,2.16,0.49,1.29,0.57,0.47,0.66,0.1,0.12,0.37,3.63,1.08,0,0.88,0.11,0,0,0.78,0.36,0,0.12,1.23,0.11,0.11,0.09,0,0,0.35,0.36,0.981176471,0.241333333,0.001548718,0.055793941

126432,RINL,0.36,0.33,0.35,3.19,2.23,0.86,0.18,0.4,0.53,0.49,0.59,0.28,0.15,1.39,0.36,0.07,0.12,0.45,1.16,2.05,3.49,0.72,1.38,2.8,0.86,4.29,0.68,0.87,1.28,1.12,1.02,0.77,0.698823529,1.529333333,0.001611775,0.057958485

339768,ESPNL,0,0.11,0.07,0,0.02,0,0.02,0.05,0.02,0.2,0,0.02,0.08,0.12,0.13,0,0,0.13,3.61,1.68,0.04,0,0,0.04,0.91,1.22,0.18,1.62,1.11,0.62,0.98,0.61,0.049411765,0.85,0.001671105,0.059981266

10875,FGL2,0.54,0.08,0.04,0.02,0.02,0.02,0.85,9.53,0.02,0,10.11,0,0,0.07,0.04,0.02,0.13,0.09,22.45,1.16,0.06,0.51,3.91,1,0.18,1.04,0.31,0.14,0.61,13.03,0.63,2.35,1.264117647,3.164666667,0.00168077,0.060166202

26499,PLEK2,0.05,0,0,0.42,0.11,0,1.23,0.45,1.11,0.07,0.05,0.12,0,1.07,0.06,0.05,0,0.81,0.09,0.77,1.25,9.22,38.72,0.54,0.2,0.78,0.17,0.13,0.86,0.37,4.33,1.08,0.281764706,3.954666667,0.001682431,0.060166202

90594,ZNF439,0.17,0.14,0.07,0.09,0.18,0.03,1.16,1.73,1.48,0.11,0.32,0,0,2.08,0.17,0,0.27,1.73,0.84,1.85,0.33,0.81,0.11,10.47,2.13,1.01,0.9,0.78,1.64,1.43,1.89,1.97,0.470588235,1.859333333,0.001710821,0.060514709

221079,ARL5B,5.94,5.85,6.41,11.54,8.32,2.77,2.73,8.06,5.2,5.92,8.37,3.36,2.86,8.28,5.43,7.41,7.17,1.9,3.36,2.04,5.59,2.11,7.38,2.63,2.77,2.02,2.59,5.14,3.4,3.07,7.25,2.03,6.212941176,3.552,0.00171923,0.060514709

6018,RLF,5.49,5.82,4.77,6.41,7.11,5.83,4.73,4.8,5.75,5.32,5.11,6.94,4.4,7.2,5.53,6.03,4.98,6.02,3.85,4.04,3.53,2.42,1.68,4.76,4.77,3.73,4.85,4.5,5.8,4.35,5.83,4.7,5.66,4.322,0.00171923,0.060514709

7984,ARHGEF5,2.36,0,0.02,0.33,0.19,1.28,2.39,0.39,2.25,0.03,4.2,0.54,0.86,1.75,0,0.3,0.19,2.65,2.13,1.74,0.78,0.37,2.71,5.71,4.35,0.87,4.52,1.66,3.24,3.28,4.22,1.79,1.004705882,2.668,0.00171923,0.060514709

3899,AFF3,26.89,4.17,12.35,0.69,0.1,9.1,5.03,40.28,19.55,1.53,10.34,16.6,7.48,2.72,11.03,19.53,16.45,7.18,0.32,3.23,0.07,3.7,0.07,10.72,0.3,0.37,2.12,0.63,4.39,1.6,1.84,1.54,11.99058824,2.538666667,0.001720914,0.060514709

256329,LMNTD2,2.61,3.49,1.62,0.89,1.42,3.2,0.89,2.48,2.39,1.81,0.54,2.98,1,4.25,5.3,2.02,1.26,0.49,0.51,0.42,0.83,0.38,2.75,5.22,0.33,0.04,0.87,1.77,0.28,0.06,1.72,0.21,2.244117647,1.058666667,0.001720914,0.060514709

6793,STK10,12.37,8.71,13.46,26.21,23.22,9.84,8.9,11.04,25.7,15.8,9.02,8.11,9.7,18.68,16.56,13.46,23.1,9.96,3.81,5.67,15.61,11.89,2.27,10.15,6.84,8.39,7.5,5.31,12.19,5.66,12.03,4.39,14.93411765,8.111333333,0.001720914,0.060514709

1429,CRYZ,4.55,4.22,2.39,10.88,9.22,4.37,6.17,8.49,7.73,4.95,3.51,12.39,13,6.56,2.87,3.51,4.91,19.37,9.83,5.91,10.72,11.64,60.51,25.35,16.45,4.4,14.78,13.9,7.48,5.9,15.3,9.37,6.454117647,15.394,0.001720914,0.060514709

55466,DNAJA4,2.83,1.07,1.77,0.08,0.82,2.91,1.47,1.9,1.87,1.63,2.2,0.03,0,5.72,1.11,0.21,0.65,2.92,3.08,2.35,6.05,4.95,4.5,94.1,1.21,30.5,1.56,2.11,2.01,2.01,4.65,1.68,1.545294118,10.912,0.001720914,0.060514709

221400,TDRD6,0,0,0.01,0.03,0.02,0.15,0,0,0,0,0.19,0,0,0,0,0,0,0.01,0.23,0.09,0.04,0.02,0.09,0.34,0.13,0.02,0.06,0.01,0,0.07,0.01,0.02,0.023529412,0.076,0.001726745,0.060514709

339894,LINC00880,0.4,1.1,3.85,0.04,0,0.07,0.04,0.33,0.34,0.24,0.07,0.04,0.23,0.15,0.96,0.51,0.23,0.08,0.13,0,0,0.17,0.14,0,0,0.08,0,0.06,0,0,0.09,0,0.505882353,0.05,0.001728962,0.060514709

25834,MGAT4C,0,0,0.13,0,0.16,0.19,0,0.26,0.13,0.2,2.57,1.29,0,2.99,0.14,0.3,0.05,0,0,0,0,0,0,0,0.1,0,0,0.1,0,0.03,0.03,0,0.494705882,0.017333333,0.001753973,0.060514709

30845,EHD3,13.22,31.76,11.92,2.98,39.03,27.66,7.91,18.12,13.54,30.04,14.89,15.36,15.98,31.33,17.34,9.73,16.25,14.35,4.16,13.17,9.21,3.66,1,1.65,9,4.97,8.39,14.78,7.23,3.52,63.74,9.84,18.65058824,11.24466667,0.001760484,0.060514709

7767,ZNF224,6.96,6.06,6.15,11.75,9.12,6.41,5.39,8.72,6.28,4.92,7.69,5.02,4.79,7.22,5.93,6.07,7.11,4.18,4.89,4.96,4.63,3.17,1.07,10.74,8.99,2.59,3.14,5.34,4.03,4.42,6.53,4.57,6.799411765,4.883333333,0.001760484,0.060514709

5931,RBBP7,37.84,76.6,66.03,109.13,96.01,112.98,24.17,91.35,69.52,72.92,70.75,83.99,54.57,100.59,39.6,36.32,34.44,36.75,34.97,33.59,59,33.4,15.56,71.62,37.43,47.85,34.72,61.19,34.64,31.84,48.16,34.38,69.22411765,41.00666667,0.001760484,0.060514709

22835,ZFP30,2.11,2.18,2.24,3.72,4.2,2.91,1.88,2.81,3.02,1.87,2.89,2.51,2.62,3.46,1.71,2.54,3.23,1.68,1.32,1.85,1.11,0.92,0.5,3.52,2.34,1.06,1.24,2.16,1.69,2.26,3.32,1.84,2.7,1.787333333,0.001760484,0.060514709

54664,TMEM106B,13.78,8.13,10.91,8.43,6.48,11.32,13.32,20.06,14.14,13.82,16.29,13.94,12.79,15.18,6.74,10.84,11.47,10.01,9.15,10.1,7.15,6.73,5.2,11.84,10.41,7.64,9.23,9.89,6.23,7.76,8.49,10.58,12.21411765,8.694,0.001760484,0.060514709

56904,SH3GLB2,24.62,17.74,24.93,35.49,36.09,24.56,20.3,37.47,32.64,16.54,33.6,42.09,22.89,38.72,24.45,51.17,33.58,17.18,13.51,25.51,21.05,16.15,6.29,29.15,19.51,13.79,18.67,26.2,15.47,22.13,34.91,16.69,30.40470588,19.74733333,0.001760484,0.060514709

23089,PEG10,6.34,24.17,10.96,10.75,7.47,14.11,2.13,12.22,7.56,12.86,18.18,37.77,29.62,14.18,9.57,10.59,5.14,2.9,1.06,1.22,5.95,0.21,0.17,5.51,1.49,6.45,1.03,43.2,6.66,2.08,32.02,6.81,13.74235294,7.784,0.001760484,0.060514709

29074,MRPL18,22.23,19.34,17.66,19.24,25.03,26.57,18.56,20.03,18.35,21.29,23.43,17.76,16.4,19.6,20.61,14.54,33.62,27.42,20.8,24.99,36.25,32,56.96,27.93,20.02,26.04,23.93,18.67,33.35,25.64,20.52,28.9,20.83882353,28.228,0.001760484,0.060514709

355,FAS,0.74,0.52,1.73,2.1,0.99,6.26,7.7,2.93,0.86,2.82,6.53,3.45,10.83,5.09,1.23,0.43,0.63,10.37,3.1,17.32,2.63,7.79,2.6,1.1,15.63,9,10.08,6.64,7.86,11.89,2.91,14.12,3.225882353,8.202666667,0.001760484,0.060514709

2878,GPX3,0.87,0.14,0.61,0.31,8.38,26.35,1.02,1.17,1.49,0.38,5.44,2.47,0.62,1.33,0.45,1.03,0.52,7.89,142.37,0.81,3.21,85.54,304.25,0.29,3.13,9.83,1.79,9.99,1.98,35.64,3.18,40.83,3.092941176,43.382,0.001760484,0.060514709

4072,EPCAM,2.46,0.28,0.44,0.09,0.41,0.17,0.09,0.48,0.5,0.66,3.54,0.1,0,1.14,0.46,1.36,0.53,0.32,0.04,0,0.16,0.05,5.89,0.05,0.06,0.05,0,0.38,0,0,0.11,0,0.747647059,0.474,0.001766541,0.060615983

101055625,OVCH1-AS1,0,0,0,0,0,0,0,0,0.18,0,0,0,0,0,0,0,0,0,0,0,0.36,0,0.12,0,0.1,0.06,0,0.11,0.26,0.19,0.07,0.29,0.010588235,0.104,0.001780623,0.060991818

653604,HIST2H3D,0,0,0,0,0,0.18,0.19,3.63,4.18,0,0,0,0.44,0.84,0,0,0.45,2.88,2.64,1.56,1.21,1.3,0,1.49,3.18,0.8,0.4,0.48,0.65,0.97,1.1,1.49,0.582941176,1.343333333,0.001792128,0.061278186

127733,UBXN10,0,0.02,0,0,0.03,0,0,0,0,0,0,0,0,0,0,0,0,0.29,0.13,0,0.09,0.09,4.68,0,0,0.11,0,0.32,0,0.07,0.06,0,0.002941176,0.389333333,0.001805059,0.061612266

246329,STAC3,0.22,0.29,0.31,0.1,0.14,0.18,0.05,0.25,0.21,0.29,0.09,0.21,0.5,3.36,0.59,0.49,0.06,0.58,0.66,0.78,0.17,0.32,1.3,0.85,0.73,308.78,0.2,1.72,9.8,0.25,0.89,1.02,0.431764706,21.87,0.001831462,0.061983388

148137,PROSER3,4.35,3.04,2.82,4.48,3.25,3.26,2.4,3.85,3.25,3.32,3.12,3.59,2.59,7.29,3.83,4.02,4.81,2.85,2.01,2.68,2.19,2.5,0.72,2.71,3.5,1.52,1.86,5.29,1.57,2.32,3.32,2.86,3.721764706,2.526666667,0.001833237,0.061983388

51347,TAOK3,4.57,4.95,3.53,4.84,3.92,5.27,5.83,5.37,4.86,5.6,4.03,5.83,6.38,4.96,2.08,5.06,5.65,3.92,5.39,7.51,6.42,9.44,3.16,22.62,8.64,5.22,7.23,10.29,6.01,7.96,7.35,5.93,4.866470588,7.806,0.001833237,0.061983388

386724,AMIGO3,3.53,2.27,2.68,3.14,2.41,1.53,1.18,2.4,3.11,1.72,4.46,2.01,2.52,2.26,3.55,2.29,3.4,1.99,1.16,1.68,1.13,0.96,6.32,2.15,1.69,0.62,1.59,2.75,1.18,0.82,1.91,1.01,2.615294118,1.797333333,0.001835014,0.061983388

23405,DICER1,5.53,6.27,7.54,16.51,14.56,7.98,5.16,7.72,11.19,8.68,11.56,7.71,5.17,17.44,8.95,10.23,9.05,4.37,5.86,4.55,7.71,5.89,3.68,7.11,5.64,8.36,5.11,7.58,7.56,6.36,6.66,5.1,9.485294118,6.102666667,0.001835014,0.061983388

57646,USP28,6.63,6.03,5.54,9.12,8.75,7.15,3.16,6.86,6.49,4.74,6.24,6.07,6,5.75,7.1,6.71,4.42,2.9,3.71,4.71,7.12,3.06,2,6.58,4.6,4.45,2.99,3.55,6.03,4.82,4.7,3.22,6.28,4.296,0.001835014,0.061983388

51676,ASB2,0,0,0,0.25,0,0,0,0,0.06,0,0,0,0,0.21,0,0,0.07,0.34,0,0,0,0.06,0.1,0.74,0.25,0.47,0.03,0.07,0.43,0.02,0.52,0.16,0.034705882,0.212666667,0.001878353,0.06333755

400745,SH2D5,0.66,0.1,0.36,0.06,0.06,0.25,1.05,0.53,0.5,0.33,0.18,0.38,0.88,0.63,0.28,0.58,1.73,0.08,0.09,0.17,0.45,0.77,0,0,0.08,0.06,0.11,0,0.19,0.07,0.12,0.05,0.503529412,0.149333333,0.00194108,0.065056258

9495,AKAP5,0.98,0.38,1.44,0.86,0.69,0.6,0.25,0.74,2.34,0.75,1.15,0.38,0.25,1.04,1.14,0.58,1.89,2.71,0.35,0.15,0.32,0.2,0.16,0.39,0.34,0.19,0.33,0.35,0.43,0.33,0.71,0.25,0.909411765,0.480666667,0.00194481,0.065056258

10841,FTCD,0.5,0.06,0.43,0.62,0.71,0.3,0.46,0.57,0.9,0.35,0.6,0.54,0.48,0.67,0.6,1.01,0.58,0.04,0.25,0.17,0,0,23.61,0,0.26,0.22,0.04,0.69,0.07,0.29,0.17,0.51,0.551764706,1.754666667,0.00194481,0.065056258

54816,ZNF280D,0.21,0.24,0.08,9.91,4.33,0.34,5,7.57,6.44,0.08,0.45,0.44,0.09,7.28,0.22,0.34,0.08,4.76,8.18,8.17,7.99,4.63,4.45,5.65,9.05,4.19,5.07,7.94,5.15,6.31,10.25,6.71,2.535294118,6.566666667,0.001948544,0.065056258

55859,BEX1,28.48,0.51,0.46,0.28,5.13,0.35,7.77,23.32,37.58,5.88,12.64,8.16,8.99,1.42,2.53,1.24,24.97,9.88,0,0,0.6,3.95,0.17,0.32,17.92,0.5,0.1,0.16,0.24,0.08,1,0.32,9.982941176,2.349333333,0.001954155,0.065056258

114794,ELFN2,2.62,17.04,5.13,1.06,0.95,1.84,0.28,0.63,0.44,14.91,1.5,2.86,8.87,11.37,4.74,3,5.85,1.96,0.02,0.82,0.18,3.62,0.08,0.05,4.82,0.41,0.35,2.51,0.17,0.11,1.38,0.02,4.887647059,1.1,0.001956027,0.065056258

55425,GPALPP1,8.65,6.62,7.2,10.48,10.5,7.24,7.54,10.55,7.91,8.09,7.56,8.31,6.89,9.01,7.19,9.33,10.31,4.89,6.98,6.11,8.85,6.36,1.5,7.76,6.24,6.08,4.89,9.85,6.9,4.9,8.47,6.51,8.434117647,6.419333333,0.001956027,0.065056258

80148,PQLC1,12.29,11.71,10.36,18.1,22.34,14.73,11.87,10.52,11.25,10.52,14.82,24.3,12.82,17.19,11.76,11.89,11.59,15.21,16.88,20.84,15.86,19.92,42.26,41.57,14.24,27.86,18.21,13.44,16.51,18.13,14.81,16.55,14.00352941,20.81933333,0.001956027,0.065056258

57419,SLC24A3,0.02,0,0,0,0,0,0,0,1.68,0.02,0,0.02,0,1.04,0,0,0,3.07,0.02,0.08,0,0,0.04,0.33,0,1.15,0.08,2.39,1,0.05,4.27,5.34,0.163529412,1.188,0.001981905,0.065281062

441294,CTAGE15,0,0,0.02,0.03,0.05,0.03,0,0,0.09,0,0.3,0.16,0,0.03,0,0.03,0,0.15,0.55,0.08,0,0.14,0.87,0.68,0.1,0.16,0.46,0.09,0.08,0,0.24,0.05,0.043529412,0.243333333,0.001994642,0.065281062

119,ADD2,0,0,0,0,0,0,0,0,0,0.05,0,0.04,0.18,0,0.02,0.02,0,5.62,0.02,6.32,0.21,0.18,0,0,0.23,0.07,0,0.07,0.4,0.39,0.21,0,0.018235294,0.914666667,0.002002043,0.065281062

9048,ARTN,0.61,1.11,0.55,0,0.09,0.48,0.09,1.44,0.71,3.14,6.27,0.44,0.42,5.19,5.03,3.41,0.3,1.18,0.04,0.04,0.12,0,0,0,0.51,0,0,0.47,0.03,0.04,1.3,0.05,1.722352941,0.252,0.002008261,0.065281062

9568,GABBR2,0,0,0,0.16,0.05,0,0,0,0.03,0,0,0.03,0.14,0.02,0.09,0,0.03,2.78,0,13.78,0.73,5.49,0.1,0,0,0.19,0.31,0.2,0.1,0.28,0.07,0.07,0.032352941,1.606666667,0.002029715,0.065281062

5324,PLAG1,4.59,5.6,3.76,5.32,4.79,8,3.5,6.46,2.94,3.78,5.38,3.74,4.28,4.36,3.86,5.85,2.25,1.37,1.55,3.21,1.8,0.87,0.1,4.5,4.12,0.9,1.19,9.37,1.33,1.02,5.11,3.47,4.615294118,2.660666667,0.002040006,0.065281062

55223,TRIM62,21.78,6.82,16.19,3.23,3.01,18.64,2.49,14.55,9.56,9.91,7.03,4.21,3.26,15.98,4.92,4.76,6.91,3.85,0.88,1.08,4.07,2.27,0.22,5.86,5.45,3.88,3.38,4.38,2.67,1.6,11.99,1.59,9.014705882,3.544666667,0.002040006,0.065281062

79982,DNAJB14,8.82,6.14,6.53,10.98,10.3,7.81,9.34,9.09,7.77,8.18,8.54,6.55,6.96,8.77,6.06,6.39,9.65,4.73,5.22,5.75,8.94,7.97,2.21,9.37,5.96,5.36,6.21,6.07,5.66,6.91,7.29,4.66,8.110588235,6.154,0.002040006,0.065281062

9439,MED23,9.85,6.92,7.33,13.8,12.37,9.34,7.76,9.49,8.3,6.88,8.78,6.53,6.08,9.71,6.78,7.35,8.92,6.14,5.91,6.47,8.57,6.29,2.01,10.6,6.59,3.87,4.83,8.15,5.07,5.59,9.29,4.68,8.599411765,6.270666667,0.002040006,0.065281062

5598,MAPK7,16.48,14.7,12.6,13.78,16.71,23.39,15.58,14.59,9.65,21.14,20.9,15.79,14.24,19.3,28.9,21.36,13.96,12.62,15.67,10.5,9.82,12.21,3,13.91,13.27,5.1,9.33,38.49,12.79,10.71,15.59,13.72,17.23941176,13.11533333,0.002040006,0.065281062

9867,PJA2,37.95,49.19,37.7,38.78,38.16,43.99,41.13,43.27,28.45,58.53,48.09,46.93,46.54,44.95,42.66,44.92,42.39,34.99,52.71,29.62,28.76,25.35,36.07,41.58,35.94,34.71,33.35,44.71,28.6,30.12,41.75,37.34,43.15470588,35.70666667,0.002040006,0.065281062

7428,VHL,14.76,13.45,10.32,15.33,13.59,12.01,8.35,16.77,10.83,10.33,12.95,13.15,8.81,12.86,10.31,11.46,10.22,9.33,8.87,8.89,10.95,6.63,4.48,9.98,11.18,8.36,8.27,9.37,9.43,10.81,11.88,11.17,12.08823529,9.306666667,0.002040006,0.065281062

142940,TRUB1,6.4,4.31,5.08,7.08,6.41,5.81,5.41,7.5,5.57,4.96,6.04,4.75,5.53,6.22,4.27,4.85,7.58,5.78,4.28,4.26,3.94,3.74,3.02,5.95,4.09,6.86,4.9,5.35,3.68,4.17,4.51,4.55,5.751176471,4.605333333,0.002040006,0.065281062

125950,RAVER1,23.24,20.06,15.05,25.74,24.6,20.39,9.89,26.22,19.63,17.47,17.57,14.66,11.45,25.25,23.82,15.79,27.89,14.94,14.04,11.55,22.53,11.29,3.21,16.74,16.88,5.84,11.43,18.49,12.01,11.06,19.86,10.11,19.92470588,13.332,0.002040006,0.065281062

550643,LINC01420,42.39,85.35,55.4,65.94,38.62,55.22,22.29,39.27,33.66,36.19,62.51,41.97,35.64,61.19,37.18,32.57,37.8,36.6,38.42,22.48,38.22,17.62,2.06,44.62,31.38,29.09,26.41,33.52,23.82,26.86,40.34,31.73,46.07,29.54466667,0.002040006,0.065281062

23307,FKBP15,14.56,10.29,11.42,13.02,14.39,12.66,10.81,14.13,13.3,11.44,13.9,11.85,10.22,16.45,14.86,16.26,15.61,10.27,9.25,8.74,11.22,11.28,4.81,16.93,13.88,10.92,10.37,10.05,11.37,10.73,11.84,9.29,13.24529412,10.73,0.002040006,0.065281062

22894,DIS3,7.08,7.22,7.13,9.46,8.52,8.03,6.33,8.14,6.12,9.89,6.44,8.26,7.89,9.77,8.9,5.53,8.97,5.36,6.34,5.18,7.83,7.62,2.45,7.9,6.87,6.61,5.42,7.07,5.95,5.71,7.36,6.26,7.863529412,6.262,0.002040006,0.065281062

11196,SEC23IP,9.69,6.33,9.83,9.23,8.78,8.67,7.98,8.46,6.99,9.71,7.4,7.89,8.56,11.7,9.04,6.8,8.49,6.47,6.63,5.52,11.92,8.59,3.57,6.75,8.16,7.48,5.87,6.87,5.86,6.34,8.27,5.2,8.561764706,6.9,0.002040006,0.065281062

347853,TBX10,0.19,0.19,0,0,0,0.05,0,0.05,0.06,0.12,0.05,0,0.24,0,0.11,0.05,0,0,0,0.05,0,0,0,0,0,0,0,0,0,0,0,0,0.065294118,0.003333333,0.002043267,0.065281062

27319,BHLHE22,0,0,0,0,0,0,0,0,0,0,0,0,0,0,0,0,0,0,0,0.05,0,0,0,0,0.11,0.07,0,0.06,0.08,0.08,0.05,0,0,0.033333333,0.002066309,0.065281062

148824,GCSAML-AS1,0,0,0,0,0,0,0,0,0,0,0,0,0,0,0,0,0,0,0,0,0.07,0,0,0,0,0.04,0.04,0.03,0.17,0.14,0.03,0,0,0.034666667,0.002066309,0.065281062

84620,ST6GAL2,84,3.5,9.48,0.12,0.08,1.7,0.12,6.05,25.27,4.69,10.67,2.43,0.13,0.04,6.03,14.86,12.62,8.65,0.12,0.08,0.01,0.14,0.04,0.03,0.1,0.19,0.05,0.07,1.54,0.04,1.5,0.18,10.69352941,0.849333333,0.002068579,0.065281062

2263,FGFR2,0.24,0.25,0.23,0.31,0.22,0.05,0.17,0.43,0.38,0.05,0.15,0.44,0.34,0.22,0.22,0.05,0.13,8.66,31.72,0.69,0.23,0.14,1.67,0.53,1.2,0.18,1.05,0.84,1.6,0.51,3.06,0.11,0.228235294,3.479333333,0.002068579,0.065281062

57830,KRTAP5-8,0,0,0,0,0,0,0,0,0,0,0,0,0,0,0,0,0,0,0.06,0.2,0,0.08,0,0,0,0.07,0.07,0,0,0.11,0.16,0,0,0.05,0.002070056,0.065281062

3738,KCNA3,0,0,0,0,0,0,0,0,0,0,0,0,0,0,0,0,0,0.02,0,0,0,0,0.17,0,0,0.02,0,0.87,0.08,0.04,0,0.05,0,0.083333333,0.002070056,0.065281062

8320,EOMES,0,0,0,0,0,0,0,0,0,0,0,0,0,0,0,0,0,0,0,0,0,0,0.3,1.16,0.04,0.03,0,0.02,0.02,0.05,0,0,0,0.108,0.002070056,0.065281062

154822,LINC00689,0,0,0,0,0,0,0,0,0,0,0,0,0,0,0,0,0,0,0.1,0.27,0,0,0,0,0.02,0.75,0,0.02,0.05,0.03,0,0,0,0.082666667,0.002070056,0.065281062

5657,PRTN3,0,0,0,0,0,0,0,0,0,0,0,0,0,0,0,0,0,0,0.07,0.08,0,0,0.29,0,0,0.17,0,0.13,0.07,0,0,0.09,0,0.06,0.002070056,0.065281062

162517,FBXO39,0,0,0,0,0,0,0,0,0,0,0,0,0,0,0,0,0,0,0.12,0.19,0,0.05,0.34,0,0,0,0,0.16,0.24,0.04,0,0,0,0.076,0.002073807,0.065281062

5554,PRH1,0,0,0,0,0,0,0,0,0,0,0,0,0,0,0,0,0,0,0,0,0,0.26,0,0.2,0,0,0.35,0.15,0,0.25,0.17,0.32,0,0.113333333,0.002073807,0.065281062

100124700,HOTAIR,0,0,0,0,0,0,0,0,0,0,0,0,0,0,0,0,0,0,1.22,0,0,0,0,0.26,5.58,4.51,0.67,0,0,1.61,1.12,0,0,0.998,0.002073807,0.065281062

3227,HOXC11,0,0,0,0,0,0,0,0,0,0,0,0,0,0,0,0,0,0,1.48,0,0,0,0,1.7,2.73,3.31,0.75,0,0,1.5,0.72,0,0,0.812666667,0.002073807,0.065281062

2255,FGF10,0,0,0,0,0,0,0,0,0,0,0,0,0,0,0,0,0,0,0.35,0,0,0,0,0,1.71,0.28,0,1.9,0.11,0,0.87,1.49,0,0.447333333,0.002073807,0.065281062

387890,TMEM233,0,0,0,0,0.25,0,0,0,0,0,0,0,0,0,0,0,0,0.15,0,0,0.36,0.06,0,0,1.33,12.07,0.21,0,0.21,0.04,0.08,0,0.014705882,0.967333333,0.002076668,0.065281062

347,APOD,0.13,0.21,0.73,0.97,1.19,119.13,0.14,0,0.15,0.09,65.55,0.23,0.65,0.09,2.81,0.07,0.16,0.07,1.09,53.74,6.71,2.83,0.75,69.3,7.17,51.85,2.3,456.87,3.85,143.6,2.26,7.73,11.31176471,54.008,0.002082355,0.065354434

845,CASQ2,0,0,0,0.03,0,0,0,0,0,0.1,0.03,0,0,4.91,0,0,0,0.32,0.27,0,0,0,0,0.48,0.14,523.53,0.12,0.33,2.66,0.27,0.23,0.64,0.298235294,35.266,0.002127536,0.06659894

554236,DPY19L2P1,0.29,0.09,0.21,0.11,0,0.03,0.03,0.15,0.16,0,0.06,0,0.03,0.32,0.35,0.11,1.22,0.03,0.13,0.06,0,0,0,0,0,0,0.05,0.02,0,0,0,0.03,0.185882353,0.021333333,0.002131979,0.06659894

203328,SUSD3,0,0.15,0,0,0,0,0,0,0,0.73,0,0,0,2.74,0,0,0,0.53,1.66,0.07,0,0,1.43,0.22,0.08,0.56,0.35,0.28,1.74,2.67,0.21,0,0.212941176,0.653333333,0.00213226,0.06659894

2139,EYA2,0.2,0.02,0,0.03,0,0.58,0,0,0,0.03,0,0.06,0,0,0.55,0,0,0.06,0.22,2.94,0,0,0,0.03,6.61,2.28,3.99,4.16,2.31,1.61,30.34,0.48,0.086470588,3.668666667,0.002140529,0.066750261

128553,TSHZ2,0,0.01,0.01,0.07,0.12,0,0,0,0,0,0.02,0.03,0.02,3.02,0,0.01,0,0.65,0,7.21,0.02,0.01,1.78,0.03,0.35,0.07,0.01,0.07,4.33,6.97,0.12,0.13,0.194705882,1.45,0.002188198,0.068072028

221711,SYCP2L,0.85,0.17,0.06,0.86,0.22,0.16,0.02,0.21,0.54,0.15,0.21,0.11,0.23,0.15,0.47,0.18,0.26,0.22,0.19,0,0,0.06,0.04,0.05,0.06,0.1,0.03,0.21,0.1,0.02,0.16,0.11,0.285294118,0.09,0.002191331,0.068072028

30835,CD209,0.3,0.45,0.36,0.4,0.5,0.41,0.22,0.47,0.52,0.42,0.38,0.53,0.28,0.28,0.37,0.36,0.27,0.28,0.21,0.21,0.43,0.26,0.78,0.36,0.22,0.2,0.22,0.29,0.12,0.2,0.24,0.25,0.383529412,0.284666667,0.002193393,0.068072028

283417,DPY19L2,1.41,1.09,1.7,4.89,3.92,5.5,0.6,2.08,1.69,0.23,1.93,0.49,0.07,3.57,2.33,3.01,3.05,0.86,0.26,0,0.87,0.72,0,3.35,0.22,0.16,0.11,0.8,0.26,0.05,1.25,1.27,2.209411765,0.678666667,0.002218229,0.068255161

23119,HIC2,1.15,1.84,1.39,3.6,3.61,0.94,0.47,1.04,1.37,2.15,1.29,1.14,1.21,3.96,2.65,2.07,1.68,0.76,0.54,0.63,1.33,0.68,0.43,1.4,0.74,0.52,0.47,1.62,1.04,0.83,1.8,0.87,1.856470588,0.910666667,0.002218229,0.068255161

3778,KCNMA1,127.35,13.75,42.34,0.38,4.1,47.62,21.62,122.28,30.4,14.73,22.08,45.11,23.61,22.77,18.79,100.25,46.34,14.7,45.01,8.56,0.02,0.66,3.89,4.12,21.22,5.76,28.49,4.94,13.28,8.94,6.39,6.39,41.38352941,11.49133333,0.002220306,0.068255161

23582,CCNDBP1,9.79,3.17,5.5,8.96,7.55,7.15,13.99,10.63,7.05,4.92,5.85,3.17,2.85,7.57,5.18,4.24,8.17,8.24,5.23,12.96,10.75,9.43,8.09,29.89,8.75,13.61,10.44,8.1,9.65,15.32,7.72,11.18,6.808235294,11.29066667,0.002220306,0.068255161

7739,ZNF185,18.05,4.6,12.49,13.64,4.41,10.13,4,24.05,16.42,8.96,13.64,7.02,3.47,25.95,27.34,6.73,23.1,12.62,3.59,1.63,4.85,47.28,0.61,1.84,4.51,1.22,2.38,3.99,3.1,2.87,9.61,4.71,13.17647059,6.987333333,0.002220306,0.068255161

9201,DCLK1,0.72,0,0.19,0.43,0.27,0.34,0.08,0.43,0.5,0.13,2.29,4.6,0.06,3.39,0.25,0.39,0.46,11.04,0.01,4.7,2.12,0.76,0.07,3.02,3.7,99.77,1.7,2.5,6.14,7.86,9.17,4.58,0.854705882,10.476,0.002220306,0.068255161

57863,CADM3,0.43,0,0,0,0.17,0,0,0.09,5.68,0.34,0.06,0,0.03,0,0.1,0.2,0.46,8.96,0.04,0.66,8.03,0.2,0.23,0.34,0.08,58.37,4.39,4.06,0.62,0.28,0.23,0.17,0.444705882,5.777333333,0.00223747,0.06867446

57624,NYAP2,0.09,0.09,0.1,0.17,0.08,0.12,0.05,0.12,0.1,0.12,0.22,0.17,0.07,0.13,0.08,0.19,0.1,0.1,0.09,0.07,0,0.02,0.03,0.08,0.1,0,0.09,0.17,0.08,0.03,0.04,0.04,0.117647059,0.062666667,0.002254422,0.068778487

445582,POTEE,0,0.05,0,1.56,1.27,0,0,0,0,0,0.03,0.13,0.39,1.42,0.07,0.16,0.13,0,0,0,0.07,0,0,0,0,0,0,0,0,0,0,0,0.306470588,0.004666667,0.002257492,0.068778487

140733,MACROD2,0.27,0,0.05,0.14,0.17,0.03,0.05,0,0,0,0.54,0.04,0.14,0.07,0.06,0.02,0,0.81,0.09,0.49,0.12,0.2,0.46,0.71,0.61,0.66,0.06,0.04,0.39,0.37,1.37,0,0.092941176,0.425333333,0.00228214,0.068778487

339184,CCDC144NL,0.23,0.15,0.11,0.06,0.22,0,0,0,0,0.17,0.39,0.18,0.13,0.41,0.06,0.1,0.35,0,0.05,0,0,0,0,0.03,0,0,0,0,0.02,0.12,0.07,0.06,0.150588235,0.023333333,0.002287239,0.068778487

440905,FAR2P1,0.03,1.68,0,17.04,15.44,0,0,0.02,0,0,1.86,2.59,1.88,14.89,1.92,5.69,3.27,0,0,0.03,0.02,0,0.06,0,0,0,0,0,0,0,0.01,0,3.900588235,0.008,0.002304155,0.068778487

3207,HOXA11,0,0,0,0,0,0,6.79,0,0,0,0,0,0.2,6.46,0,0.03,0,0,18.14,6.88,2.6,1.02,0,0.1,12.73,4.22,12.26,0.02,0,2.75,4.59,0.07,0.792941176,4.358666667,0.002309054,0.068778487

26095,PTPN20,0,0,0,0,0,0,0,0,0,0,0,0.02,0,0,0,0,0,0,0.57,0.04,0,0,0,0.05,1.17,0.13,0,0,0,0.21,0.15,0.19,0.001176471,0.167333333,0.00230951,0.068778487

116154,PHACTR3,0,0,0,0.04,0,0,0,0,0,0,0,0,0,0,0,0,0,0,0,5.51,0,0,0,2.94,0.09,0.37,0,0.12,0.35,0.15,0.06,0,0.002352941,0.639333333,0.00230951,0.068778487

147660,ZNF578,0.22,0.15,0.23,0.34,0.43,0.22,0.73,0.12,0.37,0.07,0.12,0.17,0.14,0.91,0.15,0.27,0.43,0.88,0.79,0.56,0.23,1.21,0.06,0.81,0.59,0.49,0.79,0.7,0.35,0.62,0.77,0.59,0.298235294,0.629333333,0.002351267,0.068778487

631,BFSP1,2.4,3.94,1.38,6.55,4.42,4.14,0.41,5.5,5.53,3.47,1.61,2.11,0.92,4.33,1.04,3.61,2.65,1.06,0.94,0.54,3.31,2.78,0,1.85,1.07,0.79,0.36,2.23,1.05,0.49,2.15,0.93,3.177058824,1.303333333,0.002357947,0.068778487

91695,RRP7BP,5.44,4.44,5.43,10.25,10.33,3.89,1.6,3.36,3.91,7.73,4.66,4.85,6.16,11.17,5.86,5.83,6.9,4.15,7.51,2.22,2.34,2.17,2.03,3.81,3.25,2.61,2.31,10.72,2.4,1.69,4.26,2.95,5.988823529,3.628,0.002357947,0.068778487

7543,ZFX,5.51,4.8,4.59,8.99,8.4,5.63,4.39,7.08,6.05,4.15,4.43,8.17,5.34,10.01,3.41,4.81,6.65,4.27,3.89,5.61,5.58,4.93,1.76,5.23,4.66,2.58,4.54,3.09,3.15,4.6,3.29,2.47,6.024117647,3.976666667,0.002357947,0.068778487

1739,DLG1,72.15,12.98,22.17,17.51,25.02,12.48,45.64,38.53,39.22,15.93,11.99,19.09,22.4,21.77,16.88,16.18,29.79,26.02,7.47,7.99,9.41,76.61,4.47,22.95,10.83,8.78,13.6,8.01,15.03,11.74,13,9.69,25.86647059,16.37333333,0.002357947,0.068778487

89853,MVB12B,3.45,2.87,3.33,4.66,5.45,1.04,1.5,3.61,5.03,3.79,8.52,5.27,3.6,6.15,5.2,4.65,3.55,3.76,1.47,2.66,4.44,3.2,0.77,0.11,4.05,1.77,2.49,1.25,2.65,2.52,3.58,1.68,4.215882353,2.426666667,0.002357947,0.068778487

84221,SPATC1L,13.38,17.87,14.71,27.87,17.25,12.66,6.58,11.93,14.34,16.25,15.75,12.47,9.53,13.14,13.98,14.46,12.57,7.13,7.29,10.92,15.53,0,0.12,0.78,15.06,11.32,1.81,21.33,5.92,9.26,9.09,7.87,14.39647059,8.228666667,0.002357947,0.068778487

375790,AGRN,67.76,80.15,106.88,54.7,42.3,18.16,39.69,60.63,137.85,52.84,40.21,25.44,35.86,125.31,83.91,117.67,122.69,135.04,17.67,17.57,36.48,61.05,6.02,44.36,15.31,13.86,24.39,23.64,39.03,12.58,58.94,29.86,71.29705882,35.72,0.002357947,0.068778487

60559,SPCS3,26.33,23.31,27.26,40.19,33.34,27.33,49.19,28.99,31.92,37.9,33.59,37.46,54.22,55.47,23.38,24.46,38.28,27.56,17.72,22.43,32.55,28.52,29.33,20.37,23.89,26.86,34.3,24.79,16.85,17.37,23.2,26.02,34.86,24.784,0.002357947,0.068778487

54531,MIER2,13.64,13.72,10.97,13.26,12.9,11.58,15.49,12.67,8.67,15.61,10.1,9.08,10.38,12.25,14.13,8.63,18.34,10.06,8.41,6.59,10.8,14.27,2.43,6.54,12.15,8.69,8.83,10.04,10.41,5.93,12.28,6.16,12.43647059,8.906,0.002357947,0.068778487

4893,NRAS,20.2,13.38,14.74,37.28,31.5,14.29,15.63,17.1,12.76,21.04,17.74,17.26,11.16,24.81,15.2,16.47,22.28,13.46,9.35,8.37,29.02,15.6,5.24,10.84,8.1,12.97,13.42,14.49,11.71,9.67,24.97,9.52,18.99058824,13.11533333,0.002357947,0.068778487

10791,VAMP5,15.28,12.06,12.77,295.37,326.02,83.71,25.44,16.07,6.01,22.18,44.46,0.78,0,53.72,0.53,9.2,0.42,103.72,31,69.93,173.18,94.71,21.02,75.86,88.26,241.43,53.22,48.91,55.99,49.11,82.82,57.83,54.35411765,83.13266667,0.002357947,0.068778487

94,ACVRL1,0.1,8.51,1.66,114.6,119.87,0.05,1.9,1.36,0.37,0.16,0.02,0.21,0.11,2.38,0.04,0.17,0,1.03,0.93,15.26,100.26,164.12,2.86,0.23,4.5,10.73,2.21,11.22,12.82,19.57,3.54,20.08,14.79470588,24.624,0.002357947,0.068778487

10039,PARP3,6.76,3.95,3.04,2.59,3.59,5.58,5.26,7.91,6.12,6.45,10.37,5.41,9.36,9.24,6.92,2.82,4.06,4.45,16.83,18.06,4.21,9.25,3.28,14.15,23.16,7.32,11.41,10.26,6.88,14.67,9.99,14.5,5.848823529,11.228,0.002357947,0.068778487

7431,VIM,1439.49,2313.21,1163.44,1876.86,1570.48,1623,2066.65,2437.34,1521.09,1735.02,1082.67,1837.97,3721.48,1040.85,1109.43,1460.22,1847.87,2297.73,4235.84,3764.61,2680.94,3340.65,86.13,4773.66,3044.56,934.15,2185.79,4181.19,1984.55,2931.03,4061.59,3098.8,1755.71,2906.748,0.002357947,0.068778487

728489,DNLZ,10.28,7.86,9.37,6.35,8.98,15.02,3.77,6.55,8.57,7.82,5.63,9.76,8.44,8.14,8.38,7.98,13.37,11.77,8.8,12.88,15.44,17.37,6.93,25.95,16.88,9.28,9.63,8.97,10.45,15.7,9.19,9.38,8.604117647,12.57466667,0.002357947,0.068778487

54935,DUSP23,11.37,7.49,6.8,6.74,8.44,15.24,4.39,7.07,19.64,19.26,8.97,8.17,12.4,13.81,9.9,5.52,8.66,22.81,11.78,12.72,98.01,27.5,113.1,30.01,11.31,21.53,15.18,7.61,10.71,13.71,14.47,9.01,10.22764706,27.964,0.002357947,0.068778487

1992,SERPINB1,7.19,9.14,6.24,0.52,0.59,9.9,6.58,11.61,6.61,9.88,7.2,0.24,3.92,12.45,4.05,5.05,5.47,5.32,11.57,11.02,19.1,13.41,30.97,18.36,11.52,7.15,6.95,5.98,8.03,15.53,11.85,11.33,6.272941176,12.53933333,0.002357947,0.068778487

347735,SERINC2,2.23,0.47,2.54,7.47,11.47,1.11,6,5.13,6.34,2.82,31.35,1.83,5.57,33.87,2.55,1.07,3.91,8.62,2.98,12.29,7.86,27.25,60.43,19.78,19.41,114.52,22.65,9.37,13.09,8.75,24.13,1.15,7.395882353,23.48533333,0.002357947,0.068778487

23251,KIAA1024,0.24,1.2,1.06,0.94,0.64,0.84,0.44,0.73,0.51,1.84,0.74,0.62,0.88,0.73,1.03,0.53,1.09,0.71,0.59,0.44,0.21,0.29,0.1,0.23,0.57,0.38,0.22,0.76,0.71,0.37,0.93,0.48,0.827058824,0.466,0.002359998,0.068778487

148979,GLIS1,5.3,15.35,4.18,0.03,0.17,6.94,3.2,14.26,22.68,6.54,8.22,6.32,2.76,0.72,17.51,5.77,28.97,3.4,2.45,2.25,0,0.03,0.05,0,3.52,0.78,1.53,3.79,0.28,0.26,8.14,1.36,8.76,1.856,0.002362184,0.068778487

1288,COL4A6,0.97,39.96,1.72,12.82,2.13,56.64,4.42,14.6,12.07,6.94,1.62,1.79,0.26,5.65,0.93,0.27,2.61,4.41,0.03,0.01,0.98,7.08,0.19,0.05,0.26,0.12,0.05,21.98,0.04,0.02,1.42,0.69,9.729411765,2.488666667,0.002362184,0.068778487

6119,RPA3,4.07,2.96,2.09,1.65,2.4,3.75,2.4,4.14,2.72,2.86,2.63,2.95,3.64,2.94,3.01,2.84,4.52,3.53,4.14,4.34,4.69,2.04,5.63,6.63,5.2,3.99,3.17,3.39,3.48,3.49,5.58,4.93,3.033529412,4.282,0.002362184,0.068778487

1917,EEF1A2,13.23,0.21,11.28,0.31,1.94,0.18,1.05,0.86,6.83,1.6,15.01,0.34,1.85,9.16,1.39,8.29,19.11,2.64,0.13,0.12,0.38,0.04,0.14,0.17,0.34,143.44,0,0.39,0.33,0.2,0.81,0.04,5.449411765,9.944666667,0.002362184,0.068778487

284371,ZNF841,3.55,4.65,5.8,5.96,5.37,2.87,3.02,4.17,3.47,5.5,3.41,2.78,4.94,7.63,6.52,4.73,4.07,2.26,4.51,2.07,2.83,2.64,2.51,3.34,4.73,1.7,2.44,5.89,2.15,2.62,5.18,2.56,4.614117647,3.162,0.002364371,0.068778487

116115,ZNF526,4.69,5.44,4.35,5.12,5.9,5.61,4.5,4.81,4.39,4.93,5.32,4.23,5.36,6.89,6.76,4.27,7.64,3.72,3.13,3.49,4.41,3.43,2.43,7.26,4.89,3.04,3.5,4.5,4.37,3.74,5.5,3.62,5.306470588,4.068666667,0.002364371,0.068778487

26873,OPLAH,2.54,3.13,3.76,1.4,3.19,2.36,2.49,1.21,1.04,3.03,3.32,0.77,0,3.41,0.79,2.05,0.82,3.36,14.75,4.93,3.05,0.17,3.64,3.19,4.06,2.18,3.93,1.44,4.28,4.91,7.82,3.78,2.077058824,4.366,0.002364371,0.068778487

2252,FGF7,0,2.42,0,0,0.02,0.16,1.33,0.02,0.06,0.1,4.93,1.02,6.19,1.16,0,0.02,0,3.8,10.75,41.62,0.02,0.04,0.21,0,48.38,4.43,55.89,25.38,5.22,53.14,2.82,59.95,1.025294118,20.77666667,0.002416901,0.070201802

401024,FSIP2,0.55,0.09,0.12,0.6,0.51,0.38,0.27,0.01,0.17,0.14,0.65,0,0.01,0.85,0.37,0.45,0.03,0.01,0.11,0.02,0.02,0,0.05,0.07,0.01,0.02,0.02,0.29,0.03,0,0.07,0,0.305882353,0.048,0.002446201,0.070947097

114821,ZBED9,1,0.64,0.48,0.02,0,0.9,0.36,0.17,0.41,0.38,0.53,0.78,0.59,0.33,0.88,0.25,1.05,0.52,0.09,0,0,0.11,0.54,0,0.08,0.07,0.02,0.13,0.21,0.08,0.44,0.04,0.515882353,0.155333333,0.002491702,0.071823284

122970,ACOT4,0,0.07,0.11,0.14,0.09,0.17,1.02,0.49,0.81,0.23,0.09,0,0.05,0.35,0.16,0.13,0,0.65,0.16,0.9,0.5,0,36.79,0.77,0.47,0.82,1.08,0.15,1.53,1.39,0.67,0.21,0.23,3.072666667,0.002491702,0.071823284

255480,TBX5-AS1,0,0.02,0,0,0,0,0,0,0,0,0,0,0,0,0,0,0,0,1.22,11.5,0,0,0,0,0.05,0,0.02,20.57,0.09,0.89,0.03,0,0.001176471,2.291333333,0.002495579,0.071823284

255082,CASC2,0.11,0.17,0.14,0.03,0.11,0.38,0,0.27,0.11,0,0.64,0.2,0.17,0.38,0.16,0.25,0.23,0.58,0.63,0.55,0.26,0.07,0.06,0.58,0.52,0.66,0.42,0.45,0.3,0.43,0.47,0.33,0.197058824,0.420666667,0.002500864,0.071823284

9796,PHYHIP,0.31,0.22,0.06,0.47,0.1,0.04,0.07,0.63,0.05,0.2,0.05,0,0.08,0.15,0.08,0,0.03,0.46,0.93,0.32,0.66,0.32,0.04,0.13,0.36,0.15,0.13,0.42,0.25,0.6,0.35,0.93,0.149411765,0.403333333,0.002500864,0.071823284

286467,FIRRE,0.42,2.18,0.83,2.01,1.13,0.22,0.03,0.12,0.09,1.53,0.2,1.12,1.38,2.66,1.48,0.44,0.28,0.03,0.2,0.08,0.47,0.15,0,0.24,0.07,0.13,0.05,0.72,0,0.11,0.17,0.71,0.948235294,0.208666667,0.002512346,0.071823284

55297,CCDC91,3.46,1.04,1.5,3.09,3.46,3.11,8.58,6.8,5.3,1.53,3.35,3.45,6.37,4.69,0.71,1.58,3.68,7.92,3.4,6.4,3.95,3.69,6.21,10.1,7.08,6.44,7.02,3.4,6,6.61,5.89,7.54,3.629411765,6.11,0.002514646,0.071823284

51195,RAPGEFL1,2.26,3.04,2.79,3.63,4.24,3.44,2.69,2.02,1.31,10.01,1.69,2.12,1.26,1.84,3.6,3.16,1.84,2.29,1.18,1.16,1.54,0.68,0.6,0.99,2.01,0.86,0.6,7.1,1.23,0.91,4.59,0.74,2.996470588,1.765333333,0.002514646,0.071823284

8928,FOXH1,1.8,1.58,1.21,3.32,2.43,1.37,1.21,2.9,3.06,1.6,1.16,2.46,1.04,1.96,1.64,1.87,1.62,0.89,1.29,0.91,1.15,1.32,0.38,2.22,1.22,0.82,0.87,1.52,0.42,0.43,3.45,1,1.895882353,1.192666667,0.002516947,0.071823284

55319,TMA16,9.06,5.53,7.17,5.86,4.38,6.37,6.97,10,8.21,7.74,4.9,5.5,6.36,9.03,6.85,6.48,11.44,4.17,5.38,4.25,6.76,5.83,4.7,4.99,3.61,6.86,4.99,5.65,6.21,4.45,6.39,5.3,7.167647059,5.302666667,0.002516947,0.071823284

84262,PSMG3,9.11,6.47,4.81,9.36,6.93,6.23,5.97,16.12,13.09,5.48,7.22,6.73,2.9,19.45,5.48,3.94,11.39,9.92,10.04,12.69,13.93,9.2,11.63,15.56,7.96,13.18,12.45,12.68,14.05,15.48,13.33,11.26,8.275294118,12.224,0.002516947,0.071823284

26778,SNORA70,3.57,2.73,2.24,2.7,3.7,3.5,3.8,6.73,1.02,3.4,1.74,0,3.01,6.74,4.94,5.22,9.87,1.86,4.94,0,0.87,0,0,1.02,2.45,1.96,0,6.21,1.64,0,0.8,2,3.818235294,1.583333333,0.002592165,0.073861554

8092,ALX1,4.62,1.62,1.12,9.26,7.38,4.35,0.78,0,0.53,1.69,5.85,3.5,0,0,6.2,6.77,1.27,0.06,0,0,0.16,0.2,0,18.03,0.08,0.25,0,0.4,0,0.05,0,0,3.231764706,1.282,0.00261039,0.074021927

3689,ITGB2,0.15,0.04,0.02,0.05,0.03,1.16,0.05,0.06,0,0,1.17,0,0,0.33,0,0,0,1.62,0.18,4.97,0.02,0.3,25.38,0.03,1.87,5.66,16.34,0.24,0.87,0.56,0.15,0,0.18,3.879333333,0.002633104,0.074021927

3233,HOXD4,0,0,0,0,0,0,2.03,0,0,0,0,0.2,0.64,7.44,0,0,0,1.05,4.66,3.32,5.01,0,0,3.82,1.94,0.19,0.72,0.05,3.85,3.47,4.57,0,0.606470588,2.176666667,0.002638913,0.074021927

11264,PXMP4,0.35,0.39,0.4,2.65,4.67,0.16,1.53,2.97,2.55,0.32,0.31,0.39,0.3,4.82,0.35,0.47,0.39,3.16,2.55,3.94,2.25,2.48,1.21,5.28,2.94,2.51,2.88,3.99,2.61,3.32,3.12,3.23,1.354117647,3.031333333,0.00266639,0.074021927

147837,ZNF563,0.36,0.36,0.38,0.14,0.67,0.2,0.33,0.56,0.49,0.1,0.72,0,0.03,0.21,0.69,0.5,0.42,0.81,0.75,0.71,0.05,0.53,0.85,1.12,1.02,0.47,0.7,0.53,0.45,0.5,0.47,0.75,0.362352941,0.647333333,0.002668805,0.074021927

342357,ZKSCAN2,1.87,1.76,1.31,2.21,2.11,1.82,0.92,2.36,1.66,1.8,1.75,2.08,1.64,2.6,1.76,1.98,2.94,1.53,1.13,0.94,1.6,0.94,0.15,1.66,1.45,1.6,0.85,2.34,1.32,1.07,2.37,1.25,1.915882353,1.346666667,0.002671222,0.074021927

1632,ECI1,13.12,14.57,12.46,13.74,19.64,26.72,7.99,15.6,14.44,7.33,17.47,17.61,9.26,25.43,18.58,19.69,16.48,20.93,21.73,29.3,25.1,13.66,50.45,28.38,10.31,40.17,26.88,19.6,29.23,33.61,15.32,19.69,15.89,25.624,0.002678481,0.074021927

10063,COX17,14.98,11.89,12.5,18.98,15.67,18.88,33.78,11.46,15.99,23.95,13.79,13.54,23.41,20.72,15.49,9.61,23.84,51.77,26.67,14.07,39.38,38.17,73.27,59.87,21.69,88.59,30.47,13.79,21.48,17.2,18.2,26.65,17.55764706,36.08466667,0.002678481,0.074021927

127254,ERICH3,0,0.01,0,0,0,0,0,0,0,0,0,0,0,0,0,0,0,0.02,0,0,0,0,0.04,0,0.2,0,0.01,0.03,0.02,0.01,0.04,0,0.000588235,0.024666667,0.002679874,0.074021927

84417,C2orf40,0,0,0,0,0,0,0,0,0,0,0,0,0.12,0,0,0,0,6.86,107.99,0,0,0,0,0,0.13,0.11,0.33,0,1.76,0,0.51,1.04,0.007058824,7.915333333,0.00270308,0.074021927

4656,MYOG,0,0,0,0,0,0,0,0,0,0.24,0,0,0.06,11.41,0,0,0,0.5,0.09,0,0,0,0,0.06,0.25,651.87,0.16,1.21,53.03,0.17,0.41,0.06,0.688823529,47.18733333,0.002704231,0.074021927

6594,SMARCA1,33.31,109.19,64.69,27.25,23.73,57.29,34.94,31.06,27.55,88.15,32,79.56,74.32,27.13,23.61,32.59,30.25,25.76,17.79,23.23,17.71,15.08,5.04,33.21,33.54,15.59,19.34,48,25.08,21.95,42.04,27.97,46.86,24.75533333,0.002718844,0.074021927

90987,ZNF251,9.54,10.44,9.06,16.16,17.44,8.32,8.71,11.52,9.62,8.67,9.99,9.37,7.62,12.46,11.98,9.83,11.08,5.61,6.63,7.12,3.53,4.06,0.83,11.14,10.46,3.64,6.82,11.16,7.2,6,14.35,6.58,10.69470588,7.008666667,0.002718844,0.074021927

2770,GNAI1,6.74,27.61,10.06,18.7,24.17,25.77,9.88,11.42,6.55,25.25,31.2,10.04,14.16,53.06,12.26,8.61,12.21,5.99,2.29,5.98,7.22,12.87,2.48,19.31,9.6,5.48,7.67,14.3,7.3,11.29,7.03,8.68,18.09941176,8.499333333,0.002718844,0.074021927

85364,ZCCHC3,13.74,22.4,11.57,28.25,29.67,17.04,5.68,16.17,10.64,13.19,15.74,14.23,8.95,23.07,13.08,15.9,11.46,8.71,6.01,6,21.34,3.97,3.79,12.05,8.77,6.34,6.41,19.11,9.64,6.26,14.5,8.07,15.92823529,9.398,0.002718844,0.074021927

5210,PFKFB4,4.3,8.85,12.58,1.84,1.37,5.27,7.54,2.75,7.85,14.34,5.54,10.1,11.82,9.87,25.5,5.69,16.89,4.72,4.64,2.12,0.77,0.67,0.72,4,5.94,3.09,3.41,3.5,6.23,1.69,9.64,2.82,8.947058824,3.597333333,0.002718844,0.074021927

56413,LTB4R2,3.82,1.29,5.42,8.5,8.08,2.74,1.97,7.35,9.07,3.43,1.18,1.81,1.28,3.49,7.82,4.48,3.51,0.21,4.02,1.07,2.71,1.17,1.01,2.05,2.37,0.38,1.44,2.99,1.65,2.28,2.33,1.47,4.425882353,1.81,0.002718844,0.074021927

10314,LANCL1,22.33,22.37,17.3,13.93,15.22,21.56,20.61,28.75,22.77,19.74,26.5,22.5,21.85,23.07,15.98,17.05,18.81,19.29,15.06,14.9,14.52,12.62,2.5,22.59,17.16,16.59,16.74,17.52,13.07,13.73,22.29,16.72,20.60823529,15.68666667,0.002718844,0.074021927

440299,DNM1P41,4.32,3.86,5.31,3.59,2.94,1.16,1.83,3.26,2.55,3.02,6.17,1.14,1.67,4.82,5.28,4.96,3.12,2.08,2.41,1.57,1.33,0.91,0.78,2.68,2.65,0.67,1.73,2.95,2.35,2.05,3.53,1.47,3.470588235,1.944,0.002718844,0.074021927

23567,ZNF346,6.64,5.43,6.07,7.33,8.13,6.22,4.24,7.64,5.54,3.75,6.25,5.95,3.42,6.5,5,6.26,8.68,4.38,3.83,3.87,10.62,4.71,1.45,4.74,4.63,3.4,3.34,4.96,4.79,4.19,5.97,3.94,6.061764706,4.588,0.002718844,0.074021927

63893,UBE2O,11.32,8.92,6.74,11.25,11.76,8.95,5.22,10.68,6.55,9.37,7.83,7.62,6.65,8.44,7.35,8.59,6.95,5.8,6.22,4.13,8.73,7.23,3.72,18.21,6.97,5.54,5.31,6.5,5.15,4.79,8.24,4.86,8.481764706,6.76,0.002718844,0.074021927

84934,RITA1,9.68,11.24,9.58,10.49,15.46,17.39,5.49,14.05,10.08,11.91,11.59,14.17,14.02,13.8,10.41,14.26,10.93,7.11,5.85,8.05,12.71,10.32,4.07,12.7,9.41,11.34,6.36,4.43,9.45,9.06,13.71,9.13,12.03235294,8.913333333,0.002718844,0.074021927

7705,ZNF146,24.91,18.26,22.75,25.15,24.26,25.84,16.64,24,22.57,22.62,23.29,18.4,19.66,21.73,20.69,17.35,31.72,17.64,17.72,13.91,21.98,15.74,8.73,15.04,17.71,20.65,15.26,23.13,22.2,15.69,24.57,18.12,22.34352941,17.87266667,0.002718844,0.074021927

2521,FUS,35.29,17.85,38.46,49.32,46.24,39.81,17.26,40.32,32.32,29.57,35.43,32.2,21.61,66.57,34.8,40.52,68.01,18.08,19.76,29.92,37.73,18.82,6.39,27.44,31.13,26.64,18,29.75,28.03,28.52,34.38,29.41,37.97529412,25.6,0.002718844,0.074021927

23082,PPRC1,12.24,6.64,11.23,10,10.85,11.5,8.01,12.72,12.23,10.6,9.33,10.2,7.02,18.42,12.37,10.41,24.63,8.58,9.35,6.52,11.99,8.64,3.29,6.35,11.21,9.43,6.27,9.31,8.51,7.36,10.81,6.21,11.67058824,8.255333333,0.002718844,0.074021927

23479,ISCU,42.37,34.49,42.58,45.86,38.81,58.43,39.77,46.8,35.1,32.02,54.61,52.41,49.47,40.5,46.98,29.87,23.91,68.75,30.3,95.85,45.81,62.45,34.7,76.95,73.08,55.7,61.22,48.32,45.68,62.6,50.35,89.18,41.99882353,60.06266667,0.002718844,0.074021927

207063,DHRSX,5.95,4.49,5.49,7.47,7.03,9.26,3.17,4.9,5.15,5.72,8.7,6.26,4.59,8.63,4.67,4.17,5.13,6.82,6.72,7.23,6.3,8.33,9,15.65,10.44,7.74,9.04,6.73,5.58,6.84,11.25,7.02,5.928235294,8.312666667,0.002718844,0.074021927

114796,PSMG3-AS1,2.22,0.25,0.57,1.19,1.2,1.96,1.61,3.22,2.88,0.24,1.66,0.63,0.33,2.57,0.96,0.94,3.29,2.23,3.31,2.74,1.72,2.01,0.34,7.19,6.05,2.16,3.38,1.84,2.79,4.59,2.34,4.28,1.512941176,3.131333333,0.002718844,0.074021927

54543,TOMM7,78.44,99.29,101.07,67.09,61.53,151.57,80.39,104.68,68.42,142.39,95.68,123.84,127.1,81.07,98.22,67.67,76.56,154.28,122.72,127.66,118.11,100.16,64.57,248.85,128.27,119,141.49,190.13,108.82,140.41,108.4,221.13,95.58882353,139.6,0.002718844,0.074021927

140885,SIRPA,14.91,5.41,5.13,20.92,12.41,5.12,21.41,23.39,12.68,9.45,6.7,30.03,28.52,10.55,10.82,2.45,15.45,16.52,96.79,58.97,31.1,39.11,27.21,112.97,16.76,17.6,39.75,2.9,28.12,34.38,11.29,14.96,13.84411765,36.562,0.002718844,0.074021927

81558,FAM117A,4.51,1.33,1.4,1.72,3.38,1.76,0.88,2.07,1.53,1.83,2.17,1.11,2.06,2.05,1.21,1.99,0.53,2.89,1.35,3.37,13.52,3.05,3,4.4,4.46,2.59,1.77,3.03,1.74,2.42,2.14,3.19,1.854705882,3.528,0.002718844,0.074021927

152195,NUDT16P1,0.06,0.13,0.11,0.1,0,0,0.31,0,0,0.08,0.07,0.04,0,2.51,0,0.03,0,0.8,3.87,0.28,1.33,0,3.27,0,0.17,0.21,0.15,0.14,0.22,0.45,0.55,0.08,0.202352941,0.768,0.002719328,0.074021927

1747,DLX3,0,0,0,0,0,0,0,0,0,0,0,0,0,0,0.03,0,0.03,0,2.98,0.88,0,0,0,0.03,0.56,0.47,2.72,0,0.03,1.41,0,0.03,0.003529412,0.607333333,0.002752571,0.074822337

158160,HSD17B7P2,0.92,0.45,0,0,0,0.26,0,0,0,0,0.66,0,0,0,0.85,0.76,0.7,0.78,0.86,0.46,0.21,0.13,5.67,2.68,0.71,0.54,0.32,0.8,1.58,0.64,2.53,0.34,0.270588235,1.216666667,0.002780025,0.075463341

2203,FBP1,0.13,0,0,0,0,0,0,0.22,0.05,0,0,0,0,0.26,0,0,0,0.1,0,0.47,3.24,0.32,79.01,0.06,0.18,0,0.36,0.6,0.04,3.62,0,0,0.038823529,5.866666667,0.002796482,0.075804508

100132987,LINC00856,0,0,0,0,0,0,0,0.03,0,0,0,0,0.03,0,0,0,0,0.06,0.12,0.59,0,0,0,0,0.62,0.09,0.06,0,0.02,0.19,0,0.03,0.003529412,0.118666667,0.00280487,0.07592628

5629,PROX1,0.04,0.01,0.02,0.06,0.01,0.07,0.08,0,0,0,0.05,0.01,0.01,0,0,0.02,0.02,0.13,0,0.04,0.32,0.02,10.52,0.3,0.15,1.41,0.03,0.02,0.12,0.03,0.09,0.02,0.023529412,0.88,0.002818163,0.076084757

119437,CTAGE7P,0.28,0.11,0.24,0.27,0.28,0.1,0.44,0.46,0.15,0.23,0.42,0.09,0.13,0.45,0.31,0.33,0.29,0.14,0.4,0.03,0.02,0.03,0,0.3,0.24,0.03,0.06,0.21,0.09,0.12,0.2,0.03,0.269411765,0.126666667,0.002818981,0.076084757

5322,PLA2G5,0,0,0.17,0,0,0,0,0,0,0,0,0,0.05,0,0,0,0,1.03,0,0.38,0.11,0.83,0,0,1.87,0.04,1.14,0,0,3.95,0,1.43,0.012941176,0.718666667,0.002822436,0.076084757

254065,BRWD3,2.79,2.05,1.74,2.91,3.01,1.62,1.11,1.84,1.83,2.66,2.5,1.32,1.23,1.74,2.05,3.29,2.49,0.87,1.55,0.83,2.25,0.51,0.58,2.65,1.23,1.07,1.11,2.58,1.26,1.17,1.78,1.02,2.128235294,1.364,0.002841805,0.076180446

100131827,ZNF717,0.45,0.39,0.49,0.03,0.05,0.37,0.2,0.64,0.69,0.52,0.44,0,0,0.06,0.42,0.83,0.44,0.53,1.09,0.64,0.63,0.57,0.18,1.07,1.37,0.55,0.51,0.76,0.43,0.54,0.84,0.45,0.354117647,0.677333333,0.002841805,0.076180446

653188,GUSBP3,0.37,1.26,0.64,0.79,1.19,1.33,0.25,1.03,0.85,1.06,1.17,0.1,0.79,1.05,1.56,1.98,1.19,0.17,0.24,0.47,0.14,0.26,0,1.62,0.59,0.78,0.3,0.72,0.11,0.8,0.76,0,0.977058824,0.464,0.002844348,0.076180446

146540,ZNF785,4.44,5.06,3.71,5.46,5.73,3.45,1.78,5.08,4.57,4.01,3.9,2.99,3.45,4.14,5.45,4.68,4.85,3.06,3.95,2.44,3.95,1.37,1.31,4.58,3.83,1.26,2.1,4.51,2.43,2.53,3.74,2.62,4.279411765,2.912,0.002846892,0.076180446

56981,PRDM11,1.42,2.84,1.89,3.33,3.85,3.06,0.51,1.88,2.07,0.89,2.41,0.83,1.27,4.38,1.5,1.22,2.16,0.79,0.29,1.03,1.55,0.94,0.08,1.23,0.77,0.73,0.58,4.62,0.64,1.04,0.96,1.03,2.088823529,1.085333333,0.002849438,0.076180446

54503,ZDHHC13,7.57,10.63,5.06,10.16,8.36,7.87,5.06,6.86,4.64,3.99,4.35,7.85,6.6,7.09,4.87,5.97,6.27,3.48,2.5,2.18,11.86,21.75,0.55,6.05,4.99,1.3,2.74,1.03,2.52,2.3,4.72,3.57,6.658823529,4.769333333,0.002849438,0.076180446

574029,DUSP5P1,1.47,0.72,0.25,0,0,0.52,1.1,0.97,0.62,2.26,0.79,1.04,0.4,0.16,1.45,0.41,0.04,0.03,0.68,0.51,0,0.43,0,0.04,0,0.13,0,0.03,0.08,0.24,0.18,0,0.717647059,0.156666667,0.002880051,0.076683317

55630,SLC39A4,0,0,0.03,1.45,3.15,0,0.82,2.49,1.28,0,1.56,0.04,0.04,4.3,0,0,0,3.54,2.26,1.24,2.27,0.68,1.14,1.78,3.24,1.19,2.24,18.97,1.72,2.14,4.98,1.56,0.891764706,3.263333333,0.002880051,0.076683317

8630,HSD17B6,0,0,0,0,0.84,0.14,0.21,0.17,0.23,0.13,0.7,0.17,0.24,0.07,0.12,0,0.31,2.89,0.27,0.85,0,0.59,75.5,1.4,0.53,1.42,5.01,0.13,3.5,3.64,0.3,0,0.195882353,6.402,0.002880051,0.076683317

108,ADCY2,0,0,0,0,0,0,0.01,0,0,0,0,0,0.03,0,0.01,0,0,2.23,1.8,0,0.02,0.01,0,0.53,0,2.95,0,0.01,0.26,0.01,0.47,0,0.002941176,0.552666667,0.002892157,0.076900611

84847,LINC00525,0.19,0,0.11,0.07,0.4,0.12,0,0.07,0.14,0.08,0,0.07,0.08,0.25,0.07,0.06,0.08,0,0,0.07,0,0,0,0,0,0.21,0.14,0,0,0,0,0,0.105294118,0.028,0.002906345,0.077100662

1160,CKMT2,0,0,0,0,0,0,0,0.06,0,0,0.05,0,0.17,0.13,0,0,0,0.27,0.74,0,0,0,0,0.24,0.63,31.55,0,0.84,0.09,0.14,0.3,4.71,0.024117647,2.634,0.002907593,0.077100662

338321,NLRP9,0.02,0,0.02,0.07,0,0,0.02,0.02,0,0,0,0.02,0.03,0,0,0.02,0,0,0,0,0,0,0,0,0,0,0,0,0,0,0,0,0.012941176,0,0.002970423,0.078659702

339524,LINC01140,0.07,1.61,0.15,0.19,0.1,0.23,0,0.05,0,0.29,0.24,0,0.09,0.39,0.23,0.1,0,0.31,3.44,0.11,0.22,0.13,0.6,0.08,3.34,0.39,0.1,2.63,0.56,6.45,0.57,2.04,0.22,1.398,0.002990316,0.079042023

144402,CPNE8,0.29,0,0.02,2.54,3.04,0.02,0.84,0,0,0,2.01,0.02,0.99,0.61,0.05,0.06,2.49,1.23,1.96,0.79,3.72,2.1,6.66,12.31,2.92,0.58,1.26,0.06,1.65,2.14,1.11,3.27,0.763529412,2.784,0.002992971,0.079042023

100130275,TFAP2A-AS1,1.13,0.28,1.42,0,0,1,0.24,1.22,1.06,0,0.87,0.66,0.07,0.08,0.27,0.11,0.64,0.06,0.1,0.12,0.05,0,0,13.22,0,0,0.06,0,0,0,0,0,0.532352941,0.907333333,0.002997775,0.079061764

7020,TFAP2A,15.3,3.61,24.17,0,0.55,22.24,4.72,17.44,14.24,0.28,11.99,8.76,0.26,0.41,3.12,0.11,8.67,0.31,0.24,1.11,0.15,0.08,0,230.58,0.05,0.15,0.29,0.02,0.04,0.11,0.28,0.21,7.992352941,15.57466667,0.003022279,0.079169687

79183,TTPAL,5.47,4.31,4.67,2.9,2.01,9.78,6.21,5.55,6.04,4.32,8.79,6.9,6.21,5.77,5.43,4.66,12.47,4.99,3.12,4.17,2.77,3.49,3.35,4.22,5.4,4.71,3.6,3.6,4.27,4.67,5.21,3.03,5.97,4.04,0.003024952,0.079169687

653268,AGAP7P,2.37,1.46,1.85,0.51,0.31,2.06,0.41,0.71,0.84,1.68,1.57,0.59,0.37,0.89,2.25,0.95,2.39,0,0.4,0.4,1.27,0.47,0.28,0.7,0.23,0.59,0.19,0.66,0.48,0.83,0.96,0.13,1.247647059,0.506,0.003027626,0.079169687

6452,SH3BP2,8.1,20.59,11.98,32.21,35.91,14.2,3.98,11.82,15.02,21.61,18.75,6.56,6.46,16,20.85,13.68,10.17,4.6,10.78,4.52,10.23,6.46,4.75,10.12,11.76,3.52,4.06,19.89,5.38,3.28,12.12,9.06,15.75823529,8.035333333,0.003030302,0.079169687

54790,TET2,3.35,2.79,3.36,4.61,4.37,3,2.18,4.22,4.23,2.35,3.7,1.93,1.67,3.65,4.03,2.74,3.85,2.56,1.78,1.19,3.15,1.38,0.75,2.16,2.39,3,1.9,3.18,1.99,1.3,4.13,1.84,3.295882353,2.18,0.003030302,0.079169687

1849,DUSP7,13.56,17.39,10.3,14.83,13.94,14.73,7.95,16.96,11.36,9.66,12.25,8.95,12.56,14.45,12.82,24.76,17.66,5.02,7.58,8.78,12.71,7.85,1.29,10.94,7.95,5.55,8.85,29.02,9.39,6.95,19.87,6.39,13.77235294,9.876,0.003030302,0.079169687

51103,NDUFAF1,2.91,3.33,3.43,8.03,6.92,4.79,7.29,8.07,8.28,3.82,6.12,6.6,6.39,7.2,3.7,2.81,4.44,10.23,7.62,5.49,8.96,8.82,12.22,13.73,7.71,16.55,6.39,6.58,8.18,5.5,10.34,5.72,5.537058824,8.936,0.003030302,0.079169687

282808,RAB40AL,0,0,0,0.54,0,0,0.1,0.1,0.09,0,0.21,0.11,0.1,0,0.1,0,0,0,0,0,0,0,0,0,0,0,0,0,0,0,0,0,0.079411765,0,0.003086842,0.07921071

145216,LINC00637,0.07,0.06,0.06,0.15,0.27,0.07,0,0,0,0,0,0,0,0,0.09,0.04,0,0,0,0,0,0,0,0,0,0,0,0,0,0,0,0,0.047647059,0,0.003124665,0.07921071

767846,PFN1P2,6.39,3.55,5.76,13.65,15.34,3.52,7.01,6.11,6.03,6.59,2.92,2.61,5.73,9.85,8.21,8.36,10.36,1.77,5.13,2.51,2.76,4.06,2.14,6.29,8.19,2.18,3.18,5.03,3.16,4.37,6.82,2.89,7.175882353,4.032,0.003127423,0.07921071

6608,SMO,17.9,22.63,21.05,4.38,4.77,12.76,6.01,21.58,16.9,12.9,33.23,21.15,10.89,23.1,14.84,14.79,12.27,18.71,10.29,4.16,8.21,1.04,2.1,16,10.05,2.09,7.98,9.93,10.62,5.71,19.57,2.55,15.95,8.600666667,0.003127423,0.07921071

29775,CARD10,18.59,2.5,3.88,36.21,51.07,7.16,8.69,19.44,15.58,7.37,3.15,17.98,11.99,21.62,4.51,8.76,22.86,9.01,1.17,2.17,18.37,13.41,4.77,0.39,8.37,1.46,3.32,3.6,3.42,3.95,5.39,1.75,15.37411765,5.37,0.003127423,0.07921071

79684,MSANTD2,4.63,3.53,3.64,7.93,7.79,4.24,1.84,6.3,4.07,3.5,4.6,3.95,3.67,6.9,3.76,4.41,4.66,2.75,2.27,2.76,4.19,2.46,1,4.08,3.42,1.77,2.18,4.89,2.87,2.65,5.26,2.91,4.671764706,3.030666667,0.003127423,0.07921071

373863,DND1,5.65,3.52,3.97,2.55,4.51,5.39,2.44,5.6,4.54,3.42,4.46,3.34,2.78,2.93,4.14,3.95,5.59,1.51,3.15,3.11,2.27,1.64,2.11,6.93,2.61,3.48,2.34,1.88,2.38,2.17,3.37,4.72,4.045882353,2.911333333,0.003127423,0.07921071

64764,CREB3L2,42.28,44.57,18.14,14.47,17.05,26.97,21.23,72.54,43.42,31.72,24.61,26.1,19.14,46.53,32.14,30.9,70.15,16.07,38.32,18.66,18.91,12.06,5.81,18.39,19.38,13.26,19.53,26.75,16.21,15.58,27.09,23.1,34.23294118,19.27466667,0.003127423,0.07921071

26259,FBXW8,8.26,6.63,8.35,8.27,8.76,6.66,5.78,7.58,6.85,8,5.61,6.3,6.67,9.38,11.02,7.46,9.33,4.63,6.07,3.45,7.84,4.67,1.05,8.83,5.82,5.22,5.72,6.34,7.11,5.19,8.55,4.41,7.700588235,5.66,0.003127423,0.07921071

10009,ZBTB33,5.48,9.03,8.84,10.3,9.38,13.13,3.53,4.84,4.41,11.3,6.47,9.28,8.54,6.31,3.56,4.26,6.03,4.53,3.54,2.83,7.1,4.47,2.15,9.88,4.61,3.19,2.99,5.29,3.34,3.68,5.55,3.58,7.334705882,4.448666667,0.003127423,0.07921071

58517,RBM25,22.6,15.91,26.79,33.35,28.72,18.34,15.39,23.92,21.41,23.42,20.01,15.62,14.92,34.44,21.5,30.2,27.3,13.21,21.3,13.58,22.3,18.35,5.94,18.47,21.36,15.94,12.11,20.26,12.43,10.25,26.38,10.56,23.16705882,16.16266667,0.003127423,0.07921071

8829,NRP1,101.75,59.74,72.33,76.89,94.58,101.51,21.3,217.11,33.86,88.62,120.03,114.15,113.1,58.07,66.82,81.65,49.22,16.31,20.16,38.36,61.37,50.43,9.74,0.2,111.3,25.6,23.46,49.82,31.88,51.71,54.09,132.55,86.51352941,45.132,0.003127423,0.07921071

2110,ETFDH,7.65,6.29,5.52,5.3,5.99,9.21,11.18,10.21,6.32,7.15,8.59,5.93,4.64,5.9,5.54,7.45,6.67,7.06,8.5,11.45,12.63,7.02,16.21,9.22,10.11,9.46,7.38,8.78,5.62,9.44,8.63,12.01,7.031764706,9.568,0.003127423,0.07921071

83548,COG3,7.97,8.15,7.91,8.39,9.2,8.81,7.15,9.72,7.04,8.45,7.6,8.05,8.11,10.15,9.04,7.79,10.63,6.35,6.24,6.66,6.68,5.77,11.59,9.23,6.72,6.56,5.95,8.19,6.82,6.42,8.72,5.84,8.48,7.182666667,0.003127423,0.07921071

4436,MSH2,10.05,8.01,7.82,14.37,15.03,13.08,5.25,9.51,9.4,11.62,10.99,7.58,8.2,11.07,11.05,11.29,18,5.9,4.26,6.93,14.56,5.07,4.59,11.2,6.18,8.3,5.42,5.12,7.14,5.57,12.82,5.92,10.72470588,7.265333333,0.003127423,0.07921071

23534,TNPO3,16.4,10.45,12.7,18.65,17.9,15.22,12.07,14.99,15.6,15.37,17.54,12.48,11.09,15.18,12.31,13.8,17.96,11.79,7,9.29,17.38,14.26,5.85,10.91,10.89,12.51,11.07,14.77,11.65,9.37,15.63,8.11,14.68882353,11.36533333,0.003127423,0.07921071

8409,UXT,38.61,54.7,41.5,54.37,44.06,57.9,40.65,52.28,33.3,57.01,32.69,56.94,53.75,57.26,54.39,50.94,43.8,38.13,64.64,60.61,58.64,46.04,56.03,77.87,52.52,72.63,51.73,76.58,56.28,64.54,57.45,86.24,48.47941176,61.32866667,0.003127423,0.07921071

347733,TUBB2B,36.43,10.06,21.74,30.56,8.74,8.99,2.59,13.19,32.44,11.37,26.75,2.49,2.42,79.69,20.55,11.84,46.69,92.87,58.83,2.35,1.28,0.07,0.91,4.26,2,4.78,2.44,3.78,9.6,1.12,8.62,0.74,21.56117647,12.91,0.003127423,0.07921071

11315,PARK7,107.4,82.2,105.55,94.78,95.32,142.53,88.69,134.41,102.41,98.13,104.74,114.49,98.84,119.13,88.54,77.33,129.61,129.98,113.81,110.13,152.57,119.37,149.33,134.98,107.25,207.92,105.84,103.23,117.08,108.84,130.62,118.56,104.9470588,127.3006667,0.003127423,0.07921071

147807,ZNF524,5.62,9.35,4.45,12.6,10.91,10.21,3.04,8.37,4.85,6.11,5.7,6.95,10.58,5.91,6.39,5.03,3.47,5.18,7.63,17.46,13.83,11.47,6.6,15.32,10.39,7.98,9.69,11.74,7.91,17.14,8.53,14.14,7.031764706,11.00066667,0.003127423,0.07921071

56655,POLE4,13.26,7.13,12.17,7.86,16.27,13.39,10.06,28.26,16.95,11.24,5.6,71.23,37.51,21.55,8.33,6.57,22.88,29.07,17.9,20.27,20.83,21.38,46.25,34.27,26.31,38.94,24.41,15.12,17.3,24.96,28.06,40.78,18.25058824,27.05666667,0.003127423,0.07921071

654433,PAX8-AS1,0.29,0.07,0.05,13.08,13.01,0.17,2.09,1.29,1.16,1.66,0.08,1.87,0.54,18.33,0.3,0.4,1.11,5.29,12.65,18.61,1,4.25,3.49,5.01,36.73,25.71,6.45,0.06,14.39,12.23,18.27,8.68,3.264705882,11.52133333,0.003127423,0.07921071

976,ADGRE5,2.52,2.11,0.87,1.24,3.16,2.13,17.64,23.18,7.09,4.79,11.31,3.39,4.36,16.22,0.48,0.2,0.47,10.95,3.18,41.58,3.95,2.32,4.77,16.15,13.45,25.17,52.93,16.47,16,19.41,10.92,23.69,5.950588235,17.396,0.003127423,0.07921071

1007,CDH9,0,0.81,0.04,0,0,0.02,0,0,0,0,3.44,0,0.21,0.09,0.06,0.09,0,0,0,0,0,0,0,0,0,0,0,0,0,0,0,0,0.28,0,0.003129412,0.07921071

401138,AMTN,0.56,0,1.7,0,0,0.07,0,0.08,0,0,0.14,0,0,0,2.6,29.02,2.02,0,0,0,0,0,0,0,0,0,0,0,0,0,0,0,2.128823529,0,0.003134164,0.079228086

64284,RAB17,0.08,0.06,0.07,0.29,0,0.07,0.04,0,0.09,0,0,0.32,0.05,0,0,0.04,0,0.39,0.07,0.3,0.03,0,10.34,0.47,1.32,0.65,0.21,0.04,0.25,0.36,0.07,0.24,0.065294118,0.982666667,0.003144411,0.079384159

79887,PLBD1,0.23,0.03,0.88,0,0.12,0.07,0.04,0.17,0.09,0.1,8.08,0,0,0.96,0.04,0.19,0.14,1.17,5.82,0.21,0.72,0,8.61,0.04,1.63,0.97,0.17,1.24,1.63,3.87,2.43,1.67,0.655294118,2.012,0.003179565,0.080167804

23046,KIF21B,0.1,2.81,0.66,0.87,1.61,0.28,0.05,0.29,0.6,3.95,0,0.8,0.96,1.04,1.61,0.56,0.51,0.13,0.27,0,0.23,0.56,0.43,0.07,0.07,0.1,0.01,0.29,0.15,0.04,0.7,0.11,0.982352941,0.210666667,0.003207538,0.080395119

6819,SULT1C2,0.03,0.36,0.31,0.03,0.06,0.25,0,0.12,0.03,1.05,0.08,1.61,2.13,0.03,0.47,0.52,0.03,0,0.02,0,0,0,0,0.43,0,0,0,0.14,0.05,0.02,0.16,0.09,0.418235294,0.060666667,0.003218623,0.080395119

256051,ZNF549,2.19,1.53,2.15,3.26,4.38,2.08,1.68,2.65,2.37,1.51,2.65,2.05,1.57,2.17,2.04,2.56,3.03,1.8,1.45,1.23,1.36,1.34,0.14,4.04,1.91,1.36,1.1,1.71,1.65,1.49,2.96,1.76,2.345294118,1.686666667,0.003218768,0.080395119

27120,DKKL1,0.39,0.89,0.44,0.47,0.16,0.57,0.25,0.18,0.45,1.23,1.05,0.43,0.88,0.97,0.96,0.73,0.59,0.17,0.21,0.26,0.23,0,0.15,0.53,0.11,0.33,0.08,1.43,0.34,0,0.55,0.19,0.625882353,0.305333333,0.00322158,0.080395119

29904,EEF2K,5.58,7.71,8.39,25.22,22.26,7.48,5.54,9.21,7.07,9.6,8.74,11.91,9.55,5.32,8.92,8.11,7.51,4.96,5.06,4.94,12.71,6.06,3.67,6.09,7.13,4.96,4.85,8.94,5.62,6.54,7.43,5.78,9.889411765,6.316,0.00322158,0.080395119

374659,HDDC3,5.1,3.47,3.54,5.99,7.81,6.44,3.26,7.2,3.79,3.41,4.8,3.14,5.88,6.52,3.04,3.9,4.25,4.48,6.99,4.87,9.99,8.53,6.12,8.91,6.91,5.78,6.91,6.88,4.11,5.77,7.42,7.87,4.796470588,6.769333333,0.00322158,0.080395119

115207,KCTD12,1.17,0.06,0.32,46.92,85.5,3.86,1.23,0.43,0.15,0.49,29.13,14.1,5.41,0.26,6.33,0.41,1.17,6.51,2.67,16.29,95.57,110.71,6.2,5.92,28.3,35.1,7.93,17.4,15.94,18.51,17.38,11.87,11.58470588,26.42,0.00322158,0.080395119

4094,MAF,0.96,0.63,3.92,0.07,0.32,0.3,0.38,4.06,1.18,4.99,5.03,0.1,0.07,0.47,0.78,2.62,1.61,1.56,11.38,5.09,0.04,0.65,5,24.19,5.06,1.02,3,7.17,2.05,7.79,21.97,7.38,1.617058824,6.89,0.00322158,0.080395119

9022,CLIC3,1.61,1.93,0.73,0.1,0,0,2.52,1.17,0.33,2.83,0.1,0,0,0.38,0.22,0,0.24,8,8.03,11.53,0.36,17.87,0.36,0,0.77,0.74,4.92,0.84,4.72,14.67,5.74,0.45,0.715294118,5.266666667,0.003318457,0.08270682

5348,FXYD1,0.29,1.62,0.86,0,0,0,0.16,0.97,0,0.56,0.15,7.77,12.98,0.19,3.12,1,0,0.92,16.28,10.42,0,0.35,7.52,0.51,6.81,21.31,1.29,44.39,4.68,23.9,5.61,76.25,1.745294118,14.68266667,0.003324258,0.08274559

3764,KCNJ8,1.12,0,0,0,0.13,0.12,0.58,0.03,0,0.04,0.12,0.11,1.74,0,0,0,0.04,0.39,0.14,6.94,0,0,21.71,0,6.63,1.32,0.79,4.46,2.52,4.54,1.23,8.2,0.237058824,3.924666667,0.003345129,0.08315888

4625,MYH7,0,0,0,0,0,0,0,0,0,0.23,0,0,0,4.59,0,0,0,0.34,0,0,0,0,0,0.25,0.4,684.82,0.14,0.6,0.24,0.28,0.19,0.1,0.283529412,45.824,0.00337696,0.083769865

100289137,FAM95C,0.19,0.2,0.45,0.32,0.26,2.31,0.06,0.09,0.16,0.21,0.06,0.29,0.07,2.01,0.59,0.35,1.91,0.03,0,0.03,0.31,0,0.37,0.03,0.15,0.55,0.03,0.1,0.05,0,0.14,0.07,0.560588235,0.124,0.003379669,0.083769865

9098,USP6,0.38,0.13,0.32,0.26,0.11,0.11,0.22,0.48,0.32,0.26,0.3,0.17,0.26,0.3,0.23,0.24,0.15,0.12,0.52,0.13,0.05,0.09,0.07,0.18,0.14,0.34,0.1,0.1,0.1,0.07,0.18,0.09,0.249411765,0.152,0.003382601,0.083769865

114783,LMTK3,1.5,0.17,0.46,0.25,0.09,0.14,0.31,1.13,0.47,0.11,2.53,0.68,0.52,0.58,0.19,0.48,0.09,0.81,0.19,0.19,0.06,0.07,0.06,0.53,0.35,0.03,0.05,0.01,0.07,0.08,0.09,0.14,0.570588235,0.182,0.003394341,0.083953946

100134869,UBE2Q2P2,0.61,0.49,1.33,1.51,0.97,0.22,0.41,1.12,0.46,0.75,0.41,0.11,0.76,0.49,0.9,0.84,0.59,0.33,0.11,0.2,0.39,0.34,0,1.1,0.61,0.08,0.34,0.64,0.46,0.21,0.34,0.17,0.704117647,0.354666667,0.003400221,0.083992547

340578,DCAF12L2,0,0,0,0,0,0,0,0,0,0,0.06,0,0,0,0,0,0,0.39,0.08,0.03,0.53,0,0,0,0,0.06,0,0,0.43,0.2,0.05,0,0.003529412,0.118,0.003404521,0.083992547

122525,C14orf28,1.22,0.8,1.27,1.03,1.77,1.12,2.96,1.38,1.92,0.49,0.95,1.52,1.46,1.67,1.23,1.67,1.78,2.04,2.03,3.49,0.6,1.83,3.32,3,2.39,1.28,2.36,1.09,2.03,1.88,1.79,2.96,1.425882353,2.139333333,0.003417895,0.084148587

3658,IREB2,12.62,11.99,11.06,16.85,16.87,12.96,8.82,12.86,9.69,12.72,14.09,10.38,7.48,20.09,11.23,10.9,14.59,8.96,7.7,7.89,11.37,7.61,4.01,9.99,8.15,13.21,7.87,12.86,9.92,7.85,10.96,7.71,12.65882353,9.070666667,0.003423798,0.084148587

56985,ADPRM,4.72,4.11,2.4,4.9,3.92,3.05,4.38,3.47,4.65,4.76,1.45,2.85,4.91,5.28,3.32,3.07,3.35,3.92,4.95,5.37,4.3,3.58,2.53,6.83,5.61,7.46,4.16,5.22,6.62,7.51,5.1,6.3,3.799411765,5.297333333,0.003423798,0.084148587

100506311,HOTAIRM1,0,0,0,0.1,0.15,0,0.74,6.46,0,4.07,0,0,6.83,8.98,0,0,0,1.16,1.49,2.41,7.19,6.23,0.38,4.86,3.59,5.85,2.44,7.64,1.81,1.15,7.41,0.12,1.607647059,3.582,0.003564013,0.086668732

114803,MYSM1,6.6,4.99,6.38,18.02,14.46,5.85,4.66,5.66,5.45,7.06,4.85,4.51,3.87,10.46,9.1,7.17,8.14,2.86,5.82,3.13,4.43,1.21,1.41,9.13,6.37,3.02,3.22,6.87,3.36,3.15,7.91,3.33,7.484117647,4.348,0.003589027,0.086668732

143279,HECTD2,5.66,3.9,6.97,3.51,3.82,3.01,2.23,8.57,8.15,3.65,8.48,3.99,4.43,3.62,3.87,4.54,8.67,7.27,3.4,4.74,2.14,2.11,0.32,0.64,3.04,2.43,2.48,6.3,3.52,2.5,3.67,2.71,5.121764706,3.151333333,0.003589027,0.086668732

284565,NBPF15,18.35,15.95,16.54,38.67,39.29,19.5,19.93,24.18,22.09,18.16,16.82,15.75,22.51,28.97,15.7,20.31,25.23,10.89,8.21,13.38,12.42,17.36,9.79,27.14,17.63,11.24,9.8,17.77,16.71,14.53,23.95,19.28,22.23235294,15.34,0.003589027,0.086668732

7091,TLE4,15.26,6.81,18.44,2.97,6.75,9.23,10.85,10.87,22.05,2.01,10.9,7.23,4.46,13.26,11.57,11.25,21.22,18.36,1.77,4.07,7.49,3.86,1.34,2.56,4.48,7.79,6.28,3.09,5.35,1.37,10.54,4.78,10.89,5.542,0.003589027,0.086668732

51495,HACD3,37.96,26.99,33.49,43.87,49.11,37.57,26.39,37.9,33.07,42.75,52.54,55.36,42.33,40.32,25.81,40.31,36.18,23.75,20.57,28.51,39.04,33.26,20.2,89.68,29.98,13.88,19.75,26.61,23.56,22.74,46.95,21.37,38.93823529,30.65666667,0.003589027,0.086668732

140901,STK35,4.98,8.26,5.43,6.46,7.76,5.26,3.68,6.32,4.25,7.24,5.95,6.44,11.13,7.46,6.73,7.28,4.66,4.46,4.36,4.31,5.83,4.42,1.96,5.3,5.7,2.71,4.55,8.28,3.71,3.75,5.4,4.61,6.428823529,4.623333333,0.003589027,0.086668732

29766,TMOD3,20.82,15.95,20.26,49.22,60.77,27.91,20.86,43.52,24,23.06,19.03,22.57,23.18,19.32,17.82,24.4,29.98,12.45,9.65,15.15,27.88,22.32,5.84,11.22,13.69,6.41,12.43,27.4,16.66,18.66,33.03,19.6,27.21588235,16.826,0.003589027,0.086668732

389677,RBM12B,3.12,4.34,4.35,4.98,3.91,4.3,1.65,3.9,3.43,3.44,5.08,3.15,3.3,4.8,2.8,3.88,4,2.44,3.37,2.18,3.31,2.52,2.01,3.14,2.39,2.21,2.38,5.63,2.17,2.15,4.31,2.07,3.79,2.818666667,0.003589027,0.086668732

26608,TBL2,16.69,16.54,19.18,8.84,10.59,19.8,16.07,17.9,13.92,21.37,18.84,17.65,14.18,19.85,22.19,13.23,21.71,10.38,16.39,14.7,11,12.52,10.7,9.6,12.94,10.53,12.51,15.88,15.05,15.32,15.31,17.63,16.97352941,13.364,0.003589027,0.086668732

22929,SEPHS1,20.7,9.25,12.49,19.37,15.24,16.23,9.59,22.35,19.52,8.02,22.83,15.18,12.82,15.98,13.28,14.16,20.21,8.94,5.86,11.92,18.44,10.69,9.24,14.4,10.92,7.16,8.96,10.62,13.85,11.6,14.9,10.23,15.71882353,11.182,0.003589027,0.086668732

90637,ZFAND2A,9.99,5.69,5.5,6.21,6.56,8.78,8.35,7.68,6.16,3.28,8.61,11.74,8.52,10.97,9.4,5.37,15.13,5.13,13.1,12.08,11.21,14.56,45.87,17.84,14,8.73,9.9,10.78,9.3,12.67,6.92,13.02,8.114117647,13.674,0.003589027,0.086668732

127253,TYW3,4.19,2.2,2.32,5.22,4.74,3.85,6.09,7.14,5.93,2.21,2.74,3.9,4.3,2.28,1.36,2.12,3.38,4.37,4.9,7.6,6.65,5.1,3.03,9.95,4.22,4.95,5.59,6.51,5.42,5.37,4.16,6.48,3.762941176,5.62,0.003589027,0.086668732

26872,STEAP1,0.45,1.49,0.34,0.36,0.06,0.11,7.24,1.33,0.33,1.46,12.42,2.81,33.92,0.07,2.27,1.74,0.56,0.48,9.82,16.51,22.11,9.19,37.11,1.12,73.67,4.19,5.94,1.96,3.6,3.84,1.07,16.65,3.938823529,13.81733333,0.003589027,0.086668732

1910,EDNRB,0.03,0,0.05,8.87,5.93,0,0.04,0,0,0.52,4.2,0,0,0.17,0,0.06,0,1.6,0.32,0.22,0.43,0.49,2.38,495.14,0.97,1.07,0.23,0.93,0.03,0.95,0.25,14.03,1.168823529,34.60266667,0.003595605,0.086668732

389421,LIN28B,0.04,0,0.02,1.3,1.44,0.51,0,0,0,0.08,0.44,0,0,0.87,0.25,0.25,0,0,0,0,0,0,0,0,0.02,0.01,0,0,0,0,0,0,0.305882353,0.002,0.003612488,0.086668732

64094,SMOC2,0,0,0,0,0,0,0,0.05,0,0,0,0.03,0,0,0,0,0.09,1,6,9.68,0,0,0.09,0,0.03,0.03,0,0.02,12.75,33.65,0.56,0,0.01,4.254,0.003613566,0.086668732

6707,SPRR3,0,0,0,0,0,0.08,0,0,0.1,0,0,0,0,0,0,0.08,0,0.4,0,0,0,0,0,0.2,0.9,4.7,0.28,0.89,0.75,1.06,1.22,0,0.015294118,0.693333333,0.003616568,0.086668732

6910,TBX5,0,0.05,0,0,0,0.04,0,0,0,0.03,0,0,0,0,0,0,0,0.06,3.15,5.09,0,0,0,0.02,0.03,0.06,0.02,23.47,0.2,0.97,0,0,0.007058824,2.204666667,0.003621376,0.086668732

117154,DACH2,1.2,0.13,0.08,0,0,0.65,0,0.05,0.04,0.11,6.85,0,0,0,6.33,6.07,12.39,0,0,0,0,0,0,0,0,0,0,0.08,0,0,0.42,0,1.994117647,0.033333333,0.003629195,0.086668732

284307,ZIK1,1.68,1.92,1.78,2.5,2.83,2.83,1.77,2.3,2.53,1.24,3.69,1.63,1.12,5.02,2.5,2.53,2.92,1.37,0.75,1.54,0.34,0.95,0.05,3,2.09,1.39,1.14,2.52,1.43,1.1,1.85,1.66,2.399411765,1.412,0.00363131,0.086668732

200576,PIKFYVE,5.49,6.11,5.1,8.45,7.29,5.73,4.49,6.38,5.47,5.03,6.2,4.4,4.3,5.44,4.64,6.38,5.79,3.49,4.93,3.94,5.94,2.94,2.1,7.5,4.08,3.01,3.7,5.46,3.49,4.2,6.12,3.38,5.687647059,4.285333333,0.003634408,0.086668732

9874,TLK1,6.98,5.29,6.68,8.09,7.6,7.57,7.84,9.93,13.53,4.43,6.63,7.31,6.58,7.98,5.46,7.86,7.31,5.95,4.37,7.22,8.54,4.93,3.85,5.18,6.1,4.32,6.1,6.17,5.63,6.65,6.04,6.44,7.474705882,5.832666667,0.003634408,0.086668732

340591,CA5BP1,3.24,2.29,3.1,4.8,4.54,4.38,3.91,2.23,3.43,4.06,3.2,2.66,4.67,3.85,3.13,1.79,4.11,4.68,5.92,4.87,7.02,7.76,5.55,6.52,4.32,3.92,4.82,2.95,3.46,5.29,2.32,4.32,3.493529412,4.914666667,0.003637508,0.086668732

25797,QPCT,0,0.18,2.7,0.41,3.72,0.13,8.24,3.05,12.91,0.33,0.31,9.43,30.43,1.55,0,0.04,0.05,16.64,0.95,34.22,13.51,28.34,1.32,213.39,10.83,6.36,6.28,1.8,9.66,6.73,3,10.87,4.322352941,24.26,0.003637508,0.086668732

5320,PLA2G2A,0,0,0.14,0,0,0,0.45,0,0,0,0,0.18,0,0.56,0,0,0,0,815.61,0.46,0.32,0.3,2909.5,0,1.83,0,0,0.15,0.77,101.71,0.07,0.2,0.078235294,255.3946667,0.003644834,0.086737255

563,AZGP1,0,0,0,0,0,0,0,0,0,0,0,0,0,0.08,0,0,0,0.06,0.65,0.38,0,0,436.47,1.94,0,0,0,0.1,0,0,0.05,0.07,0.004705882,29.31466667,0.003679537,0.087456315

286436,H2BFM,0.12,0.6,0.37,0.99,0.33,0.08,0,0.48,0.41,0.2,0.64,0.41,0.43,0.57,0.05,0.12,0.05,0.17,0,0.04,0,0,0,0.23,0.16,0,0,0.45,0.07,0,0.44,0,0.344117647,0.104,0.003697525,0.087776821

5020,OXT,0,0,0,0,0,0,0.34,0,0,0,0.16,0,0,0,0,0,0,0.17,0,0.51,0.3,0,0.3,0,0.64,0,0,0,0.43,0.43,0.14,0.37,0.029411765,0.219333333,0.003755082,0.089034724

1041,CDSN,0.67,1.13,0.75,0.39,0,0.99,0.15,0.51,0.33,0.48,0.2,0.5,0.18,0.23,0.34,0.2,1.09,0.09,0.03,0.03,1.16,0.2,0,0,0.19,0.03,0.03,0.13,0.08,0.05,0.51,0.27,0.478823529,0.186666667,0.003804975,0.090108084

56138,PCDHA11,0.39,0,0.04,1.49,0.28,0.04,0.35,0,0,0.06,3.32,0.03,0.03,0.12,0.29,1.32,0.38,0.26,0,0,0,0.05,0,0,0.03,0.12,0,0,0.06,0,0.02,0,0.478823529,0.036,0.003827358,0.090281456

401207,C5orf63,0.97,0.32,0.48,0,1.37,0.86,0.92,0.01,0,0.3,1.82,0,0,0.6,0.19,0.3,0.15,1.58,1.06,0.41,1.13,0.6,0.33,0.84,1.12,1.68,1.66,0.89,1.47,0.47,2.14,0.8,0.487647059,1.078666667,0.003827587,0.090281456

80036,TRPM3,0,0.01,0,0,0,0.02,0.01,0,0.03,0,0.04,0,0,0,0.07,0,0,0.46,0,0.06,0.13,0.14,0,0.09,0,0.32,0,0.06,0.01,0.04,0.2,1.39,0.010588235,0.193333333,0.003829113,0.090281456

90332,EXOC3L2,5.05,0.6,2.85,26.77,57.74,0.33,0.09,0.77,0.55,0.22,0.26,0.1,0.05,4.81,0.1,0.39,1.18,0.05,0.12,0.28,0.45,0.15,0.91,0,0,0,0.1,0.08,0.19,0.08,0.15,0.21,5.991764706,0.184666667,0.003830824,0.090281456

10418,SPON1,0.14,0.05,0.18,0.1,0.12,0.1,1.22,0.08,0.29,0.02,0.03,0.1,0.21,0.11,0.13,0.01,0.26,1.9,8.14,10.31,0.01,0.44,0.22,0.02,2.48,0.26,0.22,0.2,5.29,69.18,0.16,1.77,0.185294118,6.706666667,0.003840544,0.090383356

7634,ZNF80,0.5,0.69,0.47,0.81,0.96,0.24,0.15,0.18,0.06,0.85,1.33,0.6,0.94,0.39,0.7,0.39,0.33,0.11,0.25,0.28,0.24,0.12,0.22,0.18,0.65,0.37,0.18,0.36,0.04,0.21,0.3,0.16,0.564117647,0.244666667,0.003847032,0.090383356

158257,MIRLET7DHG,0.74,1.78,1.97,2.42,2.57,0.67,1.34,2.2,0.6,2.46,1.23,0.4,0.85,2.6,2.6,2.63,1.5,0.37,2.01,0.44,0.38,0.41,0.22,1.43,2.48,0.38,0.71,1.33,0.57,0.42,1.34,0.41,1.68,0.86,0.003853526,0.090383356

54995,OXSM,2.31,2.28,1.98,5.36,4.9,4.11,2.91,2.88,3.75,1.31,2.47,4.35,3.52,4.35,3.16,1.93,2.05,3.73,6.93,3.56,5.18,5.18,12.31,9.22,4.42,6.36,2.68,3.97,4.65,4.28,3.16,3.7,3.154117647,5.288666667,0.003856776,0.090383356

80311,KLHL15,1.34,5.84,2.23,3.28,2.97,3.04,1.07,3.44,2.69,1.75,2.02,4.4,4.69,6.91,1.91,1.94,1.82,0.91,4.23,1.47,1.06,0.38,1.94,3.09,1.25,1.84,0.94,2.61,1.23,1.09,1.62,1.49,3.02,1.676666667,0.00386328,0.090383356

57507,ZNF608,8.01,13.19,8.2,9.91,10.35,10.73,1.05,5.51,5.79,3.82,14.93,8.19,3.94,12.77,8.51,11.22,4.35,5.79,1.65,1.11,6.39,2.67,0.44,15.35,3.85,3.97,1.98,4.92,2.21,0.18,7.08,3.86,8.262941176,4.096666667,0.00386328,0.090383356

54102,CLIC6,0,0,0,0,0,0.06,0,0,0.07,0,0.02,0,0,0,0,0,0.12,0,1.45,0.04,0,0,0.29,0,0.08,2.2,0.04,0.12,0.02,1.1,0.23,0.02,0.015882353,0.372666667,0.00386761,0.090383356

5244,ABCB4,0.17,0.15,0,0,0.06,0.02,0.02,0.12,0,0.14,0.93,0,0.14,0,0,0.04,0,1.22,0.37,1.33,0,1.3,20.18,4.14,0.29,0.14,0.02,0,0.28,0.15,2.26,0.07,0.105294118,2.116666667,0.00390914,0.091244476

6990,DYNLT3,0,0,0.03,21.23,20.37,0.06,0.14,0.3,15.85,0.04,0.03,15.67,16.68,18.27,0,0,0,24.77,31.23,13.37,12.07,24.97,16.65,12.42,13.63,16.01,21.35,11.71,12.89,8.55,42.93,21.11,6.392352941,18.91066667,0.004033781,0.092588284

7498,XDH,1.39,1.04,0.93,0,0,0.5,0.15,1.34,0.09,0.46,0.45,0.3,1.07,0.2,0.8,1,0.33,0.04,0.02,0.41,0.01,0.03,5.55,0,0,0.03,0.16,0.04,0.08,0.08,0.01,0.11,0.591176471,0.438,0.004057482,0.092588284

8788,DLK1,0.65,298.65,62.62,3.28,68.68,0.27,0,0.1,0.48,16.76,2.47,243.63,42.38,0.49,11.93,3.56,1.5,1.88,0.38,0.25,0.04,0.11,1.42,0,2.94,0.1,0,9.18,0.13,0.04,1.2,0,44.55588235,1.178,0.004064268,0.092588284

6495,SIX1,181.93,82.93,112.67,0,0.41,150.81,8.7,214.69,77.47,98.5,98.23,72.51,17.03,0.69,92.78,210.52,79.64,2.26,79.39,2.69,0,1.3,0,0.13,14.14,25.09,9.05,46.8,16.88,4.28,42.3,6.84,88.20647059,16.74333333,0.00409148,0.092588284

2161,F12,0.92,0.44,0.58,0.79,0.19,0.38,0.76,0.68,1.08,1.8,0.69,0.12,0.18,1.09,1.49,0.6,0.93,0.15,1.02,0.04,0.92,0.38,64.04,0,0.14,0.16,0.08,0.03,0.32,0.03,0.09,0.04,0.748235294,4.496,0.00409148,0.092588284

171423,PDIA3P1,1.31,2.06,1.03,1.33,0.34,2.53,1.5,1.95,0.93,1.78,1.16,1.55,2.35,2.29,1.81,1.69,1.5,0.81,0.95,0.91,1.06,1.08,0.54,1.24,0.63,0.75,0.91,1.7,1.19,0.82,1.9,1.35,1.594705882,1.056,0.004098299,0.092588284

54942,FAM206A,5.78,6.34,5.13,5.93,5.93,7.88,5.78,6.98,3.77,5.3,5.29,4.96,4.74,7.8,4.42,7.54,4.76,4.82,6.01,9.77,8.29,7.75,7.78,7.34,6.54,6.3,6.76,9.65,6.24,6.4,6.48,7.99,5.784117647,7.208,0.004098299,0.092588284

1803,DPP4,0.72,0,0.17,0.23,0.26,0.61,27.35,0.65,0.02,0.98,0.34,70.1,61.58,25.47,0.27,0.17,0.02,0.75,15.59,51.27,4.23,3.72,1.17,166.03,37.29,5.72,24.96,1.07,2.95,36.02,5.85,8.03,11.11411765,24.31,0.004098299,0.092588284

5471,PPAT,4.87,3.11,4.13,4.76,3.51,4.44,2.97,3.92,3.82,6.76,3.04,4.16,6.09,5.81,4.27,3.66,7.34,2.73,1.88,1.68,6.68,2.64,3.08,5.04,3.07,3.21,1.24,5.45,2.78,2.36,3.12,2.73,4.509411765,3.179333333,0.004101711,0.092588284

143282,FGFBP3,1.13,0.65,1.88,0.78,0.91,0.53,0.3,4.36,1.65,0.37,0.61,0.33,0.99,1.52,0.75,1.53,0.78,1.31,0.39,0.22,0.24,0.1,0.05,0.47,0.35,0.6,0.48,0.81,0.18,0.23,1.4,0.44,1.121764706,0.484666667,0.004101711,0.092588284

2953,GSTT2,2.73,1.67,1.48,15.41,16.58,2.07,2.68,5.45,2.92,1.46,1.55,0,0,14.29,2.05,2.18,2.59,22.03,5.64,16.55,27.32,4.77,1.9,24.71,13.23,3.25,5.25,2.47,3.83,11.98,9.29,9.13,4.418235294,10.75666667,0.004101711,0.092588284

79187,FSD1,9.04,4.81,5.06,5.59,2.62,3.92,3.03,5.16,5.59,7.33,9.11,3.37,3.67,3.11,4.24,5.43,6.74,6.93,0.76,0.39,7.36,3.84,0.15,0.56,2.4,0.48,0.36,2.04,2.32,0.22,10,1.46,5.165882353,2.618,0.004101711,0.092588284

64762,GAREM1,0.41,0.72,0.22,0.08,0.04,1.98,0.59,0.15,0.17,0.46,0.64,0.3,0.85,0.34,0.89,0.08,1.52,1.4,0.86,1.39,0.13,0.1,2.33,2.38,1.86,1.63,0.71,0.95,1.61,1.46,0.99,1.02,0.555294118,1.254666667,0.004101711,0.092588284

7216,TRO,28.93,29.82,25.42,18.89,14.58,15.18,9.55,28.54,20.14,22.49,23.05,20.28,16,8.91,28.28,21.88,19.07,17.71,11.54,3.03,6.44,0.17,0.24,13.84,19.9,7.41,6.17,37.36,10.17,3.62,33.23,9.36,20.64764706,12.01266667,0.004109229,0.092588284

159090,FAM122B,5.16,9.88,10.34,26.11,18.04,11.66,5.43,6.76,4.95,17.62,8.17,10.36,9.1,27.25,5.62,6.56,8.6,4.57,7.85,5.05,7.14,6.24,1.34,9.48,7.49,6.29,4.17,7.5,3.11,5.08,6.84,6.49,11.27117647,5.909333333,0.004109229,0.092588284

147686,ZNF418,1.03,1.34,1.36,1.51,2.51,1.27,0.85,1.52,1.15,0.84,1.6,0.96,1.04,2.94,1.28,2.22,1.19,0.67,0.68,0.55,0.58,0.51,0,2.29,1.65,0.35,0.47,1.35,0.7,0.45,1.96,0.72,1.447647059,0.862,0.004109229,0.092588284

5217,PFN2,132.24,151.93,115.78,54.2,42.51,101.36,98.43,251.72,256.6,148.8,167.59,165.19,172.51,99.69,136.88,97.73,129.27,122.86,47.3,49.63,47.4,78.19,8.65,114.71,80.64,139.64,81.08,85.43,68,29.17,176.94,84.22,136.6135294,80.924,0.004109229,0.092588284

285598,ARL10,8.53,4.82,5.59,10.27,10.08,3.04,6.41,9.87,8.29,6.94,4.27,4.8,3.66,3.65,5.51,6.85,11.17,4.13,5.14,2.81,5.72,2.19,0.47,3.78,5.08,2.64,4.34,4.68,6,4.09,5.96,2.51,6.691176471,3.969333333,0.004109229,0.092588284

1609,DGKQ,6.99,6.24,8.22,12.58,12.85,5.81,5.76,7.71,7.35,5.79,6.63,8.38,5.92,9.81,6.89,6.36,9.48,4.49,3.67,6.43,6.46,8.15,1.4,13.41,5.42,2.15,5.91,3.54,4.55,4.75,7.36,3.61,7.81,5.42,0.004109229,0.092588284

5422,POLA1,1.97,2.58,2.68,7.09,7.77,4.66,1.58,5.74,4.96,2.96,2.51,3.03,1.99,4.94,1.11,1.84,3.62,1.57,1.64,1.9,3.49,0.98,0.71,2.41,1.67,3.26,1.21,1.55,2.45,2.1,2.35,1.86,3.59,1.943333333,0.004109229,0.092588284

55015,PRPF39,10.93,8.02,10.23,17.62,15.01,8.83,8.28,13.41,10.68,9.59,7.33,7.21,6.56,15.78,10.87,12.53,9.71,5.68,8.11,6.32,6.96,4.91,2.17,13.71,10.53,5.62,5.08,12.72,5.76,5.23,12.15,6.28,10.74058824,7.415333333,0.004109229,0.092588284

84687,PPP1R9B,50.56,22.69,22.32,25.97,33.8,24.03,18.24,50.65,30.09,23.6,25.92,31.27,19.45,19.36,26.09,27.52,36.16,20.81,12.61,25.33,23.25,13.94,2.77,25.75,24.15,9.68,21.68,20.77,20.34,20.73,29.54,19.01,28.68941176,19.35733333,0.004109229,0.092588284

51430,SUCO,11,10.04,11.91,9.21,11.29,7.89,7.12,11.44,8.25,15.28,11.08,9.07,16.66,14.27,9.68,6.79,9.9,16.97,7.67,5.26,8.44,9.89,6.84,10.24,8.96,6.42,7.45,8.09,6,3.9,8.92,4.81,10.64,7.990666667,0.004109229,0.092588284

55832,CAND1,22.69,20.05,21.9,26.74,23.84,27.59,22.67,25.86,29.22,31.12,25.69,22.16,18.48,31.26,22.73,21.37,30.36,22.9,15.09,16.06,26.82,16.45,7.76,22.19,23.35,15.23,19.82,19.84,21.59,19.87,29.86,16.67,24.92529412,19.56666667,0.004109229,0.092588284

6731,SRP72,31.4,23.09,31.93,31.83,31.33,34.67,31.28,31.97,30.98,32.82,28.63,26.53,27.8,30.47,32.21,23.32,37.89,26.73,18.42,27.82,39.74,24.12,19.19,22.52,23.4,30.21,25.43,27.16,26.78,27.76,28.98,27.91,30.47941176,26.41133333,0.004109229,0.092588284

60343,FAM3A,9.95,11.1,8.1,9.73,11.01,14.81,7.44,11.29,9.78,10.06,16.38,15.43,11.63,11.26,10.87,8.4,9.11,12,18.52,11.7,10.27,13.87,11.43,12.44,12.74,11.54,11.17,16.48,10.2,14.4,12.3,23.13,10.96176471,13.47933333,0.004109229,0.092588284

3459,IFNGR1,17.03,10.12,12.03,20.54,29.64,18.32,17.61,19.6,22.65,12.72,23.63,12.99,8.94,19.08,11.3,10.16,39.75,25.61,17.85,23.44,29.35,34.96,112.15,21.7,20.29,27.64,17.27,23.48,26.58,26.86,13.25,45.51,18.00647059,31.06266667,0.004109229,0.092588284

84883,AIFM2,4.3,1.28,2.84,0.71,0.53,3.38,3.85,7.37,4.43,2.17,5.35,2.14,1.25,5.27,1.47,2.82,6.37,4.92,3.28,7.07,4.56,4.25,11.28,9.57,6.75,7.1,5.62,2.44,4.99,10.29,2.59,6.08,3.266470588,6.052666667,0.004109229,0.092588284

440498,HSBP1L1,0.5,4.02,1.04,1.17,0.97,4.24,3.99,2.27,0.35,4.06,6.81,0.7,0,8.05,2.04,0.81,0.25,2.81,7.63,5.82,5.71,1.34,12.34,17.33,8.62,2.7,3.31,4.85,1.83,8.28,3.41,2.4,2.427647059,5.892,0.004109229,0.092588284

4837,NNMT,71.48,27.54,38.5,0.3,15.95,23.34,154.28,48.86,76.2,91.68,38.13,134.97,98.14,190.59,74.63,5.71,35.28,235.79,271.88,109.92,119.96,106.73,1222.15,0.28,317.81,739.27,237.87,8.64,205.17,153.81,51.48,135.56,66.21058824,261.088,0.004109229,0.092588284

3777,KCNK3,0.04,0.22,0.33,4.19,2.67,0.81,0,0.57,3.12,0.71,1.36,0.83,2.33,0.07,0.17,0.11,0.36,0.78,0.02,0,0,0,0.03,0,0.15,1.09,0.02,0.24,0.02,0,0.03,0.98,1.052352941,0.224,0.004215134,0.094864854

3000,GUCY2D,0,0,0,0,0,0,0,0,0,0.03,0.02,0,0,0,0,0,0,0,0,0.15,0,0,0.04,0,0.08,0.04,0.05,0.02,0.02,0.02,0.02,0,0.002941176,0.029333333,0.004231051,0.095113236

2837,UTS2R,0,0,0,0,0,0,0,0,0,0,0,0,0,0.17,0,0,0,0.21,0,0,0,0.08,0,0,0.09,34.71,0,0.06,5.11,0.18,0.06,0,0.01,2.7,0.004273876,0.095965266

90293,KLHL13,0.24,0.04,0,1.29,0.46,0.09,0.07,0,0.03,0.03,5.32,0,0,0.03,0.05,0,0,2.28,21.86,0,6.65,2.97,0,0.61,0.41,0.59,0.91,1.19,0.31,0.15,1.27,0.79,0.45,2.666,0.004299263,0.096418384

148738,HFE2,0,0,0,0,0,0,0,0,0,0,0.03,0,0,0.4,0,0,0,0.05,0,0.05,0,0,18.29,0.06,0,18.93,0,0.27,1.32,0.09,0,0.09,0.025294118,2.61,0.00432473,0.096418384

376693,RPS10P7,0.22,0.09,0.1,0.12,0.12,0.11,0.36,1.38,1.56,0.14,0.34,0,0,1.04,0.13,0,0.28,0.36,0.93,0.73,0.32,0.4,1.06,4.23,1.36,0.12,0.38,0.69,1.01,0.91,1.16,0.27,0.352352941,0.928666667,0.004324846,0.096418384

9729,KIAA0408,0,0,0,0.09,0,0.15,0,0.03,0,0,0.03,0,0,0,0,0,0,0,0,0.21,0.05,0,0.07,0.49,0.13,1.03,0,0.26,0.12,0,0.26,0.27,0.017647059,0.192666667,0.004330117,0.096418384

11248,NXPH3,0.17,0.08,0.03,0.32,0.92,1.21,0.41,0.12,0.15,0.05,0.41,0.06,0.19,0.03,0.09,0.13,0.05,2.48,2.4,2.63,0.77,0.42,0,0.31,0.63,0.06,3.06,0.36,0.53,1.26,0.27,2.11,0.26,1.152666667,0.004342693,0.096418384

647310,TEX22,0.51,0.28,0.49,0.48,0.52,0.91,0.57,0.32,0.63,0.88,1.14,0.83,0.39,0.53,0.51,1.16,0.35,0.18,0.5,0.22,0.08,0.03,0,0.81,0.5,0.13,0.23,0.76,0.2,0.52,0.46,0.37,0.617647059,0.332666667,0.004346267,0.096418384

1056,CEL,0.94,0.98,0.53,1.65,1.47,0.51,0.36,0.86,0.89,0.87,1.07,0.32,0.49,2.37,1.68,1.19,1.11,0.75,0.18,1.3,0.23,0.51,0.06,0.18,0.97,0.27,0.21,0.71,0.33,0.56,0.61,0.59,1.017058824,0.497333333,0.004349843,0.096418384

6416,MAP2K4,9.13,8.79,11.49,13.03,11.9,8.72,7.5,11.65,8.73,8.91,11.98,10.46,8.5,13.38,8.9,6.95,9.11,8.9,6.51,8.36,7.79,6.64,4.38,9.68,7.27,10.74,6.97,8,7.57,7.83,10.47,7.27,9.948823529,7.892,0.004349843,0.096418384

5584,PRKCI,10.45,12.98,11.38,7.89,6.59,9.79,11.3,11.36,10.3,10.98,8.46,11.54,12.41,13.38,8.46,9.32,9.59,14.64,7.28,7.51,6.38,6.78,1.34,4.55,8.59,7.53,9.59,7.6,9.09,9.67,10.56,7.67,10.36352941,7.918666667,0.004349843,0.096418384

6666,SOX12,18.79,26.36,17.1,23.68,27.16,13.04,7.38,17.95,17.21,14.68,15.43,19.08,13.35,18.9,17.58,18.38,8.8,13.91,7.61,7.81,15.33,8.76,1.34,25.17,13.51,5.81,6.05,14.68,13.39,9.31,17.28,9.36,17.34529412,11.288,0.004353421,0.096418384

10140,TOB1,8.13,8.69,8.86,3.53,2.81,13.18,10.1,10.34,6.13,5.89,17.45,9.7,10.46,7.96,5.5,4.5,3.83,15.69,16.11,17.6,4.89,5.75,29.14,13.58,13.19,13.49,11.86,18.98,7.57,13.18,8.84,13.79,8.062352941,13.57733333,0.004353421,0.096418384

83879,CDCA7,2.83,0.83,1.83,9.71,5.33,1.7,0.18,3.81,3.9,1.67,0.81,1.21,0.59,6.87,2.61,4.3,19.52,0.78,0.46,0.43,6.56,0.81,0.11,0.56,0.57,1.03,0.16,2.31,1.14,0.15,4.11,0.54,3.982352941,1.314666667,0.004353421,0.096418384

7151,TOP1P1,0.23,0.05,0,0,0,0.33,0,0.07,0.07,0,0.38,0.18,0.04,0,0,0.03,0.08,0.34,0.14,0.23,0.06,0.07,0.53,0.37,0.25,0.03,0.17,0.06,0.33,0.25,0.19,0.33,0.085882353,0.223333333,0.00437162,0.096711557

10586,MAB21L2,13.06,48.43,52.14,0,0,18.44,1.34,69.21,24.67,12.76,11.43,92.46,20.02,1.82,1.78,37.44,11.93,3.69,9.81,0,0.02,0.03,0.7,0,30.96,0.2,17.15,3.09,0.75,0,0.67,0,24.52529412,4.471333333,0.004492522,0.09927353

220108,FAM124A,0.13,0,0.06,0.95,1.57,0.01,0.06,0.02,0.03,0,0.15,0.3,0.13,0.08,0.02,0,0.06,0.26,0.69,0.03,3.91,2.52,0.09,4.2,0.56,0.1,0.19,0.03,0.18,0.32,0.75,0.32,0.21,0.943333333,0.004566711,0.099773141

100506385,LINC01426,0.56,0.32,0.4,0.57,0.74,0.92,2.22,0.51,0.58,0.58,0.58,0.86,0.17,1.09,0.63,0.37,0.62,0.9,1.03,1,0.44,1.01,0.35,0.58,1.93,1.5,1.56,1.25,2.31,2.41,1.09,1.39,0.689411765,1.25,0.004581632,0.099773141

197196,LINC00311,0.2,0.21,0.17,0.49,0.48,0.08,0.18,0.41,0.35,0.22,0.13,0.24,0.42,0.46,0.27,0.16,0.36,0.09,0.12,0.04,0.16,0.19,0,0.39,0,0.23,0.22,0.11,0.15,0.21,0,0.2,0.284117647,0.140666667,0.004592841,0.099773141

129450,TYW5,1.64,1.26,1.58,2.64,1.62,1.59,1.59,2.42,1.71,1.41,1.59,1.32,1.73,2.51,1.48,1.27,1.42,1.28,1.27,1.13,1.84,1.35,1.4,2.07,1.41,1.43,1.16,1.16,1.04,0.83,1.56,1.01,1.692941176,1.329333333,0.004596581,0.099773141

222696,ZSCAN23,0.84,0.8,0.92,0.99,0.68,0.49,0.41,0.72,0.69,0.44,1.31,0.4,0.28,0.52,0.79,1.12,0.6,0.73,0.25,0.17,0.15,0.03,0,0.43,0.77,0.15,0.26,0.98,0.41,0.29,0.71,0.41,0.705882353,0.382666667,0.004604065,0.099773141

353274,ZNF445,3.44,2.89,3.3,4.63,4.49,3.36,2.01,4.22,3.87,3.16,4.91,3.71,3.05,4.31,3.42,4.07,4.22,2.81,2.09,2.81,2.81,1.94,0.91,4.5,3.8,2.03,2.77,3.83,2.4,2.7,4.01,2.58,3.709411765,2.799333333,0.004604065,0.099773141

92285,ZNF585B,0.05,0.05,0.03,3.19,2.31,0.07,1.74,2.88,1.79,0.14,0.01,0.08,0.09,1.51,0.01,0.01,0.02,2.19,2.32,3.01,1.4,1.39,0.32,1.96,2.89,1.59,1.56,2.62,1.67,2.69,2.77,1.93,0.822352941,2.020666667,0.004604065,0.099773141

84935,MEDAG,0.03,0.02,0.05,3.29,1.05,3.56,79.74,75.08,4.54,0.36,0,0.18,0.15,16.3,0,0.06,0,13.56,18.14,91.71,7.23,7.25,0.59,0.04,28.63,8.77,43.34,3.48,23.91,89.51,3,41.04,10.84764706,25.34666667,0.00460781,0.099773141

85459,CEP295,2.23,1.75,1.72,3.69,2.89,1.99,1.44,2.76,2.47,1.81,1.83,2.01,1.49,3.26,2.02,2.39,2.99,1.26,1.71,1.88,2.8,1.22,0.57,2.8,1.96,1.65,1.26,1.67,1.18,1.32,1.9,1.6,2.278823529,1.652,0.004615305,0.099773141

100132101,HERC2P7,4.01,3.14,3.35,8.67,6.34,3.15,2.21,4.32,3.75,1.99,3.59,1.99,1.9,7.55,3.72,3.89,4.02,1.96,4.54,2.39,2.1,1.69,0.56,3.04,5.72,2.17,1.89,3.06,1.91,1.4,2.57,1.24,3.975882353,2.416,0.004619055,0.099773141

7637,ZNF84,4.57,4.57,4.94,11.05,8.69,4.84,3.31,4.49,4.66,4.6,8.57,4.42,4.32,5.62,5.04,6.01,4.41,3.17,4.18,2.77,2.93,3.79,0.82,5.24,4.72,3.52,2.48,6.47,3,2.86,6.02,2.71,5.535882353,3.645333333,0.004619055,0.099773141

1036,CDO1,1.9,0,0.43,0.34,0.19,24.22,0.19,0.1,0.79,0.06,6.95,0.69,0.45,0.12,0.33,1.54,0.85,10.07,13.89,0.99,1.74,0.27,55.32,1.29,5.83,0.56,1.19,2.95,4.11,2.25,3.17,0.54,2.302941176,6.944666667,0.004619055,0.099773141

51320,MEX3C,18.37,14.85,14.55,16.12,11.54,14.6,14.45,15.9,15.96,11.35,15.94,12.07,10.69,18.28,14.44,11.58,17.03,14.21,11.36,11.64,14.58,10.67,5.28,13.19,10.27,7.99,12.34,10.76,16.02,12.04,14.21,8.57,14.57176471,11.542,0.004619055,0.099773141

8495,PPFIBP2,1.52,0.12,0.48,1.98,0.07,0.28,2.1,0.22,11.13,0.03,7.48,0.4,0.03,0.98,0.29,0.18,2.69,1.41,1.32,4.79,3.92,0.32,6,7.69,2.29,5.05,6.76,0.39,3.42,6.12,3.01,2.98,1.763529412,3.698,0.004619055,0.099773141

80154,GOLGA2P10,4.91,4.21,8.23,5.16,2.6,1.84,1.75,4.02,4.47,3.89,8.24,2.47,3.7,7.1,7.71,6.89,5.14,4.84,1.56,1.26,3.13,1.26,1.25,5.36,2.34,1.33,2.53,3.6,2.94,2.65,5.09,0.89,4.842941176,2.668666667,0.004619055,0.099773141

55277,FGGY,2.71,3.02,2.56,1.89,2.28,3.83,3.12,2.04,1.76,2.35,2.71,5.27,4.49,4.16,2.17,1.62,1.4,3.49,4.26,3.08,3.77,5.62,12.67,4.38,5.61,13.9,2.84,7.06,2.64,1.86,4.27,3.1,2.787058824,5.236666667,0.004619055,0.099773141

216,ALDH1A1,0.03,0.23,0.03,5.72,94.28,0.12,1.41,0,0.07,0.08,0.06,0,0,0.33,0,0,0,0.95,0.14,0.94,228.05,31.07,12.93,0.18,0,174.42,0.21,0.3,5.91,0.85,0.08,6.02,6.021176471,30.80333333,0.004678155,0.099773141

414152,C10orf105,0,0,0,0,0.07,0,0.03,0,0,0.06,0.02,0,0,0,0,0,0,0.02,0.55,0.99,0.03,0,0.12,0,0.02,0.3,0.03,0,0,0.11,0.01,0.09,0.010588235,0.151333333,0.0046853,0.099773141

1143,CHRNB4,0.05,0.04,0.48,0.05,0,0,0,0,0.03,0,0.21,0,0,0,0.07,0.05,0.13,0,0,0,0,0,0,0.04,0,0,0,0,0,0,0,0,0.065294118,0.002666667,0.004686597,0.099773141

84187,TMEM164,3.9,8.68,5.78,10.19,10.28,9.05,2.4,10.93,8.95,18.04,4.8,15.06,6.89,12.92,3.82,3.41,4.44,4.28,3.63,2.04,7.82,8.53,1.03,6.97,4.37,7.84,2.23,3.28,2.16,2.61,5.44,4.15,8.208235294,4.425333333,0.004694303,0.099773141

441518,FAM127C,12.44,28.68,16.49,43.21,18.8,27.52,14.82,23.23,16.64,18.7,23.79,38.4,19.28,32.83,3.44,10.17,14.01,14.57,12.63,15.8,13.18,11.32,1.31,12.6,23.99,6.18,12.34,13.97,9.92,12.77,18.61,15.23,21.32058824,12.96133333,0.004694303,0.099773141

8473,OGT,68.31,36.53,69.57,43.09,36.35,32.3,62.97,69.54,64.43,41.34,26.34,35.53,27.68,62.71,64.76,62.92,38.24,20.64,45.05,27.12,18.56,19.62,9.98,39.06,44.12,17.33,34.12,52.12,32.31,28.19,48.58,30.91,49.56529412,31.18066667,0.004694303,0.099773141

5962,RDX,24.23,19.44,23.89,166.05,125.22,41.32,20.56,29.19,31.38,22.62,33.27,21.4,18.62,30.05,25.42,20.51,30.78,23.83,18.85,19.86,60.72,21.36,11.22,31.36,17.18,23.6,16.49,18.61,18.1,14.39,24.49,16.09,40.23235294,22.41,0.004694303,0.099773141

27314,RAB30,9.47,9.93,6.72,2.9,6.16,6.05,3.17,13.86,13.16,3.32,11.25,7.26,3.4,0.85,5.45,10.76,9.06,2.15,4.71,4.28,4.19,2.47,0.44,1.95,7.46,1.43,5.03,2.4,3.76,4.9,5.59,3.22,7.221764706,3.598666667,0.004694303,0.099773141

23158,TBC1D9,9.92,8.09,7.55,23.49,20.9,7.14,7.58,9.18,8.6,7.4,18.91,8.49,9.74,8.76,5.71,4.95,9.94,7.81,2.56,4.9,15.32,19.56,0.88,2.48,6.85,4.22,4.11,6.48,6.6,6.39,8.66,5.87,10.37352941,6.846,0.004694303,0.099773141

196441,ZFC3H1,11.37,9.01,11.24,13.42,11.59,7.64,14.87,10.55,8.16,11.35,14.3,8.21,9.61,13.55,10.96,10.13,6.98,6.63,13.23,5.33,7.93,6.66,2.51,10.15,9.9,7.51,7.97,12.34,5.87,7.21,11,5.02,10.76117647,7.950666667,0.004694303,0.099773141

100287216,SH3RF3-AS1,4.36,4.11,3.03,2.63,4.99,4.56,1.08,1.93,5.8,7.16,2.58,4.27,2.45,3.08,3.28,3.31,4.2,1.11,0.6,0.93,2.37,6.06,0.15,0.18,4.26,2.05,1.91,3.96,1.29,1.35,3.81,1.24,3.695294118,2.084666667,0.004694303,0.099773141

54765,TRIM44,23.07,19.89,26.15,29.6,34.3,20,19.27,24.01,24.04,21.81,9.87,18.48,18.02,25.02,19.55,22.24,24.64,16.1,14.49,15.01,26.53,17.23,7.37,16.53,19.53,18.72,16.55,24.25,18.08,17.61,23.91,16.28,22.35058824,17.87933333,0.004694303,0.099773141

55300,PI4K2B,5.5,5.22,4.52,9.85,8.16,6.36,9.43,9.69,6.98,7.6,2.62,7.42,12.09,11.05,8.99,4.39,8.12,3.45,3.98,5.02,7.09,4.97,4.18,2.98,5.82,3.9,4.61,6.19,5.55,7.05,6.33,6.42,7.528823529,5.169333333,0.004694303,0.099773141

284114,TMEM102,3.08,1.83,2.37,3.77,4.29,4.73,2.43,2.2,3.76,1.96,3.7,2.11,1.33,1.92,2.24,1.47,3.21,1.34,0.3,0.76,3.52,2.56,1.48,9.77,2.23,1.43,1.04,0.98,1.69,0.88,1.49,1.01,2.729411765,2.032,0.004694303,0.099773141

8991,SELENBP1,3.17,33.67,4.34,2.34,1.56,13.61,0.65,2.28,0.39,9.27,34.98,13.68,8.3,11.8,3.45,2.37,0,9.45,68.93,12.97,13.79,1.15,39.42,141.7,37.77,4.23,9.21,33.25,5.64,18.05,15.17,33.32,8.58,29.60333333,0.004694303,0.099773141

8742,TNFSF12,10.64,26.7,6.19,14.08,31.84,9.76,10.83,19.14,12.89,15.87,20.57,1.44,11.93,3.12,9.64,5.91,6.83,13.41,18.06,37.93,22.54,15.69,10.52,56.18,27.08,5.87,21.75,25.4,14.44,26.74,31.51,32.83,12.78705882,23.99666667,0.004694303,0.099773141

4946,OAZ1,348,342.25,286.58,266.19,317.86,451.57,440.32,387.69,284.73,276.76,312.13,371.2,336.04,296.94,318.33,214.37,321.15,339.31,371.42,536.59,533.21,436.71,311.63,381.27,355.29,511.37,522.19,225.67,430.33,480.1,340.34,444.99,327.7711765,414.6946667,0.004694303,0.099773141

5429,POLH,2.64,3.51,3.35,4.95,3.45,5.97,6.65,4.25,3.61,5.33,2.6,3.05,4.63,6.05,3.5,2.89,4.05,6.99,3.69,8.81,4.71,10.93,1.22,4.17,6.69,5.73,5.16,6.09,4.41,9.39,5.41,8.54,4.145882353,6.129333333,0.004694303,0.099773141

64114,TMBIM1,43.49,34.15,29.68,38.86,43.07,77.76,60.81,68.65,39.08,51.88,86.07,6.98,36.06,54.48,39.84,44.97,52.64,43.37,111.93,83.35,60.25,82.78,52.74,102.99,80.3,61.13,72.47,60.85,37.8,61.61,50.38,70.94,47.55705882,68.85933333,0.004694303,0.099773141

203859,ANO5,0,0,0,0,0,0,0,0,0,0.01,0.01,0,0,0.03,0,0,0,0.54,0,0,1.52,0.05,1.06,3.15,0,0.16,0.04,0,0.01,0,0.09,0,0.002941176,0.441333333,0.00474328,0.100704273

4129,MAOB,0.28,0,0,0,0,0,0.09,0,0,0,1.4,0,0,0.19,0,0.03,0.03,4.09,5.14,0.03,0,0,21.04,0.1,3.95,0.25,0,1.32,0.05,0.2,0.05,1.47,0.118823529,2.512666667,0.004778462,0.101340825

80115,BAIAP2L2,0.03,0.08,0.29,0.54,0.18,0.26,0.65,0.38,0.16,0.09,0.24,0.12,0.08,0.27,0.16,0.1,0.08,0.18,0.5,1,0.16,0.16,1.05,0.93,0.5,1.36,0.31,0.48,0.22,0.78,0.47,0.16,0.218235294,0.550666667,0.004801588,0.101720603

147495,APCDD1,0.03,0.02,0,0.03,0,0.08,0,0.06,0,0.11,0,0.6,1.16,1.25,0.1,0,0,1.15,5.18,11.18,0,0,0.16,4.46,0.3,3.84,0,3.95,0.54,8.61,4.04,0.71,0.202352941,2.941333333,0.004820746,0.101911108

1475,CSTA,0.27,0.22,0.55,0,0,0.45,0.2,0.72,0.21,1.19,0.56,0,1.36,0.12,0.33,1.1,0,0.29,0.68,7.87,0,0.22,17.86,0.43,6.7,6.44,7.15,3.18,1.99,9.19,0.24,2.72,0.428235294,4.330666667,0.004821039,0.101911108

8626,TP63,0.18,0.06,0,0,0,0,0.2,0.12,0,0.06,0.02,0.04,0.08,0.18,0.04,0.25,0,0.23,1.21,0.1,0,0.09,0,0.07,2.7,2.19,0.88,0.99,0.14,0.15,0.73,3.15,0.072352941,0.842,0.004847559,0.102266465

8787,RGS9,1.24,0.89,1.71,3.4,4.17,0.43,0.06,0.42,0.77,0.58,2,0.94,0.06,5.18,1.17,0.43,0.5,0.29,0.04,0,0.25,2.13,1.21,0.1,0,0,0,1.12,0.16,0.08,0.5,0.1,1.408823529,0.398666667,0.004848343,0.102266465

56606,SLC2A9,0.35,0.13,0,2.41,4.16,0.73,0.71,0.18,0.09,0.1,0,0,0.29,1.31,0.33,0,0.05,0.34,1.45,1.58,0.79,0.42,1.28,0.79,1.38,1.01,0.49,0.66,0.71,0.94,0.78,1.08,0.637647059,0.913333333,0.004856161,0.10232062

10351,ABCA8,0.02,0.35,0.01,0.03,0.34,3.68,10.55,6.85,0.83,0.03,4.87,0.1,0.86,0,0.06,0.01,0.11,0.39,3.83,2.3,1.6,0.02,1.28,0.04,22.38,3.78,5.59,17.45,5.81,42.31,19.06,53.56,1.688235294,11.96,0.004891426,0.102344074

203522,INTS6L,1.64,4.86,6.67,3.54,4.08,2.52,0.76,2.31,2.77,6.36,1.85,2.73,4.09,4.87,3.28,1.52,1.43,1.25,2.33,0.55,1.85,0.56,0.61,2.72,2.15,0.61,0.7,3.14,2.35,1.07,3.15,1.66,3.251764706,1.646666667,0.004895354,0.102344074

2122,MECOM,2.74,8.86,3.55,31.95,10.44,4.2,1.64,1.15,1.54,4.35,21.49,2.39,1.09,5.1,2.52,2.61,2.32,1.85,0.1,0.18,22.11,6.41,0.35,0,2.26,0.43,0.66,4.35,0.26,0,0.4,1.99,6.349411765,2.756666667,0.004895354,0.102344074

90826,PRMT9,3.24,4.13,3.35,4.28,3.78,4.91,2.95,3.87,3.27,2.96,4.18,3.6,2.9,4.65,3.79,3.91,3.64,3.51,2.52,2.23,3.57,2.55,1.33,4.71,2.9,3.51,2.35,3.94,2.68,2.42,3.52,2.82,3.73,2.970666667,0.004895354,0.102344074

26167,PCDHB5,0.1,6.05,2.61,17.4,7.98,8.39,1.75,4.25,6.62,2.49,8.42,0.03,0.32,13.21,2.27,2.63,4.06,4.72,1.94,0.6,0.38,1.12,0.43,0.32,1.51,0.9,1.33,1.59,1.28,0.57,0.69,0.39,5.210588235,1.184666667,0.004899283,0.102344074

100874058,COX10-AS1,2.75,2.97,1.95,2.44,2.18,1.73,2.54,3.2,3.6,2.99,1.16,2.21,2.35,4.59,1.13,2.57,1.98,1.98,2.12,1.89,0.79,1.72,0.51,1.88,1.27,2.06,1.54,2.66,2.08,1.94,2.02,1.45,2.490588235,1.727333333,0.004899283,0.102344074

140890,SREK1,14.04,10.1,12.11,24.11,20.03,10.02,11.82,15.99,12.25,10.77,11.74,9.19,9.18,16.55,12.06,13.82,14.2,7.41,10.66,7.24,10.02,6.15,5.99,16.92,14.03,6.67,6.71,12.22,8.27,6.24,14.01,6.87,13.41058824,9.294,0.004899283,0.102344074

80216,ALPK1,1.95,3.07,1.06,0.81,1.13,2.23,1.32,2.47,2.04,1.96,3.02,2.76,3.19,0.99,3.17,2.17,0.53,1.52,2.3,2.83,3.03,1.19,2.06,5.44,6.98,5.32,3.09,3.04,3.4,3.17,3.14,3.53,1.992352941,3.336,0.004899283,0.102344074

51252,FAM178B,0,0,0.22,0,0,0,0,0,0,0,0,0,0,0,0,0,0,0.1,0.16,0.19,0,0,0,2.34,0,1.82,0,0,0.16,0,0.07,0.31,0.012941176,0.343333333,0.00496052,0.103401638

3355,HTR1F,0,0,0,0.07,0,0,0,0,0,0,0,0,0,0,0,0,0,0,0,0.15,0.04,0,0,0.03,0.03,0,0.05,0,0.1,0.06,0.26,0,0.004117647,0.048,0.00496052,0.103401638

4633,MYL2,0,0,0,0,0,0,0,0,0,0.12,0.09,0,0,0,0,0,0,0.1,0,0,0,0.11,0,0.11,0.24,523.09,0.2,0.32,0.08,0.08,0,0,0.012352941,34.95533333,0.004971647,0.103522871

390245,KDM4E,0,0,0,0,0,0,0,0,0,0,0,0,0,0,0,0,0,0,0,0,0.03,0,0,0,0,0,0.04,0.03,0.03,0.03,0.11,0,0,0.018,0.005024319,0.104507975

284835,LINC00323,0.14,0,0,0,0,0,0,0,0.08,0,0,0,0,0,0,0,0,0.12,0,0,0.63,0.04,0,0.13,0,0,0.12,0.03,0.07,0.07,0.03,0.04,0.012941176,0.085333333,0.005074795,0.104976626

11122,PTPRT,0,0,0,0,0,0,0,0,0,0,0,0,0,0,0,0,0,0.03,0,0.01,0,0,0.01,0,0,0,0,0,0.02,0.06,0.01,0,0,0.009333333,0.005076517,0.104976626

85508,SCRT2,0,0,0,0,0,0,0,0,0,0,0,0,0,0,0,0,0,0,0,0,0,0,0,0,0.08,0.07,0,0.04,0.02,0.02,0.02,0,0,0.016666667,0.005076517,0.104976626

246119,TTTY10,0,0,0,0,0,0,0,0,0,0,0,0,0,0,0,0,0,0,0.04,0,0,0,0,0.11,0,0,0,0.04,0.04,0,0.12,0.16,0,0.034,0.005076517,0.104976626

2104,ESRRG,0,0,0,0,0,0,0,0,0,0,0,0,0,0,0,0,0,0.19,0,0,0,0,0,0.05,0,0.19,0,0.01,0,0,0.01,0.02,0,0.031333333,0.005093984,0.104976626

349149,GJC3,0,0,0,0,0,0,0,0,0,0,0,0,0,0,0,0,0,0,0,0,0.25,0.16,0.64,0.16,0,0,0,0,0,0.06,0.06,0,0,0.088666667,0.005093984,0.104976626

63973,NEUROG2,0,0,0,0,0,0,0,0,0,0,0,0,0,0,0,0,0,0.1,0,0,0,0,0,0,0.04,0.1,0,0.03,0.03,0.06,0,0,0,0.024,0.005093984,0.104976626

2346,FOLH1,0,0,0,0,0,0,0,0,0,0,0,0,0,0,0,0,0,0,0,0,2.17,0.07,0.37,0,0,0,0,0,0.03,0,0.03,0.24,0,0.194,0.00510273,0.104976626

9087,TMSB4Y,0,0,0,0,0,0,0,0,0,0,0,0,0,0,0,0,0,0,0,0,0,0,0,0.15,0,0.44,0,0.08,0.08,0,0.26,0.21,0,0.081333333,0.00510273,0.104976626

4617,MYF5,0,0,0,0,0,0,0,0,0,0,0,0,0,0,0,0,0,0,0,0,0.05,0,0,0,0,23.44,0,0.04,1.94,0,0.04,0.06,0,1.704666667,0.00510273,0.104976626

4653,MYOC,0,0,0,0,0,0,0,0,0,0,0,0,0,0,0,0,0,0,1.11,3.91,0,0,0,0,3.41,0,0.08,0,0.03,1.63,0,0,0,0.678,0.005111484,0.104976626

645323,LINC00461,0,0,0,0,0,0,0,0,0,0,0,0,0,0,0,0,0,8.4,0,0,0,0,0,0.18,0.06,0.12,0,0,0,0.04,0.35,0,0,0.61,0.005111484,0.104976626

55796,MBNL3,0,0.02,0.01,1.86,0.94,0.4,0.02,0.02,0.01,0.01,0.13,0.14,0.02,0.75,0.05,0.03,0.06,0.08,0.32,0.24,0.55,0.11,5.84,1.07,1.24,0.2,0.09,0.54,0.2,0.3,0.41,0.25,0.262941176,0.762666667,0.005137357,0.105021029

9496,TBX4,0,0,0,0,0,0,0.06,0.03,0.03,0.08,0,0,0,0.04,0,0.03,0,0,79.01,0.03,0,0.07,0,0,0.36,0.3,0.23,21.19,0.03,0.08,0.08,0.14,0.015882353,6.768,0.005152539,0.105021029

51561,IL23A,0.14,0.06,0.12,0,0.15,0.28,0.23,0.08,0.17,0.09,0.22,0,0.18,0.29,0.17,0.29,0.45,0.31,0.6,0.39,0.21,0.26,0.55,0.68,0.58,0,0.33,0.77,0.26,0.2,0.19,0.17,0.171764706,0.366666667,0.005157832,0.105021029

387755,INSC,0.03,0.08,0,0,0,0.02,0,0,0,0.07,0.52,0,0.03,0.03,0.09,0,0,0.19,2.46,0.55,0,0.21,0,0,0.71,0.7,0.43,0.56,0.02,0.09,0.09,0.06,0.051176471,0.404666667,0.005181247,0.105021029

90381,TICRR,0.89,0.34,0.6,0.75,0.44,0.85,0.49,1.08,0.77,0.42,0.43,0.62,0.66,2.38,1.29,0.61,1.91,0.32,0.4,0.6,1.28,0.24,0.04,0.2,0.35,0.16,0.32,0.48,0.56,0.75,1.03,0.28,0.854705882,0.467333333,0.005186573,0.105021029

201266,SLC39A11,10.99,9.16,9.38,4.92,6.43,10.78,4.66,8.74,5.91,12.82,10.99,8.88,9.49,14.96,8.65,8.08,3.81,3.24,5.26,3.5,2.92,5.84,5.69,10.35,4.97,6.13,3.56,11.96,4.44,4.18,7,5.69,8.744117647,5.648666667,0.005190686,0.105021029

619190,FDPSP2,0.94,0.54,0.7,0.92,0.81,0.66,0.53,1.4,1.15,0.82,0.67,0.95,0.5,1.31,0.99,0.62,1.59,0.55,1.14,0.34,0.36,0.37,0.66,0.85,0.92,0.23,0.6,0.58,0.39,0.51,0.73,0.43,0.888235294,0.577333333,0.005190686,0.105021029

285550,FAM200B,6.91,3.08,3.32,4.03,3.98,3.74,6.37,4.88,6.66,3.05,4.08,3.95,3.91,5.14,3.38,3.67,7.92,2.87,1.95,2.88,2.27,3.64,2.09,6.57,4.93,3.05,2.99,4.05,2.83,3.12,3.67,3.81,4.592352941,3.381333333,0.005190686,0.105021029

136,ADORA2B,2.88,15.41,3.39,3.71,3.55,4.86,5.29,4.71,2.67,7.04,2.88,4.79,6.59,1.61,3.77,5.02,5.06,3.47,4.27,1.57,2.5,9.23,0.15,0.74,3.79,2.09,2.21,2.6,1.76,3.47,2.25,3.54,4.895882353,2.909333333,0.005190686,0.105021029

284695,ZNF326,12.51,3.63,9.23,17.65,16.07,8.19,9.87,13.27,10.94,4.24,10.56,8.29,9.88,10.36,10.99,11.66,13.7,5.43,7.14,5.92,8.05,5.22,5.51,13.1,11.21,4.46,5.55,7.84,7.8,4.95,9.59,4.46,10.64941176,7.082,0.005194801,0.105021029

404665,CACTIN-AS1,4.71,4.52,2.61,6.13,5.76,3.14,2.58,3.95,4.6,3.84,3.9,4.58,3.87,5.18,4.59,3.11,4.36,3.33,3.33,2.68,3.67,2.77,0.81,4.06,4.1,2.44,3.27,3.86,3.44,3.1,3.74,2.99,4.201764706,3.172666667,0.005194801,0.105021029

29855,UBN1,12.97,11.23,11.92,16.04,17.26,15.48,8.7,16.3,13.36,10.82,11.51,13.36,10.55,13.46,11.5,15.3,11.94,9.05,8.55,9.65,14.16,13.08,3.45,15.55,8.47,9.66,9.41,8.63,9.12,9.27,13.54,7.21,13.04117647,9.92,0.005194801,0.105021029

79680,C22orf29,6.56,4.16,5.3,11.03,15.64,21.33,4.07,6.42,7.02,4.47,5.3,4.61,2.81,25.11,5.77,4.66,8.34,3.07,4.13,3.34,12.56,15.99,0.93,3.95,2.99,4.86,2.98,3.18,4.1,3.82,5.04,2.9,8.388235294,4.922666667,0.005194801,0.105021029

10129,FRY,0.05,0.02,0.04,6.41,5.22,0.05,8.42,0.73,1.36,0.75,1.04,0.06,0.03,1.79,0.28,0.57,0.31,2.18,5.12,3.22,5.83,1.16,2.24,2.86,1.65,4.85,8.49,0.46,1.75,1.95,1.06,1.89,1.595882353,2.980666667,0.005194801,0.105021029

28232,SLCO3A1,0.23,0.02,0.79,0,0.22,0.11,3.2,0.2,0.35,1.93,6.27,5.55,2.87,0,0.05,0.01,1.88,3.51,6.75,4.16,1.8,0.46,0.24,1.15,4.57,2.63,4.63,0.25,4.76,9.36,3.32,4.59,1.392941176,3.478666667,0.005194801,0.105021029

339751,MLK7-AS1,0.03,0.14,0.22,0.09,0,0.14,0.09,0.06,0.33,0.26,0.12,0.1,0.36,0.08,0,0.2,0.25,0.03,0.03,0.06,0,0.03,0,0,0.08,0.03,0.19,0.1,0.05,0.08,0.07,0.03,0.145294118,0.052,0.005242765,0.105021029

4703,NEB,0.11,0.01,0.01,0.05,0.07,0.04,0.03,0.02,0.05,0.05,0.05,0.01,0.01,0.29,0.01,0,0.01,0.13,0.08,0.02,0.06,0.06,0.03,0.08,0.06,71.38,0.03,0.14,0.65,0.09,0.07,0.04,0.048235294,4.861333333,0.005267958,0.105021029

100507098,ADAMTS9-AS2,0.1,0.03,0,0.45,0.31,0,0,0,0,0.25,0.07,0,0.04,0.26,0.31,0,0,0.45,2.98,0.39,0.28,0.08,0.06,0.04,0.79,0.11,0,1.12,0.41,0.21,0.2,2.56,0.107058824,0.645333333,0.005298722,0.105021029

56106,PCDHGA10,21.05,32.76,15.4,13.27,9.72,0.44,10.37,0.23,1.87,37.37,60.35,24.81,37.06,21.74,20.01,21.54,9.8,4.77,6.7,7.35,1.89,0.46,0.47,4.07,19.03,1.97,6.39,17.13,4.94,6.79,10.25,4.71,19.87,6.461333333,0.005350738,0.105021029

23596,OPN3,6.75,22.59,18.99,12.5,12.6,81.77,6.08,8.81,11.77,16.64,9.58,20.91,21.52,24,7.05,8.2,16.02,5.75,1.83,1.54,8.52,12.02,17.73,8.34,7.65,11.19,3.58,13.06,6.15,4.93,15.94,6.39,17.98705882,8.308,0.005350738,0.105021029

400322,HERC2P2,28.07,24.44,19.06,74.36,78.54,18.58,26.66,34.01,34.39,25.25,21.46,6.27,7.63,22.25,24.49,25.84,26.82,5.77,11.96,13.2,132.61,12.82,13.3,14.74,5.76,7,17.02,19.68,15.34,11.64,43.99,11.6,29.30117647,22.42866667,0.005350738,0.105021029

79616,CCNJL,3.97,3.35,2.46,13.12,13.52,0.45,0.99,2.05,2.57,2.19,2.98,1.06,0.72,2.22,0.87,3.09,5.66,2.21,0.08,0.02,5.25,3.11,0.09,3.84,0.06,0.3,0.25,0.34,0.6,0,2.23,0.19,3.604117647,1.238,0.005350738,0.105021029

57684,ZBTB26,3.75,4.25,2.22,3.69,3.9,2.74,1.82,5.27,4.46,3.85,4.79,3.2,2.03,3.95,4.17,6.56,3.03,2.4,3.81,1.24,2.69,1.19,0.15,2.66,3.07,1.9,1.25,4.09,1.96,2.37,3.99,3.45,3.745882353,2.414666667,0.005350738,0.105021029

23613,ZMYND8,8.51,8.75,8.08,21.85,25.62,30.01,6.32,12.57,9.84,9.25,16.07,5.56,7.76,14.98,30.62,9.29,11.86,4.38,10.73,3.8,12.59,11.4,2.72,27.77,9.22,6.97,4.71,6.17,4.86,4.34,7.05,3.27,13.93764706,7.998666667,0.005350738,0.105021029

3996,LLGL1,23.02,33.78,22.2,22.18,26.07,34.32,16.28,29.85,25.97,21.28,32.69,26.64,22.07,27.81,28.58,24.95,27.11,18.32,12.41,19.2,19.89,17.12,1.62,27,27.22,9.98,20.75,30.86,20.3,18.25,29.52,16.15,26.16470588,19.23933333,0.005350738,0.105021029

344558,SH3RF3,7.38,7.85,5.3,4.19,11.68,9.92,1.68,3.72,11.65,10.6,6,9.04,5.12,6.11,5.25,6.46,7.15,2.24,0.79,3.09,3.58,9.62,0.25,0.41,7.12,4.31,3.54,8.33,3.03,3.06,7.58,2.83,7.005882353,3.985333333,0.005350738,0.105021029

55293,UEVLD,6.82,5.22,5.89,11.25,10.19,8.25,7.22,7.03,8.02,7.38,4.34,5.87,5.5,6.74,5.25,6.92,10.09,5.08,3.89,4.12,6.29,5.79,1.79,6.41,6.62,6.16,4.59,5.75,6.08,4.64,8.49,4.27,7.175294118,5.331333333,0.005350738,0.105021029

10484,SEC23A,59.24,54.19,56.34,41,41.87,47.43,73.35,54.41,56.68,69.46,38.99,46.64,51.48,62.52,58.41,49.74,63.65,50.23,41.3,50.57,46.86,40.24,21.5,17.84,39.09,38.38,46.7,47.37,52.27,44.26,69.62,40.58,54.43529412,43.12066667,0.005350738,0.105021029

57602,USP36,10.98,5.04,7.12,10.44,12.04,11.99,5.99,12.85,8.9,7.22,7.59,7.31,6.65,12.37,9.81,8.87,10.93,3.74,5.83,6.13,8.17,6.37,3.39,6.84,9.04,6.89,6.21,9.26,6.55,7.67,8.02,5.91,9.182352941,6.668,0.005350738,0.105021029

10644,IGF2BP2,24.67,22.66,21.17,29.84,35.94,23.22,14.74,24.17,19.76,23.42,29.19,20.54,16.75,48.94,24.94,30.74,17.38,10.99,10.25,11.93,31.42,19.85,0.86,17.81,16.04,18.99,16.9,25.46,19.06,13.67,27.32,11.03,25.18058824,16.772,0.005350738,0.105021029

79657,RPAP3,7.6,4.7,6.7,7.58,7.8,8.68,5.65,8.19,8,6.86,5.94,6.08,5.69,5.2,3.97,6.98,11.23,5.81,3.72,5.73,7.43,5.45,3.74,7.02,4.27,6.12,3.9,3.83,5.24,4.09,6.87,4.26,6.873529412,5.165333333,0.005350738,0.105021029

4800,NFYA,9.4,7.38,7.24,9.03,8.11,5.5,4.2,8.5,5.39,9.05,10.48,6.83,6.12,5.88,8.13,7.22,8.51,4.85,4.1,3.74,7.2,6.31,1.98,9.37,4.37,4.67,3.85,9.02,4.49,2.32,8.34,4.43,7.468823529,5.269333333,0.005350738,0.105021029

9987,HNRNPDL,45.49,25.41,38.49,58.84,49.28,42,24.49,51.51,51.17,32.99,40.46,35.37,29.64,59.09,34.66,37.98,63.45,21.01,29.58,31.28,34.02,20.65,11.67,42.26,36.29,29.77,20.81,41.21,31.06,27.72,48.66,25.46,42.37176471,30.09666667,0.005350738,0.105021029

8508,NIPSNAP1,38.98,40.68,36.17,33.83,29.67,27.58,20.65,32.42,25.1,40.48,46.08,43.75,33.98,49.04,30.78,27.07,23.03,46.04,17.44,16.1,35.37,19.23,23.21,29.47,25.76,15.13,19.76,27.11,22.84,16.78,43.84,13.05,34.07588235,24.742,0.005350738,0.105021029

114904,C1QTNF6,13.58,48.22,13.39,19.73,16.53,15.48,4.49,29.09,14.85,26.9,26.64,23.91,10.21,34.15,21.03,15.23,13.86,12.49,11.79,8.5,27.92,2.07,0.81,8.62,14.32,2.71,6.25,20.69,13.4,14.14,17.6,11.81,20.42882353,11.54133333,0.005350738,0.105021029

8841,HDAC3,28.78,27.97,26.96,26.24,26.29,28.94,20.37,30.87,26.79,22.67,30.57,37.03,23.45,30.24,27.1,24.1,23.46,21.17,20.27,20.19,25.09,15.7,6.82,29.57,24.56,21.12,17.44,27.78,22.82,23.75,29.35,21.8,27.16647059,21.82866667,0.005350738,0.105021029

134430,WDR36,5.9,7.1,4.59,9.2,8.96,5.57,4.63,5.7,4.29,6.77,4.11,6.12,5.81,6.4,4.99,4.5,8.25,4.15,4.02,3.93,8.72,4.1,2.28,4.56,6.5,4.21,3.43,5.69,3.8,4.41,5.74,5.54,6.052352941,4.738666667,0.005350738,0.105021029

57472,CNOT6,7.15,4.39,4.96,8.05,8.75,5.77,3.77,6.33,5.36,5.42,3.93,4.52,3.94,5.59,3.97,5.5,8.76,5.01,2.98,2.78,8.2,3,1.52,8.1,3.21,3.72,3.8,4.83,3.98,3.13,5.08,3.33,5.656470588,4.178,0.005350738,0.105021029

5050,PAFAH1B3,13.04,12.47,14.31,23.71,22.64,12.38,2.75,13.85,14.3,13.67,15.39,29.22,17.2,12.94,14.39,22.89,14.56,10.55,3.18,3.69,21.42,6.88,2.21,44.07,11.85,1.98,2.11,6.94,8.14,3.06,25.65,4.61,15.86529412,10.42266667,0.005350738,0.105021029

6881,TAF10,48.91,59.96,47.38,35.25,29.11,57.17,46.27,48.32,28.57,54.37,30.24,63.67,65.63,72.71,48.25,50.74,44.04,70.21,57.87,56.49,66.38,45.18,50.52,88.11,63.46,69.88,61.14,70.53,49.97,52.3,73.92,57.95,48.85823529,62.26066667,0.005350738,0.105021029

84002,B3GNT5,2.68,9.74,2.75,8.05,6.97,8.71,1.06,4.66,1.46,11.44,8.69,4.51,3.29,17.86,9.37,6.74,2.03,1.4,0.79,0.04,7.48,11.87,1.58,1.37,0.64,4.21,0.46,7.49,1.2,0.24,6.06,0.33,6.471176471,3.010666667,0.005350738,0.105021029

388284,C16orf86,0.75,1.71,0.5,1.23,1.24,1.98,0.25,2.75,0.67,2.03,5.83,1.35,0.43,1.63,0.69,0.64,0,1.55,4.54,4.61,1.22,0.28,1.34,13.76,4.63,1.62,2.3,5.22,2.36,4.14,3.86,2.22,1.392941176,3.576666667,0.005350738,0.105021029

2621,GAS6,90.73,23.4,40.75,13.68,17.53,62.86,280.79,32.92,57.25,64.01,120.18,83.79,393.5,244.2,48.01,22.94,59.29,386.51,253.32,897.94,35.46,170.21,23.64,65.33,1558.69,170.55,843.66,276.35,131.51,176.94,128.61,118.85,97.40176471,349.1713333,0.005350738,0.105021029

3914,LAMB3,1.18,5.78,3.8,8.46,3.81,8.33,3.11,45.41,5.93,34.08,0.89,8.64,6.47,4.58,2.69,2.41,2.72,1.85,5.56,1.94,0.29,2.96,2.08,0.44,4.75,2.75,1.19,2.95,0.54,2.19,3.54,3.77,8.722941176,2.453333333,0.005350738,0.105021029

131450,CD200R1,0,0,0,0,0,0,0.04,0,0,0,0.03,0,0,0,0,0,0,0.07,0,0,0,0,0.06,0,0,0,0.09,0.07,0.12,0.07,0.07,0.09,0.004117647,0.042666667,0.005367467,0.105243384

389558,FAM180A,0,0,0.03,0,0,0,2.29,0,0,0,9.93,0.24,0.1,0,0.05,0,0,0.04,11.37,18.75,0,0,0.08,0,4.65,3.16,5.56,0.72,1.57,7.16,0.14,1.94,0.743529412,3.676,0.005423403,0.106233295

8120,AP3B2,0.08,0,0.08,0,0.02,0,0,0,0.11,0,0,0.05,0,0.08,0.02,0.02,0,0.12,0.12,0.11,0,0,0.07,0.27,0.57,0.15,0.02,0.31,0,0.05,0.08,0.21,0.027058824,0.138666667,0.005437507,0.106298313

9348,NDST3,0.76,0.19,1.82,0.12,0.09,0.16,0.04,1.27,0.18,0.16,0.35,0.06,0.04,0.77,0.04,2.06,0.15,0.48,0.08,0.04,0.07,0.02,0,0.01,0.13,0.01,0.09,0.68,0.06,0.03,0.14,0.01,0.485882353,0.123333333,0.00543763,0.106298313

5340,PLG,0.02,0,0,0.02,0,0,0.02,0.02,0,0,0,0,0.15,0.27,0,0,0.03,0.02,0.09,0.18,0,0.05,118.34,0,0.33,0.02,0.02,0.15,0.11,0.09,0.02,0.15,0.031176471,7.971333333,0.005463838,0.106361176

65989,DLK2,1.03,1.53,1.61,0.11,0.11,0.79,0.05,0.68,0.59,0.89,0.63,1.54,0.69,0.27,3.11,0.64,2.02,0.2,0.17,0.22,0.29,0.12,0.1,0.06,0.38,3.43,0.11,0.09,0.3,0.05,0.22,0.28,0.958235294,0.401333333,0.005484812,0.106361176

8528,DDO,0.05,0.1,0,0,0,0,0,0.05,0,0,0.1,0.11,0.06,0.06,0,0,0.06,0.15,1.7,0.1,0.04,0,2.78,0,2.9,1.53,0.39,0.72,0.04,0.83,0,1.8,0.034705882,0.865333333,0.005496465,0.106361176

389856,USP27X,1.28,1.93,1.8,2.55,2.87,3.26,1.06,2.45,2.55,2.02,2.15,2.14,1.6,1.76,2.02,2.52,1.64,1.03,1.51,0.58,1.97,0.93,0.88,4.46,1.63,1.26,1.28,1.81,1.45,1.11,2.31,1.54,2.094117647,1.583333333,0.005497719,0.106361176

10439,OLFM1,0.25,0.17,0.04,0,0.22,0.26,0.53,0.42,0.47,0.13,0.08,0.06,0.23,0.03,0.09,0.2,0.85,0.99,2.43,3.92,0,0.39,0.29,10.61,0.2,2.16,0.34,0.27,0.67,4.43,0.22,0.36,0.237058824,1.818666667,0.005497719,0.106361176

5101,PCDH9,1.42,2.25,5.97,1.28,0.36,2.77,0.45,3.68,0.87,0.47,2.37,1.86,3.09,6.82,3.9,4.52,0.57,0.53,0.82,0.09,0.41,1.38,0.2,0.26,0.47,4.78,0.04,1.77,0.61,0.53,0.93,0.48,2.508823529,0.886666667,0.005502025,0.106361176

286097,MICU3,1.88,1.97,2.14,1.48,1.05,1.76,1.82,2.17,1.54,2.59,2.48,1.25,2.42,2.67,1.34,2.17,3.14,1.11,2.44,1.44,0.73,0.76,0.66,1.72,1.34,0.57,1.52,3.15,1.03,0.88,1.86,1.24,1.992352941,1.363333333,0.005502025,0.106361176

8110,DPF3,2.76,4.24,2.8,0.54,0.79,16.65,1.27,5.89,1.08,2.76,1.69,7.42,5.32,0.69,3.42,1,3.86,0.9,0.03,1.02,1.91,5.18,0.12,0.46,1.04,1.29,0.88,1.81,0.32,0.53,1.69,0.23,3.657647059,1.160666667,0.005502025,0.106361176

4217,MAP3K5,0.55,2.4,0.77,0.39,0.27,1.53,0.41,4.22,1.48,2.71,1.82,1.44,1.69,18.47,0.33,0.3,1.96,5.46,4.26,0.65,5.15,4.37,5.61,1.85,4.37,8.33,1.82,10.51,1,0.86,2.9,3.95,2.396470588,4.072666667,0.005502025,0.106361176

54989,ZNF770,11.06,6.25,8.83,9.89,9.26,8.78,8.53,10.57,10.91,8.58,8.9,7.03,8.09,8.51,10.16,8.91,12.48,8.78,7.5,8.02,7.52,6.06,5.98,10.9,7.43,6.98,8.33,7.63,10.26,8.04,7.36,7.05,9.22,7.856,0.005506333,0.106361176

51275,MAPKAPK5-AS1,4.78,6.7,5.29,7.24,6.2,7.54,3.82,7.4,5.8,6.11,5.91,6.4,6.76,6.15,5.96,5.11,4.67,4.25,5.07,4.67,5.75,4.95,3.01,6.38,4.32,5.7,3.74,7.15,4.42,4.34,4.58,5.82,5.990588235,4.943333333,0.005506333,0.106361176

100132707,PAXIP1-AS2,0.6,3.32,0.67,1.61,1.22,1.09,2.19,1.65,1.41,0.69,3.37,0.35,1.38,2.42,0.56,1.55,1.34,0.82,2.39,2.72,2.55,2.18,2.04,0.83,4.68,1.5,2.98,3.3,3.32,4.2,2.67,4.18,1.495294118,2.690666667,0.005506333,0.106361176

786,CACNG1,0,0,0,0,0,0.05,0,0,0,0,0,0,0,0.67,0,0,0,0.06,0,0,0,0,0,0.07,0,119.03,0.13,0.2,0.41,0.1,0.1,0.07,0.042352941,8.011333333,0.005704857,0.10955387

1470,CST2,0,0,0,0,0,0.5,0,0,0,0,0,0,0,0,0,0,0,0,0.31,0,0,0,0,0.13,7.28,0.13,0.13,0.3,0,0,0.23,21.23,0.029411765,1.982666667,0.005724213,0.10955387

5988,RFPL1,0.84,0.24,0.55,0.26,0.64,0.53,0.16,0,0.58,0.32,0.56,0.12,0.98,0.13,0.3,0.2,0.19,0.21,0.14,0.05,0.14,0.06,0.1,0,0.2,0,0,1.19,0.54,0.09,0.13,0,0.388235294,0.19,0.005735892,0.10955387

23624,CBLC,0,0,0,0,0,0,0,0,0,0.12,0,0,0,0,0,0,0,0.1,0,0,0.18,0,1.96,0,0,0.1,0.05,0,0.09,0,0.04,0.11,0.007058824,0.175333333,0.005745441,0.10955387

389827,TMEM8C,0,0,0,0,0,0,0,0,0,0,0,0,0,1.75,0,0,0,0,0,0.1,0,0,0,0.22,0,301.49,0,0.5,10.9,0.09,0.24,0.22,0.102941176,20.91733333,0.005745441,0.10955387

126567,C2CD4C,1.04,0.17,0.56,0,0,0.07,0,0.21,0.54,0.39,0.41,0.16,0.29,0.16,0.03,0.28,0.7,0.05,0,0,0.02,0.03,0,0.14,0,0.08,0,0.52,0.02,0,0.18,0,0.294705882,0.069333333,0.005748895,0.10955387

414235,PRR26,0,0,0.02,0.03,0.03,0,0,0,0,0,0,0,0,0,0,0,0,0.07,0,0.03,0.31,0.62,0.8,0.06,0,0,0,0.06,0,0.02,0.04,0,0.004705882,0.134,0.0057895,0.10955387

115004,MB21D1,0,0,0,1.51,1.01,0.48,1.35,0.51,2.9,0.16,0.04,0.05,0.1,1.64,0,0.08,0.92,0.31,1.77,1.87,2.62,1.28,0.86,0.58,1.65,0.73,1.07,1.13,1.29,1.46,1.66,1.17,0.632352941,1.296666667,0.005794125,0.10955387

27124,INPP5J,2.15,0.11,0.62,0.69,0.07,0.22,0.21,2.1,3.3,0.08,0.44,0.8,0.24,1.35,0.51,0.52,1.84,0.77,0.06,0.07,0.28,0.08,0.13,0.23,0.18,0.07,0.15,0.2,0.24,0.18,0.54,0.04,0.897058824,0.214666667,0.005807609,0.10955387

3569,IL6,0.87,0.1,1.45,0.66,0.53,0.06,31.7,3.24,0.44,0.08,0.06,0.66,0.08,0,0,0,0.7,2.47,5.41,3.94,14.76,38.85,0.48,0.15,5.15,0.21,13.23,0.11,3.9,10.96,0.38,21.37,2.39,8.091333333,0.005807609,0.10955387

51209,RAB9B,1.1,0.33,1.49,0.56,0.47,0.69,0.59,3.2,2.76,0.74,1.53,2.9,2.3,1.09,0.5,0.23,0.69,1.08,0.07,0.52,0.36,0.23,0.07,0.54,0.77,1.06,0.19,0.19,0.36,0.38,0.97,0.5,1.245294118,0.486,0.005812108,0.10955387

1949,EFNB3,8.59,1.74,3.28,1.33,1.15,2.07,0.05,5.41,6.66,0.29,4.05,7.75,1.9,0.99,4.85,8.59,4.22,1.34,0,1.49,0.47,0,0,7.69,0.97,0.15,0.15,3.29,1.07,0.68,2.87,0.89,3.701176471,1.404,0.005812108,0.10955387

8448,DOC2A,2.2,1.44,1.57,3.41,4.32,2.6,1.83,4.36,2.04,1.5,1.84,2.63,1.44,4.12,1.8,1.75,2.76,1.32,1.55,1.49,1.71,1.6,1.42,2.25,1.42,1.56,1.2,2.03,1.55,1.42,2.15,1.97,2.447647059,1.642666667,0.005812108,0.10955387

9848,MFAP3L,1.11,0.01,0.06,0.63,0.15,0.38,0.76,0.18,0.42,0.05,2.19,0.01,0.05,0.05,0.12,0.02,0.38,2.31,1.1,1.14,0.07,0.21,3.63,5.2,0.36,6.31,1.51,0.03,1.72,2.39,0.47,0.27,0.386470588,1.781333333,0.005812108,0.10955387

83744,ZNF484,1.14,1.31,1.02,1.2,1.08,1.17,1.08,1.27,1.05,0.62,1.03,1.06,0.57,0.92,1.14,2.05,1.75,1,0.66,0.57,1.04,0.51,0.5,1.9,1.11,0.9,0.63,1.02,1,0.96,0.98,0.82,1.144705882,0.906666667,0.005816608,0.10955387

66004,LYNX1,0.55,0.21,1.19,0,0,1.25,4.52,3.16,1.85,0.59,7.6,1.06,1.01,0,3.42,1.9,1.56,17.66,32.94,23.62,0.7,1.39,4.01,0.98,11.33,2.31,4.18,1.14,2.12,12.61,2.7,14.18,1.757058824,8.791333333,0.005821111,0.10955387

400451,FAM174B,0.03,0.05,0.05,22.73,21.62,1.46,0.32,0.22,0.06,0.25,0.2,0.13,0,2.18,0.03,0,0.41,1.75,2.19,1.03,0.26,2.73,3.3,38.81,0.11,5.86,0.38,1.23,1.45,2.26,1.1,0.66,2.925882353,4.208,0.005825615,0.10955387

81928,CABLES2,3.54,3.51,3.13,6.19,4.67,3.55,1.67,4.27,3.57,3.15,5.67,3.79,2.74,7.56,3.25,3.57,3.34,2.44,1.9,1.81,3.8,2.64,3.46,3.95,3.04,1.11,2.37,2.38,2.17,2.37,4.47,1.92,3.951176471,2.655333333,0.005830121,0.10955387

257236,CCDC96,0.84,1.08,0.9,1.25,0.76,2.17,1.3,0.46,1.29,0.75,1.07,1.74,1.01,1.49,0.73,0.67,1.35,0.54,0.87,1.36,0.74,0.73,0.39,1.2,0.59,0.34,0.61,0.51,0.46,0.81,0.99,0.69,1.109411765,0.722,0.005830121,0.10955387

8541,PPFIA3,0.89,1.67,0.55,1,1.65,0.3,0.68,0.84,2.99,1.8,0.55,0.46,3.17,2.24,1.66,0.82,2.55,0.6,0.85,0.2,0.44,0.2,0.79,3.58,0.47,0.63,0.23,1.37,0.4,0.17,0.59,0.18,1.401176471,0.713333333,0.005830121,0.10955387

4326,MMP17,5.3,0.32,1.29,0.35,0.13,0.06,0.32,4.68,0.38,0.35,3.89,0.04,0.22,0.52,0.11,1.23,1.76,1.03,0.14,12.41,3.56,3.79,0.23,143.6,4.78,1.74,2.49,3.1,5.45,5.76,4.48,1.44,1.232352941,12.93333333,0.005830121,0.10955387

57519,STARD9,1.88,2.24,1.19,5.16,4.9,1.42,0.58,2.14,2.32,2.36,2.44,2.77,1.83,1.76,2.51,3.12,1.7,0.51,0.53,1.25,1.32,1.31,0.12,3.61,0.77,0.49,0.55,2.75,0.98,1.32,4.37,1.18,2.371764706,1.404,0.005834629,0.10955387

23390,ZDHHC17,13.83,8.83,6.19,12.12,9.21,6.95,16.19,10.29,11.04,7.75,8.93,6.17,6.02,9.09,6.08,7.03,13.41,4.4,4.32,4.69,4.34,4.11,1.86,9.45,7.02,3.7,6.68,8.77,6.45,9.44,8.72,7.02,9.360588235,6.064666667,0.005834629,0.10955387

23047,PDS5B,5.09,7.43,5.79,6.78,7.31,7.36,3.06,7.3,4.26,6.64,4.96,4.72,3.35,6.15,4.2,5.86,5.38,4.22,3.12,3.58,4.34,2.43,0.96,6.11,5.98,2.65,3.12,5.7,4.05,3.15,6.05,3.79,5.625882353,3.95,0.005834629,0.10955387

271,AMPD2,27.25,40.88,25.62,34.28,27.7,30.37,19.62,30.83,23.42,42.27,33.81,19.38,24.79,40.51,34.05,18.49,26.59,20.4,20.67,22.48,20.35,13.79,9.54,20.4,38.88,11.02,17.3,34.25,18.06,17.29,28.43,23.34,29.40352941,21.08,0.005834629,0.10955387

84140,FAM161A,1.93,2.63,1.72,2.85,2.61,2.18,0.91,2.73,1.38,1.48,2.63,1.58,1.99,1.65,2.22,2.86,2.05,0.97,0.81,1.23,1.46,0.67,0.4,4.7,1.83,0.6,0.85,3.56,0.77,0.92,2,1.25,2.082352941,1.468,0.005834629,0.10955387

5881,RAC3,32.43,15.43,8.68,31.95,22.17,29.31,8.22,18.71,14.16,34.19,18.3,11.92,12.91,26.59,37.53,13.22,15.92,15.44,8.65,1.68,17.11,18.6,2.82,9.21,3.98,3.98,1.59,21.57,5.39,0.26,38.33,4.01,20.68470588,10.17466667,0.005834629,0.10955387

51650,MRPS33,19.36,14.34,16.91,16.83,12.54,27.93,16.04,28.83,14.01,17.7,22.34,20.9,24.13,22.59,12.87,14.54,15.11,25.48,22.19,18.5,28.02,12.98,37.17,48.04,25.06,40.23,22.02,31.49,21.67,18.5,22.85,27.18,18.64529412,26.75866667,0.005834629,0.10955387

201164,PLD6,0.62,0.39,0.36,0.39,1.11,0.66,0.18,0.35,0.13,0.55,0.99,1.19,1.33,0.53,0.3,1.35,0.32,0.81,3.86,0.43,3.28,0.68,0.27,1.37,1.63,2.66,1.09,1.3,0.69,1.06,1.38,0.67,0.632352941,1.412,0.005834629,0.10955387

205860,TRIML2,2.75,0,1.88,10.07,0.86,2.41,0.49,0.13,0.4,0,0.12,0,0,8.81,3.29,2.35,31.55,0,0,0.13,0.38,2.76,0,0,0,0,0,0,0,0.16,0.05,0.07,3.83,0.236666667,0.005850267,0.109741771

30817,ADGRE2,0.02,0.08,0.01,0.01,0.03,0.01,0,0.03,0,0.07,0.05,0,0.02,0.05,0.06,0.01,0,0.03,0.07,0,0,0.01,2.29,3.55,0.96,4.2,0.09,0.52,0.22,0.03,0.1,0.22,0.026470588,0.819333333,0.005864247,0.109898244

7163,TPD52,0.04,0,0.03,0.17,0.29,0,0.64,0.02,0.11,0,0.15,0.02,0,0.56,0.02,0,0.07,2.23,0.81,0.04,4.3,0.11,12.91,0.76,0,1.08,0.04,0.26,0.62,0.18,0.36,0.02,0.124705882,1.581333333,0.005951695,0.110852584

26576,SRPK3,0.07,0,0,0.08,0.12,0.04,0.43,0,0.04,0,0.2,0.05,0.09,0.74,0.13,0.04,0.14,0.59,2.63,0.36,0,0.09,0,0.18,1.83,56.7,1.33,0.39,1.04,0.2,0.66,0.09,0.127647059,4.406,0.005979457,0.110852584

200162,SPAG17,0.01,0.01,0,0,0.01,0,0,0,0,0,0,0.02,0,0.03,0,0,0,0.04,0.84,0.08,0,0.41,0,0,0.15,0.04,0.01,0,0.08,0.02,0.14,0,0.004705882,0.120666667,0.00600653,0.110852584

219731,PROSER2-AS1,0,0,0,0,0,0,0,0,0.03,0,0.05,0,0,0.18,0,0.02,0.03,0.07,0.19,0.05,0.02,0,0.31,0.08,0.09,0,0,0.08,0.06,0.15,0,0.03,0.018235294,0.075333333,0.006021585,0.110852584

6367,CCL22,0.12,0.1,0.09,0.06,0.08,0.04,0.05,0.22,0.17,0.13,0.02,0.04,0,0.03,0.08,0.05,0.03,0,0.02,0.06,0,0.06,0,0.02,0.07,0.06,0,0.07,0,0,0.04,0,0.077058824,0.026666667,0.006037815,0.110852584

779,CACNA1S,0,0,0,0,0,0,0,0,0,0,0,0.01,0,0.65,0,0,0,0.04,0,0,0,0,0,0.01,0.02,29.35,0.01,0.17,1.81,0.02,0,0.01,0.038823529,2.096,0.006048411,0.110852584

3485,IGFBP2,217.14,1541.31,641.47,424.65,521.92,2070.03,9.31,586.32,296.7,529.75,471.83,311.92,8.52,614,1541.98,292.08,144.12,1066.68,125.7,111.46,44.52,8.5,219.71,2.08,9.49,236.82,606.93,392.36,79.75,3.17,147.3,122.2,601.3558824,211.778,0.006085801,0.110852584

55344,PLCXD1,4,13.16,2.62,10.97,11.19,4.96,1.88,2.83,2.23,9.7,5.81,4.73,6.63,15.3,3.75,4.4,3.87,1.91,1.95,1.87,3.03,1.12,3.07,8.33,2.43,2.08,1,14.13,2.88,1.16,6.57,1.38,6.354705882,3.527333333,0.006085801,0.110852584

25956,SEC31B,5.49,4.77,3.65,9.9,7.96,4.29,3.6,7.37,7.29,3.9,6.72,4.22,4.04,9.54,6.6,5.19,5.28,2.6,6.16,2.39,2.42,2.95,1.05,6.91,6.36,1.16,2.46,6.04,2.11,1.76,7.93,2.83,5.871176471,3.675333333,0.006085801,0.110852584

7188,TRAF5,8.14,3.77,8.24,11.39,10.02,3.47,12.1,7.93,27.37,9.84,6.99,5.1,2.44,11.99,11.76,12.58,19.22,10.87,3.76,4.55,5.2,4.64,0.25,6.24,5.94,2.33,4.56,4.41,9.01,3.51,9.77,3.53,10.13823529,5.238,0.006085801,0.110852584

5326,PLAGL2,4.97,4.47,3.81,15.61,17.31,5.57,2.37,5.85,5.11,3.72,5.04,3.95,2.93,12.69,4.48,5.08,4.92,2.38,1.88,2.01,7.74,2.62,0.66,4.44,3.92,2.07,2.5,5.66,3.63,2.94,5.36,2.67,6.345882353,3.365333333,0.006085801,0.110852584

9755,TBKBP1,12.59,9.92,7.72,16.55,21.07,5.59,4.68,8.59,5.09,9.83,7.37,6.16,6.7,10.91,8.89,11.15,5.27,4.31,4.88,6.14,7.01,5.18,0.5,6.69,8.07,3.53,5.06,12.52,5.84,6.11,6.92,4.77,9.298823529,5.835333333,0.006085801,0.110852584

58496,LY6G5B,9.37,3.98,6.7,14.84,12.36,4.58,6.82,9.33,12.06,5.18,5.21,4.17,2.95,15.52,13.75,9.17,12.34,2.13,7.27,3.91,4.6,4.56,2.1,8.75,8.11,2.01,3.84,7.64,3.71,2.91,12.37,2.73,8.725294118,5.109333333,0.006085801,0.110852584

1836,SLC26A2,5.8,2.32,4.07,6.69,5.31,10.66,4.49,5.59,7.5,5.51,12.63,4.59,3.26,19.29,7.2,3.98,6.66,7.32,3.21,2.48,6,11.4,1.14,3.97,2.87,3.67,3.06,2.9,2.95,3,4.92,2.59,6.797058824,4.098666667,0.006085801,0.110852584

84861,KLHL22,10.37,14.58,10.56,10.87,12.54,14.34,7.22,14.64,10.88,11.09,15.74,14.55,10.94,17.24,11.68,12.11,13.45,10.24,12,10.1,9.64,8.42,2.38,20.05,11.66,7.91,9.02,11.16,9.71,10.08,11.97,10.31,12.51764706,10.31,0.006085801,0.110852584

25976,TIPARP,60.6,8.98,18.25,6.25,5.01,11.35,21.64,49.85,18.23,4.91,8.97,13.56,14.28,8.4,17.98,20.04,31.61,4.3,13.49,7.27,7.29,15.78,3.95,4.92,18.73,12.9,7.58,6.13,4.72,3.54,9.13,7.17,18.81823529,8.46,0.006085801,0.110852584

10929,SRSF8,10.22,7.16,10.23,19.29,17.84,13.24,10.27,21.61,13.45,6.95,12.4,15.12,8.67,16.84,10.25,6.6,10.7,9.28,5.89,6.98,12.67,4.72,5.86,19.08,8.57,9.04,6.47,5.75,7.29,7.79,8.92,10.55,12.40235294,8.590666667,0.006085801,0.110852584

6738,TROVE2,6.11,4.95,5.8,9.89,8.99,5.32,6.6,6.66,8.27,7.43,7.8,4.72,3.77,6.45,6.04,7.52,7.48,4.04,3.63,3.49,5.87,6.25,2.74,9.12,5.57,4.86,3.97,6.67,4.38,4.14,6.54,3.28,6.694117647,4.97,0.006085801,0.110852584

29957,SLC25A24,17.49,13.64,12.12,32.08,24.55,14.52,10.1,17.62,13.27,14.6,11.28,13.15,12.65,28.17,11.23,13.24,18.21,6.44,6.68,9.77,14.14,15.05,1.7,15.87,9.27,11.62,7.24,14.53,10.6,8.55,16.77,9.43,16.34823529,10.51066667,0.006085801,0.110852584

53339,BTBD1,30.57,22.96,31.36,42.73,34.99,36.05,29.4,28.52,26.24,35.8,34.11,28.15,36.77,35.51,22.74,26.11,28.22,30.21,18.21,27.17,28.69,31.4,9.31,26.26,18.46,46.73,21.22,24.27,24.12,19.52,23.34,17.57,31.19,24.432,0.006085801,0.110852584

55357,TBC1D2,31.54,9.69,15.94,0.77,1.1,25.15,19.13,20.58,12.74,14.75,19.41,12.85,8.12,33.92,22.89,21.9,30.76,13.67,4.46,5.93,1.46,18.87,1.91,3.22,15.56,11.56,18.67,3.31,5.39,8.36,9.95,4.15,17.72,8.431333333,0.006085801,0.110852584

8731,RNMT,8.03,8.35,9.02,8.99,10.84,10.9,7.05,8.57,7.98,9.77,11.17,9.92,11.44,9.19,11.22,9.33,6.15,5.71,7,5.37,8.13,4.06,2.25,9.68,8.44,10.33,6.01,9.85,7.02,6.25,9.3,7.58,9.289411765,7.132,0.006085801,0.110852584

8697,CDC23,13.29,10.72,10.82,16.95,17.93,12.95,9.3,13.49,13.51,11.36,13.15,10.14,7.84,17.6,10.95,12.73,15.11,9.27,8.15,9.38,16.93,11.43,6.52,14.37,6.85,11.35,7.73,11.58,10.09,8.49,9.87,7.7,12.81411765,9.980666667,0.006085801,0.110852584

84456,L3MBTL3,5.5,8.79,5.53,14.8,10.27,5,3.71,7.14,3.25,8.2,6.22,2.07,2.1,6.8,3.62,8.4,4.2,2.39,1.04,3.21,12.61,9.86,1.85,4.61,2.85,1.4,1.69,3.33,3.19,2.41,3.7,2.83,6.211764706,3.798,0.006085801,0.110852584

11057,ABHD2,11.9,8,8.04,11.61,12.91,12.53,11.07,15.14,9.96,11.05,25.59,30.04,35,15.71,9.32,8.57,13.22,21.02,36.37,12.71,15.57,11.5,15.23,17.21,18.51,22.96,15.85,22.47,16.84,15.64,21.18,16.03,14.68588235,18.606,0.006085801,0.110852584

80700,UBXN6,59.6,61.25,44.13,40.73,46.64,52.62,58.52,66.65,51.94,51.48,56.81,64.6,50.89,47.66,59.67,37.33,42.59,51.28,56.96,94.05,56.79,52.14,25.8,78.01,77.11,66.38,71.2,76.47,55.04,83.42,69.94,67.26,52.53588235,65.45666667,0.006085801,0.110852584

51060,TXNDC12,25.08,11.22,16.68,20.52,22.51,21.48,20.87,18.21,19.51,18.71,27.11,20.59,11.5,26.11,17.79,13.72,20.99,19.64,23,18.63,35.26,23.5,24.39,29.1,22.49,19.08,22.76,22.71,24.52,28.62,21.84,29.58,19.56470588,24.34133333,0.006085801,0.110852584

55281,TMEM140,4.16,5.18,3.59,9.88,15.72,7.79,3.74,7.15,4.65,4.73,9.04,6.67,4.88,7.04,3.25,4.27,6.27,3.9,6.94,8.25,12.52,14.1,11.97,39.25,12.75,7.69,8.24,9.99,4.6,13.3,5.58,16.75,6.353529412,11.722,0.006085801,0.110852584

7538,ZFP36,5.83,9.46,9.02,6.67,9.1,35.05,8.57,14.62,6.6,4.86,10.88,12.79,3.76,15,9.9,6.85,13.08,8.92,47.54,25.12,7.24,16.27,102.9,9.48,16.64,16.14,14.42,19.82,9.51,25.99,8.45,31.48,10.70823529,23.99466667,0.006085801,0.110852584

8804,CREG1,9.49,1.38,7.55,6.78,13.15,17.09,22.35,10.31,12.15,2.33,14.04,9.3,45,40.75,9.16,5.61,14.08,19.87,44.05,22.94,10.75,24.76,84.66,16.57,42.42,59.03,23.1,3.92,9.96,43.48,11.51,88.02,14.14823529,33.66933333,0.006085801,0.110852584

401612,SLC25A53,0.26,0.14,0.32,0.76,0.54,0.15,0,0.68,0.55,0.16,0.65,0.23,0.2,0.31,0.07,0.14,0.2,0.27,0.04,0.05,0.26,0.03,0.02,0.15,0.05,0.11,0.03,0.11,0.17,0.1,0.31,0.08,0.315294118,0.118666667,0.0061475,0.111225598

494514,TYMSOS,0.27,0,0.23,0.47,0.19,0.43,0.1,0.7,0.1,0.12,0,0.21,0.22,1.3,0.42,0.44,0.44,0.38,0.41,0.87,0.6,1.5,0,0.74,2.53,0.4,0.5,1.66,0.65,0.89,0.23,2.23,0.331764706,0.906,0.006152204,0.111225598

399761,BMS1P5,0.99,0.72,0.53,1.85,1.62,0.59,0.3,1.76,1.6,1.22,0.81,0.21,0.77,1.36,1.05,1.18,1.62,0.47,1.05,0.33,0.28,0.28,0.37,0.81,1.11,0.31,0.35,0.72,0.24,0.38,0.73,0.8,1.069411765,0.548666667,0.006161618,0.111225598

151393,RMDN2,2.31,0.55,0.65,0.75,0.55,2.46,1.3,2.73,1.79,1.39,1.91,2.55,0.59,1.8,0.28,0.55,1.78,3.72,1.31,3.62,1.1,2.47,4.72,2.96,3.31,6.06,1.3,1.9,1.5,2.62,1.13,2.98,1.408235294,2.713333333,0.006161618,0.111225598

441376,AARD,0,0,0,0.02,0,0,0,0,0,0,0.87,0.19,0,0,0,0,0,0.39,0.04,0.09,0,0,0,0.19,0,0.5,0.18,0,0.44,4.75,1.64,0.05,0.063529412,0.551333333,0.006165235,0.111225598

25775,C22orf24,0.06,0.05,0.05,0.13,0,0.06,0.19,0.47,0.49,0,0.06,0.14,0.07,0.16,0.14,0.12,0.22,0,0.17,0,0,0,0,0,0,0,0,0.37,0.05,0,0.26,0,0.141764706,0.056666667,0.006168941,0.111225598

58480,RHOU,1.9,2.55,0.54,2.8,18.61,4.86,3.25,2.41,1.32,5.82,4.98,4.23,4.98,1.87,2.3,0.69,1.23,0.38,4.08,1.48,1.45,0.83,3.79,0.09,0.38,0.81,0.06,0.89,2.08,1.95,2.3,0.34,3.784705882,1.394,0.006171039,0.111225598

57717,PCDHB16,1.29,1.15,0.87,0.92,1.25,0.48,0.53,1.66,0.3,1.29,6.55,2.37,2.63,0.08,2.15,0.9,3.63,1.06,0.92,1.46,0.09,0.99,0.06,0.11,0.4,1.21,0.38,0.07,0.34,0.81,0.1,0.41,1.65,0.560666667,0.006175753,0.111225598

26046,LTN1,5.45,4.55,4.66,7.69,5.64,6.31,5.1,6.51,4.49,6.31,5,4.58,5.2,7.4,5.7,4.09,5.87,3.49,4.82,3.81,5.71,4.1,3.35,6.75,4.51,4.79,4.09,5.15,3.66,3.78,5.25,3.27,5.561764706,4.435333333,0.006175753,0.111225598

128854,TSPY26P,4.8,5.94,2.97,8.21,12.66,2.77,1.25,4.38,4.73,4.72,3.86,2.09,0.84,4.93,1.91,3.63,4.77,3.7,4.26,1.88,3.01,0.96,0.24,0.95,3.17,0.34,1.4,2.85,2.4,1.58,4.21,1.91,4.38,2.190666667,0.006180468,0.111225598

100507588,TGFBR3L,2.67,6.9,2.2,0.31,0,5.17,3.69,5.3,1.45,8.58,8.67,0.42,1.48,3.33,6.67,1.43,1.41,0,4.33,3.05,0.06,0.21,0.34,0.49,0.16,1.13,0.47,3.13,0.05,0.27,0.36,2.06,3.510588235,1.074,0.006180468,0.111225598

151613,TTC14,13.65,12.76,14.98,14.13,12.66,8.09,9.49,14.03,13.26,14.29,17.66,10.53,11.65,16.89,13.5,14.81,9.48,8.6,12.23,7.09,7.57,5.79,4.01,16.18,15.5,5.83,9.65,13.65,6.47,6.42,12.74,6.75,13.05058824,9.232,0.006180468,0.111225598

349196,LINC00965,2.05,1.33,2.27,1.5,1.41,1.63,2.91,3.37,2.61,1.25,2.75,0.98,0.4,3.71,1.57,1.59,1.23,5.47,3.66,5.75,1.23,1.09,0.54,1.6,3.85,8.34,6.13,5.38,4.68,8.67,5.18,4.6,1.915294118,4.411333333,0.006180468,0.111225598

132332,TMEM155,0.47,0,0.05,0,0,0,0,0.11,0,0,5.93,0.15,0,0,0,0,0.12,0.14,0.44,2.96,0.03,0.19,0.06,0,0.64,0.28,0.22,0.14,0.74,0.12,0,0.15,0.401764706,0.407333333,0.006199308,0.111461719

100132708,CYP4F30P,0,0.03,0,2.56,3.55,0,0,0,0,0,0.04,0.32,0.33,2.67,0.12,0.13,0.05,0,0,0,0.1,0,0,0,0.04,0,0,0,0,0,0,0,0.576470588,0.009333333,0.006217528,0.111682672

54855,FAM46C,0.01,0,0.01,0.33,1.68,0.02,0.05,0.03,0.01,0,0.01,0.01,0.03,0,0.03,0.26,0,0.34,0.36,0.59,0.04,0.12,0.85,0.03,2.07,0.01,0,0.92,0.12,0.18,0.09,0.23,0.145882353,0.396666667,0.006228304,0.111682672

440119,FZD10-AS1,0.02,0,0,0,0,0,0.02,0,0,0,0,0,0,0,0,0,0,0,0.13,0,0,0,0.08,0,0.03,1.62,0,0.12,0.21,0.02,0.39,0,0.002352941,0.173333333,0.006228787,0.111682672

56651,LINC00470,0,0,0,0.04,0,0,0,0,0,0,0,0,0,0,0,0,0,0,0,0,0,0.05,0.07,0.14,0,0.07,0,0.1,0.07,0,0,0.19,0.002352941,0.046,0.006284348,0.11257531

3311,HSPA7,0,0,0,0,0,0,0,0,0,0,0.03,0,0,0,0,0,0,0,0.05,0.08,0,0,4.54,0,0,0,0,0.1,0,0.11,0.07,0.14,0.001764706,0.339333333,0.006317472,0.113064772

134111,UBE2QL1,0.07,0,0.01,0,0,0,0,0,0,0,0.01,0,0,0,0,0.05,0,0.33,0.79,0.03,0,0,0.25,0,0.27,0.04,0.04,0.01,0.13,0,0.15,0,0.008235294,0.136,0.006352358,0.113584821

3479,IGF1,0.08,0.05,0.79,0.05,0.1,0.6,0.08,0.08,0.14,0.05,0.05,0.18,0.08,0.69,0.06,0.04,0.1,0.34,2,2.37,0.02,4.29,13.34,0.05,2.52,0.17,1.99,0.2,8.1,20.74,0.07,0.52,0.189411765,3.781333333,0.006397603,0.114288979

23145,SSPO,0.39,0.67,0.24,0.45,0.63,0.33,0,0.15,0.14,0.34,0.1,0.17,0.34,0.44,0.08,0.28,0.08,0.04,0.38,0.03,0.07,0.08,0.02,0.02,0.15,0.05,0,1.52,0.19,0.08,0.08,0.04,0.284117647,0.183333333,0.00642687,0.11470669

286319,TUSC1,0,0,0,4.52,7,0,5.62,9.14,9.65,0,0,0,0,8.07,0,0,0.04,5.68,4.07,8.86,6.56,2.44,6.29,10.97,4.93,5.61,5.19,2.39,8.51,11.97,9.49,3.7,2.590588235,6.444,0.00647626,0.115482437

162998,OR7D2,0.57,0.56,0.57,0.8,0.7,0.62,0.13,0.46,0.29,0.42,0.53,0.57,0.91,0.39,0.62,0.74,0.65,0.38,0.21,0.12,0.21,0.12,0.76,0.8,0.66,0.34,0.12,0.39,0.06,0.27,0.26,0.17,0.560588235,0.324666667,0.00649053,0.115631103

150368,FAM109B,7.61,32.97,10.11,0,1.02,15.79,15.36,21.71,5.59,0.08,5.95,0.04,0.04,12.23,0.04,0.28,0.04,11.69,15.38,24.92,16.41,9.92,0.65,9,15.72,7.83,14.15,19.68,21.63,20.17,24.65,21.31,7.58,15.54066667,0.006500353,0.115700354

6857,SYT1,8.05,4.15,4.05,2.42,0.19,1.27,2.13,8.15,0.8,0.66,12.28,0,2.5,1.48,2.48,8.55,7.07,3.68,0,1.33,1.38,1.74,0,0.33,1.71,0.89,0.31,1.34,0.49,0.03,1.05,0.31,3.895882353,0.972666667,0.006524947,0.115854116

2788,GNG7,0.76,0.54,0.61,5.71,2.34,0.81,0.05,1.01,1,0.59,2.37,2.19,1.56,0.32,0.38,0.37,0.46,0.47,0.44,0.13,0.16,0.04,0.29,15.65,0.59,0.15,0.13,0.18,0.09,0.05,0.86,0.47,1.239411765,1.313333333,0.006529872,0.115854116

162967,ZNF320,2.69,2.17,2.49,5.02,5.43,3.29,2.39,3.22,3.01,2.95,3.54,2.42,2.55,3.44,2.07,3.5,4.47,2.28,1.95,2.1,2.01,3.22,0.34,3.17,2.71,3.07,2.08,2.13,1.97,2.17,3.6,2.05,3.214705882,2.323333333,0.006539727,0.115854116

1951,CELSR3,2.21,0.34,0.62,0.16,0.15,0.85,1.41,0.69,1.66,1.33,0.98,0.95,1.96,1.54,1.09,0.35,0.75,0.7,0.64,0.33,0.14,0.36,0.07,0.51,0.43,0.27,0.77,0.98,0.32,0.38,0.35,0.21,1.002352941,0.430666667,0.006539727,0.115854116

84078,KBTBD7,2.81,3.2,2.14,3.81,4.81,3.44,2.5,3.85,4.08,2.83,4.78,2.41,1.54,2.2,2.77,2.91,2.82,2.08,4.06,1.53,2.04,0.91,1.93,5.3,2.64,1.19,2.08,3.64,1.21,2.01,2.11,1.95,3.111764706,2.312,0.006544658,0.115854116

23428,SLC7A8,0.07,0.14,2.26,0.1,1.46,0.03,0.24,0.03,0,6.07,3.15,0.11,1.04,0.95,0.52,2.05,0.06,0.74,18.37,5.25,0.72,0.21,1.19,99.68,5.29,0.81,1.63,2.7,0.94,3.08,5.95,2.69,1.075294118,9.95,0.006544658,0.115854116

64108,RTP4,0,0.42,0,0,0.08,0.43,0,0.08,0,0,0.08,0,0.27,0.1,0,0.15,0,0,0.41,1.28,0,0.44,2.12,36.06,1.1,0,0.17,0,0.27,3.5,1.53,0.88,0.094705882,3.184,0.006567134,0.116146507

154197,PNLDC1,0,0,0.03,0,0,0,0,0,0,0,0.04,0,0,0,0,0,0,0.04,0,0,0,0,0.38,0,0.05,0,0.04,0.04,0,0.15,0.33,0.33,0.004117647,0.090666667,0.006625908,0.117079727

285501,LINC01098,0.07,0,0,0.08,0,0.14,0,0.08,0,0,0.22,0,0.27,0,0,0.36,0,0,0,0,0,0,0,0,0,0,0,0,0,0,0,0,0.071764706,0,0.006636189,0.117118225

170825,GSX2,0.18,0.1,0,0,0,1.57,0,0.07,0,0,0.25,0.14,0,0,0,0,0.08,0,0,0,0,0,0,0,0,0,0,0,0,0,0,0,0.140588235,0,0.006645718,0.117118225

140678,MLLT10P1,0,0,0,0.45,0.31,0,0,0.16,0.17,0,0,0,0,0.57,0,0,0,0.31,0.4,0.16,0.55,0,0,1.19,0.39,0.16,0.16,0.38,0,0.39,0.75,0.51,0.097647059,0.356666667,0.006646114,0.117118225

23120,ATP10B,0,0,0,0,0,0,0,0,0,0.01,0,0,0,0,0,0,0,0.03,0,0,0.02,0.02,0.62,0.03,0,0.03,0,0,0,0,0.01,0,0.000588235,0.050666667,0.006757144,0.118134216

100133036,FAM95B1,0,0,0,0,0,0,0,0.05,0.28,0,0,0,0,0.13,0,0,0.06,0.62,0,0,0.69,0.29,0.55,0.23,1.04,0.05,0,0.17,0.04,0,0.54,0,0.030588235,0.281333333,0.006789909,0.118134216

10716,TBR1,0,0,0.02,0,0,0,0,0,0,0,0,0,0,0,0,0,0,0.02,0,0.05,0,0,0,0,0.09,0.15,0,0.06,0.04,0,0.04,0,0.001176471,0.03,0.006792212,0.118134216

1780,DYNC1I1,4.05,0,0.11,0.1,0.18,4.04,0.93,0.03,0.11,0.06,5.56,0,0,0.03,0.03,0,0.09,6.19,0.42,0.39,1.18,3.83,0.09,0.49,0.83,1.18,1.63,0.45,0.53,0.24,2.77,0.23,0.901176471,1.363333333,0.006845823,0.118134216

10683,DLL3,0.75,0.15,0.22,0.04,0.11,0.37,0.42,0.54,0.54,0.51,0.11,0.71,0.83,0.38,0.64,0.54,0.89,0.19,0.1,0,0.24,0.17,0,18.56,0,0.35,0,0.18,0.32,0.03,0.42,0.13,0.455882353,1.379333333,0.006850946,0.118134216

84766,CRACR2A,0.1,0.05,0.03,0.85,0.03,1.73,0.67,0,0,0.54,0,0,0,0,0.04,0,0.08,0.03,0.02,0.55,2.39,3.25,0,0.04,1.9,1.3,0.95,0.51,0.71,1.29,2.24,0.53,0.242352941,1.047333333,0.006865597,0.118134216

728378,POTEF,0,0.62,0.01,3.08,2.61,0,0,0,0,0,0.32,0.44,0.43,2.43,0.15,1.66,0.69,0.02,0,0,0.06,0.02,0.03,0,0,0,0,0,0,0,0,0,0.731764706,0.008666667,0.006873419,0.118134216

152519,NIPAL1,0.07,0.05,1.23,0.18,0.14,0.55,0.28,0.37,0.5,0.3,0.55,0.04,0,0.8,0.83,0.56,1.08,0.32,0.4,0,0.6,0.2,0.26,0,0.04,0,0,0.06,0,0,0.23,0,0.442941176,0.140666667,0.006881214,0.118134216

114899,C1QTNF3,0.49,0.42,0.28,0.57,0.34,0.34,0.06,0.26,0.44,0.38,0.45,0.57,0.65,0.27,0.27,0.19,0.2,22.39,32.02,5.03,0.36,0.44,0.19,0.79,0.65,0.76,0.28,0.68,31.36,4.64,1.58,0.19,0.363529412,6.757333333,0.006881727,0.118134216

114793,FMNL2,23.37,16.05,8.26,66.34,82.7,16.72,8.46,17.5,7.99,9.65,17.04,16.38,24.27,4.8,9.39,39.25,10.15,9.68,8.48,5.81,10.61,16.75,2.98,3.69,19.97,1.77,4.96,12.79,5.08,7.6,14.3,4.68,22.25411765,8.61,0.006907013,0.118134216

399665,FAM102A,11.21,17.48,15.06,32.55,93.82,17.02,7.69,12.53,13.06,5.3,18.3,24.8,13.88,9.82,20.99,20.89,7.1,8.2,5.83,10.77,3.66,5.23,3.44,28.23,9.61,3.05,11.19,11.32,16.48,12.91,8.74,10.82,20.08823529,9.965333333,0.006907013,0.118134216

80228,ORAI2,9.14,13.27,9.02,21.82,23.37,9.31,3.56,7.82,4.15,9.54,10.71,7.71,7.53,12.01,6.38,5.54,7.04,7.65,4.69,6.98,11.78,7.93,1.08,4.74,7.41,2.95,5.74,5.73,5.16,5.35,6.9,6.35,9.877647059,6.029333333,0.006907013,0.118134216

100505678,STARD4-AS1,9.77,6.48,9.3,2.95,2.55,2.41,4.47,14.74,15.46,3.81,5.31,3.04,2.92,8.94,4.61,8.17,19.62,3.85,3.4,5.27,1.03,0.67,0.2,0.08,3.54,0.9,5.36,2.86,3.67,4.51,6.65,2.09,7.326470588,2.938666667,0.006907013,0.118134216

90488,TMEM263,40.24,48.96,47.91,14.51,14.67,35.22,50.1,42.45,36.42,48.5,31.94,35.51,48.37,38.6,41.29,26.16,35.77,45.59,34.43,31.22,19.71,18.87,18.04,17.47,32.91,18.62,33.27,28.75,29.59,27.63,37.09,30.99,37.44823529,28.27866667,0.006907013,0.118134216

23410,SIRT3,8.66,7.89,6.6,6.27,7.63,12.12,2.97,10.5,9.09,7.51,6.7,9.26,8.18,8.13,8.76,7.99,6.52,5.53,5.98,6.28,5.62,4.23,6.31,11.31,7.88,4.29,5.16,11.26,5.93,6.2,5.75,6.94,7.928235294,6.578,0.006907013,0.118134216

3251,HPRT1,13.89,17.22,27.51,39.62,26.82,26.15,14.81,14.55,11.13,35.71,7.44,33.87,28.84,46.77,9.88,11.53,19.85,13.69,6.63,13.37,26.96,13.62,7.45,9.15,8.88,20.9,12.4,8.9,12.22,10.81,16.63,12,22.68176471,12.90733333,0.006907013,0.118134216

6045,RNF2,6.62,4.47,6.25,8.28,7.69,6.05,4.37,6.01,5.68,5.69,4.71,6.09,5.56,5.13,3.76,5.33,5.67,4.46,3.49,3.91,5.96,3.28,3.26,7.74,4.89,3.29,3.55,3.12,5.93,4.62,5.77,3.95,5.727058824,4.481333333,0.006907013,0.118134216

1829,DSG2,17.28,1.21,6.81,20.77,41.35,1.11,3.62,7.4,26.52,10.43,0.9,0.91,2.35,27.08,17.11,16.93,28.6,14.55,1.94,0.51,0.83,0.73,5.14,4.18,3.26,0.78,3.67,2.41,1.85,1.39,8.16,0.84,13.55176471,3.349333333,0.006907013,0.118134216

84132,USP42,3.91,3.25,3.1,5.91,6.27,4.71,2.08,4.85,4.51,2.91,4.45,3.6,2.34,3.94,3.26,3.93,4.86,2.94,2.58,2.74,3.9,2.32,0.81,4.48,3.14,2.37,2.61,3.56,2.97,2.96,4.27,2.27,3.992941176,2.928,0.006907013,0.118134216

50807,ASAP1,27.63,12.71,28.56,28.76,35.07,32.7,22.63,27.12,43.87,18.16,30.44,17.18,14.36,45.98,22.81,20.8,26.31,15.18,10.85,17.76,22.17,51.81,1.55,18.55,17.59,14.54,13.8,19.08,16.09,17.89,22.76,21.57,26.77,18.746,0.006907013,0.118134216

117143,TADA1,3.55,3.44,3.13,4.72,3.85,4.19,1.85,5.29,4.22,2.96,4.45,4.63,4.55,5.88,5.03,5.07,4.73,3.96,2.75,2.38,3.56,1.81,2.59,5.34,2.69,2.47,2.55,4.2,2.87,2.98,4.82,2.84,4.208235294,3.187333333,0.006907013,0.118134216

52,ACP1,46.22,34.28,43.62,37.21,32.42,36.18,37.09,47.41,39.27,39.3,34.44,41.47,38.48,55.98,43.97,30.31,44.23,29.99,31.13,29.71,39.87,40.83,24.84,32.95,30.59,41.99,27.46,37.13,34.45,25.03,36.47,37.27,40.11058824,33.314,0.006907013,0.118134216

9329,GTF3C4,7.33,6.02,5.44,8.69,8.11,6.37,4.89,8.13,6.82,5.39,7.32,7.45,4.54,10.45,6.94,10.13,9.75,5.35,4.39,4.69,9.9,5.14,1.08,4.84,6.17,5.54,5.12,5.69,5.94,6.78,7.71,4.43,7.280588235,5.518,0.006907013,0.118134216

8450,CUL4B,18.58,41.24,29.83,22.32,19.73,51.39,19.32,24.85,23.3,52.85,50.84,36.23,34.75,27.4,18.24,18.47,24.65,18.85,17.14,32.65,24.18,30.79,6.37,14.45,28.23,10.29,15.95,22.51,12.08,14.48,27.28,15.96,30.23470588,19.414,0.006907013,0.118134216

90313,TP53I13,20.32,16.12,13.71,18.84,19.83,17.52,15.86,25.66,19.26,14.8,14.53,16.6,13.83,38.05,21.09,12.73,21.44,28.76,49.67,32.1,20.71,18.88,10.22,36.68,21.5,16.46,34.18,25.84,20.84,22.26,24.87,20.69,18.83470588,25.57733333,0.006907013,0.118134216

84279,PRADC1,10.88,11.12,11.09,8.49,8.02,19.61,9.24,8.3,6.97,9.74,10.9,9.77,9.36,10.08,10.8,4.56,12.85,20.13,10.68,13.43,13.32,16.14,22.07,16.08,6.91,37.03,8.81,13.35,13.94,8.66,11.78,12.76,10.10470588,15.006,0.006907013,0.118134216

8721,EDF1,170.12,163.68,166.18,180.71,198.67,205.83,164.83,162.51,132.25,176.47,198.7,208.59,188.29,226.75,194.64,220.77,186.52,198,182.21,195.78,251.07,213.71,296.6,231.75,199.7,253.88,188.52,198.61,198.29,183.6,220.27,235.99,185.03,216.532,0.006907013,0.118134216

84875,PARP10,4.56,9.5,1.33,3.59,9.57,11.11,2.65,2.62,2.79,6.67,5.47,4.96,2.53,10.74,4.3,6.38,5.56,3.23,6.56,11.37,5.12,13.09,9.61,24.5,19.49,4.44,6.75,11.9,3.52,11.73,9.44,12.64,5.548823529,10.226,0.006907013,0.118134216

9404,LPXN,2.85,1.34,1.24,2.42,2.08,3.73,9.85,2.61,2.37,1.19,2.47,1.95,3.36,1.02,1.76,2.21,1.79,1.33,2.67,14.34,2.9,25.12,2.84,8.35,6.86,2.55,2.76,3.98,1.62,4.31,2.87,3.21,2.602352941,5.714,0.006907013,0.118134216

64478,CSMD1,0.11,0,0,0,0,0,0.01,0,0,0,0.01,0,0,0,0,0,0,0.01,0.01,0,0.02,0.14,0,0.01,0.01,0,0,0.01,0.03,0,0.01,0.2,0.007647059,0.03,0.006907463,0.118134216

645206,LINC00693,0.04,0.11,0.15,0,0,0.04,0,0,0.05,0,0.41,0,0,0,0.05,0,0,0.19,0.53,0.05,0.13,0,0.43,0.05,0,0.2,0,0.12,0.24,0.08,0.15,0.11,0.05,0.152,0.006921392,0.118134216

93349,SP140L,7.28,6.41,5.17,12.17,14.44,7.36,4.68,6.62,7.65,3.81,3.56,6.84,5.46,9.99,4.96,6.29,11.58,2.08,3.11,3.42,7.59,13.43,5.45,2.27,4.75,5.2,5.17,4.03,4.36,4.36,4.41,4.31,7.31,4.929333333,0.006922882,0.118134216

54602,NDFIP2,13.73,5.34,5.91,9.45,9.58,10.34,20.63,9.07,6.32,16.56,8.3,9.91,10.22,17.84,7.21,5.98,10.25,7.37,8.35,6.18,3.97,3,4.77,7.21,5.41,8.05,16.16,5.41,7.32,8.99,6.65,5.48,10.39058824,6.954666667,0.006922882,0.118134216

387763,C11orf96,0,1.3,0.19,12.38,23.48,3.31,12.26,0.37,1.14,1,0,10.35,1.09,0.51,0.2,0.16,0.07,10.96,5.29,12.93,2.25,22.69,229.26,20.25,6.36,0.98,12.42,1.27,11.53,8.82,0.19,22.35,3.988823529,24.50333333,0.006922882,0.118134216

9770,RASSF2,1.12,0.03,0.16,31.8,25.65,0,0.14,0.09,0.59,0.04,4.38,0.05,0.28,0.15,0.08,0.2,0.44,10.13,0.04,13.21,30.56,4.67,2.48,0.72,0.37,2.13,1.2,3.83,9.41,4.8,0.57,3.02,3.835294118,5.809333333,0.006928035,0.118134216

55313,CPPED1,3.02,0.76,1.47,11.47,8.97,3.04,5.14,2.33,3.35,0.98,4.46,1.95,1.53,12.83,2.63,1.88,2.64,3.35,5.1,5.17,17.2,22.82,4.5,12.32,6.35,10.51,4.38,3.12,3.43,3.23,7.36,2.81,4.026470588,7.443333333,0.006928035,0.118134216

4599,MX1,0.32,0.38,0.37,0.06,0.67,9.35,0.14,0.64,3.12,0.21,0.39,0.3,0.1,2.43,0.26,0.62,0.42,1.38,0.61,0.86,4.87,3.9,8.45,13.93,1.05,0.24,0.37,0.8,1.08,2.27,1.96,1.04,1.163529412,2.854,0.006928035,0.118134216

1139,CHRNA7,0.27,0.12,0.33,0.95,1.64,0.64,0,0.79,0.06,0,0.41,0,0.05,0.36,0.74,0.67,0.58,0.32,0.22,0,0.49,0.57,0,0,0.22,0,0.03,0,0.03,0,0,0,0.447647059,0.125333333,0.00695754,0.11852186

1474,CST6,0,0.1,1.21,0.13,0,0.25,0.95,0.14,0,0,0.13,0,0.16,0,0.61,0,0,2.73,6.25,9.58,0.37,0.92,0,0,1.04,5.85,5.62,0,0.35,0.46,0.22,0.76,0.216470588,2.276666667,0.006962931,0.11852186

170425,LINC00858,0.22,0.11,0,0,0,0,0,0,0,0.46,0.28,0.22,0.3,0.43,0.19,0.41,0.03,0,0,0,0,0,0,0.03,0,0,0,0,0.02,0,0.02,0,0.155882353,0.004666667,0.006996232,0.118984785

6474,SHOX2,0.14,0.02,0.08,0,0,0.05,0,0.08,0,0,18.43,0.08,0.69,0.12,0.08,0,0,0.02,3.94,14.54,0.02,0.14,0,0.03,10.17,1.13,2.58,0.12,5.04,4.98,2.73,11.38,1.162941176,3.788,0.00720944,0.122313496

7102,TSPAN7,0.12,0.07,0,3.26,19.62,0,0.3,0.05,0,0.11,0.08,0.05,0.05,0.16,0.1,0.04,0.05,2,0,0.09,30.52,2.24,3.58,0.14,0.38,159.89,0.14,0.87,0.77,0.07,1.08,0.15,1.415294118,13.46133333,0.007218833,0.122313496

221883,HOXA11-AS,0,0,0,0,0,0,2.71,0,0.11,0,0,0,0.45,0.91,0,0,0,0,4.31,1.24,1.43,0.65,0,0.16,3.8,2.28,3.56,0,0,1.54,1.02,0,0.245882353,1.332666667,0.007234466,0.122313496

57480,PLEKHG1,0.05,0,0.05,6.12,15.11,0.02,0.14,0.04,0.06,0.03,0.53,0.01,0.05,0.08,0.11,0.09,0.06,5.63,0.05,1.01,7.99,3.2,0.61,1.37,0.27,0.03,0.06,1.01,1.26,0.8,0.88,1.62,1.326470588,1.719333333,0.007250921,0.122313496

56143,PCDHA5,0.09,0.02,0.1,0.38,0.13,0.09,0,0.03,0.03,0,0.1,0,0.07,0.04,0.06,0.09,0.19,0,0.05,0.03,0.03,0.11,0,0,0,0.07,0,0,0,0,0.03,0,0.083529412,0.021333333,0.0072859,0.122313496

145270,PRIMA1,0,0.02,0,0,0,0,0,0,0,0,0,0,0,0,0,0,0,0.02,0.02,1.03,0,0,0.04,0,0.05,0,0,0,0,0.07,0.1,0,0.001176471,0.088666667,0.007299097,0.122313496

3752,KCND3,0.3,0,0,0,0,0,0,0,0.19,0,0,0,0,0.11,0.32,0,0.14,1.36,0.08,0.06,0,0.13,1.82,0,0.04,0.12,0.69,0.02,1.1,4.52,0.02,0.13,0.062352941,0.672666667,0.007303589,0.122313496

285025,CCDC141,0,0,0,0,0,0,0,0,0,0.01,0,0,0,0.19,0,0,0,0,0.01,0.01,0,0,0.02,0,0.02,6.51,0.02,0,0.08,0.01,0.01,0,0.011764706,0.446,0.007303928,0.122313496

148213,ZNF681,0.96,0.96,0.7,1.64,1.53,0.39,0.57,1.17,0.8,0.92,1.23,0.56,0.7,1.74,0.66,0.99,0.78,0.68,0.75,0.51,0.64,0.53,0.55,0.78,0.71,0.45,0.57,0.9,0.67,0.64,0.84,0.49,0.958823529,0.647333333,0.007309943,0.122313496

2329,FMO4,0.54,0.42,0.38,0,0.07,6.57,0.33,0.31,0.44,0.31,6.97,1.76,0.64,0,0.41,0.51,0.3,1.97,1.7,0.56,0.29,0.49,1.77,4.13,1.93,0.49,1.01,2.16,1.42,1.84,1.47,2,1.174117647,1.548666667,0.007320701,0.122313496

3691,ITGB4,30.25,0.38,0.99,2.88,1.96,4.66,0.26,25.42,14.41,1.41,15.04,0.92,0.16,0.99,8.22,27.55,2.86,10.88,0.43,0.18,0.79,0.85,0.85,0.19,0.32,0.28,0.27,1.33,0.35,0.18,9.88,0.41,8.138823529,1.812666667,0.007320701,0.122313496

4621,MYH3,0.51,0.06,0.23,0.31,0.61,0.18,0.33,0.23,0.48,0.42,0.09,0.19,0.12,36.87,0.26,0.19,0.33,1.51,3.3,0.22,0.08,0.04,0.57,0.87,0.97,1191.74,1.05,2.89,36.63,1.65,0.9,0.72,2.435882353,82.876,0.007320701,0.122313496

283209,PGM2L1,3.23,8.33,1.56,4.61,5.36,3.48,3.76,6.19,4.19,6.47,2.18,3.23,3.74,3.87,2.14,2.41,3.62,3.09,1.53,1.72,4.16,7.15,0.42,2.71,1.51,1.67,1.86,1.98,1.67,1.18,7.12,1.23,4.021764706,2.6,0.007326083,0.122313496

80237,ELL3,1.06,0.59,1.42,0.91,0.33,1.61,2.17,1.17,1.2,1.21,1.77,1.73,2.06,1.66,1.52,0.83,1.03,2.4,1.81,2.25,1.35,2.19,2.91,2.49,1.67,1.46,1.33,0.89,1.9,1.36,2.17,1.77,1.31,1.863333333,0.007326083,0.122313496

54566,EPB41L4B,1.77,2.19,1.08,0.06,0.04,0.58,1.55,5.38,1.29,1.62,1.87,1.26,0.76,0.05,2.96,2.4,2.58,0.19,0.57,0.19,0.18,0.07,3.35,0.02,0.31,0.46,0.09,0.34,0.45,0.23,0.64,0.09,1.614117647,0.478666667,0.007326083,0.122313496

683,BST1,0.25,5.26,5.89,4.03,1.22,10.82,56.25,11.74,22.17,0.19,3.25,0.17,0.18,8.66,2.13,0.4,0.19,11.51,6.34,17.7,9.58,43.03,2.57,9.23,11.58,2.43,34.07,2.65,17.7,26.32,15.56,40.83,7.811764706,16.74,0.007326083,0.122313496

79789,CLMN,0.4,0.76,1.41,1.64,1.74,1.39,0.04,0.53,1,1.26,1.19,4.72,0.45,1.11,0.92,0.4,0.36,0.35,0.77,0.01,0.5,0.52,1.88,0.85,0.28,0.32,0.06,0.39,0.12,0.13,0.84,0.29,1.136470588,0.487333333,0.007331467,0.122313496

84254,CAMKK1,3.56,4.35,6.88,4.97,6.6,5.06,1.74,8.1,2.97,4.77,2.74,2.81,3.81,4.32,8.88,7.86,1.74,2.06,3.7,5.61,2.01,1.65,0.22,1.62,7.99,1.01,2.24,1.39,2.9,2.49,3.78,3.23,4.774117647,2.793333333,0.007331467,0.122313496

84705,GTPBP3,7.07,5.04,4.79,11.91,9.73,5.99,3.28,7.6,6.7,7.35,6.01,4.59,5.66,8.4,5.83,5.73,10.91,3.88,4.87,3.78,5.6,3.82,2.95,10.41,5.13,3.78,3.56,6.5,3.24,3.6,7.74,4.7,6.858235294,4.904,0.007331467,0.122313496

11073,TOPBP1,7.02,5.49,4.79,9.8,9.25,6.85,4.15,7.54,6.78,6.4,6.82,5.6,4.3,9.77,6.82,6.8,10.36,4.32,2.84,4.96,9.2,4.9,1.52,8.54,4.71,3.92,3.85,6.47,5.82,5.45,6.08,4.25,6.972941176,5.122,0.007331467,0.122313496

57533,TBC1D14,15.9,19.52,12.59,18.58,15.21,26.52,10.82,17.53,14.95,14.53,25.02,18.59,13.82,16.35,12.46,16.13,15.2,13.58,9.05,12.75,11.35,8.84,4.09,52.66,14.25,14.64,10.3,16.28,11.08,12.43,17.17,10.3,16.68941176,14.58466667,0.007331467,0.122313496

6513,SLC2A1,92.23,24.98,71.37,9.95,11.35,50.78,65.84,45.64,99.56,100.77,57.7,63.26,70.61,93.28,97.19,42.17,92.23,107.92,51.13,15.78,9.94,17.87,2.15,8.22,38.09,26.16,49.31,11.6,65.18,14.33,32.22,27.51,64.05352941,31.82733333,0.007331467,0.122313496

200373,CFAP221,0,0,0,0,0,0,0,0,0,0,0,0,0,0.07,0,0,0,0.48,0.92,0.18,0,0.52,0.64,0,0.09,0,0,0,0,0.02,0,0,0.004117647,0.19,0.007336299,0.122313496

100131825,CADM3-AS1,0.18,0.02,0,0,0.05,0,0,0,1.46,0.06,0,0,0,0,0.05,0,0.08,2.22,0,0.14,1.41,0.05,0.08,0.03,0.03,11.89,0.8,1.02,0.22,0.08,0,0.03,0.111764706,1.2,0.007365695,0.122698629

8857,FCGBP,0.04,0,0.01,0,0,0.1,0.03,0,0.01,0.04,0.01,0.08,0.15,0.05,0.02,0.01,0.02,0.09,0.27,0.04,0,0.09,1.92,0.03,0.08,0.08,0.02,0.31,0.1,0.05,0.06,0.04,0.033529412,0.212,0.007471771,0.124359369

81704,DOCK8,0.02,0.01,0,0,0,0,0.02,0,0,0,0.01,0.01,0,0,0.01,0,0.04,0.06,0.53,0,0.05,2.22,2.4,3.84,0,0.25,0,0,0.08,0.17,0,0.23,0.007058824,0.655333333,0.007497742,0.124685143

2306,FOXD2,0.05,0.03,0.03,0.1,0.03,0.63,0,0.1,0.14,0,0.03,0,0.02,0,0.11,0.05,0.04,0,1.83,0.07,0.04,0.02,0.18,0.53,0.64,0.21,0.11,1.44,0.18,0.04,2.79,0.11,0.08,0.546,0.00752704,0.125065645

1638,DCT,0.03,0,0,0,0,0.03,0,0,0,0.45,0.16,0,0.04,0,0.04,0,0,0.51,0.06,0.1,0,0,0,872.08,0.04,0,0.32,0.79,0.17,0.12,0.05,0.42,0.044117647,58.31066667,0.0075345,0.125082977

22875,ENPP4,0.05,0,0.03,1.02,0.16,0,1.28,0,0.12,0,2.13,0.25,0.96,0.06,0.02,0.02,0,1.09,0.51,1.15,3.72,3.66,3.72,0,0.37,7.98,1.62,0.01,0.44,0.93,0.18,0.35,0.358823529,1.715333333,0.007554753,0.12531246

51299,NRN1,2.4,0.03,3.38,0,0.11,3.14,0.23,0.04,0.12,0,0,0.33,22.91,0,0,2.58,6.31,1.55,83.2,59.79,30.28,10.07,0.82,0,41.77,5.6,6.87,0.28,4.79,8.99,1.22,0.08,2.445882353,17.02066667,0.007565853,0.125389868

1641,DCX,0,0,0,0,0,0,0,0,0,0,0,0,0,0.28,0,0,0,0.08,0,0,0,0.01,0,0,0.05,0.11,0,0.05,0.17,0.03,0.01,0,0.016470588,0.034,0.007650553,0.125899404

51233,DRICH1,0.06,0,0.1,0,0,0,0.06,0.06,0,0,0,0,0.21,0,0,0,0,0,0.15,0.3,0.05,0.27,0.11,0.13,0.08,0.06,0,0,0.1,0.26,0,0.07,0.028823529,0.105333333,0.007654536,0.125899404

3127,HLA-DRB5,0,0,0,0,0,0,0,0,0,0,0,0.07,0.08,0,0,0,0,0,0.12,0,0,0,33.58,0,27.11,0.14,0,0,0.06,0.12,0.61,0.08,0.008823529,4.121333333,0.007680493,0.125899404

375513,GUSBP4,1.81,1.66,0.98,0.75,1.55,0.97,0.59,4.08,1.46,0,0,0,0,3.16,1.55,1.44,0.4,0.86,2.28,3.28,0.59,1,1.01,1.75,3.25,3.02,1.56,4.48,3.07,3.87,2.08,3.24,1.2,2.356,0.007694115,0.125899404

56098,PCDHGC4,1.65,0.62,0.31,0.4,0.31,0.24,0.12,0.67,0.31,0.97,0.78,0.15,1.66,0.6,0.14,0.77,0.76,0.39,0.32,0.32,0.03,0.04,0.18,0.14,0.47,0.1,0.03,0.3,0.11,0.25,1.15,0.07,0.615294118,0.26,0.007722145,0.125899404

196047,EMX2OS,0.41,0.43,0.4,0.74,0.87,0.47,0.27,0.45,0.4,0.34,0.29,0.78,0.88,0.78,0.66,0.37,0.46,3.24,0.89,37.85,0.57,0.46,0.4,0.7,0.81,0.51,1.02,0.48,2.98,12.26,0.52,0.52,0.529411765,4.214,0.007722145,0.125899404

83448,PUS7L,0,0,0,1.38,2.52,0,2.63,2.59,1.93,0.28,0,0.78,2.11,2.78,0,0,0,2.76,2.42,1.57,3.36,2.51,1.42,1.92,3.18,1.74,1.78,4.11,2.08,1.85,3.32,1.31,1,2.355333333,0.00772224,0.125899404

388630,TRABD2B,0.02,0,0,0.01,0.04,0,0.11,0,0,0.01,0,0.04,0.05,0.72,0,0,0,0.63,0.35,0.01,0,0.01,0.2,0,0.04,0.06,0.02,0.05,0.67,1.39,0.17,0.75,0.058823529,0.29,0.007724522,0.125899404

414777,HCG18,5.21,2.1,4.25,3.93,3.93,3.21,4.55,4.1,4.11,2.5,3.03,2.85,3.58,3.48,3.19,2.61,5.47,3.59,2.63,2.09,2.42,2.49,1.56,2.72,3.58,2.98,3.58,2.36,3.6,2.95,2.85,2.4,3.652941176,2.786666667,0.007727758,0.125899404

5342,PLGLB2,1.56,1.01,1.45,2.04,1.31,1.18,1.14,0.43,1.92,0.89,0.67,0.4,1.38,1.78,0.84,0.99,1.74,0.41,0.58,0.4,0.51,0.75,88.89,0,1.2,0.32,0.68,0,0.75,1.26,1.13,0,1.219411765,6.458666667,0.007727758,0.125899404

84671,ZNF347,3.16,3.34,2.57,4.76,4.78,3.54,2.62,4.11,3.98,3.21,3.34,3.95,4.34,4.28,3.1,3.28,5.92,2.29,2.29,3.42,2.56,2.6,2.6,4.25,3.31,2.56,3.26,3.92,2.05,3.16,3.74,2.73,3.781176471,2.982666667,0.007733372,0.125899404

84622,ZNF594,2.16,1.44,1.58,1.44,1.14,1.06,1.24,2.34,1.68,1.02,2.34,1.66,1.53,2.13,1.75,2.68,1.33,1.35,1.55,0.52,0.58,0.57,0.17,1.26,1.47,1.17,0.95,2.18,1.01,0.87,1.98,1.24,1.677647059,1.124666667,0.007744608,0.125899404

160897,GPR180,3.02,3.33,3.86,3.14,2.78,2.42,3.86,3.25,2.65,4.48,2.45,2.82,3.54,4.19,3.24,1.82,4.3,3.11,2.86,2.43,2.41,3.33,3.18,3.64,1.81,2.22,1.86,2.26,2.49,1.54,2.6,1.86,3.244117647,2.506666667,0.007744608,0.125899404

645676,ASH1L-AS1,0.99,1.21,1.05,1.52,1.66,1.08,0.51,1.25,0.94,0.43,1.06,1.34,0.64,2.02,1.5,1.48,1.01,0.71,1.43,0.36,0.92,0.49,0.43,0.88,1.55,0.66,0.59,0.95,0.77,0.71,0.96,0.4,1.158235294,0.787333333,0.007750229,0.125899404

55055,ZWILCH,6.12,4.58,4.86,6.17,5.94,7.29,4.31,8.99,7.04,6.32,5.37,4.89,2.95,8.11,5.72,6.15,9.3,4.82,1.73,5.07,9.17,4.54,0.66,3.29,3.12,4.42,3.12,5.88,4.31,3.53,8,4.38,6.124117647,4.402666667,0.007750229,0.125899404

100132352,FRG1HP,3.58,2.2,2.24,4.7,5.38,2.98,6.35,8.39,6.27,0.6,3.07,2.18,1.37,4.38,2.72,3.35,1.45,2.96,6.56,6.51,4.88,3.48,1.61,12.64,7.03,3.85,5.2,7.57,6.2,7.42,6.27,7.52,3.600588235,5.98,0.007755852,0.125899404

90634,N4BP2L1,0.59,0.04,0.16,0.83,4.16,1.14,0.2,1.29,0.39,0,0.42,3.52,0.36,0.73,0.37,1.78,0.3,0.66,1.38,2.16,1.02,0.37,2.98,15.03,2.8,0.8,1.33,1.93,0.75,3.06,1.09,3.59,0.957647059,2.596666667,0.007755852,0.125899404

115908,CTHRC1,26.04,19.49,39.57,0.44,0.84,73.3,30.11,17.28,4.34,56.83,151.16,0.22,2.59,7.32,17.06,6.72,1.7,103.65,31.58,112.87,62.64,104.18,0.22,1.52,176.25,79.49,36.72,180.25,123.08,42.67,297.64,17.15,26.76529412,91.32733333,0.007755852,0.125899404

3123,HLA-DRB1,0,0.05,0,0,0,0,0,0.07,0,2.05,0.06,0.7,3.25,0,0.14,0,0,0.07,2.03,0.07,0,0.07,65.73,0,289.65,0.2,0.54,0.11,0.16,0.8,0.91,1.27,0.371764706,24.10733333,0.007816221,0.125899404

1158,CKM,0,0,0,0,0,0.13,0,0.05,0,0.17,0,0.05,0,11.18,0.11,0.04,0,0.62,0.12,0,0,0.05,0,0.26,0.12,359.95,0.2,0.99,1.64,0.16,0.08,0.26,0.69,24.29666667,0.007822617,0.125899404

90990,KIFC2,9.18,7.71,6.58,21.46,17.93,9.04,5.84,15.9,18.91,9.5,7.07,16.07,10.07,12.59,9.28,13.08,16.89,4.9,7.68,4.06,4.29,11.01,1.69,12.97,11.46,2.39,5.13,9.73,2.37,3.08,18.54,7.79,12.18235294,7.139333333,0.007822687,0.125899404

5089,PBX2,21.21,41.78,21.78,67.99,54.34,29.41,13.22,29.4,22.03,28.8,29.61,25.33,21.27,48.7,21.13,29.55,19.8,12.86,16.75,19.3,34.7,13.9,11.27,27.59,23.51,17.63,11.73,52.36,14.51,13.37,27.71,16.76,30.90294118,20.93,0.007822687,0.125899404

113201,CASC4,47.47,38.35,50.94,22.39,23.43,36.91,51.52,54.67,38.1,49.07,66.84,51.44,60.9,40.19,30.26,28.69,36.2,36.27,21.69,59.37,21.33,22.98,15.74,15.19,35.99,18.47,44.15,32.06,34.24,39.94,35.71,31.98,42.78647059,31.00733333,0.007822687,0.125899404

7581,ZNF33A,4,2.79,4.12,10.18,8.41,4.58,3.2,5.46,5.94,3.92,3.71,3.3,2.9,7.34,4.03,4.61,5.56,3.22,3.78,2.44,3.83,2.07,2.69,6.25,4.55,2.89,1.97,3.85,2.74,2.92,7.33,2.19,4.944117647,3.514666667,0.007822687,0.125899404

1968,EIF2S3,38.48,65.34,40.85,93.71,80.42,53.66,42.7,86.18,82.65,86.04,31,61.15,64.96,95.72,38.14,41.86,33.79,33.16,27.38,32.16,65.22,33.42,24.11,40.8,40.1,45.33,33.34,45.72,31.99,50.79,43.95,42.87,60.97941176,39.356,0.007822687,0.125899404

135293,PM20D2,3.51,1.18,2.07,4.64,2.32,3.09,2.12,7.12,6.07,2.69,6.09,2.41,1.17,4.9,1.96,3.64,5.92,2.83,1.98,0.88,2.31,1.33,2.66,1.07,1.4,5.3,0.77,1.39,1.02,1.11,5.29,1.97,3.582352941,2.087333333,0.007822687,0.125899404

2313,FLI1,9.63,3.2,6.35,88.47,71.75,3.17,5.13,9.17,6.34,2.77,4.35,3.46,2.61,12.21,2.9,3.39,7.87,0.93,1.46,3.37,69.5,17.6,2.92,0.28,2.8,0.47,4.5,0.11,5.44,2.76,2.19,1.62,14.28058824,7.73,0.007822687,0.125899404

80207,OPA3,5.01,4.34,4.77,6.22,5.93,7.01,4.01,5.19,5.84,4.4,4.97,4.52,3.94,7.44,4.64,4.54,7.37,4.08,4.62,3.45,6.15,4.59,2.48,7.96,4.27,4.9,3.11,4.26,3.35,2.96,4.31,3.17,5.302352941,4.244,0.007822687,0.125899404

153769,SH3RF2,1.64,0.35,0.6,1.89,0.43,0.49,3.61,4.98,4.96,0.5,0.61,0.73,1.04,2.97,0.63,0.24,14.59,2.73,0.4,0.05,4.1,0.69,0.32,0,2.25,0.13,0.27,0.02,0.62,0.07,0.21,0.31,2.368235294,0.811333333,0.007822687,0.125899404

23268,DNMBP,10.19,11.03,6.05,14.77,14.39,16.34,10.74,19.88,6.5,8.87,9.68,9.22,9.42,18.9,6.54,9.95,11.14,4.57,4.37,8.26,14.9,17.74,1.68,6.28,7.21,4.06,5.27,9.15,5.07,8.53,11.3,6.58,11.38882353,7.664666667,0.007822687,0.125899404

57590,WDFY1,18.13,13.98,20.19,30.49,23.34,19.22,28.28,21.49,20.69,17.58,17.43,33.83,16.77,17.71,15.79,18.17,24.93,17.47,12.49,17.36,21.45,21.53,6.27,263.7,11.11,8.05,12.7,15.24,16.48,17.77,15.51,14.34,21.06,31.43133333,0.007822687,0.125899404

10519,CIB1,45.17,51.43,40.46,48.26,54,44.75,53.17,52.98,33.65,53.22,39.09,47.74,47.28,40.47,51.78,28.68,34.54,45.16,73.79,59.04,58.62,60.91,156.54,46.86,57.1,45.57,54.28,52.01,35.25,49.9,51.01,60.37,45.09823529,60.42733333,0.007822687,0.125899404

1936,EEF1D,158.82,182.99,154.61,145.11,127.46,184.82,114.82,187.9,132.51,142.03,107.86,215.15,190.88,151.06,164.23,148.9,162.15,145.94,149.43,218.39,248.75,153.72,153.82,205.93,206.48,211.52,163.68,166.04,183.29,257.55,202.36,361.73,157.1352941,201.9086667,0.007822687,0.125899404

84439,HHIPL1,2.94,3.24,2.85,0.18,0.72,2.4,4.91,1.87,1.46,8.89,1.85,1.35,0.16,0.7,1.18,2.59,1.15,9.02,8.74,5.01,3.51,1.68,0.12,2.18,8.52,1.78,9.3,2.81,4.68,3.8,8.37,5.48,2.261176471,5,0.007822687,0.125899404

8714,ABCC3,2.76,0.15,0.85,2.18,0.32,2.1,1.25,1.72,0.71,2.28,11.51,0.18,0.81,1.37,0.27,0.28,2.23,17.35,2.74,13.74,2.37,5.57,22.8,0.02,14.9,1.73,5.32,0.43,1.45,6.01,2.25,2.08,1.821764706,6.584,0.007822687,0.125899404

176,ACAN,0.02,0,0,0.01,0.1,0,0,0,0.14,0.03,0,2.25,1.53,0,0,0,0.51,0,880.68,0.2,0.28,0.27,0,0.06,0.05,0.98,47.2,0,9.22,3.47,3.41,3.56,0.27,63.292,0.007876151,0.12665527

23732,FRRS1L,2.08,2.01,0.87,0,0.07,0.92,0.27,0.92,0,3.03,0.71,5.96,1.02,0,0.15,0.25,0.47,0.2,0.99,0.07,0,0,0,0,0.09,0.07,0.07,0.17,0.23,0,0.44,0,1.101764706,0.155333333,0.007893365,0.126827435

100128553,CTAGE4,0.46,0,0,0,0.16,0.08,0.6,0,0.62,0.11,0.84,0,0.07,0.19,0.03,0.03,0,0.37,0.99,0.08,0,0,3.89,2.53,0.99,0,1.66,0.34,2.15,1.19,1.07,0.88,0.187647059,1.076,0.007940154,0.12747414

64595,TTTY15,0,0.02,0.01,0.03,0.01,0,0,0,0,0,0,0,0,0,0,0,0,0,1.06,0,0,0,0.42,1.27,0,1.07,0.02,1.21,1.26,0,2.07,0.51,0.004117647,0.592666667,0.007948655,0.127505583

440689,HIST2H2BF,0.49,0.4,0,0,0,0.18,1.26,0.37,0.57,0,0.33,0.38,1.07,0,0.19,1.38,0,2.09,0.72,0.36,0.07,0.42,0.51,0.75,2.69,1.14,0.91,0.76,0.75,1.2,0.57,0.38,0.389411765,0.888,0.00798636,0.128005062

118856,MMP21,0.04,0,0,0,0.04,0,0,0,0,0,0,0,0,0,0,0,0,0.08,0.04,0,0.18,0,0,0.05,0.05,0.04,0.09,0,0,0.04,0,0,0.004705882,0.038,0.008057178,0.129034022

5166,PDK4,0.06,0.03,0,12.54,64.48,7.27,0.89,0.73,0.05,1.14,1.42,0.02,0,1.75,0,0.02,0.02,0.19,2.37,10.14,6.28,1.01,16.85,2.52,1.81,6.04,0.49,32.85,0.09,7.7,6.86,4.51,5.318823529,6.647333333,0.008161073,0.12965067

23591,FAM215A,2.97,0.32,1.45,0.94,0.42,0.29,0.32,2.23,0.92,0,0.81,1.26,1.1,0.92,0.59,0.69,0.49,0.32,0.46,0.65,0.28,0.6,0,0.59,0.54,0.22,0.34,0.18,0.18,0.9,0.26,0.35,0.924705882,0.391333333,0.008161073,0.12965067

8123,PWAR5,1.05,0.6,0.68,1.02,0.58,0.56,0.5,0.36,1.05,0.61,1.19,0.43,0.99,1.38,0.93,1.14,0.99,0.58,0.4,0.19,1.11,0.67,0.08,0.36,0.46,1.24,0.24,0.99,0.21,0.45,0.26,0.21,0.827058824,0.496666667,0.008161073,0.12965067

100131320,RAB6C-AS1,1.59,1.57,1.47,1.93,1.71,1.03,1.79,1.36,1.4,1.84,1.82,0.97,1.47,1.42,1.23,0.97,1.88,1.64,1.09,1.06,1.24,1.2,0.91,1.12,0.95,1.15,0.97,1.44,1.2,1.65,1.12,0.99,1.497058824,1.182,0.00816693,0.12965067

284802,FRG1BP,0.24,0.19,0.19,10.69,10.23,0,3.07,11.16,9.75,0,0.16,0.28,0,10.48,0.18,0.16,0.28,2.62,7.9,11.29,4.34,3.48,2.06,15.55,9.5,7.65,6.97,14.99,9.07,9.24,8.17,12.44,3.356470588,8.351333333,0.00816693,0.12965067

83547,RILP,0.81,1.17,1.89,6.12,3.18,1.47,1,0.92,1.04,1.48,5.48,2.19,1.16,2.23,0.63,1.18,1.17,1.92,5.44,3.55,4.37,3.08,10.33,4.01,4.9,2.73,2.04,1.02,1.07,3.55,3.55,3.41,1.948235294,3.664666667,0.008178651,0.12965067

92270,ATP6AP1L,0.92,1,0.35,0.97,0.98,0.4,1.2,0.69,0.71,1.19,2.92,1.54,1.63,2.58,0.97,1.63,0.26,1.53,4.42,1,0.98,1.35,0.2,4.11,3.04,2.74,1.15,4.72,1.94,2.23,2.48,3.03,1.172941176,2.328,0.008184514,0.12965067

84623,KIRREL3,3.88,2.19,6.48,0,0,4.31,5.51,2.71,7.45,13.52,0.98,2.39,9.06,4.33,1.51,1.96,7.2,1.35,0.98,6.06,0.04,0.05,0.06,0.03,0.23,0.88,3.42,0.17,1.7,2.74,0.89,1.51,4.322352941,1.340666667,0.00819038,0.12965067

317649,EIF4E3,0.5,0.2,0.44,2.86,5.04,1.06,1.15,0.91,0.95,0.21,0.39,0.51,0.72,0.77,0.57,1.27,0.81,2.11,1.22,2.88,1.13,1.61,1.27,0.91,0.48,1.42,1.34,0.6,1.93,1.77,1.6,1.26,1.08,1.435333333,0.008196248,0.12965067

1762,DMWD,24.65,21.82,19.77,37.05,35.91,16.7,12.55,24,24,23.17,23.88,19.11,13.48,29,24.85,21.78,26.05,17.46,17.87,19.25,14.55,18.04,2.95,13.11,18.31,14.57,16.93,24.99,15.61,13.8,28.9,14.74,23.39823529,16.73866667,0.008202118,0.12965067

9382,COG1,19.68,13.26,12.86,18.2,20.19,16.65,14.24,23.72,15.26,19.58,12.73,8.63,10.45,19.7,17.51,13.3,19.49,11.38,12.99,10.56,14.61,17.37,4.24,14.88,12.16,9.01,10.85,15.83,9.7,10.56,17.07,8.65,16.20294118,11.99066667,0.008202118,0.12965067

125965,COX6B2,2.82,3.57,3.49,4.18,2.69,1.55,1.57,2.92,2.87,9.06,3.9,2.7,4.1,5.25,2.64,4.72,3,2.18,3.12,2.1,1.85,2.1,2.06,4.43,2.85,1.26,1.58,2.78,2.03,2.76,2.3,1.76,3.59,2.344,0.008202118,0.12965067

160418,TMTC3,6.63,5.72,4.88,5.62,5.25,6.57,8.28,7.55,5.68,9.2,10.21,7.88,7.34,6.01,3.65,5.35,9.12,6.47,4.23,8.98,6.6,8,1.69,2.52,4.83,3.54,4.93,4.81,4.47,4.81,5.64,4.59,6.761176471,5.074,0.008202118,0.12965067

56994,CHPT1,8.81,6.45,9.56,14.43,11.33,24.22,13.36,13.59,8.02,10.8,20.65,11.32,14.72,24.78,8.18,12.74,15.1,18.46,22.37,27.84,12.48,16.46,31.75,64.44,14.93,33.24,17.85,10.68,17.47,14.93,10.76,22.52,13.41529412,22.412,0.008202118,0.12965067

100128191,TMPO-AS1,2.17,1.71,1.34,2.89,2.19,2.19,0.2,1.89,2,1.52,1.46,10.57,5.83,4.51,1.48,2.59,2.66,0.89,0.76,1.38,3.93,0.91,0.57,1.63,1.08,0.54,0.99,1.23,2.44,2.57,1.68,0.8,2.776470588,1.426666667,0.008202118,0.12965067

100130311,C17orf107,0.28,0.19,0.24,0.47,0.85,0.23,2,4.34,1.66,0.37,0.46,0.08,0.06,1.97,0.36,0.23,0.09,2.28,2.97,2.32,0.18,0.14,2.52,0.44,1.48,0.73,3.3,2.4,2.66,1.61,2.78,2.77,0.816470588,1.905333333,0.008202118,0.12965067

54674,LRRN3,0.08,0,0,0,0.02,1.03,4.78,0.05,0.53,0.03,11.5,0,0.05,0,0.02,0,0,4.12,0.07,0.85,0.07,0.02,0.08,1.32,0.33,0.29,0.05,2.78,0.29,0.77,1.69,2.08,1.064117647,0.987333333,0.008271655,0.130643884

408029,C2orf27B,0.38,0.05,0,0,0.09,0.23,0.28,0.22,0.58,0,0,0,0.12,0,0.09,0.08,0.43,0.56,0.22,0.19,0.84,0.32,0,0.11,0.25,0.38,0.1,1.14,0.24,0.54,0.42,0.58,0.15,0.392666667,0.00831358,0.131184616

1285,COL4A3,0.01,0,0,0,0,0.01,0,0,0,0,0,0,0,0,0,0.01,0.01,0.91,0,0,0,0.01,0.12,0,0.02,0.24,0,0.03,0,0.03,0.08,0.19,0.002352941,0.108666667,0.008319353,0.131184616

121214,SDR9C7,0,0,0,0,0,0,0,0.05,0,0,0,0,0,0,0,0,0,0,0.12,0,0,0,0,0.15,0.11,0.05,0.05,0,0.04,0,0.07,0,0.002941176,0.039333333,0.008458503,0.133271001

399948,COLCA1,0.01,0.02,0.03,0.01,0,0.05,0,0.07,0.03,0,0.03,0,0,0.05,0,0.03,0.08,0.04,0.08,0,0.02,0.03,0.13,0.03,0.09,0.07,0.02,0.05,0.04,0.08,0.07,0.09,0.024117647,0.056,0.008480898,0.133515913

155051,CRYGN,0,0,0,0,0,0,0,0,0,0,0.11,0,0,0,0,0,0,0,0.1,0,0,0,0,0.25,0.14,0,0,0.18,0,0.28,0.09,0.25,0.006470588,0.086,0.008489742,0.133547274

4632,MYL1,0,0,0,0,0,0,0,0,0,0.58,0,0,0,17.05,0,0,0,1.33,0,0,0,0,0,0.84,0,592.12,0.6,1.21,6.73,0.32,0.46,0.32,1.037058824,40.262,0.008533754,0.133996483

3240,HP,0,0,0,0,0,0,0,0,0,0,0,0.18,0,13.94,0,0,0,0,0,13.99,0.45,0,35297.97,0,0.86,0.62,0.07,0.1,0,0.05,0,0.07,0.830588235,2354.278667,0.008533754,0.133996483

79369,B3GNT4,0.19,0.39,0.16,0.05,0.05,0.05,0.1,0.05,0,0.12,0.05,0.33,0.35,0.32,0.4,0.24,0,0.05,0,0,0,0.17,0,0.06,0,0.16,0.05,0.04,0.09,0,0.08,0,0.167647059,0.046666667,0.008544613,0.133996483

441432,AQP7P3,0.99,1.26,1.1,9.38,5.43,0.97,0.3,1.27,0.16,3.3,0.72,0,0,0.56,1.34,0.71,1.06,0.3,0.13,0,0.27,1.19,0.54,0,2.11,0.16,0.16,0.13,0.13,0.39,0.12,0.17,1.679411765,0.386666667,0.008561305,0.133996483

643596,RNF224,0.73,0.04,0.25,0.41,0.26,0.38,0.31,0.33,0.28,0.32,0.35,0.63,0.61,0.33,0.41,0.98,0.06,0.05,0.41,0.16,0,0.06,0,0.35,0.4,0.27,0.33,0.13,0.04,0.13,0.17,0.12,0.392941176,0.174666667,0.008597866,0.133996483

100287227,TIPARP-AS1,0.82,0.26,0.56,0.03,0.21,0.39,0.12,1.51,0.36,0.3,0.32,0.23,0.18,0.11,0.58,0.48,0.64,0.12,0.26,0.25,0.19,0.21,0.05,0.17,0.35,0.16,0.26,0.1,0.1,0.05,0.12,0.24,0.417647059,0.175333333,0.008610071,0.133996483

84217,ZMYND12,0.41,0.2,0.12,0.15,0.15,0.81,0,0.41,0,0.64,1.74,0.27,0.21,0,0.1,0.12,0.11,0.44,1.27,0.32,0.24,0.11,0.4,4.39,1.3,0.23,0.38,1.5,0.34,0.84,0.52,0.44,0.32,0.848,0.008622285,0.133996483

79846,CFAP69,0.44,0.3,0.24,0.06,0.26,0.22,0.49,0.8,0.24,0.22,0.39,0.33,1.29,0.43,0.27,0.39,0.3,0.3,1.45,1.71,0.48,0.27,0.18,0.47,3.16,0.58,0.63,0.32,0.6,1.44,0.62,1.98,0.392352941,0.946,0.008628395,0.133996483

79441,HAUS3,1.98,1.97,1.84,4.85,3.94,2.18,1.82,2.4,2.33,2.3,1.85,1.54,2.13,3.32,2.14,2.45,3.89,1.24,2.04,1.54,2.73,1.84,1.13,4.4,1.58,1.47,1.91,2.35,1.51,1.6,1.84,1.36,2.525294118,1.902666667,0.008646738,0.133996483

1181,CLCN2,1.73,1.98,1.68,2.21,1.69,2.04,0.66,2.83,1.28,2.69,2.08,2.45,1.27,3.14,2.42,2.6,1.51,1.4,1.56,1.15,1.15,2,0.4,1.56,1.66,0.66,0.82,3.17,0.74,0.94,2.41,1.22,2.015294118,1.389333333,0.008658978,0.133996483

79577,CDC73,9.96,6.19,6.87,8.52,8.23,8.94,9.85,9.24,8.23,7.57,8.32,6,6.71,9.28,6.36,6.77,11.12,6.81,6.22,5.68,7.19,7.73,2.17,8.3,5.95,8.69,6.25,6.78,6.7,6.2,6.87,5.72,8.127058824,6.484,0.008665101,0.133996483

158219,TTC39B,0.86,1.16,1.26,0.42,0.84,1.16,1.75,1.47,1.44,1.03,1.38,0.6,0.99,1.57,0.93,0.28,0.66,1.21,2.26,1.11,1.38,0.69,1.96,0.45,1.43,1.53,2.59,1.48,2,1.71,3.56,1.49,1.047058824,1.656666667,0.008665101,0.133996483

100131691,MZF1-AS1,2.53,1.5,0.91,0.74,0.78,1.54,0.98,1.35,0.61,1.56,1.88,1.34,1.49,0.59,1,2.25,1.8,0.57,1.89,1.06,0.24,0.37,0.53,0.86,1.27,0.28,0.6,0.77,0.43,1.02,1.01,1,1.344117647,0.793333333,0.008671226,0.133996483

3653,IPW,3.94,2.39,2.76,6.3,3.8,3.85,1.95,2.63,3.15,3.52,5.27,1.88,2.7,6.67,4.39,3.39,5.02,2.85,2.14,1.61,3.71,2.02,0.4,2.61,3.24,4.27,1.33,4,1.88,0.81,2.18,1.96,3.741764706,2.334,0.008671226,0.133996483

55728,N4BP2,2.11,2.5,2.49,2.2,1.75,1.61,0.96,2.3,2.55,1.33,1.93,1.17,0.82,2.82,1.99,1.42,3.2,1.21,0.68,1,1.44,0.35,3.15,1.09,0.97,1.38,0.76,1.57,1.75,1.29,2,0.61,1.95,1.283333333,0.008671226,0.133996483

339448,C1orf174,13.29,11.05,10.71,14.37,16.57,12.66,9.45,16.79,14.8,8.75,11.18,11.58,10.15,13.16,12.52,11.72,14.74,10.85,7.32,13,11.6,7.34,7.2,11.13,12.22,6.86,10.27,8.62,12.66,12.18,10.3,7.33,12.55823529,9.925333333,0.008671226,0.133996483

22850,ADNP2,5.18,3.91,4.42,7.06,7.15,5.72,2.87,6.59,4.77,4.19,4.55,5.38,4.58,7.74,4.85,4.44,5.6,5.72,3.75,3.51,5.61,3.5,2.36,5.25,4.15,3.47,3.98,4.28,4.2,3.65,4.26,4.53,5.235294118,4.148,0.008671226,0.133996483

2048,EPHB2,19.29,22.21,18,13.62,19.07,14.81,4.33,15.3,15.1,22.19,8.54,16.98,21.43,10.56,15.15,21.41,19.21,35.27,0.42,4.47,21.51,47.96,0.29,0.02,2.01,0.59,2.61,3.14,4.31,0.56,15.3,0.09,16.30588235,9.236666667,0.008671226,0.133996483

22944,KIN,2.84,2.11,2.3,2.83,2.61,2.53,1.89,3.09,2.73,2.26,2.46,2.19,2.02,3.03,2.09,2.25,2.58,1.96,1.52,1.29,2.19,1.78,1.02,2.74,2.27,2.31,1.7,1.92,2.13,2.01,3.1,1.98,2.459411765,1.994666667,0.008671226,0.133996483

629,CFB,1.62,7.21,1.56,0,0.09,0.27,11.77,7.08,0.1,1.57,0.03,9.03,1.54,1.99,0.07,0.63,0.24,1.5,30.08,13.08,2.97,0.56,1423.92,0,48.66,0.61,7.54,16.81,5.37,83.81,7.66,72.41,2.635294118,114.332,0.008671226,0.133996483

84059,ADGRV1,0.04,0.12,0,0.06,0.38,0.18,0.22,0.01,0.01,0.02,0.36,0.16,0,0.1,0.04,0.05,0,0.06,0,0,0,0.06,0.01,0.01,0.01,0.06,0,0,0,0,0.01,0.01,0.102941176,0.015333333,0.00868356,0.133996483

120376,COLCA2,0,0,0,0,0,0.07,0,0,0.08,0,0.07,0,0.08,0,0,0.05,0,0.31,0.06,0.3,0.07,0,0.65,0,0.09,0.08,0,0,0.09,0.65,0.17,0,0.020588235,0.164666667,0.008701732,0.133996483

3598,IL13RA2,0,0,0.23,0.06,0,0.05,0.12,0,0,0,0,0.06,40.79,0.14,0,0,0,3.19,15.65,1.06,1.35,0,0,0,2.41,0.6,0.12,0.05,0.15,7.58,0.09,4.33,2.438235294,2.438666667,0.008799507,0.133996483

8552,INE1,2.24,1.77,2.25,4.26,3.91,1.38,0.96,2.36,3.21,1.65,0.99,0.61,0.83,5.07,1.6,1.71,2.89,0.8,1.1,1.54,0.79,1.18,0.57,1.5,2.42,0.33,1.78,1.13,1.01,0.89,1.74,0.53,2.217058824,1.154,0.00884143,0.133996483

284069,FAM171A2,20.68,21.14,11.88,32.09,38.92,10.39,2.04,10.89,7.45,16.02,25.05,18.46,9.67,10.38,7.77,17.8,4.73,8.38,0.69,6.54,10.31,7.5,0.32,8.01,5.63,14.88,2.39,28.16,6.92,2.66,15.48,5.62,15.60941176,8.232666667,0.00884143,0.133996483

100287616,LOXL1-AS1,6.08,4.81,4.23,7.54,6.47,6.38,13.19,17.71,20.37,5.72,3.57,9.69,6.53,9.12,3.45,4.41,19.82,6.81,5.04,8.18,2.64,0.77,0,0.04,4.59,1.95,6.05,6.15,5.05,3.3,8.01,1.81,8.77,4.026,0.00884143,0.133996483

27018,BEX3,127.89,357.62,254.35,390.14,300.47,307.38,83.38,138.1,87.52,258.87,287.14,314.09,288.27,289.54,77.91,128.05,83.65,129.08,84.49,69.48,150.92,107.32,14.68,137.55,158.29,101.47,93.34,128.67,82.69,66.58,177.95,120.66,222.0217647,108.2113333,0.00884143,0.133996483

340526,RGAG4,4.98,9.84,4.9,8.12,3.66,14.34,2.81,5.91,3.62,5.79,8.41,12.87,11.07,8.51,4.68,14.39,4.42,7.99,2.56,4.71,1.73,1.25,0.7,0.9,7.01,1.99,2.49,8.54,2.42,3.84,7.76,9.11,7.548235294,4.2,0.00884143,0.133996483

2004,ELK3,38.36,30.39,21.48,99.64,143.92,25.34,18.45,45.18,22.35,26.73,37.51,26.72,27.72,21.8,23.35,24.33,26.4,14.74,19.54,27.84,55.02,16.41,1.61,8.17,27.32,13.02,11.67,26.64,21.08,21.4,34.17,13.25,38.80411765,20.792,0.00884143,0.133996483

8635,RNASET2,9.37,26.34,28.8,12.33,8.39,26.37,14.24,16.6,12.52,14.3,134.12,26.26,20.46,34.7,16.69,17.86,8.97,8.71,24.86,8.99,6.07,11.58,14.92,7.02,19.17,5.74,16.29,7.67,8.5,12.7,11.06,17.57,25.19529412,12.05666667,0.00884143,0.133996483

100505881,MAGI2-AS3,13.56,14.5,13.84,13.72,13.88,13.68,13.07,11.52,11.62,11.67,16.71,7.03,7.4,21.11,8.51,10.68,13.7,5.33,14.65,9.25,11.85,6.97,1.19,11.65,10.55,10.3,10.83,8.18,9.13,10.67,9.88,12.98,12.71764706,9.560666667,0.00884143,0.133996483

54899,PXK,26.83,22.03,19.61,19.31,25.96,18.21,12.34,19.04,14.31,24.28,16.84,39.51,35.74,13.68,27.51,14.48,12.47,7.71,10.52,18.34,7.43,7.61,4.89,9.94,28.07,7.34,18.46,17.79,21.29,16.52,13.95,17.68,21.30294118,13.836,0.00884143,0.133996483

9639,ARHGEF10,23.45,9.94,19.26,17.21,15.84,9.78,15.17,25.58,19.12,14.5,14.22,10.97,7.44,15.22,14.05,21.06,22.26,8.61,8.13,18.15,12.43,15.79,0.35,11.16,9.72,6.77,13.58,5.49,13.36,17.7,14.39,10.44,16.18058824,11.07133333,0.00884143,0.133996483

10152,ABI2,10.99,13.03,12.32,13.26,14.41,12.39,7.57,13.64,9.79,12.13,13.84,10.27,9.65,14.6,8.67,11.44,10.35,13.18,11.6,10.28,10.3,8.53,2.08,10.26,10.13,7.45,8.59,11.71,7.7,9.44,11.38,8.39,11.66764706,9.401333333,0.00884143,0.133996483

57799,RAB40C,9.41,11.55,7.02,11.65,15.41,7.88,5.15,10.52,7.18,6.93,8.38,6.31,7.37,8.83,9.23,8.31,8.04,5.97,6.62,6.89,8.97,7.78,3.47,5.38,7.6,4.05,6.04,8.68,6.8,7.07,9.27,6.18,8.774705882,6.718,0.00884143,0.133996483

9462,RASAL2,7.14,3.75,4.67,7.45,9.73,4.95,3.35,8.28,4.2,5.93,4.04,4.39,4.26,4.61,4.55,5.25,6,5.33,2.5,3.17,3.28,2.86,0.62,3.82,7.23,7.41,4.19,3.06,3.92,4.03,5.79,2.88,5.444117647,4.006,0.00884143,0.133996483

83737,ITCH,10.44,9.81,10.25,14.97,15.08,11.14,12,11.88,9.54,12.47,10.64,8.79,10,11.45,9.93,8.88,11.13,9.3,9.61,9.32,11.06,10.13,6.2,10.89,7.99,9.56,9.42,11.63,8.7,9.31,10.79,8.55,11.08235294,9.497333333,0.00884143,0.133996483

9805,SCRN1,54.5,39.87,36.65,55.71,33.23,47.85,52.04,48.81,34.5,52.39,54.05,41.71,45.65,47.26,29.16,38.57,63.12,40.01,28.24,32.59,53.15,37.38,0.85,48.56,28.85,24.59,30.13,44.09,40,39.77,40.76,16.4,45.59235294,33.69133333,0.00884143,0.133996483

10098,TSPAN5,20.98,29.7,24.43,24.24,55.03,49.85,29.47,27.81,37.56,42.57,20.43,46.67,36.42,20.25,40.74,22.95,48.66,36.48,11.92,13.19,25.32,14.65,0.65,9.97,28.44,17.63,21.03,42.48,23.31,18.4,56.31,18.71,33.98588235,22.566,0.00884143,0.133996483

440275,EIF2AK4,20.33,22.06,17.97,20.34,17.17,20.22,19.34,24.82,19.17,13.41,20.45,20.2,17.74,25.33,18.85,16.56,17.06,14.73,11.13,11.89,22.75,21.51,2.63,11.18,18.15,15.74,13.56,17.46,10.98,10.05,30.05,12.82,19.47176471,14.97533333,0.00884143,0.133996483

7514,XPO1,34.15,24.5,26.2,51.17,47.7,38.42,28.39,38.13,37.57,33.31,46.42,34.38,32.52,44.63,36.12,41.65,40.83,31.92,25.84,24.08,41.74,34.13,13.76,38.35,32.61,28.9,28.6,30.63,30.37,23.06,40.73,24.17,37.41705882,29.926,0.00884143,0.133996483

10985,GCN1,22.17,14.05,18.63,19.49,22.13,24.19,16.45,19.97,18.92,19.47,21.12,15.3,14.73,23.64,21.82,15.81,27.67,15.05,12.85,14.91,20.04,20.92,6,19.64,14.95,15.46,15.65,18.62,15.51,13.9,20.58,12.55,19.73882353,15.77533333,0.00884143,0.133996483

124045,SPATA33,2.71,2.29,2.9,3.17,3.39,4.46,2.52,4.3,3.07,2.13,2.61,2.11,2.12,5.38,2.42,2.86,6,2.25,1.27,1.94,2.65,2.73,1.54,3.72,2.18,1.91,1.76,3.1,1.9,1.57,4.33,1.39,3.202352941,2.282666667,0.00884143,0.133996483

162989,DEDD2,14.97,9.6,13.36,10.97,12.75,11.72,9.22,15.69,12.85,8.4,9.97,13.28,13.4,11.5,11.08,9.92,11.58,8.35,7.84,9.29,11.78,10.52,19.56,10.32,10.81,8.04,8.78,9.45,8.86,9.84,10.6,9.64,11.78,10.24533333,0.00884143,0.133996483

11270,NRM,16.13,17.94,10.18,16.44,11.63,20.08,3.08,25.25,12.01,16.08,26.43,13.1,3.49,20.91,16.39,20.38,19.39,7.97,3.97,11.46,12.61,3.66,1.44,12.15,6.86,4.23,8.77,19.12,8.95,7.43,21.21,11.69,15.81823529,9.434666667,0.00884143,0.133996483

84266,ALKBH7,18.55,31.02,18.52,14.98,16.36,19.88,9.92,20.66,15.89,21.32,17.06,18.9,21.74,21.29,27.39,16.56,9.3,23.93,32.41,20.99,22.42,15.82,30.42,38.8,15.9,26.74,17.92,22.34,26.81,24.97,22.83,24.88,18.78470588,24.47866667,0.00884143,0.133996483

9352,TXNL1,36.54,27.59,29.1,40.56,32.43,32.89,32.63,34.21,32.94,35.34,25.01,32.82,43.05,27.39,25.63,29.71,31.08,31.72,19.44,36.03,45.66,34.02,48.02,41.19,47.59,40.79,29.57,33.77,38.28,41.92,49.42,39.05,32.28941176,38.43133333,0.00884143,0.133996483

93655,ST7-OT3,0.15,0.24,0.82,0,0.08,0.15,0.08,0.25,0.26,0.1,0.52,0.17,0.28,0.39,0.17,0.22,0.09,0,0.13,0.39,0.14,0,0,0.09,0,0.16,0.08,0.06,0.2,0.14,0,0.09,0.233529412,0.098666667,0.008872573,0.134363981

10633,RASL10A,0.34,0.73,0.59,0.36,1.37,0.29,0.05,0,0.29,0.19,0.1,0.06,0.12,0.33,0.06,0.25,0.31,0.16,0,0,0.24,0.18,0.38,0,0,0.22,0,0.04,0.36,0,0,0,0.32,0.105333333,0.008886022,0.134463176

730013,ABCC6P2,0.42,3.96,1.14,0.89,0.9,2.5,0,0.9,0.29,1.83,2.51,1.17,2.52,2.7,0.37,1.23,0,0.45,0.74,0.41,0.38,0,0.34,0.12,0,0.15,0,4.58,0.26,0,1.02,0.2,1.372352941,0.576666667,0.008941873,0.135098535

441295,OR2A9P,2.1,0,0,1.1,1.29,1.68,2.65,0.95,1.9,0.1,6.74,0.54,1.43,2.19,0,0,0,1.1,2.47,3.28,1.72,0,0.29,15.15,5.5,1.85,5.46,1.36,8.06,4.86,9.33,5.35,1.333529412,4.385333333,0.008941873,0.135098535

23114,NFASC,0.13,0.02,0.08,0,0.02,0.01,0.71,0,0.1,0.08,0.03,2.78,14.21,0.01,0,0,0.02,19.04,4.51,14.06,0,0.07,0.05,0.02,14.33,7.34,10.29,0.03,1.04,7.18,0.12,7.44,1.070588235,5.701333333,0.00896082,0.135279929

3384,ICAM2,0.13,0.05,0.22,383.89,336.81,0,1.89,0,0.08,0.08,0.27,0.08,0.24,0.61,0.08,0.13,0,0.35,1.02,0.35,426.59,269.62,6.24,0,8.1,0.91,1.15,1.04,0.24,4.39,0.17,28.39,42.62117647,49.904,0.009024122,0.13613014

100287765,LINC00630,0.76,0.82,0.24,1.57,0.59,0.71,0.78,0.74,0.64,0.76,0.68,0.89,0.77,0.83,0.62,0.76,0.73,0.33,0.64,0.64,0.53,0.79,0.07,0.25,0.75,0.31,0.47,0.62,0.34,0.76,0.75,0.66,0.758235294,0.527333333,0.009068566,0.136239263

389643,NUGGC,0.33,0.17,0.24,0.35,0.33,0.06,0.23,0.34,0.34,0.23,0.33,0.23,0.37,0.42,0.26,0.13,0.14,0.04,0.21,0.11,0.18,0.03,2.2,0.29,0.17,0.17,0.1,0.2,0.15,0.1,0.16,0.21,0.264705882,0.288,0.009074924,0.136239263

64798,DEPTOR,0.14,0.07,0,0.85,0.12,8.76,1.62,0.13,0.16,0.25,5.23,0.16,0.28,0.41,0.13,0,0.07,3.05,0.25,15.81,0,0,1.7,0.66,4.78,10.61,11.01,1.4,14.6,59.2,0.41,4.74,1.081176471,8.548,0.009074924,0.136239263

200539,ANKRD23,0.95,1.13,0.99,2.02,1.05,1.1,0.81,1.24,1.11,0.91,2.28,1.09,0.84,1.77,0.98,0.98,1.09,0.27,0.65,0.49,0.67,0.57,1.24,8.88,0.87,0.91,0.83,1.33,0.56,0.83,1.02,0.67,1.196470588,1.319333333,0.009132246,0.136239263

440944,THUMPD3-AS1,3.8,1.3,3.75,4.65,4.66,1.89,3.49,3.07,2.8,2.43,3.23,2.05,1.84,5.14,3.58,3.84,2.52,1.92,3.58,1.74,1.93,1.59,1.05,2.77,2.05,1.86,1.9,3.91,1.86,1.43,2.98,1.67,3.178823529,2.149333333,0.009151394,0.136239263

54957,TXNL4B,2.29,2.54,3.45,2.39,3.55,3.38,2.64,4.01,2.64,1.85,3.42,2.7,2.65,2.84,3.06,2.1,3.7,4.08,2.41,3.04,5.75,2.99,6.24,7.62,4.32,4.18,3.46,3.28,3.6,2.74,3.45,2.7,2.894705882,3.990666667,0.009151394,0.136239263

80131,LRRC8E,1.85,2.1,2.01,0.06,0.23,2.3,1.26,4.27,2.92,2.78,1.46,3.07,2.67,2.94,3.73,2.51,2.96,1.88,2.38,1.52,0.56,1.12,1.52,1.03,2.29,0.42,2.01,0.41,0.78,1.75,1.85,1.53,2.301176471,1.403333333,0.009151394,0.136239263

6103,RPGR,1.64,2.46,1.63,3.98,4.52,2.09,0.97,3.92,2.09,2.71,1.75,1.74,1.9,0.98,1.2,1.2,2.55,0.86,1.38,0.66,11.42,2.01,1,1.17,0.9,1.89,0.86,1.15,0.92,1.08,1.76,1.52,2.195882353,1.905333333,0.009151394,0.136239263

55186,SLC25A36,12.75,10.94,13.92,17.45,17.09,13.6,14.81,14.24,14.11,16.66,16.32,15.82,17.02,18.12,17.8,16.25,9.67,8.57,14.11,11.74,7.36,8.01,0.42,31.65,14.81,10.76,10.65,17.63,12.45,12.49,13.26,11.09,15.09235294,12.33333333,0.009157781,0.136239263

64793,CEP85,3.41,3,2.87,3.53,3.22,5.1,2.26,5.09,5.13,4.76,4.29,2.97,3,7.73,3.67,5.13,8.44,3.07,3.56,2.45,4.5,3.28,1.48,4.35,1.43,2.62,2.32,6.17,2.73,2.23,2.24,1.49,4.329411765,2.928,0.009157781,0.136239263

79598,CEP97,2.14,1.8,1.19,1.2,1.27,1.91,1.49,2.68,1.94,1.84,3.14,1.14,1.03,1.96,1.63,1.75,3.26,0.91,1.39,1.29,1.18,1.01,0.38,2.76,1.14,2.17,1.27,1.24,1.42,0.94,1.15,0.86,1.845294118,1.274,0.009157781,0.136239263

84708,LNX1,0.96,1.46,0.28,2.31,0.97,1.18,0.19,1.32,0.52,0.29,0.7,3.14,0.52,0.7,0.39,0.2,1.67,3.21,4.5,7.38,1.11,4.4,0.24,0.62,2.84,4.45,1.44,0.59,1.54,1.8,1.3,1.94,0.988235294,2.490666667,0.009157781,0.136239263

90673,PPP1R3E,1.68,1.43,1.48,2.72,2.65,2.26,1.16,3.01,2.12,1.48,2.13,2.15,1.38,2.1,1.88,4.28,2.08,1,2.22,0.82,0.87,0.56,0.62,3.73,1.1,1.04,0.6,2.86,1.25,1.02,2.35,1.23,2.117058824,1.418,0.00916417,0.136239263

10748,KLRA1P,1.35,1.19,1.54,1.37,1.6,1.29,0.99,2.81,1.62,1.08,1.63,1.01,1.77,2.35,2.65,1.16,1.49,0.39,0.61,0.87,0.67,0.88,0.19,1.65,1.61,0.37,0.64,3.48,0.48,1.12,2.03,0.67,1.582352941,1.044,0.00916417,0.136239263

1820,ARID3A,2.31,6.24,4.13,15.23,13.96,3.26,1.98,2.88,2.27,3.38,3.18,2.2,2.34,19.32,5.39,3.5,1.9,1.57,1.33,2.19,3.1,1.64,1.14,2.14,4.83,1.29,2.14,6.1,1.16,1.58,7.01,2.71,5.498235294,2.662,0.00916417,0.136239263

2342,FNTB,8.66,4.51,5.75,6.84,4.52,3.61,7.19,8.23,5.36,5.02,7.91,4.18,4.77,11.4,5.75,8.89,8.55,5.04,2.98,6.37,5,2.43,0,4.35,5.78,3.14,3.93,4.02,5.85,7.23,4.87,3.59,6.537647059,4.305333333,0.00916417,0.136239263

905,CCNT2,5.07,4.56,4.1,10.2,9.46,4.43,4.22,4.92,5.35,4.16,4.09,3.47,3.31,8.01,4.82,4.63,6.45,3.75,3.95,3.17,4.48,3.4,2.27,6.99,4.4,4.17,3.3,4.48,3.45,2.91,5.36,3.06,5.367647059,3.942666667,0.00916417,0.136239263

9883,POM121,23.16,18.08,19.27,29.96,27.3,19.53,14.1,22.34,19.19,21.63,24.94,16.87,14.16,30.71,20.13,18.69,26.9,12.75,21.92,16.05,17.62,13.44,7.69,25.59,20.37,16.37,12.74,22.64,14.52,16.37,18.56,13.06,21.58588235,16.646,0.00916417,0.136239263

790,CAD,17.55,9.65,12.69,15.21,13.97,17.73,8.53,15.99,16.72,16.72,12.5,11.43,10.15,18.83,17.2,19.93,28.53,10.88,6.34,9.88,17.53,14.28,4.65,12.16,13.07,5.01,7.37,17.22,11.48,8.48,15.58,7.75,15.49,10.77866667,0.00916417,0.136239263

827,CAPN6,0,0,0,0,0,0,0,0,0,0.05,0,0.16,0,0.24,0,0.14,0,0.04,1.57,0.07,0,0,0.23,0.02,0.05,2.13,0,0.07,1.27,0.83,0.07,0,0.034705882,0.423333333,0.00927753,0.137819407

6820,SULT2B1,0,0.05,0,0,0.07,0.24,0,0.14,0.07,0,0.06,0,0.15,0.24,0,0,0,0.2,0.11,0.27,0.12,0.07,0,0.29,0.25,0.14,0.35,0.27,0.11,0,0.63,0,0.06,0.187333333,0.009290995,0.13791432

5455,POU3F3,0.05,0,0,0,0,0,0,0,0,0,0,0.06,0,0,0,0,0,17.7,0.14,0,0,0,0,0,0,0.28,2.24,0.26,0.04,0.09,0.04,0,0.006470588,1.386,0.009417718,0.139688981

4499,MT1M,0.53,0.43,0.15,0.37,0,1.73,0.57,0.4,0.62,0.46,0,1.83,2.58,0.47,0,0.88,0,0,0.33,4.79,0.17,0.64,3004.14,0.63,7.93,7.11,0.6,1.73,1.12,4.95,1.85,12.31,0.648235294,203.22,0.009549173,0.141216965

5239,PGM5,0,0,0,0,0,0,0.06,0.03,0,0.08,0.02,0.54,6.71,0.5,0,0.05,0,0.57,0.24,0.05,0.02,0,1.17,0.05,0.03,2.34,0.02,0.54,0.98,0.4,0.33,10.71,0.47,1.163333333,0.00957323,0.141216965

5896,RAG1,0.24,0.55,0.28,1.15,0.53,0.77,0.1,0.3,0.34,0.35,0.34,0.51,0.31,0.39,0.43,0.32,0.46,0.26,0.06,0.12,0.35,0.8,0.04,0.95,0.16,0.12,0.04,0.27,0.15,0.08,0.4,0.08,0.433529412,0.258666667,0.009655343,0.141216965

7373,COL14A1,1.02,0.01,2.67,0.11,0.04,6.38,2.38,0.01,0.01,0.09,20.3,43.69,0.91,10.42,3.34,0.83,0.03,59.91,34.85,25.35,0.25,0.03,0.36,0.05,171.27,2.76,21.17,30.99,85.75,55.31,6.11,123.76,5.425882353,41.19466667,0.009655343,0.141216965

115362,GBP5,0.61,0.72,0.53,0.59,1.15,0.55,0.58,0.23,0.13,0.46,1.47,0.67,1.2,0.42,0.48,0.68,0.15,0.48,0.32,0.4,0.37,0.28,1.05,0.33,0.37,0.18,0.26,0.24,0.21,0.19,0.52,0.32,0.624705882,0.368,0.009668655,0.141216965

5158,PDE6B,1.04,0.3,0.25,1.73,3.7,0.58,0.42,0.93,0.64,0.51,1.25,0.24,0.29,1.53,0.25,1.75,0.82,1.02,0.4,0.43,1.18,0.22,0.2,0.19,0.11,0.24,0.11,1.09,0.44,0.17,0.46,0.08,0.954705882,0.422666667,0.009668655,0.141216965

65243,ZFP69B,2.97,1.47,2.48,0.65,0.62,1.37,2.07,1.95,3.01,1.63,2.36,0.8,1.07,0.62,1.67,1.18,2.83,1.65,0.23,0.55,0.72,1.04,0.21,0.64,0.87,1.56,0.69,0.77,1.88,0.92,1.18,0.77,1.691176471,0.912,0.009668655,0.141216965

51804,SIX4,17.71,9.29,12.96,1.68,1.64,10.72,4.18,21.06,6.01,11.22,5.62,12.53,19.93,3.72,9.13,24.06,5.27,7.65,9.33,2.59,1.58,1.49,0.66,1.11,4.3,5.63,2.97,12.39,5.62,1.98,9.16,2.97,10.39588235,4.628666667,0.009675315,0.141216965

23161,SNX13,6.96,5.91,6.86,7.55,7.91,6.76,6.27,7.49,5.47,6.02,8.65,6.59,6.59,6.52,4.85,6.97,6.82,4.7,6.19,5.03,5.61,4.03,2.56,6.52,7.61,5.93,5.27,6.2,4.81,6.34,7.25,6.48,6.717058824,5.635333333,0.009675315,0.141216965

55632,G2E3,2.41,2.75,2.82,3.7,2.9,2.88,2.36,2.56,2.95,3.5,3.25,1.84,2.56,4.86,2.53,3.49,4.28,1.96,2.22,1.87,4.04,2.13,0.7,3.09,2.73,1.75,1.89,3.27,1.84,1.92,2.84,1.71,3.037647059,2.264,0.009675315,0.141216965

7552,ZNF711,3.63,5.45,3.03,3.78,3.88,0.39,0.02,2.85,2.95,1.95,0.82,0.22,0.24,2.01,3.24,2.9,2.54,3.85,0.41,0.04,1.04,0.14,0.23,0.06,0.26,1.58,0.31,0.61,1.93,0.05,2.72,0.02,2.347058824,0.883333333,0.009681976,0.141216965

23042,PDXDC1,32.72,34.04,38.75,24.26,26.84,24.7,29.61,33.79,31.27,42.61,22.37,20.52,21.63,37.43,37.03,22.23,37.46,24.26,25.86,20.33,25.9,20.92,25.41,57.64,21.87,18.12,22.11,23.23,19.98,17.88,33.56,19.96,30.42705882,25.13533333,0.009681976,0.141216965

84803,GPAT3,2.04,1.47,1.95,0.88,0.06,2.01,0.03,2.87,0.58,1.56,2.21,1.78,2.47,2.03,0.63,1.13,2.51,0.15,0.44,0.12,1.53,1.06,1.17,0.92,2.41,1.01,0.03,0.25,0.07,0.28,0.48,0.17,1.541764706,0.672666667,0.009681976,0.141216965

200765,TIGD1,2.94,2.05,2.75,1.7,0.96,1.39,1.91,3.26,2.35,1.9,2.66,2.44,1.59,3.27,3.06,3.69,2.27,2.52,1.54,0.75,1.91,0.21,0.4,1.56,2.25,1.69,0.88,5.1,1.16,0.74,2.49,1.03,2.364117647,1.615333333,0.009681976,0.141216965

23370,ARHGEF18,12.4,10.32,9,9.51,11.81,13.81,5.32,11,12.22,8.87,15.6,9.15,8.26,20.29,12.75,8.61,16.92,10.41,4.5,6.72,8.88,7.54,3.59,21.33,9.89,5.94,9.68,8.95,7.84,6.97,9.15,7.34,11.52,8.582,0.009681976,0.141216965

643155,SMIM15,16.95,12.72,14.57,16.57,11.56,17.89,14.81,14.91,13.8,12.95,14.8,15.68,15.09,16.78,15.49,11.75,20.17,10.7,9.54,15.18,13.53,10.94,14.59,14.72,12.76,9.76,12.72,13.3,14.37,16.16,12.47,13.67,15.08764706,12.96066667,0.009681976,0.141216965

2622,GAS8,3.68,1.19,2.95,6.46,5.97,2.57,1.27,3.03,0.21,2.05,5.34,0.39,0.3,3.65,3.6,4.63,3.57,3.66,8.32,4.58,6.56,4.71,2.15,21.46,6.9,2.84,4.1,4.36,3.26,4.2,5.34,4.9,2.991764706,5.822666667,0.009681976,0.141216965

11092,C9orf9,2.25,2.52,0.92,2.48,2.74,2.4,1.94,3.98,1.99,2.92,4.16,1.74,2.9,2.42,1.78,3.31,0.53,4.35,6.31,3.96,1.64,2.83,0.2,7.35,6.38,3.82,2.66,4.86,3.3,3.31,6.13,2.54,2.410588235,3.976,0.009681976,0.141216965

343637,RSPO4,0,0,0,0,0,0.06,0,0,0,0,0,0,0,0.88,0.03,0.03,0.04,0,0.05,1.27,0,0.03,0,0.03,0.08,0.51,0.13,0.43,0.05,0.05,0.07,0,0.061176471,0.18,0.009683301,0.141216965

53632,PRKAG3,0,0,0,0,0,0,0,0,0,0.04,0,0,0,0,0,0,0,0.1,0,0.03,0,0,0,0.04,0,34.05,0,0,0.32,0.03,0,0.04,0.002352941,2.307333333,0.009768306,0.141216965

3214,HOXB4,0.14,0,0,16.1,18.84,0,0.04,0,0,0.09,0,0,4.33,8.44,0,0,0,4.81,0,2.74,12.74,2.5,0.35,3.07,0.34,1.52,0.08,42.11,6.13,2.31,9.84,0.69,2.822352941,5.948666667,0.009802754,0.141216965

79057,PRRG3,0,0,0,0,0,0,0,0,0,0,0,0,0,0,0.07,0,0,0.06,0,0.19,0,0,0,0,0,0.06,0,0,0.21,0.16,0.05,0.27,0.004117647,0.066666667,0.00981481,0.141216965

100499227,USP2-AS1,0.06,0,0.06,0,0,0,0.21,0,0.08,0,0,0,0,0,0,0.06,0.16,0.07,0.18,0.28,0.06,0,0,0,0.18,0.65,0.15,0.06,0.24,0.06,0.06,0.08,0.037058824,0.138,0.009914224,0.141216965

6335,SCN9A,4.46,0.58,3.51,7.61,4.97,3.6,21.45,1.27,8.96,0.41,0.06,0.5,1.03,2.25,2.23,2.16,6.33,1.64,0.02,0.36,1.06,0.18,0.4,0.09,1.15,0.59,4.41,0.82,3.25,0.93,0.62,0.92,4.198823529,1.096,0.009972716,0.141216965

1290,COL5A2,628.85,662.39,852.76,40.13,60.19,209.97,570.31,553.01,684.52,324.33,779.55,356.8,148.19,208.77,406.7,253.59,510.36,321.02,321.56,221.15,64.64,106.67,1.88,18.23,108.38,223.86,289.78,272.76,338.78,242.91,212.84,152.7,426.4952941,193.144,0.009972716,0.141216965

149478,BTBD19,13.89,15.78,13.1,13.94,10.48,2.66,11.2,19.6,11.76,10.82,4.25,3.58,4.27,9.11,3.6,7.85,10.52,3.18,5.9,4.21,6.22,4.13,0.63,2.63,14.18,1.16,6.04,3.69,4.98,6.73,7.84,4.33,9.788823529,5.056666667,0.009972716,0.141216965

8564,KMO,1.82,5.61,3.91,3.52,3.44,22.83,1.47,1.87,3.25,4.49,3.3,5.79,5.74,5.82,1.69,2.14,4.31,1.57,0.4,0.25,2.05,2.97,8.87,2.45,2.3,2.63,0.65,3.46,1.59,1.22,4.23,1.41,4.764705882,2.403333333,0.009972716,0.141216965

196,AHR,56.97,19.15,64.5,33.77,35.63,21.82,25.41,61.89,49.68,19.18,5.49,15.88,5.05,14.42,10.05,46.88,51.08,24.1,19.03,25.27,8.61,5.38,4.56,2.1,20.42,7,32.66,6.91,6.92,9.28,13.41,26.56,31.57941176,14.14733333,0.009972716,0.141216965

81618,ITM2C,99.9,113.26,153.37,77.91,47,76.01,49.26,115.67,69.75,124.82,277.64,151.81,94.06,171.95,89.12,76.96,73.07,93.89,79.94,25.4,12.06,1.91,9.95,124.66,34.41,46.62,54,98,80.71,17.28,136.03,67.54,109.5035294,58.82666667,0.009972716,0.141216965

57449,PLEKHG5,15.03,12.7,9.24,51.02,41.13,7.74,10.04,19.79,10.12,17.53,32.1,10.64,13.64,12.32,11.99,24.91,15.46,11.79,2.79,4.2,13.41,21.77,0.51,1.35,15.64,23.87,5.93,3.92,8.92,1.43,14.9,4.55,18.55294118,8.998666667,0.009972716,0.141216965

10449,ACAA2,22.38,22.5,17.62,27.29,28.44,30.31,25.57,26.03,26.14,24.19,39.47,25.4,23.52,16.88,14.65,14.72,32.07,31.79,17.74,35.09,29.36,26.47,81.71,44.77,29.25,46.05,24.65,31.92,27.16,38.02,30.23,19.12,24.54,34.222,0.009972716,0.141216965

7072,TIA1,18.4,22.81,17.08,33.75,28.78,16.13,8.28,21.03,15.78,20.32,21.12,13.03,12.15,26.2,15.99,18.86,13.75,10.96,9.92,8.24,15.75,10.75,4.69,31.44,19.27,7.46,7.52,19.81,8.36,6.7,26.58,8.98,19.02705882,13.09533333,0.009972716,0.141216965

9697,TRAM2,182.12,50.83,30.23,37.23,40.72,107.44,57.87,136.5,92.02,61.09,80.08,192.45,66.65,59.8,87.43,62.51,63.91,51.48,48.36,38.09,39.18,80.01,4.09,14.18,53.08,38.99,69.57,27.48,32.46,32.63,82.37,73.37,82.87529412,45.68933333,0.009972716,0.141216965

114883,OSBPL9,24.85,11.62,38.61,27.39,25.69,19.99,15.43,23.02,42,22.12,30.06,16.8,9.19,25.07,30.3,20.47,27.72,17.12,8.99,9.02,27.55,13.38,14.95,87.87,11.53,8.24,14.63,17.22,14.37,17.1,24.26,11.72,24.13705882,19.86333333,0.009972716,0.141216965

11180,WDR6,58.17,56.88,46.84,76.01,79.85,50.25,30.83,67.95,50.11,53.45,56.95,50.17,33.28,77.43,43.46,54.14,50.4,44.57,39.39,29.38,54.41,26.61,12.23,35.63,74.07,24.91,28.75,75.42,23.81,33.7,63.19,23.16,55.06882353,39.282,0.009972716,0.141216965

22823,MTF2,4.47,3.3,3.88,10.35,10.18,3.57,1.82,3.7,3.63,3.29,4.59,3,2.54,9.06,2.31,4.23,5.66,3.51,1.99,2.03,5.77,2.83,1.2,4.61,2.44,2.28,2.14,3.74,2.67,1.47,3.41,1.57,4.681176471,2.777333333,0.009972716,0.141216965

84450,ZNF512,15.01,12.74,11.87,19.7,21.19,15.33,8.2,17.5,13.01,11.91,20.81,10.81,9.68,14.31,10.82,14.02,12.78,11.42,8.4,7.36,10.49,8.13,1.53,16.87,13.23,11.5,6.77,15.25,9.62,6.43,18.36,7.48,14.09941176,10.18933333,0.009972716,0.141216965

55028,C17orf80,13.67,7.3,6.4,9.74,10.47,8.96,4.86,13.89,9.49,6.25,9.29,6.61,4.93,7.28,7.17,7.82,12.08,5.98,5.62,6.03,7.76,3.68,1.84,9.85,5.42,5.48,4.83,8.21,6.3,5.54,9.92,6.09,8.600588235,6.17,0.009972716,0.141216965

29894,CPSF1,32.86,31.26,25.53,43.65,39.79,27.85,18.78,35.2,32.27,30.58,32.87,20.93,17.3,28.42,31.37,28.34,31.67,12.43,15.05,19.73,36.1,27.76,5.5,29.89,23.6,11.64,11.79,33.1,14.56,16.96,36.63,14.8,29.92176471,20.636,0.009972716,0.141216965

57666,FBRSL1,9.09,7.7,7.72,16.12,14.77,5.66,3.73,10.33,7.73,8.54,6.3,5.36,4.69,9.43,10.63,12.18,8.12,4.47,6.66,5.98,6.42,4.51,3.53,12.12,6.96,4.63,5.79,8.33,4.42,5.4,7.03,4.98,8.711764706,6.082,0.009972716,0.141216965

8906,AP1G2,8.91,11.77,10.73,14.01,15.05,15.89,3.67,13.27,12.3,11,7.14,9.27,6.01,25.44,9.94,11.88,8.01,2.82,10.42,3.39,8.24,5.79,5.93,20.57,7.36,1.18,2.18,18.95,4.45,4.64,9.58,8.26,11.42882353,7.584,0.009972716,0.141216965

10735,STAG2,12.83,31.63,17.46,21.96,22.02,35.8,10.53,16.62,12.11,31.53,16.45,26.81,21.39,13.88,11.46,17.11,11.19,8.85,12.87,15.08,16.13,7.84,4.75,20.83,12.78,11.3,10.79,15.36,9.85,13.39,17.36,15.09,19.45764706,12.818,0.009972716,0.141216965

10776,ARPP19,29.95,22.1,20.12,56.59,46.99,31.02,22.02,31.61,29.02,28,46.89,24.11,18.78,31.48,15.87,21.16,30.13,25.78,16.71,16.77,34.99,27.84,6.45,56.17,13.71,15.01,18.49,21.1,18.05,16.7,24.73,15.98,29.75529412,21.89866667,0.009972716,0.141216965

27304,MOCS3,5.62,3.95,4.48,7.32,8.11,6.62,3.88,6.14,4.71,3.82,5.27,4.76,4.73,6.53,4.8,4.19,5.8,4.05,3.83,3.66,4.44,3.22,1.93,8.14,4.61,4.32,3.97,4.96,4.45,4.67,4.62,3.51,5.337058824,4.292,0.009972716,0.141216965

7994,KAT6A,6.15,7.24,5.77,12.16,12.58,9.01,3.2,9.31,6.69,6.4,7.87,5.84,4.84,7.39,6.1,8.58,7.1,3.82,4.15,4.77,8.77,3.88,2.96,9.58,5.66,3.83,4.2,8.37,4.66,5.39,6.97,4.47,7.425294118,5.432,0.009972716,0.141216965

79813,EHMT1,14.6,11.93,10.65,20.97,21.12,14.87,7.26,14.39,11.95,11.36,14.7,11.9,7.59,16.81,13.82,16.47,15.37,10.57,6.77,10.43,18.7,13,3.22,12.75,8.96,7.53,9.41,14.25,10.55,9.62,13.94,8.58,13.86823529,10.552,0.009972716,0.141216965

7486,WRN,4.2,2.54,3.64,5.39,5.09,4.79,3.17,5.28,4.88,3.56,3.63,3.07,2.35,4.54,4.81,3.76,5.3,1.68,4.35,2.52,4.92,2.95,1.01,1.91,3.18,1.89,2.18,3.81,3.24,2.88,5.35,2.51,4.117647059,2.958666667,0.009972716,0.141216965

8621,CDK13,10.2,6.84,7.78,12.07,10.13,9.12,7.87,9.62,9.23,6.96,8.29,8.5,7.58,9.85,7.98,8.07,10.02,6.91,6,6.43,10.09,7.24,3.38,10.91,7.27,5.5,7.64,7.44,8.14,7.81,8.68,5.96,8.83,7.293333333,0.009972716,0.141216965

55668,GPATCH2L,6.68,5.01,4.95,9.13,9.24,5.59,4.72,5.15,6.12,6.27,4.26,3.79,4.61,7.12,6.02,6.85,6.8,5.14,5.42,2.6,7.6,6.54,1.6,4.23,4.41,5.66,3.58,4.43,3.22,3.67,5.65,2.85,6.018235294,4.44,0.009972716,0.141216965

8518,IKBKAP,10.15,7.9,7.71,10.76,9.52,11.24,9.17,11.8,9.22,11.38,10.5,7.15,8.26,10.37,10.08,13.47,9.64,6.08,7.8,7.36,9.6,10.55,3.93,8.78,10.28,7.42,6.64,10.2,7.08,7.81,10.21,7.16,9.901176471,8.06,0.009972716,0.141216965

6574,SLC20A1,82.73,21.76,73.43,36.97,28.8,37.38,69.97,107.36,46.06,71.4,18.4,30.56,70.37,46.78,36.02,49.26,97.9,49.46,9.78,20.24,34.88,47.01,5.06,10.3,59.67,48.48,44.16,9.97,39.15,19.8,22.63,15.22,54.42058824,29.054,0.009972716,0.141216965

55571,CNOT11,15.78,13.77,16.78,23.19,21.08,21.04,13.08,19.93,17.51,14.55,18.3,14.83,11.41,22.59,14.63,13.21,18.84,13.29,11.53,11.18,20.54,14.96,11.19,19.38,12.75,16.92,10.91,13.83,10.79,10.31,17.94,11.28,17.08941176,13.78666667,0.009972716,0.141216965

23468,CBX5,13.79,12.97,9.22,13.36,12.02,17.76,6.59,17.64,11.68,12.16,12.01,9.63,9.27,18.03,12.56,16.26,18.45,9.95,12.69,9.18,13.96,6.84,3.16,6.51,8.67,8.48,7.23,14.04,11.03,7.65,13.88,8.98,13.14117647,9.483333333,0.009972716,0.141216965

8309,ACOX2,0.25,6.44,1.52,0.39,1.13,1.9,0.69,0.38,0.07,5.15,0.89,5.55,8.44,1.41,0.78,0.31,0.08,3.69,0.77,4.83,2.52,1.82,1.91,0.11,10.5,2,5.25,9.63,4.93,8.33,7.37,7.49,2.081176471,4.743333333,0.009972716,0.141216965

84527,ZNF559,1.54,1.39,0.85,4.89,5.01,1.69,3.04,4.47,2.34,0.72,1.02,0.03,0,3.79,1.19,1.25,1.43,2.95,3.99,2.28,2.37,2.88,1.38,5.35,4.38,4.01,2.12,3.09,3.06,4.25,4.19,5.1,2.038235294,3.426666667,0.009972716,0.141216965

50813,COPS7A,36.2,29.04,27.19,19.87,26.33,46.14,46.35,44.83,29.77,31.07,38.51,36.09,46.29,38.76,30.6,30.19,37.81,47.01,46.96,46.45,27.88,44.41,21.16,52.26,49.44,70.94,46.74,37.15,32.64,38.49,50.04,42.09,35.00235294,43.57733333,0.009972716,0.141216965

22837,COBLL1,0.92,0.11,0.47,1.08,0.36,0.21,29.39,1.69,4.71,0.38,1.77,0.34,0.91,3.33,1.65,1.12,4.76,4.41,10.18,1.95,8.78,3.02,3.44,2.26,0.75,3.26,5.34,0.4,2.01,5.8,2.67,4.08,3.129411765,3.89,0.009972716,0.141216965

84836,ABHD14B,19.61,15.5,13.11,10.81,15.95,33.01,21.38,25.16,12.25,16.26,29.26,13.56,31.66,18.8,16.65,15.4,10.04,12.27,36.02,28.73,18.46,17.02,22.06,70.9,36.26,24.32,18.51,40.58,21.74,25.08,26.57,28.39,18.73,28.46066667,0.009972716,0.141216965

353322,ANKRD37,3.25,4.58,3.72,5.25,3.92,5.49,46.28,3.67,6.35,4.76,2.34,5.79,5.64,5.53,3.78,3.5,2.65,6.37,15.93,6.74,2.19,5.98,11.08,11.49,5.41,3.73,14.31,3.99,16.91,21.13,5.36,11.94,6.852941176,9.504,0.009972716,0.141216965

4208,MEF2C,0.74,0.2,1.43,23.08,19.1,1.56,0.47,0.93,0.19,0.87,3.95,0.21,0.17,2.62,2.06,1.57,0.67,3.63,5.46,1.44,4.2,1.73,0.22,17.01,1.96,165.56,2.4,1.9,3.86,1.71,5.36,4.65,3.518823529,14.73933333,0.009972716,0.141216965

388963,C2orf81,1.07,1.36,0.84,0.28,0.1,1.02,0.66,1.29,0.49,0.76,2.07,1.53,1.34,0.3,1.56,1.17,0.53,2.41,2.28,4.47,0.62,0.08,0.06,5.07,3.17,0.58,1.92,3.71,1.94,3.56,2.86,2.88,0.962941176,2.374,0.009972716,0.141216965

126321,MFSD12,17.85,17.63,18.33,17.78,18.57,34.22,19.85,13.91,17.5,27.35,23.61,16.68,18.02,39.13,20.07,12.41,25.95,17.92,12.03,10.68,14.95,22.37,12.55,1702.48,15.71,17.01,14.02,15.95,10.85,11.47,20.08,12.02,21.10941176,127.3393333,0.009972716,0.141216965

6092,ROBO2,0.3,0.08,0.01,0.03,0.01,0.01,0.17,0,0.01,0.09,0.14,0.02,0.03,0.31,0.01,0,0,1.34,0.08,0.47,0.06,0.05,0.02,0.47,0.25,0.39,0,1.15,0.1,0.16,1.25,0.02,0.071764706,0.387333333,0.009977207,0.141216965

53832,IL20RA,0,0,0,0,0,0.06,0,0,0.07,0,0,0.02,0.02,0.03,0,0.12,0,0.27,0,0.26,0.04,0,0.26,0,0.03,4.84,0.02,0.02,2.77,1.66,0.27,0,0.018823529,0.696,0.010034346,0.141922649

6703,SPRR2D,0,0,0,0,0,0,0,0,0.13,0,0.12,0,0,0,0,0,0,0.24,0,0,0,0,0,0,0.16,0.26,0.13,0.1,0.1,0.21,0.2,0,0.014705882,0.093333333,0.010056581,0.14213398

91828,EXOC3L4,0,0,0,0,0,0,0,0,0,0,0,0,0,0.04,0,0.06,0,0,0,0,0,0.03,2.06,0,0.04,0.31,0,0,0.08,0.13,0.05,0.67,0.005882353,0.224666667,0.010066739,0.142174457

220136,CFAP53,0.32,0,0.03,0,0.13,0.16,0.13,0.36,0.19,0,0.08,0.14,0.1,0,0.05,0.2,0.1,0.26,0.55,0.22,0.23,0.1,0.23,0.57,0.16,0.27,0.18,0.36,0.14,0.04,0.28,0.05,0.117058824,0.242666667,0.010094182,0.142458805

56977,STOX2,0.06,0,0.01,0,0.05,0,0.73,0.05,0.02,0,0.67,0,0.02,0,0.07,0,0.06,0.68,0.04,0.03,0.03,0.19,1.4,0.5,0.06,0.21,0.05,0.17,0.1,0.23,0.17,0,0.102352941,0.257333333,0.010126753,0.142662119

124961,ZFP3,0,0.02,0.78,2.77,1.76,0.43,0.02,0,0.2,0.02,1.55,0,0,1.69,0.79,0.59,0.9,1.74,1.67,1.49,1.1,0.91,0.33,0.96,1.28,1.54,1.54,1.14,1.32,1.16,1.85,1.5,0.677647059,1.302,0.010128713,0.142662119

203102,ADAM32,0.08,0.02,0.11,0.06,0.17,0.1,0.25,0.21,0.34,0.1,0.03,0.06,0.17,0.04,0.16,0.21,0.07,0.06,0.68,0.52,0.15,0.48,0,0.13,0.5,0.18,0.45,0.28,0.31,0.76,0.39,0.1,0.128235294,0.332666667,0.010163301,0.142662119

1565,CYP2D6,0.75,0.27,0.6,0.98,2.23,0.43,0.42,0.74,1.24,0.73,0.52,1.03,1.19,1.5,0.62,1.07,0.79,0.31,0.16,0.21,0.16,0.37,16.34,1.82,0.48,0.16,0.06,0.9,0.16,0.39,0.84,0.12,0.888823529,1.498666667,0.010163301,0.142662119

147804,TPM3P9,1.6,1.42,1.54,2.48,1.86,1.86,1.16,2.48,1.96,1.83,1.78,1.1,1.17,2.05,2.46,2.33,2.66,0.58,1.57,1.16,1.89,0.85,1.05,2.21,1.17,1.16,0.73,1.99,1.61,0.86,1.64,1.42,1.867058824,1.326,0.010177153,0.142662119

54933,RHBDL2,0.04,0.04,0.17,0.32,0.09,0.23,0,0.18,0.09,0.19,0.26,0.33,0.25,0.11,0.05,0.41,0.11,0.66,1.33,0.56,0.18,1.15,0,0.21,0.86,0.13,0.55,0.15,0.36,0.26,0.42,0.19,0.168823529,0.467333333,0.010184082,0.142662119

9662,CEP135,1.76,1.35,1.12,4.43,4.16,2.02,1.12,2.79,1.82,1.38,1.33,1.71,2.1,3.29,1.11,1.82,3.8,1.1,0.59,1.25,2.41,1.15,0.5,1.48,2.07,0.76,1.25,0.98,1.57,1.31,2.58,0.98,2.182941176,1.332,0.010204883,0.142662119

2310,FOXO3B,2.11,2.8,2.08,2.04,2.2,2.29,0.97,2.38,1.9,1.55,3.69,2.15,1.9,2.44,2.19,1.94,2.86,1.85,1.32,1.55,2.12,0.62,0.02,2.05,2.12,0.8,0.95,2.99,2.15,1.25,1.82,1.97,2.205294118,1.572,0.010204883,0.142662119

692312,PPAN-P2RY11,3.79,1.01,2.76,5.47,5.88,5.85,3.48,2.39,3.7,3.39,3.64,6.24,3.64,5.07,3.78,1.35,2.8,2.36,3.38,1.64,2.28,2.24,1.68,4.32,2.11,3.38,1.68,4.11,2,2.67,1.26,3.2,3.778823529,2.554,0.010211821,0.142662119

9044,BTAF1,9.67,8.83,11.56,11.36,10.16,9.23,7.07,11.36,8.82,14.31,10,6.81,7.07,17.62,12.43,15.56,9.96,5.08,22.66,5.8,5.08,4.62,3.92,10.11,8.09,4.89,7.09,22.4,5.85,5.65,10.03,6.9,10.69529412,8.544666667,0.010211821,0.142662119

54991,C1orf159,4.26,3.25,3.75,5.59,6.25,5.55,2.69,4.99,5.25,3.09,5.25,3.96,2.23,8.35,4.78,3.62,6.81,2.4,2.86,3.95,3.83,2.69,1.46,6.76,3.45,1.39,3.49,3.96,2.43,2.56,4.85,2.77,4.686470588,3.256666667,0.010211821,0.142662119

11201,POLI,5.74,5.8,3.71,6.16,5.64,7.29,4.66,7.11,5.03,5.47,4.03,3.47,2.79,4.78,3.91,4.23,3.69,3.02,4.12,2.75,3.04,3.04,0.75,6.01,4.89,2.6,2.89,4.91,3.36,3.25,5.9,3.29,4.912352941,3.588,0.010225704,0.142662119

55761,TTC17,19.19,18.27,15.12,23.48,23.96,16.92,13.33,18.27,17.31,21.51,11.12,16.15,14.56,20.89,14.67,15.56,15.65,11.9,15.53,12.49,14.57,18.28,11.17,13,13.9,9.05,11.13,34.61,12.96,12.83,18.68,12.04,17.40941176,14.80933333,0.010225704,0.142662119

54841,BIVM,10.37,7.3,8.08,9.87,10.8,11.69,6.53,12.67,9.43,7.89,12.63,9.91,8.76,9.78,7.03,9.41,8.85,6.93,6.69,9.6,4.75,5.11,3.21,10.19,6.93,8.01,7.74,7.68,8.68,7.85,11.28,7.71,9.470588235,7.490666667,0.010225704,0.142662119

1676,DFFA,12.3,11.09,10.97,16.23,17.9,13.85,8.34,12.49,10.66,13.52,12.13,11.94,15.29,13.18,8.75,10.38,15.11,9.89,8.02,9.02,18.5,13.13,3.21,10.85,10.25,10.01,7.37,11.71,10.25,8.25,13.08,9.38,12.59588235,10.19466667,0.010225704,0.142662119

25841,ABTB2,0.77,0.43,0.69,1.18,0.42,1.74,1.59,0.98,0.71,0.3,1.4,1.58,2.76,3.08,0.61,0.7,0.42,3.01,4.3,2.73,0.11,0.37,2.51,16.41,3.2,5.63,2.8,3.05,2.64,4.08,3.28,0.18,1.138823529,3.62,0.010225704,0.142662119

54847,SIDT1,0.01,0.02,0,0.06,0.03,0,0,0,0,0,0,0,0.05,0,0,0,0,0,0.01,0.02,0,0.02,0.19,0.51,0.02,0.13,0,0.15,0.01,0,0.07,0.07,0.01,0.08,0.010258347,0.142941416

150572,SMYD1,0,0,0,0,0,0,0,0,0,0.04,0,0,0,1.24,0,0,0,0.02,0,0,0,0,0,0,0.04,53.7,0.02,0.04,1.38,0.03,0.03,0.04,0.075294118,3.686666667,0.010260392,0.142941416

88,ACTN2,0.02,0.08,0.01,0,0.09,0.02,0,0,0,0.12,0,0,0,6.18,0.11,0.02,0,0.19,0.01,0,0.06,0,0,0.42,0.13,290.54,0.16,0.74,7.6,0.16,0.15,0.76,0.391176471,20.06133333,0.01032036,0.143674155

5349,FXYD3,0,0,0,0,0,0.05,0,0.06,0,0.28,0,0,0.33,0,0.06,0,0,0.17,0.05,0.06,0,0,0.9,9.38,0,0.12,0.3,0.19,0.09,0,0.09,0.32,0.045882353,0.778,0.010330247,0.143709147

5923,RASGRF1,0.07,0,0.05,0.13,0.02,0.48,0,0,0.12,0.13,0.02,0.1,0,2.26,0.44,0.33,0.13,0,0,0.02,0,0.02,0,0.05,0,0.46,0,0.04,0.03,0,0,0,0.251764706,0.041333333,0.010343757,0.143772481

1620,BRINP1,0,0,0,0,0.02,6.69,0,0,0.16,0.85,0.14,0,0,0.03,0.14,0.54,0,3.79,0.38,3.09,0.39,0.03,0.04,0,15.51,0.23,7.87,0,0.1,0.94,0.21,0.73,0.504117647,2.220666667,0.010349553,0.143772481

43847,KLK14,0,0.05,0,0.21,0.07,0,0,0,0,0,0,0,0.08,0,0,0,0,0,0.12,0.14,0.06,0,0,0.08,0.09,0.37,0.08,0.18,0,0.24,0.06,0,0.024117647,0.094666667,0.010396387,0.144320214

4036,LRP2,0,0,0,0,0,0,0,0,0,0,0,0,0,0.02,0,0,0,0.02,0,0.01,0,0,0.53,0,0,0.03,0.01,0,0.01,0,0.04,0,0.001176471,0.043333333,0.010494975,0.145585097

64800,EFCAB6,0.14,0.06,0.22,0.02,0.06,0.23,0.07,0.12,0.02,0.04,0.45,0.06,0.18,0.12,0.14,0.14,0.02,0.19,0.3,0.14,0,0.11,0.15,0.26,0.14,0.25,0.23,0.18,0.16,0.25,0.32,0.23,0.122941176,0.194,0.010551832,0.146269707

152831,KLB,0.1,0.11,0.08,0.23,0.14,0.07,0.05,0.15,0.1,0.14,0.11,0.09,0.08,0.1,0.12,0.15,0.09,0.04,0.02,0.04,0.03,0.02,3.76,0.04,0.18,0.05,0.06,0.07,0.05,0.14,0.11,0.04,0.112352941,0.31,0.010580471,0.146562456

161725,OTUD7A,0.02,0.04,0,0.03,0.05,0,0.03,0.03,0.14,0.31,0.02,0,0,0.03,0,0.02,0.03,0.15,0.04,0.1,0.07,0,0.09,0.03,0.03,0.05,0.08,0.02,0.11,0.2,0.08,0.06,0.044117647,0.074,0.010631961,0.147148258

5122,PCSK1,0.05,0,0.09,0.28,0.18,0.46,0.04,0.04,0,0.07,0.44,0.25,0.82,0,0.34,0,0.02,0.21,84.65,0.33,0.33,2.13,0.1,0,0.98,0.49,0.31,0.51,0.12,0.74,0.05,1.27,0.181176471,6.148,0.01063786,0.147148258

5027,P2RX7,0.18,0.38,0,1.6,1.1,0,0.32,0.07,0.48,0.38,0.18,0.02,0.03,0,0.07,0.04,0,0.11,0.55,0.38,1.33,1.09,1.33,132.32,0.37,0.02,0.78,0.18,0.12,0.73,0.39,0.54,0.285294118,9.349333333,0.010652231,0.147242539

51361,HOOK1,0,0.01,0.01,0.03,0.01,0.02,0,0.01,0.01,0,0.03,0,0,0.02,0,0,0,0.17,0.01,0,0.07,0.01,8.38,0.57,0.02,0.14,0.01,0.07,0.01,0,0.04,0.01,0.008823529,0.634,0.010669814,0.147381064

55203,LGI2,0,0,0.01,0.02,0.02,0,0,0.01,0,0,0.01,0.01,0.06,0,0.01,0,0,0.02,0.02,0.11,0,0,0,0,0.05,0.08,0.03,0.06,0.22,0.34,0.01,0.18,0.008823529,0.074666667,0.010699852,0.147561568

340544,MORF4L2-AS1,0.15,0.54,0.26,0.99,0.32,0.42,0.03,0.88,0.21,0.2,0.26,0.03,0.19,1.01,0.3,0.3,0.13,0,0.37,0.08,0.02,0.03,0.63,0.06,0.17,0.03,0.14,0.13,0.11,0.05,0.24,0.24,0.365882353,0.153333333,0.010702602,0.147561568

151888,BTLA,0.05,0.02,0,0,0,0,0,0,0,0,0,0,0,0,0,0,0,0.03,0,0.07,0,0,0,0,0.13,0,0.08,0.04,0.02,0.02,0.06,0,0.004117647,0.03,0.010723276,0.147561568

64799,IQCH,0.15,0.03,0.13,0.22,0.24,0.08,0.34,0.36,0.12,0.1,0.31,0.17,0.18,0.2,0.14,0.05,0.04,0.4,0.39,0.35,0.16,0.14,0.16,0.52,0.21,0.45,0.27,0.21,0.31,0.22,0.34,0.12,0.168235294,0.283333333,0.010753086,0.147561568

339829,CCDC39,0.68,0.49,0.74,0.84,0.86,0.33,0.4,0.52,0.3,0.84,0.61,0.35,0.95,1.19,0.62,0.94,0.05,0.12,0.29,0.3,0.12,0.04,0.04,0.75,0.5,1.49,0.21,0.81,0.22,0.29,0.51,0.18,0.63,0.391333333,0.010767531,0.147561568

285103,MED15P9,0.58,0.6,0.26,1.83,1.59,0.08,0.34,0.33,0.36,0.36,1.41,1.22,0.9,2.38,0.92,1.44,1.1,0.38,0.42,0.28,0.27,0.27,0.16,0.58,0.29,0.25,0.23,0.54,0.46,0.48,0.45,0.43,0.923529412,0.366,0.010781986,0.147561568

80178,C16orf59,2.77,0.99,1.2,2.79,1.58,2.23,0.67,3.99,2.81,1.34,1.1,0.58,0.83,6.04,1.92,1.61,6.2,1.1,0.16,0.98,2.87,1.18,0.69,1.01,0.67,1,0.51,1.36,1.05,0.9,1.58,0.48,2.273529412,1.036,0.010781986,0.147561568

6607,SMN2,7.04,5,6.34,0.2,0,8.06,13.56,14.93,10.31,5.32,6.7,12.07,12.83,0,5.87,6.3,11.57,11.83,11.23,11.96,17.57,5.29,11.91,12.74,13.93,21.43,5.21,14.93,12.77,9.7,12.29,15.76,7.417647059,12.57,0.010789216,0.147561568

64856,VWA1,2,6.88,6.62,0.49,0.68,1.49,0.48,2.26,1.19,6.5,3.07,3.19,1.29,9.22,5.94,3.27,0.5,92.07,19.9,0.86,3.04,6.22,42.04,6.77,9.01,4.72,2.85,8.34,9.05,1.49,8.34,5,3.239411765,14.64666667,0.010789216,0.147561568

10290,SPEG,34.52,30.88,25.65,7.6,4.01,20.87,27.64,29,37.99,29.5,46.02,70.05,52.45,39.91,22.67,19.64,25.76,42.88,27.34,19.64,1.16,0.16,0.2,0.21,40.88,6.8,22.94,23.55,17.21,10.4,24.27,17.25,30.83294118,16.99266667,0.010796449,0.147561568

192683,SCAMP5,6.49,1.88,6.73,8.1,12.39,3.32,0.78,0.92,3.15,2.63,14.97,7.37,6.68,0.87,4.97,3.63,5.88,5.65,3.9,3.15,6.66,1.59,0.38,1.55,0.81,1.03,1.21,0.51,3.04,0.65,0.98,2.64,5.338823529,2.25,0.010796449,0.147561568

5578,PRKCA,25.88,15.68,14.87,7.96,9.4,25.47,17.99,28.55,26.75,16.86,10.18,19.79,18.34,8.43,15.06,16.68,29.27,9.73,20.22,9.89,4.89,11.66,1.42,8.79,15.99,4.72,12.04,18.15,4.88,4.94,19.12,10.18,18.06823529,10.44133333,0.010796449,0.147561568

51444,RNF138,3.9,2.54,3.23,8.49,10.22,3.3,2.42,4.53,4.35,3.07,3.33,3.71,3.53,5.82,2.34,3.84,5.62,2.4,2.43,2.52,6.69,3.27,3.45,3.81,1.91,2.45,2.29,3.5,2,2.42,3.13,3.1,4.367058824,3.024666667,0.010796449,0.147561568

27230,SERP1,44.18,52.4,67.67,32.26,31.51,43.73,45.41,48.35,39.66,56.81,43.34,40.24,48.87,50.43,61.24,34.64,42.56,37.08,30.65,38.42,43.73,37.05,144.64,17.21,22.66,21.39,32.65,42.64,37.82,36.24,43.11,42.93,46.07647059,41.88133333,0.010796449,0.147561568

3671,ISLR,0.72,6.64,4.36,0,0.04,1.14,2.36,1.28,1.43,1.46,3.08,5.32,18.27,0.18,3.15,8.85,0.65,27.42,311.19,209.79,0.13,0.12,0.46,0.04,198.84,18.45,28.02,573.75,213.63,381.29,399.64,28.47,3.466470588,159.416,0.010796449,0.147561568

26266,SLC13A4,0,0,0.02,0.08,0.08,0,0.13,0.08,0.09,0.06,0.1,0,0,0.03,0.03,0,0.12,0.29,0.05,0.11,0,0.06,0.05,0.03,0.2,0.17,0.14,0.11,0.05,0.14,0.49,0.12,0.048235294,0.134,0.010866425,0.148386789

56964,WDR93,0.06,0,0,0,0,0,0,0,0.03,0,0.03,0,0,0.04,0.14,0,0,0,0.11,0,0.03,0.11,0,0.25,0.04,0.03,0.07,0.08,0.05,0,0.13,0.04,0.017647059,0.062666667,0.010872054,0.148386789

3036,HAS1,0.03,0.03,0,0,0,0,0,0,0,0.63,0.04,0.04,0.22,0,0,0.07,0,0,0.13,0.49,0,0,0.07,0,1.45,1.16,6.06,0.06,4.16,0.54,0.06,0.08,0.062352941,0.950666667,0.010924474,0.148997896

59344,ALOXE3,0.02,0,0,0,0,0.04,0.12,0.1,0.03,0.03,0,0.05,0.06,0,0.08,0.02,0.06,0,0,0.02,0,0,0,0.03,0.03,0.02,0,0,0,0,0,0,0.035882353,0.006666667,0.010940495,0.149112056

27285,TEKT2,0.14,0.71,0.25,0.1,0.1,0.05,0,0.49,0.22,0.13,0.35,0.06,0,0.19,0.12,0.34,0.18,0.05,0.22,0,0.09,0.06,0,0,0.07,0,0,0.39,0,0,0.12,0.12,0.201764706,0.074666667,0.011047005,0.149503043

5729,PTGDR,0,0,0,0,0,0.05,0,1.52,0,0,0,0,0,0,0,0,0,0,0,0.03,0,0,0.24,0,0.53,0.03,0,0.09,0.02,0.04,0.99,0.18,0.092352941,0.143333333,0.011052658,0.149503043

3352,HTR1D,4.51,0.02,0.22,1.05,1.88,0.15,0.19,1.7,1.33,0,0.13,0.09,0.03,0.51,0,0.13,1.11,0.16,0,0.03,0.71,14.24,0.05,0,0.03,0,0,0,0.02,0.05,0,0,0.767647059,1.019333333,0.011054542,0.149503043

79628,SH3TC2,0.09,0.06,0.06,0.79,0.28,0.12,0.1,0.28,0.22,0.15,0.12,0.04,0.07,0.06,0.01,0.16,0.07,0.05,0.09,0.04,0.07,0.09,0.04,0.1,0.01,0.06,0.03,0.06,0.03,0.03,0.2,0.05,0.157647059,0.063333333,0.011095891,0.149503043

284353,NKPD1,0.08,0.09,0.07,0.08,0.07,0.07,0,0.1,0.14,0.12,0.09,0.09,0.12,0.06,0.18,0.11,0.02,0.02,0.07,0.02,0.04,0.02,0.03,0.13,0.08,0,0,0.31,0.04,0.01,0.03,0.04,0.087647059,0.056,0.011118219,0.149503043

151354,FAM84A,0,0.03,0.01,0.23,0.04,0,0.3,0,0.01,0.07,0,0.04,0.08,0.08,0.04,0,0,1,0,0.42,0.03,0.05,0.24,0,1.28,3.29,0.04,0.42,0.17,0.08,1.35,0.04,0.054705882,0.560666667,0.01117546,0.149503043

5064,PALM,37.52,14,20.26,77.53,89.51,12.69,13.15,32.6,29.82,1.64,61.59,24.28,4.79,13.12,13.09,22.39,17.95,46.28,0.22,8.93,2.12,1.45,0.92,14.9,0.37,5.91,8.66,6.89,25.64,82.37,11.17,11.74,28.58411765,15.17133333,0.011226259,0.149503043

64857,PLEKHG2,11.59,15.14,12.73,23.51,24.95,7.47,6.62,11.23,24.3,9.84,12.3,5.5,2.35,18.2,14.56,16.16,28.68,15.29,5.94,7.75,7.89,9.28,0.92,5.76,10.66,6.35,6.88,11.22,11.64,7,11.42,7.33,14.41941176,8.355333333,0.011226259,0.149503043

731275,LINC01347,7.3,4,4.83,7.09,5.93,4.35,2.09,4.99,4.7,4.05,5.5,3.08,2.97,7.37,7.07,8.21,7.95,2.54,6.08,2.29,2.45,2.36,2.05,5.81,5.67,1.91,3.26,7.68,2.69,2.63,4.66,2.06,5.381176471,3.609333333,0.011226259,0.149503043

401149,LINC01061,7.82,4.73,6.73,6.2,7.33,5.66,2.9,8.07,6.02,4.55,6.99,4.95,4.57,9.07,9.96,8.59,6.82,2.97,10.12,3.23,3.32,2.5,4.06,12.14,4.79,1.71,3.64,8.19,3.61,3.36,4.16,3.49,6.527058824,4.752666667,0.011226259,0.149503043

23117,NPIPB3,24.52,9.98,18.66,38.95,32.8,16.2,15.26,25.06,23.1,10.67,12.95,10.19,13.1,23.38,20.83,15.38,34.33,8.82,13.32,13.02,9.77,10.36,10.5,101.58,9.59,15.58,13.23,7.3,3.76,16.56,16.7,11.93,20.31529412,17.468,0.011226259,0.149503043

80184,CEP290,4.08,5.26,3.58,5.49,5.29,4.51,3.49,5.3,3.24,7.55,4.98,3.28,5.44,4.75,4.42,4.81,4.46,2.54,2.59,2.82,3.84,4.92,0.59,2.85,5.09,2.66,2.41,8.11,2.65,2.71,5.98,3.57,4.701764706,3.555333333,0.011226259,0.149503043

64760,FAM160B2,23.42,22.11,22.68,28.62,34.88,21.96,15.97,24.9,23.96,19.72,27.32,21.43,18.62,32.5,21.45,18.21,23.28,17.44,22.33,19.6,16.95,13.41,8.67,32.52,23.31,20.64,18.9,15.88,18.67,18.75,23.15,15.71,23.59,19.062,0.011226259,0.149503043

2245,FGD1,10.04,12.83,10.07,10.56,10.96,9.39,4.93,10.62,9.36,9.15,11.63,11.66,9.16,11.27,7.97,10.95,8.5,5.12,5.59,9.84,7.65,4.46,0.32,15.53,8.73,3.78,6.39,11.88,9.21,7.53,9.65,6.44,9.944117647,7.474666667,0.011226259,0.149503043

162966,ZNF600,1.75,1.06,1.6,3.3,3.83,1.98,2.29,2.54,2.65,1.13,1.41,1.11,1.35,2.38,1.61,1.97,2.32,0.7,1.08,0.75,0.82,2.06,1.1,2.75,3.32,0.54,1.04,1.18,0.93,1.65,1.82,0.44,2.016470588,1.345333333,0.011226259,0.149503043

54876,DCAF16,7,7.95,7.88,8.91,6.88,7.67,4.48,9.01,7.61,7.24,7.5,8.03,5.99,14.74,7.68,9.02,5.91,6.1,6.81,4.2,5.8,3.27,1.07,10.15,5.47,6.29,4.07,9.15,4.94,5.18,8.29,6.54,7.852941176,5.822,0.011226259,0.149503043

57130,ATP13A1,20.08,14.38,16.46,20.56,24.01,21.03,12.75,18.98,15.25,15.72,25.51,14.32,9.04,22.91,18.49,13.48,21.43,16.54,12.15,12.42,14.37,11.31,8.77,19.75,18.87,11.23,15.43,17.27,10.83,11.5,17.33,12.82,17.90588235,14.03933333,0.011226259,0.149503043

8396,PIP4K2B,29.79,21.11,20.64,27.66,33.88,23.92,14.52,35.04,21.68,20.37,30.47,27.02,22.56,21.64,19.23,25.72,25.74,21.13,14.13,20.85,18.04,16.15,2.83,28.84,24.91,18.07,16.61,25.26,18.7,20.54,26.41,19.94,24.76411765,19.494,0.011226259,0.149503043

26151,NAT9,28.7,20.07,19.76,14.99,17.53,16.79,16.62,30.51,14.06,19.94,12.77,15.35,18.46,21.71,22.35,13.93,18.07,11.9,19.88,15.36,14.68,14.84,8.21,16.86,19.9,14.62,13.91,12.92,13.01,13.73,21.35,13.11,18.91823529,14.952,0.011226259,0.149503043

10927,SPIN1,25.09,23.06,24.75,25.98,27.21,24.73,16.15,28.27,24.49,20.06,44.83,29,25.33,31.1,18.27,30.27,27.19,23.87,19.26,18.86,17.35,11.56,2.69,26.33,33.69,15.52,21.73,21.54,20.75,19.62,27.94,20.24,26.22235294,20.06333333,0.011226259,0.149503043

441191,RNF216P1,7.03,7.22,8.53,11.84,12.37,8.52,7.45,6.82,8.25,4.32,6.92,4.67,4.77,14.27,8.56,6.46,9.5,7.95,5.27,5.65,8.44,5.9,3.8,5.55,5.92,6.35,6.1,5.25,6.89,4.96,8.47,4.18,8.088235294,6.045333333,0.011226259,0.149503043

58486,ZBED5,13.01,16.02,13.09,15.85,13.84,17.56,10.76,21.78,14.94,11.82,8.6,18.97,13.12,18.09,14.81,16.82,19.83,11.01,13.57,10.6,9.83,7.37,1.8,12.93,16.51,11.44,10.8,17.61,9.96,9.13,21.32,11.79,15.23,11.71133333,0.011226259,0.149503043

2176,FANCC,2.05,7.98,1.85,3.76,4.2,3.98,1.25,3.87,3.17,3.15,1.91,2.33,1.93,3.86,3.7,3.04,5.01,1.16,2.12,2.09,3.58,1.63,2.92,3.5,1.77,1.58,1.43,2.86,2.4,1.75,2.84,2.04,3.355294118,2.244666667,0.011226259,0.149503043

84792,FAM220A,13.11,9.4,10.63,10.07,10.05,12.14,8.58,12.74,12.77,10.53,12,11.06,9.88,8.07,11.53,10.32,16.99,12.48,5.56,9.58,6.53,9.22,3.72,10.47,8.86,7.83,10.03,10.23,12.6,9.27,10.21,9.46,11.16882353,9.07,0.011226259,0.149503043

8738,CRADD,2.74,3.52,1.88,4.81,5.41,13.39,6.39,5.07,4.42,5.75,10.43,9.17,7.19,4.08,3.05,2.18,2.81,9.35,7.07,8.09,5.14,7.35,9.57,13.69,7.93,11.28,7.5,6.17,4.18,4.22,5.5,9.38,5.428823529,7.761333333,0.011226259,0.149503043

51227,PIGP,4.32,5.15,4.22,5.71,5.69,7.46,11.39,12.92,8.3,6.01,9.36,9.45,12.57,5.6,3.87,6.05,6.21,6,17.68,11.47,9.62,8.14,12.38,22.79,11.86,18.86,8.35,10.57,6.77,6.79,6.74,7.36,7.310588235,11.02533333,0.011226259,0.149503043

3837,KPNB1,108.5,42.57,62.72,70.06,73.79,56.89,67.58,82.55,56.96,70.32,54.18,53.01,66.29,76.61,53.99,50.92,83.45,49.36,31.34,42.06,101.56,66.09,22.36,51.81,54.13,65.25,49.94,56.69,53.82,46.96,70.27,45.37,66.49352941,53.80066667,0.011226259,0.149503043

6041,RNASEL,1.91,2.56,0.7,1.2,2.03,2.69,2.3,3.6,2.38,0.55,3.75,0,0.02,2.63,1.42,1.95,2.62,2.49,3.92,3.82,1.07,0.49,0.82,7,3.72,2.66,2.97,4.41,2.52,3.41,3.84,4.34,1.900588235,3.165333333,0.011226259,0.149503043

55361,PI4K2A,11.88,5.71,9.79,8.58,10.96,12.41,11.08,9.92,9.5,6.68,11.45,12.72,13.22,11.28,7.12,9.2,11.99,13.16,7.45,13.09,12.05,13.49,15.64,20.21,14.11,12.86,13.12,7.93,11.6,13.96,11.19,7.77,10.20529412,12.50866667,0.011226259,0.149503043

29780,PARVB,13.87,11.56,10.35,56.68,55.98,9.89,8.52,3.68,6.5,14.59,9.62,0.09,4.09,14.85,11.62,4.61,7.84,13.3,14.25,29.85,64,52.56,4.11,27.75,13.95,31.68,13.83,22.73,12.79,8.9,16.28,21.34,14.37294118,23.15466667,0.011226259,0.149503043

138649,ANKRD19P,64.65,72.56,65.66,55.75,55.09,78.17,49.44,73.34,56.9,61.22,43.59,92.93,79.62,62.09,69.44,68.56,68.5,61.72,65.18,92.36,106.6,68.33,60.85,82.24,82.01,85.19,71.1,66.39,77.97,104.01,85.54,148.07,65.73588235,83.83733333,0.011226259,0.149503043

53340,SPA17,3.58,1.1,1.99,2.94,3.4,2.72,2.38,4.13,1.39,1.76,3.1,2.97,2.85,1.8,1.52,3.74,2.49,3.41,2.79,3.64,4.81,4.02,1.22,5.47,3.02,4.35,3.88,2.76,2.45,3.13,4.89,3.51,2.58,3.556666667,0.011226259,0.149503043

8835,SOCS2,1.89,0.16,0.17,4.66,5.34,3.57,6.68,1.13,4.36,0.32,39.73,3.86,1.95,5.11,0.62,0.67,2.45,4.03,31.59,11.11,1.98,1.63,19.17,7.15,3.72,2.77,9.96,5.57,2.35,8.09,5.8,7.67,4.862941176,8.172666667,0.011226259,0.149503043

256472,TMEM151A,0.23,0.05,0.12,0.03,0.46,0.08,0.27,0.13,0.2,3.94,0.21,0.37,0.78,0.96,0.34,0,0.54,0.28,0.32,0.06,0.03,0,0.28,0,0.04,0.29,0.03,0.03,0.13,0.05,0.02,0,0.512352941,0.104,0.011230175,0.149503043

55258,THNSL2,0.05,0.04,0.2,0,0.2,0,3.72,8.75,6.82,0,0.05,4.71,0.6,10.36,0.17,0,0.06,0.48,18.35,5.4,0,0.65,4.34,24.97,5.32,3.62,4.61,6.43,6.76,14.65,4.57,1.36,2.101764706,6.767333333,0.011237658,0.149503043

7544,ZFY,0,0.01,0,0.03,0,0,0,0.02,0.02,0,0.02,0.04,0.02,0.06,0,0,0.04,0.02,1.63,0,0.04,0,1.55,3.48,0,2.52,0.02,2.2,2.26,0.01,2.41,2.08,0.015294118,1.214666667,0.011257946,0.149670786

284217,LAMA1,0.52,0,0.98,0.01,0.12,0.09,1.09,0.13,0.12,0.01,0.05,0,0,0.42,1.11,0.03,0.52,2.19,0.43,0.71,0,0.02,0.13,0.14,0.61,2.19,0.55,0.84,2.13,0.97,3.84,2.75,0.305882353,1.166666667,0.011290103,0.149990855

9452,ITM2A,0.2,0,0,12.19,1.51,0,0.59,0,0.1,0,0.33,0.05,0.05,0,0,0.04,0,0.34,20.88,0,9.88,44.18,1.37,0.42,0,0.31,0.05,0.11,6.36,3.5,2.88,0.05,0.885882353,6.022,0.011299425,0.149990855

4542,MYO1F,0.02,0.13,0.1,0.16,0.13,0.29,0.2,0.18,0.18,0.02,0.27,0.16,0.04,0.07,0.21,0.07,0.11,0.06,0.25,0.25,0.25,0.35,6.56,0.62,0.09,0.17,0.14,0.22,0.17,0.27,0.22,0.21,0.137647059,0.655333333,0.011305109,0.149990855

100505483,PRKAG2-AS1,0,0,0,0,0,0,0,0,0.33,0,0,0,0,0,0,0,0,0,0.18,0,0,0,0.91,3.16,0,0.11,0,1.02,0,0.09,0.08,0,0.019411765,0.37,0.011334099,0.150064297

340156,MYLK4,0.19,0.29,0.52,0.62,0.47,0.49,0.2,0.62,0.65,0.24,0.21,0.37,0.11,0.75,0.45,0.37,0.51,0.13,0.15,0.29,0.2,0.16,0.12,0.21,0.08,0.5,0.09,0.22,0.32,0.41,0.47,0.3,0.415294118,0.243333333,0.011357702,0.150064297

284408,ZNF790-AS1,0,0.14,0,1.08,1.27,0,0.44,1,1.15,0,0.09,0.1,0,1.43,0,0.09,0,0.68,0.72,1.31,0.48,0.45,0,1.35,1.91,1.05,0.72,1.19,0.8,0.84,1.01,1.82,0.399411765,0.955333333,0.011365642,0.150064297

340485,ACER2,0.23,0.23,0.11,4.61,64.99,0.22,0.19,0.26,0.33,0.43,0.39,0.06,0.22,3.58,0.15,0.18,0.03,1.39,0.19,0.36,4.77,3.76,0.54,0.42,1,0.51,0.35,0.27,0.39,0.49,0.64,2.32,4.482941176,1.16,0.01137275,0.150064297

7464,CORO2A,2.72,0.84,1.85,0.31,0.17,0.99,1.06,1.2,1.26,0.75,1.18,1.12,0.38,4.17,0.61,1.31,1.34,4.07,0.55,0.14,0.3,1.66,0.93,1.05,0.14,0.55,0.39,0.2,0.39,0.25,0.37,0.16,1.250588235,0.743333333,0.011380277,0.150064297

8029,CUBN,0.22,0.24,0.44,0.64,0.35,0.29,0.22,0.39,0.17,0.1,0.2,0.21,0.57,0.15,0.14,0.16,0.05,0.54,0.77,0.86,1.01,1.32,0.03,2.05,0.44,0.19,0.16,0.33,0.7,0.34,0.49,0.42,0.267058824,0.643333333,0.011380277,0.150064297

72,ACTG2,12.9,104.64,39.36,0.7,6.4,2.21,95.86,41.34,834.45,168.04,28.95,21.39,25.76,1.15,146.37,24.84,406.4,632.73,0.17,0,0.12,3.33,0.12,0,1.83,0.49,1.33,62.39,102.56,0.46,81.36,4.67,115.3388235,59.43733333,0.011387807,0.150064297

100288730,PAN3-AS1,2.04,1.79,1.85,2.31,2.69,0.87,1.02,2.76,1.38,1.55,1.77,1.39,1.77,2.3,2.93,1.55,1.16,0.39,1.01,1.23,1.08,0.04,0.38,2.43,2.1,0.55,0.64,1.94,1.17,1.03,1.8,1.07,1.831176471,1.124,0.011387807,0.150064297

27244,SESN1,2.63,5.67,6.57,12.36,8,10.06,5.06,4.14,3.69,7.05,3.65,2.73,10.92,8.31,4.4,1.89,1.72,15.35,3.2,17.42,8.31,7.91,5.03,11.07,10.98,12.93,5.06,9.52,5.23,8.25,8.02,13.78,5.814705882,9.470666667,0.011387807,0.150064297

203523,ZNF449,3.22,2.65,3.13,19.06,9.29,2.98,3.68,4.14,4.05,4.02,3.15,2.73,3.59,5.33,5.26,3.41,4.02,2.54,4.18,2.08,2.87,3.16,0.62,6.61,2.83,2.04,2.41,3.37,2.84,1.73,6.66,2.07,4.924117647,3.067333333,0.011395339,0.150064297

26043,UBXN7,6.72,7.31,5.05,10.59,8.08,5.78,6.24,7.86,6.87,6.87,7.2,6.22,5.33,6.27,5.53,7.48,4.72,4.45,5.36,3.99,6.97,5.16,0.35,4.71,6.8,2.81,4.56,7.96,4.73,5.7,8.01,3.51,6.712941176,5.004666667,0.011395339,0.150064297

2769,GNA15,0,0,0,0.04,0,0,0.04,0,0,0.13,0,0,0,0,0.04,0,0,0.04,0,0.04,0.03,0.12,1.32,0,0.05,0.08,0,0.06,0.03,0.03,0,0.04,0.014705882,0.122666667,0.011438629,0.150532671

4139,MARK1,0.97,0,0.01,1.35,1.27,0.05,0.23,0.03,0.02,0,0.94,0,0,0,0,0,0.02,1.79,0.21,0.13,1.3,0.05,0.03,1.95,0.09,0.08,0.08,0.01,0.44,0.39,1.3,0.26,0.287647059,0.540666667,0.011549641,0.151842712

7479,WNT8B,0,0,0,0.08,0.04,0,0.12,0.12,0.09,0,0.04,0.04,0,0.1,0.04,0.07,0.14,0,0,0,0,0.04,0,0,0,0,0,0,0,0,0.06,0.09,0.051764706,0.012666667,0.011553758,0.151842712

387700,SLC16A12,0.16,0.64,0.39,0.02,0.13,0.94,0.87,1.03,0.67,2.13,0.22,0.24,0.16,2.07,0.22,1.38,0.27,0.27,0.02,0.02,0.01,0.02,0.03,0,0.04,2.73,0.02,5.28,0.03,0.03,0.63,0.17,0.678823529,0.62,0.011820461,0.155067106

146547,PRSS36,0.09,0.05,0.02,0.03,0.06,0.21,0,0.19,0,0.07,0,0.07,0.35,0.08,0.17,0.08,0.04,0.12,0.13,0.16,0.11,0.12,0.17,0.37,0.17,0.1,0.07,0.05,0.1,0.22,0.2,0.1,0.088823529,0.146,0.011882774,0.155067106

148198,ZNF98,0,0,0,0,0,0,0,0,0,0,0,0,0,0,0,0,0,0,0,0,0,0.04,0,0,0,0,0,0.03,0.03,0.03,0.03,0,0,0.010666667,0.011906723,0.155067106

80129,CCDC170,0.07,0,0,0,0,0.08,0.09,0.06,0.19,0,0.81,0.1,0.15,0,0.13,0.01,0.22,0.18,1.27,0.53,0.01,0.21,0,0,1.19,0.35,0.91,0.01,0.21,0.68,0.47,0.29,0.112352941,0.420666667,0.011973605,0.155067106

23150,FRMD4B,0.29,0.06,0.04,12.25,13.55,0.21,0.09,0.14,0.13,0.04,0.01,0,0.09,4.91,0.02,0.14,0.14,0.16,5.72,0,14.91,6.5,1.48,13.64,0.15,5.24,0.34,0.22,0.49,0.1,0.46,0.48,1.888823529,3.326,0.011976531,0.155067106

843,CASP10,0.03,0.15,0.04,5.94,9.23,0.06,0.08,0.09,0.09,0.07,0.04,0.1,0.28,0.4,0.06,0.14,0.1,0.01,0.15,1.27,10.33,3.44,1.56,0.12,0.67,0.24,0.22,0.29,0.13,1.21,0.24,0.89,0.994117647,1.384666667,0.01198436,0.155067106

653499,LGALS7B,0.16,0,0,0,0.17,0,0,0,0,0.78,0,0,0,0.42,0.19,0.16,0,1.43,0,0.17,0.15,0,0,0.19,0,1.21,0.54,0.85,0.44,0.43,0.36,0.71,0.110588235,0.432,0.01199821,0.155067106

144406,WDR66,0.45,0.39,0.14,1.25,2.25,0.9,1.14,0.42,0.44,0.76,0.95,0.3,1.2,1.8,3.79,0.98,2.15,0.9,0.54,0.36,0.75,0.98,0.12,0.31,0.79,0.24,0.71,0.14,0.25,0.09,0.75,0.55,1.135882353,0.498666667,0.012000024,0.155067106

54058,C21orf58,2.37,1.47,1.19,1.77,1.01,1.7,1.22,2.74,2.12,1.92,1.7,0.83,1.36,5.71,2.82,1.91,3.12,1.07,0.96,1.91,1.42,0.55,0.28,1.35,1.57,0.78,0.69,2.79,1.34,1.07,1.97,1.1,2.056470588,1.256666667,0.01200786,0.155067106

11169,WDHD1,2.02,0.99,1.53,2.1,1.4,2.02,1.26,2.69,1.89,1.7,1.62,1.47,1.62,3.35,1.76,2.59,5.27,1.48,0.49,1.46,3.61,1.27,0.07,1.8,1.49,1.05,1.3,2.06,1.41,0.99,1.52,0.75,2.075294118,1.383333333,0.01200786,0.155067106

136051,ZNF786,1.88,1.85,1.36,3.33,2.9,2.59,1.47,2.05,3.48,1.67,2.62,1.35,0.94,2.31,1.27,2.35,3.13,1.47,0.92,1.43,2.37,0.77,0.73,3.66,1.44,1.24,1.24,1.01,1.68,1.06,2.22,1.12,2.15,1.490666667,0.012015698,0.155067106

51755,CDK12,9.07,5.27,6.35,10.12,8.81,7.37,5.68,9.54,6.49,5.05,5.92,6.03,4.82,9.24,5.81,6.37,7.39,5.09,4.68,4.97,8.28,6.18,2.99,7.71,5.69,5.56,5.08,5.68,5.21,5.56,6.39,3.95,7.019411765,5.534666667,0.012015698,0.155067106

54753,ZNF853,11.52,1.06,6.31,4.56,7.74,4.15,0.35,11.44,10.97,0.81,14.65,3.96,0.83,4.49,3.61,4.58,12.44,6.87,1.51,0.57,0.97,0.07,0.16,4.11,0.3,1.06,0.71,4.74,4.47,0.41,5.4,0.99,6.086470588,2.156,0.012023538,0.155067106

91316,GUSBP11,4.44,5.34,4.11,7.03,6.88,2.91,2.07,6.53,5.6,4.95,4.65,2.64,3.25,6.16,7.12,8.32,4.38,1.62,5.62,2.61,2.83,2.75,2.07,5.47,5.02,1.31,3.11,5.06,2.55,3.07,4.27,3.5,5.081176471,3.390666667,0.012023538,0.155067106

4820,NKTR,13.69,12.63,15.6,30.66,23.14,8.71,11.39,16.28,12.37,17.19,10.35,7.82,10.27,24.95,18.88,16.3,14.08,6.6,16.03,7.07,9.78,8.18,4.22,16.63,14.82,5.56,6.61,17.19,7.64,6.76,22.9,7.62,15.54764706,10.50733333,0.012023538,0.155067106

100129792,CCDC152,0.17,0.59,1.7,1.76,3.93,4.14,0.93,2.3,2.29,2.21,36.72,0.19,0.13,7.05,0.6,1.39,0.16,1.98,5.25,9.54,1.39,0.12,76.81,2.71,13.98,4.07,3.56,56.13,1.48,19.41,15.35,20.14,3.897647059,15.46133333,0.012023538,0.155067106

284338,PRR19,1.16,1.07,1.25,2.81,2.28,1.37,0.31,1.69,2.14,1.26,1.19,3.39,1.98,2.26,1.21,2.12,1.63,0.98,0.36,0.47,2.18,0.81,0.28,2.98,1.63,0.22,0.11,0.34,0.96,0.49,2.42,0.87,1.712941176,1.006666667,0.012023538,0.155067106

23074,UHRF1BP1L,2.98,3.55,3.11,1.5,1.26,4.33,4.31,4.18,2.69,4.07,2.71,4.7,5.51,3.69,3.42,3.03,4.87,4.04,6.41,5.35,4.53,3.57,3.14,4.7,6.29,9.26,4.76,3.76,3.28,5.79,3.56,4.79,3.524117647,4.882,0.012023538,0.155067106

79632,FAM184A,0.07,0,0,0.07,0.08,0.14,0,0.05,0.02,0,0.3,0,0.1,0.03,0.02,0,0.05,0.31,0.32,0.04,0.22,0.02,0.32,0.02,0.28,0.46,0.02,0.05,0.24,0.02,0.38,0.09,0.054705882,0.186,0.012077104,0.155067106

4821,NKX2-2,0,0,0,0,0,0,0,0,0,0,0,0,0,0,0,0,0,0.56,0.03,0,0,0,0,0,0,0.08,0.12,0,0,0,0.03,0,0,0.054666667,0.012083454,0.155067106

442319,ZNF727,0,0,0,0,0,0,0,0,0,0,0,0,0,0,0,0,0,0,0.98,0,0,0.05,0.08,0,0,0,0.05,0,0,0.16,0,0,0,0.088,0.012083454,0.155067106

5100,PCDH8,0,0,0,0,0,0,0,0,0,0,0,0,0,0,0,0,0,0.46,0,0,0,0,0,0.02,0.03,0.06,0,0.02,0,0,0,0,0,0.039333333,0.012083454,0.155067106

81626,SHCBP1L,0,0,0,0,0,0,0,0,0,0,0,0,0,0,0,0,0,0,0.03,0,0,0,0,0,0.04,0,0,0.05,0,0.11,0.03,0,0,0.017333333,0.012083454,0.155067106

100526772,TMEM110-MUSTN1,0,0,0,0,0,0,0,0,0,0,0,0,0,0,0,0,0,0,0.4,0,0.3,0,3.92,0,0,0,0,0,0.35,0,0,0.24,0,0.347333333,0.012103166,0.155067106

55843,ARHGAP15,0,0,0,0,0,0,0,0,0,0,0,0,0,0,0,0,0,0,0,0,0,0,0.88,0.2,0,0,0,0.04,0,0,0.07,0.4,0,0.106,0.012103166,0.155067106

100528019,HSPB2-C11orf52,0,0,0,0,0,0,0,0,0,0,0,0,0,0,0,0,0,0,0.54,0.21,0,0,0,0.24,0,0,0,0,0,0.2,0,0.23,0,0.094666667,0.012103166,0.155067106

154860,FEZF1-AS1,0,0,0,0,0,0,0,0,0,0,0,0,0,0,0,0,0,0.12,0,0,0,0,0.16,0.07,0,0.06,0,0.02,0,0,0,0,0,0.028666667,0.012103166,0.155067106

6656,SOX1,0,0,0,0,0,0,0,0,0,0,0,0,0,0,0,0,0,0.09,0,0,0,0,0.07,1.73,0,0,0,0,0,0.02,0.05,0,0,0.130666667,0.012103166,0.155067106

9745,ZNF536,0,0,0,0,0,0,0,0,0,0,0,0,0,0,0,0,0,0.47,0,1.34,0,0,0,0.76,0,0,0,0,0.14,0,0.08,0,0,0.186,0.012103166,0.155067106

27022,FOXD3,0,0,0,0,0,0,0,0,0,0,0,0,0,0,0,0,0,0.04,0,0,0,0,0,20.8,0,0,0,0.06,0,0,0.4,0.13,0,1.428666667,0.012103166,0.155067106

11211,FZD10,0,0,0,0,0,0,0,0,0,0,0,0,0,0,0,0,0,0,0.02,0,0,0,0,0,0,3.24,0,0.22,0.42,0,0.32,0,0,0.281333333,0.012103166,0.155067106

150568,LINC01102,0,0,0,0,0,0,0,0,0,0,0,0,0,0,0,0,0,0.52,0,0,0,0,0,0,0.07,0.11,0,0.09,0,0.04,0,0,0,0.055333333,0.012103166,0.155067106

441161,OOEP,0,0,0,0,0,0,0,0,0,0,0,0,0,0,0,0,0,0,0.31,0,0,0.14,0,0,0.15,0,0,0,0.1,0,0.39,0,0,0.072666667,0.012103166,0.155067106

442211,ATP6V0CP3,0,0,0,0,0,0,0,0,0,0,0,0,0,0,0,0,0,0,0.35,0.14,0.12,0,0,0.3,0,0,0,0.57,0,0,0,0,0,0.098666667,0.012103166,0.155067106

84701,COX4I2,0,0,0,0,0,0,0,0,0,0,0,0,0,0,0,0,0,0.12,0.21,0,0,0,0,0,0,0,0,0.41,0,0,0.1,0.68,0,0.101333333,0.012103166,0.155067106

56165,TDRD1,0,0,0,0,0,0,0,0,0,0,0,0,0,0,0,0,0,0,0,0,0,0,0,0,0,0.11,0,0.04,0.07,0.09,0.08,0,0,0.026,0.012103166,0.155067106

387695,C10orf99,0,0,0,0,0,0,0,0,0,0.12,0,0,0,0,0,0,0,0.1,0,0,0,0,0,0,0.12,0.31,0,0.08,0,0.17,0.08,0.11,0.007058824,0.064666667,0.012134972,0.155067106

254528,MEIOB,0.17,0.37,0,0.32,0.05,0.11,0,0.19,1.54,0.55,0.39,0,0.05,0.23,0.15,0.51,1.1,0.14,0.04,0.05,0,0.1,0,0,0.06,0.17,0,0.08,0.04,0.08,0.15,0.1,0.337058824,0.067333333,0.012165003,0.155067106

54894,RNF43,0,0.01,0,0,0,0,0,0,0,0.1,0,0,0,0,0,0,0,0.14,0,0,0.02,0,0.3,0.11,0.02,0,0,0.01,0,0,0.01,0.02,0.006470588,0.042,0.012176847,0.155067106

54627,MAP10,0,0,0,1.3,0.88,0,0.38,0.45,0.4,0,0,0,0,1.16,0,0,0,0.96,0.92,0.45,0.76,0.4,0.1,0.36,0.94,0.34,0.31,0.83,0.49,0.3,0.86,0.22,0.268823529,0.549333333,0.012207339,0.155067106

3209,HOXA13,0,0,0,0.03,0,0,3.19,0,0,0,0,0,0,0,0,0,0,0,4.14,1.11,0.06,0,0,0,2.03,0.42,4.38,0,0,0.21,0.07,0,0.189411765,0.828,0.012270966,0.155067106

2532,ACKR1,0,0,0,0,0,0,0,0,0.55,0.08,0,0,0,0,0,0,0,0.23,0,0.06,5.3,0,1.26,0,0,5.33,0.2,0.58,0.03,0,0,0,0.037058824,0.866,0.012270966,0.155067106

286333,FAM225A,0.27,0.09,0,0,0,0.01,0.46,0,0,2.25,0.01,0,0,1.19,0.33,0,0,0.7,0,0.34,0,0.79,0.36,0.06,9.73,2.22,3.04,0,0.92,3.42,1.35,0.2,0.271176471,1.542,0.012335354,0.155067106

100526837,EEF1E1-BLOC1S5,0.23,0.84,0,0.62,0.24,0.56,0,0,0,0.62,0.87,0,0.8,0,0,0,0,0,0.25,0,1.42,0,0.59,2.98,0.91,1.31,0.99,0,0.82,1.05,1.82,1.7,0.281176471,0.922666667,0.012351028,0.155067106

400765,MIR137HG,0.56,0.12,0.48,0,0.06,0.09,0.85,0.57,0.38,0.31,0.06,0.28,0.62,0.08,0.07,0.44,0.66,0.16,0,0.26,0.06,0.39,0,0,0.44,0,0.54,0.05,0.03,0,0.05,0.18,0.331176471,0.144,0.012357806,0.155067106

165082,ADGRF3,0.17,0.14,0.09,0.3,0.29,0.07,0.17,0.12,0.08,0.11,0.14,0.12,0.11,0.18,0.03,0.05,0.15,0.06,0.03,0.11,0.06,0.04,0.06,0.04,0.09,0.12,0.03,0.11,0.08,0.12,0.04,0.2,0.136470588,0.079333333,0.012398149,0.155067106

5919,RARRES2,44.71,0.74,5.99,0.11,2.69,3.05,0,0.34,0,0.26,0.41,0,0.37,83.3,0,4.93,1.76,82.67,1.67,11.51,0,0,525.1,2.15,43.84,53.34,8.78,81.34,127.02,205.45,1.4,17.74,8.744705882,77.46733333,0.012406225,0.155067106

3049,HBQ1,0.36,0,0.1,0.38,0.26,0,0,0,0,0.94,0,0.28,0.15,0.48,0.43,0.36,0.15,0.39,0,0,0,0,0,0,0,0,0.27,0,0,0,0,0.14,0.228823529,0.053333333,0.012453518,0.155067106

55613,MTMR8,0.03,0,0,0,0.06,0,0,0,0,0,0.11,0,0,0,0,0,0,0.06,0,0,0.1,0.07,0.1,0.48,0,0.03,0.03,0,0.12,0,0.16,0,0.011764706,0.076666667,0.012463792,0.155067106

25830,SULT4A1,0.38,1.57,2.6,0.09,0.32,0.46,0.22,0,0.34,3.15,3.89,0.21,0.07,4.15,2.83,0.09,0.22,0.82,0.08,0,0.06,0,0,0,0.08,0.3,0.03,0.5,0.56,0.03,0.54,0.04,1.211176471,0.202666667,0.012495221,0.155067106

57172,CAMK1G,0.87,3.12,0.1,2.74,0.84,5.78,0.12,3.32,0.54,7.07,2.96,3.07,2.82,15.32,0.21,0.29,0.07,0.12,0,0,0,0.21,0.06,0,2.98,0.03,0,8.86,0.18,0.32,9.9,0.24,2.896470588,1.526666667,0.012511433,0.155067106

388531,RGS9BP,0.1,0.06,0.06,0.03,0.05,0.07,0.08,0,0.09,0.13,0.08,0.03,0.25,0,0.09,0.05,0.13,0,0.07,0,0,0.03,0.1,0,0,0.08,0.03,0.11,0,0,0.02,0,0.076470588,0.029333333,0.012514938,0.155067106

51716,CES1P1,0,0.21,0,0,0,0,0,0,0,0.72,0,0,0,0,0,0,0,0.31,0,0.14,0,0,0.31,0,0.04,0.22,0,0.09,0,0.09,0.47,0.04,0.054705882,0.114,0.012521788,0.155067106

7915,ALDH5A1,1.85,0.23,1.56,2.36,2.11,0.73,1.24,2.79,2.91,1.72,2.56,0.72,1.03,3.01,0.86,1.67,2.64,1.55,1.98,0.35,0.88,0.24,4.03,0.26,0.21,1.89,0.82,0.29,0.56,0.41,1.31,0.12,1.764117647,0.993333333,0.012612716,0.155067106

79047,KCTD15,20.91,10.6,19.04,24,31.83,13.77,10.71,39.73,35.38,12.16,16.84,12.67,8.91,3.8,21.48,19.18,31.24,15.18,11.91,10.81,9.91,11.15,1.46,23.12,6.32,6.08,10.56,6.75,23.95,11.4,14.01,7.84,19.54411765,11.36333333,0.012612716,0.155067106

10299,6-Mar,15.64,23.18,14.22,21.6,20.76,18.27,14.28,17.23,15.47,23.19,30.46,22.55,13.7,19.62,20.48,20.35,8.96,15.78,12.94,10.29,11.63,11.55,5.77,18.23,17.86,8.48,11.94,34.76,9.59,9.17,23.77,14.07,18.82117647,14.38866667,0.012612716,0.155067106

27122,DKK3,234.13,216.46,432.45,70.14,54.53,262.4,94.88,281.43,262.43,256.91,168.57,365.07,174.98,178.61,91.7,204.19,208.54,298.08,189.44,155.33,59.26,747.77,2.93,25.6,101.98,54.52,23.35,145.37,84.84,48.55,117.57,157.13,209.26,147.448,0.012612716,0.155067106

171546,SPTSSA,12.47,13.99,14.17,15.98,20.61,17.56,7.97,14.23,14.02,12.8,26.08,12.04,13.97,15.64,6.52,14.39,12.19,11.81,13.33,10.08,14.22,7.69,10.44,27.61,5.85,8.35,5.74,11.33,10.22,8.65,10.64,15.02,14.39,11.39866667,0.012612716,0.155067106

55691,FRMD4A,45.47,7.3,6.19,32.54,32.47,8.23,8.15,23.26,28.34,10.98,10.15,19.3,14.12,12.51,4.18,11.33,26.01,5.39,0.59,3.52,18.88,24.45,1.97,6.98,8.37,3.5,8.24,8.79,9.68,4.87,27.03,8.1,17.67823529,9.357333333,0.012612716,0.155067106

22893,BAHD1,6.19,8.93,8.31,10.5,13.82,12.08,4.57,7.55,9.11,9.17,11.03,12.99,9.06,7.11,5.72,7.87,8.27,6.24,3.37,6.04,9.37,7.48,2.93,20,6.22,4.74,5.36,9.95,4.11,4.99,6.59,6.29,8.957647059,6.912,0.012612716,0.155067106

9139,CBFA2T2,5.09,6.75,5.03,9.86,8.98,4.66,3.5,5.92,5.39,5.49,6.26,4.75,5.8,6.04,6.02,8.02,5.21,3.48,5.41,3.9,3.78,3.7,2.06,8.58,5.24,3.24,3.84,6.59,4.64,4.54,6.08,4.87,6.045294118,4.663333333,0.012612716,0.155067106

6431,SRSF6,56.75,40.69,49.37,67.62,54.41,34.4,38.39,62.1,53.02,34.02,49.16,33.84,27.62,81.9,44.03,39.19,62.02,32.28,51.87,24.91,44.35,32.3,28.01,42.23,58.22,37.45,26.36,32.59,30.72,28.9,58.2,30.19,48.73705882,37.23866667,0.012612716,0.155067106

55778,ZNF839,3.61,2.61,3.17,4.9,4.86,3.74,2.62,4.43,3.28,3.34,3.68,3.22,2.6,4.95,3.29,4.65,5.35,2.31,3.75,2.78,2.51,2.92,1.1,4.97,2.9,1.91,2.49,3.82,2.1,2.38,3.31,3.55,3.782352941,2.853333333,0.012612716,0.155067106

126792,B3GALT6,19.53,12.11,15.77,21.93,28.87,17.87,11.06,16.52,13.49,14.24,18.94,18.33,13.74,18.31,12.71,13.65,24.27,19.39,11.6,13.39,16.85,11.28,5.86,11.43,12.47,10.36,15.56,13.36,14.56,13.27,16.66,13.09,17.13764706,13.27533333,0.012612716,0.155067106

729975,LINC01530,3.89,2.77,2.74,5.6,4.43,2.32,1.8,3.58,3.21,2.65,2.59,3.02,2.13,4.92,2.96,2.4,2.7,1.74,3.75,1.15,2.11,2.26,2.24,4.49,2.95,1.23,1.39,3.26,1.17,2.63,2.19,2.09,3.159411765,2.31,0.012612716,0.155067106

55139,ANKZF1,14.55,11.4,10.62,14.26,15.49,12.91,10.64,15.28,13.77,13.54,12.29,13.92,10.48,15.54,20.96,13.82,9.71,9.06,15.02,9.98,9.18,6.94,5.78,18.23,14.4,6.29,7.44,13.4,8.5,7.43,15.17,8.61,13.48117647,10.362,0.012612716,0.155067106

9610,RIN1,15.34,48.7,15.45,14.84,13.21,29.87,4.66,47.49,10.16,50.67,8.53,30.81,15.41,46.19,27.39,27.07,27.13,3.35,5.91,15.44,4.67,10.62,0.99,7.04,27.65,6.37,14.25,48.57,4.87,9.91,30.07,11.62,25.46588235,13.422,0.012612716,0.155067106

54778,RNF111,5.93,5.28,5.4,12.27,8.93,6.64,6.11,6.06,6.56,7.27,7.22,5.75,5.92,5.59,4.76,5.56,7.31,4.96,5.57,5.19,6.79,4.63,2.29,8.48,6.81,3.41,5.54,4.89,5.48,4.66,6.28,4.52,6.621176471,5.3,0.012612716,0.155067106

56998,CTNNBIP1,10.28,13.54,10.29,38.16,47.45,8.61,6.64,11.79,8.87,9.75,15.52,13.67,11.93,16.5,15.27,8.45,12.45,14.4,5.56,8.47,14.32,12.65,2.36,11.12,7.2,6.51,6.58,8.24,8.35,7.38,15.59,8.22,15.24529412,9.13,0.012612716,0.155067106

23641,LDOC1,27.85,25.1,29.18,38.7,31.5,33.77,20.48,37.32,26.29,29.11,25.52,38.82,43.75,48.56,24.37,22.7,20.21,25.83,17.43,13.2,35.46,23.12,1.54,7.31,44.62,10.28,11.98,28.78,13.24,13.25,71.61,19.78,30.77823529,22.49533333,0.012612716,0.155067106

2582,GALE,7.52,9.69,11.02,30.69,32.92,17.25,8.83,11.65,6.85,22.34,11.03,8.16,10.72,33.46,13.39,11.48,21,6.53,11.74,5.47,24.84,14.13,10.58,20.62,7.45,10.73,6.08,4.13,4.47,2.18,8.8,3.5,15.76470588,9.416666667,0.012612716,0.155067106

9520,NPEPPS,54.26,37.34,32.59,37.71,30.42,34.76,30.2,62.95,24.29,37.33,34.78,40.96,37.79,55.44,24.3,37.89,17.14,19.85,15.06,19.51,26.77,31.01,4.55,30.76,42.29,36.59,18.12,33.98,22.56,22.47,40.17,24.67,37.06764706,25.89066667,0.012612716,0.155067106

100996485,C5orf66,1.55,1.01,1.39,2.6,1.91,1.31,1.41,1.52,1.63,1.56,1.68,1.17,2.22,3.41,1.93,1.64,3.11,1.19,2.08,1.09,1.43,0.95,0.28,1.53,3.08,1.34,1.6,1.1,0.82,1.26,1.51,1.05,1.826470588,1.354,0.012612716,0.155067106

26057,ANKRD17,12.25,11.49,12.77,18.2,16.58,16.05,11.47,12.6,10.37,14.16,15.27,12.17,10,14.14,12.3,12.45,13.81,10.15,8.9,7.44,15.69,12.18,5.46,16.84,12.15,12,8.1,12.53,8.66,8.98,12.78,7.92,13.29882353,10.652,0.012612716,0.155067106

79850,FAM57A,13.48,10.13,13.28,20.41,21,13.62,21.67,20.68,14.79,13.35,16.22,22.09,22.25,22.42,16.84,11.38,16.8,11.01,17,17.18,18.75,11.84,1.88,7.05,13.07,9.31,14.25,11.7,13.61,11.48,16.9,11.62,17.08294118,12.44333333,0.012612716,0.155067106

26115,TANC2,17.38,5.24,4.88,4.78,7.05,4.82,4.64,9.76,6.74,6.71,6.65,8.69,8.44,12.21,7.87,3.84,6.26,5.09,1.04,5.3,4.07,5.36,0.5,4.18,7.63,5.38,4.51,8.12,5.25,3.59,6.36,1.77,7.409411765,4.543333333,0.012612716,0.155067106

60561,RINT1,8.27,7.06,7.7,8.61,7.94,9.77,8.29,8.45,5.98,12.02,8.16,9.31,9.25,8.74,10.6,6.16,10.15,6.66,6.55,6.09,10.05,9.21,6.26,7.12,7.21,8.53,5.85,6.36,5.57,5.31,9.2,6.86,8.615294118,7.122,0.012612716,0.155067106

26173,INTS1,23.38,19.41,18.07,30.53,32.13,24.83,12.24,20.93,20.08,20.74,28.06,19.29,14.07,25.91,21.31,20.49,27.11,16.71,11.79,16.13,26.62,17.28,5.31,29.2,17.58,14.06,19.72,18.11,15.1,16.02,25.55,13.02,22.26941176,17.48,0.012612716,0.155067106

6120,RPE,15.25,9.66,15.77,20.84,17.54,17.68,22.54,15.54,14.71,16.58,17.4,16.18,17.43,15.45,9.2,14.57,16.21,16.01,9.89,15.89,17.24,15.09,8.67,13.18,14.63,10.85,11.87,12.32,14.66,15.63,14.95,15.12,16.03235294,13.73333333,0.012612716,0.155067106

81603,TRIM8,38,32.89,21.78,89.11,101.51,29.1,24.08,42.79,30.71,25.99,24.7,30.15,30.06,45.68,23.04,27.44,22.13,32.08,34.86,58.98,65.7,57.13,23.08,27.72,58.7,81.07,54.83,38.33,46.49,59.17,37.1,53,37.59764706,48.54933333,0.012612716,0.155067106

7355,SLC35A2,18.56,14.09,15.24,12.64,13.18,12.96,13.81,15.14,11.82,13.82,21.28,13.17,7.48,16.85,15.67,11.24,16.22,13.24,19.96,8.31,13.57,12.9,11.8,10.54,12.08,11.46,11.35,11.67,10.94,9.15,15.49,11.07,14.30411765,12.23533333,0.012612716,0.155067106

203068,TUBB,557.96,298.53,402.37,624.2,541.82,472.97,344.23,471.22,410.6,652.17,521.4,431.22,471.75,625.33,445.67,441.28,845.71,440.74,186.82,350.4,576.64,437.65,112.65,273.54,319.08,413.71,373.98,446.78,460.83,336.95,649.81,220.24,503.4370588,373.3213333,0.012612716,0.155067106

3181,HNRNPA2B1,140.72,79.26,155.74,194.19,189.81,150.94,84.61,152.96,137.05,88.07,154.07,74.07,66.94,172.79,142.68,116.1,171.33,74.41,79.82,75.58,186.19,78.44,126.9,113.79,113.86,108.77,68.64,111.28,88.09,61.5,135.87,62.15,133.6076471,99.01933333,0.012612716,0.155067106

55276,PGM2,10.17,7.32,6.54,15.16,17.8,7.65,7.17,7.69,9.7,11.58,10.61,9.26,10.04,12.49,10.77,5.5,13.27,7.05,10.37,2.76,20.29,20.85,2.33,8.03,6.46,3.54,6.13,6.48,6.07,3.68,8.52,3.86,10.16,7.761333333,0.012612716,0.155067106

10404,CPQ,16.21,7.08,16.28,9.03,19.91,43.73,33.71,29.33,30.51,15.94,37.55,0.22,24.39,23.56,11.38,21.42,17.6,31.18,42.32,57.42,12.1,23.43,9.37,81.15,50.46,18.65,46.82,23.11,29.17,41.61,34.73,66.6,21.05,37.87466667,0.012612716,0.155067106

1478,CSTF2,6.74,7.67,8.2,7.96,7.87,11.7,4.36,5.8,6.64,5.03,7.47,7.37,7.38,7.73,5.58,5.64,12.15,6.67,3,4.77,11.42,8.91,1.81,7.26,5.27,6.49,4.68,5.38,4.87,5.14,6.3,3.33,7.37,5.686666667,0.012612716,0.155067106

54881,TEX10,6.63,5.58,6.45,8.56,7.83,7.4,5.62,9.16,5.96,6.39,4.19,9.27,7.82,10.12,6.68,10.97,8.1,5.76,5.81,4.79,8.22,4.68,1.83,6.47,6.19,9.17,5.01,6.36,5.95,4.82,7.11,5.55,7.454705882,5.848,0.012612716,0.155067106

51271,UBAP1,17.86,15.07,12.92,15.62,20.53,23.36,17.76,18.51,17.55,14.93,10.93,19.17,14.21,16.7,13.84,11.28,12.55,26.11,16.48,15.27,21.7,22.26,11.95,29.55,20.31,25.52,17.15,15.54,19.43,18.92,18.26,20.6,16.04647059,19.93666667,0.012612716,0.155067106

3398,ID2,6.14,2.25,25.77,3.7,1.53,5.02,18.17,14.9,8.45,4.2,27.75,3.35,2.24,11.32,2.85,6.25,33.78,34.6,171.99,106.04,6.04,11.35,142.15,20.51,27.31,9.64,22.75,2.65,19.72,59.9,3.71,10.39,10.45117647,43.25,0.012612716,0.155067106

116540,MRPL53,29.76,37.89,22.51,34.8,32.14,39.32,17.5,42.14,23.25,31.24,24.59,23.58,20.01,30.09,15.44,26.24,25.72,30.08,32.71,30.85,51.35,19.45,27.72,56.3,38.95,44.16,28.24,52.65,27.96,35.44,43.2,40.32,28.01294118,37.292,0.012612716,0.155067106

57648,KIAA1522,10.96,8.11,13.67,13.77,9.17,5.54,7.33,9.5,14.51,6.59,7.26,12.7,11.45,8.56,2.94,10.29,19.46,14.01,1.77,5.74,8.37,9.69,4.49,16.55,6.31,2.16,3.76,2.39,5.9,4.72,8.97,2.75,10.10647059,6.505333333,0.012612716,0.155067106

9572,NR1D1,6.25,1.83,2.65,5.26,7.14,10.42,3.33,7.59,5.84,2.03,3.58,2.23,3.99,2.82,1.31,5.15,3.05,6.95,22.34,9.96,3.21,4.69,1.35,10.01,17.35,3.8,7.69,6.33,8.54,8.84,3.5,8.63,4.380588235,8.212666667,0.012612716,0.155067106

25901,CCDC28A,8.85,6.89,4.59,6.15,5.49,5.14,4.47,7.18,5.04,5.7,4.25,4.78,3.98,3.22,4.94,4.43,3.69,6.28,9.45,8.16,7.74,5.39,10.41,19.42,5.86,2.91,6.88,6.45,5.62,5.6,4.64,6.07,5.222941176,7.392,0.012612716,0.155067106

5464,PPA1,25.89,33.99,26.02,17.85,10.61,35.43,55.78,32.29,43.35,47.19,27.32,21.58,30.64,39.09,15.82,16.81,47.49,39.45,28.99,50.63,77.55,60.96,106.16,39.6,33.79,50.46,30.16,54.91,27.23,35.7,39.85,33.7,31.00882353,47.276,0.012612716,0.155067106

5098,PCDHGC3,30.63,28.57,9.77,10.91,18.24,25.54,29.13,65.46,23.98,21.55,56.19,14.33,4.15,13.92,5.38,22.64,19.37,49.3,74.06,67.3,6.79,3.22,12.35,100.04,57.63,24.94,40.63,146.99,33.62,50.29,65.59,47.13,23.51529412,51.992,0.012612716,0.155067106

1318,SLC31A2,4.1,1.48,1.77,2.87,4.18,2.72,3.6,3.02,1.82,2.99,2.91,2.4,3.73,4.89,1.67,4.11,2.82,3.22,2.5,4.7,3.03,8.89,15.49,5.81,8.88,18.71,8.12,2.64,4.42,12.52,2.62,2.41,3.004705882,6.930666667,0.012612716,0.155067106

718,C3,0.3,0.14,0.02,0.04,0.14,0.22,41.66,0.13,0.03,0.13,0.16,143.02,21.43,4.29,0.3,0.03,0.04,0.2,3.3,8.65,0.55,0.46,1731.55,0.03,20.2,2.97,0.39,4.66,2.85,68.24,0.44,82.28,12.47529412,128.4513333,0.01262519,0.155067106

387590,TPTEP1,0.05,0.08,0.1,0.15,0.22,0,0.05,0,0,0.16,0.23,0.12,0,0.07,0,0.1,0.25,0.05,0.18,3.06,2.06,0.18,0,0.24,0.31,1.61,0,0,0.54,3.3,2.33,0.9,0.092941176,0.984,0.012627115,0.155067106

25788,RAD54B,2.08,2.24,1.19,1.67,0.89,2.41,0.77,1.67,1.13,0.94,2.28,1.58,1.38,2.84,1.48,1.67,2.86,1.37,0.61,1.34,2.3,1.36,0.23,3.11,0.96,0.5,0.54,0.76,0.75,1.15,1.34,1.12,1.710588235,1.162666667,0.012649628,0.155067106

25854,FAM149A,0.44,0,0.05,0.1,0,0.45,0.58,0.51,1.02,0.04,1.58,0.56,0.2,0,0.86,0.27,0.83,0.69,0.96,0.7,0.13,0.9,1.39,3.21,0.24,0.54,0.78,0.24,0.82,1.3,0.93,2.02,0.440588235,0.99,0.012649628,0.155067106

256356,GK5,1.96,1.81,2.98,2.08,1.5,1.92,2.67,3.05,3.46,2.66,3.27,2.12,2.46,3.52,3.43,2.95,3.14,1.63,3.46,1.63,1.09,1.16,0.52,1.75,2.28,1.54,2.01,3.2,1.72,1.82,3.27,1.88,2.645882353,1.930666667,0.012665932,0.155067106

6877,TAF5,1.87,1.48,1.45,2.99,2.22,1.74,1.15,1.94,1.28,1.29,1.09,2.1,0.8,1.74,1.19,1.68,2.64,0.69,0.96,1.23,2.95,1.11,1.36,1.89,1.19,1.01,0.75,1.59,1.28,1.05,0.95,1.03,1.685294118,1.269333333,0.012665932,0.155067106

11215,AKAP11,7.51,9.08,6.89,10.28,10.11,10.09,8.1,8.62,6.89,12.08,7.78,10.94,12.27,11.5,8.45,8.06,10.11,5.71,6.18,7.73,6.05,7.22,1.81,10.33,9.84,7.18,7.52,12.12,6.3,7.06,8.65,7.68,9.338823529,7.425333333,0.012674088,0.155067106

56929,FEM1C,3.72,8.16,4.28,6.18,6.16,5.54,6.09,5.8,4.83,4.69,5.98,11.57,7.43,9.45,6.86,7.38,4.72,4.11,3.56,5.07,4.51,6.1,2.95,5.58,5.18,5.8,4.28,5.96,4.8,4.56,6.25,3.79,6.402352941,4.833333333,0.012674088,0.155067106

23185,LARP4B,8.35,4.96,6.15,9.41,9.98,8.2,5.95,7.7,7.1,6.76,6.92,5.61,5.04,9.33,6.23,6.35,9.55,5.71,5.05,4.89,7.86,4.96,2.39,9.03,5.25,7.38,5.45,5.72,5.61,5.64,7.01,4.61,7.27,5.770666667,0.012674088,0.155067106

286410,ATP11C,4.58,10.48,7.98,26.01,22.41,9.91,11.76,19.43,15.87,13.01,5.94,11.99,21.2,6.67,3.6,5.31,5.2,4.03,7.06,5.24,8.98,5.22,2.55,4.83,7.36,2.86,6.39,6.05,6.27,6.39,6.15,10.24,11.84411765,5.974666667,0.012682246,0.155067106

5411,PNN,31.61,15.45,24.71,45.53,42.66,15.45,20.17,35.76,27.58,26.15,20.21,15.79,13.12,47.91,28.5,41.01,38.76,16.1,23.85,11.22,21.48,16.01,15.18,24.6,31.03,14.89,12.91,31.25,14.84,10.54,32.02,14.15,28.84529412,19.338,0.012682246,0.155067106

6683,SPAST,7.02,4.49,4.36,8.99,8.22,6.14,5.74,7.34,6.26,5.3,6.32,5.34,5.95,5.61,3.98,5.17,4.45,4.46,3.2,3.96,6.82,4.28,1.93,5.91,4.56,4.64,4.01,5.39,4.64,4.06,6.59,4.26,5.922352941,4.580666667,0.012682246,0.155067106

23049,SMG1,8.96,5.67,8.51,14.69,12.34,10.05,8.13,10.97,11.16,6.76,7.83,9.47,8.61,8.5,7.04,8.99,10.66,6.42,6.02,7.07,9.74,6.1,3.79,12.94,8.02,6.89,8.53,6.02,7.59,8.6,7.63,6.4,9.314117647,7.450666667,0.012682246,0.155067106

29058,TMEM230,61.51,39.84,49.99,68.68,63.63,76.44,63.54,59.11,44.01,53.9,67.93,67.93,72.84,63.33,33.04,33.35,44.79,73.07,67.3,80.05,68.33,70.68,50.37,109.39,65.35,73.01,58.07,57.45,68.32,64.68,68.29,72.62,56.69764706,69.79866667,0.012682246,0.155067106

8605,PLA2G4C,0.69,0.02,0.13,13.08,8.41,0.03,1.29,0.97,0.57,0.26,0.27,0.37,0.68,2.11,0.1,0.09,0.54,0.22,3.27,2.16,12.39,25.06,0.66,2.17,0.12,5.82,2.27,0.66,1.98,2.1,1.43,4.53,1.741764706,4.322666667,0.012682246,0.155067106

7200,TRH,0.07,0,0,0,0,0,6.85,0,0,0,0,0.13,24.89,0,0.04,0,0,0.08,1.37,0.36,0.03,0,0,0,0.1,0.04,55,0.06,1.08,55.26,0.06,0.43,1.881176471,7.591333333,0.012702452,0.155216793

729176,KATNBL1P6,0.07,0.14,0.03,0.11,0.04,0,0,0,0.04,0,0.04,0,0,0.14,0,0.1,0.17,0.04,0,0,0,0.04,0,0,0,0,0,0,0.03,0,0,0,0.051764706,0.007333333,0.012768902,0.155931021

89792,GAL3ST3,1.08,0.09,0.64,0.07,0,0.02,0.16,0.98,0.98,0.06,0.6,0.51,0.03,0.15,0.31,0.43,0.16,7.38,0.24,0,0.23,0,0,0,0.12,0.02,0.05,0.02,0.02,0,0.34,0,0.368823529,0.561333333,0.012809604,0.156330109

100652770,DSG2-AS1,0.66,0,0.28,0.93,1.89,0,0.08,0.21,1.11,0.19,0,0,0.14,0.98,1.18,0.88,1.47,0.51,0,0,0,0,0.21,0.09,0.25,0,0.17,0.03,0,0.03,0.13,0,0.588235294,0.094666667,0.012889216,0.157203261

7066,THPO,0.04,0,0,0,0,0,0,0.23,0,0,0.08,0,0,0.11,0.29,0.12,0,0.04,0.68,0.31,0,0,6.12,0,0.06,0.18,0.05,0.07,0.22,0.83,0.07,0.63,0.051176471,0.617333333,0.01293988,0.157722491

58528,RRAGD,0.04,0,0.06,0.05,0.05,0.03,0,0,0.02,0.04,3.67,0,0.02,0.25,0.02,0.34,0.02,1.93,0.62,0.03,0.29,0,2.93,29.06,0.04,4.85,0.07,0.12,0.24,0.07,0.19,0.02,0.271176471,2.697333333,0.012984913,0.158088679

312,ANXA13,0,0,0.09,0,0,0,0,0,0,0,0,0,0,0,0,0,0,0.16,0.05,0,0.05,0,1.63,0,0.07,0,0,0,0.05,0,0.04,0,0.005294118,0.136666667,0.012986145,0.158088679

4888,NPY6R,0,0,0,0,0,0,0,0,0,0,0,0,0,0.06,0,0,0,0,0.11,0,0,0,0.05,0,0,0.65,0.03,0.02,0,0,0.02,0.03,0.003529412,0.060666667,0.013014902,0.158218617

8999,CDKL2,0.11,0,0.17,0.52,0.2,0.16,0.03,0.19,0.41,0.04,0.36,0.14,0.13,0.51,0.15,0.34,0.3,0.52,0.01,0,0.1,0.05,0,1.05,0.04,0.03,0,0.04,0.01,0,0.22,0,0.221176471,0.138,0.013026813,0.158218617

56961,SHD,0,0,0,0,0,0,0,0,0,0,0,0,0,0.86,0,0,0,0,0,0,0,0,0.05,0.03,0,69.53,0,0.22,9.5,0.15,0.05,0,0.050588235,5.302,0.013029291,0.158218617

146862,UNC45B,0,0,0,0,0,0,0,0,0,0,0,0,0,0.8,0,0,0,0.07,0,0,0,0,0,0,0.03,39.37,0,0.09,2.27,0.03,0.02,0,0.047058824,2.792,0.013029291,0.158218617

51458,RHCG,0,0,0.03,0,0.04,0,0,0,0,0.05,0.08,0,0,0,0,0,0.05,0.08,0,0,0,0.18,0,0.09,0.25,0.67,0,0.1,0.07,0.28,0.1,0,0.014705882,0.121333333,0.013194459,0.160124533

5454,POU3F2,0,0,0,0,0,0,0,0,0.02,0,0,0,0,0,0,0.02,0.02,7.23,0,0,0,0,0,0.04,0.12,0.14,0,0.03,0.08,0.02,0.17,0,0.003529412,0.522,0.013248851,0.16029143

144347,FAM101A,1.15,0.86,0,0,0.07,0.26,11.57,3.63,1.65,0.04,1.15,0,0.13,2.21,0.04,0,0.47,35.36,3.96,2.25,10.66,21.53,0.13,0.24,2.56,1.21,5.04,1.18,0.78,0.28,0.51,0.89,1.366470588,5.772,0.013271039,0.16029143

165140,OXER1,0.28,0.13,0.28,0.09,0.13,0.04,0,0.55,0,0,0.12,0,0.1,0.38,0,0,0,0.09,0.15,0.18,0.15,0,0.7,0.1,0.76,1.58,0.05,1.19,0.88,0.04,0.52,0.48,0.123529412,0.458,0.013320734,0.16029143

51554,ACKR4,0.26,0.03,0.06,0.14,0.07,0.06,10.67,0.22,0.11,0.09,0,0.23,2.41,0.18,0,0.16,0.11,0,2.06,81.39,2.36,0.66,0.12,0.16,1.51,0.04,0.45,0.59,1.78,25.1,0.4,3.7,0.870588235,8.021333333,0.013321824,0.16029143

101,ADAM8,0.63,0.48,0.47,1.15,0.56,0.45,0.26,0.94,0.78,3.73,1.02,0.72,0.48,1.19,1.57,1.16,1.58,0.28,0.34,0.21,0.09,0.4,0.87,0.98,0.65,0.23,0.45,1.71,0.37,0.11,0.98,0.48,1.01,0.543333333,0.013330297,0.16029143

85015,USP45,1.54,1.31,2.24,3.54,3.17,1.68,2.21,1.96,2.29,1.81,1.69,1.21,1.71,2.92,1.6,1.36,2.19,1,1.6,1.36,1.57,2.55,0.76,2.04,1.65,1.39,1.14,1.36,1.28,1.3,2.28,1.29,2.025294118,1.504666667,0.013338772,0.16029143

55638,SYBU,0.78,0,0.26,2.26,0.06,0.11,0.09,0.18,0.09,0.03,9.66,0.06,0,2.71,2.36,0.25,0.07,1.05,17.09,3.02,0.05,5.64,17.06,0.22,0.26,0.87,0.21,0.22,8.05,3.92,0.65,2.22,1.115882353,4.035333333,0.013338772,0.16029143

4660,PPP1R12B,1.64,1.47,1.09,1.63,2.12,1.38,1.45,1.63,2.45,1.3,2.7,1.28,1.23,1.42,1.26,1.42,1.69,5.62,2.04,1.62,0.93,1.69,1.02,3.08,2.18,13.87,1.95,1.94,1.85,2.98,1.75,3.17,1.597647059,3.046,0.01335573,0.16029143

50619,DEF6,1.44,6.34,3.06,0.33,0.57,2.45,0.37,0.78,0.95,4.99,1.58,1.51,2.55,6.14,2.48,1.32,0.55,0.4,0.81,0.07,0.15,0.38,1.27,1.38,0.55,0.07,0.39,3.56,0.94,0.38,3.42,0.11,2.200588235,0.925333333,0.01335573,0.16029143

8736,MYOM1,0.13,0.11,0.07,0.04,0.04,0.38,0.67,0.22,0.44,0.08,2.07,0.3,0.15,1.49,0.06,0.27,0.19,1.22,0.15,0.34,0.01,0.23,12.14,0.74,0.7,13.73,0.28,0.6,0.7,1.02,0.68,1.1,0.394705882,2.242666667,0.01335573,0.16029143

375484,SIMC1,3.13,7.23,2.68,3.2,3.16,3.46,1.5,3.57,3.48,5.55,2.74,3.66,4.74,9.19,3.42,2.73,5.26,2.17,2.25,2.83,2.28,1.26,1.55,3.48,2.19,1.29,1.82,9.85,3.36,2.8,3.33,3.36,4.041176471,2.921333333,0.013364212,0.16029143

100533107,RTEL1-TNFRSF6B,3.54,3.18,2.12,2.99,3.74,3.86,2.25,5.77,3.34,2.06,2.82,2.12,1.7,4.15,3.5,3.42,4.74,1.7,2.57,2.01,1.97,1.69,0.52,4.91,2.93,1.07,2.08,2.88,2.45,2.13,3.96,1.71,3.252941176,2.305333333,0.013364212,0.16029143

10947,AP3M2,5.57,6.73,5.85,9.2,7.46,7.53,6.31,6.76,6.94,7.8,7.8,5.73,6.62,10.49,9.17,10.92,8.29,5.54,3.58,4.45,6.57,2.92,0.69,25.77,5.36,11.1,6.71,6.62,5.41,5.81,5.28,8.27,7.598235294,6.938666667,0.013364212,0.16029143

374900,ZNF568,1.15,1.16,1.22,1.5,1.27,1.45,1.83,2.23,1.97,1.52,1.99,0.13,0.2,1.2,1.39,1.65,1.53,1.94,1.93,2.58,1.33,1.07,0.58,2.34,2.75,4.16,1.47,2.23,2,2.39,1.96,1.94,1.375882353,2.044666667,0.013364212,0.16029143

100131089,SRP14-AS1,1.13,0.87,0.58,1.33,0.33,1.23,0.55,0.92,0.89,0.83,1,0.54,0.91,0.77,0.37,0.96,0.53,0.78,1.53,1.45,1.54,0.27,0.78,3.14,0.84,3.7,0.99,2.51,1.18,0.91,1.11,1.6,0.808235294,1.488666667,0.013364212,0.16029143

23162,MAPK8IP3,20.84,37.24,26.52,39.29,35.48,18.74,10.04,26.25,21.02,48.23,37.32,14.24,14.93,52.53,37,32.31,18.2,13.83,31.6,10.61,10.48,18.74,5.11,14.77,27.85,18.68,16.2,45.68,8.48,7.84,35.68,10.27,28.83411765,18.388,0.013372697,0.16029143

388272,C16orf87,2.58,4.27,3.45,3.96,5.02,1.9,3.1,3.09,4.47,3.49,5.14,2.4,1.77,5.07,3.71,3.74,5.73,5.03,1.51,2.3,2.98,3.1,2.73,2.88,2.28,4.23,3.47,1.6,3.43,1.59,2.05,1.64,3.699411765,2.721333333,0.013372697,0.16029143

90639,COX19,3.02,3.58,4.16,3.06,3.52,2.92,3.08,3.99,3.59,2.73,2.56,2.83,2.62,4.23,4.97,3.16,4.77,2.3,2.33,2.87,3.22,2.12,0.88,3.93,2.32,2.25,2.11,2.95,3.9,2.96,2.73,3.67,3.458235294,2.702666667,0.013372697,0.16029143

79768,KATNBL1,6.38,5.76,5.47,11.53,8.3,5.56,7.17,7.02,6.36,6.77,5.33,4.53,5.83,7.64,5.38,4.7,8.15,5.9,4.07,4.38,8.67,7.14,3.05,4.46,5.39,4.13,4.87,5.49,4.83,5.25,6.68,4.53,6.581176471,5.256,0.013372697,0.16029143

2202,EFEMP1,59.06,92.98,30.22,67.97,121.44,90.92,440.65,17.13,4.92,72.61,40.26,79.07,201.56,95.07,40.67,1.61,3.77,258.28,1250.69,447.85,1861.23,1576.84,4.93,0.71,445.7,46.19,318.49,95.07,130.95,806.91,26.8,797.35,85.87705882,537.866,0.013372697,0.16029143

117144,CATSPER1,0,0.09,0.02,0.23,0.03,0.13,0,0.37,0.16,0.29,0.11,0.03,0.07,0.07,0.07,0.17,0.35,0,0.03,0.09,0.11,0.07,0,0.03,0.04,0.06,0.03,0.05,0,0,0.02,0.03,0.128823529,0.037333333,0.01338101,0.160292493

57468,SLC12A5,0.05,0.04,0.05,0.53,0.41,0,0.05,0.03,0.39,0.03,0.01,0.08,0.77,0.32,0.1,0.01,0.01,0.04,0.01,0,0.07,0.03,0.02,0.01,0.06,0,0,0,0,0.02,0.06,0.03,0.169411765,0.023333333,0.013475977,0.161330949

1016,CDH18,2.35,0.75,1.4,0.03,0,0.07,0.35,4.2,0.06,0,19.56,0.17,0.73,0.32,0.03,0.31,0.27,0.08,0,0.05,0,0.03,0,0,0.16,0.03,0.06,0.22,0.38,0.07,0.24,0,1.8,0.088,0.013510585,0.161645978

283102,KRT8P41,0.04,0.03,0,0,0,0,0,0,0,0.1,0.04,0,0,0,0,0.04,0.05,0,0,0,0,0,0,0,0,0,0,0,0,0,0,0,0.017647059,0,0.01354115,0.161912274

11075,STMN2,0,0,0,0,3.44,0.03,0.03,0,0,0,8.21,0,7.87,247.14,0.04,0.03,0,0.67,0.39,180.94,0,0,0.95,0.19,53.46,3.87,0.07,1.58,0.24,23.12,31.01,3.61,15.69352941,20.00666667,0.013586666,0.162356915

23217,ZFR2,0,0,0.07,0,0,0,0,0,0.2,0,0,0,0.11,0.23,0,0.09,0.22,0,0,0,0,0,0,0,0,0,0,0,0,0,0,0,0.054117647,0,0.013615197,0.162527867

1812,DRD1,0,0.04,0.02,0,0,0,0.02,0,0,0.22,0.02,0,0,0,0,0,0.08,0.69,0,0.44,0,0.03,0.04,0,0.12,0,0.05,0,0.27,0.53,0.41,1.29,0.023529412,0.258,0.013764602,0.162527867

55321,TMEM74B,0.09,0.07,0.07,0.18,0.14,0.46,0,0.15,0.15,0.67,0.04,0.3,0.21,0.23,0.46,0.09,0.21,0,0.67,0.05,0.04,0.1,0.08,0.05,0,0.14,0,0.11,0.04,0,0.11,0.15,0.207058824,0.102666667,0.013788882,0.162527867

6588,SLN,0,0,0,0.22,0,0,0,0,0,0.82,0,0,0,0.56,0,0,0,0.45,0,0.11,0,0,0,0.12,0.14,295.07,0.48,0.65,0.38,0.19,0,4.12,0.094117647,20.114,0.013794648,0.162527867

256355,RPS2P32,0.07,0,0.06,2.68,1.55,0,0.08,0.74,0.76,0.09,0,0,0.09,0.19,0.09,0.22,0.09,0.7,0.67,0.55,2.36,1.22,0,0.6,0.79,1.38,0.33,0.58,0.26,0.46,0.25,0.43,0.394705882,0.705333333,0.013841344,0.162527867

4619,MYH1,0,0,0,0,0,0,0.03,0,0,0,0,0,0,0.05,0,0,0,0.03,0,1.12,0,0,0,0,0,17.78,0.01,0.02,0.12,2.39,0,0.03,0.004705882,1.433333333,0.013907629,0.162527867

29841,GRHL1,0.08,0.05,0.07,0.04,0.13,0.04,0.17,0.11,0.09,0.03,0.14,0.05,0.1,0,0.02,0.04,0.05,0.24,0.11,0.81,0.21,0.24,3.67,0,0,0.13,0.3,0.29,0.04,0.52,0.07,0.12,0.071176471,0.45,0.013911433,0.162527867

7125,TNNC2,0,0.18,0.19,0.12,0,0.55,0.12,0.25,0,0.43,0.11,0.91,0.27,18.39,0.13,0.22,0.28,0.96,0.72,2.67,0.21,0,0,0.26,0.3,171.55,0.88,1.49,4.15,2.14,0.48,1.06,1.302941176,12.458,0.013920205,0.162527867

26137,ZBTB20,0.09,0.11,0.02,0.16,0.16,0.11,0.1,0.26,0.18,0,0.15,0.08,0.17,0.26,0.13,0.22,0.08,0.18,0.26,0.31,0.17,0.16,0.25,0.18,0.03,0.72,0.22,0.14,0.22,0.4,0.23,0.1,0.134117647,0.238,0.01392898,0.162527867

9976,CLEC2B,0,5.67,0.03,7.78,3.44,2.37,0.08,0,0,0.23,0.88,0,1.02,1.62,0.09,0.32,0,0.15,0.13,14.41,9.23,6.78,2.4,1.31,2.07,3.27,0.16,3.91,0.06,7.83,2.09,3.93,1.384117647,3.848666667,0.01392898,0.162527867

55195,C14orf105,0,0,0,0,0.07,0,0.06,0,0.03,0,0.03,0,0,0.08,0,0.03,0,0.09,0,0.03,0.05,0,2.35,0.03,0.12,0.07,0.1,0.05,0,0,0.05,0.1,0.017647059,0.202666667,0.013964823,0.162527867

285548,LINC01096,0.21,0,0.07,0,0,0.57,0,0,0,0.22,0.65,0.39,0.26,0.11,0.1,1.26,0.11,0,0.35,0,0,0,0,0,0,0.05,0,0,0,0.04,0.11,0,0.232352941,0.036666667,0.013971669,0.162527867

9547,CXCL14,0,0.03,0.03,0,0,0,0,0,0,0.1,0,0,0.09,0,0,0,0.14,16.2,2.45,0.12,0,0,0,0,0.5,0.08,0,0.13,0.13,0.07,0.32,0.04,0.022941176,1.336,0.013992303,0.162527867

90423,ATP6V1E2,0.4,0.41,0.16,0.11,0.35,0.6,0.15,0.49,0.51,0.47,0.41,1.31,0.41,0.48,0.39,0.22,0.45,0.16,0.63,0.79,0.55,0.78,0.77,1.5,0.44,0.36,0.58,0.29,0.95,1.43,0.47,0.78,0.430588235,0.698666667,0.014043273,0.162527867

57116,ZNF695,0.31,0.29,0.27,0.51,0.6,0.15,0.09,0.14,0.43,0.54,0.25,0.79,0.61,0.97,0.48,0.72,0.3,0.09,0.2,0.5,1.11,0.29,0.08,0.21,0.07,0.09,0.05,0.27,0.12,0.04,0.92,0.13,0.438235294,0.278,0.014052082,0.162527867

30832,ZNF354C,0.4,2.13,0.58,0,0,0.03,1.96,3.28,4.54,1.71,1.85,0,0.04,0.04,1.79,1.22,1.87,1.44,2.78,2.63,1.99,0.95,0.06,2.68,3.13,1.74,2.15,4.41,2.26,2.58,3.79,1.43,1.261176471,2.268,0.014060894,0.162527867

6347,CCL2,121.66,0.17,0,0,0.65,9.45,176.24,77.38,20.11,0.4,0.83,70.8,86.9,0,0.24,0.1,5.72,257.03,34.35,37.87,278.34,294.2,2.33,2.41,136.84,59.79,5.64,57.88,18.28,29.87,12.97,249,33.56764706,98.45333333,0.014069708,0.162527867

140685,ZBTB46,1.27,2.92,3.19,16.71,21.14,5.21,0.43,0.98,3.52,2.75,2.6,0.97,0.51,6.2,2.39,6.71,4.13,1.39,0.53,0.97,1.75,0.48,0.34,0.98,0.17,1.99,0.17,8.51,1.18,0.7,4.98,1.47,4.801764706,1.707333333,0.014078524,0.162527867

747,DAGLA,1.94,1.94,2.81,5.11,5.36,1.69,1.42,2.37,3.6,2.9,3.36,1.11,1.2,2.17,3.14,2.78,4.7,2.31,0.36,2.24,1.84,2.53,0.31,0.87,2.09,2,1.86,1.49,1.39,2,2.14,1.51,2.8,1.662666667,0.014087343,0.162527867

23148,NACAD,6.77,4.82,2.81,2.94,3.47,2.29,3.43,4.89,6.22,5.19,15.92,6.79,4.32,2.56,3.27,5.09,5.4,5.54,3.12,2.29,1.08,1.38,0.09,3.71,2.32,1.5,1.25,3.83,3.81,3.27,8.49,2.6,5.069411765,2.952,0.014087343,0.162527867

80778,ZNF34,2.37,2.35,1.92,2.16,3.56,4.24,0.69,3.54,2.5,2.06,2.6,1.77,1.13,1.82,2.17,1.66,3.35,1.66,1.18,1.77,1.63,1.04,0.65,4.49,1.94,1.6,1.72,1.62,2.12,1.35,2.25,1.76,2.346470588,1.785333333,0.014087343,0.162527867

8226,PUDP,4.86,15.62,9.63,87.34,67.35,15.07,5.36,10.32,6.95,20.1,6.98,9.74,11.32,44.89,3.37,6.95,8.08,3.82,4.5,6.26,22.16,10.56,2.39,7.1,5.46,9.85,4.63,8.59,3.32,6.14,6.68,3.58,19.64294118,7.002666667,0.014096164,0.162527867

283981,LINC00685,3.03,2.03,3,4.74,4.69,1.86,1.43,2.95,3.46,2.61,1.58,2.08,2.29,3.08,2.44,6.41,3.88,1.79,2.71,1.75,0.51,1.41,0.19,5.04,3.16,1.12,1.9,1.79,1.66,1.72,3.72,0.96,3.032941176,1.962,0.014096164,0.162527867

7768,ZNF225,2.23,2.18,1.47,2.65,2.43,1.89,1.71,2.41,2.2,2.11,2.85,2.06,1.76,1.6,2.1,2.18,2.03,1.14,1.42,1.1,1.83,0.74,0.26,2.66,2.08,1.07,1.12,2.99,1.49,1.57,2.48,1.56,2.109411765,1.567333333,0.014096164,0.162527867

94009,SERHL,4.21,2.66,3.86,3.84,2.95,4.19,1.5,1.93,2.57,3.09,4.17,2.47,1.38,2.34,3.85,3.94,7.9,2.55,1.23,2.61,2.13,4.01,1.02,3.46,1.4,2.36,0.91,2.03,1.54,1.3,1.3,4.61,3.344117647,2.164,0.014096164,0.162527867

9063,PIAS2,7.88,3.21,6.56,7.01,6.2,5.01,4.27,4.52,4.2,5.56,5.96,5.4,4.62,10.23,4.52,4.96,4.02,5.13,4.58,4.43,4.93,2.52,2.23,4.21,4.74,5.7,3.3,3.98,3.56,3.44,5.59,2.77,5.537058824,4.074,0.014096164,0.162527867

317,APAF1,4.08,6.84,5.89,9.19,6.61,5.49,3.11,4.08,4.02,6.23,3.17,3.77,5.01,6.96,6.82,4.42,4.62,3.68,3.41,3.03,5.27,5.37,1.54,4.32,2.99,3.35,3.31,5.17,4.64,3.3,6.11,2.42,5.312352941,3.860666667,0.014096164,0.162527867

54665,RSBN1,2.38,2.74,2.08,3.1,3.57,3.27,1.94,3.27,2.61,3.36,3.22,3.51,2.5,2.79,2.02,3.35,3.12,1.91,2.31,2.01,2.63,1.28,1.47,4.4,2.48,1.22,2.13,3.72,2.18,1.89,2.59,2.36,2.872352941,2.305333333,0.014096164,0.162527867

996,CDC27,25.61,12.55,14.7,19.7,17.22,17.19,18.26,27.56,14.04,15.75,14.65,15.52,15.75,20.54,14.56,11.87,18.19,11.24,12.27,12.57,23.22,17.88,4.73,11.23,15.28,17.48,11.26,11.91,13.11,13.33,17.46,11.55,17.27411765,13.63466667,0.014096164,0.162527867

4213,MEIS3P1,4.41,6.11,2.64,0.32,1.08,3.18,5.4,6.8,3.15,2.5,6.6,0.03,0.03,2.07,0.76,1.13,4.11,5.42,6.08,5.65,3.07,0.09,0.24,11.06,6.56,3.55,6.87,5.07,7.41,10.73,5.97,7.94,2.96,5.714,0.014096164,0.162527867

257194,NEGR1,2.31,0.05,1.31,0.11,0.04,0.84,6.05,1.83,1.3,1.5,1.06,1.27,3.73,0.14,0.61,0.04,3.13,1.56,30.61,10.29,0.86,9.45,0,0.48,14.77,4.89,8.77,1.2,1.61,3.3,6.37,3.98,1.489411765,6.542666667,0.014096164,0.162527867

1646,AKR1C2,0.42,1.02,0.11,0.41,0.36,3.41,3.21,3.94,0.24,0.91,5.33,2.35,3.06,0.15,0.31,0.77,0.38,0.54,18.07,16.45,0.1,1.87,35.52,0.19,23.88,6.95,1.53,2.38,2.54,20.22,5.71,19.88,1.551764706,10.38866667,0.01414301,0.162527867

51477,ISYNA1,29.33,48.74,37.28,43.15,60.15,11.03,16.37,32.76,32.03,44.41,15.56,8.71,8.51,71.72,23.72,39.49,15.97,35.38,10.89,8,23.41,18.94,1.93,27.08,16.61,8.37,4.75,38.8,9.87,7.04,33.93,14.6,31.70176471,17.30666667,0.01414301,0.162527867

540,ATP7B,2.06,1.96,1.56,6.26,5.79,1.47,2.77,2.18,2.71,2.57,2.26,1.23,2.02,3.6,1.48,1.44,1.28,1.93,0.96,0.79,2.53,2.58,2.55,3.31,1.01,1.03,0.94,1.09,1.46,1.08,1.58,0.91,2.508235294,1.583333333,0.01414301,0.162527867

79921,TCEAL4,57.24,72.51,92.86,139.81,125.47,161.65,56.15,57.32,49.97,99.55,95.58,59.46,44.74,130.11,53.97,55.76,49.66,64.71,41.77,42.44,62.43,49.68,21.91,78.15,64.64,94.04,46.58,53.01,43.22,37.63,58.56,48.89,82.45941176,53.844,0.01414301,0.162527867

91151,TIGD7,2.04,2.75,2.12,2.81,2.35,3.51,2.14,2.26,2.69,2.44,4.79,3.44,2.78,3.41,3.08,3.73,1.5,3.09,1.92,0.92,1.07,1.16,0.85,3.03,2.05,3.34,1.53,3.29,1.31,0.95,2.96,1.94,2.814117647,1.960666667,0.01414301,0.162527867

9295,SRSF11,62.54,41.49,53.32,85.66,81.95,45.55,43.09,63.02,46.63,43.4,45.84,34.74,36.54,81.14,53.39,52.4,48.6,35.7,59.12,34.26,42,32.61,25.72,66.47,50.11,25.3,31.25,55,31.31,30.03,57.51,32.24,54.07647059,40.57533333,0.01414301,0.162527867

10347,ABCA7,2.52,4.12,3.29,5.43,3.34,2.79,2.29,2.28,2.1,3.97,5.12,1.33,0.59,7.66,4.24,4.72,1.14,1.4,0.41,1.12,2.11,1.75,1.54,12.99,5.19,1.57,1.6,1.66,0.54,0.62,2.16,1.48,3.348823529,2.409333333,0.01414301,0.162527867

2052,EPHX1,9.78,8.96,10.61,10.47,20.44,19.85,11.55,9.28,8.85,9.94,114.4,50.53,20.67,13.45,11.08,12.52,10.05,21.49,20.02,65.06,13.22,26.9,10.34,138.15,79.79,9.96,15.1,40.65,10.84,56.26,13.09,56.4,20.73117647,38.48466667,0.01414301,0.162527867

9757,KMT2B,8.38,6.89,7.28,15.51,17.31,10.03,4.65,8.92,9.64,8.72,7.67,6.99,6.22,12.02,8.24,8.99,10.63,5.07,7.3,5.83,7.63,5.4,3.37,13.22,8.34,4.81,6.02,11.02,6.18,6.09,8.85,5.57,9.299411765,6.98,0.01414301,0.162527867

57787,MARK4,10.39,15.59,11.35,21.47,21.66,13.74,8.31,9.35,10.12,16.74,15.02,12.58,13.25,12.09,11.12,9.77,8.54,9.93,6.52,9.89,12.17,11.61,5,13.18,10.72,8.13,7.45,16.13,6.98,7.31,12.04,8.05,13.00529412,9.674,0.01414301,0.162527867

23005,MAPKBP1,7.79,6.03,4.46,12.89,10.94,7.38,3.46,5.23,5.92,6.07,11.51,4.9,5.43,4.49,18.52,8.17,11.83,6.32,3.29,3.61,8.02,5.78,0.91,9.01,6.79,2.56,3.5,5.08,3.94,2.68,6.08,2.38,7.942352941,4.663333333,0.01414301,0.162527867

79415,C17orf62,52.24,40.96,36.65,31.95,33.3,35.73,19.73,47.96,24.32,32.87,32.81,48.96,32.3,29.97,32.2,28.31,26.08,21.69,29.4,25.97,23.42,18.83,31.9,36.16,43.45,15.58,24.31,25.93,25.99,28.97,34.29,29.89,34.49058824,27.71866667,0.01414301,0.162527867

6929,TCF3,35.38,31.36,31.99,57.36,53.34,31.41,12.6,26.51,28.26,21.51,40.82,28.6,18.81,31.31,37.55,31.87,31.25,23.19,9.96,24.15,38.82,33.7,1.99,17.25,19.75,14.79,18.74,27.37,29.77,22.73,34.85,17.44,32.34882353,22.3,0.01414301,0.162527867

5358,PLS3,84.3,93.98,161.6,281.33,229.07,133.25,171.1,188.55,316.98,147.46,111.02,138.14,123.93,168.68,80.32,68.02,141.31,99.6,34.57,49.96,159.26,204.34,57.24,94.13,92.31,33.65,129.12,58.4,110.96,160.74,119.45,90.19,155.2376471,99.59466667,0.01414301,0.162527867

2033,EP300,14.02,10.26,9.65,19.27,18.44,17.41,8.79,15.24,15.45,10.05,13.06,13.19,9.8,14.96,12.05,14.7,13.81,9.52,8.07,9.27,14.47,8.66,3.5,16.83,12.82,8.16,9.87,13.15,10.84,11.48,14.12,9.55,13.53823529,10.68733333,0.01414301,0.162527867

9231,DLG5,20.94,20.41,14.53,2.54,2.13,13.36,11.34,18.08,19.18,11.02,29.29,18.3,20.16,16.07,14.94,15.17,16.28,16.58,6.42,14.16,5.32,5.1,0.85,8.59,15.11,7.74,13.54,10.59,17.97,13.6,14.95,9.97,15.51411765,10.69933333,0.01414301,0.162527867

28985,MCTS1,3.39,4.14,5.74,5.1,3.92,5.57,2.92,3.72,3.37,6.73,3.86,4.54,4.94,3.89,2.75,2.55,3.74,2.79,3,3.1,4.37,3.96,3.45,4.15,3.6,3.7,2.24,3.36,2.53,2.26,3.67,2.31,4.168823529,3.232666667,0.01414301,0.162527867

23512,SUZ12,10.47,6.72,6.4,11.01,9.46,9.67,7.51,9.24,8.76,7.91,7.24,10.37,7.95,10,5.09,9.33,12.4,6.94,8.32,5.29,10.71,6.01,3.32,10.78,6.6,4.67,5.81,9.09,6.69,5.74,7.73,6.52,8.795882353,6.948,0.01414301,0.162527867

98,ACYP2,2.65,1.53,1.41,3.83,3.88,4.19,4.58,3.48,2.39,4.63,3.04,2.05,2.47,3.14,1.23,2.23,3.17,4.66,3.68,5.2,4.92,4.79,6.72,3.94,3.27,13.22,2.81,2.85,2.84,4.48,2.25,3.1,2.935294118,4.582,0.01414301,0.162527867

84331,FAM195A,5.41,5.82,7.35,7.45,4.74,10.09,2.63,8.14,3.43,10.98,8.49,2.77,5.88,10.7,6.74,5.08,3.59,8.25,12.25,8.75,9.28,11.25,33.77,7.29,7.22,15.11,9.39,8.47,3.79,7.42,5.54,8.7,6.428823529,10.432,0.01414301,0.162527867

54620,FBXL19,10.96,13.3,8.09,13.24,13.91,9.98,5.49,11.16,9.39,10.75,9.64,9.51,7.55,8.1,8.3,7.61,8.59,9.11,3.92,5.31,13.2,7.81,1.63,12.24,8.08,3.08,5.2,8.96,5.79,4.3,12.02,5.07,9.739411765,7.048,0.01414301,0.162527867

90102,PHLDB2,20.22,8.01,9.59,16.75,19.14,29.31,45.26,22.49,31.41,7.74,26.07,32.74,18.42,21.39,31.51,13.05,21.24,13.55,0.86,3.97,21.3,24.4,3.24,1.95,12.94,12.22,24.04,6.7,12.43,10.73,26.85,14.46,22.02,12.64266667,0.01414301,0.162527867

112574,SNX18,9.72,16.98,11,7.84,9.93,10.49,5.4,10.74,10.66,11.38,12.68,14.96,8.21,8.64,9.33,11.22,9.68,21.1,9.9,22.47,11.45,12.56,4,7.78,16.99,22.92,14.02,11.24,11.21,22.19,11.8,23.19,10.52117647,14.85466667,0.01414301,0.162527867

51027,BOLA1,11.22,8.58,5.51,5.65,8.72,13.36,3.46,10.38,7.14,7.29,10.78,7.65,7.47,10.5,7.3,7.71,6.47,9.48,12.29,10.98,9.51,4.43,8.73,22.45,10.57,10.28,10.13,13.82,8.11,10.41,9.5,13.58,8.187647059,10.95133333,0.01414301,0.162527867

10456,HAX1,53.63,27.77,41.64,26.2,27.95,43.37,53.59,43.6,47.19,27.3,36.05,24.23,32.55,27.23,41.48,25.5,46.16,47.11,43.55,53.42,44.42,37.83,69.57,55.34,29.94,46.71,54.72,33.76,60.08,49.57,34.06,40.36,36.79058824,46.696,0.01414301,0.162527867

116151,FAM210B,25.22,21.25,27.12,32.66,34.47,23.99,17.84,22.56,15.02,16.94,30.87,23.58,25.47,22.41,14.79,14.65,9.54,35.23,16.31,35.99,28.39,37.22,12.98,45.97,40.83,25.66,20.66,30.69,22.89,29.97,41.36,31.59,22.25764706,30.38266667,0.01414301,0.162527867

51012,PRELID3B,13.82,5.23,7.36,8.41,7.29,8.68,9.8,9.88,10.63,8.92,10.8,4.17,4.36,9.5,5.99,8.31,11.13,11.54,6.36,12.96,11.87,10.17,11.99,14.56,6.85,12.67,11.79,7.45,10.02,10.24,9.92,10.2,8.487058824,10.57266667,0.01414301,0.162527867

51020,HDDC2,15.7,9.69,12.26,2.71,3.04,19.45,8.35,14.46,12.4,11.18,16.72,15.36,13.36,11.61,10.88,7.91,17.14,20,19.73,13.86,11.13,2.53,2.73,22.92,18.61,28.84,15.48,13.74,18.63,20.43,17.7,25.87,11.89529412,16.81333333,0.01414301,0.162527867

79723,SUV39H2,2.32,1.39,1.73,3.57,2.64,2.38,1.72,2.73,2.15,1.83,1.87,1.85,1.52,3.27,1.68,2.04,4.04,1.46,1.04,1.7,5.05,1.8,1.26,2.55,1.48,1.41,1,1.91,1.62,1.38,2.35,1.65,2.278235294,1.844,0.01414301,0.162527867

51042,ZNF593,16.59,9.96,13.75,15.32,14.11,22.56,11.55,17.04,14.25,13.73,13.14,12.95,18.46,22.48,10.26,9.48,21.18,21.71,14.3,13.2,25.28,28.36,27.73,25.18,15.49,17.32,12.35,16.79,17.45,20.66,22.13,16.27,15.10647059,19.61466667,0.01414301,0.162527867

84296,GINS4,2.45,1.45,1.02,1.32,0.6,4.91,0.62,3.57,1.71,1.04,2.59,0.68,0.96,3.3,1.08,1.22,3.08,0.87,0.31,0.8,3.45,0.81,0.04,0.22,1.37,0.84,0.58,1.06,1.46,0.88,1.1,0.61,1.858823529,0.96,0.01414301,0.162527867

899,CCNF,2.65,2.19,1.9,4.19,3.5,3.72,1.33,3.12,2.78,2.91,2.06,1.72,1.36,8.89,3.73,2.5,6.17,1.14,1.07,1.34,9.2,2.95,0.26,3.42,1.93,1.26,1.24,2.4,2.48,1.25,2.69,0.65,3.218823529,2.218666667,0.01414301,0.162527867

2353,FOS,0.95,0.86,4.5,0,0.29,6.64,0.72,0.73,2.01,0.18,2.47,1.03,0.67,4.85,1.38,0.88,3.85,2.54,377.78,7.02,2.34,44.97,105.73,0.6,2.98,4.8,2.89,1.3,2.33,3.05,0.38,9.74,1.882941176,37.89666667,0.01414301,0.162527867

715,C1R,12.27,154.94,23.71,0.24,0.74,88.11,106.11,24.96,3.89,9.63,270.95,229.27,38.89,27.5,20.5,15.47,3.28,40.38,303.74,277.27,11.62,0.55,730.46,5.89,524.12,90.96,138.21,181.87,154.84,698.7,62.65,942.89,60.61529412,277.61,0.01414301,0.162527867

377841,ENTPD8,0.1,0.3,0.09,0.24,0.67,0.07,0,0.2,0.16,0,0.14,0.42,0,0.09,0.04,0.24,0.04,0,0.13,0,0,0.04,0.53,0.04,0,0,0,0.06,0,0,0.14,0.04,0.164705882,0.065333333,0.014159681,0.162623566

440585,FAM183A,0.3,2.09,0,0.16,0,0,0,0.17,0,0.2,0,0,0,0.6,0.72,1.67,0.38,0,0,0,0,0,0,0.36,0,0,0,0.13,0,0,0,0,0.37,0.032666667,0.014223944,0.163265413

3216,HOXB6,0,0,0,38.23,38.2,0,0.09,0,0,0,0,0,1.69,58.62,0,0,0,0.8,0,2.48,8.04,1.79,1.09,3.74,0.35,2.15,0,17.07,4.2,3.53,22.77,0.16,8.048823529,4.544666667,0.014465972,0.165945728

4604,MYBPC1,0,0,0,0,0,0.04,0,0,0,0.07,0,0,0,1.06,0,0,0,0.08,0,0,0,0,0.04,0.05,0.05,120.78,0.06,0.34,0.03,0.02,0,0.02,0.068823529,8.098,0.014598595,0.167351951

90668,LRRC16B,0.08,0.03,0.03,0,0.03,0.05,0.02,0.07,0.24,0.06,0.11,0.04,0.04,0.04,0,0.11,0.04,0.02,0.03,0.02,0.01,0.02,0,0.48,0.02,0,0.02,0.03,0.04,0,0.05,0,0.058235294,0.049333333,0.01460573,0.167351951

126,ADH1C,0,0,0,0,0,0.44,0,0,0,0,0.21,0,0.06,0,0,0.05,0,0,0.05,0.49,0,0,0.59,0,0.13,0,0,5.79,1,3.45,0.3,0.9,0.044705882,0.846666667,0.014621502,0.167434238

29958,DMGDH,0.21,0,0.08,0,0,0.18,0.35,0.29,1.01,0,0.74,0.14,0.03,0,0.84,0.05,0.03,0.25,0.13,0.94,0.16,0.14,3.42,0.25,0.57,0.42,0.45,0.15,0.93,2.38,0.06,0.67,0.232352941,0.728,0.01465285,0.167694686

202333,CMYA5,0.09,0.13,0.11,0.02,0.02,0.14,0.19,0.09,0.09,0.06,0.15,0.02,0.06,0.46,0.23,0.07,0.05,0.34,2.37,0.97,0.01,0.03,0.06,0.11,1.02,3.66,0.44,1.27,0.32,0.6,0.51,0.08,0.116470588,0.786,0.014744113,0.168120553

80164,PRR36,0.1,0.44,0.4,0.12,0.14,0.02,0.2,0.2,0.11,0.23,0.33,0.13,0.4,0.28,0.15,0.81,0,0.03,0.01,0.12,0.06,0.06,0.03,0,0.15,0.05,0.04,0.2,0.01,0.07,0.37,0.31,0.238823529,0.100666667,0.014762396,0.168120553

79915,ATAD5,0.66,0.24,0.2,0.9,0.55,0.44,0.09,0.62,0.61,0.49,0.14,0.19,0.19,1.59,0.54,0.61,1.35,0.15,0.16,0.36,0.78,0.18,0.18,0.37,0.06,0.18,0.17,0.47,0.29,0.14,0.53,0.17,0.553529412,0.279333333,0.014789838,0.168120553

9708,PCDHGA8,0.86,0.36,0.46,0.73,0.44,0.94,0.33,1.24,0.23,0.31,1.16,0.53,0.64,0.59,0.31,0.41,0.14,0.36,0.46,0.7,0.12,0.25,0.26,0.16,0.38,0.07,0.35,0.14,0.22,0.16,1.17,0.29,0.569411765,0.339333333,0.014817302,0.168120553

54558,SPATA6,1.88,0.63,0.89,0.73,1.38,2.29,0.26,0,0,0.45,2.08,2.41,0.64,1.02,0,0.07,0.16,1.4,2.35,1.42,0.81,0.45,0.08,5.42,2.59,1.07,0.91,4.07,0.88,1.15,3.8,2.82,0.875882353,1.948,0.014817302,0.168120553

51710,ZNF44,1.14,0.47,0.71,2.71,2.07,0.21,1.25,1.44,1.14,0.53,1.49,0,0,1.77,1.04,1.02,1.05,1.56,1.24,1.43,1.38,1.32,2.36,2.41,1.43,1.77,1.55,1.16,1.59,0.9,1.54,1.53,1.061176471,1.544666667,0.014826462,0.168120553

4902,NRTN,0.13,0.38,0.11,0.21,0.14,0.13,0,0.3,0.08,0.6,0.88,0.15,0.08,0.61,0.16,0.07,0.08,0,0,0,0.06,0,12.5,2.19,0,0,0,0.12,0.12,0,0.23,0,0.241764706,1.014666667,0.014833492,0.168120553

348327,ZNF530,0.54,0.44,0.65,1.61,1.34,0.91,0.86,1.22,0.71,0.45,0.93,0.6,0.7,1.9,0.91,1.05,1.74,0.45,0.56,0.67,0.33,0.47,0,1.16,0.69,0.63,0.42,0.89,0.62,0.67,1.27,0.35,0.974117647,0.612,0.014835624,0.168120553

51131,PHF11,4.38,8.24,2.17,8.62,8.85,7.86,6.67,6.67,8.17,6.03,4.09,7.66,8.58,7.2,2.78,3.31,7.89,7.83,6.52,10.45,6.98,11.52,11.52,4.46,13.39,8.24,6.93,8.94,6.29,10.75,10.65,12.9,6.421764706,9.158,0.014835624,0.168120553

391712,TRIM61,0.23,0.68,0.52,0.05,0.39,1.3,0.05,0.16,0.32,0.12,0.09,1.13,0.06,0.06,0.22,0.14,1.03,0.54,0.59,0.1,0.18,0.22,0.27,1.09,1.06,1.64,0.42,1.15,1.87,1.1,0.79,0.88,0.385294118,0.793333333,0.014835624,0.168120553

54742,LY6K,0.34,0.3,0.04,0,0.14,4.77,0.23,1.44,0.41,0.4,0.52,0.05,0.21,0,0.5,0.34,17.48,0.14,3.04,0.59,0.76,0.7,0.24,0.26,2.01,2.72,1.15,0.91,0.6,0.88,6.08,3.96,1.598235294,1.602666667,0.014835624,0.168120553

285352,KIF9-AS1,1.06,0.78,1.38,1.06,0.58,1.48,0.77,0.75,1.03,0.97,2.09,0.84,1.57,1.27,1.65,1.4,1.11,0.65,0.87,0.59,0.38,0.86,0.11,0.41,0.85,0.77,1.14,1.66,1.53,0.63,0.62,0.72,1.164117647,0.786,0.014844788,0.168120553

84312,BRMS1L,4.04,4.79,4.79,8.08,6.11,6.65,3.52,5.72,5.82,5.09,5.26,4.85,5.5,9.2,3.71,7.09,5.69,4.17,2.16,4.21,3.88,2.75,1.67,5.88,3.77,6.08,2.07,5.26,3.51,3.85,5.83,5.15,5.641764706,4.016,0.014844788,0.168120553

26146,TRAF3IP1,5.24,2.75,3.9,5.67,5.04,4.05,3.43,5.48,4.21,2.77,3.5,4.3,4.52,5.47,3.47,3.86,4.67,3.47,3.18,3.37,4.19,4.12,1.63,3.28,3.84,3.84,3.77,3.92,3.25,3.35,3.89,3.52,4.254705882,3.508,0.014844788,0.168120553

57786,RBAK,3.12,2.82,2.52,5.6,5.56,3.4,3.93,3.48,3.82,3.38,3.25,2.74,2.96,5.62,3.21,3.08,4.03,2.64,3.66,2.01,2.42,1.97,1.61,6.22,2.33,3.2,2.76,3.45,2.96,2.44,3.59,2.61,3.677647059,2.924666667,0.014853956,0.168120553

100528062,ARMCX5-GPRASP2,0.92,1.02,1.45,0,2.17,1.43,0.68,2.04,1.11,0.57,1.79,1.82,1.12,1.29,0.98,0.28,0.74,0.3,1.14,3.7,1.68,0.68,1.87,2.88,3.12,1.97,2.27,1.93,1.46,2.25,1.38,1.16,1.141764706,1.852666667,0.014853956,0.168120553

10073,SNUPN,12.26,8.89,5.99,11.52,9.77,13.43,11.2,11.86,6.9,10.96,9.27,11.93,10.83,9.2,6.99,10.79,6.47,8.73,16.14,16.85,11.93,11.08,4.17,11.88,18.86,8.78,12.65,11.18,12.41,14.19,13.89,16.93,9.897647059,12.64466667,0.014853956,0.168120553

2954,GSTZ1,8.6,5.62,5.5,2.73,5.06,7.62,6.83,8.55,5.65,6.96,4.67,7.91,6.39,6.46,6.51,9.72,5.2,6.34,13.18,5.04,7.3,6.51,11.21,18.95,9.01,11.31,7.8,9.94,5.81,6.54,10.42,8.41,6.469411765,9.184666667,0.014853956,0.168120553

286077,FAM83H,1.58,0.04,0.3,0,0.22,0.89,0.51,0.94,1.98,1.28,0.17,1.13,0.35,7.11,0.72,0.32,1.91,1.86,3.26,1.32,0.12,0.22,3.19,8.35,1,4.22,1.66,1.69,0.93,2.32,1.85,2.24,1.144117647,2.282,0.014853956,0.168120553

3239,HOXD13,0,0,0,0,0,0,0.23,0,0,0,0,0,0,0,0,0,0,0,0.34,0.03,0.03,0.04,0,0.04,0,0.03,0,0,0,0,0.05,0,0.013529412,0.037333333,0.014897869,0.168519717

3237,HOXD11,0,0,0,0,0,0,4.44,0,0,0,0,0,0,0,0,0,0,0,0.79,5.09,0,0,0,0.06,0,0.11,0,0,0.05,0.28,1.49,0,0.261176471,0.524666667,0.014977494,0.169322137

81607,NECTIN4,0,0,0.02,0,0,0,0.09,0,0.05,0.05,0.06,0,0.08,0.06,0.02,0.02,0,0.22,0,0.02,0.02,0.12,0.16,0.02,0.55,0.21,0.14,0.02,0.48,0.06,0,0.05,0.026470588,0.138,0.015073485,0.170308535

100134015,UBOX5-AS1,0.08,0.17,0.07,0.43,0.31,0.24,0,0.14,0.14,0.11,0.17,0.19,0.36,0,0.25,0.29,0.1,0.04,0.04,0,0,0.1,0.32,0.15,0.11,0.14,0.14,0.07,0.04,0.04,0.07,0,0.179411765,0.084,0.015211959,0.170351609

3818,KLKB1,0,0,0,0,0.03,0,0.07,0,0.08,0,0,0.08,0,0,0.04,0,0,0,0.75,0,0,0.08,5.06,0,0.04,0.11,0.11,0.06,0.03,0.03,0.06,0.04,0.017647059,0.424666667,0.015297663,0.170351609

146556,C16orf89,0.04,0.03,0,0,0,0,0,0,0,0,0.05,0,0.06,0.1,0.14,0.08,0.05,0.36,0.26,0,0,0.11,0.29,0.28,0,0,0.11,9.43,0.04,0.04,1.78,0.45,0.032352941,0.876666667,0.01531015,0.170351609

9379,NRXN2,0.07,0.02,0,0,0,0.06,0.19,0.01,0.12,0.05,0.14,2.86,4.22,0,0.05,0.18,0.23,0.07,0.02,0.31,0,0.02,3.51,0.19,7.65,2.29,3.56,0.26,1.22,0.46,0.27,4.72,0.482352941,1.636666667,0.015400639,0.170351609

161145,TMEM229B,0.14,0,0,0.02,0,0.35,0.38,0.14,0.06,0.05,0.04,0.27,0.04,0.31,0.02,0.11,0.07,0.93,0.15,0.58,0.03,0.21,0,8.98,1.1,0.28,0.32,0.02,0.16,0.67,0.14,0.38,0.117647059,0.93,0.015466913,0.170351609

7096,TLR1,0.31,0.29,0.26,0,0.06,0.13,0.32,0.14,0.03,0.16,0.05,1.19,0.36,0.03,0.26,0.26,0.22,0.24,0.15,0.22,2.44,3.48,1.4,0.22,1.74,0.54,0.39,0.26,0.12,0.45,0.29,0.33,0.239411765,0.818,0.015504839,0.170351609

972,CD74,0.05,5.16,0.25,0,0.3,5.06,0.18,0,0,6.78,0.06,0.61,0.98,14.25,0.13,0.44,0,1.16,10.07,0.18,5.41,1.6,202.46,0.13,395.8,0.38,2.78,5.81,0.15,1.2,12.17,1.5,2.014705882,42.72,0.015542805,0.170351609

79696,ZC2HC1C,0.52,0.09,0.12,0.22,0.11,0.27,0,0.05,0.16,0.43,0.22,0.05,0,0.52,0.04,0.19,0.4,0.65,0.06,0.23,0.81,0.79,7.2,0.39,0.38,0.12,0.27,0.25,0.34,0.22,0.24,0.34,0.199411765,0.819333333,0.015561803,0.170351609

167359,NIM1K,0.32,0.05,0.09,0.06,0.05,0.37,0.42,0.17,0.03,0.08,0.27,0.76,0.51,0.04,0.42,0.1,0.07,0.27,4.66,0.46,0.05,0.12,0.11,1.44,1.64,0.34,0.27,0.41,1.01,0.6,0.19,0.56,0.224117647,0.808666667,0.015571306,0.170351609

403341,ZBTB34,2.59,4.33,2.54,5.26,6.56,2.64,1.69,2.97,2.51,2.11,3.71,2.49,2.25,2.67,2.97,3.91,3.05,1.32,1.52,2.52,2.97,1.28,0.57,4.19,2.04,1.46,1.96,3.28,2.59,1.89,3.3,1.69,3.191176471,2.172,0.015599829,0.170351609

101101775,TMEM220-AS1,0.47,0.32,0.11,1.14,0,0.64,0.42,0.51,0.61,0.35,0.65,0.15,0,0.32,0.16,0.37,0.49,0.98,0.7,0.43,0.38,0,1.18,1.52,0.36,0.59,0.3,0.66,1.05,0.72,1.01,0.73,0.394705882,0.707333333,0.015609341,0.170351609

91614,DEPDC7,2.4,1.19,1.29,0,0,1.86,2.48,1.45,1.45,0.7,1.19,0.19,0.67,1.39,1.39,0.46,1.24,2,1.61,2.76,0.04,0.15,12.93,3.74,4.54,8.75,3.06,1.22,1.46,0.95,1.65,2.04,1.138235294,3.126666667,0.015618856,0.170351609

81930,KIF18A,1.39,0.91,0.36,0.91,0.52,1.73,0.89,1.02,1.75,2.72,0.69,0.49,1.02,6.26,1.4,0.9,3.99,0.78,0.46,1.12,2.92,0.8,0.08,0.3,0.9,0.44,0.5,0.84,0.93,0.36,1.01,0.23,1.585294118,0.778,0.015618856,0.170351609

100129482,ZNF37BP,3,2.19,2.18,3.81,3.5,3.53,1.91,4.19,2.81,1.52,2.43,3.48,3.23,3.28,2.07,3.12,2.55,0.75,2.46,2.38,1.04,0.94,0.49,3.05,4.17,1.11,1.53,2.07,1.86,2.14,4.06,2.18,2.870588235,2.015333333,0.015637894,0.170351609

256471,MFSD8,2.33,1.85,2.48,5.34,4.79,2.71,2.92,3.61,3.86,2.22,2.45,2.8,3.05,5.77,2.54,3.3,3.49,1.47,3.05,2.84,2.66,2.52,1.89,3.17,2.24,1.99,2.39,2.14,2.27,2.45,3.11,1.97,3.265294118,2.410666667,0.015637894,0.170351609

57654,UVSSA,3,2.48,2.85,4.79,4.55,3.02,2.13,4.36,3.64,3.09,4.28,1.79,2.39,3.85,4.07,5.04,3.64,1.94,3.2,1.62,1.46,1.04,1.16,7.1,3.67,1.62,1.81,9.18,1.66,2.19,3.04,1.69,3.468823529,2.825333333,0.015637894,0.170351609

4983,OPHN1,3.98,4.14,3.36,9.51,7.43,4.3,5.99,4.94,4.95,3.86,5.76,3.64,4.84,7.81,3.41,4.08,4.95,3.46,3.4,2.58,4.62,4.07,2.25,5.04,4.31,3.71,3.02,4.59,3.47,3.46,5.32,3.22,5.114705882,3.768,0.015637894,0.170351609

10282,BET1,7.25,8.54,7.86,9.21,9.3,9.6,6.91,6.91,5.48,8.01,6.92,8.36,11.99,6.69,6.81,7.86,7.81,6.53,4.48,6.59,6.93,6.18,8.31,7.12,7.48,4.52,7.2,6.63,5.65,4.99,8.41,7.89,7.971176471,6.594,0.015637894,0.170351609

4277,MICB,5.76,0.99,1.37,5.81,3.82,5.6,2.46,4.06,3.73,0.99,8.81,2.6,3.05,4.96,4.39,6.01,9.69,0.25,0.86,3.34,8.65,8.34,1.01,1.77,2.99,1.3,1.43,0.91,1.14,1.96,2.49,1.43,4.358823529,2.524666667,0.015637894,0.170351609

55304,SPTLC3,1.21,5.35,3.76,1.41,2.85,4.2,1.16,11.2,1.55,7.28,1.12,8.22,3.39,9.96,2.13,6.42,3.53,3.58,3.59,0.29,0.29,0.61,1.3,0.04,5.74,2.69,2.54,1.65,1.17,0.95,4.52,0.25,4.396470588,1.947333333,0.015647417,0.170351609

149076,ZNF362,25.57,11.87,17.75,19.15,22.69,17.22,5.27,21.24,18.74,8.74,24.05,10.28,5.66,15.33,10.28,12.93,15.85,13.49,5.39,10.34,10.58,4.41,1.11,17.56,13.01,6.78,9.63,12.78,11.47,10.12,15.29,7.95,15.44823529,9.994,0.015647417,0.170351609

9020,MAP3K14,6.08,7.46,5.18,3.6,6.06,9.5,4.18,6.15,3.28,7.22,2.25,3.63,3.66,8.06,8.7,6.72,6.09,4.56,2.64,1.83,3.22,1.72,1.86,4.67,6.45,6.75,3.22,3.96,2.94,3.01,9.66,2.91,5.754117647,3.96,0.015647417,0.170351609

8899,PRPF4B,11.74,8.14,9.79,15.78,15,9.39,8.69,13.51,11.2,9.07,8.82,8.33,6.33,13.99,9.06,9.88,11.88,5.64,7.17,6.88,12.01,6.59,4.99,14.46,10.21,5.93,5.93,10.58,6.37,6.8,10.62,5.95,10.62352941,8.008666667,0.015647417,0.170351609

161829,EXD1,3.38,3.65,2.72,4.39,3.96,2.32,2.43,3.23,4.57,2.93,3.2,3.03,3.31,4.59,2.65,2.07,3.96,2.74,2.84,1.76,2.39,2.83,1.93,6.41,2.64,2.12,2.23,2.8,2,3.25,2.54,3.07,3.317058824,2.77,0.015647417,0.170351609

166614,DCLK2,6.32,3.58,8.64,3.24,3.85,1.48,1.54,2.91,3.24,0.85,4.95,4.16,0.8,20.12,1.88,5.12,4.24,17.09,2.18,1.59,0.81,2.11,0.1,0.88,0.64,3.29,2.57,1.39,2.21,1.03,1.62,1.77,4.524705882,2.618666667,0.015647417,0.170351609

6461,SHB,10.81,7.78,6.77,13.01,13.43,4.67,11.37,15.77,15.82,9.46,3.58,14.72,9.5,9.5,2.02,9.47,17.7,11.55,5.7,3.41,6.04,12.26,5.24,4.46,6.43,1.48,3.76,4.96,6.55,3.35,7.58,14.53,10.31647059,6.486666667,0.015647417,0.170351609

117584,RFFL,6.2,5.6,4.3,6.01,6.16,5.2,3.33,6.9,4.76,9.28,5.55,5.2,5.77,7.3,4.79,5.04,3.62,4.62,3.65,2.85,3.95,3.41,10.04,4.32,5.26,2.62,3.7,5.95,3.68,2.9,5.88,3.51,5.588823529,4.422666667,0.015647417,0.170351609

114791,TUBGCP5,4.99,2.88,4.91,5.68,4.44,6.81,5.02,5.91,4.87,4.62,5.23,3.8,4.98,7.49,4.21,4.56,8,4.31,3.76,4.15,4.19,4.1,1.77,5.33,4.53,6.1,3.51,4.53,3.47,3.63,6.31,4.18,5.2,4.258,0.015647417,0.170351609

9202,ZMYM4,11.32,10.12,10.81,14.03,12.87,11.34,9.41,13.1,10.94,9.53,10.43,11.2,10.94,11.88,9.28,9.52,12.01,9.87,5.28,8.29,15.56,8.61,2.48,10.52,10.18,11.25,8.18,9.48,10.41,9.14,12.31,9.15,11.10176471,9.380666667,0.015647417,0.170351609

79572,ATP13A3,10.96,6.36,9,7.72,6.17,6.08,31.14,11.86,11.86,8.52,10.37,18.3,17.52,11.39,5.98,7.31,12.55,12.13,17.05,13.26,13.08,21.82,8.48,11.52,16.14,16.93,23.01,15.33,8.55,14.69,10.39,19.5,11.35823529,14.792,0.015647417,0.170351609

8468,FKBP6,0.24,0.15,0.07,0.17,0.1,0,0.11,0.16,0,0.06,0,0.12,0.14,0.06,0.11,0,0.06,0.05,0.06,0,0,0,0.09,0,0.06,0.05,0,0.17,0.04,0,0.04,0,0.091176471,0.037333333,0.015744479,0.170351609

641455,POTEM,0,0.11,0.01,1.49,1.33,0.02,0.01,0,0,0,0.19,0.23,0.28,1.18,0.13,0.63,0.36,0,0.01,0,0.04,0.1,0,0,0.12,0.01,0.02,0.01,0,0,0,0.04,0.351176471,0.023333333,0.01576451,0.170351609

7857,SCG2,8.64,131.47,24.19,0.03,0.69,7.74,3.13,2,10.41,48.46,12.7,147.87,37.65,525.29,12.26,2.7,3.62,33.11,0.65,0.55,0,0.54,1.14,0.6,13.38,1.85,0.51,6.92,4.15,0.91,7.16,35.23,57.57941176,7.113333333,0.015828977,0.170351609

55366,LGR4,26.26,12.99,24.23,25.42,10.82,32.36,11.89,30.36,30.97,9.14,3.75,28.79,17.9,8.51,34.28,13.66,22.03,17.99,14.82,27.58,4.68,4.59,8.24,5.45,16.67,2.22,10.4,1.05,24.99,22.07,5.21,9.38,20.19764706,11.68933333,0.015828977,0.170351609

201232,SLC16A13,2.03,1.77,1.86,60.3,29.86,0.85,1.02,1.27,1.31,2.05,1.75,2.43,1.71,1.78,0.8,2.62,1.45,0.62,0.69,0.91,4.66,3.05,10.16,0.64,1.07,0.42,0.52,0.48,0.98,0.88,1.47,1.29,6.756470588,1.856,0.015828977,0.170351609

100131897,FAM196B,6.52,5.62,8.05,6.63,12.31,1.13,1.81,3.8,1.54,3.02,1.82,3.42,4.14,0.44,1.05,10.1,11.17,1.62,0.02,1.56,3.72,4.77,0.16,0.05,3.36,0.14,0.6,1.74,1.8,2.66,4.64,2.59,4.857058824,1.962,0.015828977,0.170351609

84874,ZNF514,4.17,5.1,5.24,10.49,9.63,3.53,5.26,7.28,5.68,6.52,4.74,4.88,3.95,7.05,5.31,6.03,5.59,3.24,8.52,2.37,2.29,1.82,1.18,10.58,4.92,2.52,3.39,5.87,3.29,2.74,8.34,3.79,5.908823529,4.324,0.015828977,0.170351609

81557,MAGED4B,39.19,48.19,40.81,6.4,9.38,24.57,15.15,39.72,28.81,38.34,36.2,31.25,20.86,30.2,48.26,29.67,33.16,30.68,22.31,2.95,7.71,0.18,0.24,17.62,24.86,9.29,10.24,76.83,16.8,6.96,54.82,13.22,30.59764706,19.64733333,0.015828977,0.170351609

728239,MAGED4,39.19,48.19,40.81,6.4,9.38,24.57,15.15,39.72,28.81,38.34,36.2,31.25,20.86,30.2,48.26,29.67,33.16,30.68,22.31,2.95,7.71,0.18,0.24,17.62,24.86,9.29,10.24,76.83,16.8,6.96,54.82,13.22,30.59764706,19.64733333,0.015828977,0.170351609

57719,ANO8,4.06,5.22,2.97,10.52,10.74,2.94,2.37,5.99,4.41,7.3,7.29,4.72,4.5,10.58,5.15,4.35,5.91,2.99,3.19,3.23,3.05,2.08,2.77,6.61,7.22,2.78,2.62,3.6,3.33,2.9,8.02,4.22,5.824705882,3.907333333,0.015828977,0.170351609

138311,FAM69B,4.89,3.18,5.66,180.73,171.59,3.02,1.57,4.06,6.15,6.46,24.91,9.78,3.5,12.42,8.12,9.84,3.67,6.5,0.86,0.51,115.6,30.13,1.14,32.76,0.59,0.17,0.18,2.09,0.4,0.34,3.69,3.33,27.03235294,13.21933333,0.015828977,0.170351609

7267,TTC3,60.37,92.73,76.13,34.31,31.01,48.15,56.16,58.3,43.57,95.58,72.78,76.53,81.43,40.41,60.2,56.09,44.82,58.28,31.95,47.93,26.97,31.1,9.97,82.05,57.16,28.46,41.11,49.05,42.49,38.15,59.16,42.25,60.50411765,43.072,0.015828977,0.170351609

286128,ZFP41,6.13,4.11,3.18,6.37,7.09,4.9,2.98,7.08,5.25,3.49,5.37,4.94,3.79,5.58,4.48,4.74,5.82,3.66,3.12,4.08,3.86,2.16,0.9,4.64,5.68,3.44,3.67,4.36,4.27,4.71,4.96,3.92,5.017647059,3.828666667,0.015828977,0.170351609

4968,OGG1,12.7,14.19,8.89,12.92,12.11,13.3,5.97,12.95,9.84,10.75,10.62,9.43,11.81,15.23,10.72,12.76,13.61,9.09,7.51,11.36,7.24,6.51,3.08,9.21,7.8,8.17,7.2,13.93,11.6,10.39,13.34,11.18,11.63529412,9.174,0.015828977,0.170351609

375743,PTAR1,6.55,4.7,4.26,8.21,6.26,6.45,6.19,7.72,5.08,6.91,7.18,4.6,5.79,6.34,4.74,8.14,6.94,4.99,6.22,3.46,3.92,4.54,1.47,8.48,5.27,3.37,4.28,7.48,3.95,3.4,7.86,3.9,6.238823529,4.839333333,0.015828977,0.170351609

22890,ZBTB1,9.39,10.86,8.88,19.66,15.18,12.15,9.08,11.2,12.25,9.77,14.82,10.28,10.16,12.02,9.48,15.3,12.55,7.89,13.37,8.93,9.2,7.59,3.77,14.64,14.35,6.89,7.08,9.84,9.31,9.05,13.73,8.94,11.94294118,9.638666667,0.015828977,0.170351609

56006,SMG9,23.41,13.56,9.95,18.15,19.33,13.98,12.75,23.43,15.1,12.72,16.35,11.43,14.48,14.53,13.14,18.02,25.06,12.01,8.14,14.67,13.28,12.5,4.74,11.44,13.96,9.37,16.41,11.79,14.33,14.24,16.85,8.62,16.19941176,12.15666667,0.015828977,0.170351609

64089,SNX16,4.14,5.08,1.75,3.15,2.79,3.96,3.5,3.75,2.31,3.91,3.62,2.16,2.94,1.86,2.02,4.76,3.34,1.76,0.87,3,2.47,2.27,1.25,4.88,2.54,1.85,1.21,2.08,2.05,2.83,2.46,3.29,3.237647059,2.320666667,0.015828977,0.170351609

4957,ODF2,13.54,16.07,19.76,22.06,23.41,15.13,9.66,13.47,13.08,27.71,11.45,8.13,8.6,25.83,25.34,26.24,20.9,11,9.54,9.76,16.81,10.1,10.24,13.2,12.31,9.7,6.5,18.06,10.61,6.83,25.38,9.92,17.66941176,11.99733333,0.015828977,0.170351609

9315,NREP,276.49,207.93,240.6,27.96,35.59,72.31,107.26,359.48,446.71,73.03,200,97.79,70.14,163.18,94.45,144.69,451.92,139.77,32.63,145.55,27.94,20.31,2.67,1.5,49.87,39.03,162.78,53.39,133.01,157.61,146.53,78.86,180.5605882,79.43,0.015828977,0.170351609

10501,SEMA6B,3.61,16.71,2.23,191.37,246.82,11.01,1.01,3.42,5.47,6,41.58,18.18,8.57,5.86,3.17,1.58,1.57,0.25,0.18,0.24,129.33,82.62,2.88,0.11,0.64,12.01,0,1.61,9.94,0.07,0.93,2.9,33.42117647,16.24733333,0.015828977,0.170351609

64943,NT5DC2,96.98,247.18,92.55,34.92,41.2,68.32,64.92,92.15,101.81,288.06,131.82,68.76,91.63,107.69,181.72,66.85,89.92,95.93,24.51,39.18,99.63,66.14,1.78,32.73,61.46,40,55.7,100.17,95.69,30.83,110.2,28.79,109.7929412,58.84933333,0.015828977,0.170351609

81792,ADAMTS12,23.5,10.2,9.2,4.59,7.43,10.27,14.39,9.26,20.11,28.23,5.35,14.46,13.72,16.88,2.42,4.72,6.93,12.65,0.11,4.2,0.06,0.98,0.28,0.03,1.63,2.22,10.38,5.02,15.6,14.2,15.48,4.84,11.86235294,5.845333333,0.015828977,0.170351609

7257,TSNAX,11.72,10.48,11.33,15.41,14.83,17,13.25,14.05,10.02,9.77,14.89,12.06,13.09,15.8,8.48,10.26,12.62,11.01,10.35,10.08,10.66,8.25,5.84,34.27,11.16,12.89,10.9,9.07,9.2,8.72,11.94,9.38,12.65058824,11.58133333,0.015828977,0.170351609

440288,UBL7-AS1,2.72,2.71,2.26,2.35,4.19,1.3,1.01,2.46,1.64,2.51,3.15,1.92,2.04,3.33,2.98,2.3,2.52,1.42,2.32,1.06,2.61,1,1.74,2.69,1.76,1.19,1.6,2.39,2.17,1.25,2.23,2.02,2.434705882,1.83,0.015828977,0.170351609

4089,SMAD4,9.12,7.49,7.29,10.54,9.76,9.87,6.42,10.91,9.23,7.4,7.27,7.09,6.24,8.95,8.41,8.28,8.83,6.15,5.42,8.17,7.15,4.37,3.57,12.09,6.53,5.9,6.22,7.5,7.47,7.46,8.84,7.45,8.417647059,6.952666667,0.015828977,0.170351609

23215,PRRC2C,18.42,14.32,17.36,33.62,27.42,26.43,14.84,20.32,17.12,17.16,23.32,19.87,20.52,23.22,17.21,19.49,22.91,12.85,15.72,12.6,26.05,20.06,6.75,23.38,17.91,14.24,14.59,19.51,13.95,15,19.62,14.13,20.79705882,16.424,0.015828977,0.170351609

9685,CLINT1,28.18,14.33,26.06,25.58,27.67,20.11,28.7,22.84,18.81,22.2,30.29,17.48,16.43,23.31,25.44,18.14,23.98,17.04,23.95,17.6,27.03,20.74,20.87,16.39,15.4,20.82,17.35,12.97,19.2,19.75,18.98,12.71,22.91470588,18.72,0.015828977,0.170351609

10179,RBM7,18.8,12.8,14.41,12.76,15.05,12.67,15.34,15.29,16.21,13.79,12.9,13.82,12.34,21.34,12.51,12.26,18.44,9.97,14.08,12.27,14.79,10,8.7,11.09,13.25,9.25,11.27,9.8,13.68,15.35,13.08,14.73,14.74882353,12.08733333,0.015828977,0.170351609

7525,YES1,21.38,11.94,13.55,43.64,37.05,13.36,12.67,23.56,13.18,12.19,20.79,18.6,14.87,12.43,12.4,15.98,12.58,11.01,7.79,8.88,26.67,14.63,11.23,11.18,13.1,13.47,9,9.77,13.99,13.33,15.69,11.22,18.24529412,12.73066667,0.015828977,0.170351609

571,BACH1,11.44,6.95,7.1,10.93,18.65,12.86,30.14,13.72,37.16,11.35,10.07,13.35,14.22,18.37,14.61,15.69,30.04,9.49,2.7,12.14,4.77,4.3,4.44,5.97,7.37,6.41,64.61,11.32,12.25,11.71,20.87,12.88,16.27352941,12.74866667,0.015828977,0.170351609

9790,BMS1,8.97,6.03,7.41,13.26,11.03,7.72,5.67,9.1,7.45,7.29,6.62,4.75,4.87,9.51,7.11,6.94,10.47,6.08,5.21,5.07,10.14,5.64,3.64,7.3,5.76,7.73,5.36,5.84,6.14,5.37,6.74,5.04,7.894117647,6.070666667,0.015828977,0.170351609

112755,STX1B,4.15,2.38,4.35,0.07,0.05,1.85,0.98,2.98,2.64,5.1,2.89,3,7.5,1.42,1.82,3.5,3.66,2.5,1.64,0.92,0.09,0.23,0.34,0.85,4.17,0.28,0.79,3.25,1.52,0.76,2.18,0.15,2.843529412,1.311333333,0.015828977,0.170351609

7697,ZNF138,8.48,6.48,7.86,10.03,9.21,6.87,3.71,9.68,7.76,5.01,10.07,5.73,5.58,9.08,6.21,7.2,10.6,5.21,2.82,5.66,8.97,5.48,3.85,8.13,5.08,7.26,4.94,7.38,6.13,5.57,7.29,5.31,7.621176471,5.938666667,0.015828977,0.170351609

4999,ORC2,6.52,3.65,4.22,8.21,8.4,5.45,4.65,9.24,5.72,5.19,5.26,4.67,3.69,5.37,4.52,5.23,8.44,3.92,4.8,4.24,7.68,3.91,1.24,6.24,5.58,2.87,2.98,3.98,4.04,3.07,5.62,3.5,5.79,4.244666667,0.015828977,0.170351609

27316,RBMX,57.99,67.97,55.18,80.23,67.56,70.74,29.6,61.4,45.34,65.17,49.02,51.54,53.33,69.23,50.52,68.16,58.02,30.24,47.01,36.49,75.54,30.77,19.05,47.34,58.72,39.16,28.45,108.25,38.49,42.84,66.8,42.76,58.88235294,47.46066667,0.015828977,0.170351609

1969,EPHA2,48.28,18.29,16.9,43.61,52.67,33.58,29.59,55.45,46.16,46.81,15.99,5.93,4.81,31.91,20.41,13.48,56.08,54.27,5.33,8.45,51.17,65.95,2.42,0.13,16.21,3.97,13.7,4.54,5.83,5.1,16.7,11.18,31.76176471,17.66333333,0.015828977,0.170351609

9128,PRPF4,11.62,7.41,8.22,9.08,9.45,12.04,7.1,10.33,9.13,11.98,10.08,8.49,8.46,12.36,11.45,9.87,15.95,9.3,7.06,7.33,11.84,10.8,6.66,8.66,7.97,9.84,7.73,8.17,8.28,7.58,9.33,7.22,10.17764706,8.518,0.015828977,0.170351609

2932,GSK3B,13.94,6.92,10.8,11.09,11.6,10.42,13.69,12.99,17.58,8.62,14.21,9.02,12.05,15.62,10.21,11.94,11,9.62,7.57,9.29,10.49,12.64,2.1,14.11,8.16,12.06,10.12,8.95,9.27,9.42,9.45,8.13,11.86470588,9.425333333,0.015828977,0.170351609

5480,PPIC,26.58,9.94,35.87,20.96,26.12,36.69,28.95,13.74,16.26,11.9,74.37,30.96,30.55,6.67,19.12,8.01,31.69,57.58,97.6,66.58,29.39,30.72,15.84,14.8,47.43,38,66.32,12.51,62.9,57.32,26.3,50.01,25.19882353,44.88666667,0.015828977,0.170351609

1519,CTSO,9.91,6.33,4.25,3.16,6.35,13.34,4.79,10.09,6.74,13.31,19.17,16.49,7.16,9.01,6.61,4.56,6.87,11.64,17.07,13.29,8.96,10.72,4.04,13.24,19.07,10.4,10.88,24.2,6.98,14.26,13.11,17.43,8.714117647,13.01933333,0.015828977,0.170351609

55785,FGD6,1.96,4.87,1.92,4.1,3.45,1.44,1.43,2.44,2.17,3.58,3.25,1.79,1.45,3.18,2.77,2.15,1.67,1.29,0.34,1,3.06,3.62,0.28,0.94,2.04,0.37,1.19,3.01,1.47,1.04,9.25,0.46,2.565882353,1.957333333,0.015828977,0.170351609

8496,PPFIBP1,11.58,7.01,10.26,23.78,41.78,11.03,16.27,12.21,11.83,15.14,62.77,8.13,26.61,12.77,14.88,13.05,14.54,17.26,53.26,20.89,20.94,14.95,1.98,30.79,31.75,16.74,32,20.25,18.73,18.39,33.5,19.95,18.44941176,23.42533333,0.015828977,0.170351609

219402,MTIF3,14.85,21.69,11.37,22.22,18.96,23.84,17.24,21.49,15.71,23.63,10.48,14.18,19.01,23.02,12.46,17.06,13.33,20,17.4,22.04,24.6,27.32,22.2,35.71,29.22,17.91,17.76,24.02,16.51,16.54,25.98,21.18,17.67882353,22.55933333,0.015828977,0.170351609

28969,BZW2,23.86,23.99,27.61,43.12,40.36,24.9,33.65,37.12,38.38,33.77,22.13,37.17,35.02,24.87,20.54,20.08,46.28,22.78,25.82,17.36,56.93,25.01,10.43,23.27,18.1,29.58,13.12,36.26,18.88,14.03,28.92,15.12,31.34411765,23.70733333,0.015828977,0.170351609

1327,COX4I1,97.65,159.68,145.79,191.34,149.99,246.03,127.39,162.79,120.02,189.38,148.91,204.58,201.51,172.09,148.39,145.66,122.13,163.87,184.22,172.07,257.84,196.81,152.85,303.93,170.43,282.51,146.65,192.08,156.98,170.93,206.85,216.62,160.7841176,198.3093333,0.015828977,0.170351609

389203,SMIM20,14.35,18.31,15.76,8.14,7.29,19.82,9.73,14.7,13.52,15.98,20.27,21.33,21.17,16.68,10.68,12.88,9.81,19.91,15.03,17.02,14.49,13.63,19.17,24.25,20.15,19.35,15.28,22.96,16.64,20.52,21.67,20.67,14.73058824,18.716,0.015828977,0.170351609

10606,PAICS,34.18,25.9,35.43,29.48,21.88,41.66,30.91,32.94,30.69,59.28,21.29,35.1,53.26,41.09,37.53,22.35,47.83,25.64,23.67,17.6,49.93,17.97,22.1,30.25,29.24,37.32,14.39,30.31,26.32,19.28,33.71,25.99,35.34117647,26.91466667,0.015828977,0.170351609

401152,C4orf3,23.79,26.4,26.32,41.58,42.81,33.85,43.42,43.51,33.59,22.53,26.27,73.59,65.09,49.14,23.3,19.07,16.97,58.31,55.15,55.6,34.5,24.95,16.96,69.32,73.17,93.44,54.94,62.71,42.51,35.87,57.12,68.8,35.95470588,53.55666667,0.015828977,0.170351609

25777,SUN2,26.04,13.09,14.77,30.6,39.11,30.86,29.85,29.83,44.09,26.32,28.87,15.5,9.32,34.65,30.75,16.22,37.45,55.9,26.09,37.96,48.66,55.55,30.79,32.79,36.28,31.01,42.82,27.76,41.2,31.83,30.47,25.17,26.90117647,36.952,0.015828977,0.170351609

10412,NSA2,35.39,43.3,32.58,44.94,33.15,59.49,29.64,47.48,34.51,49.28,28.78,49.12,45.95,42.43,32.61,40.74,31.1,29.91,47.31,59.37,69.28,36.92,24.56,64.54,46.37,60.28,34.65,78.53,49.23,54.6,70.45,53.51,40.02882353,51.96733333,0.015828977,0.170351609

408050,NOMO3,3.72,1.98,7.23,10.77,9.88,3.12,24.41,16.24,17.84,3.82,7.84,1.51,1.33,11.59,5.41,2.19,5.63,38.26,47.65,9.34,41.11,11.97,8.54,8.47,7.54,11.1,27.38,8.78,15.55,4.71,13.3,4.27,7.912352941,17.198,0.015828977,0.170351609

1490,CTGF,472.37,17.19,175.1,157.72,248.88,356.67,915.68,210.28,238.85,266.33,247.87,47.4,39.03,128.51,11.74,2.88,449.52,1191.07,1941.15,644.38,1308.78,1946.54,15.49,1.95,522.3,413.95,1563.61,21.9,319.05,544.98,291,299.17,234.4717647,735.0213333,0.015828977,0.170351609

51296,SLC15A3,0.11,0.75,1.53,0.08,0.24,9.92,0.23,6.89,1.77,0.71,1.08,3.7,2.29,1.79,0.09,0.72,1.46,0.66,2.45,5.97,1.31,2.39,5.67,0.22,7.72,1.91,1.78,2.73,2.76,7.94,3.42,11.17,1.962352941,3.873333333,0.015828977,0.170351609

1,A1BG,7.03,15.01,8.84,6,4.38,8.7,4.05,9.32,5.24,10.73,7.54,10.83,10.78,8.64,13.3,5.75,5.52,5.32,1.35,5.46,0.68,1.55,419.12,8.02,3.89,4.28,1.52,10.37,2.38,3.02,6.19,10.82,8.332941176,32.26466667,0.015828977,0.170351609

4493,MT1E,13.38,12.83,21.51,9.97,5.46,65.24,29.69,71.23,59.59,19.14,21.22,35.13,13.11,26.3,34.87,16.91,75.94,13.27,7.46,36.79,26.15,100.34,1050.13,26.82,75.82,132.68,39.73,86.61,38.28,39.98,62.92,100.03,31.26588235,122.4673333,0.015828977,0.170351609

196410,METTL7B,0.1,0,0.13,0,0,0.05,0.36,0,0,0.13,0.4,0.06,0.06,0.45,0.12,0.58,0.91,11.76,0.18,1.63,0.05,0,53.43,0,2.03,14.3,1.32,0.22,1.14,0.49,0.13,0.23,0.197058824,5.794,0.015879377,0.1707997

145773,FAM81A,0,0.02,0.05,0,0,0.1,0,0,0.02,0,0.39,0,0,0,0,0.02,0.03,0.07,0.02,0.05,0,0,0.04,3.07,0.06,0.16,0.05,0.15,0.02,0.06,0.05,0,0.037058824,0.253333333,0.015894208,0.170864925

401427,OR2A7,0.19,0,0,0.17,0,0,0.09,0.09,0.28,0,0.33,0,0.1,0,0,0,0,0,0.42,0.25,0,0,0.41,0.89,0.46,0.07,0.87,0,0.39,0.1,0.1,0.81,0.073529412,0.318,0.01607361,0.172698264

94122,SYTL5,0.58,1.99,15.07,0,0,3.19,0,0.6,3.26,2.12,2.54,3.13,0.56,0.73,0.51,0.18,1.62,1.96,0.08,0,0.06,0.04,0.5,0,0.12,0,0.1,0.3,0.47,0.06,9.65,0.02,2.122352941,0.890666667,0.016133352,0.173244644

729540,RGPD6,1.81,0.39,0.4,1.63,0.85,1.12,0.4,0,0,0.58,0,0.55,0.72,0,0.44,1.35,0.57,0.42,0.95,0.17,1.1,0,0.3,0,0,0,0,0.16,0,0.18,0,0,0.635882353,0.218666667,0.016154317,0.17337425

388815,MIR99AHG,0.36,0.7,0,0.02,0.13,0.09,0.34,1.52,0.28,0,0,1.01,0.38,0,0.16,0.04,0.33,0.94,1.35,0.57,1.07,0.18,0.38,0,0.3,0.56,0.61,0.18,0.7,1.01,0.8,0.61,0.315294118,0.617333333,0.016241317,0.174212032

643904,RNF222,0.07,0.16,0.12,0.19,0.41,0.14,0.1,0.35,0.2,0.09,0.05,0.11,0.18,0.27,0.17,0.35,0.22,0.12,0.13,0.15,0.05,0.08,0.27,0.33,0.07,0.03,0.07,0.06,0.06,0.04,0.1,0.14,0.187058824,0.113333333,0.016379166,0.174696001

100131244,ANKRD63,0,0,0.11,0.28,0.14,0.06,0,0.07,0.46,0,0,0.61,0.33,0,0.08,0.13,0,0,0,0.07,0.13,0,0,0,0,0.07,0,0,0,0,0,0,0.133529412,0.018,0.016382962,0.174696001

148229,ATP8B3,0.55,0.9,0.33,0.3,0.14,0.66,0.35,0.96,0.74,1.14,0.63,0.33,0.35,1.53,0.92,0.6,0.37,0.18,0.48,0.21,0.09,0.25,0.08,1.11,0.56,0.14,0.17,0.66,0.13,0.35,0.9,0.23,0.635294118,0.369333333,0.016408769,0.174696001

10815,CPLX1,0.27,0,0,0,0,0,0,0.08,0,0.22,0.07,0.12,0.04,0.09,0,0.03,0.25,0.29,1.53,0.04,0.89,0.68,5.12,0.04,0.18,2.11,0,0.77,0.03,0,0.09,0.04,0.068823529,0.787333333,0.016421401,0.174696001

3111,HLA-DOA,0.02,0,0,0.11,0,0.02,0,0.03,0.24,0,0,0.02,0.03,0,0.02,0,0.03,0.07,0.06,0,0.04,0,0.4,0.12,16.47,0.05,0,0.17,0.02,0.02,0.34,0.05,0.030588235,1.187333333,0.016433067,0.174696001

57150,SMIM8,1.92,1.51,1.09,2.36,2.29,2.17,1.16,2.28,1.23,1.68,1.7,1.62,1.76,1.57,1.84,0.66,1.39,1.39,1.42,1.35,1.27,0.91,1.04,2.79,1.22,0.91,1.27,1.64,0.76,1.13,1.35,1.3,1.660588235,1.316666667,0.016448275,0.174696001

51015,ISOC1,3.02,1.39,2.83,4.88,2.11,2.43,2.3,3.88,3.56,1.82,6.87,2.43,3.15,3.64,2.26,2.15,4.58,10.53,8.88,2.18,9.08,4.27,11.78,5.25,2.5,5.57,2.45,3.03,3.42,2.5,3.88,4.75,3.135294118,5.338,0.016458157,0.174696001

338817,LINC01252,0.72,1.05,0.85,1.91,1.32,0.59,0.17,0.18,0.27,1.5,1.16,0.55,0.89,0.87,1.13,0.99,0.29,0.13,0.96,0.49,0.08,0.16,0.23,0.53,2.91,0.15,0.54,0.72,0.25,0.16,1.03,0.28,0.849411765,0.574666667,0.016468042,0.174696001

503542,SPRN,2.85,1.57,2.02,1.85,3.17,1.53,5,2.29,2.43,1.95,5.7,1.25,1.39,3.99,2.5,1.95,2.44,3.17,0.65,1.96,1.54,1.75,1.93,2.21,1.41,1.88,1.06,1.64,1.58,0.84,2.08,0.9,2.581176471,1.64,0.016468042,0.174696001

55775,TDP1,3.95,4.2,2.53,7.05,6.93,5.38,2.77,4.79,3.24,6.75,3.51,3.77,4.75,6.24,3.79,4.83,5.38,2.87,2.09,2.65,6.42,4.01,0.98,4.66,2.82,3.8,1.64,6.28,2.7,1.77,4.52,2.53,4.697647059,3.316,0.016468042,0.174696001

414918,DENND6B,5.8,3.76,4.73,6.61,11.26,4.68,3.05,5.51,4.49,5.4,9.35,6.64,4.13,8.02,4.66,6.89,4.74,2.59,2.83,5.4,2.8,4.95,0.56,5.23,5.42,1.33,4.23,6.02,2.32,3.08,7.79,3.11,5.865882353,3.844,0.01647793,0.174696001

284309,ZNF776,3.33,2.82,2.35,5.67,5.68,3.35,2.79,3.81,3.85,2.58,3.66,2.7,2.53,5.17,2.88,3.18,3.77,2.42,1.91,1.48,2.86,1.19,0.73,3.87,3.81,2.39,1.7,3.88,1.57,1.86,4.11,2.46,3.536470588,2.416,0.01647793,0.174696001

6654,SOS1,7.17,5.56,6.14,9.2,7.7,7.67,8.1,8.48,8.53,6.51,8.97,9.15,7.09,6.84,6.05,7.38,6.28,6.01,5.47,5.91,6.15,5.21,2.07,9.11,6.79,5.33,5.48,7.54,6.07,7.85,7.7,6.89,7.46,6.238666667,0.01647793,0.174696001

220930,ZEB1-AS1,1.8,1.69,1.41,2.91,2.28,1.66,1.36,1.59,0.8,2.13,1.99,1.76,1.67,2.01,1.92,2.45,1.27,1.58,1.57,0.89,1.32,0.75,0.44,0,0.8,2.23,1.34,2.86,1.33,1.14,2,1.61,1.805882353,1.324,0.01647793,0.174696001

9895,TECPR2,6.8,7.06,6.21,8.92,7.91,9.62,4.27,8.08,7.04,6.25,11.69,7.59,6.11,8.22,5.4,9.14,7.52,5.62,4.56,5.96,7.84,6.21,1,15.86,5.6,4.35,5.3,9.13,4.81,4.59,7.51,3.89,7.519411765,6.148666667,0.01647793,0.174696001

100507290,ZNF865,7.19,6.54,5.46,7.26,7.25,7.3,3.62,8.3,6.98,5.45,8.29,6.88,4.68,6.74,6.2,5.94,9.8,4.81,4.92,7.95,4.68,2.78,2.11,5.58,6.56,4.04,5.24,6.96,5.56,6.99,5.13,6.17,6.698823529,5.298666667,0.01647793,0.174696001

64924,SLC30A5,17.42,16.76,15.37,13.79,13.4,18.8,18.02,23.42,14.96,20.46,18.19,14.92,13.88,17.54,16.56,12.83,20.55,13.7,14.98,14.23,13.51,13.94,9.37,18.59,15.25,13.2,13.88,16.68,12.54,15.32,14.65,14.54,16.87470588,14.292,0.01647793,0.174696001

202052,DNAJC18,5.83,4.74,3.44,5.64,4.48,4.38,3.64,6.49,5.93,4.75,6.24,4.81,4.78,4.29,7.65,5.3,5.27,4.28,4.16,3.87,3.6,2.37,0.08,7.81,4.29,2.26,2.64,7.09,4.21,3.18,6.91,4.31,5.156470588,4.070666667,0.01647793,0.174696001

23476,BRD4,21.76,11,15.64,22.94,19.74,15.08,13.76,23.75,29.24,10.92,16.55,17.68,8.03,16.5,16.82,18.85,28.54,10.08,11.48,11.89,17.39,9.94,9.38,15.64,16.57,14.02,13.13,12.51,16.68,15.31,16.26,13.74,18.04705882,13.60133333,0.01647793,0.174696001

55835,CENPJ,1.72,1.64,1.35,2.11,1.48,2.12,1.12,2.21,1.91,2.49,1.2,1.06,1.05,3.94,2.31,1.39,3.58,0.97,1.4,1.07,1.82,1.18,0.41,2.2,1.2,0.93,0.7,2.59,1.15,0.82,1.92,0.98,1.922352941,1.289333333,0.01647793,0.174696001

28962,OSTM1,5.82,1.59,2.13,3.95,6.3,4.56,15.98,3.81,6.81,7.05,5.19,3.2,4.15,5.77,1.57,3.48,13.17,15.45,6.03,5.91,9.19,11.43,4.76,82.81,5.9,13.89,9.86,3.8,5.52,6.81,5.02,9.71,5.560588235,13.07266667,0.01647793,0.174696001

84419,C15orf48,0.27,0,1.6,0.08,1.07,0.18,0,0.21,0.11,0.59,0.28,0.43,1.02,9.61,0.76,0.27,0.69,0.1,0.25,0.17,0,0.71,3.12,0,0.12,0,0.1,0.24,0,0,0.16,0,1.01,0.331333333,0.01648363,0.174696001

56134,PCDHAC2,0.8,0.02,1.04,0.26,0.68,0.11,0.65,0,0.13,0.11,1.27,0.51,0.51,0.15,0.33,0.14,0.37,0.07,0,0,0.41,0.24,0.11,0,0,0,0,0.04,0.68,0.79,0.09,0.26,0.416470588,0.179333333,0.016715411,0.177056179

2646,GCKR,0,0.11,0.03,0,0.11,0,0.75,0,0.04,0.17,0.03,0.08,0,0,0.2,0,0,0.39,1.13,0,0.16,0.48,50.59,0,0.54,0.11,0.42,0.12,0,0.03,0.14,0.24,0.089411765,3.623333333,0.016766031,0.177495891

27128,CYTH4,0,1.4,0.02,0.1,0.02,0.56,0.05,0.08,0.03,0.45,0,0.03,0.14,1.27,0.14,0.12,0.58,0,0,0,0.07,0.06,2.94,0,0.16,0,0,0.04,0,0,0,0.06,0.293529412,0.222,0.016807847,0.177841989

1943,EFNA2,0.1,0.06,0.74,0.4,0.18,0,0,0.04,0.28,0.13,3.58,0.2,0.13,0.37,0.37,0.75,0.77,1.43,0,0,0,0,4.2,0.12,0,0,0.08,0,0.12,0,0.24,0,0.476470588,0.412666667,0.016818308,0.17785612

80032,ZNF556,0,0,0,0.05,0,0,0.14,0,0,0,0,0,0,0,0,0,0,0,0.13,0.05,0,0,0,0.1,0.06,15.35,0,0,0.7,0.04,0.04,0,0.011176471,1.098,0.016847028,0.178048389

4547,MTTP,0,0.01,0,0,0.02,0.05,0.02,0.04,0,0,0.09,0,0,0.21,0,0,0,0.11,0.02,0.19,0,0.04,9.78,0.21,0.02,0.12,0.02,0.06,0.06,0.03,0,0,0.025882353,0.710666667,0.016855561,0.178048389

3174,HNF4G,0.19,0.14,0,0.02,0.17,0.21,0.04,0,0,0.02,0.11,0,0.02,0.49,0.13,0.04,0.07,0,0,0,0,0,2.14,0,0,0.04,0,0.03,0.06,0,0.02,0,0.097058824,0.152666667,0.016863896,0.178048389

10265,IRX5,0,0,0.03,0,0.04,3.47,0.6,2.32,0.94,0,0,0.74,0.92,0.57,0.84,0.11,0.57,2.01,1.4,14.55,0.03,0,0.14,5.15,5.86,0.67,9.27,0.13,3.25,3.12,1.46,0.68,0.655882353,3.181333333,0.016979052,0.179167148

8632,DNAH17,0.27,0.08,0.13,0.29,0.34,0.05,0.05,0.22,0.11,0.18,0.12,0.12,0.05,0.25,0.09,0.1,0.14,0.03,0.05,0.1,0.06,0.03,0.04,0.09,0.22,0.15,0.03,0.15,0.08,0.03,0.18,0.04,0.152352941,0.085333333,0.017070712,0.180036891

22844,FRMPD1,0,0,0,0,0,0,0.02,0,0,0,0,0.02,0,0.1,0,0,0.02,0.03,0,0.03,0,0,0,0,0.02,1.25,0.02,0.03,0.78,2.71,0.16,0,0.009411765,0.335333333,0.017112153,0.180376347

8681,JMJD7-PLA2G4B,1.97,2.52,1.75,0.93,0.62,0,0.62,2.44,1.54,1.8,2.1,2.12,1.48,1.95,1.39,1.99,2.45,0.47,0.95,0,0.72,0,0,3.98,1.31,0.66,0.54,1.61,0.2,0,3.67,1.28,1.627647059,1.026,0.017142144,0.1805325

1048,CEACAM5,0.21,0.06,0.3,0.15,0.17,0.04,0.23,0.07,0.55,0.16,0.23,0,0.13,0.31,0.13,0.06,0.4,0.03,0.09,0.1,0.06,0.07,0.15,0.05,0.14,0.1,0.05,0.11,0.2,0.02,0.12,0.02,0.188235294,0.087333333,0.017244405,0.1805325

286205,SCAI,1.82,2.1,1.48,3.18,2.82,1.61,1.64,2.08,1.76,1.7,1.68,1.46,2.97,1.76,1.88,2.58,1.74,1.35,1.46,1.41,1.48,1.49,1.18,2.4,2.05,1.07,1.69,2.56,1.28,1.8,1.8,1.43,2.015294118,1.63,0.017316137,0.1805325

10773,ZBTB6,4.09,3.45,2.38,3.66,3.24,4.07,2.94,3.97,3.67,2.99,2.69,3.54,2.15,3.67,2.82,4.1,5.41,2.94,2.49,2.7,2.74,1.48,0.85,5.48,3.17,2.78,2.78,3.27,2.77,2.32,3.04,2.99,3.461176471,2.786666667,0.017316137,0.1805325

199746,U2AF1L4,8.44,7.37,5.3,5.46,9.34,5.34,3,5.44,4.3,5.35,8,5.46,2.86,8.71,5.77,6.55,9.86,3.26,5.43,5.06,2.81,1.53,5.11,12.36,8.78,2.57,3.8,5.11,4.93,3.57,5.38,3.97,6.267647059,4.911333333,0.017336654,0.1805325

64084,CLSTN2,56.03,0.74,10.43,0,0.3,19.28,0.44,1.73,22.77,15.09,0.55,7.17,1.67,18.83,6.91,11.47,26.1,17.16,0,0.02,0.2,0.14,0.03,0.09,0.86,50.96,0.23,0.55,5.67,1.36,6.93,0.21,11.73588235,5.627333333,0.017336654,0.1805325

55916,NXT2,3.08,4.63,1.88,3.11,3.95,4.96,2.47,7.7,4.47,4.2,4.59,6.25,5.67,2.97,1.63,1.7,2.4,1.9,2.17,2.37,1.67,1.18,2.28,6.34,2.68,0.91,2.48,2.39,2.15,2.86,3.96,2.68,3.862352941,2.534666667,0.017346916,0.1805325

1789,DNMT3B,0.91,0.78,0.67,7.7,3.71,0.42,0.26,1.07,2.11,0.58,0.75,1.11,1.34,3.59,1.03,0.95,1.4,0.48,0.4,0.33,2.34,0.87,0.04,1.14,0.88,0.48,0.52,0.49,0.65,0.23,1.76,0.27,1.669411765,0.725333333,0.017346916,0.1805325

25829,TMEM184B,54.07,34.53,39.73,41.07,42.03,31.97,50.06,51.29,46.05,41.77,56.09,30.28,28.31,48.3,34.4,31.72,51.29,34.22,37.29,49.86,45.74,61.89,4.11,29.65,28.34,20.5,38.03,28.37,26.74,31.1,33.01,21.39,41.93882353,32.68266667,0.017346916,0.1805325

400506,KNOP1,10.45,12.5,5.73,9.24,8,7.75,6.07,8.48,5.96,13.09,9.16,4.31,2.73,7.3,6,9.8,12.67,6.33,6.23,4.05,12.55,6.03,2.29,7.77,4.87,4.27,5.03,6.36,6.49,5.35,5.77,4.27,8.190588235,5.844,0.017346916,0.1805325

64976,MRPL40,21.25,20.1,20.16,20.36,18.95,33.7,20.5,25.06,20.28,21.25,24.97,25.16,23.92,18.63,19.21,17.52,22.41,22.12,23.22,18.98,25.67,30.89,44.58,38.66,24.43,39.83,21.73,26.69,20.52,21.98,24.72,20.99,21.96647059,27.00066667,0.017346916,0.1805325

729440,CCDC61,2.67,4.78,2.07,5.58,6.21,3.86,2.18,2.48,2.23,3.06,4.64,3.04,2.58,5.13,1.77,2.91,2.8,3.77,4.94,4.23,5.74,2.59,3.3,7.59,5.78,3.66,4.04,4.34,4.16,4.11,6.35,4.11,3.411176471,4.580666667,0.017346916,0.1805325

11277,TREX1,2.8,1.99,2.47,3.43,4.58,2.88,3.83,6.61,3.14,1.97,5.16,3.09,2.19,2.46,1.14,1.34,3.87,2.72,5.5,3.67,6.98,5,1.55,10.81,3.88,4.05,1.93,5.64,4.05,5.09,4.54,5.57,3.114705882,4.732,0.017346916,0.1805325

654,BMP6,0.86,0.98,1.57,44.02,62.17,0.64,2.64,1.13,0.6,0.94,1.44,0.9,0.93,0.81,0.84,0.93,1.05,11.82,27.8,7.59,126.52,86.5,3.78,0.17,0.66,1.04,4.2,2.85,2.28,15.56,1.09,3.69,7.202941176,19.70333333,0.017346916,0.1805325

283971,CLEC18C,0,0.06,0.07,0,0,0,0,0.04,0,0,0.34,0,0,0,0,0,0,2.5,6.9,0,0.07,0.07,0,0.1,0,0.1,0.09,0,0.31,0,0.13,0,0.03,0.684666667,0.017358304,0.1805325

5553,PRG2,0,0.07,0,1.02,0.47,0.18,0,0,0,0.23,0,0,0.11,0.12,0.42,0,0,0,0.73,0.1,0.09,0.22,0.35,0.31,0.12,0.1,0.41,0.86,0.08,0.56,0.3,1.35,0.154117647,0.372,0.017408429,0.1805325

80243,PREX2,0,0,0,1.83,5.62,0,0.06,0,0,0,0,0,0,0,0,0,0,0.33,0.04,0.52,22.77,1.5,0.27,0,0,0.03,0,0,0.02,0.07,0,0.17,0.441764706,1.714666667,0.017423322,0.1805325

80094,BIN3-IT1,0,0,0.03,0,0,0,0,0,0,0,0,0,0,0,0,0,0,0.04,0.04,0,0,0.24,0,0,0,0.09,0,0,0,0.07,0.03,0,0.001764706,0.034,0.01751737,0.1805325

3737,KCNA2,0,0,0,0,0.03,0,0,0,0,0,0,0,0,0,0,0,0,0.12,0,0,0,0,0,0.08,0.05,0.14,0,0.03,0,0,0,0.04,0.001764706,0.030666667,0.017537131,0.1805325

11136,SLC7A9,0.26,0.04,0.07,0.09,0.28,0.04,0.28,0.61,0.19,0.23,0.13,0.15,0.05,0.12,0.1,0,0.22,0,0,0.09,0,0,1.58,0.15,0.06,0.05,0,0.08,0,0.08,0.04,0.16,0.168235294,0.152666667,0.017579524,0.1805325

23566,LPAR3,0.34,0,0.07,0,0,0.33,0,0.05,0.05,0,0.96,0.35,1,0,0.25,1.86,0.11,0,0,0,0,0,0.08,0.3,0,0,0,0.68,0,0,0,0,0.315882353,0.070666667,0.017605605,0.1805325

8642,DCHS1,20.58,40.65,22,51.33,77.58,19.97,0.28,22.24,9.23,15.08,22.94,7.99,23.23,29.91,25.08,28.02,7.31,19.13,11.36,16.99,29.55,25.35,0.62,3.58,2.29,0.98,1.52,22.3,16.39,4.59,19.9,2.08,24.90705882,11.77533333,0.017682737,0.1805325

9338,TCEAL1,8.62,17.04,10.78,24.09,25.48,25.21,8.22,32.62,22.71,11.84,16.77,21.32,13.23,15.47,5.68,8.45,10.18,7.98,6.84,7.75,12.71,5.26,4.4,18.37,13.76,24.45,6.23,11.69,7.38,8.56,9.1,12.53,16.33588235,10.46733333,0.017682737,0.1805325

23635,SSBP2,5.41,14.37,6.44,8.23,8.45,11.39,4.22,8.36,5.19,8.11,6.64,17.91,8.55,9.47,9.26,9.11,3.35,4.4,3.37,7.01,2.25,2.24,0.23,4.65,7.26,4.66,5.35,12.58,5.63,6,6.93,10.17,8.497647059,5.515333333,0.017682737,0.1805325

440456,PLEKHM1P1,6.38,4.15,3.54,9.9,10.21,2.99,2.5,8,4.52,2.86,3.34,2.36,1.5,4.96,4.07,4.28,2.81,1.52,3.16,2.28,2.48,3.29,0.93,5.44,4.38,1.72,1.96,3.79,2.35,2.37,4.45,2.12,4.61,2.816,0.017682737,0.1805325

9820,CUL7,26.17,52.36,23.75,26.59,31.69,31.34,14.84,27.41,18.04,37.96,29.27,27.93,26.64,34.67,30.35,29.82,19.04,14.8,30.81,11.83,19.69,8.44,2.21,16.21,32.67,11.77,15.93,55.7,15.67,13.11,37.71,17.84,28.69823529,20.29266667,0.017682737,0.1805325

6451,SH3BGRL,58.69,79.29,92.97,96.6,106.33,108.92,61.99,73.46,80.3,57.25,106.45,92.66,74.5,63.8,55.01,59.44,54.92,98.55,35.87,54.14,43.15,53.8,8.63,111.54,67.85,34.2,59.14,68.75,46.95,52.71,75.16,68.18,77.79882353,58.57466667,0.017682737,0.1805325

6414,SEPP1,0.17,2.46,3.09,3.08,10.36,12.13,0.72,1.3,1.34,1.85,102.65,0.87,0.25,19.21,1.82,3.58,0.84,1.49,14.94,25.95,1.71,0.2,168.74,7.4,43.32,10.24,8.53,151.79,1.68,53.6,40.78,55.99,9.748235294,39.09066667,0.017682737,0.1805325

84914,ZNF587,4.09,3.02,4.38,7.4,5.75,3.01,3.74,5.22,4.56,3.95,3.91,3.49,4.32,7.62,4.44,4.93,5.17,3.51,3.61,2.02,4.13,3.76,1.85,7.55,4.62,2.97,1.93,4.46,2.67,2.52,4.75,1.96,4.647058824,3.487333333,0.017682737,0.1805325

26502,NARF,46.87,47.28,23.85,54.44,63.1,30.78,18.66,40.89,26.14,35.58,27.27,28.07,26.1,32.89,29.87,31.24,18.09,21.36,13.14,18.53,26.01,27.67,26.86,82.14,21.17,21.34,17.97,35.48,25.14,14.69,33.68,20.16,34.18352941,27.02266667,0.017682737,0.1805325

56478,EIF4ENIF1,7.83,5.61,6.2,13.18,14.59,9.52,7.46,9.71,10.73,7.74,11.12,8.84,6.62,10.56,7.28,8.65,10.79,7.11,4.61,5.82,9.18,8.4,2.65,12.42,6.3,7.44,5.56,9.2,6.01,6.28,9.97,5.71,9.201764706,7.110666667,0.017682737,0.1805325

149951,COMMD7,17.51,11.41,16.84,54.49,42.97,17.34,19.11,23.61,21.53,15.74,20.26,31.12,19.09,32.37,13.98,17.27,19.59,16.82,8.28,14.24,31.78,28.19,9.74,21.16,16.15,10.2,14.93,10.28,14.02,10.87,32.14,13.48,23.19,16.81866667,0.017682737,0.1805325

23473,CAPN7,9.17,6.01,8.3,8.24,7.86,8.6,9.25,9.79,10.32,10.43,9.62,6,4.53,8.56,8.78,9.59,9.07,6.42,6.8,7.91,7.98,4.24,3.99,10.9,7.56,5.99,6.36,9.99,6.9,7.09,7.47,7.21,8.477647059,7.120666667,0.017682737,0.1805325

3632,INPP5A,18,5.85,9.37,12.32,10.09,8.28,8.6,25.02,19.36,7.16,13.97,10.34,8.25,12.73,13.04,6.81,15.09,7.13,4.62,10.21,8.01,7.39,2.05,10.8,4.8,7.19,5.28,9.86,9.02,9.9,16.37,8.17,12.01647059,8.053333333,0.017682737,0.1805325

81034,SLC25A32,9.09,6.85,10.25,13.23,12.24,7.85,7.87,7.99,7.38,11.22,6,10.32,14.93,15.84,12.35,6.82,13.35,5.65,9.86,6.95,10.83,8.29,5.81,7.45,7.49,7.95,6.11,6.25,7.37,7.69,8.16,8.47,10.21058824,7.622,0.017682737,0.1805325

2976,GTF3C2,16.37,14.79,13.25,17.66,16.51,19.99,8.75,15.8,12.16,15.9,20.03,15.49,12.06,18.81,15.59,13.24,17.52,13.58,10.53,9.58,17.4,11.33,3.58,20.28,13.64,10.97,10.64,15.52,10.48,9.84,16.41,9.7,15.52470588,12.232,0.017682737,0.1805325

23457,ABCB9,2.61,2.5,4.15,4.82,2.2,2.96,1.94,3.24,1.71,2.71,6.09,2.73,2.67,3.53,4.37,2.16,1.68,1.47,2.42,0.99,3.57,1.83,0.08,3.48,3.3,1.5,2.01,2.85,1.52,1.09,1.96,2.22,3.062941176,2.019333333,0.017682737,0.1805325

55578,SUPT20H,15.2,12.33,13.46,19.72,17.74,11.29,9.21,15.25,16.31,11.46,12.11,9.14,6.94,20.22,12.63,12.39,17.82,12.15,7.95,9.1,15.14,12.64,3.23,17.57,10.93,9.81,8.16,11.37,10.55,6.17,14.86,8.62,13.71882353,10.55,0.017682737,0.1805325

57418,WDR18,32.37,16.08,19.9,37.25,34.7,26.81,16.97,31.68,30.19,18.98,25.62,16.91,11.73,31.61,29.12,20.64,27.18,17.74,10.91,17.42,25.5,22.48,21.91,18.75,15.99,17.36,16.69,12.33,20.25,20.95,18.71,22.99,25.16117647,18.66533333,0.017682737,0.1805325

2256,FGF11,13.32,11.77,9.27,1.09,2.76,6.64,12.33,1.28,2.75,21.82,9.3,15.73,4.33,11.58,26.26,10.96,0.59,4.06,2.98,0.34,0.45,0.41,0.25,0.12,4.94,7.72,0.51,10.97,15.19,2.78,5.96,7.12,9.516470588,4.253333333,0.017682737,0.1805325

6392,SDHD,41.91,38.53,34.45,45.67,43.86,71.76,36.65,49.89,29.84,35.02,54.43,41.61,37.04,43.43,27.17,32.24,36.8,31.52,70.32,48.78,56.29,40.56,75.74,67.05,49.62,59.82,35.37,50.02,37.56,45.22,46.66,50.57,41.19411765,51.00666667,0.017682737,0.1805325

65244,SPATS2,13.7,18.13,14.27,12.46,13.49,11.69,8.11,12.23,8.2,20.78,9.73,11.4,14.92,11.06,12.33,14.65,6.47,8.82,6.64,6.33,18.27,29.08,1.39,8.67,9.32,8.42,4.8,11.42,8.75,5.85,12.21,8.43,12.56588235,9.893333333,0.017682737,0.1805325

83862,TMEM120A,8.4,17.45,9.04,14.24,19.83,16.85,10.15,11.18,8.81,16.67,13.98,14.88,16.23,11.9,9.34,8.31,10.02,8.33,17.89,21.32,77.57,14.05,23.4,40.08,25.76,32.82,8.71,14.82,12.59,17.02,12.87,17.4,12.78117647,22.97533333,0.017682737,0.1805325

8624,PSMG1,20.51,9.38,21.25,21.12,18.09,20.57,19.15,26.52,22.49,14.27,14.36,23.64,12.6,20.59,21.35,15.02,23.5,9.53,17.19,13.74,29.05,17.61,21.49,16.56,10.32,13.37,9.06,7.41,15.36,14.07,18.35,11.98,19.08294118,15.006,0.017682737,0.1805325

6284,S100A13,84.86,116.89,87.96,47.52,46.1,142.08,82.86,93.15,62.61,70.3,61.77,93.72,202.12,80.8,78.06,85.49,59.13,109.99,90.66,152.13,126.19,92.46,6.74,107.05,86.75,183.4,119.82,112.45,98.23,136.54,152.44,75.87,87.96588235,110.048,0.017682737,0.1805325

28998,MRPL13,13.22,10.98,14.65,18.4,13.81,20.27,17.58,15.77,12.51,13.01,15.99,14.58,14.57,14.96,11.38,10.81,14.7,21.59,15.68,15.82,23.32,17.7,19.85,19.45,15.28,17.86,15.17,11.67,15.03,11.62,20.52,14.68,14.54058824,17.016,0.017682737,0.1805325

9143,SYNGR3,1.65,1.42,1.37,0.26,0.19,4.4,0.68,0.52,1.56,2.52,4.78,1.74,1.05,2.83,1.22,1.66,2.82,0.79,1.85,0.04,0.37,0.21,0.14,0.13,1.52,1.02,0.08,2.99,0.41,0.36,2.16,0.63,1.804117647,0.846666667,0.017682737,0.1805325

22808,MRAS,8.75,0.57,5.8,0.46,0.73,5.68,12.2,5.51,10.57,4.14,26.39,1.13,3.06,7.3,5.72,1.12,8.22,17.43,7.9,15.43,3.8,3.2,0.29,19.14,14.49,175.71,16.33,6.66,25.45,12.11,12.43,5.95,6.314705882,22.42133333,0.017682737,0.1805325

4717,NDUFC1,23.42,18.39,25.41,29.01,30.15,44.53,27.05,26.89,27.3,27.66,31.32,30.98,27.41,28.52,18.76,19.49,22.65,36.8,33.66,31.85,53.7,41.93,22.96,41.83,28.45,36.47,27.98,30.19,21.04,23.87,29.95,32.97,26.99647059,32.91,0.017682737,0.1805325

10458,BAIAP2,9.58,7.3,10.14,6.96,13.31,9.82,4.8,9.31,13.35,12.1,11.63,5.66,7.4,16.39,8.01,7.73,9.49,11.87,7.27,9.13,4.94,5.5,13.38,54.61,3.51,7.19,4.25,5.8,5.32,5.28,4.55,1.44,9.587058824,9.602666667,0.017682737,0.1805325

2242,FES,3.48,5.19,1.42,17.18,18.34,4.26,1.82,4.6,1.32,1.73,2.87,4.13,1.5,3.4,1.38,3.21,0.73,0.95,6.32,5.16,21.39,11.94,6.54,12.71,10.68,1.09,4.1,6.13,4.05,7.12,8.33,5.49,4.503529412,7.466666667,0.017682737,0.1805325

2013,EMP2,3.61,2.1,5.21,7.55,7.18,4.17,1.39,8.95,4.29,3.53,29.65,8.66,5.88,8.58,3.86,6.82,5.05,5.01,21.79,12.08,4.86,1.53,8.27,6.28,25.5,18.93,9.09,6.92,8.41,10.14,12.2,22.21,6.851764706,11.548,0.017682737,0.1805325

3693,ITGB5,72.92,19.31,73.74,33.15,26.79,71.79,56.69,79.2,80,35.1,199.51,82.01,92.49,46.92,17.81,38.55,31.55,312.18,92.2,201.34,59.55,147.55,17.27,35.79,169.34,102.87,172.19,24.44,86.98,166.33,81.61,88.95,62.20764706,117.2393333,0.017682737,0.1805325

4692,NDN,32.94,15.09,16.28,3.77,4.98,0.83,2.77,0.13,0.23,8.46,40.33,15,1.11,13.77,15.14,10.64,16.49,31.42,21.45,38.42,20.72,4.47,0.37,14.11,17.21,18.01,8.93,74,32.36,40.15,34.35,13.29,11.64470588,24.61733333,0.017682737,0.1805325

290,ANPEP,39.7,89.14,13.17,7.69,4.85,26.91,69.6,106.68,11.01,135.32,29.86,0.16,1.11,14.38,50.67,56.71,13.49,6.26,68.97,187.57,34.33,273.08,106.51,1.12,150.98,63.36,94.9,52.14,34.16,121.23,122.01,71.01,39.43823529,92.50866667,0.017682737,0.1805325

1534,CYB561,6.2,4.61,3.93,12.03,8.59,10.18,8.37,10.5,3.29,8.46,11.85,1.23,1.02,6.57,4.51,1.5,2.78,8.08,6.47,14.35,19.6,9.37,18.98,6.82,13.21,8.4,11.46,4.39,5.28,23.88,8.71,7.47,6.212941176,11.098,0.017682737,0.1805325

2,A2M,0.11,0.15,1.01,24.77,42.48,1.92,0.72,0.05,0.02,0.38,96.57,19.61,7.07,64.54,29.99,3.28,0.04,17.39,43.71,8.36,18.43,4.97,335.58,284.37,1.66,21.16,1.14,590.17,5.84,44.25,17.11,122.53,17.21823529,101.1113333,0.017682737,0.1805325

5092,PCBD1,12.3,5.54,11.18,19.56,7.97,9.22,22.29,20.63,18.1,17.44,23.14,14.19,23.64,25.26,7.65,5.38,10.42,26.62,8.98,18.56,71.85,42.33,119.75,23.62,13.08,28.74,17.89,20.69,16.91,16.79,28.73,17.42,14.93588235,31.464,0.017682737,0.1805325

790952,ESRG,0.66,0.77,0.87,1.34,1.65,0.65,1.79,1.87,3.21,2.76,0.52,0.92,1.38,2.12,0.58,0.78,4.31,0.67,1.52,0.43,0.48,0.69,1.11,1.56,1.01,0.54,0.47,0.82,0.46,0.95,0.79,0.36,1.54,0.790666667,0.017682737,0.1805325

316,AOX1,1.42,0.89,0.16,0.02,0.05,21.82,10.36,0.59,1.23,6.58,0.21,1.59,2.39,1.15,3.17,0.17,2.47,0.47,55.71,19.47,0.11,13.62,129,0.03,16.77,24.76,5.56,5.46,3.84,20.78,2.29,6.24,3.192352941,20.274,0.017682737,0.1805325

200150,PLD5,0,0,0,0,0,0,0.03,0,0,0,0,0,0,1.14,0,0,0,0.14,0,0.03,0,0,0,0,0.07,0.03,0,0.21,1.25,0.02,0.12,0,0.068823529,0.124666667,0.017899468,0.182649541

167838,TXLNB,0.03,0.03,0,0.21,0.16,0,0.03,0.24,0.04,0.06,0.54,0.2,0.81,0.06,0.02,0.02,0,0.1,0.01,0.54,0.44,0.7,0.03,0.05,2.53,23.53,0.03,2.74,0.33,0.33,0.65,0.07,0.144117647,2.138666667,0.017969249,0.183265653

9468,PCYT1B,2.25,0.05,0.77,0.08,0.1,0.12,0.17,0.76,0.18,0.04,3.68,2.45,0.44,0.04,0.23,0.51,0.22,0.86,0,0.04,0.12,0,0.03,0.1,0.33,0.08,0,0,0.26,0.04,0.3,0.11,0.711176471,0.151333333,0.018022181,0.183709371

64409,WBSCR17,0,0,0,0,0,0,0,0,0.02,0,0,0.02,0.26,0,0,0,0,18.61,0,0,0,0,0,0,0.17,6.97,0,0.15,0.85,2.91,0.5,0.02,0.017647059,2.012,0.018097489,0.184380581

3235,HOXD9,0,0,0,0,0.04,0,2.19,0,0,0.05,0,0.14,1.3,0.1,0,0,0,0,3.45,7.96,1,0,0,0.46,0.16,0.86,0.7,0,1.99,2.44,2.27,0,0.224705882,1.419333333,0.018218127,0.184642554
[truncated: 3,676,893 more chars]
