# Supplementary material for: Use of deep neural network ensembles to identify embryonic-fetal transition markers: repression of COX7A1 in embryonic and cancer cells
Source: Oncotarget. 2017 Dec 28;9(8):7796–811. doi: 10.18632/oncotarget.23748 (PMC5814259; doi:10.18632/oncotarget.23748)
Supplement: Supplementary file 3 [file oncotarget-09-7796-s003.doc]

CLASS,DATASET,PLATFORM,SAMPLE,TITLE / SOURCE NAME,,

ESC,BIOTIME,GPL10588,MBA_688,EH2 P9 matrigel (prop mefs prev),,

iPSC,BIOTIME,GPL10588,MBA_689,ESI 035 P19 mefs,,

iPSC,BIOTIME,GPL10588,MBA_695,E164 P14 ctrl (RGD expt),,

iPSC,BIOTIME,GPL10588,MBA_696,E164 P14 D7 (RGD) C.S. MM,,

iPSC,BIOTIME,GPL10588,MBA_697,E164 P14 D14 C.S. MM (RGD expt),,

iPSC,BIOTIME,GPL10588,MBA_698,E164 P14 D14 alginate (RGD expt),,

iPSC,BIOTIME,GPL10588,MBA_699,Z11 P14 ctrl (RGD expt),,

iPSC,BIOTIME,GPL10588,MBA_700,Z11 P14 D7 C.S. MM (RGD expt),,

iPSC,BIOTIME,GPL10588,MBA_701,Z11 P14 D16 C.S. MM (RGD expt),,

iPSC,BIOTIME,GPL10588,MBA_702,W10 P15 ctrl (RGD expt),,

iPSC,BIOTIME,GPL10588,MBA_703,W10 P15 D7 alginate (RGD expt),,

iPSC,BIOTIME,GPL10588,MBA_704,W10 P15 D7 C.S. MM (RGD expt),,

iPSC,BIOTIME,GPL10588,MBA_705,W10 P15 D14 C.S. MM (RGD expt),,

iPSC,BIOTIME,GPL10588,MBA_706,E15 P18 ctrl (RGD expt),,

iPSC,BIOTIME,GPL10588,MBA_707,E15 P18 D7 C.S. MM (RGD expt) ,,

iPSC,BIOTIME,GPL10588,MBA_708,E15 P18 alginate D7 (RGD expt) ,,

iPSC,BIOTIME,GPL10588,MBA_709,E15 P18 D14 C.S. MM (RGD expt),,

iPSC,BIOTIME,GPL10588,MBA_710,E15 P18 D14 Alginate (RGD expt),,

iPSC,BIOTIME,GPL10588,MBA_712,SM30 P12 D14 alginate stability,,

iPSC,BIOTIME,GPL10588,MBA_713,SM30 P12 D14 MM (chondro stability),,

iPSC,BIOTIME,GPL10588,MBA_714,7PEND24 P16 ctrl (RGD expt),,

iPSC,BIOTIME,GPL10588,MBA_715,7PEND24 P16 D7 RGD- Alginate (RGD expt),,

iPSC,BIOTIME,GPL10588,MBA_716,7PEND24 P16 D7 C.S. MM (RGD expt),,

iPSC,BIOTIME,GPL10588,MBA_717,7PEND24 P16 D14 C.S. MM (RGD expt),,

iPSC,BIOTIME,GPL10588,MBA_718,7PEND24 P16 D14 RGD- alginate (RGD expt),,

iPSC,BIOTIME,GPL10588,MBA_720,4D20.8 P15 C.S. D14 (chondro stability),,

iPSC,BIOTIME,GPL10588,MBA_721,4D20.8 P15 D14 alginate (chondro stability),,

iPSC,BIOTIME,GPL10588,MBA_722,4D20.8 P15 D14 MM (chondro stability),,

iPSC,BIOTIME,GPL10588,MBA_723,4D20.8 P15 ctrl (RGD expt),,

iPSC,BIOTIME,GPL10588,MBA_724,4D20.8 P15 D7 C.S. MM (RGD expt),,

iPSC,BIOTIME,GPL10588,MBA_725,4D20.8 P15 D7 alginate (RGD expt),,

iPSC,BIOTIME,GPL10588,MBA_726,4D20.8 P15 D14 C.S. MM (RGD expt),,

iPSC,BIOTIME,GPL10588,MBA_727,4D20.8 P15 D14 alginate (RGD expt),,

iPSC,BIOTIME,GPL10588,MBA_729,4D20.8 P33 (Stability) Chondro D14 C.S. MM,,

iPSC,BIOTIME,GPL10588,MBA_730,4D20.8 P33 (Stability) Chondro D14 alginate,,

iPSC,BIOTIME,GPL10588,MBA_731,4D20.8 P33 (Stability) Chondro D14 MM,,

iPSC,BIOTIME,GPL10588,MBA_732,7SMOO32 P13 ctrl (RGD expt),,

iPSC,BIOTIME,GPL10588,MBA_733,7SMOO32 P13 D7 C.S. MM (RGD Expt),,

iPSC,BIOTIME,GPL10588,MBA_734,7SMOO32 P13 D7 alginate (RGD Expt),,

iPSC,BIOTIME,GPL10588,MBA_735,7SMOO32 P13 D14 C. S. MM (RGD expt),,

iPSC,BIOTIME,GPL10588,MBA_736,7SMOO32 P13 D14 RGD-alginate,,

iPSC,BIOTIME,GPL10588,MBA_738,CMO2 P22 D7 C.S. MM,,

iPSC,BIOTIME,GPL10588,MBA_739,CMO2 P22 D14 C.S. MM,,

iPSC,BIOTIME,GPL10588,MBA_740,CMO2 P22 D14 (RGD-alginate),,

iPSC,BIOTIME,GPL10588,MBA_741,7SMOO32 P14 ctrl (LONZA),,

iPSC,BIOTIME,GPL10588,MBA_742,7SMOO32 P14 D14 Lonza-alginate,,

iPSC,BIOTIME,GPL10588,MBA_743,7SMOO32 P14 D14 MM,,

iPSC,BIOTIME,GPL10588,MBA_744,E33 P17 ctrl ,,

iPSC,BIOTIME,GPL10588,MBA_745,E33 P17 D7 C.S. MM (RGD expt),,

iPSC,BIOTIME,GPL10588,MBA_746,E33 P17 D7 RGD alginate (RGD expt),,

iPSC,BIOTIME,GPL10588,MBA_747,E33 P17 D14 C.S. MM (RGD expt),,

iPSC,BIOTIME,GPL10588,MBA_748,E33 P17 D14 RGD alginate (RGD expt),,

iPSC,BIOTIME,GPL10588,MBA_749,Z11 P15 DE2.1 gel ctrl,,

iPSC,BIOTIME,GPL10588,MBA_750,Z11 P15 DE2.3 FN ctrl,,

iPSC,BIOTIME,GPL10588,MBA_751,Z11 P15 DE2.5,,

iPSC,BIOTIME,GPL10588,MBA_752,Z11 P15 DE2.6,,

iPSC,BIOTIME,GPL10588,MBA_753,Z11 P15 DE2.7,,

iPSC,BIOTIME,GPL10588,MBA_754,Z11 P15 DE2.8,,

iPSC,BIOTIME,GPL10588,MBA_755,Z11 P15 DE2.9,,

iPSC,BIOTIME,GPL10588,MBA_756,Z11 P15 DE2.10,,

iPSC,BIOTIME,GPL10588,MBA_757,Z11 P15 DE2.11,,

iPSC,BIOTIME,GPL10588,MBA_758,Z11 P15 DE2.12,,

iPSC,BIOTIME,GPL10588,MBA_759,MEL2 P22 DE2.1 gel ctrl,,

iPSC,BIOTIME,GPL10588,MBA_760,MEL2 P22 DE2.1 gel ctrl,,

iPSC,BIOTIME,GPL10588,MBA_761,MEL2 P22 DE2.5,,

iPSC,BIOTIME,GPL10588,MBA_762,MEL2 P22 DE2.6,,

iPSC,BIOTIME,GPL10588,MBA_763,MEL2 P22 DE2.7,,

iPSC,BIOTIME,GPL10588,MBA_764,MEL2 P22 DE2.8,,

iPSC,BIOTIME,GPL10588,MBA_765,MEL2 P22 DE2.9,,

iPSC,BIOTIME,GPL10588,MBA_766,MEL2 P22 DE2.10,,

iPSC,BIOTIME,GPL10588,MBA_767,MEL2 P22 DE2.11,,

iPSC,BIOTIME,GPL10588,MBA_768,MEL2 P22 DE2.12,,

iPSC,BIOTIME,GPL10588,MBA_769,7PEND24 P12 DE2.1 gel ctrl,,

iPSC,BIOTIME,GPL10588,MBA_770,7PEND24 P12 DE2.3 FN ctrl,,

iPSC,BIOTIME,GPL10588,MBA_771,7PEND24 P12 DE2.5,,

iPSC,BIOTIME,GPL10588,MBA_772,7PEND24 P12 DE2.6,,

iPSC,BIOTIME,GPL10588,MBA_773,7PEND24 P12 DE2.7,,

iPSC,BIOTIME,GPL10588,MBA_774,7PEND24 P12 DE2.8,,

iPSC,BIOTIME,GPL10588,MBA_775,7PEND24 P12 DE2.9,,

iPSC,BIOTIME,GPL10588,MBA_776,7PEND24 P12 DE2.10,,

iPSC,BIOTIME,GPL10588,MBA_777,7PEND24 P12 DE2.11,,

iPSC,BIOTIME,GPL10588,MBA_778,7PEND24 P12 DE2.12,,

iPSC,BIOTIME,GPL10588,MBA_780,4SKEL20 P12 DE2.3 FN ctrl,,

iPSC,BIOTIME,GPL10588,MBA_781,4SKEL20 P12 DE2.5,,

iPSC,BIOTIME,GPL10588,MBA_782,4SKEL20 P12 DE2.6,,

iPSC,BIOTIME,GPL10588,MBA_783,4SKEL20 P12 DE2.7,,

iPSC,BIOTIME,GPL10588,MBA_784,4SKEL20 P12 DE2.8,,

iPSC,BIOTIME,GPL10588,MBA_785,4SKEL20 P12 DE2.9,,

iPSC,BIOTIME,GPL10588,MBA_786,4SKEL20 P12 DE2.10,,

iPSC,BIOTIME,GPL10588,MBA_787,4SKEL20 P12 DE2.11,,

iPSC,BIOTIME,GPL10588,MBA_788,4SKEL20 P12 DE2.12,,

iPSC,BIOTIME,GPL10588,MBA_790,SM30 P33 D14 MM chondro stability,,

iPSC,BIOTIME,GPL10588,MBA_792,SM30 P15 D14 MM CSE,,

iPSC,BIOTIME,GPL10588,MBA_793,SM30 P15 alginate D14 ,,

iPSC,BIOTIME,GPL10588,MBA_794,SM30 P15 ctrl RS_45 CSE,,

iPSC,BIOTIME,GPL10588,MBA_795,SM30 P15 D7 C.S. MM (RGD expt),,

iPSC,BIOTIME,GPL10588,MBA_796,SM30 P15 D7 RGD alginate (RGD expt),,

iPSC,BIOTIME,GPL10588,MBA_797,SM30 P15 D14 C.S. MM (RGD-alginate),,

iPSC,BIOTIME,GPL10588,MBA_798,SM30 P15 D14 RGD-alginate,,

iPSC,BIOTIME,GPL10588,MBA_799,SM30 P15 D14 MM (RGD expt),,

iPSC,BIOTIME,GPL10588,MBA_800,SM30 P17 ctrl in-vivo construct,,

iPSC,BIOTIME,GPL10588,MBA_801,"SM30 P17 D14 alginate (Lonza, const. expt)",,

iPSC,BIOTIME,GPL10588,MBA_802,SM30 P17 D14 RGD-alginate (const. expt),,

iPSC,BIOTIME,GPL10588,MBA_803,SM30 P22 ctrl (chondro stability),,

iPSC,BIOTIME,GPL10588,MBA_804,SM30 P22 D14 alg,,

iPSC,BIOTIME,GPL10588,MBA_805,SM30 P22 D14 RGD-alg,,

iPSC,BIOTIME,GPL10588,MBA_806,SM30 P22 D14 MM,,

iPSC,BIOTIME,GPL10588,MBA_807,7PEND24 P14 Chondro ctrl CSE,,

iPSC,BIOTIME,GPL10588,MBA_809,"7PEND24 P21 D14 alginate (Lonza, const expt)",,

iPSC,BIOTIME,GPL10588,MBA_810,7PEND24 P21 D14 RGD-alginate (const expt),,

iPSC,BIOTIME,GPL10588,MBA_811,4D20.8 P15 ctrl (in-vivo alg contruct study),,

iPSC,BIOTIME,GPL10588,MBA_812,"4D20.8 P14 D14 Alginate (Lonza, construct expt)",,

iPSC,BIOTIME,GPL10588,MBA_813,4D20.8 P14 D14 RGD-alginate (construct expt),,

iPSC,BIOTIME,GPL10588,MBA_815,"E15 P19 D14 alginate (Lonza, construct expt)",,

iPSC,BIOTIME,GPL10588,MBA_816,E15 P19 D14 RGD-alginate (construct expt),,

iPSC,BIOTIME,GPL10588,MBA_817,SK11 P13 ctrl (chondro MM alg study),,

iPSC,BIOTIME,GPL10588,MBA_818,SK11 P13 D14 C.S. MM (RGD expt),,

iPSC,BIOTIME,GPL10588,MBA_819,SK11 P13 D14 RGD-alginate (RGD expt),,

iPSC,BIOTIME,GPL10588,MBA_820,"SK11 P13 D14 alginate (Lonza, RGD expt)",,

iPSC,BIOTIME,GPL10588,MBA_821,SK11 P13 D14 RGD- alginate,,

iPSC,BIOTIME,GPL10588,MBA_822,SK11 P13 D14 alginate (Lonza),,

iPSC,BIOTIME,GPL10588,MBA_823,SK11 P13 D14 MM,,

AC,BIOTIME,GPL10588,MBA_825,"Xgene FB P17 alginate Lonza, constr expt)",,

iPSC,BIOTIME,GPL10588,MBA_827,Z1 P18 D7 alginate (RGD expt),,

iPSC,BIOTIME,GPL10588,MBA_828,Z1 P18 D7 C.S. MM (RGD expt),,

iPSC,BIOTIME,GPL10588,MBA_829,Z1 P18 D14 C.S. MM (RGD expt),,

iPSC,BIOTIME,GPL10588,MBA_830,Z1 P18 D14 RGD-alginate,,

iPSC,BIOTIME,GPL10588,MBA_831,4D20.8 P42 ctrl chondro (stability),,

iPSC,BIOTIME,GPL10588,MBA_832,4D20.8 P42 D14 alginate (stability),,

iPSC,BIOTIME,GPL10588,MBA_833,4D20.8 P42 D14 MM (stability),,

iPSC,BIOTIME,GPL10588,MBA_835,7SMOO32 P13 D14 alginate,,

iPSC,BIOTIME,GPL10588,MBA_836,7SMOO32 P13 D14 RGD-alginate (VLVG),,

iPSC,BIOTIME,GPL10588,MBA_837,7SMOO32 P13 D14 MM,,

iPSC,BIOTIME,GPL10588,MBA_839,W10 P14 D14 alg (stability),,

iPSC,BIOTIME,GPL10588,MBA_840,W10 P14 D14 MM (stability),,

AC,BIOTIME,GPL10588,MBA_841,MSC2 P7 ctrl (in-vivo alg construct study),,

AC,BIOTIME,GPL10588,MBA_842,"MSC2 P7 D14 alginate (Lonza, constr expt)",,

AC,BIOTIME,GPL10588,MBA_843,MSC2 P7 D14 RGD- alginate (constr expt),,

AC,BIOTIME,GPL10588,MBA_844,MSC1 P23 CTRL CHONDRO STABILITY,,

AC,BIOTIME,GPL10588,MBA_845,MSC1 P23 D14 ALGINATE CHONDRO STABILITY,,

AC,BIOTIME,GPL10588,MBA_846,MSC1 P23 D14 MM,,

AC,BIOTIME,GPL10588,MBA_848,MSC1 P13 D14 C.S. MM STABILITY,,

AC,BIOTIME,GPL10588,MBA_849,MSC1 P13 D14 ALGINATE STABILITY,,

AC,BIOTIME,GPL10588,MBA_850,MSC1 P13 D14 MM STABILITY,,

iPSC,BIOTIME,GPL10588,MBA_851,E15 P16 ctrl (chondro stability),,

iPSC,BIOTIME,GPL10588,MBA_852,E15 P16 D14 RGD-alg (chrondro stability),,

iPSC,BIOTIME,GPL10588,MBA_853,E15 P16 D14 alg (chondro stability),,

iPSC,BIOTIME,GPL10588,MBA_854,E15 P16 D14 MM (chondro stability),,

iPSC,BIOTIME,GPL10588,MBA_855,E15 P33 ctrl (chondro stability),,

iPSC,BIOTIME,GPL10588,MBA_856,E15 P33 D14 alginate (chondro stability),,

iPSC,BIOTIME,GPL10588,MBA_857,E15 P33 D14 RGD-alginate,,

iPSC,BIOTIME,GPL10588,MBA_858,E15 P33 D14 MM (chondro stability),,

iPSC,BIOTIME,GPL10588,MBA_877,ESI 035 P30 evacuated hypotonic ReCyte Dry P3,,

iPSC,BIOTIME,GPL10588,MBA_878,RASMO12 P17 D4 (in nephro med low attach_ prev in RPTEC medium_A),,

iPSC,BIOTIME,GPL10588,MBA_879,RASMO12 P17 D4 (in nephro med low attach_prev in RPTEC + cond med_B),,

iPSC,BIOTIME,GPL10588,MBA_880,RASMO12 P17 D4 (in nephro med low attach_prev in RPTEC + SM med_C),,

iPSC,BIOTIME,GPL10588,MBA_881,RASMO12 P17 A media (renal only ctrl),,

iPSC,BIOTIME,GPL10588,MBA_882,RASMO12 P17 B media (renal and renal conditioned with RPTEC),,

iPSC,BIOTIME,GPL10588,MBA_883,RASMO12 P17 C media (renal and smooth muscle),,

iPSC,BIOTIME,GPL10588,MBA_884,"RASMO12 P17 Nephrosphere D7 on gelatin coating cond A,B,C combine",,

iPSC,BIOTIME,GPL10588,MBA_886,RASMO12 P14 DE2.3 FN CTRL,,

iPSC,BIOTIME,GPL10588,MBA_888,SM35 P12 DE2.3 FN ctrl,,

iPSC,BIOTIME,GPL10588,MBA_889,SM35 P12 DE2.5,,

iPSC,BIOTIME,GPL10588,MBA_890,SM35 P12 DE2.6,,

iPSC,BIOTIME,GPL10588,MBA_891,SM35 P12 DE2.7,,

iPSC,BIOTIME,GPL10588,MBA_892,SM35 P12 DE2.8,,

iPSC,BIOTIME,GPL10588,MBA_893,SM35 P12 DE2.9,,

iPSC,BIOTIME,GPL10588,MBA_894,SM35 P12 DE2.10,,

iPSC,BIOTIME,GPL10588,MBA_895,SM35 P12 DE2.11,,

iPSC,BIOTIME,GPL10588,MBA_896,SM35 P12 DE2.12,,

iPSC,BIOTIME,GPL10588,MBA_898,T36 P19 DE2.3 FN ctrl,,

iPSC,BIOTIME,GPL10588,MBA_899,T36 P19 DE 2.5,,

iPSC,BIOTIME,GPL10588,MBA_900,T36 P19 DE 2.6,,

iPSC,BIOTIME,GPL10588,MBA_901,T36 P19 DE 2.10,,

iPSC,BIOTIME,GPL10588,MBA_902,E69 P15 DE2 ctrl FN,,

iPSC,BIOTIME,GPL10588,MBA_904,E69 P15 DE2.5,,

iPSC,BIOTIME,GPL10588,MBA_905,E69 P15 DE2.8,,

iPSC,BIOTIME,GPL10588,MBA_906,E69 P15 DE2.10,,

iPSC,BIOTIME,GPL10588,MBA_907,E69 P15 DE2.11,,

iPSC,BIOTIME,GPL10588,MBA_908,E69 P15 DE2.12,,

iPSC,BIOTIME,GPL10588,MBA_910,W11 P12 DE2.3 FN ctrl,,

iPSC,BIOTIME,GPL10588,MBA_911,W11 P12 DE2.5,,

iPSC,BIOTIME,GPL10588,MBA_912,W11 P12 DE2.7,,

iPSC,BIOTIME,GPL10588,MBA_913,W11 P12 DE2.8,,

iPSC,BIOTIME,GPL10588,MBA_914,W11 P12 DE2.9,,

iPSC,BIOTIME,GPL10588,MBA_915,W11 P12 DE2.10,,

iPSC,BIOTIME,GPL10588,MBA_916,W11 P12 DE2.11,,

iPSC,BIOTIME,GPL10588,MBA_917,W11 P12 DE2.12,,

iPSC,BIOTIME,GPL10588,MBA_919,Z2 P12 DE2.3 FN ctrl,,

iPSC,BIOTIME,GPL10588,MBA_920,Z2 P12 DE2.5,,

iPSC,BIOTIME,GPL10588,MBA_921,Z2 P12 DE2.6,,

iPSC,BIOTIME,GPL10588,MBA_922,Z2 P12 DE2.10,,

iPSC,BIOTIME,GPL10588,MBA_923,C4.4 P14 DE2.1 gel ctrl,,

iPSC,BIOTIME,GPL10588,MBA_924,C4.4 P14 DE2.3 FN ctrl,,

iPSC,BIOTIME,GPL10588,MBA_925,C4.4 P14 DE2.5,,

iPSC,BIOTIME,GPL10588,MBA_926,C4.4 P14 DE2.7,,

iPSC,BIOTIME,GPL10588,MBA_927,C4.4 P14 DE2.8,,

iPSC,BIOTIME,GPL10588,MBA_928,C4.4 P14 DE2.9,,

iPSC,BIOTIME,GPL10588,MBA_929,C4.4 P14 DE2.10,,

iPSC,BIOTIME,GPL10588,MBA_930,C4.4 P14 DE2.11,,

iPSC,BIOTIME,GPL10588,MBA_931,C4.4 P14 DE2.12,,

iPSC,BIOTIME,GPL10588,MBA_933,SK17 P15 DE2.3 ctrl FN,,

iPSC,BIOTIME,GPL10588,MBA_934,SK17 P15 DE2.5,,

iPSC,BIOTIME,GPL10588,MBA_935,SK17 P15 DE2.8,,

iPSC,BIOTIME,GPL10588,MBA_936,SK17 P15 DE2.10,,

iPSC,BIOTIME,GPL10588,MBA_937,SK17 P15 DE2.12,,

iPSC,BIOTIME,GPL10588,MBA_939,7SMOO7 P18 DE2.3 FN ctrl,,

iPSC,BIOTIME,GPL10588,MBA_940,7SMOO7 P18 DE2.5,,

iPSC,BIOTIME,GPL10588,MBA_941,7SMOO7 P18 DE2.6,,

iPSC,BIOTIME,GPL10588,MBA_942,7SMOO7 P18 DE2.7,,

iPSC,BIOTIME,GPL10588,MBA_943,7SMOO7 P18 DE2.8,,

iPSC,BIOTIME,GPL10588,MBA_944,7SMOO7 P18 DE2.9,,

iPSC,BIOTIME,GPL10588,MBA_945,7SMOO7 P18 DE2.10,,

iPSC,BIOTIME,GPL10588,MBA_946,7SMOO7 P18 DE2.11,,

iPSC,BIOTIME,GPL10588,MBA_947,7SMOO7 P18 DE2.12,,

iPSC,BIOTIME,GPL10588,MBA_949,SK31 P16 DE2.1 ctrl FN,,

iPSC,BIOTIME,GPL10588,MBA_950,SK31 P16 DE2.5,,

iPSC,BIOTIME,GPL10588,MBA_951,SK31 P16 DE2.6,,

iPSC,BIOTIME,GPL10588,MBA_952,SK31 P16 DE2.7,,

iPSC,BIOTIME,GPL10588,MBA_953,SK31 P16 DE2.8,,

iPSC,BIOTIME,GPL10588,MBA_954,SK31 P16 DE2.9,,

iPSC,BIOTIME,GPL10588,MBA_955,SK31 P16 DE2.10,,

iPSC,BIOTIME,GPL10588,MBA_956,SK31 P16 DE2.11,,

iPSC,BIOTIME,GPL10588,MBA_957,SK31 P16 DE2.12,,

iPSC,BIOTIME,GPL10588,MBA_959,T43 P17 DE2.3 FN ctrl,,

iPSC,BIOTIME,GPL10588,MBA_960,T43 P17 DE2.5 ,,

iPSC,BIOTIME,GPL10588,MBA_961,T43 P17 DE2.6,,

iPSC,BIOTIME,GPL10588,MBA_962,T43 P17 DE2.7,,

iPSC,BIOTIME,GPL10588,MBA_963,T43 P17 DE2.8,,

iPSC,BIOTIME,GPL10588,MBA_964,T43 P17 DE2.9,,

iPSC,BIOTIME,GPL10588,MBA_965,T43 P17 DE2.10,,

iPSC,BIOTIME,GPL10588,MBA_966,T43 P17 DE2.11,,

iPSC,BIOTIME,GPL10588,MBA_967,T43 P17 DE2.12,,

iPSC,BIOTIME,GPL10588,MBA_968,E44 P18 DE2.1 gel ctrl,,

iPSC,BIOTIME,GPL10588,MBA_969,E44 P18 DE2.3 FN ctrl,,

iPSC,BIOTIME,GPL10588,MBA_970,E44 P18 DE2.5,,

iPSC,BIOTIME,GPL10588,MBA_971,E44 P18 DE2.6,,

iPSC,BIOTIME,GPL10588,MBA_972,E44 P18 DE2.7,,

iPSC,BIOTIME,GPL10588,MBA_973,E44 P18 DE2.8,,

iPSC,BIOTIME,GPL10588,MBA_974,E44 P18 DE2.9,,

iPSC,BIOTIME,GPL10588,MBA_975,E44 P18 DE2.10,,

iPSC,BIOTIME,GPL10588,MBA_976,E44 P18 DE2.11,,

iPSC,BIOTIME,GPL10588,MBA_977,E44 P18 DE2.12,,

iPSC,BIOTIME,GPL10588,MBA_982,Z1 P14 DE2.1 gel ctrl,,

iPSC,BIOTIME,GPL10588,MBA_983,Z1 P14 DE2.3 FN ctrl,,

iPSC,BIOTIME,GPL10588,MBA_984,Z1 P14 DE2.6,,

iPSC,BIOTIME,GPL10588,MBA_985,Z1 P14 DE2.7,,

iPSC,BIOTIME,GPL10588,MBA_986,Z1 P14 DE2.8,,

iPSC,BIOTIME,GPL10588,MBA_987,Z1 P14 DE2.9,,

iPSC,BIOTIME,GPL10588,MBA_988,Z1 P14 DE2.10,,

iPSC,BIOTIME,GPL10588,MBA_989,Z1 P14 DE2.11,,

iPSC,BIOTIME,GPL10588,MBA_990,Z1 P14 DE2.12,,

iPSC,BIOTIME,GPL10588,MBA_991,Z11 P11 ctrl (chondro stability),,

iPSC,BIOTIME,GPL10588,MBA_992,Z11 P11 D14 alginate (chondro stability),,

iPSC,BIOTIME,GPL10588,MBA_993,Z11 P11 D14 RGD-VLVG alginate (chondro stability),,

iPSC,BIOTIME,GPL10588,MBA_994,Z11 P11 D14 MM (chondro stability),,

iPSC,BIOTIME,GPL10588,MBA_996,Z11 P25 D14 alginate (chondro stability),,

iPSC,BIOTIME,GPL10588,MBA_997,Z11 P25 D14 RGD-alg (chondro stability),,

iPSC,BIOTIME,GPL10588,MBA_998,Z11 P25 D14 MM (chondro stability),,

iPSC,BIOTIME,GPL10588,MBA_1000,W10 P29 D14 MM (chondro stability),,

iPSC,BIOTIME,GPL10588,MBA_1002,E15 P24 D14 alg (chondro stability),,

iPSC,BIOTIME,GPL10588,MBA_1003,E15 P24 D14 MM (chondro stability),,

iPSC,BIOTIME,GPL10588,MBA_1005,MEL2 P18 D14 alg,,

iPSC,BIOTIME,GPL10588,MBA_1006,MEL2 P18 D14 RGD-alg,,

iPSC,BIOTIME,GPL10588,MBA_1007,MEL2 P18 D14 MM,,

iPSC,BIOTIME,GPL10588,MBA_1009,SM30 P14 D14 RGD-alg,,

iPSC,BIOTIME,GPL10588,MBA_1010,SM30 P14 D14 MM,,

iPSC,BIOTIME,GPL10588,MBA_1012,7PEND24 P22 D14 alg,,

iPSC,BIOTIME,GPL10588,MBA_1013,7PEND24 P22 D14 RGD-alg,,

iPSC,BIOTIME,GPL10588,MBA_1014,7PEND24 P22 D14 MM,,

iPSC,BIOTIME,GPL10588,MBA_1015,SK31 P14 ctrl (chondro stability),,

iPSC,BIOTIME,GPL10588,MBA_1016,SK31 P14 D14 MM (chondro stability),,

AC,BIOTIME,GPL10588,MBA_1017,MSC1 P30 ctrl (chondro stability),,

AC,BIOTIME,GPL10588,MBA_1018,MSC1 P30 D14 MM (chondro stability),,

iPSC,BIOTIME,GPL10588,MBA_1020,4D20.8 P17 D14 alg,,

iPSC,BIOTIME,GPL10588,MBA_1021,4D20.8 P17 D14 RGD-alg 2x,,

iPSC,BIOTIME,GPL10588,MBA_1022,4D20.8 P17 D14 Hystem,,

iPSC,BIOTIME,GPL10588,MBA_1023,4D20.8 P17 D14 MM,,

iPSC,BIOTIME,GPL10588,MBA_1025,B28 P14 D14 alg ,,

iPSC,BIOTIME,GPL10588,MBA_1026,B28 P14 D14 MM,,

iPSC,BIOTIME,GPL10588,MBA_1028,E15 P21 D14 alg,,

iPSC,BIOTIME,GPL10588,MBA_1029,E15 P21 D14 HyStem,,

iPSC,BIOTIME,GPL10588,MBA_1030,E15 D14 P21 MM,,

iPSC,BIOTIME,GPL10588,MBA_1032,SK11 P21 D14 Hystem,,

iPSC,BIOTIME,GPL10588,MBA_1034,7SMOO32 P13 D14 Hystem ,,

iPSC,BIOTIME,GPL10588,MBA_1035,7SMOO32 P13 D14 MM,,

iPSC,BIOTIME,GPL10588,MBA_1037,SM30 P16 D14 Hystem ,,

iPSC,BIOTIME,GPL10588,MBA_1038,SM30 P16 D14 RGD-alg 2x,,

AC,BIOTIME,GPL10588,MBA_1040,NHAC P15 D14 alg (chondro stability),,

AC,BIOTIME,GPL10588,MBA_1041,NHAC P15 D14 MM (chondro stability),,

AC,BIOTIME,GPL10588,MBA_1043,NHAC 18 D14 alg (chondro stability),,

AC,BIOTIME,GPL10588,MBA_1044,NHAC P18 D14 MM (chondro stability),,

AC,BIOTIME,GPL10588,MBA_1046,CASMC P14 D14 alg (chondro stability),,

AC,BIOTIME,GPL10588,MBA_1047,CASMC P14 D14 MM (chondro stability),,

AC,BIOTIME,GPL10588,MBA_1049,CASMC P19 D14 alg (chondro stability),,

AC,BIOTIME,GPL10588,MBA_1050,CASMC P19 D14 MM (chondro stability),,

iPSC,BIOTIME,GPL10588,MBA_1052,MEL2 P19 D14 RGD-alg,,

iPSC,BIOTIME,GPL10588,MBA_1053,MEL2 P19 D14 HyStem,,

iPSC,BIOTIME,GPL10588,MBA_1054,MEL2 P19 D14 MM,,

iPSC,BIOTIME,GPL10588,MBA_1055,U31 P17 PromoCell MSC media ctrl,,

iPSC,BIOTIME,GPL10588,MBA_1056,U31 P17 MesenCult ctrl,,

iPSC,BIOTIME,GPL10588,MBA_1057,U31 P17 PromoCell (18 days chondro no TGF),,

iPSC,BIOTIME,GPL10588,MBA_1058,U31 P17 MesenCult (18 days chondro no TGF),,

ESC,BIOTIME,GPL10588,MBA_1059,IMR90 EB larocca P3 matrigel mTeSR1,,

iPSC,BIOTIME,GPL10588,MBA_1561,7PEND24 P23 chondro Hystem ,,

iPSC,BIOTIME,GPL10588,MBA_1562,7PEND24 P23 RGD-alg2x,,

iPSC,BIOTIME,GPL10588,MBA_1563,7PEND24 P23 alg,,

iPSC,BIOTIME,GPL10588,MBA_1565,SM30 P21 D14 alg chondro stability,,

iPSC,BIOTIME,GPL10588,MBA_1566,SM30 P21 D14 RGD-alg2x chondro stability,,

iPSC,BIOTIME,GPL10588,MBA_1567,SM30 P21 D14 MM chondro stability,,

iPSC,BIOTIME,GPL10588,MBA_1569,W10 P43 D14 alg chondro stability,,

iPSC,BIOTIME,GPL10588,MBA_1570,W10 P43 D14 RGD-alg2x chondro stability,,

iPSC,BIOTIME,GPL10588,MBA_1571,W10 P43 D14 MM chondro stability,,

AC,BIOTIME,GPL10588,MBA_1573,NHAC P21 D14 alg chondro stability,,

AC,BIOTIME,GPL10588,MBA_1574,NHAC P21 D14 RGD-alg2x chondro stability,,

AC,BIOTIME,GPL10588,MBA_1575,NHAC P21 D14 MM chondro stability,,

iPSC,BIOTIME,GPL10588,MBA_1577,Z11 P40 D14 MM chondro stability,,

iPSC,BIOTIME,GPL10588,MBA_1579,EN7 P14 D15 chondro Hystem,,

iPSC,BIOTIME,GPL10588,MBA_1580,EN7 P14 D28 RA 1uM Hystem,,

iPSC,BIOTIME,GPL10588,MBA_1581,EN7 P14 D28 EGF100ng/ml Hystem,,

iPSC,BIOTIME,GPL10588,MBA_1582,EN7 P14 D28 EGF 100ng/ml confluence,,

iPSC,BIOTIME,GPL10588,MBA_1584,RAD20.6 P15 D15 chondro RGD-alg2x,,

iPSC,BIOTIME,GPL10588,MBA_1585,RAD20.6 P15 D28 RA 1uM RGD-alg2x,,

iPSC,BIOTIME,GPL10588,MBA_1586,RAD20.6 P15 D28 EGF 100ng/ml RGD-alg2x,,

iPSC,BIOTIME,GPL10588,MBA_1588,RAPEND15 P17 D14 chondro RGD-alg2x,,

iPSC,BIOTIME,GPL10588,MBA_1589,RAPEND15 P17 D28 RA 1uM RGD-alg2x,,

iPSC,BIOTIME,GPL10588,MBA_1590,RAPEND15 P17 D28 EGF 100ng/ml RGD-alg2x,,

iPSC,BIOTIME,GPL10588,MBA_1591,RAPEND15 P17 D28 EGF 100ng/ml confluence ,,

iPSC,BIOTIME,GPL10588,MBA_1593,EN2 P14 D14 chondro Hystem,,

iPSC,BIOTIME,GPL10588,MBA_1594,EN2 P14 D28 RA 1uM Hystem ,,

iPSC,BIOTIME,GPL10588,MBA_1595,EN2 P14 D28 EGF 100ng/ml Hystem,,

iPSC,BIOTIME,GPL10588,MBA_1596,EN2 P14 D28 EGF 100ng/ml confluence,,

iPSC,BIOTIME,GPL10588,MBA_1598,RASMO19 P14 D14 Hystem,,

iPSC,BIOTIME,GPL10588,MBA_1599,RASMO19 P14 D28 RA 1uM Hystem,,

iPSC,BIOTIME,GPL10588,MBA_1600,RASMO19 P14 D28 EGF 100ng/ml Hystem,,

iPSC,BIOTIME,GPL10588,MBA_1601,RASMO19 P14 D28 EGF 100ng/ml confluence,,

iPSC,BIOTIME,GPL10588,MBA_1603,Z1 P17 D14 chondro Hystem,,

iPSC,BIOTIME,GPL10588,MBA_1604,Z1 P17 D28 RA 1uM Hystem ,,

iPSC,BIOTIME,GPL10588,MBA_1605,Z1 P17 D28 EGF 100ng/ml Hystem ,,

iPSC,BIOTIME,GPL10588,MBA_1606,Z1 P17 D28 EGF 100ng/ml confluence ,,

iPSC,BIOTIME,GPL10588,MBA_1608,F15 P20 D14 chondro Hystem ,,

iPSC,BIOTIME,GPL10588,MBA_1609,F15 P20 D28 RA 1uM Hystem ,,

iPSC,BIOTIME,GPL10588,MBA_1610,F15 P20 D28 EGF 100ng/ml Hystem,,

iPSC,BIOTIME,GPL10588,MBA_1611,F15 P20 D28 EGF 100ng/ml confluence,,

iPSC,BIOTIME,GPL10588,MBA_1613,RAD20.5 P14 D14 chondro Hystem,,

iPSC,BIOTIME,GPL10588,MBA_1614,RAD20.5 P14 D28 RA 1uM Hystem,,

iPSC,BIOTIME,GPL10588,MBA_1615,RAD20.5 P14 D28 EGF 100ng/ml Hystem,,

iPSC,BIOTIME,GPL10588,MBA_1616,RAD20.5 P14 D28 EGF 100ng/ml (confluence),,

iPSC,BIOTIME,GPL10588,MBA_1618,SM22 P16 D14 chondro Hystem,,

iPSC,BIOTIME,GPL10588,MBA_1619,SM22 P16 D28 RA Hystem,,

iPSC,BIOTIME,GPL10588,MBA_1620,SM22 P16 D28 EGF Hystem,,

iPSC,BIOTIME,GPL10588,MBA_1621,SM22 P16 D28 EGF confluence,,

iPSC,BIOTIME,GPL10588,MBA_1622,ESI 035 P49 mefs for P10 (previously on matrigel),,

iPSC,BIOTIME,GPL10588,MBA_1650,W10 P10 TGFb Inhib,,

iPSC,BIOTIME,GPL10588,MBA_1658,7PEND24 P22 ctrl (cartilage repair expt),,

iPSC,BIOTIME,GPL10588,MBA_1659,7PEND24 P22 D14 RGD-alg2x cartilage repair,,

iPSC,BIOTIME,GPL10588,MBA_1660,7PEND24 P22 D14 Hystem cartilage repair,,

iPSC,BIOTIME,GPL10588,MBA_1661,7PEND24 P22 D14 Pellet cartilage repair,,

iPSC,BIOTIME,GPL10588,MBA_1662,SM30 P17 ctrl (cartilage repair expt),,

iPSC,BIOTIME,GPL10588,MBA_1663,SM30 P17 D14 RGD-alg2x cartilage repair,,

iPSC,BIOTIME,GPL10588,MBA_1664,SM30 P17 D14 Hystem cartilage repair,,

iPSC,BIOTIME,GPL10588,MBA_1665,SM30 P17 Pellet cartilage repair,,

iPSC,BIOTIME,GPL10588,MBA_1666,4D20.8 P18 ctrl chondro cartilage repair,,

iPSC,BIOTIME,GPL10588,MBA_1667,4D20.8 P18 D14 RGD-alg2x cartilage repair,,

iPSC,BIOTIME,GPL10588,MBA_1668,4D20.8 P18 D14 Pellet cartilage repair,,

iPSC,BIOTIME,GPL10588,MBA_1669,4D20.8 P18 D14 Hystem cartilage repair,,

AC,BIOTIME,GPL10588,MBA_1670,MSC2 P10 ctrl chondro cartilage repair,,

AC,BIOTIME,GPL10588,MBA_1671,MSC2 P10 D14 RGD-alg2x cartilage repair,,

AC,BIOTIME,GPL10588,MBA_1672,MSC2 P10 D14 Hystem cartilage repair,,

AC,BIOTIME,GPL10588,MBA_1673,MSC2 P10 D14 Pellet cartilage repair,,

iPSC,BIOTIME,GPL10588,MBA_1674,E15 P17 ctrl Chondro cartiliage repair,,

iPSC,BIOTIME,GPL10588,MBA_1675,E15 P17 D14 RGD-alg2x cartilage repair,,

iPSC,BIOTIME,GPL10588,MBA_1676,E15 P17 D14 Pellet cartilage repair,,

iPSC,BIOTIME,GPL10588,MBA_1677,E15 P17 D14 Hystem cartilage repair,,

AC,BIOTIME,GPL10588,MBA_1678,CASMC P27 ctrl (chondro stability),,

AC,BIOTIME,GPL10588,MBA_1679,CASMC P27 D14 MM chondro stability,,

iPSC,BIOTIME,GPL10588,MBA_1681,EN16 P15 D14 chondro Hystem,,

iPSC,BIOTIME,GPL10588,MBA_1682,EN16 P15 D28 RA 1uM Hystem,,

iPSC,BIOTIME,GPL10588,MBA_1683,EN16 P15 D28 EGF 100ng/ml Hystem,,

iPSC,BIOTIME,GPL10588,MBA_1684,EN16 P15 D28 EGF 100ng/ml confluence,,

iPSC,BIOTIME,GPL10588,MBA_1686,B16 P19 D14 chondro Hystem,,

iPSC,BIOTIME,GPL10588,MBA_1687,B16 P19 D28 RA 1uM Hystem,,

iPSC,BIOTIME,GPL10588,MBA_1688,B16 P19 D28 EGF 100ng/ml ,,

iPSC,BIOTIME,GPL10588,MBA_1689,B16 P19 D28 EGF 100ng/ml confluence,,

iPSC,BIOTIME,GPL10588,MBA_1691,E44 P20 D19 chondro Hystem,,

iPSC,BIOTIME,GPL10588,MBA_1692,E44 P20 D28 EGF 100ng/ml Hystem,,

iPSC,BIOTIME,GPL10588,MBA_1693,E44 P20 D33 EGF 100ng/ml confluence,,

iPSC,BIOTIME,GPL10588,MBA_1695,E33 P18 D14 Hystem chondro ,,

iPSC,BIOTIME,GPL10588,MBA_1696,E33 P18 D28 RA 1uM Hystem,,

iPSC,BIOTIME,GPL10588,MBA_1697,E33 P18 D28 EGF 100ng/ml Hystem,,

iPSC,BIOTIME,GPL10588,MBA_1698,E33 P18 D28 EGF 100ng/ml confluence,,

iPSC,BIOTIME,GPL10588,MBA_1700,RASMO12 P18 D14 Hystem Chondro,,

iPSC,BIOTIME,GPL10588,MBA_1701,RASMO12 P18 D28 RA 1uM Hystem,,

iPSC,BIOTIME,GPL10588,MBA_1702,RASMO12 P18 D28 EGF 100ng/ml Hystem,,

iPSC,BIOTIME,GPL10588,MBA_1703,RASMO12 P18 D28 EGF 100ng/ml confluence,,

iPSC ,BIOTIME,GPL10588,MBA_1704,17PENDX11 P5,,

iPSC ,BIOTIME,GPL10588,MBA_1705,17SMOOX4 P6,,

iPSC ,BIOTIME,GPL10588,MBA_1706,17PENDX1 P6,,

iPSC ,BIOTIME,GPL10588,MBA_1707,17SKELX21 P6 Good grower,,

iPSC ,BIOTIME,GPL10588,MBA_1708,17SKELX23 P6 Good grower,,

iPSC,BIOTIME,GPL10588,MBA_1711,EN1 P14 D28 RA 1uM Hystem,,

iPSC,BIOTIME,GPL10588,MBA_1712,EN1 P14 D28 EGF 100ng/ml Hystem,,

iPSC,BIOTIME,GPL10588,MBA_1713,EN1 P14 D28 EGF 100ng/ml confluence,,

iPSC,BIOTIME,GPL10588,MBA_1714,EN1 P14 D14 Hystem chondro,,

iPSC,BIOTIME,GPL10588,MBA_1716,E120 P18 D28 EGF 100ng/ml confluence,,

iPSC,BIOTIME,GPL10588,MBA_1717,E120 P18 D14 Hystem chondro,,

iPSC,BIOTIME,GPL10588,MBA_1718,E111 P18 ctrl Hystem,,

iPSC,BIOTIME,GPL10588,MBA_1719,E111 P18 D28 RA 1uM Hystem,,

iPSC,BIOTIME,GPL10588,MBA_1720,E111 P18 D28 EGF 100ng/ml confluence,,

iPSC,BIOTIME,GPL10588,MBA_1721,RASKEL8 P16 D14 MM,,

iPSC,BIOTIME,GPL10588,MBA_1723,RASKEL8 P16 D28 EGF 100ng/ml confluence,,

iPSC,BIOTIME,GPL10588,MBA_1725,T7 P18 D28 EGF 100ng/ml confluence,,

iPSC,BIOTIME,GPL10588,MBA_1727,U31 P20 D14 Hystem chondro,,

iPSC,BIOTIME,GPL10588,MBA_1728,U31 P20 D28 RA 1uM hystem,,

iPSC,BIOTIME,GPL10588,MBA_1729,U31 P20 D28 EGF 100ng/ml Hystem,,

iPSC,BIOTIME,GPL10588,MBA_1730,U31 P20 D28 EGF 100ng/ml confluence,,

iPSC,BIOTIME,GPL10588,MBA_1732,SK31 P18 D14 MM (chondro stability),,

iPSC,BIOTIME,GPL10588,MBA_1734,SK31 P22 D14 Alg (chondro stability),,

iPSC,BIOTIME,GPL10588,MBA_1735,SK31 P22 D14 MM Chondro stability,,

iPSC,BIOTIME,GPL10588,MBA_1736,E15 P18 ctrl Chondro Growth Factor,,

iPSC,BIOTIME,GPL10588,MBA_1737,E15 P18 D14 Hystem 5H (100ng/ml BMP7 no TGFb3),,

iPSC,BIOTIME,GPL10588,MBA_1738,E15 P18 D14 Hystem 6H (10ng/ml TGFb3),,

iPSC,BIOTIME,GPL10588,MBA_1739,E15 P18 D14 Hystem 7H (10ng/ml TGFb3 + 100ng/ml BMP7),,

iPSC,BIOTIME,GPL10588,MBA_1740,E15 P18 D14 Hystem 8H (25ng/ml TGFb3),,

iPSC,BIOTIME,GPL10588,MBA_1741,4D20.8 P18 ctrl BMP growth factor expt,,

iPSC,BIOTIME,GPL10588,MBA_1742,4D20.8 P18 D14 Alg 1A (BMP7 TGFb expt),,

iPSC,BIOTIME,GPL10588,MBA_1743,4D20.8 P18 D14 Alg 2A,,

iPSC,BIOTIME,GPL10588,MBA_1744,4D20.8 P18 D14 Alg 3A (BMP7 TGFb expt),,

iPSC,BIOTIME,GPL10588,MBA_1745,4D20.8 P18 D14 Alg 4A,,

iPSC,BIOTIME,GPL10588,MBA_1746,4D20.8 P16 ctrl normal growth,,

iPSC,BIOTIME,GPL10588,MBA_1747,4D20.8 P16 ctrl FGF2,,

iPSC,BIOTIME,GPL10588,MBA_1748,4D20.8 P16 D14 alg normal growth,,

iPSC,BIOTIME,GPL10588,MBA_1749,4D20.8 P16 D14 alg + FGF2 10ng/ml during growth 1,,

iPSC,BIOTIME,GPL10588,MBA_1750,4D20.8 P16 D14 MM +FGF2 10ng/ml during growth 1,,

iPSC,BIOTIME,GPL10588,MBA_1751,4D20.8 P16 D14 MM normal growth 1,,

iPSC ,BIOTIME,GPL10588,MBA_1765,17SKEL-X2 P24,,

iPSC ,BIOTIME,GPL10588,MBA_1766,17SKEL-X23 P28,,

iPSC ,BIOTIME,GPL10588,MBA_1767,17PEND-X11 P32,,

iPSC ,BIOTIME,GPL10588,MBA_1768,17SKEL-X2 P6,,

iPSC,BIOTIME,GPL10588,MBA_1775,4D20.8 P19 ctrl + FGF2 10ng/ml round 2,,

iPSC,BIOTIME,GPL10588,MBA_1777,4D20.8 P19 D14 alg no FGF2 during growth round 2,,

iPSC,BIOTIME,GPL10588,MBA_1778,4D20.8 P19 D14 alg + FGF2 10ng/ml round 2,,

iPSC,BIOTIME,GPL10588,MBA_1779,4D20.8 P19 D14 MM + FGF2 10ng/ml round 2,,

iPSC,BIOTIME,GPL10588,MBA_1780,4D20.8 P19 D14 MM normal round 2,,

iPSC,BIOTIME,GPL10588,MBA_1781,4D20.8 P19 D14 confluence + FGF2 10mg/ml round 2,,

iPSC,BIOTIME,GPL10588,MBA_1782,4D20.8 P19 D14 confluence normal round 2,,

iPSC,BIOTIME,GPL10588,MBA_1784,4D20.8 P20 D14 Hystem BMP7 100ng/ml,,

iPSC,BIOTIME,GPL10588,MBA_1785,4D20.8 P20 D14 Hystem TGFb3 10ng/ml,,

iPSC,BIOTIME,GPL10588,MBA_1786,4D20.8 P20 D14 Hystem TGFb3 10ng/ml + BMP7 100ng/ml,,

iPSC,BIOTIME,GPL10588,MBA_1787,4D20.8 P20 D14 Hystem TGFb3 25ng/ml,,

iPSC,BIOTIME,GPL10588,MBA_1788,4D20.8 P20 D14 Hystem BMP4 10ng/ml,,

iPSC,BIOTIME,GPL10588,MBA_1791,4D20.8 P23 D14 Hystem BMP7 100ng/ml,,

iPSC,BIOTIME,GPL10588,MBA_1792,4D20.8 P23 D14 Hystem TGFb3 10ng/ml,,

iPSC,BIOTIME,GPL10588,MBA_1793,4D20.8 P23 D14 Hystem BMP7 100ng/ml + TGFb3 10ng/ml,,

iPSC,BIOTIME,GPL10588,MBA_1794,4D20.8 P23 D14 Hystem TGFb3 25ng/ml,,

iPSC,BIOTIME,GPL10588,MBA_1795,4D20.8 P23 D14 Hystem BMP4 10ng/ml,,

iPSC,BIOTIME,GPL10588,MBA_1797,4D20.8 P26 + FGF2 10ng/ml ctrl (FGF2 expt) ,,

iPSC,BIOTIME,GPL10588,MBA_1798,4D20.8 P26 no FGF2 ctrl (FGF2 expt),,

iPSC,BIOTIME,GPL10588,MBA_1799,4D20.8 P26 D14 alg no FGF2,,

iPSC,BIOTIME,GPL10588,MBA_1800,4D20.8 P26 D14 alg + FGF2 10ng/ml,,

iPSC,BIOTIME,GPL10588,MBA_1801,4D20.8 P26 D14 MM no FGF2,,

iPSC,BIOTIME,GPL10588,MBA_1802,4D20.8 P26 D14 MM + FGF2 10ng/ml,,

iPSC,BIOTIME,GPL10588,MBA_1817,E15 P22 ctrl (Hystem BMP expt),,

iPSC,BIOTIME,GPL10588,MBA_1818,E15 P22 D14 Chondro Hystem BMP7 100ng/ml,,

iPSC,BIOTIME,GPL10588,MBA_1819,E15 P22 D14 Chondro Hystem TGFb3 10ng/ml,,

iPSC,BIOTIME,GPL10588,MBA_1820,E15 P22 D14 Chondro Hystem BMP7 100ng/ml + TGFb3 10ng/ml,,

iPSC,BIOTIME,GPL10588,MBA_1821,E15 P22 D14 Chondro Hystem BMP4 10ng/ml + TGFb3 10ng/ml,,

iPSC,BIOTIME,GPL10588,MBA_1822,E3 P10 chondro wide screen ctrl,,

iPSC,BIOTIME,GPL10588,MBA_1823,E3 P10 D14 MM wide screen,,

iPSC,BIOTIME,GPL10588,MBA_1824,7PEND24 P20 ctrl (Hystem BMP expt),,

iPSC,BIOTIME,GPL10588,MBA_1825,7PEND24 P20 Chondro Hystem 100ng/ml BMP7,,

iPSC,BIOTIME,GPL10588,MBA_1826,7PEND24 P20 Chondro Hystem TGFb3 10ng/ml,,

iPSC,BIOTIME,GPL10588,MBA_1827,7PEND24 P20 Chondro Hystem 100ng/ml BMP7 +TBFb3 10ng/ml,,

iPSC,BIOTIME,GPL10588,MBA_1828,7PEND24 P20 Chondro Hystem 10ng/ml BMP4 + TGFb3 10ng/ml,,

iPSC,BIOTIME,GPL10588,MBA_1830,4D20.8 P36 with bFGF 10nM,,

iPSC,BIOTIME,GPL10588,MBA_1831,4D20.8 P36 D14 alg +FGF2 Growth media,,

iPSC,BIOTIME,GPL10588,MBA_1832,4D20.8 P36 D14 alg no FGF,,

iPSC,BIOTIME,GPL10588,MBA_1833,4D20.8 P36 D14 MM +FGF2 Growth media,,

iPSC,BIOTIME,GPL10588,MBA_1834,4D20.8 P36 D14 MM no FGF,,

iPSC,BIOTIME,GPL10588,MBA_1835,4D20.8 P20 ctrl Hystem,,

iPSC,BIOTIME,GPL10588,MBA_1836,4D20.8 P20 D14 Hystem B7T10,,

iPSC,BIOTIME,GPL10588,MBA_1837,4D20.8 P20 D14 Hystem B4 10ng/ml,,

iPSC,BIOTIME,GPL10588,MBA_1838,4D20.8 P20 D14 Hystem B2 50ng/ml,,

iPSC,BIOTIME,GPL10588,MBA_1839,4D20.8 P20 Hystem B2T,,

AC,BIOTIME,GPL10588,MBA_1840,knNHAC P8 6 tubes for chondro qPCR control,,

iPSC,BIOTIME,GPL10588,MBA_1841,CM50-5 P11 ctrl wide screen,,

iPSC,BIOTIME,GPL10588,MBA_1842,CM50-5 P11 D15 MM,,

iPSC,BIOTIME,GPL10588,MBA_1843,B11 P17 ctrl wide screen,,

iPSC,BIOTIME,GPL10588,MBA_1844,B11 P17 D14 MM,,

iPSC,BIOTIME,GPL10588,MBA_1846,E163 P11 D14 MM,,

iPSC,BIOTIME,GPL10588,MBA_1847,CMO2 P16 ctrl epithelial expt,,

iPSC,BIOTIME,GPL10588,MBA_1848,"CMO2 P16 matrigel Fast media with SB431542 10uM, ROCK inh 10uM, EGF 100ng/ml 14 days",,

iPSC,BIOTIME,GPL10588,MBA_1849,"CMO2 P16 matrigel Fast media with SB431542 10uM, ROCK inh 10uM 14 days",,

iPSC,BIOTIME,GPL10588,MBA_1850,CMO2 P16 matrigel Fast media with SB431542 10uM 14 days,,

iPSC,BIOTIME,GPL10588,MBA_1851,"CMO2 P16 matrigel TGFinhibitor study with SB431542 10uM, ROCK inh 10uM, EGF 100ng/ml 14 days with fast in growth to confl",,

iPSC,BIOTIME,GPL10588,MBA_1852,"CMO2 P16 matrigel TGFinhibitor study with SB431542 10uM, ROCK inh 10uM, 14 days with fast in growth to confl",,

iPSC,BIOTIME,GPL10588,MBA_1853,E164 P19 ctrl epithelial expt,,

iPSC,BIOTIME,GPL10588,MBA_1854,"E164 P19 matrigel Fast media with SB431542 10uM, ROCK inh 10uM, EGF 100ng/ml 14 days",,

iPSC,BIOTIME,GPL10588,MBA_1855,"E164 P19 matrigel Fast media with SB431542 10uM, ROCK inh 10uM 14 days",,

iPSC,BIOTIME,GPL10588,MBA_1856,E164 P19 matrigel Fast media with SB431542 10uM 14 days,,

iPSC,BIOTIME,GPL10588,MBA_1857,"E164 P19 matrigel TGFinhibitor study with SB431542 10uM, ROCK inh 10uM, EGF 100ng/ml 14 days with fast in growth to confl",,

iPSC,BIOTIME,GPL10588,MBA_1858,"E164 P19 matrigel TGFinhibitor study with SB431542 10uM, ROCK inh 10uM, 14 days with fast in growth to confl",,

iPSC,BIOTIME,GPL10588,MBA_1859,MEL2 P19 ctrl TGFinhibitor,,

iPSC,BIOTIME,GPL10588,MBA_1860,"MEL2 P19 matrigel Fast media with SB431542 10uM, ROCK inh 10uM, EGF 100ng/ml 14 days",,

iPSC,BIOTIME,GPL10588,MBA_1861,"MEL2 P19 matrigel Fast media with SB431542 10uM, ROCK inh 10ul 14 days",,

iPSC,BIOTIME,GPL10588,MBA_1862,MEL2 P19 matrigel Fast media with SB431542 10uM 14 days,,

iPSC,BIOTIME,GPL10588,MBA_1863,"MEL2 P19 matrigel TGFinhibitor study with SB431542 10uM, ROCK inh 10uM, EGF 100ng/ml 14 days with fast in growth to confl",,

iPSC,BIOTIME,GPL10588,MBA_1864,"MEL2 P19 matrigel TGFinhibitor study with SB431542 10uM, ROCK inh 10uM 14 days with fast in growth to confl",,

iPSC,BIOTIME,GPL10588,MBA_1865,E33 P18 ctrl TGFinhibitor,,

iPSC,BIOTIME,GPL10588,MBA_1866,"E33 P18 matrigel Fast media with SB431542 10uM, ROCK inh 10uM, EGF 100ng/ml 14 days",,

iPSC,BIOTIME,GPL10588,MBA_1867,"E33 P18 matrigel Fast media with SB431542 10uM, ROCK inh 10uM 14 days",,

iPSC,BIOTIME,GPL10588,MBA_1868,E33 P18 matrigel Fast media with SB431542 10uM 14 days,,

iPSC,BIOTIME,GPL10588,MBA_1869,"E33 P18 matrigel TGFinhibitor study with SB431542 10uM, ROCK inh 10uM, EGF 100ng/ml 14 days with fast in growth to confl",,

iPSC,BIOTIME,GPL10588,MBA_1870,"E33 P18 matrigel TGFinhibitor study with SB431542 10uM, ROCK inh 10uM 14 days with fast in growth to confl",,

iPSC,BIOTIME,GPL10588,MBA_1871,E111 P16 ctrl TGFinhibitor,,

iPSC,BIOTIME,GPL10588,MBA_1872,"E111 P16 matrigel Fast media with SB431542 10uM, ROCK inh 10uM, EGF 100ng/ml 14 days TGFinhibitor",,

iPSC,BIOTIME,GPL10588,MBA_1873,"E111 P16 matrigel Fast media with SB431542 10uM, ROCK inh 10uM, for 14 days TGFinhibitor",,

iPSC,BIOTIME,GPL10588,MBA_1874,E111 P16 matrigel Fast media with SB431542 10uM 14 days TGFinhibitor,,

iPSC,BIOTIME,GPL10588,MBA_1875,"E111 P16 matrigel Fast media with SB431542 10uM, ROCK inh 10uM, EGF 100ng/ml 14 days with fast with growth to confl",,

iPSC,BIOTIME,GPL10588,MBA_1876,"E111 P16 matrigel Fast media with SB431542 10uM, ROCK inh 10uM 14 days with fast and with growth to confluence",,

iPSC,BIOTIME,GPL10588,MBA_1877,E68 P15 ctrl TGF inhibitor,,

iPSC,BIOTIME,GPL10588,MBA_1878,"E68 P15 matrigel Fast media with SB431542 10uM, ROCK inh 10uM, EGF 100ng/ml 14 days TGFinhibitor",,

iPSC,BIOTIME,GPL10588,MBA_1879,"E68 P15 matrigel Fast media with SB431542 10uM, ROCK inh 14 days TGFinhibitor",,

iPSC,BIOTIME,GPL10588,MBA_1880,"E68 P15 matrigel Fast media with SB431542 10uM, 14 days TGFinhibitor",,

iPSC,BIOTIME,GPL10588,MBA_1881,"E68 P15 matrigel Fast media with SB431542 10uM, ROCK inh 10uM, EGF 100ng/ml 14 days and in growth to confl",,

iPSC,BIOTIME,GPL10588,MBA_1882,"E68 P15 matrigel Fast media with SB431542 10uM, ROCK inh 10uM 14 days and in growth to confl",,

iPSC,BIOTIME,GPL10588,MBA_1883,E44 P17 ctrl TGFinhibitor,,

iPSC,BIOTIME,GPL10588,MBA_1884,"E44 P17 matrigel Fast media with SB431542 10uM, ROCK inh 10uM, EGF 100ng/ml 14 days TGFinhibitor",,

iPSC,BIOTIME,GPL10588,MBA_1885,"E44 P17 matrigel Fast media with SB431542 10uM, ROCK inh 10uM 14 days TGFinhibitor",,

iPSC,BIOTIME,GPL10588,MBA_1886,E44 P17 matrigel Fast media with SB431542 10uM 14 days TGFinhibitor,,

iPSC,BIOTIME,GPL10588,MBA_1887,"E44 P17 matrigel Fast media with SB431542 10uM, ROCK inh 10uM, EGF 100ng/ml 14 days and in growth to confl ",,

iPSC,BIOTIME,GPL10588,MBA_1888,"E44 P17 matrigel Fast media with SB431542 10uM, ROCK inh 10uM 14 days and in growth to confl ",,

iPSC,BIOTIME,GPL10588,MBA_1889,B16 P20 ctrl TGFinhibitor,,

iPSC,BIOTIME,GPL10588,MBA_1890,"B16 P20 matrigel Fast media with SB431542 10uM, ROCK inh 10uM, EGF 100ng/ml 14 days TGFinhibitor",,

iPSC,BIOTIME,GPL10588,MBA_1891,"B16 P20 matrigel Fast media with SB431542 10uM, ROCK inh 10uM 14 days TGFinhibitor",,

iPSC,BIOTIME,GPL10588,MBA_1892,B16 P20 matrigel Fast media with SB431542 10uM 14 days TGFinhibitor,,

iPSC,BIOTIME,GPL10588,MBA_1893,"B16 P20 matrigel TGFinhibitor study with SB431542 10uM, ROCK inh 10uM, EGF 100ng/ml 14 days with fast in growth to confl",,

iPSC,BIOTIME,GPL10588,MBA_1894,"B16 P20 matrigel TGFinhibitor study with SB431542 10uM, ROCK inh 10uM 14 days with fast in growth to confl",,

AC,BIOTIME,GPL10588,MBA_1898,HS1 Testicular 7002 P6 not fasted,,

iPSC,BIOTIME,GPL10588,MBA_1901,"F15 P17 matrigel Fast media with SB431542 10uM, ROCK inh 10uM, EGF 100ng/ml 14 days TGFinhibitor",,

iPSC,BIOTIME,GPL10588,MBA_1902,"F15 P17 matrigel Fast media with SB431542 10uM, ROCK inh 10uM 14 days TGFinhibitor",,

iPSC,BIOTIME,GPL10588,MBA_1903,"F15 P17 matrigel Fast media with SB431542 10uM, ROCK inh 14 days TGFinhibitor",,

iPSC,BIOTIME,GPL10588,MBA_1904,"F15 P17 matrigel Fast media with SB431542 10uM, ROCK inh 10uM, EGF 100ng/ml 14 days with fast and with growth to confluence",,

iPSC,BIOTIME,GPL10588,MBA_1905,"F15 P17 matrigel Fast media with SB431542 10uM, ROCK inh 10uM 14 days with fast and with growth to confluence",,

AC,BIOTIME,GPL10588,MBA_1906,Xgene FB P13 ctrl,,

AC,BIOTIME,GPL10588,MBA_1907,"Xgene P13 matrigel Fast media with SB431542 10uM, ROCK inh 10uM, EGF 100ng/ml 14 days ",,

AC,BIOTIME,GPL10588,MBA_1908,"Xgene P13 matrigel Fast media with SB431542 10uM, ROCK inh 10uM 14 days",,

AC,BIOTIME,GPL10588,MBA_1909,Xgene P13 matrigel Fast media with SB431542 14 days,,

AC,BIOTIME,GPL10588,MBA_1910,"Xgene Fibroblast P13 matrigel Fast media with SB431542 10uM, ROCK inh 10uM, EGF 100ng/ml 14 days with fast and with growth to confluence",,

AC,BIOTIME,GPL10588,MBA_1911,"Xgene Fibroblast P13 matrigel Fast media with SB431542 10uM, ROCK inh 10uM, EGF 100ng/ml 14 days with fast and with growth to confluence",,

iPSC,BIOTIME,GPL10588,MBA_1912,SM30 P14 ctrl well 1 NS,,

iPSC,BIOTIME,GPL10588,MBA_1913,SM30 P14 D14 ctrl,,

iPSC,BIOTIME,GPL10588,MBA_1914,SM30 P14 D14 SB431542 10uM,,

iPSC,BIOTIME,GPL10588,MBA_1915,SM30 P14 D28 ctrl,,

iPSC,BIOTIME,GPL10588,MBA_1916,SM30 P14 D28 SB431542 10uM,,

iPSC,BIOTIME,GPL10588,MBA_1917,Z11 P13 ctrl well 1 NS,,

iPSC,BIOTIME,GPL10588,MBA_1918,Z11 P13 D14 ctrl,,

iPSC,BIOTIME,GPL10588,MBA_1919,Z11 P13 D14 SB431542 10uM (in fast medium as ctrl),,

iPSC,BIOTIME,GPL10588,MBA_1920,Z11 P13 D28 ctrl,,

iPSC,BIOTIME,GPL10588,MBA_1921,Z11 P13 D28 SB431542 10uM,,

iPSC,BIOTIME,GPL10588,MBA_1922,EN13 P17 ctrl well 1 NS,,

iPSC,BIOTIME,GPL10588,MBA_1923,EN13 P17 D14 ctrl,,

iPSC,BIOTIME,GPL10588,MBA_1924,EN13 P17 D14 SB431542 10uM,,

iPSC,BIOTIME,GPL10588,MBA_1925,EN13 P17 D28 ctrl,,

iPSC,BIOTIME,GPL10588,MBA_1926,EN13 P17 D28 SB431542 10uM,,

iPSC,BIOTIME,GPL10588,MBA_1927,E164 P19 ctrl well 1 NS,,

iPSC,BIOTIME,GPL10588,MBA_1928,E164 P19 D14 ctrl,,

iPSC,BIOTIME,GPL10588,MBA_1929,E164 P19 D14 SB431542 10uM,,

iPSC,BIOTIME,GPL10588,MBA_1930,E164 P19 D28 ctrl,,

iPSC,BIOTIME,GPL10588,MBA_1931,E164 P19 D28 SB431542 10uM,,

iPSC,BIOTIME,GPL10588,MBA_1932,E111 P19 ctrl well 1 NS,,

iPSC,BIOTIME,GPL10588,MBA_1933,E111 P19 D14 ctrl,,

iPSC,BIOTIME,GPL10588,MBA_1934,E111 P19 D14 SB431542 10uM,,

iPSC,BIOTIME,GPL10588,MBA_1935,E111 P19 D28 ctrl,,

iPSC,BIOTIME,GPL10588,MBA_1936,E111 P19 D28 SB431542 10uM,,

iPSC,BIOTIME,GPL10588,MBA_1937,E68 P16 ctrl well 1 NS,,

iPSC,BIOTIME,GPL10588,MBA_1938,E68 P16 D14 ctrl,,

iPSC,BIOTIME,GPL10588,MBA_1939,E68 P16 D14 SB431542 10uM,,

iPSC,BIOTIME,GPL10588,MBA_1940,E68 P16 D28 ctrl,,

iPSC,BIOTIME,GPL10588,MBA_1941,E68 P16 D28 SB431542 10uM,,

iPSC,BIOTIME,GPL10588,MBA_1942,W10 P14 ctrl well 1 NS Epithelial expt 5 day fast,,

iPSC,BIOTIME,GPL10588,MBA_1943,W10 P14 D14 cont,,

iPSC,BIOTIME,GPL10588,MBA_1944,W10 P14 D14 SB431542 10uM,,

iPSC,BIOTIME,GPL10588,MBA_1945,W10 P14 D28 ctrl TGF inhib,,

iPSC,BIOTIME,GPL10588,MBA_1946,W10 P14 D28 SB431542 10uM,,

iPSC,BIOTIME,GPL10588,MBA_1947,CMO2 P23 ctrl well 1 NS Epithelial expt 5 day fast,,

iPSC,BIOTIME,GPL10588,MBA_1948,CMO2 P23 D14 ctrl,,

iPSC,BIOTIME,GPL10588,MBA_1949,CMO2 P23 D14 SB431542,,

iPSC,BIOTIME,GPL10588,MBA_1950,CMO2 P23 D28 ctrl TGF inhib,,

iPSC,BIOTIME,GPL10588,MBA_1951,CMO2 P23 D28 SB431542 10uM,,

iPSC,BIOTIME,GPL10588,MBA_1953,4D20.8 P19 D14 Hystem PDGF-BB 10ng/ml +TGFb3 10ng/ml,,

iPSC,BIOTIME,GPL10588,MBA_1954,4D20.8 P19 D14 Hystem PDGF-BB 10ng/ml,,

iPSC,BIOTIME,GPL10588,MBA_1955,4D20.8 P19 D14 Hystem TGFb3 10ng/ml,,

iPSC,BIOTIME,GPL10588,MBA_1956,4D20.8 P19 D14 Hystem TGFb3 10ng/ml + IGF-1 100ng/ml,,

iPSC,BIOTIME,GPL10588,MBA_1957,4D20.8 P19 D14 Hystem IGF-1 100ng/ml no TGFb3,,

iPSC,BIOTIME,GPL10588,MBA_1958,4D20.8 P19 D14 Hystem TGFb3 10ng/ml ,,

iPSC,BIOTIME,GPL10588,MBA_1959,4D20.8 P19 D14 Hystem BMP6 30ng/ml + TGFb3 10ng/m,,

iPSC,BIOTIME,GPL10588,MBA_1960,4D20.8 P19 D14 Hystem BMP6 30ng/ml no TGF,,

iPSC,BIOTIME,GPL10588,MBA_1961,4D20.8 P19 D14 Hystem TGF 10ng/ml,,

iPSC,BIOTIME,GPL10588,MBA_1963,4D20.8 P18 D14 Hystem GDF5 100ng/ml + TGFb3 10ng/ml,,

iPSC,BIOTIME,GPL10588,MBA_1964,4D20.8 P18 D14 Hystem GDF5 100ng/ml,,

iPSC,BIOTIME,GPL10588,MBA_1965,4D20.8 P18 D14 Hystem TGFb3 10ng/ml,,

iPSC,BIOTIME,GPL10588,MBA_1966,4D20.8 P22 Hystem D14 BMP4 10ng/ml + BMP7 100ng/ml no TGFb3,,

iPSC,BIOTIME,GPL10588,MBA_1967,4D20.8 P22 Hystem D14 BMP2 50ng/ml + BMP7 100ng/ml no TGFb3,,

iPSC,BIOTIME,GPL10588,MBA_1968,4D20.8 P22 Hystem D14 BMP4 10ng/ml + BMP7 100ng/ml + TGFb3 10ng/ml,,

iPSC,BIOTIME,GPL10588,MBA_1969,4D20.8 P22 Hystem D14 BMP2 50ng/ml + BMP7 100ng/ml + TGFb3 10ng/ml,,

iPSC,BIOTIME,GPL10588,MBA_1970,4D20.8 P22 Hystem D14 BMP2 50ng/ml + BMP4 10ng/ml no TGFb3,,

iPSC,BIOTIME,GPL10588,MBA_1971,4D20.8 P22 Hystem D14 BMP2 50ng/ml + BMP4 10ng/ml + TGFb3 10ng/ml,,

iPSC,BIOTIME,GPL10588,MBA_1972,4D20.8 P23 D14 Pellet BMP2 50ng/ml + TGFb3 10ng/ml,,

iPSC,BIOTIME,GPL10588,MBA_1973,4D20.8 P23 D14 Pellet BMP4 10ng/ml + TGFb3 10ng/ml,,

iPSC,BIOTIME,GPL10588,MBA_1974,4D20.8 P23 D14 Pellet BMP6 30ng/ml + TGFb3 10ng/ml,,

iPSC,BIOTIME,GPL10588,MBA_1975,4D20.8 P23 D14 Pellet BMP7 100ng/ml + TGFb3 10ng/ml,,

iPSC,BIOTIME,GPL10588,MBA_1976,4D20.8 P23 D14 Pellet GDF5 100ng/ml + TGFb3 10ng/ml,,

AC,BIOTIME,GPL10588,MBA_2156,"HS tRNA (Human Synoviocyte Cell total RNA), 10 _g",,

iPSC,BIOTIME,GPL10588,MBA_2386,E15 P19 D14 Pellet BMP2 50ng/ml + TGFb3 10ng/ml,,

iPSC,BIOTIME,GPL10588,MBA_2387,E15 P19 D14 Pellet BMP4 10ng/ml + TGFb3 10ng/ml,,

iPSC,BIOTIME,GPL10588,MBA_2388,E15 P19 D14 Pellet BMP6 30ng/ml + TGFb3 10ng/ml,,

iPSC,BIOTIME,GPL10588,MBA_2389,E15 P19 D14 Pellet BMP7 100ng/ml + TGFb3 10ng/ml,,

iPSC,BIOTIME,GPL10588,MBA_2390,E15 P19 D14 Pellet GDF5 100ng/ml + TGFb3 10ng/ml,,

iPSC,BIOTIME,GPL10588,MBA_2392,7PEND24 P19 D14 pellet BMP2 50ng/ml +TGFb3 10ng/ml,,

iPSC,BIOTIME,GPL10588,MBA_2393,7PEND24 P19 D14 pellet BMP4 10ng/ml +TGFb3 10ng/ml,,

iPSC,BIOTIME,GPL10588,MBA_2394,7PEND24 P19 D14 pellet BMP6 30ng/ml +TGFb3 10ng/ml,,

iPSC,BIOTIME,GPL10588,MBA_2395,7PEND24 P19 D14 pellet BMP7 100ng/ml +TGFb3 10ng/ml,,

iPSC,BIOTIME,GPL10588,MBA_2396,7PEND24 P19 D14 pellet GDF5 100ng/ml +TGFb3 10ng/ml,,

iPSC,BIOTIME,GPL10588,MBA_2397,CM50.5 P12 matrigel D14 with SB431542,,

iPSC,BIOTIME,GPL10588,MBA_2398,CM50.5 P12 matrigel D28 with SB431542,,

iPSC,BIOTIME,GPL10588,MBA_2399,SK11 P13 matrigel D14 with SB431542 study,,

iPSC,BIOTIME,GPL10588,MBA_2400,E163 P15 matrigel D14 with SB431542,,

iPSC,BIOTIME,GPL10588,MBA_2401,RASKEL8 P14 D14 matrigel with SB431542,,

iPSC,BIOTIME,GPL10588,MBA_2402,4D20.8 P20 matrtrigel D28 with SB431542,,

iPSC,BIOTIME,GPL10588,MBA_2403,C4ELSR5.1 P13 matrigel D14 with SB431542,,

iPSC,BIOTIME,GPL10588,MBA_2404,SK11 P13 matrigel D28 with SB431542,,

iPSC,BIOTIME,GPL10588,MBA_2405,E163 P15 matrigel D28 with SB431542,,

iPSC,BIOTIME,GPL10588,MBA_2406,RASKEL-8 P14 matrigel D28 with SB431542,,

iPSC,BIOTIME,GPL10588,MBA_2407,C4ELSR5.1 P13 matrigel D28 with SB431542,,

iPSC,BIOTIME,GPL10588,MBA_3678,CDD4-SKEL-22 P8 D14 MM,,

iPSC,BIOTIME,GPL10588,MBA_3679,CDD4-MV2-24 P8 D14 MM,,

iPSC,BIOTIME,GPL10588,MBA_3680,CDD4-MV2-18 P8 D14 MM,,

iPSC,BIOTIME,GPL10588,MBA_3681,CDD4-MV2-19 P8 D14MM,,

iPSC,BIOTIME,GPL10588,MBA_3683,CDD4-DM20-20 P8 D14MM,,

iPSC,BIOTIME,GPL10588,MBA_3685,CDD4-DM20-17 D14 MM,,

iPSC,BIOTIME,GPL10588,MBA_3687,CDD4-MV2-7 P9 D14 MM,,

iPSC,BIOTIME,GPL10588,MBA_3688,CDD4-MV2-23 P9 D14 MM,,

iPSC,BIOTIME,GPL10588,MBA_3689,CDD4-MV2-14 P9 D14 MM,,

iPSC,BIOTIME,GPL10588,MBA_3690,CDD4-MV2-6 P10 D14MM,,

iPSC,BIOTIME,GPL10588,MBA_3691,CDD4-MV2-13 P10 D14MM,,

iPSC,BIOTIME,GPL10588,MBA_3692,CDD4-MV2-16 P10 D14MM,,

AC,BIOTIME,GPL10588,MBA_3694,DPSC P8 D14 Pellet TGFb3 10ng/ml no BMP7,,

AC,BIOTIME,GPL10588,MBA_3695,DPSC P8 D14 Pellet TGFb3 10ng/ml + BMP7 100ng/ml,,

AC,BIOTIME,GPL10588,MBA_3696,DPSC P8 D14 MM TGFb3 10ng/ml + BMP7 100ng/ml,,

AC,BIOTIME,GPL10588,MBA_3697,DPSC P8 D14 MM TGFb3 10ng/ml no BMP7,,

iPSC,BIOTIME,GPL10588,MBA_3698,4D20.8 P19 D14 Chondro Hystem Sponge GDF5 100ng/ml +TGFb3 10ng/ml,,

iPSC,BIOTIME,GPL10588,MBA_3699,4D20.8 P19 D14 Chondro Hystem Sponge BMP7 100ng/ml + TGFb3 10ng/ml,,

AC,BIOTIME,GPL10588,MBA_3700,NHAC P6 Alginate D25 Lonza Differentiation Instructions and Cells,,

iPSC,BIOTIME,GPL10588,MBA_3701,4D20.8 P15 D21 Hystem 1.2%w/v TGFb3 10ng/ml +BMP7 100ng/ml,,

iPSC,BIOTIME,GPL10588,MBA_3702,4D20.8 P15 D21 Hystem no gelatin 1.2%w/v TGFb3 10ng/ml +BMP7 100ng/ml,,

iPSC,BIOTIME,GPL10588,MBA_3703,4D20.8 P15 D21 Hystem 2.4%w/v TGFb3 10ng/ml +BMP7 100ng/ml,,

iPSC,BIOTIME,GPL10588,MBA_3704,4D20.8 P15 D21 Hystem no gelatin 2.4%w/v TGFb3 10ng/ml +BMP7 100ng/ml,,

iPSC,BIOTIME,GPL10588,MBA_3705,RP1-SKEL-8 P9 2% horse serum Day 9 matrigel 60% density myoblast diff,,

iPSC,BIOTIME,GPL10588,MBA_3706,RP1-SKEL-8 P9 2% horse serum Day 9 matrigel 100% density myoblast diff,,

iPSC,BIOTIME,GPL10588,MBA_3707,RP1-SKEL-8 P9 2% horse serum Day 9 gelatin 60% density myoblast diff,,

iPSC,BIOTIME,GPL10588,MBA_3708,RP1-SKEL-8 P9 2% horse serum Day 9 gelatin 100% density myoblast diff,,

iPSC,BIOTIME,GPL10588,MBA_3709,RP1-SKEL-8 P14 D8 Myo 2 with Dex and Ins 90%,,

iPSC,BIOTIME,GPL10588,MBA_3710,RP1-SKEL-8 P14 D8 Myo 2 with Dex and Ins 80%,,

iPSC,BIOTIME,GPL10588,MBA_3711,RP1-SKEL-8 P14 D8 control SKEL +SB,,

iPSC,BIOTIME,GPL10588,MBA_3712,RP1-MV2-6 P13 on matrigel ,,

iPSC,BIOTIME,GPL10588,MBA_3713,RP1-MV2-6 P13 on plastic,,

iPSC,BIOTIME,GPL10588,MBA_3714,RP1-SKEL-5 P9 in MV2+SB431542 for P2 then prepped in 6 well,,

iPSC,BIOTIME,GPL10588,MBA_3715,RP1- SKEL-5 P15 for P8 MV2 media with SB431542,,

iPSC,BIOTIME,GPL10588,MBA_3736,SM30 P14 media change MEL Fast,,

iPSC,BIOTIME,GPL10588,MBA_3737,E111 P19 Media Change Mel Fast,,

iPSC,BIOTIME,GPL10588,MBA_3738,E111 P9 Medium Change Epilife Fast,,

iPSC,BIOTIME,GPL10588,MBA_3739,W10 P17 Medium Change MEL fast,,

iPSC,BIOTIME,GPL10588,MBA_3741,J16 P13 D17 Adipo alpha MEM,,

iPSC,BIOTIME,GPL10588,MBA_3742,J16 P13 D17 Adipo epilife,,

AC,BIOTIME,GPL10588,MBA_3762,ENDMT-48HAMH 10ng/ml,,

iPSC,BIOTIME,GPL10588,MBA_3763,SM30 P14 media change MEL Fast,,

AC,BIOTIME,GPL10588,MBA_3818,BH2 P11,,

AC,BIOTIME,GPL10588,MBA_3819,Xgene P20 lifespan study,,

AC,BIOTIME,GPL10588,MBA_3820,SR1 P19 lifespan study,,

iPSC,BIOTIME,GPL10588,MBA_3821,T42 P17 Post-production testing,,

iPSC,BIOTIME,GPL10588,MBA_3825,4D20.8 P15 D42 Hystem BMP7 100ng/ml + TGFb3 10ng/ml,,

iPSC,BIOTIME,GPL10588,MBA_3826,4D20.8 P15 D42Hystem no Gelatin BMP7 100ng/ml + TGFb3 10ng/ml,,

iPSC,BIOTIME,GPL10588,MBA_3827,4D20.8 P15 D42 Hystem 2X BMP7 100ng/ml + TGFb3 10ng/ml,,

iPSC,BIOTIME,GPL10588,MBA_3828,4D20.8 P15 D42 Hystem 2X no Gelatin BMP7 100ng/ml + TGFb3 10ng/ml,,

iPSC,BIOTIME,GPL10588,MBA_3830,4D20.8 P17 D14 pellet GDF5 100ng/ml,,

iPSC,BIOTIME,GPL10588,MBA_3831,4D20.8 P17 D14 pellet GDF5 100ng/ml + TGFb3 10ng/ml,,

iPSC,BIOTIME,GPL10588,MBA_3832,4D20.8 P17 D14 pellet TGFb3 10ng/ml,,

iPSC,BIOTIME,GPL10588,MBA_3833,4D20.8 P17 D14 pellet BMP7 100ng/ml + TGFb3 10ng/ml,,

iPSC,BIOTIME,GPL10588,MBA_3834,4D20.8 P16 D21 Hystem 2x w/o gelatin BMP7 100ng/ml + TGFb3 10ng/ml,,

iPSC,BIOTIME,GPL10588,MBA_3835,4D20.8 P16 D21 Hystem 2x BMP7 100ng/ml + TGFb3 10ng/ml,,

iPSC,BIOTIME,GPL10588,MBA_3836,4D20.8 P16 D21 Hystem 2x w/o gelatin GDF5 100ng/ml + TGFb3 10ng/ml,,

iPSC,BIOTIME,GPL10588,MBA_3837,4D20.8 P16 D21 Hystem 2x GDF5 100ng/ml + TGFb3 10ng/ml,,

iPSC,BIOTIME,GPL10588,MBA_3838,CM02 P18 P7 with MEL medium change,,

iPSC,BIOTIME,GPL10588,MBA_3839,W10 P17 Medium Change CnT 07,,

iPSC,BIOTIME,GPL10588,MBA_3840,SM30 P16 P4 CnT 07 (BM1) medium change,,

iPSC,BIOTIME,GPL10588,MBA_3841,E164 P19 Medium Change EpiLife,,

iPSC,BIOTIME,GPL10588,MBA_3842,E68 P18 P7 Mel media change,,

iPSC,BIOTIME,GPL10588,MBA_3843,E68 P18 P7 EpiLife media change,,

iPSC,BIOTIME,GPL10588,MBA_3844,CMO2 P18 P7 EpiLife media change,,

iPSC,BIOTIME,GPL10588,MBA_3862,CMO2 P19 D14 Hystem BMP7 100ng/ml,,

iPSC,BIOTIME,GPL10588,MBA_3863,CMO2 P19 D14 Hystem TGFb3 10ng/ml,,

iPSC,BIOTIME,GPL10588,MBA_3864,CMO2 P19 D14 Hystem BMP7 100ng/ml + TGFb3 10ng/ml,,

iPSC,BIOTIME,GPL10588,MBA_3865,CMO2 P19 D14 Hystem BMP2 50ng/ml + TGFb3 10ng/ml,,

iPSC,BIOTIME,GPL10588,MBA_3866,CMO2 P19 D14 Hystem BMP4 10ng/ml,,

iPSC,BIOTIME,GPL10588,MBA_3867,CMO2 P19 D14 Hystem GDF5 100ng/ml,,

iPSC,BIOTIME,GPL10588,MBA_3868,CMO2 P19 D14 Hystem GDF5 100ng/ml +TGFb3 10ng/ml,,

iPSC,BIOTIME,GPL10588,MBA_3869,CMO2 P19 D14 Hystem BMP6 30ng/ml,,

iPSC,BIOTIME,GPL10588,MBA_3870,CMO2 P19 D14 Hystem BMP6 30ng/ml +TGFb3 10ng/ml,,

iPSC,BIOTIME,GPL10588,MBA_3871,E68 P18 Media change CnT-07 +SB431542 P7,,

iPSC,BIOTIME,GPL10588,MBA_3872,SM30 P16 P4 CnT 07 (BM1) medium change,,

iPSC,BIOTIME,GPL10588,MBA_3873,CMO2 P18 Medium Change CnT-07 P7,,

iPSC,BIOTIME,GPL10588,MBA_3894,RP1-MV2-16 P19 lifespan,,

iPSC,BIOTIME,GPL10588,MBA_3896,7PEND24 P22 D14 Hystem BMP7 100ng/ml,,

iPSC,BIOTIME,GPL10588,MBA_3897,7PEND24 P22 D14 Hystem TGFb3 10ng/ml,,

iPSC,BIOTIME,GPL10588,MBA_3898,7PEND24 P22 D14 Hystem BMP7 100ng/ml + TGFb3 10ng/ml,,

iPSC,BIOTIME,GPL10588,MBA_3899,7PEND24 P22 D14 Hystem BMP2 50ng/ml + TGFb3 10ng/ml,,

iPSC,BIOTIME,GPL10588,MBA_3900,7PEND24 P22 D14 Hystem BMP4 10ng/ml,,

iPSC,BIOTIME,GPL10588,MBA_3902,MEL2 P26 D14 Hystem BMP7 100ng/ml,,

iPSC,BIOTIME,GPL10588,MBA_3903,MEL2 P26 D14 Hystem TGFb3 10ng/ml,,

iPSC,BIOTIME,GPL10588,MBA_3904,MEL2 P26 D14 Hystem BMP7 100ng/ml + TGFb3 10ng/ml,,

iPSC,BIOTIME,GPL10588,MBA_3905,MEL2 P26 D14 Hystem BMP2 50ng/ml + TGFb3 10ng/ml,,

iPSC,BIOTIME,GPL10588,MBA_3906,MEL2 P26 D14 Hystem BMP4 10ng/ml,,

iPSC,BIOTIME,GPL10588,MBA_3908,4D20.8 P16 D44 Hystem 2x G- BMP7 100ng/ml + TGFb3 10ng/ml,,

iPSC,BIOTIME,GPL10588,MBA_3909,4D20.8 P16 D44 Hystem 2x G- BMP7 100ng/ml + TGFb3 10ng/ml,,

iPSC,BIOTIME,GPL10588,MBA_3910,4D20.8 P16 D44 Hystem 2x G- BMP7 100ng/ml + TGFb3 10ng/ml,,

iPSC,BIOTIME,GPL10588,MBA_3911,4D20.8 P16 D44 Hystem 2x BMP7 100ng/ml + TGFb3 10ng/ml,,

iPSC,BIOTIME,GPL10588,MBA_3912,4D20.8 P16 D44 Hystem 2x BMP7 100ng/ml + TGFb3 10ng/ml,,

iPSC,BIOTIME,GPL10588,MBA_3913,4D20.8 P16 D44 Hystem 2x BMP7 100ng/ml + TGFb3 10ng/ml,,

iPSC,BIOTIME,GPL10588,MBA_3914,4D20.8 P16 D44 Hystem 2x G- GDF5 100ng/ml + TGFb3 10ng/ml,,

iPSC,BIOTIME,GPL10588,MBA_3915,4D20.8 P16 D44 Hystem 2x G- GDF5 100ng/ml + TGFb3 10ng/ml,,

iPSC,BIOTIME,GPL10588,MBA_3916,4D20.8 P16 D44 Hystem 2x G- GDF5 100ng/ml + TGFb3 10ng/ml,,

iPSC,BIOTIME,GPL10588,MBA_3917,4D20.8 P16 D44 Hystem 2x GDF5 100ng/ml + TGFb3 10ng/ml,,

iPSC,BIOTIME,GPL10588,MBA_3918,4D20.8 P16 D44 Hystem 2x GDF5 100ng/ml + TGFb3 10ng/ml,,

iPSC,BIOTIME,GPL10588,MBA_3919,4D20.8 P16 D44 Hystem 2x GDF5 100ng/ml + TGFb3 10ng/ml,,

iPSC,BIOTIME,GPL10588,MBA_3921,SM30 P15 D14 Hystem BMP7 100ng/ml,,

iPSC,BIOTIME,GPL10588,MBA_3922,SM30 P15 D14 Hystem BMP2 50ng/ml + TGFb3 10ng/ml,,

iPSC,BIOTIME,GPL10588,MBA_3923,SM30 P15 D14 Hystem BMP4 10ng/ml ,,

AC,BIOTIME,GPL10588,MBA_3925,BH-2 P4 lifespan study,,

AC,BIOTIME,GPL10588,MBA_3926,SR-1 P4 lifespan study,,

AC,BIOTIME,GPL10588,MBA_3927,Xgene P7 lifespan study,,

iPSC,BIOTIME,GPL10588,MBA_3969,MEL2 P28 D14 Hystem GDF5 100ng/ml,,

iPSC,BIOTIME,GPL10588,MBA_3970,MEL2 P28 D14 Hystem GDF5 100ng/ml + TGFb3 10ng/ml,,

iPSC,BIOTIME,GPL10588,MBA_3971,MEL2 P28 D14 Hystem BMP6 30ng/ml ,,

iPSC,BIOTIME,GPL10588,MBA_3972,MEL2 P28 D14 Hystem BMP6 30ng/ml + TGFb3 10ng/ml,,

iPSC,BIOTIME,GPL10588,MBA_3973,MEL2 P28 D14 Hystem BMP2 50ng/ml,,

iPSC,BIOTIME,GPL10588,MBA_3975,E111 P16 D14 Hystem GDF5 100ng/ml + TGFb3 10ng/ml,,

iPSC,BIOTIME,GPL10588,MBA_3977,SK11 P17 D14 Hystem BMP7 100ng/ml,,

iPSC,BIOTIME,GPL10588,MBA_3978,SK11 P17 D14 Hystem TGFb3 10ng/ml,,

iPSC,BIOTIME,GPL10588,MBA_3979,SK11 P17 D14 Hystem BMP7 100ng/ml + TGFb3 10ng/ml,,

iPSC,BIOTIME,GPL10588,MBA_3980,SK11 P17 D14 Hystem BMP2 50ng/ml + TGFb3 10ng/ml,,

iPSC,BIOTIME,GPL10588,MBA_3981,SK11 P17 D14 Hystem BMP4 10ng/ml,,

iPSC,BIOTIME,GPL10588,MBA_3983,SK11 P18 D14 Hystem TGFb3 10ng/ml,,

iPSC,BIOTIME,GPL10588,MBA_3984,SK11 P18 D14 Hystem RA 1uM,,

iPSC,BIOTIME,GPL10588,MBA_3985,SK11 P18 D14 Hystem BMP7 100ng/ml,,

iPSC,BIOTIME,GPL10588,MBA_3986,SK11 P18 D14 Hystem BMP7 100ng/ml + TGFb3 10ng/ml,,

iPSC,BIOTIME,GPL10588,MBA_3987,SK11 P18 D14 Hystem GDF5 100ng/ml,,

iPSC,BIOTIME,GPL10588,MBA_3988,SK11 P18 D14 Hystem GDF5 100ng/ml + TGFb3 10ng/ml,,

iPSC,BIOTIME,GPL10588,MBA_3989,SK11 P18 D14 Hystem BMP4 10ng/ml,,

iPSC,BIOTIME,GPL10588,MBA_3991,SK11 P18 D14 Hystem BMP6 30ng/ml ,,

iPSC,BIOTIME,GPL10588,MBA_3992,SK11 P18 D14 Hystem BMP6 30ng/ml + TGFb3 10ng/ml,,

iPSC,BIOTIME,GPL10588,MBA_3993,SK11 P18 D14 Hystem BMP2 50ng/ml,,

iPSC,BIOTIME,GPL10588,MBA_3994,SK11 P18 D14 Hystem BMP2 50ng/ml + TGFb3 10ng/ml,,

iPSC,BIOTIME,GPL10588,MBA_3995,SM30 P18 ctrl,,

iPSC,BIOTIME,GPL10588,MBA_3996,SM30 P17 D14 Hystem BMP6 30ng/ml + TGFb3 10ng/ml,,

iPSC,BIOTIME,GPL10588,MBA_3997,SM30 P17 D14 Hystem GDF5 100ng/ml,,

iPSC,BIOTIME,GPL10588,MBA_3998,SM30 P17 D14 Hystem GDF5 100ng/ml + TGFb3 10ng/ml,,

iPSC,BIOTIME,GPL10588,MBA_3999,SM30 P17 D14 Hystem BMP2 50ng/ml,,

iPSC,BIOTIME,GPL10588,MBA_4000,SM30 P17 D14 Hystem BMP6 30ng/ml,,

iPSC,BIOTIME,GPL10588,MBA_4001,SM30 P18 D14 Hystem TGFb3 10ng/ml,,

iPSC,BIOTIME,GPL10588,MBA_4002,SM30 P18 D14 Hystem RA 1uM,,

iPSC,BIOTIME,GPL10588,MBA_4003,SM30 P18 D14 Hystem BMP7 100ng/ml,,

iPSC,BIOTIME,GPL10588,MBA_4004,SM30 P18 D14 Hystem BMP7 100ng/ml + TGFb3 10ng/ml,,

iPSC,BIOTIME,GPL10588,MBA_4005,SM30 P18 D14 Hystem GDF5 100ng/ml,,

iPSC,BIOTIME,GPL10588,MBA_4006,SM30 P18 D14 Hystem GDF5 100ng/ml + TGFb3 10ng/ml,,

iPSC,BIOTIME,GPL10588,MBA_4007,SM30 P18 D14 Hystem BMP4 10ng/ml,,

iPSC,BIOTIME,GPL10588,MBA_4009,SM30 P18 D14 Hystem BMP6 30ng/ml,,

iPSC,BIOTIME,GPL10588,MBA_4010,SM30 P18 D14 Hystem BMP6 30ng/ml + TGFb3 10ng/ml,,

iPSC,BIOTIME,GPL10588,MBA_4011,SM30 P18 D14 Hystem BMP2 50ng/ml,,

iPSC,BIOTIME,GPL10588,MBA_4012,SM30 P18 D14 Hystem BMP2 50ng/ml + TGFb3 10ng/ml,,

iPSC,BIOTIME,GPL10588,MBA_4014,EN7 P13 D14 Hystem TGFb3 10ng/ml,,

iPSC,BIOTIME,GPL10588,MBA_4015,EN7 P13 D14 Hystem RA 1uM,,

iPSC,BIOTIME,GPL10588,MBA_4016,EN7 P13 D14 Hystem BMP7 100ng/ml,,

iPSC,BIOTIME,GPL10588,MBA_4017,EN7 P13 D14 Hystem BMP7 100ng/ml + TGFb3 10ng/ml,,

iPSC,BIOTIME,GPL10588,MBA_4018,EN7 P13 D14 Hystem GDF5 100ng/ml,,

iPSC,BIOTIME,GPL10588,MBA_4019,EN7 P13 D14 Hystem GDF5 100ng/ml + TGFb3 10ng/ml,,

iPSC,BIOTIME,GPL10588,MBA_4020,EN7 P13 D14 Hystem BMP4 10ng/ml,,

iPSC,BIOTIME,GPL10588,MBA_4021,EN7 P13 D14 Hystem BMP4 10ng/ml + TGFb3 10ng/ml,,

iPSC,BIOTIME,GPL10588,MBA_4022,EN7 P13 D14 Hystem BMP6 30ng/ml,,

iPSC,BIOTIME,GPL10588,MBA_4023,EN7 P13 D14 Hystem BMP6 30ng/ml + TGFb3 10ng/ml,,

iPSC,BIOTIME,GPL10588,MBA_4024,EN7 P13 D14 Hystem BMP2 50ng/ml,,

iPSC,BIOTIME,GPL10588,MBA_4025,EN7 P13 D14 Hystem BMP2 50ng/ml + TGFb3 10ng/ml,,

iPSC,BIOTIME,GPL10588,MBA_4026,EN7 P13 D21 Hystem BMP7 100ng/ml + TGFb3 10ng/ml,,

iPSC,BIOTIME,GPL10588,MBA_4027,EN7 P13 D21 Hystem GDF5 100ng/ml + TGFb3 10ng/ml,,

iPSC,BIOTIME,GPL10588,MBA_4028,EN7 P13 D21 Hystem BMP4 10ng/m +TGFb3 10ng/mll,,

iPSC,BIOTIME,GPL10588,MBA_4029,7SMOO32 P17 D14 Hystem TGFb3 10ng/ml ,,

iPSC,BIOTIME,GPL10588,MBA_4030,7SMOO32 P17 D14 Hystem RA 1uM ,,

iPSC,BIOTIME,GPL10588,MBA_4031,7SMOO32 P17 D14 Hystem BMP7 100ng/ml ,,

iPSC,BIOTIME,GPL10588,MBA_4032,7SMOO32 P17 D14 Hystem BMP7 100ng/ml + TGFb3 10ng/ml ,,

iPSC,BIOTIME,GPL10588,MBA_4033,7SMOO32 P17 D14 Hystem GDF5 100ng/ml ,,

iPSC,BIOTIME,GPL10588,MBA_4034,7SMOO32 P17 D14 Hystem GDF5 100ng/ml + TGFb3 10ng/ml ,,

iPSC,BIOTIME,GPL10588,MBA_4035,7SMOO32 P17 D14 Hystem BMP4 10ng/ml ,,

iPSC,BIOTIME,GPL10588,MBA_4037,7SMOO32 P17 D14 Hystem BMP6 30ng/ml ,,

iPSC,BIOTIME,GPL10588,MBA_4038,7SMOO32 P17 D14 Hystem BMP6 30ng/ml +TGFb3 10ng/ml ,,

iPSC,BIOTIME,GPL10588,MBA_4039,7SMOO32 P17 D14 Hystem BMP2 50ng/ml ,,

iPSC,BIOTIME,GPL10588,MBA_4040,7SMOO32 P17 D14 Hystem BMP2 50ng/ml + TGFb3 10ng/ml ,,

iPSC,BIOTIME,GPL10588,MBA_4042,W8 P13 D14 Hystem TGFb3 10ng/ml ,,

iPSC,BIOTIME,GPL10588,MBA_4043,W8 P13 D14 Hystem RA 1uM,,

iPSC,BIOTIME,GPL10588,MBA_4044,W8 P13 D14 Hystem BMP7 100ng/ml,,

iPSC,BIOTIME,GPL10588,MBA_4045,W8 P13 D14 Hystem BMP7 100ng/ml + TGFb3 10ng/ml,,

iPSC,BIOTIME,GPL10588,MBA_4046,W8 P13 D14 Hystem GDF5 100ng/ml,,

iPSC,BIOTIME,GPL10588,MBA_4047,W8 P13 D14 Hystem GDF5 100ng/ml + TGFb3 10ng/ml,,

iPSC,BIOTIME,GPL10588,MBA_4048,W8 P13 D14 Hystem BMP4 10ng/ml,,

iPSC,BIOTIME,GPL10588,MBA_4049,W8 P13 D14 Hystem BMP4 10ng/ml + TGFb3 10ng/ml,,

iPSC,BIOTIME,GPL10588,MBA_4050,W8 P13 D14 Hystem BMP6 30ng/ml,,

iPSC,BIOTIME,GPL10588,MBA_4051,W8 P13 D14 Hystem BMP6 30ng/ml + TGFb3 10ng/ml,,

iPSC,BIOTIME,GPL10588,MBA_4052,W8 P13 D14 Hystem BMP2 50ng/ml,,

iPSC,BIOTIME,GPL10588,MBA_4053,W8 P13 D14 Hystem BMP2 50ng/ml + TGFb3 10ng/ml,,

iPSC,BIOTIME,GPL10588,MBA_4054,W8 P13 D14 Hystem BMP7 + TGFb3 10ng/ml agarose,,

iPSC,BIOTIME,GPL10588,MBA_4055,Z11 P17 D14 Hystem TGFb3 10ng/ml,,

iPSC,BIOTIME,GPL10588,MBA_4056,Z11 P17 D14 Hystem BMP7 100ng/ml,,

iPSC,BIOTIME,GPL10588,MBA_4057,Z11 P17 D14 Hystem BMP7 100ng/ml + TGFb3 10ng/ml,,

iPSC,BIOTIME,GPL10588,MBA_4058,Z11 P17 D14 Hystem GDF5 100ng/ml,,

iPSC,BIOTIME,GPL10588,MBA_4059,Z11 P17 D14 Hystem GDF5 100ng/ml + TGFb3 10ng/ml,,

iPSC,BIOTIME,GPL10588,MBA_4060,Z11 P17 D14 Hystem BMP4 10ng/ml + TGFb3 10ng/ml,,

iPSC,BIOTIME,GPL10588,MBA_4061,Z11 P17 D14 Hystem BMP6 30ng/ml + TGFb3 10ng/ml,,

iPSC,BIOTIME,GPL10588,MBA_4062,Z11 P17 D14 Hystem BMP2 50ng/ml + TGFb3 10ng/ml,,

iPSC,BIOTIME,GPL10588,MBA_4063,SM22 P14 D14 Hystem BMP4 10ng/ml + TGFb3 10ng/ml,,

iPSC,BIOTIME,GPL10588,MBA_4064,SM22 P14 D14 Hystem BMP2 50ng/ml,,

AC,BIOTIME,GPL10588,MBA_4065,U937 P8,,

iPSC,BIOTIME,GPL10588,MBA_4068,"W8 P14 D15 Hystem TGFb3 10ng/ml using PEGSSDA (not gelated well, loose cell clumps)",,

iPSC,BIOTIME,GPL10588,MBA_4069,"W8 P14 D15 Hystem BMP7 100ng/ml + TGFb3 10ng/ml using PEGSSDA (not gelated well, loose cell clumps)",,

iPSC,BIOTIME,GPL10588,MBA_4070,SK11 P14 ctrl ,,

iPSC,BIOTIME,GPL10588,MBA_4071,SK11 P14 D14 Hystem TGFb3 10ng/ml,,

iPSC,BIOTIME,GPL10588,MBA_4072,SK11 P14 D14 Hystem BMP7 100ng/ml + TGFb3 10ng/ml,,

iPSC,BIOTIME,GPL10588,MBA_4073,SK11 P14 D14 Hystem GDF5 + TGFb3 10ng/ml,,

iPSC,BIOTIME,GPL10588,MBA_4075,SK11 P14 D14 Hystem BMP6 30ng/ml + TGFb3 10ng/ml,,

iPSC,BIOTIME,GPL10588,MBA_4076,SK11 P14 D14 Hystem BMP2 50ng/ml + TGFb3 10ng/ml,,

iPSC,BIOTIME,GPL10588,MBA_4078,SK11 P14 D14 Hystem BMP7 100ng/ml + TGFb3 10ng/ml low density,,

iPSC,BIOTIME,GPL10588,MBA_4079,RP1-MV2-16 P8 Lifespan Walter,,

iPSC,BIOTIME,GPL10588,MBA_4080,RP1-MV2-16 P12 Lifespan Walter,,

iPSC,BIOTIME,GPL10588,MBA_4081,RP1-MV2-16 P14 Lifespan Walter,,

iPSC,BIOTIME,GPL10588,MBA_4082,RP1-MV2-16 P19 Lifespan Walter,,

iPSC,BIOTIME,GPL10588,MBA_4083,RP1-MV2-18 P10 Lifespan Walter,,

iPSC,BIOTIME,GPL10588,MBA_4084,RP1-MV2-18 P13 Lifespan Walter,,

iPSC,BIOTIME,GPL10588,MBA_4085,RP1-MV2-18 P15 Lifespan Walter,,

iPSC,BIOTIME,GPL10588,MBA_4106,4D20.8 P22 D45 Hystem 2x GDF5 100ng/ml + TGFb3 10ng/ml,,

iPSC,BIOTIME,GPL10588,MBA_4107,4D20.8 P22 D45 Hystem 2x GDF5 100ng/ml + TGFb3 10ng/ml,,

iPSC,BIOTIME,GPL10588,MBA_4108,4D20.8 P22 D45 Hystem 2x GDF5 100ng/ml + TGFb3 10ng/ml,,

iPSC,BIOTIME,GPL10588,MBA_4109,E44 P20 ctrl fast DE3 screen BMP SB,,

iPSC,BIOTIME,GPL10588,MBA_4110,E44 P20 D14 SB431542 10uM in MV2 supplemented with 10ng/ml bFGF and VEGFA 10ng/ml,,

iPSC,BIOTIME,GPL10588,MBA_4111,E44 P20 D14 MM RA 1uM (no dexamethasone),,

iPSC,BIOTIME,GPL10588,MBA_4112,E44 P20 D14 MM BMP4 10ng/ml,,

iPSC,BIOTIME,GPL10588,MBA_4114,E15 P18 D14 MM RA 1uM (no dexamethasone),,

iPSC,BIOTIME,GPL10588,MBA_4115,E15 P18 D14 MM BMP4 10ng/ml ,,

iPSC,BIOTIME,GPL10588,MBA_4116,E15 P18 D14 MM BMP2 50ng/ml +TGFb3 10ng/ml,,

iPSC,BIOTIME,GPL10588,MBA_4117,E15 P18 D14 SB431542 10nM in MV2 DE3 screen,,

iPSC,BIOTIME,GPL10588,MBA_4118,E68 P14 ctrl DE3 screen,,

iPSC,BIOTIME,GPL10588,MBA_4119,E68 P14 D14 MM BMP2 50ng/ml + TGFb3 10ng/ml,,

iPSC,BIOTIME,GPL10588,MBA_4120,E68 P14 D14 MM BMP4 10ng/ml,,

iPSC,BIOTIME,GPL10588,MBA_4121,E68 P14 D14 MM RA 1uM (-DEX),,

iPSC,BIOTIME,GPL10588,MBA_4122,E68 P14 D14 confluence SB431542 in MV2 with bFGF 10ng/ml and VEGFA 1ong/ml,,

iPSC,BIOTIME,GPL10588,MBA_4124,EN13 P12 D14MM RA 1um (-DEX) DE3 screen,,

iPSC,BIOTIME,GPL10588,MBA_4125,EN13 P12 D14 MM BMP4 10ng/ml DE3 screen,,

iPSC,BIOTIME,GPL10588,MBA_4126,T42 P20 ctrl DE3 screen,,

iPSC,BIOTIME,GPL10588,MBA_4127,T42 P20 DE3 screen D14 MM BMP2 50ng/ml + TGFb3 10ng/ml,,

iPSC,BIOTIME,GPL10588,MBA_4128,T42 P20 DE3 screen D14 MM BMP4 10ng/ml ,,

iPSC,BIOTIME,GPL10588,MBA_4129,T42 P20 DE3 screen D14 SB431542 in supplemented MV2 FGF2 10ng/ml and VEGFA 10ng/ml,,

iPSC,BIOTIME,GPL10588,MBA_4130,E69 P22 ctrl DE3 screen,,

iPSC,BIOTIME,GPL10588,MBA_4131,E69 P22 DE3 screen D14 MM BMP2 50ng/ml + TGFb3 10ng/ml,,

iPSC,BIOTIME,GPL10588,MBA_4132,E69 P22 DE3 screen D14 MM BMP4 10ng/ml ,,

iPSC,BIOTIME,GPL10588,MBA_4133,E69 P22 DE3 screen D14 SB431542 10uM in supplemented MV2 bFGF 10ng/ml + VEGFA 10ng/ml,,

iPSC,BIOTIME,GPL10588,MBA_4134,E69 P22 DE3 screen D14 MM RA 1uM,,

iPSC,BIOTIME,GPL10588,MBA_4136,EN47 P13 D14 MM TGFb3 10ng/ml + BMP2 50ng/ml,,

iPSC,BIOTIME,GPL10588,MBA_4137,EN47 P13 D14 MM BMP4 10ng/ml,,

iPSC,BIOTIME,GPL10588,MBA_4138,EN47 P13MM RA 1uM (no dexamethasone),,

iPSC,BIOTIME,GPL10588,MBA_4139,EN47 P13 D14 confluence 10uM SB431542 in MV2 with bFGF 10ng/ml and VEGFA 10ng/ml ,,

iPSC,BIOTIME,GPL10588,MBA_4141,C4ELSR10 P20 D14 MM BMP2 50ng/ml + TGFb3 10ng/ml,,

iPSC,BIOTIME,GPL10588,MBA_4142,C4ELSR10 P20 D14 MM BMP4 10ng/ml ,,

iPSC,BIOTIME,GPL10588,MBA_4143,C4ELSR10 P20 D14 MM RA 1uM no dex,,

iPSC,BIOTIME,GPL10588,MBA_4144,C4ELSR10 P20 confluence 10uM SB431542 in MV2 with bFGF 10ng/ml and VEGFA 10ng/ml ,,

iPSC,BIOTIME,GPL10588,MBA_4145,W11 P12 D14 MM RA 1uM no dex,,

iPSC,BIOTIME,GPL10588,MBA_4146,W11 P12 D14 MM BMP4 10ng/ml,,

iPSC,BIOTIME,GPL10588,MBA_4147,W11 P12 D14 MM BMP2 50ng/ml + TGFb3 10ng/ml ,,

iPSC,BIOTIME,GPL10588,MBA_4148,W11 P12 D14 confluence 10uM SB431542 in MV2 with bFGF 10ng/ml and VEGFA 10ng/ml ,,

iPSC,BIOTIME,GPL10588,MBA_4150,7PEND24 P22 D14 MM BMP4 10ng/ml DE3 screen ,,

iPSC,BIOTIME,GPL10588,MBA_4151,7PEND24 P22 D14 MM BMP2 50ng/ml +TGFb3 10ng/ml 4 DE3 screen ,,

iPSC,BIOTIME,GPL10588,MBA_4152,7PEND24 P22 D14 MM RA 1uM (no dexamethasone) DE3 screen ,,

iPSC,BIOTIME,GPL10588,MBA_4153,7PEND24 P22 D14 Hystem BMP4 10ng/ml tendon,,

iPSC,BIOTIME,GPL10588,MBA_4155,7PEND24 P22 D14 Hystem GDF6 100ng/ml ,,

iPSC,BIOTIME,GPL10588,MBA_4156,4D20.8 P17 ctrl Hystem,,

iPSC,BIOTIME,GPL10588,MBA_4157,4D20.8 P17 D14 Hystem TGFb3 10ng/ml,,

iPSC,BIOTIME,GPL10588,MBA_4158,4D20.8 P17 D14 Hystem TGFb3 10ng/ml,,

iPSC,BIOTIME,GPL10588,MBA_4159,4D20.8 P17 D14 Hystem TGFb3 10ng/ml,,

iPSC,BIOTIME,GPL10588,MBA_4160,4D20.8 P18 D14 Hystem TGFb3 10ng/ml +GDF5 100ng/ml,,

iPSC,BIOTIME,GPL10588,MBA_4161,4D20.8 P18 D14 Hystem TGFb3 10ng/ml +GDF5 100ng/ml,,

iPSC,BIOTIME,GPL10588,MBA_4162,4D20.8 P18 D14 Hystem TGFb3 10ng/ml +GDF5 100ng/ml,,

iPSC,BIOTIME,GPL10588,MBA_4163,4D20.8 P18 D14 Hystem TGFb3 10ng/ml +BMP7 100ng/ml,,

iPSC,BIOTIME,GPL10588,MBA_4164,4D20.8 P18 D14 Hystem TGFb3 10ng/ml +BMP7 100ng/ml,,

iPSC,BIOTIME,GPL10588,MBA_4165,4D20.8 P18 D14 Hystem TGFb3 10ng/ml +BMP7 100ng/ml,,

iPSC,BIOTIME,GPL10588,MBA_4166,4D20.8 P18 D14 Hystem TGFb3 10ng/ml +BMP6 30ng/ml,,

iPSC,BIOTIME,GPL10588,MBA_4167,4D20.8 P18 D14 Hystem TGFb3 10ng/ml +BMP6 30ng/ml,,

iPSC,BIOTIME,GPL10588,MBA_4168,4D20.8 P18 D14 Hystem TGFb3 10ng/ml +BMP6 30ng/ml,,

iPSC,BIOTIME,GPL10588,MBA_4169,4D20.8 P18 D14 Hystem TGFb3 10ng/ml +BMP4 10ng/ml,,

iPSC,BIOTIME,GPL10588,MBA_4172,4D20.8 P18 D14 Hystem TGFb3 10ng/ml +BMP2 50ng/ml,,

iPSC,BIOTIME,GPL10588,MBA_4173,4D20.8 P18 D14 Hystem TGFb3 10ng/ml +BMP2 50ng/ml,,

iPSC,BIOTIME,GPL10588,MBA_4174,4D20.8 P18 D14 Hystem TGFb3 10ng/ml +BMP2 50ng/ml,,

iPSC,BIOTIME,GPL10588,MBA_4175,E85 P17 D14 Hystem TGFb3 10ng/ml,,

iPSC,BIOTIME,GPL10588,MBA_4176,E85 P17 D14 Hystem RA 1uM,,

iPSC,BIOTIME,GPL10588,MBA_4177,E85 P17 D14 Hystem GDF5 100ng/ml,,

iPSC,BIOTIME,GPL10588,MBA_4178,E85 P17 D14 Hystem GDF5 100ng/ml +TGFb3 10ng/ml,,

iPSC,BIOTIME,GPL10588,MBA_4179,E85 P17 D14 Hystem BMP7 100ng/ml,,

iPSC,BIOTIME,GPL10588,MBA_4180,E85 P17 D14 Hystem BMP7 100ng/ml +TGFb3 10ng/ml,,

iPSC,BIOTIME,GPL10588,MBA_4181,E85 P17 D14 Hystem BMP4 10ng/ml,,

iPSC,BIOTIME,GPL10588,MBA_4182,E85 P17 D14 Hystem BMP4 10ng/ml +TGFb3 10ng/ml,,

iPSC,BIOTIME,GPL10588,MBA_4183,E85 P17 D14 Hystem BMP2 50ng/ml,,

iPSC,BIOTIME,GPL10588,MBA_4184,E85 P17 D14 Hystem BMP2 50ng/ml + TGFb3 10ng/ml,,

AC,BIOTIME,GPL10588,MBA_4185,BH2 P6 (early aging) log phase,,

AC,BIOTIME,GPL10588,MBA_4186,SR1 P6 (early aging) log phase,,

AC,BIOTIME,GPL10588,MBA_4188,BH2 P6 (Aging early passage) 0.5% FBS 5day fast,,

AC,BIOTIME,GPL10588,MBA_4189,SR1 P6 (Aging early passage) 0.5% FBS 5day fast,,

AC,BIOTIME,GPL10588,MBA_4191,BH2 P10 Aging middle passage 2 days after feed,,

AC,BIOTIME,GPL10588,MBA_4192,SR1 P13 Aging middle passage 2 days after feed,,

AC,BIOTIME,GPL10588,MBA_4193,Xgene P16 Aging middle passage 2 days after feed,,

AC,BIOTIME,GPL10588,MBA_4194,BH2 P10 Aging middle passage 0.5% Serum 5D,,

AC,BIOTIME,GPL10588,MBA_4195,SR1 P13 Aging middle passage 0.5% Serum 5D,,

AC,BIOTIME,GPL10588,MBA_4196,Xgene P16 (Aging middle passage) 0.5% FBS 5day fast,,

AC,BIOTIME,GPL10588,MBA_4197,BH2 P14 Aging Senescent log growth 2D,,

AC,BIOTIME,GPL10588,MBA_4198,SR1 P20 Aging Senescent log growth 2D,,

AC,BIOTIME,GPL10588,MBA_4199,Xgene P21 Aging Senescent log growth 2D,,

AC,BIOTIME,GPL10588,MBA_4200,BH2 P14 Aging Senescent 0.5% serum 5D,,

AC,BIOTIME,GPL10588,MBA_4201,SR1 P20 Aging Senescent 0.5% serum 5D,,

AC,BIOTIME,GPL10588,MBA_4202,Xgene P21 Aging Senescent 0.5% serum 5D,,

iPSC,BIOTIME,GPL10588,MBA_4227,U31 P16 D14 Hystem TGFb3 10ng/ml,,

iPSC,BIOTIME,GPL10588,MBA_4228,U31 P16 D14 Hystem GDF5 100ng/ml,,

iPSC,BIOTIME,GPL10588,MBA_4229,U31 P16 D14 Hystem GDF5 100ng/ml + TGFb3 10ng/ml,,

iPSC,BIOTIME,GPL10588,MBA_4230,U31 P16 D14 Hystem BMP7 100ng/ml,,

iPSC,BIOTIME,GPL10588,MBA_4231,U31 P16 D14 Hystem BMP7 100ng/ml + TGFb3 10ng/ml,,

iPSC,BIOTIME,GPL10588,MBA_4232,U31 P16 D14 Hystem BMP4 10ng/ml,,

iPSC,BIOTIME,GPL10588,MBA_4233,U31 P16 D14 Hystem BMP4 10ng/ml + TGFb3 10ng/ml,,

iPSC,BIOTIME,GPL10588,MBA_4234,U31 P16 D14 Hystem BMP2 50ng/ml ,,

iPSC,BIOTIME,GPL10588,MBA_4235,U31 P16 D14 Hystem BMP2 50ng/ml + TGFb3 10ng/ml,,

iPSC,BIOTIME,GPL10588,MBA_4236,U31 P16 D14 confluence 10uM SB431542 in MV2 with bFGF 10ng/ml and VEGFA 10ng/ml ,,

iPSC,BIOTIME,GPL10588,MBA_4237,E15 P18 D14 Hystem TGFb3 10ng/ml,,

iPSC,BIOTIME,GPL10588,MBA_4238,E15 P18 D14 Hystem GDF5 100ng/ml + TGFb3 10ng/ml,,

iPSC,BIOTIME,GPL10588,MBA_4239,E15 P18 D14 Hystem BMP7 100ng/ml,,

iPSC,BIOTIME,GPL10588,MBA_4240,E15 P18 D14 Hystem BMP7 100ng/ml +TGFb3 10ng/ml,,

iPSC,BIOTIME,GPL10588,MBA_4241,E15 P18 D14 Hystem BMP6 30ng/ml +TGFb3 10ng/ml,,

iPSC,BIOTIME,GPL10588,MBA_4243,E15 P18 D14 Hystem BMP2 50ng/ml,,

iPSC,BIOTIME,GPL10588,MBA_4244,E15 P18 D14 Hystem BMP2 50ng/ml +TGFb3 10ng/ml,,

iPSC,BIOTIME,GPL10588,MBA_4245,SM30 P17 D14 Hystem TGFb3 10ng/ml,,

iPSC,BIOTIME,GPL10588,MBA_4246,SM30 P17 D14 Hystem BMP2 50ng/ml +TGF3 10ng/ml,,

iPSC,BIOTIME,GPL10588,MBA_4248,SM30 P17 D14 Hystem BMP6 30ng/ml +TGF3 10ng/ml,,

iPSC,BIOTIME,GPL10588,MBA_4249,SM30 P17 D14 Hystem BMP7 100ng/ml +TGF3 10ng/ml,,

iPSC,BIOTIME,GPL10588,MBA_4250,SM30 P17 D14 Hystem GDF5 100ng/ml +TGF3 10ng/ml,,

iPSC,BIOTIME,GPL10588,MBA_4251,SM30 P17 D14 Hystem BMP2 50ng/ml ,,

iPSC,BIOTIME,GPL10588,MBA_4252,SM30 P17 D14 Hystem BMP4 10ng/ml,,

iPSC,BIOTIME,GPL10588,MBA_4253,SM30 P17 D14 Hystem BMP6 30ng/ml ,,

iPSC,BIOTIME,GPL10588,MBA_4254,SM30 P17 D14 Hystem BMP7 100ng/ml ,,

iPSC,BIOTIME,GPL10588,MBA_4255,SM30 P17 D14 Hystem GDF5 100ng/ml ,,

AC,BIOTIME,GPL10588,MBA_4257,hMSC P5 D14 Hystem TGFb3 10ng/ml,,

AC,BIOTIME,GPL10588,MBA_4258,hMSC P5 D14 Hystem GDF5 100ng/ml,,

AC,BIOTIME,GPL10588,MBA_4259,hMSC P5 D14 Hystem GDF5 100ng/ml + TGFb3 10ng/ml,,

AC,BIOTIME,GPL10588,MBA_4260,hMSC P5 D14 Hystem BMP7 100ng/ml,,

AC,BIOTIME,GPL10588,MBA_4261,hMSC P5 D14 Hystem BMP7 100ng/ml + TGFb3 10ng/ml,,

AC,BIOTIME,GPL10588,MBA_4262,hMSC P5 D14 Hystem BMP6 30ng/ml,,

AC,BIOTIME,GPL10588,MBA_4263,hMSC P5 D14 Hystem BMP6 30ng/ml + TGFb3 10ng/ml,,

AC,BIOTIME,GPL10588,MBA_4264,hMSC P5 D14 Hystem BMP4 10ng/ml,,

AC,BIOTIME,GPL10588,MBA_4266,hMSC P5 D14 Hystem BMP2 50ng/ml,,

AC,BIOTIME,GPL10588,MBA_4267,hMSC P5 D14 Hystem BMP2 50ng/ml + TGFb3 10ng/ml,,

iPSC,BIOTIME,GPL10588,MBA_4268,7PEND24 P23 D14 Hystem TGFb3 10ng/ml,,

iPSC,BIOTIME,GPL10588,MBA_4269,7PEND24 P23 D14 Hystem BMP2 50ng/ml,,

iPSC,BIOTIME,GPL10588,MBA_4270,7PEND24 P23 D14 Hystem BMP2 50ng/ml + TGFb3 10ng/ml,,

iPSC,BIOTIME,GPL10588,MBA_4271,7PEND24 P23 D14 Hystem BMP4 10ng/ml ,,

iPSC,BIOTIME,GPL10588,MBA_4273,7PEND24 P23 D14 Hystem BMP6 30ng/ml ,,

iPSC,BIOTIME,GPL10588,MBA_4274,7PEND24 P23 D14 Hystem BMP6 30ng/ml + TGFb3 10ng/ml,,

iPSC,BIOTIME,GPL10588,MBA_4275,7PEND24 P23 D14 Hystem BMP7 100ng/ml ,,

iPSC,BIOTIME,GPL10588,MBA_4276,7PEND24 P23 D14 Hystem BMP7 100ng/ml + TGFb3 10ng/ml,,

iPSC,BIOTIME,GPL10588,MBA_4277,7PEND24 P23 D14 Hystem GDF5 100ng/ml,,

iPSC,BIOTIME,GPL10588,MBA_4278,7PEND24 P23 D14 Hystem GDF5 100ng/ml + TGFb3 10ng/ml,,

iPSC,BIOTIME,GPL10588,MBA_4279,SK11 P16 D14 Hystem TGFb3 10ng/ml,,

iPSC,BIOTIME,GPL10588,MBA_4280,SK11 P16 D14 Hystem BMP2 50ng/ml,,

iPSC,BIOTIME,GPL10588,MBA_4281,SK11 P16 D14 Hystem BMP2 50ng/ml + TGFb3 10ng/ml,,

iPSC,BIOTIME,GPL10588,MBA_4282,SK11 P16 D14 Hystem BMP4 10ng/ml ,,

iPSC,BIOTIME,GPL10588,MBA_4284,SK11 P16 D14 Hystem BMP6 30ng/ml,,

iPSC,BIOTIME,GPL10588,MBA_4285,SK11 P16 D14 Hystem BMP6 30ng/ml + TGFb3 10ng/ml,,

iPSC,BIOTIME,GPL10588,MBA_4286,SK11 P16 D14 Hystem BMP7 100ng/ml,,

iPSC,BIOTIME,GPL10588,MBA_4287,SK11 P16 D14 Hystem BMP7 100ng/ml + TGFb3 10ng/ml,,

iPSC,BIOTIME,GPL10588,MBA_4288,SK11 P16 D14 Hystem GDF5 100ng/ml,,

iPSC,BIOTIME,GPL10588,MBA_4289,SK11 P16 D14 Hystem GDF5 100ng/ml + TGFb3 10ng/ml,,

iPSC,BIOTIME,GPL10588,MBA_4290,MEL2 P23 D14 Hystem TGFb3 10ng/ml,,

iPSC,BIOTIME,GPL10588,MBA_4291,MEL2 P23 D14 Hystem TGFb3 10ng/ml +BMP2 50ng/ml,,

iPSC,BIOTIME,GPL10588,MBA_4293,MEL2 P23 D14 Hystem TGFb3 10ng/ml +BMP6 30ng/ml,,

iPSC,BIOTIME,GPL10588,MBA_4294,MEL2 P23 D14 Hystem TGFb3 10ng/ml +BMP7 100ng/ml,,

iPSC,BIOTIME,GPL10588,MBA_4295,MEL2 P23 D14 Hystem TGFb3 10ng/ml +GDF5 100ng/ml,,

iPSC,BIOTIME,GPL10588,MBA_4296,MEL2 P23 D14 Hystem BMP2 50ng/ml,,

iPSC,BIOTIME,GPL10588,MBA_4297,MEL2 P23 D14 Hystem BMP4 10ng/ml,,

iPSC,BIOTIME,GPL10588,MBA_4298,MEL2 P23 D14 Hystem BMP6 30ng/ml,,

iPSC,BIOTIME,GPL10588,MBA_4299,MEL2 P23 D14 Hystem BMP7 100ng/ml,,

iPSC,BIOTIME,GPL10588,MBA_4300,MEL2 P23 D14 Hystem GDF5 100ng/ml,,

iPSC,BIOTIME,GPL10588,MBA_4301,E15 P18 D14 Hystem TGFb3 10ng/ml,,

iPSC,BIOTIME,GPL10588,MBA_4302,E15 P18 D14 Hystem GDF5 100ng/ml,,

iPSC,BIOTIME,GPL10588,MBA_4303,E15 P18 D14 Hystem GDF5 100ng/ml + TGFb3 10ng/ml,,

iPSC,BIOTIME,GPL10588,MBA_4304,E15 P18 D14 Hystem BMP7 100ng/ml,,

iPSC,BIOTIME,GPL10588,MBA_4305,E15 P18 D14 Hystem BMP7 100ng/ml +TGFb3 10ng/ml,,

iPSC,BIOTIME,GPL10588,MBA_4306,E15 P18 D14 Hystem BMP6 30ng/ml,,

iPSC,BIOTIME,GPL10588,MBA_4307,E15 P18 D14 Hystem BMP6 30ng/ml +TGFb3 10ng/ml,,

iPSC,BIOTIME,GPL10588,MBA_4308,E15 P18 D14 Hystem BMP4 10ng/ml,,

iPSC,BIOTIME,GPL10588,MBA_4310,E15 P18 D14 Hystem BMP2 50ng/ml,,

iPSC,BIOTIME,GPL10588,MBA_4311,E15 P18 D14 Hystem BMP2 50ng/ml +TGFb3 10ng/ml,,

iPSC,BIOTIME,GPL10588,MBA_4312,4D20.8 LacZ P20 D14 MM ,,

iPSC,BIOTIME,GPL10588,MBA_4313,4D20.8 LacZ P20 D14 MM TGF10ng/ml +BMP7 100ng/ml,,

iPSC,BIOTIME,GPL10588,MBA_4365,U31 P17 D14 MM BMP2 50ng/ml + TGFb3 1ng/ml,,

iPSC,BIOTIME,GPL10588,MBA_4366,U31 P17 D14 MM BMP4 10ng/ml,,

iPSC,BIOTIME,GPL10588,MBA_4367,U31 P17 D14 confluence on matrigel with MV2 medium + VEGFA 10ng/ml + bFGF 10ng/ml + SB431542 10uM,,

iPSC,BIOTIME,GPL10588,MBA_4368,CM02 P25 D14 MM BMP4 10ng/ml,,

iPSC,BIOTIME,GPL10588,MBA_4369,CM02 P25 D14 confluent matrigel MV2 +bFGF 10ng/ml VEGFA 10ng/ml + SB431542 10uM,,

iPSC,BIOTIME,GPL10588,MBA_4370,CM02 P25 D14 MM BMP2 50ng/ml + TGFb3 10ng/ml,,

iPSC,BIOTIME,GPL10588,MBA_4371,CM02 P25 D14 MM -dex + RA 1uM,,

iPSC,BIOTIME,GPL10588,MBA_4373,SM35 P15 D14 MM BMP4 10ng/ml ,,

iPSC,BIOTIME,GPL10588,MBA_4374,SM35 P15 D14 confluence fast medium with RA 1uM,,

iPSC,BIOTIME,GPL10588,MBA_4375,SM35 P15 D14 confluence MV2 + bFGF 10ng/ml + VEGFA 10ng/ml + SB431542 10uM,,

iPSC,BIOTIME,GPL10588,MBA_4377,SM22 P12 D14 confluence Fast media RA 1uM,,

iPSC,BIOTIME,GPL10588,MBA_4378,SM22 P12 D14 confluence MV2 + 10ng/ml VEGFA + 10ng/ml bFGF + SB431542 10uM,,

iPSC,BIOTIME,GPL10588,MBA_4379,SM22 P12 D14 MM BMP2 50ng/ml + TGFb3 10ng/ml,,

iPSC,BIOTIME,GPL10588,MBA_4380,SM22 P14 D14 MM BMP4 10ng/ml ,,

iPSC,BIOTIME,GPL10588,MBA_4381,4D20.8 P17 D14 Hystem TGFb3 10ng/ml,,

iPSC,BIOTIME,GPL10588,MBA_4382,4D20.8 P17 D14 Hystem GDF5 100ng/ml,,

iPSC,BIOTIME,GPL10588,MBA_4383,4D20.8 P17 D14 Hystem GDF5 100ng/ml + TGFb3 10ng/ml,,

iPSC,BIOTIME,GPL10588,MBA_4384,4D20.8 P17 D14 Hystem BMP7 100ng/ml,,

iPSC,BIOTIME,GPL10588,MBA_4385,4D20.8 P17 D14 Hystem BMP7 100ng/ml + TGFb3 10ng/ml,,

iPSC,BIOTIME,GPL10588,MBA_4386,4D20.8 P17 D14 Hystem BMP6 30ng/ml,,

iPSC,BIOTIME,GPL10588,MBA_4387,4D20.8 P17 D14 Hystem BMP6 30ng/ml + TGFb3 10ng/ml,,

iPSC,BIOTIME,GPL10588,MBA_4388,4D20.8 P17 D14 Hystem BMP4 10ng/ml,,

iPSC,BIOTIME,GPL10588,MBA_4390,4D20.8 P17 D14 Hystem BMP2 50ng/ml,,

iPSC,BIOTIME,GPL10588,MBA_4391,4D20.8 P17 D14 Hystem BMP2 50ng/ml + TGFb3 10ng/ml,,

iPSC,BIOTIME,GPL10588,MBA_4392,7PEND24 P22 D14 Hystem TGFb3 10ng/ml,,

iPSC,BIOTIME,GPL10588,MBA_4393,7PEND24 P22 D14 Hystem BMP2 50ng/ml + TGFb3 10ng/ml,,

iPSC,BIOTIME,GPL10588,MBA_4395,7PEND24 P22 D14 Hystem BMP6 30ng/ml + TGFb3 10ng/ml,,

iPSC,BIOTIME,GPL10588,MBA_4396,7PEND24 P22 D14 Hystem BMP7 100ng/ml + TGFb3 10ng/ml,,

iPSC,BIOTIME,GPL10588,MBA_4397,7PEND24 P22 D14 Hystem GDF5 100ng/ml + TGFb3 10ng/ml,,

iPSC,BIOTIME,GPL10588,MBA_4398,7PEND24 P21 D14 Hystem TGFb3 10ng/ml,,

iPSC,BIOTIME,GPL10588,MBA_4399,7PEND24 P21 D14 Hystem BMP2 50ng/ml + TGFb3 10ng/ml,,

iPSC,BIOTIME,GPL10588,MBA_4401,7PEND24 P21 D14 Hystem BMP6 30ng/ml + TGFb3 10ng/ml,,

iPSC,BIOTIME,GPL10588,MBA_4402,7PEND24 P21 D14 Hystem BMP7 100ng/ml + TGFb3 10ng/ml,,

iPSC,BIOTIME,GPL10588,MBA_4403,7PEND24 P21 D14 Hystem GDF5 100ng/ml + TGFb3 10ng/ml,,

iPSC,BIOTIME,GPL10588,MBA_4404,7PEND24 P21 D14 Hystem BMP2 50ng/ml,,

iPSC,BIOTIME,GPL10588,MBA_4405,7PEND24 P21 D14 Hystem BMP4 10ng/ml ,,

iPSC,BIOTIME,GPL10588,MBA_4406,7PEND24 P21 D14 Hystem BMP6 30ng/ml ,,

iPSC,BIOTIME,GPL10588,MBA_4407,7PEND24 P21 D14 Hystem BMP7 100ng/ml,,

iPSC,BIOTIME,GPL10588,MBA_4408,7PEND24 P21 D14 Hystem GDF5 100ng/ml,,

iPSC,BIOTIME,GPL10588,MBA_4409,7SMOO32 P17 D14 Hystem TGFb3 10ng/ml,,

iPSC,BIOTIME,GPL10588,MBA_4410,7SMOO32 P17D14 Hystem BMP2 50ng/ml,,

iPSC,BIOTIME,GPL10588,MBA_4411,7SMOO32 P17 D14 Hystem TGFb3 10ng/ml +BMP2 50ng/ml,,

iPSC,BIOTIME,GPL10588,MBA_4412,7SMOO32 P17 D14 Hystem BMP4 10ng/ml,,

iPSC,BIOTIME,GPL10588,MBA_4414,7SMOO32 P17 D14 Hystem BMP6 30ng/ml,,

iPSC,BIOTIME,GPL10588,MBA_4415,7SMOO32 P17 D14 Hystem TGFb3 10ng/ml +BMP6 30ng/ml,,

iPSC,BIOTIME,GPL10588,MBA_4416,7SMOO32 P17 D14 Hystem BMP7 100ng/ml,,

iPSC,BIOTIME,GPL10588,MBA_4417,7SMOO32 P17 D14 Hystem TGFb3 10ng/ml +BMP7 100ng/ml,,

iPSC,BIOTIME,GPL10588,MBA_4418,7SMOO32 P17 D14 Hystem GDF5 100ng/ml,,

iPSC,BIOTIME,GPL10588,MBA_4419,7SMOO32 P17 D14 Hystem TGFb3 10ng/ml +GDF5 100ng/ml,,

AC,BIOTIME,GPL10588,MBA_4421,hMSC P7 D14 Hystem TGFb3 10ng/ml,,

AC,BIOTIME,GPL10588,MBA_4422,hMSC P7 D14 Hystem GDF5 100ng/ml,,

AC,BIOTIME,GPL10588,MBA_4423,hMSC P7 D14 Hystem GDF5 100ng/ml + TGFb3 10ng/ml,,

AC,BIOTIME,GPL10588,MBA_4424,hMSC P7 D14 Hystem BMP7 100ng/ml + TGFb3 10ng/ml,,

AC,BIOTIME,GPL10588,MBA_4425,hMSC P7 D14 Hystem BMP6 30ng/ml + TGFb3 10ng/ml,,

AC,BIOTIME,GPL10588,MBA_4427,hMSC P7 D14 Hystem BMP2 50ng/ml + TGFb3 10ng/ml,,

AC,BIOTIME,GPL10588,MBA_4428,hMSC P7 D14 Hystem BMP2 50ng/ml,,

AC,BIOTIME,GPL10588,MBA_4429,hMSC P7 D14 Hystem BMP4 10ng/ml,,

AC,BIOTIME,GPL10588,MBA_4430,hMSC P7 D14 Hystem BMP6 30ng/ml,,

AC,BIOTIME,GPL10588,MBA_4431,hMSC P7 D14 Hystem BMP7 100ng/ml,,

iPSC,BIOTIME,GPL10588,MBA_4432,E15 P19 D42 Hystem GDF5 100ng/ml + TGFb3 10ng/ml,,

iPSC,BIOTIME,GPL10588,MBA_4433,E15 P22 D14 Hystem TGFb3 10ng/ml,,

iPSC,BIOTIME,GPL10588,MBA_4434,E15 P22 D14 Hystem BMP2 50ng/ml +TGFb3 10ng/ml,,

iPSC,BIOTIME,GPL10588,MBA_4436,E15 P22 D14 Hystem BMP6 30ng/ml +TGFb3 10ng/ml,,

iPSC,BIOTIME,GPL10588,MBA_4437,E15 P22 D14 Hystem BMP7 100ng/ml +TGFb3 10ng/ml,,

iPSC,BIOTIME,GPL10588,MBA_4438,E15 P22 D14 Hystem GDF5 100ng/ml + TGFb3 10ng/ml,,

iPSC,BIOTIME,GPL10588,MBA_4439,E15 P22 D14 Hystem BMP2 50ng/ml,,

iPSC,BIOTIME,GPL10588,MBA_4440,E15 P22 D14 Hystem BMP4 10ng/ml,,

iPSC,BIOTIME,GPL10588,MBA_4441,E15 P22 D14 Hystem BMP6 30ng/ml,,

iPSC,BIOTIME,GPL10588,MBA_4442,E15 P22 D14 Hystem BMP7 100ng/ml,,

iPSC,BIOTIME,GPL10588,MBA_4443,E15 P22 D14 Hystem GDF5 100ng/ml,,

iPSC,BIOTIME,GPL10588,MBA_4445,7SMOO32 P13 D14 Hystem TGFb3 10ng/ml,,

iPSC,BIOTIME,GPL10588,MBA_4446,7SMOO32 P13 D14 Hystem GDF5 100ng/ml,,

iPSC,BIOTIME,GPL10588,MBA_4447,7SMOO32 P13 D14 Hystem TGFb3 10ng/ml +GDF5 100ng/ml,,

iPSC,BIOTIME,GPL10588,MBA_4448,7SMOO32 P13 D14 Hystem BMP7 100ng/ml,,

iPSC,BIOTIME,GPL10588,MBA_4449,7SMOO32 P13 D14 Hystem TGFb3 10ng/ml +BMP7 100ng/ml,,

iPSC,BIOTIME,GPL10588,MBA_4450,7SMOO32 P13 D14 Hystem BMP6 30ng/ml,,

iPSC,BIOTIME,GPL10588,MBA_4451,7SMOO32 P13 D14 Hystem TGFb3 10ng/ml +BMP6 30ng/ml,,

iPSC,BIOTIME,GPL10588,MBA_4452,7SMOO32 P13 D14 Hystem BMP4 10ng/ml,,

iPSC,BIOTIME,GPL10588,MBA_4454,7SMOO32 P13 D14 Hystem BMP2 50ng/ml,,

iPSC,BIOTIME,GPL10588,MBA_4455,7SMOO32 P13 D14 Hystem TGFb3 10ng/ml +BMP2 50ng/ml,,

iPSC,BIOTIME,GPL10588,MBA_4456,7SMOO32 P13 D21 Hystem TGFb3 10ng/ml,,

iPSC,BIOTIME,GPL10588,MBA_4457,7SMOO32 P13 D21 Hystem TGFb3 10ng/ml +BMP7 100ng/ml,,

iPSC,BIOTIME,GPL10588,MBA_4458,EN47 P14 D14 MM RA 1uM,,

iPSC,BIOTIME,GPL10588,MBA_4459,EN47 P14 D14 MM BMP2 50ng/ml +TGFb3 10ng/ml,,

iPSC,BIOTIME,GPL10588,MBA_4460,EN47 P14 D14 confluence matrigel MV2 + bFGF 10ng/ml +VEGFA 10ng/ml+ SB431542 10uM ,,

iPSC,BIOTIME,GPL10588,MBA_4462,7PEND12 P13 D14 MM BMP4 10ng/ml,,

iPSC,BIOTIME,GPL10588,MBA_4463,7PEND12 P13 D14 confluence fast medium + RA 1uM,,

iPSC,BIOTIME,GPL10588,MBA_4464,7PEND12 P13 D14 matrigel confluence MV2 + VEGFA 10ng/ml + bFGF 10ng/ml + SB431542 10uM,,

iPSC,BIOTIME,GPL10588,MBA_4465,EN4 P13 ctrl,,

iPSC,BIOTIME,GPL10588,MBA_4466,EN4 P13 D14 confluent RA 1uM,,

iPSC,BIOTIME,GPL10588,MBA_4467,EN4 P13 D14 MM BMP2 50ng/ml + TGFb3 10ng/ml,,

iPSC,BIOTIME,GPL10588,MBA_4468,EN4 P13 D14 matrigel confluent MV2 + bFGF 10ng/ml +VEGFA 10ng/ml+ SB431542 10uM ,,

iPSC,BIOTIME,GPL10588,MBA_4470,EN7 P18 D21 Hystem BMP4 10ng/ml,,

iPSC,BIOTIME,GPL10588,MBA_4471,EN7 P18 D21 Hystem GDF5 100ng/ml,,

iPSC,BIOTIME,GPL10588,MBA_4472,EN7 P18 D21 Hystem BMP4 10ng/ml + TGFb3 10ng/ml,,

iPSC,BIOTIME,GPL10588,MBA_4473,EN7 P18 D21 Hystem GDF5 100ng/ml + TGFb3 10ng/ml,,

iPSC,BIOTIME,GPL10588,MBA_4475,EN26 P13 D21 Hystem BMP4 10ng/ml,,

iPSC,BIOTIME,GPL10588,MBA_4476,EN26 P13 D21 Hystem BMP4 10ng/ml + TGFb3 10ng/ml,,

iPSC,BIOTIME,GPL10588,MBA_4477,EN26 P13 D21 Hystem GDF5 100ng/ml + TGFb3 10ng/ml,,

iPSC,BIOTIME,GPL10588,MBA_4478,EN27 P14 ctrl,,

iPSC,BIOTIME,GPL10588,MBA_4479,EN27 P14 D21 Hystem BMP4 10ng/ml + TGFb3 10ng/ml,,

iPSC,BIOTIME,GPL10588,MBA_4480,EN27 P14 D21 Hystem BMP4 10ng/ml ,,

iPSC,BIOTIME,GPL10588,MBA_4481,EN27 P14 D21 Hystem GDF5 100ng/ml + TGFb3 10ng/ml,,

iPSC,BIOTIME,GPL10588,MBA_4482,EN27 P14 D21 Hystem GDF5 100ng/ml ,,

iPSC,BIOTIME,GPL10588,MBA_4484,EN13 P14 D21 Hystem BMP4 10ng/ml,,

iPSC,BIOTIME,GPL10588,MBA_4485,EN13 P14 D21 Hystem GDF5 100ng/ml,,

iPSC,BIOTIME,GPL10588,MBA_4486,EN13 P14 D21 Hystem BMP4 10ng/ml + TGFb3 10ng/ml,,

iPSC,BIOTIME,GPL10588,MBA_4487,EN13 P14 D21 Hystem GDF5 100ng/ml +TGFb3 10ng/ml,,

iPSC,BIOTIME,GPL10588,MBA_4488,EN31 P13 ctrl,,

iPSC,BIOTIME,GPL10588,MBA_4489,EN31 P13 D21 Hystem BMP4 10ng/ml + TGFb3 10ng/ml,,

iPSC,BIOTIME,GPL10588,MBA_4490,EN31 P13 D21 Hystem BMP4 10ng/ml ,,

iPSC,BIOTIME,GPL10588,MBA_4491,EN31 P13 D21 Hystem GDF5 100ng/ml + TGFb3 10ng/ml,,

iPSC,BIOTIME,GPL10588,MBA_4493,W10 P16 D14 Hystem TGFb3 10ng/ml,,

iPSC,BIOTIME,GPL10588,MBA_4494,W10 P16 D14 Hystem TGFb3 10ng/ml + BMP2 50ng/ml,,

iPSC,BIOTIME,GPL10588,MBA_4495,W10 P16 D14 Hystem TGFb3 10ng/ml + BMP4 10ng/ml,,

iPSC,BIOTIME,GPL10588,MBA_4496,W10 P16 D14 Hystem TGFb3 10ng/ml + BMP7 100ng/ml,,

iPSC,BIOTIME,GPL10588,MBA_4497,W10 P16 D14 Hystem TGFb3 10ng/ml + GDF5 100ng/ml,,

iPSC,BIOTIME,GPL10588,MBA_4498,W10 P16 D14 Hystem BMP2 50ng/ml,,

iPSC,BIOTIME,GPL10588,MBA_4499,W10 P16 D14 Hystem BMP4 10ng/ml,,

iPSC,BIOTIME,GPL10588,MBA_4500,W10 P16 D14 Hystem BMP7 100ng/ml,,

iPSC,BIOTIME,GPL10588,MBA_4501,W10 P16 D14 Hystem GDF5 100ng/ml,,

iPSC,BIOTIME,GPL10588,MBA_4502,W10 P16 D14 MM TGFb3 10ng/ml,,

iPSC,BIOTIME,GPL10588,MBA_4503,W10 P16 D14 MM TGFb3 10ng/ml + BMP2 50ng/ml,,

iPSC,BIOTIME,GPL10588,MBA_4504,W10 P16 D14 MM TGFb3 10ng/ml + BMP4 10ng/ml,,

iPSC,BIOTIME,GPL10588,MBA_4505,W10 P16 D14 MM TGFb3 10ng/ml + BMP7 100ng/ml,,

iPSC,BIOTIME,GPL10588,MBA_4506,W10 P16 D14 MM TGFb3 10ng/ml + GDF5 100ng/ml,,

iPSC,BIOTIME,GPL10588,MBA_4507,W10 P16 D14 MM BMP2 50ng/ml,,

iPSC,BIOTIME,GPL10588,MBA_4508,W10 P16 D14 MM BMP4 10ng/ml,,

iPSC,BIOTIME,GPL10588,MBA_4509,W10 P16 D14 MM BMP7 100ng/ml,,

iPSC,BIOTIME,GPL10588,MBA_4510,W10 P16 D14 MM GDF5 100ng/ml,,

iPSC,BIOTIME,GPL10588,MBA_4511,4D20.8 P15 D14 MM TGFb3 10ng/ml,,

iPSC,BIOTIME,GPL10588,MBA_4512,4D20.8 P15 D14 MM TGFb3 10ng/ml + KGN 10uM,,

iPSC,BIOTIME,GPL10588,MBA_4513,4D20.8 P15 D14 MM GDF5 100ng/ml + TGFb3 10ng/ml,,

iPSC,BIOTIME,GPL10588,MBA_4514,4D20.8 P15 D14 MM GDF5 100ng/ml + TGFb3 10ng/ml + KGN 10uM,,

iPSC,BIOTIME,GPL10588,MBA_4515,4D20.8 P16 D14 MM TGFb3 10ng/ml + DMSO 0.01%,,

iPSC,BIOTIME,GPL10588,MBA_4516,4D20.8 P16 D14 MM TGFb3 10ng/ml + Celecoxib 10uM,,

iPSC,BIOTIME,GPL10588,MBA_4517,4D20.8 P16 D14 MM BMP2 50ng/ml + TGFb3 10ng/ml + DMSO 0.01%,,

iPSC,BIOTIME,GPL10588,MBA_4518,4D20.8 P16 D14 MM BMP2 50ng/ml + TGFb3 10ng/ml + Celecoxib 10uM,,

iPSC,BIOTIME,GPL10588,MBA_4519,4D20.8 P16 D14 MM GDF5 100ng/ml + TGFb3 10ng/ml + DMSO 0.01%,,

iPSC,BIOTIME,GPL10588,MBA_4520,4D20.8 P16 D14 MM GDF5 100ng/ml + TGFb3 10ng/ml + Celecoxib 10uM,,

iPSC,BIOTIME,GPL10588,MBA_4521,7PEND24 P21 D42 Hystem GDF5 100ng/ml + TGFb3 10ng/ml,,

iPSC,BIOTIME,GPL10588,MBA_4522,SK11 P14 D42 Hystem GDF5 100ng/ml +TGFb3 10ng/ml,,

iPSC,BIOTIME,GPL10588,MBA_4523,"7SMOO32 P16 D42 Hystem GDF5 100ng/ml + TGFb3 10ng/ml Combined samples a,b,c",,

AC,BIOTIME,GPL10588,MBA_4524,hMSC2 P6 D42 Hystem GDF5 100ng/ml + TGFb3 10ng/ml,,

iPSC,BIOTIME,GPL10588,MBA_4525,SM30 P15 D42 Hystem GDF5 100ng/ml + TGFb3 10ng/ml,,

iPSC,BIOTIME,GPL10588,MBA_4526,4D20.8 P19 D42 Hystem GDF5 100ng/ml + TGFb3 10ng/ml,,

iPSC,BIOTIME,GPL10588,MBA_4527,MEL2 P21 D42 Hystem GDF5 100ng/ml + TGFb3 10ng/ml,,

iPSC,BIOTIME,GPL10588,MBA_4569,EN31 P13 D21 Hystem GDF5 100ng/ml ,,

iPSC,BIOTIME,GPL10588,MBA_4571,E69 P15 D14 MM BMP4 10ng/ml,,

iPSC,BIOTIME,GPL10588,MBA_4571,E69 P15 D14 MM BMP4 10ng/ml,,

iPSC,BIOTIME,GPL10588,MBA_4572,E69 P15 D21 MM BMP4 10ng/ml,,

iPSC,BIOTIME,GPL10588,MBA_4572,E69 P15 D21 MM BMP4 10ng/ml,,

iPSC,BIOTIME,GPL10588,MBA_4573,E69 P15 D21 Hystem BMP4 10ng/ml,,

iPSC,BIOTIME,GPL10588,MBA_4574,E69 P17 D21 Hystem BMP4 10ng/ml +TGFb3 10ng/ml,,

iPSC,BIOTIME,GPL10588,MBA_4575,E69 P17 D21 Hystem GDF5 100ng/ml +TGFb3 10ng/ml,,

iPSC,BIOTIME,GPL10588,MBA_4576,E69 P17 D21 Hystem DKK1 100ng/ml 0.5% serum fasting medium,,

iPSC,BIOTIME,GPL10588,MBA_4577,E69 P17 D21 Hystem SHH 250ng/ml 0.5% serum fasting medium,,

iPSC,BIOTIME,GPL10588,MBA_4578,E69 P17 D21 HystemSHH 250ng/ml + FGF8 100ng/ml 0.5% serum fasting medium ,,

iPSC,BIOTIME,GPL10588,MBA_4580,EN16 P14 D21Hystem BMP4 10ng/ml + TGFb3 10ng/ml,,

iPSC,BIOTIME,GPL10588,MBA_4581,EN16 P14 D21 Hystem BMP4 10ng/ml,,

iPSC,BIOTIME,GPL10588,MBA_4582,EN16 P14 D21 Hystem GDF5 100ng/ml + TGFb3 10ng/ml ,,

iPSC,BIOTIME,GPL10588,MBA_4583,EN16 P14 D21 Hystem GDF5 100ng/ml ,,

iPSC,BIOTIME,GPL10588,MBA_4584,EN16 P14 D21 Hystem DKK1 100ng/ml ,,

iPSC,BIOTIME,GPL10588,MBA_4586,EN18 P13 D21 Hystem BMP4 10ng/ml + TGFb3 10ng/ml,,

iPSC,BIOTIME,GPL10588,MBA_4587,EN18 P13 D21 Hystem BMP4 10ng/ml ,,

iPSC,BIOTIME,GPL10588,MBA_4588,EN18 P13 D21 Hystem GDF5 100ng/ml 100ng/ml + TGFb3 10ng/ml,,

iPSC,BIOTIME,GPL10588,MBA_4589,EN18 P13 D21 Hystem GDF5 100ng/ml ,,

iPSC,BIOTIME,GPL10588,MBA_4590,EN18 P13 D21 Hystem DKK1 100ng/ml,,

iPSC,BIOTIME,GPL10588,MBA_4592,EN8 P13 D21 Hystem BMP4 10ng/ml + TGFb3 10ng/ml,,

iPSC,BIOTIME,GPL10588,MBA_4593,EN8 P13 D21 Hystem BMP4 10ng/ml ,,

iPSC,BIOTIME,GPL10588,MBA_4594,EN8 P13 D21 Hystem GDF5 100ng/ml + TGFb3 10ng/ml,,

iPSC,BIOTIME,GPL10588,MBA_4595,EN8 P13 D21 Hystem GDF5 100ng/ml ,,

iPSC,BIOTIME,GPL10588,MBA_4596,EN8 P13 D21 Hystem DKK1 100ng/ml ,,

iPSC,BIOTIME,GPL10588,MBA_4598,E68 P21 D14 Hystem BMP4 10ng/ml,,

iPSC,BIOTIME,GPL10588,MBA_4599,E68 P21 D14 MM BMP4 10ng/ml,,

iPSC,BIOTIME,GPL10588,MBA_4600,E68 P21 D21 Hystem BMP4 10ng/ml,,

iPSC,BIOTIME,GPL10588,MBA_4601,E68 P21 D21 MM BMP4 10ng/ml,,

iPSC,BIOTIME,GPL10588,MBA_4603,T42 P20 D14 MM BMP4 10ng/ml,,

iPSC,BIOTIME,GPL10588,MBA_4603 ,T42 P20 D14 MM BMP4 10ng/ml,,

iPSC,BIOTIME,GPL10588,MBA_4604,T42 P20 D21 MM BMP4 10ng/ml,,

iPSC,BIOTIME,GPL10588,MBA_4606,ESI SK153 P15 D21 Hystem BMP4 10ng/ml + TGFb3 10ng/ml,,

iPSC,BIOTIME,GPL10588,MBA_4607,ESI SK153 P15 D21 Hystem GDF5 100ng/ml + TGFb3 10ng/ml,,

iPSC,BIOTIME,GPL10588,MBA_4608,ESI SK153 P15 D21 Hystem DKK1 100ng/ml 0.5% serum fasting medium,,

iPSC,BIOTIME,GPL10588,MBA_4609,ESI SK153 P15 D21 Hystem SHH 250ng/ml 0.5% serum fasting medium,,

iPSC,BIOTIME,GPL10588,MBA_4610,ESI SK153 P15 D21 Hystem SHH 250ng/ml + FGF8 100ng/ml 0.5% serum fasting medium,,

iPSC,BIOTIME,GPL10588,MBA_4611,4D20.8 P19 D21 Alginate GDF5 100ng/ml,,

iPSC,BIOTIME,GPL10588,MBA_4612,4D20.8 P19 D21 Alginate GDF5 100ng/ml + TGFb3 10ng/ml,,

iPSC,BIOTIME,GPL10588,MBA_4613,"4D20.8 P19 D42 Hystem GDF5 100ng/ml + TGF 10ng/ml, Cryoprotected-frozen-thawed, hystem construct placed in GDF5 100ng/ml +TGFb3 10ng/ml for 21 days",,

iPSC,BIOTIME,GPL10588,MBA_4614,E15 P17 D21 Alginate GDF5 100ng/ml + TGFb3 10ng/ml,,

iPSC,BIOTIME,GPL10588,MBA_4615,E15 P17 D21 Alginate BMP4 10ng/ml + TGFb3 10ng/ml,,

iPSC,BIOTIME,GPL10588,MBA_4616,E15 P17 D21 Alginate BMP7 100ng/ml + TGFb3 10ng/ml,,

iPSC,BIOTIME,GPL10588,MBA_4617,"E15 P17 D21 Alginate BMP4 10ng/ml + TGFb3 10ng/ml, depolymerize gel, then reseed cells as monolayer in same medium 8 days",,

iPSC,BIOTIME,GPL10588,MBA_4618,"E15 P17 D21 Alginate BMP7 100ng/ml + TGFb3 10ng/ml, depolymerize gel, then reseed cells as monolayer in same medium 8 days",,

iPSC,BIOTIME,GPL10588,MBA_4619,7PEND24 P14 D21 Alginate GDF5 100ng/ml + TGFb3 10ng/ml,,

iPSC,BIOTIME,GPL10588,MBA_4620,7PEND24 P14 D21 Alginate BMP6 50ng/ml + TGFb3 10ng/ml,,

iPSC,BIOTIME,GPL10588,MBA_4621,7PEND24 P14 D21 Alginate D21 BMP4 10ng/ml + TGFb3 10ng/ml,,

iPSC,BIOTIME,GPL10588,MBA_4622,"SM30 P15 D42 Hystem GDF100ng/ml + TGFb3 10ng/ml, cryopreserve (-196C) Hystem 1 month, thaw and put Hystem construct in same chondro medium 1 week",,

iPSC,BIOTIME,GPL10588,MBA_4623,"4D20.8 P19 D21 Alginate GDF5 100ng/ml + TGFb3 10ng/ml, depolymerize gel, then reseed cells as monolayer in same medium for 21 days",,

iPSC,BIOTIME,GPL10588,MBA_4624,"4D20.8 P19 D21 Alginate GDF5 100ng/ml + TGFb3 10ng/ml, depolymerize gel, then reseed cells as monolayer in same medium 12 days, then place growth medium 9 days",,

iPSC,BIOTIME,GPL10588,MBA_4626,EN7 P17 D21 Hystem BMP4 10ng/ml,,

iPSC,BIOTIME,GPL10588,MBA_4627,EN7 P17 D21 Hystem BMP4 10ng/ml + TGFb3 10ng/ml,,

iPSC,BIOTIME,GPL10588,MBA_4628,EN7 P17 D21 Hystem GDF5 100ng/ml + TGFb3 10ng/ml,,

iPSC,BIOTIME,GPL10588,MBA_4629,EN7 P17 D21 Hystem DKK1 100ng/ml 0.5% serum fasting medium,,

iPSC,BIOTIME,GPL10588,MBA_4630,EN7 P17 D21 Hystem SHH 250ng/ml 0.5% serum fasting medium,,

iPSC,BIOTIME,GPL10588,MBA_4631,EN7 P17 D21 Hystem SHH 250ng/ml + FGF8 100ng/ml 0.5% serum fasting medium,,

iPSC,BIOTIME,GPL10588,MBA_4699,E68 P23 D21 Hystem BMP4 10ng/ml + TGFb3 10ng/ml,,

iPSC,BIOTIME,GPL10588,MBA_4700,E68 P23 D21 Hystem GDF5 100ng/ml + TGFb3 10ng/ml,,

iPSC,BIOTIME,GPL10588,MBA_4701,E68 P23 D21 Hystem DKK1 100ng/ml 0.5% serum ,,

iPSC,BIOTIME,GPL10588,MBA_4702,E68 P23 D21 Hystem SHH 250ng/ml 0.5% serum,,

iPSC,BIOTIME,GPL10588,MBA_4703,E68 P23 D21 Hystem SHH 250ng/ml + FGF8 100ng/ml 0.5% serum,,

iPSC,BIOTIME,GPL10588,MBA_4705,E120 P15 D21 Hystem BMP4 10ng/ml + TGFb3 10ng/ml,,

iPSC,BIOTIME,GPL10588,MBA_4706,E120 P15 D21 Hystem BMP4 10ng/ml ,,

iPSC,BIOTIME,GPL10588,MBA_4707,E120 P15 D21 Hystem GDF5 100ng/ml + TGFb3 10ng/ml,,

iPSC,BIOTIME,GPL10588,MBA_4708,E120 P15 D21 Hystem DKK1 100ng/ml 0.5% serum ,,

iPSC,BIOTIME,GPL10588,MBA_4709,E120 P15 D21 Hystem SHH 250ng/ml 0.5% serum ,,

iPSC,BIOTIME,GPL10588,MBA_4710,E120 P15 D21 Hystem SHH 250ng/ml + FGF8 100ng/ml 0.5% serum,,

iPSC,BIOTIME,GPL10588,MBA_4712,RP1-DM10-18 P10 D21 Hystem BMP4 10ng/ml + TGFb3 10ng/ml,,

iPSC,BIOTIME,GPL10588,MBA_4713,RP1-DM10-18 P10 D21 Hystem BMP4 10ng/ml,,

iPSC,BIOTIME,GPL10588,MBA_4714,RP1-DM10-18 P10 D21 Hystem RA 1uM,,

iPSC,BIOTIME,GPL10588,MBA_4715,RP1-DM10-18 P10 D21 Hystem DKK1 100ng/ml 0.5% serum ,,

iPSC,BIOTIME,GPL10588,MBA_4716,RP1-DM10-18 P10 D21 Hystem SHH 250ng/ml,,

iPSC,BIOTIME,GPL10588,MBA_4717,RP1-DM10-18 P10 D21 Hystem SHH 250ng/ml + FGF8 100ng/ml 0.5% serum ,,

iPSC,BIOTIME,GPL10588,MBA_4719,EN42 P12 D21 Hystem BMP4 100ng/ml + TGFb3 10ng/ml ,,

iPSC,BIOTIME,GPL10588,MBA_4720,EN42 P12 D21 Hystem BMP4 100ng/ml ,,

iPSC,BIOTIME,GPL10588,MBA_4721,EN42 P12 D21 Hystem RA 1uM,,

iPSC,BIOTIME,GPL10588,MBA_4722,EN42 P12 D21 Hystem SHH 250ng/ml 0.5% serum ,,

iPSC,BIOTIME,GPL10588,MBA_4723,EN42 P12 D21 Hystem SHH 250ng/ml + FGF8 100ng/ml 0.5% serum,,

iPSC,BIOTIME,GPL10588,MBA_4725,RP1-DM10-19 P11 D21 Hystem BMP4 10ng/ml + TGFb3 10ng/ml,,

iPSC,BIOTIME,GPL10588,MBA_4726,RP1-DM10-19 P11 D21 Hystem BMP4 10ng/ml ,,

iPSC,BIOTIME,GPL10588,MBA_4727,RP1-DM10-19 P11 D21 Hystem RA 1uM,,

iPSC,BIOTIME,GPL10588,MBA_4728,RP1-DM10-19 P11 D21 Hystem DKK1 100ng/ml 0.5% serum + ITS,,

iPSC,BIOTIME,GPL10588,MBA_4729,RP1-DM10-19 P11 D21 Hystem SHH 250ng/ml 0.5% serum + ITS,,

iPSC,BIOTIME,GPL10588,MBA_4730,RP1-DM10-19 P11 D21 Hystem SHH 250ng/ml + FGF8 100ng/ml 0.5% serum + ITS,,

iPSC,BIOTIME,GPL10588,MBA_4732,RP1-MV2-8 P12 D21 Hystem BMP4 10ng/ml + TGFb3 10ng/ml,,

iPSC,BIOTIME,GPL10588,MBA_4733,RP1-MV2-8 P12 D21 Hystem BMP4 10ng/ml ,,

iPSC,BIOTIME,GPL10588,MBA_4734,RP1-MV2-8 P12 D21 Hystem RA 1uM,,

iPSC,BIOTIME,GPL10588,MBA_4735,RP1-MV2-8 P12 D21 Hystem DKK1 100ng/ml 0.5% serum+ ITS,,

iPSC,BIOTIME,GPL10588,MBA_4736,RP1-MV2-8 P12 D21 Hystem SHH 250ng/ml 0.5% serum + ITS,,

iPSC,BIOTIME,GPL10588,MBA_4737,RP1-MV2-8 P12 D21 Hystem SHH 250ng/ml + FGF8 100ng/ml 0.5% serum + ITS,,

iPSC,BIOTIME,GPL10588,MBA_4738,EN18 P15 D21Hystem BMP4 10ng/ml,,

iPSC,BIOTIME,GPL10588,MBA_4739,EN18 P15 D21 Hystem BMP4 10ng/ml + TGFb3 10ng/ml,,

iPSC,BIOTIME,GPL10588,MBA_4740,EN18 P15 D21 Hystem BMP2 50ng/ml + TGFb3 10ng/ml,,

iPSC,BIOTIME,GPL10588,MBA_4741,EN18 P15 D21 Hystem RA 1uM,,

iPSC,BIOTIME,GPL10588,MBA_4742,EN18 P15 D21 Hystem DKK1 100ng/ml 0.5% serum fast + ITS,,

iPSC,BIOTIME,GPL10588,MBA_4743,EN18 P15 D21 Hystem SHH 250ng/ml + FGF8 100ng/ml 0.5% serum + ITS,,

iPSC,BIOTIME,GPL10588,MBA_4745,EN22 P13 D14 adipose medium at confluence,,

iPSC,BIOTIME,GPL10588,MBA_4746,EN22 P13 D21 Hystem BMP4 10ng/ml,,

iPSC,BIOTIME,GPL10588,MBA_4747,EN22 P13 D21 Hystem BMP4 10ng/ml + TGFb3 10ng/ml,,

iPSC,BIOTIME,GPL10588,MBA_4748,EN22 P13 D21 Hystem RA 1uM,,

iPSC,BIOTIME,GPL10588,MBA_4749,EN22 P13 D21 Hystem BMP2 50ng/ml + TGFb3 10ng/ml,,

iPSC,BIOTIME,GPL10588,MBA_4750,EN22 P13 D21 confluence DKK1 100ng/ml 0.5% serum + ITS,,

iPSC,BIOTIME,GPL10588,MBA_4752,W8 P13 D21 Hystem BMP4 10ng/ml,,

iPSC,BIOTIME,GPL10588,MBA_4753,W8 P13 D21 Hystem BMP4 10ng/ml + TGFb3 10ng/ml,,

iPSC,BIOTIME,GPL10588,MBA_4754,W8 P13 D21 Hystem RA 1uM,,

iPSC,BIOTIME,GPL10588,MBA_4755,W8 P13 D21 Hystem BMP2 50ng/ml + TGFb3 10ng/ml,,

iPSC,BIOTIME,GPL10588,MBA_4756,W8 P13 D14 confluence adipose,,

iPSC,BIOTIME,GPL10588,MBA_4758,T42 P20 D21 Hystem BMP4 10ng/ml,,

iPSC,BIOTIME,GPL10588,MBA_4759,T42 P20 D21 Hystem BMP4 10ng/ml + TGFb3 10ng/ml,,

iPSC,BIOTIME,GPL10588,MBA_4760,T42 P20 D21 Hystem RA 1uM,,

iPSC,BIOTIME,GPL10588,MBA_4761,T42 P20 D21 MM BMP4 10ng/ml + TGFb3 10ng/ml,,

iPSC,BIOTIME,GPL10588,MBA_4762,T42 P20 D21 MM RA 1uM,,

iPSC,BIOTIME,GPL10588,MBA_4763,T42 P20 D21 MM BMP4 10ng/ml,,

iPSC,BIOTIME,GPL10588,MBA_4764,4D20.8 P15 D7 alg GDF5 100ng/ml + TGFb3 10 ng/ml ,,

iPSC,BIOTIME,GPL10588,MBA_4765,4D20.8 P15 D14 alg (LVG) GDF5 100ng/ml + TGFb3 10 ng/ml ,,

iPSC,BIOTIME,GPL10588,MBA_4766,4D20.8 P15 D14 alg (LVM) GDF5 100ng/ml + TGFb3 10 ng/ml ,,

iPSC,BIOTIME,GPL10588,MBA_4767,"4D20.8 P15 D14 alg (LVM) GDF5 100ng/ml + TGFb3 10 ng/ml, depolymerize replate D7 monolayer BMP4 10ng/ml + TGFb3 10ng/ml",,

iPSC,BIOTIME,GPL10588,MBA_4768,"4D20.8 P15 D14 alg (LVG) GDF5 100ng/ml + TGFb3 10 ng/ml, depolymerize, replate D7 monolayer BMP4 10ng/ml + TGFb3 10ng/ml + 10uM ROCK inh",,

iPSC,BIOTIME,GPL10588,MBA_4769,"4D20.8 P19 D14 alg BMP4 10ng/ml + TGFb3 10ng/ml, depolymerize and seed monolayer D7 BMP4 10ng/ml + TGFb3 10ng/ml",,

iPSC,BIOTIME,GPL10588,MBA_4770,4D20.8 P19 D21 alg GDF5 100ng/ml + TGFb3 10 ng/ml from cryo,,

iPSC,BIOTIME,GPL10588,MBA_4771,4D20.8 P19 D21 alg BMP4 10ng/ml + TGFb3 10ng/ml,,

iPSC,BIOTIME,GPL10588,MBA_4772,"4D20.8 P19 D21 alg GDF5 100ng/ml + TGFb3 10 ng/ml from cryo, D7 monolayer BMP4 10ng/ml + TGFb3 10ng/ml",,

iPSC,BIOTIME,GPL10588,MBA_4773,"4D20.8 P19 D21 alg BMP4 10ng/ml +TGFb3 10ng/ml, depolymerize, replate D7 monolayer same medium",,

iPSC,BIOTIME,GPL10588,MBA_4774,4D20.8 P19 D7 monolayer BMP4 10ng/ml + TGFb3 10ng/ml ,,

iPSC,BIOTIME,GPL10588,MBA_4775,4D20.8 P19 D7 MM BMP4 10ng/ml + TGFb3 10ng/ml ,,

iPSC,BIOTIME,GPL10588,MBA_4776,4D20.8 P19 D14 alg BMP4 10ng/ml + TGFb3 10ng/ml,,

iPSC,BIOTIME,GPL10588,MBA_4777,4D20.8 P19 D14 monolayer BMP4 10ng/ml + TGFb3 10ng/ml,,

iPSC,BIOTIME,GPL10588,MBA_4778,4D20.8 P19 D14 MM BMP4 10ng/ml + TGFb3 10ng/ml,,

iPSC,BIOTIME,GPL10588,MBA_4779,E15 P18 D7 alg BMP4 10ng/ml + TGFb3 10ng/ml,,

iPSC,BIOTIME,GPL10588,MBA_4780,7SMOO32 P24 D14 alg BMP4 10ng/ml + TGFb3 10ng/ml,,

iPSC,BIOTIME,GPL10588,MBA_4792,C4ELS5.6 P13 Neural Basal polylysine N2,,

iPSC,BIOTIME,GPL10588,MBA_4793,C4ELS5.6 P13 Neural Basal polylysine B27,,

iPSC,BIOTIME,GPL10588,MBA_4794,J8 P9 Neural Basal polylysine N2,,

iPSC,BIOTIME,GPL10588,MBA_4795,J8 P9 Neural Basal polylysine B27,,

iPSC,BIOTIME,GPL10588,MBA_4796,E68 P18 Neural Basal polylysine N2,,

iPSC,BIOTIME,GPL10588,MBA_4797,E68 P18 Neural Basal polylysine B27,,

iPSC,BIOTIME,GPL10588,MBA_4798,E111 P18 Neural Basal polylysine N2,,

iPSC,BIOTIME,GPL10588,MBA_4799,E111 P18 Neural Basal polylysine B27,,

iPSC,BIOTIME,GPL10588,MBA_4801,E33 P22 D21 Hystem BMP4 10ng/ml + TGFb3 10ng/ml,,

iPSC,BIOTIME,GPL10588,MBA_4802,E33 P22 D21 Hystem BMP4 10ng/ml ,,

iPSC,BIOTIME,GPL10588,MBA_4803,E33 P22 D21 Hystem BMP2 50ng/ml + TGFb3 10ng/ml,,

iPSC,BIOTIME,GPL10588,MBA_4804,E33 P22 D21 Hystem RA 1uM,,

iPSC,BIOTIME,GPL10588,MBA_4805,E33 P22 D14 confluent adipose,,

iPSC,BIOTIME,GPL10588,MBA_4806,E33 P22 D21 confluent DKK1 100ng/ml,,

iPSC,BIOTIME,GPL10588,MBA_4808,T20 P13 D21 Hystem BMP4 10ng/ml,,

iPSC,BIOTIME,GPL10588,MBA_4809,T20 P13 D21 Hystem BMP4 10ng/ml + TGFb3 10ng/ml,,

iPSC,BIOTIME,GPL10588,MBA_4810,T20 P13 D21 Hystem RA 1uM,,

iPSC,BIOTIME,GPL10588,MBA_4811,T20 P13 D21 Hystem BMP2 50ng/ml + TGFb3 10ng/ml,,

iPSC,BIOTIME,GPL10588,MBA_4812,T20 P13 D21 Hystem Osteo,,

iPSC,BIOTIME,GPL10588,MBA_4813,T20 P13 D14 adipose,,

iPSC,BIOTIME,GPL10588,MBA_4814,SK50 P19 D21 Hystem BMP4 10ng/ml + TGFb3 10ng/ml,,

iPSC,BIOTIME,GPL10588,MBA_4815,SK50 P19 D21 Hystem BMP2 50ng/ml + TGFb3 10ng/ml,,

iPSC,BIOTIME,GPL10588,MBA_4816,SK50 P19 D14 confluent adipose,,

iPSC,BIOTIME,GPL10588,MBA_4818,SK25 P13 D21 Hystem BMP4 10ng/ml + TGFb3 10ng/ml,,

iPSC,BIOTIME,GPL10588,MBA_4819,SK25 P13 D21 Hystem BMP4 10ng/ml ,,

iPSC,BIOTIME,GPL10588,MBA_4820,SK25 P13 D21 Hystem BMP2 50ng/ml + TGFb3 10ng/ml,,

iPSC,BIOTIME,GPL10588,MBA_4821,SK25 P13 D14 confluent adipose,,

iPSC,BIOTIME,GPL10588,MBA_4823,F15 P18 D21 Hystem BMP4 10ng/ml,,

iPSC,BIOTIME,GPL10588,MBA_4824,F15 P18 D21 Hystem BMP4 10ng/ml + TGFb3 10ng/ml,,

iPSC,BIOTIME,GPL10588,MBA_4825,F15 P18 D21 Hystem RA 1uM,,

iPSC,BIOTIME,GPL10588,MBA_4826,F15 P18 D21 Hystem BMP2 50ng/ml + TGFb3 10ng/ml,,

iPSC,BIOTIME,GPL10588,MBA_4827,F15 P18 D21 Hystem osteo,,

iPSC,BIOTIME,GPL10588,MBA_4828,F15 P18 D14 confluence adipose,,

iPSC,BIOTIME,GPL10588,MBA_4829,T42 P19 D21 Hystem BMP4 10ng/ml,,

iPSC,BIOTIME,GPL10588,MBA_4830,T42 P19 D21 Hystem BMP4 10ng/ml + TGFb3 10ng/ml,,

iPSC,BIOTIME,GPL10588,MBA_4831,T42 P19 D21 Hystem RA 1uM,,

iPSC,BIOTIME,GPL10588,MBA_4832,T42 P19 D21 Hystem BMP2 50ng/ml + TGFb3 10ng/ml,,

iPSC,BIOTIME,GPL10588,MBA_4833,T42 P19 D21Hystem osteo,,

iPSC,BIOTIME,GPL10588,MBA_4834,T42 P19 D14 confluence adipose,,

iPSC,BIOTIME,GPL10588,MBA_4836,E44 P19 D21 Hystem BMP4 10ng/ml,,

iPSC,BIOTIME,GPL10588,MBA_4837,E44 P19 D21 Hystem BMP4 10ng/ml + TGFb3 10ng/ml,,

iPSC,BIOTIME,GPL10588,MBA_4838,E44 P19 D21 Hystem RA 1uM,,

iPSC,BIOTIME,GPL10588,MBA_4839,E44 P19 D21 Hystem BMP2 50ng/ml + TGFb3 10ng/ml,,

iPSC,BIOTIME,GPL10588,MBA_4840,E44 P19 D21 Hystem osteo,,

iPSC,BIOTIME,GPL10588,MBA_4841,E44 P19 D14 confluent adipose,,

iPSC,BIOTIME,GPL10588,MBA_4843,T7 P13 D21 Hystem BMP4 10ng/ml,,

iPSC,BIOTIME,GPL10588,MBA_4844,T7 P13 D21 Hystem BMP4 10ng/ml + TGFb3 10ng/ml,,

iPSC,BIOTIME,GPL10588,MBA_4845,T7 P13 D21 Hystem RA 1uM,,

iPSC,BIOTIME,GPL10588,MBA_4846,T7 P13 D21 Hystem BMP2 50ng/ml + TGFb3 10ng/ml,,

iPSC,BIOTIME,GPL10588,MBA_4847,T7 P13 D21 Hystem osteo,,

iPSC,BIOTIME,GPL10588,MBA_4848,T7 P13 D14 confluent adipose,,

iPSC,BIOTIME,GPL10588,MBA_4850,SK44 P15 D21 Hystem BMP4 10ng/ml + TGFb3 10ng/ml,,

iPSC,BIOTIME,GPL10588,MBA_4851,SK44 P15 D21 Hystem BMP4 10ng/ml ,,

iPSC,BIOTIME,GPL10588,MBA_4852,SK44 P15 D21 Hystem BMP2 50ng/ml + TGFb3 10ng/ml,,

iPSC,BIOTIME,GPL10588,MBA_4853,SK44 P15 D21 Hystem RA 1uM,,

iPSC,BIOTIME,GPL10588,MBA_4854,SK44 P15 D21 Hystem osteo,,

iPSC,BIOTIME,GPL10588,MBA_4855,SK44 P15 D14 confluent adipose,,

iPSC,BIOTIME,GPL10588,MBA_4857,RAPEND18 P13 D21 Hystem BMP4 10ng/ml,,

iPSC,BIOTIME,GPL10588,MBA_4858,RAPEND18 P13 D21 Hystem BMP4 10ng/ml + TGFb3 10 ng/ml,,

iPSC,BIOTIME,GPL10588,MBA_4859,RAPEND18 P13 D21 Hystem RA 1uM,,

iPSC,BIOTIME,GPL10588,MBA_4860,RAPEND18 P13 D21 Hystem BMP2 50ng/ml + TGFb3 10 ng/ml,,

iPSC,BIOTIME,GPL10588,MBA_4861,RAPEND18 P13 D21 Hystem osteo,,

iPSC,BIOTIME,GPL10588,MBA_4862,RAPEND18 P13 D14 confluence adipo,,

iPSC,BIOTIME,GPL10588,MBA_4864,SK11 P16 D21 Hystem BMP4 10ng/ml,,

iPSC,BIOTIME,GPL10588,MBA_4865,SK11 P16 D21 Hystem BMP4 10ng/ml + TGFb3 10ng/ml,,

iPSC,BIOTIME,GPL10588,MBA_4866,SK11 P16 D21 Hystem RA 1uM,,

iPSC,BIOTIME,GPL10588,MBA_4867,SK11 P16 D21 Hystem BMP2 50ng/ml + TGFb3 10ng/ml,,

iPSC,BIOTIME,GPL10588,MBA_4868,SK11 P16 D21 Hystem osteo,,

iPSC,BIOTIME,GPL10588,MBA_4869,SK11 P16 D14 confluence adipose,,

iPSC,BIOTIME,GPL10588,MBA_4870,SK11 P15 D21 MM TGFb3 10ng/ml,,

iPSC,BIOTIME,GPL10588,MBA_4871,SK11 P15 D21 MM BMP4 10ng/ml + TGFb3 10ng/ml,,

iPSC,BIOTIME,GPL10588,MBA_4872,SK11 P15 D21 MM TGFb3 10ng/ml humanzyme,,

iPSC,BIOTIME,GPL10588,MBA_4873,SK11 P15 D21 MM BMP4 10ng/ml + TGFb3 10ng/ml humanzyme,,

iPSC,BIOTIME,GPL10588,MBA_4874,W8 P13 D21 confluent DKK1 100ng/ml,,

iPSC,BIOTIME,GPL10588,MBA_4876,MW1 P15 D21 Hystem BMP4 10ng/ml,,

iPSC,BIOTIME,GPL10588,MBA_4877,MW1 P15 D21 Hystem BMP4 10ng/ml + TGFb3 10ng/ml,,

iPSC,BIOTIME,GPL10588,MBA_4878,MW1 P15 D21 Hystem RA 1uM,,

iPSC,BIOTIME,GPL10588,MBA_4879,MW1 P15 D21 Hystem BMP2 50ng/ml + TGFb3 10ng/ml,,

iPSC,BIOTIME,GPL10588,MBA_4880,MW1 P15 D21 Hystem osteo,,

iPSC,BIOTIME,GPL10588,MBA_4881,MW1 P15 D14 confluence adipose,,

iPSC,BIOTIME,GPL10588,MBA_4883,E75 P11 D14 confluence adipose,,

iPSC,BIOTIME,GPL10588,MBA_4884,E75 P11 D21 Hystem BMP4 10ng/ml,,

iPSC,BIOTIME,GPL10588,MBA_4885,E75 P11 D21 Hystem BMP4 10ng/ml + TGFb3 10ng/ml,,

iPSC,BIOTIME,GPL10588,MBA_4886,E75 P11 D21 Hystem RA 1uM,,

iPSC,BIOTIME,GPL10588,MBA_4887,E75 P11 D21 Hystem BMP2 50ng/ml + TGFb3 10ng/ml,,

iPSC,BIOTIME,GPL10588,MBA_4888,E75 P11 D21 Hystem osteo,,

iPSC,BIOTIME,GPL10588,MBA_4890,RAPEND15 P14 D21 BMP4 10ng/ml,,

iPSC,BIOTIME,GPL10588,MBA_4891,RAPEND15 P14 D21 BMP4 10ng/ml + TGFb3 10ng/ml,,

iPSC,BIOTIME,GPL10588,MBA_4892,RAPEND15 P14 D21 Hystem RA 1uM,,

iPSC,BIOTIME,GPL10588,MBA_4893,RAPEND15 P14 D21 BMP2 50ng/ml + TGFb3 10ng/ml,,

iPSC,BIOTIME,GPL10588,MBA_4894,RAPEND15 P14 D21 Hystem osteo,,

iPSC,BIOTIME,GPL10588,MBA_4895,RAPEND15 P14 D14 confluence adipose,,

iPSC,BIOTIME,GPL10588,MBA_4897,SK5 P12 D21 Hystem BMP4 10ng/ml,,

iPSC,BIOTIME,GPL10588,MBA_4898,SK5 P12 D21 Hystem BMP4 10ng/ml + TGFb3 10ng/ml,,

iPSC,BIOTIME,GPL10588,MBA_4899,SK5 P12 D21 Hystem RA 1uM,,

iPSC,BIOTIME,GPL10588,MBA_4900,SK5 P12 D21 Hystem BMP2 50ng/ml + TGFb3 10ng/ml,,

iPSC,BIOTIME,GPL10588,MBA_4901,SK5 P12 D21 Hystem osteo,,

iPSC,BIOTIME,GPL10588,MBA_4902,SK5 P12 D14 confluence adipose,,

iPSC,BIOTIME,GPL10588,MBA_4904,E72 P11 D21 Hystem BMP4 10ng/ml,,

iPSC,BIOTIME,GPL10588,MBA_4905,E72 P11 D21 Hystem BMP4 10ng/ml + TGFb3 10ng/ml,,

iPSC,BIOTIME,GPL10588,MBA_4906,E72 P11 D21 Hystem RA 1uM,,

iPSC,BIOTIME,GPL10588,MBA_4907,E72 P11 D21 Hystem BMP2 50ng/ml + TGFb3 10ng/ml,,

iPSC,BIOTIME,GPL10588,MBA_4908,E72 P11 D21 Hystem osteo,,

iPSC,BIOTIME,GPL10588,MBA_4909,E72 P11 D14 confluence adipose,,

iPSC,BIOTIME,GPL10588,MBA_4910,RAPEND10 P12 D14 confluence adipose,,

iPSC,BIOTIME,GPL10588,MBA_4911,RAPEND10 P12 D21 Hystem BMP4 10mg/ml +TGFb3 10ng/ml,,

iPSC,BIOTIME,GPL10588,MBA_4912,RAPEND10 P12 D21 Hystem BMP4 10mg/ml ,,

iPSC,BIOTIME,GPL10588,MBA_4913,RAPEND10 P12 D21 Hystem BMP2 50mg/ml +TGFb3 10ng/ml,,

iPSC,BIOTIME,GPL10588,MBA_4914,RAPEND10 P12 D21 Hystem RA 1uM,,

iPSC,BIOTIME,GPL10588,MBA_4915,RAPEND10 P12 D21 Hystem osteo,,

iPSC,BIOTIME,GPL10588,MBA_4917,SM30 P16 D21 Hystem BMP4 10ng/ml,,

iPSC,BIOTIME,GPL10588,MBA_4918,SM30 P16 D21 Hystem BMP4 10ng/ml + TGFb3 10ng/ml,,

iPSC,BIOTIME,GPL10588,MBA_4919,SM30 P16 D21 Hystem RA 1uM,,

iPSC,BIOTIME,GPL10588,MBA_4920,SM30 P16 D21 Hystem osteo,,

iPSC,BIOTIME,GPL10588,MBA_4921,SM30 P16 D14 confluence adipo,,

iPSC,BIOTIME,GPL10588,MBA_4922,SM30 P18 D7 alg BMP4 10ng/ml + TGFb3 10ng/ml,,

iPSC,BIOTIME,GPL10588,MBA_4923,SM30 P18 D14 alg BMP4 10ng/ml + TGFb3 10ng/ml,,

iPSC,BIOTIME,GPL10588,MBA_4924,SM30 P18 alg D21 BMP4 10ng/ml + TGFb3 10ng/ml,,

iPSC,BIOTIME,GPL10588,MBA_4925,E15 P18 D14 alg BMP4 10ng/ml + TGFb3 10ng/ml,,

iPSC,BIOTIME,GPL10588,MBA_4926,E15 P18 D21 alg BMP4 10ng/ml + TGFb3 10ng/ml,,

iPSC,BIOTIME,GPL10588,MBA_4927,Koichi Bovine Tissue time zero,,

iPSC,BIOTIME,GPL10588,MBA_4928,"Koichi Bovine Tissue + ES cells 200,000 time zero",,

iPSC,BIOTIME,GPL10588,MBA_4929,Koichi Bovine Tissue + Xgene cells 1x10e6 time zero,,

iPSC,BIOTIME,GPL10588,MBA_4930,Koichi Bovine Tissue time 72 hours,,

iPSC,BIOTIME,GPL10588,MBA_4931,"Koichi Bovine Tissue + ES cells 200,000 time 72 hours",,

iPSC,BIOTIME,GPL10588,MBA_4932,Koichi Bovine Tissue + Xgene cells 1x10e6 time 72 hours,,

iPSC,BIOTIME,GPL10588,MBA_4933,"Koichi Rabbit disc + Xgene pellet 200,000 time 72 hours",,

iPSC,BIOTIME,GPL10588,MBA_4934,"Koichi Rabbit disc + Xgene 200,000 in lactated ringers time 72 hours",,

iPSC,BIOTIME,GPL10588,MBA_4935,"Koichi Rabbit disc + Xgene Hystemt 200,000 time 72 hours",,

AC,BIOTIME,GPL10588,MBA_4936,Xgene FB Koichi,,

iPSC,BIOTIME,GPL10588,MBA_4945,4D20.9 P12 D14 confluence adipo,,

iPSC,BIOTIME,GPL10588,MBA_4946,4D20.9 P12 D21 Hystem BMP4 10ng/ml,,

iPSC,BIOTIME,GPL10588,MBA_4947,4D20.9 P12 D21 Hystem BMP4 10ng/ml + TGFb3 10ng/ml,,

iPSC,BIOTIME,GPL10588,MBA_4948,4D20.9 P12 D21 Hystem RA 1uM looked strange on microarray,,

iPSC,BIOTIME,GPL10588,MBA_4949,4D20.9 P12 D21 Hystem BMP2 50ng/ml + TGFb3 10ng/ml,,

iPSC,BIOTIME,GPL10588,MBA_4950,4D20.9 P12 D21 Hystem osteo,,

iPSC,BIOTIME,GPL10588,MBA_4952,E69 P14 D14 Hystem TGFb3 10ng/ml,,

iPSC,BIOTIME,GPL10588,MBA_4953,E69 P14 D14 Hystem BMP4 10ng/ml ,,

iPSC,BIOTIME,GPL10588,MBA_4954,E69 P14 D14 Hystem BMP4 10ng/ml + TGFb3 10ng/ml,,

iPSC,BIOTIME,GPL10588,MBA_4954,E69 P14 D14 Hystem BMP4 10ng/ml + TGFb3 10ng/ml,,

iPSC,BIOTIME,GPL10588,MBA_4956,T42 P17 D14 Hystem TGFb3 10ng/ml,,

iPSC,BIOTIME,GPL10588,MBA_4957,T42 P17 D14 Hystem BMP4 10ng/ml,,

iPSC,BIOTIME,GPL10588,MBA_4957,T42 P17 D14 Hystem BMP4 10ng/ml,,

iPSC,BIOTIME,GPL10588,MBA_4958,T42 P17 D14 Hystem BMP4 10ng/ml + TGFb3 10ng/ml,,

iPSC,BIOTIME,GPL10588,MBA_4958 ,T42 P17 D14 Hystem BMP4 10ng/ml + TGFb3 10ng/ml,,

iPSC,BIOTIME,GPL10588,MBA_4960,E19 P11 D14 confluence adipo,,

iPSC,BIOTIME,GPL10588,MBA_4961,E19 P11 D21 Hystem BMP4 10ng/ml,,

iPSC,BIOTIME,GPL10588,MBA_4962,E19 P11 D21 Hystem BMP4 10ng/ml + TGFb3 10ng/ml,,

iPSC,BIOTIME,GPL10588,MBA_4963,E19 P11 D21 Hystem RA 1uM,,

iPSC,BIOTIME,GPL10588,MBA_4964,E19 P11 D21 Hystem BMP2 50ng/ml + TGFb3 10ng/ml,,

iPSC,BIOTIME,GPL10588,MBA_4965,E19 P11 D21 Hystem osteo,,

iPSC,BIOTIME,GPL10588,MBA_4967,E68 P17 D14 Hystem TGFb3 10ng/ml,,

iPSC,BIOTIME,GPL10588,MBA_4968,E68 P17 D14 Hystem BMP4 10ng/ml,,

iPSC,BIOTIME,GPL10588,MBA_4969,E68 P17 D14 Hystem BMP4 10ng/ml + TGFb3 10ng/ml,,

iPSC,BIOTIME,GPL10588,MBA_4971,T14 P11 D21 Hystem BMP4 10ng/ml,,

iPSC,BIOTIME,GPL10588,MBA_4972,T14 P11 D21 Hystem BMP4 10ng/ml + TGFb3 10ng/ml,,

iPSC,BIOTIME,GPL10588,MBA_4973,T14 P11 D21 Hystem RA 1uM,,

iPSC,BIOTIME,GPL10588,MBA_4974,T14 P11 D21 Hystem BMP2 50ng/ml + TGFb3 10ng/ml,,

iPSC,BIOTIME,GPL10588,MBA_4975,T14 P11 D21 Hystem osteo,,

iPSC,BIOTIME,GPL10588,MBA_4976,T14 P11 confluence adipo,,

iPSC,BIOTIME,GPL10588,MBA_4977,4D20.8 P18 D14 Monolayer BMP4 10ng/ml + TGFb3 10ng/ml intact,,

iPSC,BIOTIME,GPL10588,MBA_4978,"4D20.8 P18 D14 Monolayer BMP410ng/ml + TGFb3 10ng/ml disocciated to single cells with trypsin, deactivated, spun, and lysed",,

iPSC,BIOTIME,GPL10588,MBA_4979,4D20.8 P18 D21 monolayer BMP4 10ng/ml + TGFb3 10ng/ml intact,,

iPSC,BIOTIME,GPL10588,MBA_4980,"4D20.8 P18 D21 monolayer BMP4 10ng/ml + TGFb3 10ng/ml dissociated to single cells with trypsin, deactivated, spun, and lysed",,

iPSC,BIOTIME,GPL10588,MBA_4982,EN5 P12 D14 confluence adipo,,

iPSC,BIOTIME,GPL10588,MBA_4983,EN5 P12 D21 Hystem BMP4 10ng/ml,,

iPSC,BIOTIME,GPL10588,MBA_4984,EN5 P12 D21 Hystem BMP4 10ng/ml + TGFb3 10ng/ml,,

iPSC,BIOTIME,GPL10588,MBA_4985,EN5 P12 D21 Hystem RA 1uM,,

iPSC,BIOTIME,GPL10588,MBA_4986,EN5 P12 D21 Hystem BMP2 50ng/ml + TGFb3 10ng/ml,,

iPSC,BIOTIME,GPL10588,MBA_4987,EN5 P12 D21 Hystem osteo,,

iPSC,BIOTIME,GPL10588,MBA_4989,EN26 P10 D14 confluence adipose,,

iPSC,BIOTIME,GPL10588,MBA_4990,EN26 P10 D21 Hystem BMP4 10ng/ml,,

iPSC,BIOTIME,GPL10588,MBA_4991,EN26 P10 D21 Hystem BMP4 10ng/ml + TGFb3 10ng/ml,,

iPSC,BIOTIME,GPL10588,MBA_4992,EN26 P10 D21 Hystem RA 1uM,,

iPSC,BIOTIME,GPL10588,MBA_4993,EN26 P10 D21 Hystem BMP2 50ng/ml + TGFb3 10ng/ml,,

iPSC,BIOTIME,GPL10588,MBA_4994,EN26 P10 D21 Hystem osteo,,

iPSC,BIOTIME,GPL10588,MBA_4996,T43 P13 D14 confluence adipo,,

iPSC,BIOTIME,GPL10588,MBA_4997,T43 P13 D21 Hystem BMP4 10ng/ml,,

iPSC,BIOTIME,GPL10588,MBA_4998,T43 P13 D21 Hystem BMP4 10ng/ml + TGFb3 10ng/ml,,

iPSC,BIOTIME,GPL10588,MBA_4999,T43 P13 D21 Hystem RA 1uM,,

iPSC,BIOTIME,GPL10588,MBA_5000,T43 P13 D21 Hystem BMP2 50ng/ml + TGFb3 10ng/ml,,

iPSC,BIOTIME,GPL10588,MBA_5001,T43 P13 D21 Hystem osteo,,

iPSC,BIOTIME,GPL10588,MBA_5003,7PEND30 P13 D21 Hystem BMP4 10ng/ml,,

iPSC,BIOTIME,GPL10588,MBA_5004,7PEND30 P13 D21 Hystem BMP4 10ng/ml + TGFb3 10ng/ml,,

iPSC,BIOTIME,GPL10588,MBA_5005,7PEND30 P13 D21 Hystem RA 1uM,,

iPSC,BIOTIME,GPL10588,MBA_5006,7PEND30 P13 D21 Hystem BMP2 50ng/ml + TGFb3 10ng/ml,,

iPSC,BIOTIME,GPL10588,MBA_5007,7PEND30 P13 D21 Hystem osteo,,

iPSC,BIOTIME,GPL10588,MBA_5008,7PEND30 P13 D14 confluence adipo,,

iPSC,BIOTIME,GPL10588,MBA_5010,4D20.3 P10 D14 confluence adipo,,

iPSC,BIOTIME,GPL10588,MBA_5011,4D20.3 P10 D21 Hystem BMP4 10ng/ml,,

iPSC,BIOTIME,GPL10588,MBA_5012,4D20.3 P10 D21 Hystem BMP4 10ng/ml + TGFb3 10ng/ml,,

iPSC,BIOTIME,GPL10588,MBA_5013,4D20.3 P10 D21 Hystem RA 1uM,,

iPSC,BIOTIME,GPL10588,MBA_5014,4D20.3 P10 D21 Hystem BMP2 50ng/ml + TGFb3 10ng/ml,,

iPSC,BIOTIME,GPL10588,MBA_5015,4D20.3 P10 D21 Hystem osteo,,

iPSC,BIOTIME,GPL10588,MBA_5017,U18 P12 D14 confluence adipo,,

iPSC,BIOTIME,GPL10588,MBA_5018,U18 P12 D21 Hystem BMP4 10ng/ml,,

iPSC,BIOTIME,GPL10588,MBA_5019,U18 P12 D21 Hystem BMP4 10ng/ml + TGFb3 10ng/ml,,

iPSC,BIOTIME,GPL10588,MBA_5020,U18 P12 D21 Hystem RA 1uM,,

iPSC,BIOTIME,GPL10588,MBA_5021,U18 P12 D21 Hystem BMP2 50ng/ml + TGFb3 10ng/ml,,

iPSC,BIOTIME,GPL10588,MBA_5022,U18 P12 D21 Hystem osteo,,

iPSC,BIOTIME,GPL10588,MBA_5024,T44 P11 D14 confluence adipo,,

iPSC,BIOTIME,GPL10588,MBA_5025,T44 P14 D21 Hystem BMP4 10ng/ml + TGFb3 10ng/ml,,

iPSC,BIOTIME,GPL10588,MBA_5026,T44 P14 D21 Hystem BMP4 10ng/ml ,,

iPSC,BIOTIME,GPL10588,MBA_5027,T44 P14 D21 Hystem BMP2 20ng/ml + TGFb3 10ng/ml,,

iPSC,BIOTIME,GPL10588,MBA_5028,T44 P14 D21 Hystem RA 1uM,,

iPSC,BIOTIME,GPL10588,MBA_5029,T44 P14 D21 Hystem osteo,,

iPSC,BIOTIME,GPL10588,MBA_5031,RAD20.5 P16 D14 confluence adipo,,

iPSC,BIOTIME,GPL10588,MBA_5032,RAD20.5 P16 D21 Hystem BMP4 10ng/ml,,

iPSC,BIOTIME,GPL10588,MBA_5033,RAD20.5 P16 D21 Hystem BMP4 10ng/ml + TGFb3 10ng/ml,,

iPSC,BIOTIME,GPL10588,MBA_5034,RAD20.5 P16 D21 Hystem RA 1uM,,

iPSC,BIOTIME,GPL10588,MBA_5035,RAD20.5 P16 D21 Hystem BMP2 50ng/ml + TGFb3 10ng/ml,,

iPSC,BIOTIME,GPL10588,MBA_5036,RAD20.5 P16 D21 Hystem osteo,,

iPSC,BIOTIME,GPL10588,MBA_5038,T42 P19 D14 MM SCF 10ng/ml,,

iPSC,BIOTIME,GPL10588,MBA_5039,T42 P19 D14 MM SCF 10ng/ml + BMP4 10ng/ml,,

iPSC,BIOTIME,GPL10588,MBA_5040,T42 P19 D14 Hystem SCF 10ng/ml,,

iPSC,BIOTIME,GPL10588,MBA_5041,T42 P19 D14 Hystem SCF 10ng/ml +BMP4 10ng/ml,,

AC,BIOTIME,GPL10588,MBA_5043,hMSC P6 D14 MM-incomlpete only ,,

AC,BIOTIME,GPL10588,MBA_5044,hMSC P6 D14 MM RA 1uM,,

AC,BIOTIME,GPL10588,MBA_5045,hMSC P6 D14 MM SCF 10ng/ml,,

AC,BIOTIME,GPL10588,MBA_5046,hMSC P6 D14 MM SCF 10ng/ml + BMP4 10ng/ml,,

iPSC,BIOTIME,GPL10588,MBA_5047,SK17 P14 3Days Neurobasal,,

iPSC,BIOTIME,GPL10588,MBA_5048,RP1-SKEL8 P16 D3 Neurobasal medium 3D,,

iPSC,BIOTIME,GPL10588,MBA_5049,ESI-RP1-SK153 P7 D6 Neurobasal medium,,

iPSC,BIOTIME,GPL10588,MBA_5051,RAD20.16 P13 D14 confluence adipo,,

iPSC,BIOTIME,GPL10588,MBA_5052,RAD20.16 P13 D21 Hystem BMP4 10ng/ml,,

iPSC,BIOTIME,GPL10588,MBA_5053,RAD20.16 P13 D21 Hystem BMP4 10ng/ml + TGFb3 10ng/ml,,

iPSC,BIOTIME,GPL10588,MBA_5054,RAD20.16 P13 D21 Hystem RA 1uM,,

iPSC,BIOTIME,GPL10588,MBA_5055,RAD20.16 P13 D21 Hystem BMP2 50ng/ml + TGFb3 10ng/ml,,

iPSC,BIOTIME,GPL10588,MBA_5056,RAD20.16 P13 D21 Hystem osteo,,

iPSC,BIOTIME,GPL10588,MBA_5058,RASMO.19 P16 D21 Hystem BMP4 10ng/ml,,

iPSC,BIOTIME,GPL10588,MBA_5059,RASMO.19 P16 D21 Hystem BMP4 10ng/ml + TGFb3 10ng/ml,,

iPSC,BIOTIME,GPL10588,MBA_5060,RASMO.19 P16 D21 Hystem RA 1uM,,

iPSC,BIOTIME,GPL10588,MBA_5061,RASMO.19 P16 D21 Hystem BMP2 50ng/ml + TGFb3 10ng/ml,,

iPSC,BIOTIME,GPL10588,MBA_5062,RASMO.19 P16 D21 Hystem osteo,,

iPSC,BIOTIME,GPL10588,MBA_5064,E163 P13 D21 Hystem BMP4 10ng/ml + TGFb3 10ng/ml,,

iPSC,BIOTIME,GPL10588,MBA_5065,E163 P13 D21 Hystem BMP4 10ng/ml ,,

iPSC,BIOTIME,GPL10588,MBA_5066,E163 P13 D21 Hystem BMP2 50ng/ml + TGFb3 10ng/ml,,

iPSC,BIOTIME,GPL10588,MBA_5067,E163 P13 D21 Hystem RA 1uM,,

iPSC,BIOTIME,GPL10588,MBA_5068,E163 P13 D21 Hystem osteo,,

iPSC,BIOTIME,GPL10588,MBA_5069,RASMO12 P16 D14 confluence adipo,,

iPSC,BIOTIME,GPL10588,MBA_5070,RASMO19 P16 D14 confluence adipo,,

iPSC,BIOTIME,GPL10588,MBA_5071,E163 P13 D14 confluence adipo,,

iPSC,BIOTIME,GPL10588,MBA_5072,RASMO12 P16 D21 Hystem BMP4 10ng/ml,,

iPSC,BIOTIME,GPL10588,MBA_5073,RASMO12 P16 D21 Hystem BMP410ng/ml + TGFb3 10ng/ml,,

iPSC,BIOTIME,GPL10588,MBA_5074,RASMO12 P16 D21 Hystem RA 1uM,,

iPSC,BIOTIME,GPL10588,MBA_5075,RASMO12 P16 D21 Hystem BMP2 50ng/ml + TGFb3 10ng/ml,,

iPSC,BIOTIME,GPL10588,MBA_5076,RASMO12 P16 D21 Hystem osteo,,

iPSC,BIOTIME,GPL10588,MBA_5078,7PEND11 P11 D14 confluence adipo ,,

iPSC,BIOTIME,GPL10588,MBA_5079,7PEND11 P11 D21 Hystem BMP4 10ng/ml ,,

iPSC,BIOTIME,GPL10588,MBA_5080,7PEND11 P11 D21 Hystem BMP4 10ng/ml + TGFb3 10ng/ml,,

iPSC,BIOTIME,GPL10588,MBA_5081,7PEND11 P11 D21 Hystem RA 1uM,,

iPSC,BIOTIME,GPL10588,MBA_5082,7PEND11 P11 D21 Hystem BMP2 50ng/ml + TGFb3 10ng/ml,,

iPSC,BIOTIME,GPL10588,MBA_5083,7PEND11 P11 D21 Hystem osteo,,

iPSC,BIOTIME,GPL10588,MBA_5085,7SMOO25 P12 confluence adipose,,

iPSC,BIOTIME,GPL10588,MBA_5086,7SMOO25 P12 D21 Hystem BMP4 10ng/ml + TGFb3 10ng/ml,,

iPSC,BIOTIME,GPL10588,MBA_5087,7SMOO25 P12 D21 Hystem BMP4 10ng/ml ,,

iPSC,BIOTIME,GPL10588,MBA_5088,7SMOO25 P12 D21 Hystem BMP2 50ng/ml + TGFb3 10ng/ml,,

iPSC,BIOTIME,GPL10588,MBA_5089,7SMOO25 P12 D21 Hystem RA 1uM,,

iPSC,BIOTIME,GPL10588,MBA_5090,7SMOO25 P12 D21 Hystem osteo,,

iPSC,BIOTIME,GPL10588,MBA_5092,E69 P16 D21 Hystem SCF 10ng/ml,,

iPSC,BIOTIME,GPL10588,MBA_5093,E69 P16 D21 Hystem SCF 10ng/ml + BMP4 10ng/ml,,

iPSC,BIOTIME,GPL10588,MBA_5094,E69 P16 D14 MM SCF 10ng/ml,,

iPSC,BIOTIME,GPL10588,MBA_5095,E69 P16 D14 MM SCF 10ng/ml + BMP4 10ng/ml,,

iPSC,BIOTIME,GPL10588,MBA_5097,E68 P19 D14 MM SCF 10ng/ml,,

iPSC,BIOTIME,GPL10588,MBA_5098,E68 P19 D14 MM SCF 10ng/ml + BMP4 10ng/ml,,

iPSC,BIOTIME,GPL10588,MBA_5099,E68 P19 D14 Hystem SCF 10ng/ml ,,

iPSC,BIOTIME,GPL10588,MBA_5100,E68 P19 D14 Hystem SCF 10ng/ml + BMP4 10ng/ml,,

AC,BIOTIME,GPL10588,MBA_5101,hMSC P6 D21 confluence Osteo,,

AC,BIOTIME,GPL10588,MBA_5102,hMSC P6 D21 hystem-TCP lyophilized Osteo,,

iPSC,BIOTIME,GPL10588,MBA_5103,SM30 P18 D21 confluence Osteo ,,

iPSC,BIOTIME,GPL10588,MBA_5104,SM30 P18 D21 hystem-TCP lysophilized Osteo,,

iPSC,BIOTIME,GPL10588,MBA_5105,SK11 P17 D21 confluence Osteo,,

iPSC,BIOTIME,GPL10588,MBA_5106,SK11 P17 D21 hystem-TCP lyophilized Osteo,,

iPSC,BIOTIME,GPL10588,MBA_5107,SK11 P17 D21 Hystem lyophilized Osteo,,

iPSC,BIOTIME,GPL10588,MBA_5108,4D20.8 P22 D14 MM SCF 10ng/ml,,

iPSC,BIOTIME,GPL10588,MBA_5109,4D20.8 P22 D14 MM SCF 10ng/ml + BMP4 10ng/ml,,

iPSC,BIOTIME,GPL10588,MBA_5110,4D20.8 P22 D14 Hystem SCF 10ng/ml,,

iPSC,BIOTIME,GPL10588,MBA_5111,4D20.8 P22 D14 Hystem SCF 10ng/ml + BMP4 10ng/ml,,

iPSC,BIOTIME,GPL10588,MBA_5112,"4D20.9 P13 D14 MethoCult MC-cytokine, 100,000 cell/well",,

iPSC,BIOTIME,GPL10588,MBA_5113,"4D20.9 P13 D14 MethoCult MC-cytokine, 20,000 cell/well",,

iPSC,BIOTIME,GPL10588,MBA_5114,"4D20.9 P13 D14 MethoCult MC-cytokine added after attach, 300,000 cell/well ",,

iPSC,BIOTIME,GPL10588,MBA_5115,"ESI-RP1-SK153 P13 D14 MethoCult MC-cytokine, 20,000 cell/well",,

iPSC,BIOTIME,GPL10588,MBA_5116,"ESI-RP1-SK153 P13 D14 MethoCult MC-cytokine, 100,000 cell/well",,

iPSC,BIOTIME,GPL10588,MBA_5117,"ESI-RP1-SK153 P13 D14 MethoCult MC-cytokine added after attach, 300,000 cell/well ",,

iPSC,BIOTIME,GPL10588,MBA_5134,SK11 P20 + SM30 P17 ctrl,,

iPSC,BIOTIME,GPL10588,MBA_5135,SK11 P20 + SM30 P17 D21 Hystem BMP4 10ng/ml,,

iPSC,BIOTIME,GPL10588,MBA_5136,SK11 P20 + SM30 P17 D21 Hystem BMP4 10ng/ml + TGFb3 10ng/ml,,

iPSC,BIOTIME,GPL10588,MBA_5137,SK11 P20 + SM30 P17 D21 Hystem BMP2 50ng/ml + TGFb3 10ng/ml,,

iPSC,BIOTIME,GPL10588,MBA_5138,SK11 P20 + SM30 P17 D21 Hystem osteo,,

iPSC,BIOTIME,GPL10588,MBA_5140,EN1 P15 D14 confluence adipo,,

iPSC,BIOTIME,GPL10588,MBA_5141,EN1 P15 D21 Hystem BMP4 10ng/ml,,

iPSC,BIOTIME,GPL10588,MBA_5142,EN1 P15 D21 Hystem BMP4 10ng/ml + TGFb3 10ng/ml,,

iPSC,BIOTIME,GPL10588,MBA_5143,EN1 P15 D21 Hystem RA 1uM,,

iPSC,BIOTIME,GPL10588,MBA_5144,EN1 P15 D21 Hystem BMP2 50ng/ml + TGFb3 10ng/ml,,

iPSC,BIOTIME,GPL10588,MBA_5145,EN1 P15 D21 Hystem osteo,,

iPSC,BIOTIME,GPL10588,MBA_5147,RASMO12 P16 D14 confluence adipo,,

iPSC,BIOTIME,GPL10588,MBA_5148,RASMO12 P16 D21 Hystem BMP4 10ng/ml,,

iPSC,BIOTIME,GPL10588,MBA_5149,RASMO12 P16 D21 Hystem BMP410ng/ml + TGFb3 10ng/ml,,

iPSC,BIOTIME,GPL10588,MBA_5150,RASMO12 P16 D21 Hystem RA 1uM,,

iPSC,BIOTIME,GPL10588,MBA_5151,RASMO12 P16 D21 Hystem BMP2 50ng/ml + TGFb3 10ng/ml,,

iPSC,BIOTIME,GPL10588,MBA_5152,RASMO12 P16 D21 Hystem osteo,,

iPSC,BIOTIME,GPL10588,MBA_5154,7SMOO12 P11 confluence adipo,,

iPSC,BIOTIME,GPL10588,MBA_5155,7SMOO12 P11 D21 Hystem BMP4 10ng/ml + TGFb3 10ng/ml,,

iPSC,BIOTIME,GPL10588,MBA_5156,7SMOO12 P11 D21 Hystem BMP4 10ng/ml,,

iPSC,BIOTIME,GPL10588,MBA_5157,7SMOO12 P11 D21 Hystem BMP2 50ng/ml + TGFb3 10ng/ml,,

iPSC,BIOTIME,GPL10588,MBA_5158,7SMOO12 P11 D21 Hystem RA 1uM,,

iPSC,BIOTIME,GPL10588,MBA_5159,7SMOO12 P11 D21 Hystem osteo,,

iPSC,BIOTIME,GPL10588,MBA_5161,E169 P16 D14 confluence adipo,,

iPSC,BIOTIME,GPL10588,MBA_5162,E169 P16 D21 Hystem BMP4 10ng/ml + TGFb3 10ng/ml,,

iPSC,BIOTIME,GPL10588,MBA_5163,E169 P16 D21 Hystem BMP4 10ng/ml,,

iPSC,BIOTIME,GPL10588,MBA_5164,E169 P16 D21Hystem BMP2 50ng/ml + TGFb3 10ng/ml,,

iPSC,BIOTIME,GPL10588,MBA_5165,E169 P16 D21 Hystem RA 1uM,,

iPSC,BIOTIME,GPL10588,MBA_5166,E169 P16 D21 osteo,,

iPSC,BIOTIME,GPL10588,MBA_5168,EN55 P13 D14 confluence adipo,,

iPSC,BIOTIME,GPL10588,MBA_5169,EN55 P13 D21 Hystem BMP4 10ng/ml,,

iPSC,BIOTIME,GPL10588,MBA_5170,EN55 P13 D21 Hystem BMP4 10ng/ml + TGFb3 10ng/ml,,

iPSC,BIOTIME,GPL10588,MBA_5171,EN55 P13 D21 Hystem RA 1uM,,

iPSC,BIOTIME,GPL10588,MBA_5172,EN55 P13 D21 Hystem BMP2 50ng/ml + TGFb3 10ng/ml,,

iPSC,BIOTIME,GPL10588,MBA_5173,EN55 P13 D21 Hystem osteo,,

iPSC,BIOTIME,GPL10588,MBA_5175,EN51 P13 confluence adipo,,

iPSC,BIOTIME,GPL10588,MBA_5176,EN51 P13 D21 Hystem BMP4 10ng/ml,,

iPSC,BIOTIME,GPL10588,MBA_5177,EN51 P13 D21 Hystem BMP4 10ng/ml + TGFb3 10ng/ml,,

iPSC,BIOTIME,GPL10588,MBA_5178,EN51 P13 D21 Hystem RA 1uM,,

iPSC,BIOTIME,GPL10588,MBA_5179,EN51 P13 D21 Hystem BMP2 50ng/ml + TGFb3 10ng/ml,,

iPSC,BIOTIME,GPL10588,MBA_5180,EN51 P13 D21 Hystem osteo,,

iPSC,BIOTIME,GPL10588,MBA_5182,SM2 P12 confluence adipo,,

iPSC,BIOTIME,GPL10588,MBA_5183,SM2 P12 D21 Hystem BMP4 10ng/ml,,

iPSC,BIOTIME,GPL10588,MBA_5184,SM2 P12 D21 Hystem BMP4 10ng/ml + TGFb3 10ng/ml,,

iPSC,BIOTIME,GPL10588,MBA_5185,SM2 P12 D21 Hystem RA 1uM,,

iPSC,BIOTIME,GPL10588,MBA_5186,SM2 P12 D21 Hystem BMP2 50ng/ml + TGFb3 10ng/ml,,

iPSC,BIOTIME,GPL10588,MBA_5187,SM2 P12 D21 Hystem osteo using incomplete no serum,,

iPSC,BIOTIME,GPL10588,MBA_5197,SM33 P13 confluent adipo,,

iPSC,BIOTIME,GPL10588,MBA_5198,SM33 P13 D21 Hystem BMP4 10ng/ml,,

iPSC,BIOTIME,GPL10588,MBA_5199,SM33 P13 D21 Hystem BMP4 10ng/ml + TGFb3 10ng/ml,,

iPSC,BIOTIME,GPL10588,MBA_5200,SM33 P13 D21 Hystem RA 1uM,,

iPSC,BIOTIME,GPL10588,MBA_5201,SM33 P13 D21 Hystem BMP2 50ng/ml + TGFb3 10ng/ml,,

iPSC,BIOTIME,GPL10588,MBA_5202,SM33 P13 D21 Hystem osteo using incomplete no serum,,

iPSC,BIOTIME,GPL10588,MBA_5204,E164 P14 D14 confluence adipo,,

iPSC,BIOTIME,GPL10588,MBA_5205,E164 P14 D21 Hystem BMP4 10ng/ml + TGFb3 10ng/ml,,

iPSC,BIOTIME,GPL10588,MBA_5206,E164 P14 D21 Hystem BMP4 10ng/ml ,,

iPSC,BIOTIME,GPL10588,MBA_5207,E164 P14 D21 Hystem BMP2 50ng/ml + TGFb3 10ng/ml,,

iPSC,BIOTIME,GPL10588,MBA_5208,E164 P14 D21 Hystem RA 1uM,,

iPSC,BIOTIME,GPL10588,MBA_5209,E164 P14 D21 Hystem osteo,,

iPSC,BIOTIME,GPL10588,MBA_5211,Z2 P12 D14 confluence adipose,,

iPSC,BIOTIME,GPL10588,MBA_5212,Z2 P12 D21 Hystem BMP4 10ng/ml + TGFb3 10ng/ml,,

iPSC,BIOTIME,GPL10588,MBA_5213,Z2 P12 D21 Hystem BMP4 10ng/ml ,,

iPSC,BIOTIME,GPL10588,MBA_5214,Z2 P12 D21 Hystem BMP2 50ng/ml + TGFb3 10ng/ml,,

iPSC,BIOTIME,GPL10588,MBA_5215,Z2 P12 D21 Hystem RA 1uM,,

iPSC,BIOTIME,GPL10588,MBA_5216,Z2 P12 D21 Hystem osteo no serum,,

iPSC,BIOTIME,GPL10588,MBA_5218,7SMOO9 P13 D14 confluence adipose,,

iPSC,BIOTIME,GPL10588,MBA_5219,7SMOO9 P12 D21 Hystem BMP4 10ng/ml,,

iPSC,BIOTIME,GPL10588,MBA_5220,7SMOO9 P12 D21 Hystem BMP4 10ng/ml + TGFb3 10ng/ml,,

iPSC,BIOTIME,GPL10588,MBA_5221,7SMOO9 P12 D21 Hystem RA 1uM,,

iPSC,BIOTIME,GPL10588,MBA_5222,7SMOO9 P12 D21 Hystem BMP2 50ng/ml + TGFb3 10ng/ml,,

iPSC,BIOTIME,GPL10588,MBA_5223,7SMOO9 P12 D21 Hystem osteo no serum,,

iPSC,BIOTIME,GPL10588,MBA_5224,Z11 P16 D14 confluence adipose,,

iPSC,BIOTIME,GPL10588,MBA_5225,Z11 P16 D21 Hystem BMP4 10ng/ml,,

iPSC,BIOTIME,GPL10588,MBA_5226,Z11 P16 D21 Hystem BMP4 10ng/ml + TGFb3 10ng/ml,,

iPSC,BIOTIME,GPL10588,MBA_5227,Z11 P16 D21 Hystem RA 1uM,,

iPSC,BIOTIME,GPL10588,MBA_5228,Z11 P16 D21 Hystem BMP2 50ng/ml + TGFb3 10ng/ml,,

iPSC,BIOTIME,GPL10588,MBA_5229,Z11 P16 D21 Hystem osteo (no serum),,

iPSC,BIOTIME,GPL10588,MBA_5230,SM30 P18 D21 confluence BMP2 50ng/ml,,

iPSC,BIOTIME,GPL10588,MBA_5231,SM30 P18 D21 confluence BMP2 50ng/ml + TGFb3 10ng/ml,,

iPSC,BIOTIME,GPL10588,MBA_5232,SM30 P18 D21 confluence BMP4 10ng/ml + TGFb3 10ng/ml,,

iPSC,BIOTIME,GPL10588,MBA_5233,SM30 P18 D21 confluence b-glycerophosphate 10mM+ BMP2 50ng/ml,,

iPSC,BIOTIME,GPL10588,MBA_5234,SM30 P18 D21 confluence b-glycerophosphate 10mM + BMP2 50ng/ml + TGFb3 10ng/ml,,

iPSC,BIOTIME,GPL10588,MBA_5235,SM30 P18 D21 confluence b-glycerophosphate 10mM+ BMP2 50ng/ml + high Ascorbate (2x),,

iPSC,BIOTIME,GPL10588,MBA_5236,SM30 P18 D21 confluence b-glycerophosphate 10mM+ BMP2 50ng/ml (no dex),,

iPSC,BIOTIME,GPL10588,MBA_5237,SM30 P18 D21 confluence b-glycerophosphate 10mM+ BMP2 50ng/ml (10nM Dex-low 1/10),,

iPSC,BIOTIME,GPL10588,MBA_5238,SM30 P18 D21 confluence b-glycerophosphate 10mM+ BMP2 50ng/ml (10nM Dex-low 1/10 + high Ascorbate-2X),,

iPSC,BIOTIME,GPL10588,MBA_5239,SK11 P18 D21 confluence BMP2 50ng/ml,,

iPSC,BIOTIME,GPL10588,MBA_5240,SK11 P18 D21 confluence BMP2 50ng/ml + TGFb3 10ng/ml,,

iPSC,BIOTIME,GPL10588,MBA_5241,SK11 P18 D21 confluence BMP4 10ng/ml + TGFb3 10ng/ml,,

iPSC,BIOTIME,GPL10588,MBA_5242,SK11 P18 D21 confluence b-glycerophosphate 10mM+ BMP2 50ng/ml,,

iPSC,BIOTIME,GPL10588,MBA_5243,SK11 P18 D21 confluence b-glycerophosphate 10mM + BMP2 50ng/ml + TGFb3 10ng/ml,,

iPSC,BIOTIME,GPL10588,MBA_5244,SK11 P18 D21 confluence b-glycerophosphate 10mM+ BMP2 50ng/ml + high Ascorbate (2x),,

iPSC,BIOTIME,GPL10588,MBA_5245,SK11 P18 D21 confluence b-glycerophosphate 10mM+ BMP2 50ng/ml (no dex),,

iPSC,BIOTIME,GPL10588,MBA_5246,SK11 P18 D21 confluence b-glycerophosphate 10mM+ BMP2 50ng/ml (10nM Dex-low 1/10),,

iPSC,BIOTIME,GPL10588,MBA_5247,SK11 P18 D21 confluence b-glycerophosphate 10mM+ BMP2 50ng/ml (10nM Dex-low 1/10 + high Ascorbate-2X),,

iPSC,BIOTIME,GPL10588,MBA_5254,7PEND24 P21 D14 confluence adipose,,

iPSC,BIOTIME,GPL10588,MBA_5255,7PEND24 P21 D21 Hystem BMP4 10ng/ml,,

iPSC,BIOTIME,GPL10588,MBA_5256,7PEND24 P21 D21 Hystem BMP4 10ng/ml + TGFb3 10ng/ml,,

iPSC,BIOTIME,GPL10588,MBA_5257,7PEND24 P21 D21 Hystem BMP2 50ng/ml + TGFb3 10ng/ml,,

iPSC,BIOTIME,GPL10588,MBA_5258,7PEND24 P21 D21 Hystem RA 1uM,,

iPSC,BIOTIME,GPL10588,MBA_5259,7PEND24 P21 D21 Hystem osteo no serum,,

iPSC,BIOTIME,GPL10588,MBA_5261,EN16 P15 D21 Hystem BMP4 10ng/ml ,,

iPSC,BIOTIME,GPL10588,MBA_5262,EN16 P15 D21 Hystem BMP4 10ng/ml + TGFb3 10ng/ml,,

iPSC,BIOTIME,GPL10588,MBA_5263,EN16 P15 D21 Hystem RA 1uM,,

iPSC,BIOTIME,GPL10588,MBA_5264,EN16 P15 D21 Hystem BMP2 50ng/ml + TGFb3 10ng/ml,,

iPSC,BIOTIME,GPL10588,MBA_5265,EN16 P15 D21 Hystem osteo (no serum),,

iPSC,BIOTIME,GPL10588,MBA_5266,EN16 P15 D14 confluence adipo,,

iPSC,BIOTIME,GPL10588,MBA_5268,7SMOO4 P12 D21 Hystem BMP4 10ng/ml + TGFb3 10ng/ml,,

iPSC,BIOTIME,GPL10588,MBA_5269,7SMOO4 P12 D21 Hystem BMP4 10ng/ml ,,

iPSC,BIOTIME,GPL10588,MBA_5270,7SMOO4 P12 D21 Hystem BMP2 50ng/ml + TGFb3 10ng/ml,,

iPSC,BIOTIME,GPL10588,MBA_5271,7SMOO4 P12 D21 Hystem RA 1uM,,

iPSC,BIOTIME,GPL10588,MBA_5272,7SMOO4 P12 D21 Hystem osteo (no serum),,

iPSC,BIOTIME,GPL10588,MBA_5273,7SMOO4 P12 D14 confluence adipo,,

iPSC,BIOTIME,GPL10588,MBA_5275,Z3 P12 D21 Hystem BMP4 10ng/ml + TGFb3 10ng/ml,,

iPSC,BIOTIME,GPL10588,MBA_5276,Z3 P12 D21 Hystem BMP4 10ng/ml ,,

iPSC,BIOTIME,GPL10588,MBA_5277,Z3 P12 D21 Hystem BMP2 50ng/ml + TGFb3 10ng/ml,,

iPSC,BIOTIME,GPL10588,MBA_5278,Z3 P12 D21 Hystem RA 1uM,,

iPSC,BIOTIME,GPL10588,MBA_5279,Z3 P12 D21 Hystem osteo (no serum),,

iPSC,BIOTIME,GPL10588,MBA_5280,Z3 P12 D14 confluence adipo,,

iPSC,BIOTIME,GPL10588,MBA_5282,T36 P11 D21 Hystem BMP4 10ng/ml + TGFb3 10ng/ml,,

iPSC,BIOTIME,GPL10588,MBA_5283,T36 P11 D21 Hystem BMP4 10ng/ml ,,

iPSC,BIOTIME,GPL10588,MBA_5284,T36 P11 D21 Hystem BMP2 50ng/ml + TGFb3 10ng/ml,,

iPSC,BIOTIME,GPL10588,MBA_5285,T36 P11 D21 Hystem RA 1uM,,

iPSC,BIOTIME,GPL10588,MBA_5286,T36 P11 D21 Hystem osteo (no serum),,

iPSC,BIOTIME,GPL10588,MBA_5287,T36 P12 confluence adipo,,

AC,BIOTIME,GPL10588,MBA_5288,R1 joint capsule,,

AC,BIOTIME,GPL10588,MBA_5289,R2 Synovial membrane,,

AC,BIOTIME,GPL10588,MBA_5290,R3 Muscle (Vastus medialis) bulk of muscle,,

AC,BIOTIME,GPL10588,MBA_5291,R4 Muscle (Quadraceps) insertion Fibrocartilage patellar insertion,,

AC,BIOTIME,GPL10588,MBA_5292,R5 fat pad (may be contaminated with synovial membrane),,

AC,BIOTIME,GPL10588,MBA_5293,R6 Patellar tendon,,

AC,BIOTIME,GPL10588,MBA_5294,R7 ACL,,

AC,BIOTIME,GPL10588,MBA_5295,R8 PCL,,

AC,BIOTIME,GPL10588,MBA_5296,R9 Lateral mesiscus,,

AC,BIOTIME,GPL10588,MBA_5297,R10 Medial meniscus,,

AC,BIOTIME,GPL10588,MBA_5298,R11 Perichondrium,,

AC,BIOTIME,GPL10588,MBA_5299,R12 Subcutaneous fat,,

AC,BIOTIME,GPL10588,MBA_5302,R15 Articular cartilage (Femoral condyle),,

AC,BIOTIME,GPL10588,MBA_5304,R17 Subchondral bone (Tibial plateau),,

AC,BIOTIME,GPL10588,MBA_5305,R18 Subcondral bone (Femoral condyle),,

AC,BIOTIME,GPL10588,MBA_5306,R19 Subcondral bone (patella),,

AC,BIOTIME,GPL10588,MBA_5307,R20 Articular cartilage (Femoral trochlear groove),,

iPSC,BIOTIME,GPL10588,MBA_5309,RAD20.5 P17 D14 Hystem TGFb3 10ng/ml,,

iPSC,BIOTIME,GPL10588,MBA_5310,RAD20.5 P17 D14 Hystem TGFb3 10ng/ml,,

iPSC,BIOTIME,GPL10588,MBA_5311,RAD20.5 P17 D14 Hystem TGFb3 10ng/ml,,

iPSC,BIOTIME,GPL10588,MBA_5312,RAD20.5 P17 D14 Hystem BMP2 50ng/ml,,

iPSC,BIOTIME,GPL10588,MBA_5313,RAD20.5 P17 D14 Hystem BMP2 50ng/ml,,

iPSC,BIOTIME,GPL10588,MBA_5314,RAD20.5 P17 D14 Hystem BMP2 50ng/ml,,

iPSC,BIOTIME,GPL10588,MBA_5315,RAD20.5 P17 D14 Hystem BMP2 50ng/ml + TGFb3 10ng/ml,,

iPSC,BIOTIME,GPL10588,MBA_5316,RAD20.5 P17 D14 Hystem BMP2 50ng/ml + TGFb3 10ng/ml,,

iPSC,BIOTIME,GPL10588,MBA_5317,RAD20.5 P17 D14 Hystem BMP2 50ng/ml + TGFb3 10ng/ml,,

iPSC,BIOTIME,GPL10588,MBA_5318,RAD20.5 P17 D14 Hystem BMP4 10ng/ml,,

iPSC,BIOTIME,GPL10588,MBA_5319,RAD20.5 P17 D14 Hystem BMP4 10ng/ml,,

iPSC,BIOTIME,GPL10588,MBA_5320,RAD20.5 P17 D14 Hystem BMP4 10ng/ml,,

iPSC,BIOTIME,GPL10588,MBA_5321,RAD20.5 P17 D14 Hystem BMP4 10ng/ml + TGFb3 10ng/ml,,

iPSC,BIOTIME,GPL10588,MBA_5322,RAD20.5 P17 D14 Hystem BMP4 10ng/ml + TGFb3 10ng/ml,,

iPSC,BIOTIME,GPL10588,MBA_5323,RAD20.5 P17 D14 Hystem BMP4 10ng/ml + TGFb3 10ng/ml,,

iPSC,BIOTIME,GPL10588,MBA_5324,RAD20.5 P17 D14 Hystem BMP6 30ng/ml,,

iPSC,BIOTIME,GPL10588,MBA_5325,RAD20.5 P17 D14 Hystem BMP6 30ng/ml,,

iPSC,BIOTIME,GPL10588,MBA_5326,RAD20.5 P17 D14 Hystem BMP6 30ng/ml,,

iPSC,BIOTIME,GPL10588,MBA_5327,RAD20.5 P17 D14 Hystem BMP6 30ng/ml + TGFb3 10ng/ml,,

iPSC,BIOTIME,GPL10588,MBA_5328,RAD20.5 P17 D14 Hystem BMP6 30ng/ml + TGFb3 10ng/ml,,

iPSC,BIOTIME,GPL10588,MBA_5329,RAD20.5 P17 D14 Hystem BMP6 30ng/ml + TGFb3 10ng/ml,,

iPSC,BIOTIME,GPL10588,MBA_5330,RAD20.5 P17 D14 Hystem BMP7 100ng/ml,,

iPSC,BIOTIME,GPL10588,MBA_5331,RAD20.5 P17 D14 Hystem BMP7 100ng/ml,,

iPSC,BIOTIME,GPL10588,MBA_5332,RAD20.5 P17 D14 Hystem BMP7 100ng/ml,,

iPSC,BIOTIME,GPL10588,MBA_5333,RAD20.5 P17 D14 Hystem BMP7 100ng/ml + TGFb3 10ng/ml,,

iPSC,BIOTIME,GPL10588,MBA_5334,RAD20.5 P17 D14 Hystem BMP7 100ng/ml + TGFb3 10ng/ml,,

iPSC,BIOTIME,GPL10588,MBA_5335,RAD20.5 P17 D14 Hystem BMP7 100ng/ml + TGFb3 10ng/ml,,

iPSC,BIOTIME,GPL10588,MBA_5336,RAD20.5 P17 D14 Hystem GDF5 100ng/ml,,

iPSC,BIOTIME,GPL10588,MBA_5337,RAD20.5 P17 D14 Hystem GDF5 100ng/ml,,

iPSC,BIOTIME,GPL10588,MBA_5338,RAD20.5 P17 D14 Hystem GDF5 100ng/ml,,

iPSC,BIOTIME,GPL10588,MBA_5339,RAD20.5 P17 D14 Hystem GDF5 100ng/ml + TGFb3 10ng/ml,,

iPSC,BIOTIME,GPL10588,MBA_5340,RAD20.5 P17 D14 Hystem GDF5 100ng/ml + TGFb3 10ng/ml,,

iPSC,BIOTIME,GPL10588,MBA_5341,RAD20.5 P17 D14 Hystem GDF5 100ng/ml + TGFb3 10ng/ml,,

iPSC,BIOTIME,GPL10588,MBA_5342,RAD20.5 P17 D14 Hystem F4 (10ng/ml each) cocktail,,

iPSC,BIOTIME,GPL10588,MBA_5343,RAD20.5 P17 D14 Hystem F4 (10ng/ml each) cocktail,,

iPSC,BIOTIME,GPL10588,MBA_5344,RAD20.5 P17 D14 Hystem F4 (10ng/ml each) cocktail,,

iPSC,BIOTIME,GPL10588,MBA_5345,RAD20.5 P17 D14 Hystem F4 (10ng/ml each) cocktail + TGFb3 10ng/ml,,

iPSC,BIOTIME,GPL10588,MBA_5346,RAD20.5 P17 D14 Hystem F4 (10ng/ml each) cocktail + TGFb3 10ng/ml,,

iPSC,BIOTIME,GPL10588,MBA_5347,RAD20.5 P17 D14 Hystem F4 (10ng/ml each) cocktail + TGFb3 10ng/ml,,

iPSC,BIOTIME,GPL10588,MBA_5348,RAD20.5 P17 D14 Hystem F4 + BMP2 50ng/ml,,

iPSC,BIOTIME,GPL10588,MBA_5349,RAD20.5 P17 D14 Hystem F4 + BMP2 50ng/ml,,

iPSC,BIOTIME,GPL10588,MBA_5350,RAD20.5 P17 D14 Hystem F4 + BMP2 50ng/ml,,

iPSC,BIOTIME,GPL10588,MBA_5351,RAD20.5 P17 D14 Hystem F4 + BMP2 50ng/ml + TGFb3 10ng/ml,,

iPSC,BIOTIME,GPL10588,MBA_5352,RAD20.5 P17 D14 Hystem F4 + BMP2 50ng/ml + TGFb3 10ng/ml,,

iPSC,BIOTIME,GPL10588,MBA_5353,RAD20.5 P17 D14 Hystem F4 + BMP2 50ng/ml + TGFb3 10ng/ml,,

iPSC,BIOTIME,GPL10588,MBA_5354,RAD20.5 P17 D14 Hystem F4 + BMP4 10ng/ml,,

iPSC,BIOTIME,GPL10588,MBA_5355,RAD20.5 P17 D14 Hystem F4 + BMP4 10ng/ml,,

iPSC,BIOTIME,GPL10588,MBA_5356,RAD20.5 P17 D14 Hystem F4 + BMP4 10ng/ml,,

iPSC,BIOTIME,GPL10588,MBA_5357,RAD20.5 P17 D14 Hystem F4 + BMP4 10ng/ml + TGFb3 10ng/ml,,

iPSC,BIOTIME,GPL10588,MBA_5358,RAD20.5 P17 D14 Hystem F4 + BMP4 10ng/ml + TGFb3 10ng/ml,,

iPSC,BIOTIME,GPL10588,MBA_5359,RAD20.5 P17 D14 Hystem F4 + BMP4 10ng/ml + TGFb3 10ng/ml,,

iPSC,BIOTIME,GPL10588,MBA_5360,RAD20.5 P17 D14 Hystem F4 + GDF5 100ng/ml ,,

iPSC,BIOTIME,GPL10588,MBA_5361,RAD20.5 P17 D14 Hystem F4 + GDF5 100ng/ml ,,

iPSC,BIOTIME,GPL10588,MBA_5362,RAD20.5 P17 D14 Hystem F4 + GDF5 100ng/ml ,,

iPSC,BIOTIME,GPL10588,MBA_5363,RAD20.5 P17 D14 Hystem F4 + GDF5 100ng/ml + TGFb3 10ng/ml,,

iPSC,BIOTIME,GPL10588,MBA_5364,RAD20.5 P17 D14 Hystem F4 + GDF5 100ng/ml + TGFb3 10ng/ml,,

iPSC,BIOTIME,GPL10588,MBA_5365,RAD20.5 P17 D14 Hystem F4 + GDF5 100ng/ml + TGFb3 10ng/ml,,

iPSC,BIOTIME,GPL10588,MBA_5367,RAD20.24 P12 D21 Hystem BMP4 10ng/ml + TGFb3 10ng/ml,,

iPSC,BIOTIME,GPL10588,MBA_5368,RAD20.24 P12 D21 Hystem BMP4 10ng/ml ,,

iPSC,BIOTIME,GPL10588,MBA_5369,RAD20.24 P12 D21 Hystem BMP2 50ng/ml + TGFb3 10ng/ml,,

iPSC,BIOTIME,GPL10588,MBA_5370,RAD20.24 P12 D21 RA 1uM,,

iPSC,BIOTIME,GPL10588,MBA_5371,RAD20.24 P12 D21 Hystem osteo (no serum),,

iPSC,BIOTIME,GPL10588,MBA_5372,RAD20.24 P12 D14 confluence adipo,,

iPSC,BIOTIME,GPL10588,MBA_5374,E3 P13 D21 Hystem BMP4 10ng/ml,,

iPSC,BIOTIME,GPL10588,MBA_5375,E3 P13 D21 Hystem BMP4 10ng/ml + TGFb3 10ng/ml,,

iPSC,BIOTIME,GPL10588,MBA_5376,E3 P13 D21 Hystem RA 1uM,,

iPSC,BIOTIME,GPL10588,MBA_5377,E3 P13 D21 Hystem BMP2 50ng/ml + TGFb3 10ng/ml,,

iPSC,BIOTIME,GPL10588,MBA_5378,E3 P13 D21 Hystem osteo,,

iPSC,BIOTIME,GPL10588,MBA_5379,E3 P13 D14 confluence adipo,,

iPSC,BIOTIME,GPL10588,MBA_5381,C4ELSR14 P13 D21 Hystem BMP4 10ng/ml + TGFb3 10ng/ml,,

iPSC,BIOTIME,GPL10588,MBA_5382,C4ELSR14 P13 D21 Hystem BMP4 10ng/ml ,,

iPSC,BIOTIME,GPL10588,MBA_5383,C4ELSR14 P13 D21 Hystem RA 1uM,,

iPSC,BIOTIME,GPL10588,MBA_5384,C4ELSR14 P13 D21 Hystem osteo (no serum),,

iPSC,BIOTIME,GPL10588,MBA_5385,C4ELSR14 P13 D14 confluence adipo,,

iPSC,BIOTIME,GPL10588,MBA_5387,RAPEND4 P12 D21 Hystem BMP4 10ng/ml + TGFb3 10ng/ml,,

iPSC,BIOTIME,GPL10588,MBA_5388,RAPEND4 P12 D21 Hystem BMP4 10ng/ml ,,

iPSC,BIOTIME,GPL10588,MBA_5389,RAPEND4 P12 D21 Hystem BMP2 50ng/ml + TGFb3 10ng/ml,,

iPSC,BIOTIME,GPL10588,MBA_5390,RAPEND4 P12 D21 Hystem RA 1uM,,

iPSC,BIOTIME,GPL10588,MBA_5391,RAPEND4 P12 D21 Hystem osteo (no serum),,

iPSC,BIOTIME,GPL10588,MBA_5392,RAPEND4 P12 D14 confluence adipo,,

iPSC,BIOTIME,GPL10588,MBA_5394,E69 P16 D14 MM BMP4 10ng/ml,,

iPSC,BIOTIME,GPL10588,MBA_5396,T42 P17 D14 MM BMP4 10ng/ml,,

iPSC,BIOTIME,GPL10588,MBA_5398,MEL2 P19 D14 MM BMP4 10ng/ml,,

iPSC,BIOTIME,GPL10588,MBA_5400,E72 P12 D14 Hystem BMP4 10ng/ml,,

iPSC,BIOTIME,GPL10588,MBA_5401,E72 P12 D14 Hystem BMP4 10ng/ml,,

iPSC,BIOTIME,GPL10588,MBA_5402,E72 P12 D14 MM BMP4 10ng/ml,,

iPSC,BIOTIME,GPL10588,MBA_5403,E72 P12 D14 MM BMP4 10ng/ml,,

iPSC,BIOTIME,GPL10588,MBA_5404,E72 P12 D14 confluence BMP4 10ng/ml,,

iPSC,BIOTIME,GPL10588,MBA_5405,E72 P13 D14 Hystem BMP4 10ng/ml,,

iPSC,BIOTIME,GPL10588,MBA_5406,E72 P13 D14 Hystem BMP4 10ng/ml,,

iPSC,BIOTIME,GPL10588,MBA_5408,E75 P12 D14 Hystem BMP4 10ng/ml,,

iPSC,BIOTIME,GPL10588,MBA_5409,E75 P12 D14 Hystem BMP4 10ng/ml,,

iPSC,BIOTIME,GPL10588,MBA_5410,E75 P12 D14 confluence BMP4 10ng/ml,,

iPSC,BIOTIME,GPL10588,MBA_5411,E75 P12 D14 MM BMP4 10ng/ml,,

iPSC,BIOTIME,GPL10588,MBA_5412,E75 P12 D14 MM BMP4 10ng/ml,,

iPSC,BIOTIME,GPL10588,MBA_5413,E75 P14 D14 Hystem BMP4 10ng/ml,,

iPSC,BIOTIME,GPL10588,MBA_5415,E163 P13 D14 MM BMP4 10ng/ml,,

iPSC,BIOTIME,GPL10588,MBA_5416,E163 P13 D14 MM BMP4 10ng/ml,,

iPSC,BIOTIME,GPL10588,MBA_5417,E163 P13 D14 Hystem BMP4 10ng/ml,,

iPSC,BIOTIME,GPL10588,MBA_5418,E163 P13 D14 Hystem BMP4 10ng/ml,,

iPSC,BIOTIME,GPL10588,MBA_5419,E163 P12 D14 confluence BMP4 10ng/ml,,

AC,BIOTIME,GPL10588,MBA_5421,hMSC P7 D14 Hystem F4 10ng/ml each,,

AC,BIOTIME,GPL10588,MBA_5422,hMSC P7 D14 Hystem F4 10ng/ml each + BMP2 50ng/ml,,

AC,BIOTIME,GPL10588,MBA_5423,hMSC P7 D14 Hystem F4 10ng/ml each + BMP4 10ng/ml,,

AC,BIOTIME,GPL10588,MBA_5424,hMSC P7 D14 Hystem F4 10ng/ml each + GDF5 100ng/ml,,

AC,BIOTIME,GPL10588,MBA_5425,hMSC P7 D14 Hystem F4 10ng/ml each + TGFb3 10ng/ml,,

AC,BIOTIME,GPL10588,MBA_5426,hMSC P7 D14 Hystem F4 10ng/ml each + BMP2 50ng/ml + TGFb3 10ng/ml,,

AC,BIOTIME,GPL10588,MBA_5427,hMSC P7 D14 Hystem F4 10ng/ml each + BMP4 10ng/ml + TGFb3 10ng/ml,,

AC,BIOTIME,GPL10588,MBA_5428,hMSC P7 D14 Hystem F4 10ng/ml each + GDF 100ng/ml + TGFb3 10ng/ml,,

iPSC,BIOTIME,GPL10588,MBA_5430,RASKEL8 P11 D21 Hystem BMP4 10ng/ml,,

iPSC,BIOTIME,GPL10588,MBA_5431,RASKEL8 P11 D21 Hystem BMP4 10ng/ml + TGFb3 10ng/ml,,

iPSC,BIOTIME,GPL10588,MBA_5432,RASKEL8 P11 D21 Hystem RA 1uM,,

iPSC,BIOTIME,GPL10588,MBA_5433,RASKEL8 P11 D21 Hystem BMP2 50ng/ml + TGFb3 10ng/ml,,

iPSC,BIOTIME,GPL10588,MBA_5434,RASKEL8 P11 D21 Hystem osteo (no serum),,

iPSC,BIOTIME,GPL10588,MBA_5435,RASKEL8 P11 D14 confluence adipo,,

iPSC,BIOTIME,GPL10588,MBA_5437,RASKEL9 P12 D14 adipo,,

iPSC,BIOTIME,GPL10588,MBA_5438,RASKEL9 P12 D21 Hystem BMP4 10ng/ml ,,

iPSC,BIOTIME,GPL10588,MBA_5439,RASKEL9 P12 D21 Hystem BMP4 10ng/ml + TGFb3 10ng/ml,,

iPSC,BIOTIME,GPL10588,MBA_5440,RASKEL9 P12 D21 RA 1uM,,

iPSC,BIOTIME,GPL10588,MBA_5441,RASKEL9 P12 D21 Hystem BMP2 50ng/ml + TGFb3 10ng/ml,,

iPSC,BIOTIME,GPL10588,MBA_5442,RASKEL9 P12 D21 Hystem osteo (no serum),,

iPSC,BIOTIME,GPL10588,MBA_5444,RASKEL21 P11 D21 Hystem BMP4 10ng/ml,,

iPSC,BIOTIME,GPL10588,MBA_5445,RASKEL21 P11 D21 Hystem BMP4 10ng/ml + TGFb3 10ng/ml,,

iPSC,BIOTIME,GPL10588,MBA_5446,RASKEL21 P11 D21 Hystem RA 1uM,,

iPSC,BIOTIME,GPL10588,MBA_5447,RASKEL21 P11 D21 Hystem BMP2 50ng/ml + TGFb3 10ng/ml,,

iPSC,BIOTIME,GPL10588,MBA_5448,RASKEL21 P11 D21 Hystem osteo (no serum),,

iPSC,BIOTIME,GPL10588,MBA_5449,RASKEL21 P11 confluence adipo,,

iPSC,BIOTIME,GPL10588,MBA_5451,RASKEL3 P12 D21 Hystem BMP4 10ng/ml,,

iPSC,BIOTIME,GPL10588,MBA_5452,RASKEL3 P12 D21 Hystem BMP4 10ng/ml +TGFb3 10ng/ml,,

iPSC,BIOTIME,GPL10588,MBA_5453,RASKEL3 P12 D21 Hystem RA 1uM,,

iPSC,BIOTIME,GPL10588,MBA_5454,RASKEL3 P12 D21 Hystem BMP2 50ng/ml +TGFb3 10ng/ml,,

iPSC,BIOTIME,GPL10588,MBA_5455,RASKEL3 P12 D21 Hystem osteo (no serum),,

iPSC,BIOTIME,GPL10588,MBA_5456,RASKEL3 P12 confluence adipo,,

iPSC,BIOTIME,GPL10588,MBA_5458,E67 P14 D21 Hystem BMP4 10ng/ml,,

iPSC,BIOTIME,GPL10588,MBA_5459,E67 P14 D21 Hystem BMP4 10ng/ml + TGFb3 10ng/ml,,

iPSC,BIOTIME,GPL10588,MBA_5460,E67 P14 D21 Hystem RA 1uM,,

iPSC,BIOTIME,GPL10588,MBA_5461,E67 P14 D21 Hystem BMP2 50ng/ml + TGFb3 10ng/ml,,

iPSC,BIOTIME,GPL10588,MBA_5462,E67 P14 D21 Hystem osteo (no serum),,

iPSC,BIOTIME,GPL10588,MBA_5463,E67 P14 D14 confluence adipo,,

iPSC,BIOTIME,GPL10588,MBA_5465,E73 P11 D21 Hsytem BMP4 10ng/ml,,

iPSC,BIOTIME,GPL10588,MBA_5466,E73 P11 D21 Hystem BMP4 10ng/ml + TGFb3 10ng/ml,,

iPSC,BIOTIME,GPL10588,MBA_5467,E73 P11 D21 Hystem RA 1uM,,

iPSC,BIOTIME,GPL10588,MBA_5468,E73 P11 D21 Hystem BMP2 50ng/ml + TGFb3 10ng/ml,,

iPSC,BIOTIME,GPL10588,MBA_5469,E73 P11 D21 Hystem osteo (no serum),,

iPSC,BIOTIME,GPL10588,MBA_5470,E73 P11 D14 adipo,,

iPSC,BIOTIME,GPL10588,MBA_5472,E53 P15 D21 Hystem BMP4 10ng/ml + TGFb3 10ng/ml,,

iPSC,BIOTIME,GPL10588,MBA_5473,E53 P15 D21 Hystem BMP4 10ng/ml,,

iPSC,BIOTIME,GPL10588,MBA_5474,E53 P15 D21 Hystem BMP2 50ng/ml + TGFb3 10ng/ml,,

iPSC,BIOTIME,GPL10588,MBA_5475,E53 P15 D21 Hystem RA 1uM,,

iPSC,BIOTIME,GPL10588,MBA_5476,E53 P15 D21 Hystem osteo (no serum),,

iPSC,BIOTIME,GPL10588,MBA_5477,E53 P15 D14 confluence adipo,,

iPSC,BIOTIME,GPL10588,MBA_5479,E17 P12 D21 Hystem BMP4 10ng/ml + TGFb3 10ng/ml,,

iPSC,BIOTIME,GPL10588,MBA_5480,E17 P12 D21 Hystem BMP4 10ng/ml ,,

iPSC,BIOTIME,GPL10588,MBA_5481,E17 P12 D21 Hystem RA 1uM,,

AC,BIOTIME,GPL10588,MBA_5484,RT MCL P1,,

AC,BIOTIME,GPL10588,MBA_5485,RT meniscus P1 (a),,

AC,BIOTIME,GPL10588,MBA_5487,Inferior patellar tendon P1,,

AC,BIOTIME,GPL10588,MBA_5489,Left meniscus (b) P1,,

AC,BIOTIME,GPL10588,MBA_5491,RT medial tendon P1,,

AC,BIOTIME,GPL10588,MBA_5494,Rt femur cartilage P1,,

iPSC,BIOTIME,GPL10588,MBA_5498,MW6 P10 D21 Hystem BMP4 10ng/ml,,

iPSC,BIOTIME,GPL10588,MBA_5499,MW6 P10 D21 Hystem BMP4 10ng/ml + TGFb3 10ng/ml,,

iPSC,BIOTIME,GPL10588,MBA_5500,MW6 P10 D21 Hystem RA 1uM,,

iPSC,BIOTIME,GPL10588,MBA_5501,MW6 P10 D21 Hystem BMP2 50ng/ml + TGFb3 10ng/ml,,

iPSC,BIOTIME,GPL10588,MBA_5502,MW6 P10 D21 Hystem osteo (no serum),,

iPSC,BIOTIME,GPL10588,MBA_5503,MW6 P10 D14 confluence adipo,,

iPSC,BIOTIME,GPL10588,MBA_5505,C4ELS5.5 P15 D21 Hystem BMP4 10ng/ml + TGFb3 10ng/ml,,

iPSC,BIOTIME,GPL10588,MBA_5506,C4ELS5.5 P15 D21 Hystem BMP2 50ng/ml + TGFb3 10ng/ml,,

iPSC,BIOTIME,GPL10588,MBA_5507,C4ELS5.5 P15 D21 Hystem osteo (no serum),,

iPSC,BIOTIME,GPL10588,MBA_5509,SK8 P10 D21 Hystem BMP4 10ng/ml,,

iPSC,BIOTIME,GPL10588,MBA_5510,SK8 P10 D21 Hystem BMP4 10ng/ml + TGFb3 10ng/ml,,

iPSC,BIOTIME,GPL10588,MBA_5511,SK8 P10 D21 Hystem RA 1uM,,

iPSC,BIOTIME,GPL10588,MBA_5512,SK8 P10 D21 Hystem BMP2 50ng/ml + TGFb3 10ng/ml,,

iPSC,BIOTIME,GPL10588,MBA_5513,SK8 P10 D21 Hystem osteo (no serum),,

iPSC,BIOTIME,GPL10588,MBA_5514,SK8 P10 D14 confluence adipo,,

iPSC,BIOTIME,GPL10588,MBA_5516,E65 P11 D14 confluence adipo,,

iPSC,BIOTIME,GPL10588,MBA_5517,E65 P11 D21 Hystem BMP4 10ng/ml + TGFb3 10ng/ml,,

iPSC,BIOTIME,GPL10588,MBA_5518,E65 P11 D21 Hystem BMP4 10ng/ml ,,

iPSC,BIOTIME,GPL10588,MBA_5519,E65 P11 D21 Hystem BMP2 50ng/ml + TGFb3 10ng/ml,,

iPSC,BIOTIME,GPL10588,MBA_5520,E65 P11 D21 Hystem RA 1uM,,

iPSC,BIOTIME,GPL10588,MBA_5521,E65 P11 D21 Hystem osteo (no serum),,

iPSC,BIOTIME,GPL10588,MBA_5522,E15 P20 D1 confluence BMP4 10ng/ml + TGFb3 10ng/ml,,

iPSC,BIOTIME,GPL10588,MBA_5523,E15 P20 D1 confluence BMP4 10ng/ml + TGFb3 10ng/ml 10x,,

iPSC,BIOTIME,GPL10588,MBA_5524,E15 P20 D3 confluence BMP4 10ng/ml + TGFb3 10ng/ml 1 feed,,

iPSC,BIOTIME,GPL10588,MBA_5525,E15 P20 D3 confluence BMP4 100ng/ml + TGFb3 100ng/ml 1 feed,,

iPSC,BIOTIME,GPL10588,MBA_5526,E15 P20 D7 confluence BMP4 10ng/ml + TGFb3 10ng/ml 1 feed,,

iPSC,BIOTIME,GPL10588,MBA_5527,E15 P20 D7 confluence BMP4 100ng/ml + TGFb3 100ng/ml multiple feed,,

iPSC,BIOTIME,GPL10588,MBA_5528,E15 P20 D7 confluence BMP4 100ng/ml + TGFb3 100ng/ml 1 feed,,

iPSC,BIOTIME,GPL10588,MBA_5529,4D20.8 P14 D1 BMP4 10ng/ml + TGFb3 10ng/ml 1 feed,,

iPSC,BIOTIME,GPL10588,MBA_5530,4D20.8 P14 D1 BMP4 100ng/ml + TGFb3 100ng/ml 1 feed,,

iPSC,BIOTIME,GPL10588,MBA_5531,4D20.8 P14 D3 BMP4 10ng/ml + TGFb3 10ng/ml 1 feed,,

iPSC,BIOTIME,GPL10588,MBA_5532,4D20.8 P14 D3 BMP4 100ng/ml + TGFb3 100ng/ml 1feed,,

iPSC,BIOTIME,GPL10588,MBA_5533,4D20.8 P14 D7 BMP4 10ng/ml + TGFb3 10ng/ml 1 feed,,

iPSC,BIOTIME,GPL10588,MBA_5534,4D20.8 P14 D7 BMP4 100ng/ml + TGFb3 100ng/ml multiple feed,,

iPSC,BIOTIME,GPL10588,MBA_5535,4D20.8 P14 D7 BMP4 100ng/ml + TGFb3 100ng/ml 1 feed,,

iPSC,BIOTIME,GPL10588,MBA_5536,RAD20.5 P17 D1 confluence BMP4 10ng/ml + TGFb3 10ng/ml,,

iPSC,BIOTIME,GPL10588,MBA_5537,RAD20.5 P17 D1 confluence BMP4 100ng/ml + TGFb3 100ng/ml,,

iPSC,BIOTIME,GPL10588,MBA_5538,RAD20.5 P17 D3 confluence BMP4 10ng/ml + TGFb3 10ng/ml,,

iPSC,BIOTIME,GPL10588,MBA_5539,RAD20.5 P17 D3 confluence BMP4 100ng/ml + TGFb3 100ng/ml 1 feed,,

iPSC,BIOTIME,GPL10588,MBA_5540,RAD20.5 P17 D7 confluence BMP4 10ng/ml + TGFb3 10ng/ml,,

iPSC,BIOTIME,GPL10588,MBA_5541,RAD20.5 P17 D7 confluence BMP4 100ng/ml + TGFb3 100ng/ml multiple dose,,

iPSC,BIOTIME,GPL10588,MBA_5542,RAD20.5 P17 D7 confluence BMP4 100ng/ml + TGFb3 100ng/ml single dose,,

AC,BIOTIME,GPL10588,MBA_5543,hMSC P5 D1 confluence BMP4 10ng/ml + TGFb3 10ng/ml,,

AC,BIOTIME,GPL10588,MBA_5544,hMSC P5 D1 confluence BMP4 100ng/ml + TGFb3 100ng/ml,,

AC,BIOTIME,GPL10588,MBA_5545,hMSC P5 D3 confluence BMP4 10ng/ml + TGFb3 10ng/ml 1 feed,,

AC,BIOTIME,GPL10588,MBA_5546,hMSC P7 D3 confluence BMP4 100ng/ml + TGFb3 100ng/ml,,

AC,BIOTIME,GPL10588,MBA_5547,hMSC P7 D7 confluence BMP4 10ng/ml + TGFb3 10ng/ml,,

AC,BIOTIME,GPL10588,MBA_5548,hMSC P7 D7 confluence BMP4 100ng/ml + TGFb3 100ng/ml multiple dose,,

AC,BIOTIME,GPL10588,MBA_5549,hMSC P7 D7 confluence BMP4 100ng/ml + TGFb3 100ng/ml 1 dose,,

iPSC,BIOTIME,GPL10588,MBA_5550,SM30 P16 D1 confluence BMP4 10ng/ml + TGFb3 10ng/ml,,

iPSC,BIOTIME,GPL10588,MBA_5551,SM30 P16 D1 confluence BMP4 100ng/ml + TGFb3 100ng/ml,,

iPSC,BIOTIME,GPL10588,MBA_5552,SM30 P16 D3 confluence BMP4 10ng/ml + TGFb3 10ng/ml,,

iPSC,BIOTIME,GPL10588,MBA_5553,SM30 P16 D3 confluence BMP4 100ng/ml + TGFb3 100ng/ml,,

iPSC,BIOTIME,GPL10588,MBA_5554,SM30 P16 D7 confluence BMP4 10ng/ml + TGFb3 10ng/ml,,

iPSC,BIOTIME,GPL10588,MBA_5555,SM30 P16 D7 confluence BMP4 100ng/ml + TGFb3 100ng/ml multiple dose,,

iPSC,BIOTIME,GPL10588,MBA_5556,SM30 P16 D7 confluence BMP4 100ng/ml + TGFb3 100ng/ml single dose,,

iPSC,BIOTIME,GPL10588,MBA_5557,7SMOO32 P15 D1 BMP4 10ng/ml + TGFb3 10ng/ml,,

iPSC,BIOTIME,GPL10588,MBA_5558,7SMOO32 P15 D1 BMP4 100ng/ml + TGFb3 100ng/ml,,

iPSC,BIOTIME,GPL10588,MBA_5559,7SMOO32 P15 D3 BMP4 10ng/ml + TGFb3 10ng/ml,,

iPSC,BIOTIME,GPL10588,MBA_5560,7SMOO32 P15 D3 BMP4 100ng/ml + TGFb3 100ng/ml,,

iPSC,BIOTIME,GPL10588,MBA_5561,7SMOO32 P15 D7 congluence BMP4 10ng/ml + TGFb3 10ng/ml,,

iPSC,BIOTIME,GPL10588,MBA_5562,7SMOO32 P15 D7 confluence BMP4 100ng/ml + TGFb3 100ng/ml multiple dose,,

iPSC,BIOTIME,GPL10588,MBA_5563,7SMOO32 P15 D7 confluence BMP4 100ng/ml + TGFb3 100ng/ml single dose,,

iPSC,BIOTIME,GPL10588,MBA_5564,T42 P19 D3 confluence BMP4 10ng/ml,,

iPSC,BIOTIME,GPL10588,MBA_5565,T42 P19 D3 confluence BMP4 25ng/ml,,

iPSC,BIOTIME,GPL10588,MBA_5566,T42 P19 D3 confluence BMP4 100ng/ml,,

iPSC,BIOTIME,GPL10588,MBA_5567,T42 P19 D3 confluence BMP4 10ng/ml + TGFb3 10ng/ml,,

iPSC,BIOTIME,GPL10588,MBA_5568,T42 P19 D3 confluence BMP4 25ng/ml + TGFb3 10ng/ml,,

iPSC,BIOTIME,GPL10588,MBA_5569,T42 P19 D3 confluence BMP4 100ng/ml + TGFb3 10ng/ml,,

iPSC,BIOTIME,GPL10588,MBA_5570,T42 P19 D3 confluence GDF5 100ng/ml ,,

iPSC,BIOTIME,GPL10588,MBA_5571,T42 P19 D3 confluence GDF5 250ng/ml ,,

iPSC,BIOTIME,GPL10588,MBA_5572,T42 P19 D3 confluence GDF5 100ng/ml + TGFb3 10ng/ml,,

iPSC,BIOTIME,GPL10588,MBA_5573,T42 P19 D3 confluence GDF5 250ng/ml + TGFb3 10ng/ml,,

iPSC,BIOTIME,GPL10588,MBA_5574,T42 P19 D7 confluence BMP4 10ng/ml,,

iPSC,BIOTIME,GPL10588,MBA_5575,T42 P19 D7 confluence BMP4 25ng/ml,,

iPSC,BIOTIME,GPL10588,MBA_5576,T42 P19 D7 confluence BMP4 100ng/ml,,

iPSC,BIOTIME,GPL10588,MBA_5577,T42 P19 D7 confluence BMP4 10ng/ml + TGFb3 10ng/ml,,

iPSC,BIOTIME,GPL10588,MBA_5578,T42 P19 D7 confluence BMP4 25ng/ml + TGFb3 10ng/ml,,

iPSC,BIOTIME,GPL10588,MBA_5579,T42 P19 D7 confluence BMP4 100ng/ml + TGFb3 10ng/ml,,

iPSC,BIOTIME,GPL10588,MBA_5580,T42 P19 D7 confluence GDF5 100ng/ml ,,

iPSC,BIOTIME,GPL10588,MBA_5581,T42 P19 D7 confluence GDF5 250ng/ml ,,

iPSC,BIOTIME,GPL10588,MBA_5582,T42 P19 D7 confluence GDF5 100ng/ml + TGFb3 10ng/ml,,

iPSC,BIOTIME,GPL10588,MBA_5583,T42 P19 D7 confluence GDF5 250ng/ml + TGFb3 10ng/ml,,

iPSC,BIOTIME,GPL10588,MBA_5584,T42 P19 D14 confluence BMP4 10ng/ml,,

iPSC,BIOTIME,GPL10588,MBA_5585,T42 P19 D14 confluence BMP4 25ng/ml,,

iPSC,BIOTIME,GPL10588,MBA_5586,T42 P19 D14 confluence BMP4 100ng/ml,,

iPSC,BIOTIME,GPL10588,MBA_5587,T42 P19 D14 confluence BMP4 10ng/ml + TGFb3 10ng/ml,,

iPSC,BIOTIME,GPL10588,MBA_5588,T42 P19 D14 confluence BMP4 25ng/ml + TGFb3 10ng/ml,,

iPSC,BIOTIME,GPL10588,MBA_5589,T42 P19 D14 confluence BMP4 100ng/ml + TGFb3 10ng/ml,,

iPSC,BIOTIME,GPL10588,MBA_5590,T42 P19 D14 confluence GDF5 100ng/ml ,,

iPSC,BIOTIME,GPL10588,MBA_5591,T42 P19 D14 confluence GDF5 250ng/ml ,,

iPSC,BIOTIME,GPL10588,MBA_5592,T42 P19 D14 confluence GDF5 250ng/ml ,,

iPSC,BIOTIME,GPL10588,MBA_5593,T42 P19 D14 confluence GDF5 100ng/ml + TGFb3 10ng/ml,,

iPSC,BIOTIME,GPL10588,MBA_5594,T42 P19 D14 confluence GDF5 250ng/ml + TGFb3 10ng/ml,,

iPSC,BIOTIME,GPL10588,MBA_5595,T42 P19 D14 confluence GDF5 250ng/ml + TGFb3 10ng/ml,,

iPSC,BIOTIME,GPL10588,MBA_5596,E69 P14 D3 confluence BMP4 10ng/ml ,,

iPSC,BIOTIME,GPL10588,MBA_5597,E69 P14 D3 confluence BMP4 25ng/ml ,,

iPSC,BIOTIME,GPL10588,MBA_5598,E69 P14 D3 confluence BMP4 100ng/ml ,,

iPSC,BIOTIME,GPL10588,MBA_5599,E69 P14 D3 confluence BMP4 10ng/ml + TGFb3 10ng/ml,,

iPSC,BIOTIME,GPL10588,MBA_5600,E69 P14 D3 confluence BMP4 25ng/ml + TGFb3 10ng/ml,,

iPSC,BIOTIME,GPL10588,MBA_5601,E69 P14 D3 confluence BMP4 100ng/ml + TGFb3 10ng/ml,,

iPSC,BIOTIME,GPL10588,MBA_5602,E69 P14 D3 confluence GDF5 100ng/ml ,,

iPSC,BIOTIME,GPL10588,MBA_5603,E69 P14 D3 confluence GDF5 250ng/ml ,,

iPSC,BIOTIME,GPL10588,MBA_5604,E69 P14 D3 confluence GDF5 100ng/ml + TGFb3 10ng/ml,,

iPSC,BIOTIME,GPL10588,MBA_5605,E69 P14 D3 confluence GDF5 250ng/ml + TGFb3 10ng/ml,,

iPSC,BIOTIME,GPL10588,MBA_5606,E69 P14 D7 confluence BMP4 10ng/ml ,,

iPSC,BIOTIME,GPL10588,MBA_5607,E69 P14 D7 confluence BMP4 25ng/ml ,,

iPSC,BIOTIME,GPL10588,MBA_5608,E69 P14 D7 confluence BMP4 100ng/ml ,,

iPSC,BIOTIME,GPL10588,MBA_5609,E69 P14 D7 confluence BMP4 10ng/ml + TGFb3 10ng/ml,,

iPSC,BIOTIME,GPL10588,MBA_5610,E69 P14 D7 confluence BMP4 25ng/ml + TGFb3 10ng/ml,,

iPSC,BIOTIME,GPL10588,MBA_5611,E69 P14 D7 confluence BMP4 100ng/ml + TGFb3 10ng/ml,,

iPSC,BIOTIME,GPL10588,MBA_5612,E69 P14 D7 confluence GDF5 100ng/ml ,,

iPSC,BIOTIME,GPL10588,MBA_5613,E69 P14 D7 confluence GDF5 250ng/ml ,,

iPSC,BIOTIME,GPL10588,MBA_5614,E69 P14 D7 confluence GDF5 100ng/ml + TGFb3 10ng/ml,,

iPSC,BIOTIME,GPL10588,MBA_5615,E69 P14 D7 confluence GDF5 250ng/ml + TGFb3 10ng/ml,,

iPSC,BIOTIME,GPL10588,MBA_5616,E69 P14 D14 confluence BMP4 10ng/ml ,,

iPSC,BIOTIME,GPL10588,MBA_5617,E69 P14 D14 confluence BMP4 25ng/ml ,,

iPSC,BIOTIME,GPL10588,MBA_5618,E69 P14 D14 confluence BMP4 100ng/ml ,,

iPSC,BIOTIME,GPL10588,MBA_5619,E69 P14 D14 confluence BMP4 10ng/ml + TGFb3 10ng/ml,,

iPSC,BIOTIME,GPL10588,MBA_5620,E69 P14 D14 confluence BMP4 25ng/ml + TGFb3 10ng/ml,,

iPSC,BIOTIME,GPL10588,MBA_5621,E69 P14 D14 confluence BMP4 100ng/ml + TGFb3 10ng/ml,,

iPSC,BIOTIME,GPL10588,MBA_5622,E69 P14 D14 confluence GDF5 100ng/ml ,,

iPSC,BIOTIME,GPL10588,MBA_5623,E69 P14 D14 confluence GDF5 250ng/ml ,,

iPSC,BIOTIME,GPL10588,MBA_5624,E69 P14 D14 confluence GDF5 100ng/ml + TGFb3 10ng/ml,,

iPSC,BIOTIME,GPL10588,MBA_5625,E69 P14 D14 confluence GDF5 250ng/ml + TGFb3 10ng/ml,,

AC,BIOTIME,GPL10588,MBA_5626,hMSC P6 D14 confluence BMP2 50ng/ml + TGFb3 10ng/ml,,

AC,BIOTIME,GPL10588,MBA_5627,hMSC P6 D14 confluence BMP2 50ng/ml + TGFb3 10ng/ml,,

AC,BIOTIME,GPL10588,MBA_5628,hMSC P6 D14 confluence BMP2 50ng/ml + TGFb3 10ng/ml,,

AC,BIOTIME,GPL10588,MBA_5629,hMSC P6 D14 Lanx HA (hydroxyapatite) BMP2 50ng/ml + TGFb3 10ng/ml,,

AC,BIOTIME,GPL10588,MBA_5630,hMSC P6 D14 Lanx HA BMP2 50ng/ml + TGFb3 10ng/ml,,

AC,BIOTIME,GPL10588,MBA_5631,hMSC P6 D14 Lanx HA BMP2 50ng/ml + TGFb3 10ng/ml,,

iPSC,BIOTIME,GPL10588,MBA_5632,SK11 P15 D14 confluence BMP2 50ng/ml + TGFb3 10ng/ml,,

iPSC,BIOTIME,GPL10588,MBA_5633,SK11 P15 D14 confluence BMP2 50ng/ml + TGFb3 10ng/ml,,

iPSC,BIOTIME,GPL10588,MBA_5634,SK11 P15 D14 confluence BMP2 50ng/ml + TGFb3 10ng/ml,,

iPSC,BIOTIME,GPL10588,MBA_5635,SK11 P15 D14 Lanx HA BMP2 50ng/ml + TGFb3 10ng/ml,,

iPSC,BIOTIME,GPL10588,MBA_5636,SK11 P15 D14 Lanx HA BMP2 50ng/ml + TGFb3 10ng/ml,,

iPSC,BIOTIME,GPL10588,MBA_5637,SK11 P15 D14 Lanx HA BMP2 50ng/ml + TGFb3 10ng/ml,,

iPSC,BIOTIME,GPL10588,MBA_5638,SM30 P20 D14 confluence BMP2 50ng/ml + TGFb3,,

iPSC,BIOTIME,GPL10588,MBA_5639,SM30 P20 D14 confluence BMP2 50ng/ml + TGFb3,,

iPSC,BIOTIME,GPL10588,MBA_5640,SM30 P20 D14 confluence BMP2 50ng/ml + TGFb3,,

iPSC,BIOTIME,GPL10588,MBA_5641,SM30 P20 D14 Lanx HA (hydroxyapatite) BMP2 50ng/ml + TGFb3,,

iPSC,BIOTIME,GPL10588,MBA_5642,SM30 P20 D14 Lanx HA BMP2 50ng/ml + TGFb3,,

iPSC,BIOTIME,GPL10588,MBA_5643,SM30 P20 D14 Lanx HA BMP2 50ng/ml + TGFb3,,

iPSC,BIOTIME,GPL10588,MBA_5647,ESI EP004 NP95 EN P9 D14 confluence adipo,,

iPSC,BIOTIME,GPL10588,MBA_5648,ESI EP004 NP95 EN P9 D21 Hystem BMP4 10ng/ml + TGFb3 10ng/ml,,

iPSC,BIOTIME,GPL10588,MBA_5649,ESI EP004 NP95 EN P9 D21 Hystem BMP4 10ng/ml ,,

iPSC,BIOTIME,GPL10588,MBA_5650,ESI EP004 NP95 EN P9 D21 Hystem BMP2 50ng/ml + TGFb3 10ng/ml,,

iPSC,BIOTIME,GPL10588,MBA_5651,ESI EP004 NP95 EN P9 D21 Hystem RA 1uM,,

iPSC,BIOTIME,GPL10588,MBA_5652,ESI EP004 NP95EN P9 D21 Hystem osteo (no serum),,

iPSC,BIOTIME,GPL10588,MBA_5654,ESI EP004 NP111SM P8 D21 Hystem BMP4 10ng/ml,,

iPSC,BIOTIME,GPL10588,MBA_5655,ESI EP004 NP111SM P8 D21 Hystem BMP4 10ng/ml + TGFb3 10ng/ml,,

iPSC,BIOTIME,GPL10588,MBA_5656,ESI EP004 NP111SM P8 D21 RA 1uM,,

iPSC,BIOTIME,GPL10588,MBA_5657,ESI EP004 NP111SM P8 D21 Hystem BMP2 50ng/ml + TGFb3 10ng/ml,,

iPSC,BIOTIME,GPL10588,MBA_5658,ESI EP004 NP111SM P8 D21 osteo (no serum),,

iPSC,BIOTIME,GPL10588,MBA_5659,ESI EP004 NP111SM P8 D14 confluence adipo,,

iPSC,BIOTIME,GPL10588,MBA_5661,ESI EP004 NP113SM P11 D14 confluence adipo,,

iPSC,BIOTIME,GPL10588,MBA_5662,ESI EP004 NP113SM P11 D21 Hystem BMP4 10ng/ml + TGFb3 10ng/ml,,

iPSC,BIOTIME,GPL10588,MBA_5663,ESI EP004 NP113SM P11 D21 Hystem BMP4 10ng/ml ,,

iPSC,BIOTIME,GPL10588,MBA_5664,ESI EP004 NP113SM P11 D21 Hystem BMP2 50ng/ml + TGFb3 10ng/ml,,

iPSC,BIOTIME,GPL10588,MBA_5665,ESI EP004 NP113SM P11 D21 Hystem RA 1uM,,

iPSC,BIOTIME,GPL10588,MBA_5666,ESI EP004 NP113SM P11 D21 Hystem osteo (no serum),,

iPSC,BIOTIME,GPL10588,MBA_5668,ESI EP004 NP110SM P8 D21 Hystem BMP4 10ng/ml + TGFb3 10ng/ml,,

iPSC,BIOTIME,GPL10588,MBA_5669,ESI EP004 NP110SM P8 D21 Hystem BMP4 10ng/ml ,,

iPSC,BIOTIME,GPL10588,MBA_5670,ESI EP004 NP110SM P8 D21 Hystem RA 1uM,,

iPSC,BIOTIME,GPL10588,MBA_5671,ESI EP004 NP110SM P8 D21 Hystem BMP2 50ng/ml + TGFb3 10ng/ml,,

iPSC,BIOTIME,GPL10588,MBA_5672,ESI EP004 NP110SM P8 D21 Hystem osteo (no serum),,

iPSC,BIOTIME,GPL10588,MBA_5673,ESI EP004 NP110 SM P8 confluence adipo,,

iPSC,BIOTIME,GPL10588,MBA_5675,ESI EP004 NP93 SM P10 D21 Hystem BMP4 10ng/ml + TGFb3 10ng/ml,,

iPSC,BIOTIME,GPL10588,MBA_5676,ESI EP004 NP93 SM P10 D21 Hystem BMP4 10ng/ml,,

iPSC,BIOTIME,GPL10588,MBA_5677,ESI EP004 NP93 SM P10 D21 Hystem BMP2 50ng/ml + TGFb3 10ng/ml,,

iPSC,BIOTIME,GPL10588,MBA_5678,ESI EP004 NP93 SM P10 D21 Hystem RA 1uM,,

iPSC,BIOTIME,GPL10588,MBA_5679,ESI EP004 NP93 SM P10 D21 Hystem osteo (no serum),,

iPSC,BIOTIME,GPL10588,MBA_5680,ESI EP004 NP93 SM P10 D14 confluence adipo,,

iPSC,BIOTIME,GPL10588,MBA_5682,ESI EP004 NP91 SM P9 D21 Hystem BMP4 10ng/ml,,

iPSC,BIOTIME,GPL10588,MBA_5683,ESI EP004 NP91 SM P9 D21 Hystem BMP4 10ng/ml + TGFb3 10ng/ml,,

iPSC,BIOTIME,GPL10588,MBA_5684,ESI EP004 NP91 SM P9 D21 Hystem RA 1uM,,

iPSC,BIOTIME,GPL10588,MBA_5685,ESI EP004 NP91 SM P9 D21 Hystem BMP2 50ng/ml + TGFb3 10ng/ml,,

iPSC,BIOTIME,GPL10588,MBA_5686,ESI EP004 NP91 SM P9 D21 Hystem osteo (no serum),,

iPSC,BIOTIME,GPL10588,MBA_5687,ESI EP004 NP91 SM P9 D14 confluence adipo,,

iPSC,BIOTIME,GPL10588,MBA_5689,ESI EP004 NP92 SM P9 D21 Hystem BMP4 10ng/ml,,

iPSC,BIOTIME,GPL10588,MBA_5690,ESI EP004 NP92 SM P9 D21 Hystem BMP4 10ng/ml + TGFb3 10ng/ml,,

iPSC,BIOTIME,GPL10588,MBA_5691,ESI EP004 NP92 SM P9 D21 Hystem RA 1uM,,

iPSC,BIOTIME,GPL10588,MBA_5692,ESI EP004 NP92 SM P9 D21 Hystem BMP2 50ng/ml + TGFb3 10ng/ml,,

iPSC,BIOTIME,GPL10588,MBA_5693,ESI EP004 NP92 SM P9 D21 Hystem osteo (no serum),,

iPSC,BIOTIME,GPL10588,MBA_5694,ESI EP004 NP92 SM P9 D14 confluence adipo,,

iPSC,BIOTIME,GPL10588,MBA_5696,ESI EP004 NP101 EN P9 D21 Hystem BMP4 10ng/ml + TGFb3 10ng/ml,,

iPSC,BIOTIME,GPL10588,MBA_5697,ESI EP004 NP101 EN P9 D21 Hystem BMP4 10ng/ml ,,

iPSC,BIOTIME,GPL10588,MBA_5698,ESI EP004 NP101 EN P9 D21 Hystem BMP2 50ng/ml + TGFb3 10ng/ml,,

iPSC,BIOTIME,GPL10588,MBA_5699,ESI EP004 NP101 EN P9 D21 Hystem RA 1uM,,

iPSC,BIOTIME,GPL10588,MBA_5700,ESI EP004 NP101 EN P9 D21 Hystem osteo (no serum),,

iPSC,BIOTIME,GPL10588,MBA_5701,ESI EP004 NP101 EN P9 confluence adipo,,

iPSC,BIOTIME,GPL10588,MBA_5703,ESI EP004 NP90 SM P11 D21 Hystem BMP4 10ng/ml + TGFb3 10ng/ml,,

iPSC,BIOTIME,GPL10588,MBA_5704,ESI EP004 NP90 SM P11 D21 Hystem BMP4 10ng/ml,,

iPSC,BIOTIME,GPL10588,MBA_5705,ESI EP004 NP90 SM P11 D21 Hystem BMP2 50ng/ml + TGFb3 10ng/ml,,

iPSC,BIOTIME,GPL10588,MBA_5706,ESI EP004 NP90 SM P11 D21 RA 1uM,,

iPSC,BIOTIME,GPL10588,MBA_5707,ESI EP004 NP90 SM P11 D21 Hystem osteo (no serum),,

iPSC,BIOTIME,GPL10588,MBA_5708,RAD20.5 P19 D14 MM TGFb3 10ng/ml,,

iPSC,BIOTIME,GPL10588,MBA_5709,RAD20.5 P19 D14 MM BMP2 50ng/ml + TGFb3 10ng/ml,,

iPSC,BIOTIME,GPL10588,MBA_5710,RAD20.5 P19 D14 MM BMP7 100ng/ml + TGFb3 10ng/ml,,

iPSC,BIOTIME,GPL10588,MBA_5711,RAD20.5 P19 D14 MM GDF5 100ng/ml + TGFb3 10ng/ml,,

iPSC,BIOTIME,GPL10588,MBA_5712,RAD20.5 P19 D14 MM PD173074 FGF inhib 50nM,,

iPSC,BIOTIME,GPL10588,MBA_5713,RAD20.5 P19 D14 MM PD173074 50nM + BMP7 100ng/ml + TGFb3 10ng/ml,,

iPSC,BIOTIME,GPL10588,MBA_5714,RAD20.5 P19 D21 MM TGFb3 10ng/ml,,

iPSC,BIOTIME,GPL10588,MBA_5715,RAD20.5 P19 D21 MM BMP2 50ng/ml + TGFb3 10ng/ml,,

iPSC,BIOTIME,GPL10588,MBA_5716,RAD20.5 P19 D21 MM BMP7 100ng/ml + TGFb3 10ng/ml,,

iPSC,BIOTIME,GPL10588,MBA_5717,RAD20.5 P19 D21 MM GDF5 100ng/ml + TGFb3 10ng/ml,,

iPSC,BIOTIME,GPL10588,MBA_5718,RAD20.5 P19 D21 MM PD173074 FGF inhib 50nM,,

iPSC,BIOTIME,GPL10588,MBA_5719,RAD20.5 P19 D21 MM PD173074 50nM + BMP7 100ng/ml + TGFb3 10ng/ml,,

iPSC,BIOTIME,GPL10588,MBA_5721,E3 P14 D21 Hystem BMP4 50ng/ml,,

iPSC,BIOTIME,GPL10588,MBA_5722,E3 P14 D21 Hystem BMP4 10ng/ml,,

iPSC,BIOTIME,GPL10588,MBA_5723,E3 P14 D21 Hystem Rosiglitazone 1uM ( PPAR? agonist),,

iPSC,BIOTIME,GPL10588,MBA_5724,E3 P14 D21 Hystem Rosiglitazone 1uM + BMP4 10ng/ml,,

iPSC,BIOTIME,GPL10588,MBA_5725,E3 P14 D21 Hystem T0070907 10nM (PPAR? antagonist),,

iPSC,BIOTIME,GPL10588,MBA_5726,E3 P14 D21 Hystem T0070907 10nM (PPAR? antagonist) + BMP4 10ng/ml,,

iPSC,BIOTIME,GPL10588,MBA_5727,E3 P14 D21 Hystem BMP7 100ng/ml,,

iPSC,BIOTIME,GPL10588,MBA_5728,E3 P14 D21 Hystem BMP7 100ng/ml + Rosiglitazone 1uM,,

iPSC,BIOTIME,GPL10588,MBA_5729,E3 P14 D21 Hystem adipo,,

iPSC,BIOTIME,GPL10588,MBA_5730,E3 P14 D21 Hystem adipo + Rosiglitazone 1uM,,

iPSC,BIOTIME,GPL10588,MBA_5731,E3 P14 D21 confluence BMP4 50ng/ml,,

iPSC,BIOTIME,GPL10588,MBA_5732,E3 P14 D21 confluence BMP4 10ng/ml,,

iPSC,BIOTIME,GPL10588,MBA_5733,E3 P14 D21 confluence Rosiglitazone 1uM ( PPAR? agonist),,

iPSC,BIOTIME,GPL10588,MBA_5734,E3 P14 D21 confluence Rosiglitazone 1uM + BMP4 10ng/ml,,

iPSC,BIOTIME,GPL10588,MBA_5735,E3 P14 D21 confluence T0070907 10nM (PPAR? antagonist),,

iPSC,BIOTIME,GPL10588,MBA_5736,E3 P14 D21 confluence T0070907 10nM (PPAR? antagonist) + BMP4 10ng/ml,,

iPSC,BIOTIME,GPL10588,MBA_5737,E3 P14 D21 confluence BMP7 100ng/ml,,

iPSC,BIOTIME,GPL10588,MBA_5738,E3 P14 D21 confluence BMP7 100ng/ml + Rosiglitazone 1uM,,

iPSC,BIOTIME,GPL10588,MBA_5739,E3 P15 D20 Hystem adipo,,

iPSC,BIOTIME,GPL10588,MBA_5740,E3 P15 D20 Hystem adipo + Rosiglitazone 1uM,,

AC,BIOTIME,GPL10588,MBA_5741,hMSC P8 D7 confluence BMP4 10ng/ml (control for adipose expt),,

AC,BIOTIME,GPL10588,MBA_5742,hMSC P8 D7 confluence BMP7 100ng/ml (control for adipose expt),,

AC,BIOTIME,GPL10588,MBA_5743,hMSC P8 D7 confluence adipo,,

AC,BIOTIME,GPL10588,MBA_5744,hMSC P8 D14 confluence BMP4 10ng/ml,,

AC,BIOTIME,GPL10588,MBA_5745,hMSC P8 D14 confluence BMP7 100ng/ml,,

AC,BIOTIME,GPL10588,MBA_5746,hMSC P8 D14 confluence adipo,,

iPSC,BIOTIME,GPL10588,MBA_5748,4D20.8 P19 D7 neuroscreen 1,,

iPSC,BIOTIME,GPL10588,MBA_5749,4D20.8 P19 D7 neuroscreen 2,,

iPSC,BIOTIME,GPL10588,MBA_5750,4D20.8 P19 D7 neuroscreen 3,,

iPSC,BIOTIME,GPL10588,MBA_5751,4D20.8 P19 D7 neuroscreen 4,,

iPSC,BIOTIME,GPL10588,MBA_5752,4D20.8 P19 D7 neuroscreen 5,,

iPSC,BIOTIME,GPL10588,MBA_5754,E68 P18 D3 neuroscreen 1,,

iPSC,BIOTIME,GPL10588,MBA_5755,E68 P18 D3 neuroscreen 2,,

iPSC,BIOTIME,GPL10588,MBA_5756,E68 P18 D3 neuroscreen 3,,

iPSC,BIOTIME,GPL10588,MBA_5757,E68 P18 D3 neuroscreen 4,,

iPSC,BIOTIME,GPL10588,MBA_5758,E68 P18 D3 neuroscreen 5,,

iPSC,BIOTIME,GPL10588,MBA_5760,E69 P13 D4 neuroscreen 1,,

iPSC,BIOTIME,GPL10588,MBA_5761,E69 P13 D4 neuroscreen 2,,

iPSC,BIOTIME,GPL10588,MBA_5762,E69 P13 D4 neuroscreen 3,,

iPSC,BIOTIME,GPL10588,MBA_5763,E69 P13 D4 neuroscreen 4,,

iPSC,BIOTIME,GPL10588,MBA_5764,E69 P13 D4 neuroscreen 5,,

iPSC,BIOTIME,GPL10588,MBA_5766,CM02 P16 D2 neuroscreen 1,,

iPSC,BIOTIME,GPL10588,MBA_5767,CM02 P16 D2 neuroscreen 2,,

iPSC,BIOTIME,GPL10588,MBA_5768,CM02 P16 D2 neuroscreen 3,,

iPSC,BIOTIME,GPL10588,MBA_5769,CM02 P16 D2 neuroscreen 4,,

iPSC,BIOTIME,GPL10588,MBA_5770,CM02 P16 D2 neuroscreen 5,,

iPSC,BIOTIME,GPL10588,MBA_5772,T44 P10 D14 neuroscreen 1,,

iPSC,BIOTIME,GPL10588,MBA_5773,T44 P10 D14 neuroscreen 2,,

iPSC,BIOTIME,GPL10588,MBA_5774,T44 P10 D14 neuroscreen 3,,

iPSC,BIOTIME,GPL10588,MBA_5775,T44 P10 D14 neuroscreen 4,,

iPSC,BIOTIME,GPL10588,MBA_5776,T44 P10 D14 neuroscreen 5,,

iPSC,BIOTIME,GPL10588,MBA_5778,T42 P18 D7 neuroscreen 1,,

iPSC,BIOTIME,GPL10588,MBA_5779,T42 P18 D7 neuroscreen 2,,

iPSC,BIOTIME,GPL10588,MBA_5780,T42 P18 D7 neuroscreen 3,,

iPSC,BIOTIME,GPL10588,MBA_5781,T42 P18 D7 neuroscreen 4,,

iPSC,BIOTIME,GPL10588,MBA_5782,T42 P18 D7 neuroscreen 5,,

iPSC,BIOTIME,GPL10588,MBA_5784,RAD20.5 P19 D6 neuroscreen 1,,

iPSC,BIOTIME,GPL10588,MBA_5785,RAD20.5 P19 D6 neuroscreen 2,,

iPSC,BIOTIME,GPL10588,MBA_5786,RAD20.5 P19 D6 neuroscreen 3,,

iPSC,BIOTIME,GPL10588,MBA_5787,RAD20.5 P19 D7 neuroscreen 4,,

iPSC,BIOTIME,GPL10588,MBA_5788,RAD20.5 P19 D6 neuroscreen 5,,

iPSC,BIOTIME,GPL10588,MBA_5790,RAD20.24 P12 D3 neuroscreen 1,,

iPSC,BIOTIME,GPL10588,MBA_5791,RAD20.24 P12 D3 neuroscreen 2,,

iPSC,BIOTIME,GPL10588,MBA_5792,RAD20.24 P12 D3 neuroscreen 3,,

iPSC,BIOTIME,GPL10588,MBA_5793,RAD20.24 P12 D4 neuroscreen 4,,

iPSC,BIOTIME,GPL10588,MBA_5794,RAD20.24 P12 D3 neuroscreen 5,,

iPSC,BIOTIME,GPL10588,MBA_5796,E44 P18 D7 neuroscreen 1,,

iPSC,BIOTIME,GPL10588,MBA_5797,E44 P18 D7 neuroscreen 2,,

iPSC,BIOTIME,GPL10588,MBA_5798,E44 P18 D7 neuroscreen 3,,

iPSC,BIOTIME,GPL10588,MBA_5799,E44 P18 D7 neuroscreen 4,,

iPSC,BIOTIME,GPL10588,MBA_5800,E44 P18 D7 neuroscreen 5,,

iPSC,BIOTIME,GPL10588,MBA_5802,RASKEL8 P11 D21 Hystem BMP4 10ng/ml,,

iPSC,BIOTIME,GPL10588,MBA_5803,RASKEL8 P11 D21 Hystem BMP4 10ng/ml + TGFb3 10ng/ml,,

iPSC,BIOTIME,GPL10588,MBA_5804,RASKEL8 P11 D21 Hystem BMP2 50ng/ml + TGFb3 10ng/ml,,

iPSC,BIOTIME,GPL10588,MBA_5805,RASKEL8 P11 D21 Hystem osteo (no serum),,

iPSC,BIOTIME,GPL10588,MBA_5807,RASKEL9 P12 D21 Hystem BMP4 10ng/ml ,,

iPSC,BIOTIME,GPL10588,MBA_5808,RASKEL9 P12 D21 Hystem BMP4 10ng/ml + TGFb3 10ng/ml,,

iPSC,BIOTIME,GPL10588,MBA_5809,RASKEL9 P12 D21 Hystem BMP2 50ng/ml + TGFb3 10ng/ml,,

iPSC,BIOTIME,GPL10588,MBA_5810,RASKEL9 P12 D21 Hystem osteo (no serum),,

X,BIOTIME,GPL10588,MBA_5811,Total Human Reference RNA CloneTech,,

iPSC,BIOTIME,GPL10588,MBA_5813,EN42 P12 D7 neuroscreen 1,,

iPSC,BIOTIME,GPL10588,MBA_5814,EN42 P12 D7 neuroscreen 2,,

iPSC,BIOTIME,GPL10588,MBA_5815,EN42 P12 D7 neuroscreen 3,,

iPSC,BIOTIME,GPL10588,MBA_5816,EN42 P12 D7 neuroscreen 4,,

iPSC,BIOTIME,GPL10588,MBA_5817,EN42 P12 D7 neuroscreen 5,,

iPSC,BIOTIME,GPL10588,MBA_5819,RAD20.16 P12 D7 neuroscreen 1,,

iPSC,BIOTIME,GPL10588,MBA_5820,RAD20.16 P12 D4 neuroscreen 2,,

iPSC,BIOTIME,GPL10588,MBA_5821,RAD20.16 P12 D7 neuroscreen 3,,

iPSC,BIOTIME,GPL10588,MBA_5822,RAD20.16 P12 D7 neuroscreen 4,,

iPSC,BIOTIME,GPL10588,MBA_5823,RAD20.16 P12 D7 neuroscreen 5,,

iPSC,BIOTIME,GPL10588,MBA_5825,RAD20.6 P16 D7 neuroscreen 1,,

iPSC,BIOTIME,GPL10588,MBA_5826,RAD20.6 P16 D7 neuroscreen 2,,

iPSC,BIOTIME,GPL10588,MBA_5827,RAD20.6 P16 D7 neuroscreen 3,,

iPSC,BIOTIME,GPL10588,MBA_5828,RAD20.6 P16 D11 neuroscreen 4,,

iPSC,BIOTIME,GPL10588,MBA_5829,RAD20.6 P16 D7 neuroscreen 5,,

iPSC,BIOTIME,GPL10588,MBA_5831,EN7 P11 D14 neuroscreen 1,,

iPSC,BIOTIME,GPL10588,MBA_5832,EN7 P11 D14 neuroscreen 2,,

iPSC,BIOTIME,GPL10588,MBA_5833,EN7 P11 D14 neuroscreen 3,,

iPSC,BIOTIME,GPL10588,MBA_5834,EN7 P11 D14 neuroscreen 4,,

iPSC,BIOTIME,GPL10588,MBA_5835,EN7 P11 D14 neuroscreen 5,,

iPSC,BIOTIME,GPL10588,MBA_5837,T7 P12 D4 neuroscreen 1,,

iPSC,BIOTIME,GPL10588,MBA_5838,T7 P12 D4 neuroscreen 2,,

iPSC,BIOTIME,GPL10588,MBA_5839,T7 P12 D4 neuroscreen 3,,

iPSC,BIOTIME,GPL10588,MBA_5840,T7 P12 D4 neuroscreen 4,,

iPSC,BIOTIME,GPL10588,MBA_5841,T7 P12 D4 neuroscreen 5,,

iPSC,BIOTIME,GPL10588,MBA_5843,E15 P20 neuroscreen 1,,

iPSC,BIOTIME,GPL10588,MBA_5844,E15 P20 neuroscreen 2,,

iPSC,BIOTIME,GPL10588,MBA_5845,E15 P20 neuroscreen 3,,

iPSC,BIOTIME,GPL10588,MBA_5846,E15 P20 neuroscreen 4,,

iPSC,BIOTIME,GPL10588,MBA_5847,E15 P20 neuroscreen 5,,

iPSC,BIOTIME,GPL10588,MBA_5849,T14 P11 D10 neuroscreen 1,,

iPSC,BIOTIME,GPL10588,MBA_5850,T14 P11 D10 neuroscreen 2,,

iPSC,BIOTIME,GPL10588,MBA_5851,T14 P11 D10 neuroscreen 3,,

iPSC,BIOTIME,GPL10588,MBA_5852,T14 P11 D10 neuroscreen 4,,

iPSC,BIOTIME,GPL10588,MBA_5853,T14 P11 D10 neuroscreen 5,,

iPSC,BIOTIME,GPL10588,MBA_5855,T20 P14 D3 neuroscreen 1,,

iPSC,BIOTIME,GPL10588,MBA_5856,T20 P14 D3 neuroscreen 2,,

iPSC,BIOTIME,GPL10588,MBA_5857,T20 P14 D3 neuroscreen 3,,

iPSC,BIOTIME,GPL10588,MBA_5858,T20 P14 D3 neuroscreen 4,,

iPSC,BIOTIME,GPL10588,MBA_5859,T20 P14 D3 neuroscreen 5,,

iPSC,BIOTIME,GPL10588,MBA_5861,U18 P15 D4 neuroscreen 1,,

iPSC,BIOTIME,GPL10588,MBA_5862,U18 P15 D4 neuroscreen 2,,

iPSC,BIOTIME,GPL10588,MBA_5863,U18 P15 D4 neuroscreen 3,,

iPSC,BIOTIME,GPL10588,MBA_5864,U18 P15 D4 neuroscreen 4,,

iPSC,BIOTIME,GPL10588,MBA_5865,U18 P15 D4 neuroscreen 5,,

iPSC,BIOTIME,GPL10588,MBA_5867,W11 P11 D14 neuroscreen 1,,

iPSC,BIOTIME,GPL10588,MBA_5868,W11 P11 D14 neuroscreen 2,,

iPSC,BIOTIME,GPL10588,MBA_5869,W11 P11 D14 neuroscreen 3,,

iPSC,BIOTIME,GPL10588,MBA_5870,W11 P11 D14 neuroscreen 4,,

iPSC,BIOTIME,GPL10588,MBA_5871,W11 P11 D14 neuroscreen 5,,

iPSC,BIOTIME,GPL10588,MBA_5873,U31 P14 D7 neuroscreen 1,,

iPSC,BIOTIME,GPL10588,MBA_5874,U31 P14 D7 neuroscreen 2,,

iPSC,BIOTIME,GPL10588,MBA_5875,U31 P14 D7 neuroscreen 3,,

iPSC,BIOTIME,GPL10588,MBA_5876,U31 P14 D11 neuroscreen 4,,

iPSC,BIOTIME,GPL10588,MBA_5877,U31 P14 D7 neuroscreen 5,,

iPSC,BIOTIME,GPL10588,MBA_5879,E164 P20 D14 neuroscreen 1,,

iPSC,BIOTIME,GPL10588,MBA_5880,E164 P20 D14 neuroscreen 2,,

iPSC,BIOTIME,GPL10588,MBA_5881,E164 P20 D14 neuroscreen 3,,

iPSC,BIOTIME,GPL10588,MBA_5882,E164 P20 D7 neuroscreen 4,,

iPSC,BIOTIME,GPL10588,MBA_5883,E164 P20 D5 neuroscreen 5,,

iPSC,BIOTIME,GPL10588,MBA_5885,Z1 P14 D7 neuroscreen 1,,

iPSC,BIOTIME,GPL10588,MBA_5886,Z1 P14 D7 neuroscreen 2,,

iPSC,BIOTIME,GPL10588,MBA_5887,Z1 P14 D7 neuroscreen 3,,

iPSC,BIOTIME,GPL10588,MBA_5888,Z1 P14 D7 neuroscreen 4,,

iPSC,BIOTIME,GPL10588,MBA_5889,Z1 P14 D7 neuroscreen 5,,

iPSC,BIOTIME,GPL10588,MBA_5891,Z11 P11 D14 neuroscreen 1,,

iPSC,BIOTIME,GPL10588,MBA_5892,Z11 P11 D14 neuroscreen 2,,

iPSC,BIOTIME,GPL10588,MBA_5893,Z11 P11 D14 neuroscreen 3,,

iPSC,BIOTIME,GPL10588,MBA_5894,Z11 P11 D14 neuroscreen 4,,

iPSC,BIOTIME,GPL10588,MBA_5895,Z11 P11 D14 neuroscreen 5,,

iPSC,BIOTIME,GPL10588,MBA_5897,E111 P12 D4 neuroscreen 1,,

iPSC,BIOTIME,GPL10588,MBA_5898,E111 P12 D4 neuroscreen 2,,

iPSC,BIOTIME,GPL10588,MBA_5899,E111 P12 D4 neuroscreen 3,,

iPSC,BIOTIME,GPL10588,MBA_5900,E111 P12 D4 neuroscreen 4,,

iPSC,BIOTIME,GPL10588,MBA_5901,E111 P12 D4 neuroscreen 5,,

iPSC,BIOTIME,GPL10588,MBA_5903,E169 P16 D6 neuroscreen 1,,

iPSC,BIOTIME,GPL10588,MBA_5904,E169 P16 D6 neuroscreen 2,,

iPSC,BIOTIME,GPL10588,MBA_5905,E169 P16 D6 neuroscreen 3,,

iPSC,BIOTIME,GPL10588,MBA_5906,E169 P16 D5 neuroscreen 4,,

iPSC,BIOTIME,GPL10588,MBA_5907,E169 P16 D5 neuroscreen 5,,

iPSC,BIOTIME,GPL10588,MBA_5909,E33 P11 D1 neuroscreen 1,,

iPSC,BIOTIME,GPL10588,MBA_5910,E33 P11 D1 neuroscreen 2,,

iPSC,BIOTIME,GPL10588,MBA_5911,E33 P11 D1 neuroscreen 3,,

iPSC,BIOTIME,GPL10588,MBA_5912,E33 P11 D1 neuroscreen 4,,

iPSC,BIOTIME,GPL10588,MBA_5913,E33 P11 D1 neuroscreen 5,,

iPSC,BIOTIME,GPL10588,MBA_5915,4D20.9 P14 D7 neuroscreen 1,,

iPSC,BIOTIME,GPL10588,MBA_5916,4D20.9 P14 D7 neuroscreen 2,,

iPSC,BIOTIME,GPL10588,MBA_5917,4D20.9 P14 D7 neuroscreen 3,,

iPSC,BIOTIME,GPL10588,MBA_5918,4D20.9 P14 D4 neuroscreen 4,,

iPSC,BIOTIME,GPL10588,MBA_5919,4D20.9 P14 D4 neuroscreen 5,,

iPSC,BIOTIME,GPL10588,MBA_5921,ESI EP004 NP92 SM P10 D3 neuroscreen 1,,

iPSC,BIOTIME,GPL10588,MBA_5922,ESI EP004 NP92 SM P10 D3 neuroscreen 2,,

iPSC,BIOTIME,GPL10588,MBA_5923,ESI EP004 NP92 SM P10 D3 neuroscreen 3,,

iPSC,BIOTIME,GPL10588,MBA_5924,ESI EP004 NP92 SM P10 D3 neuroscreen 4,,

iPSC,BIOTIME,GPL10588,MBA_5925,ESI EP004 NP92 SM P10 D3 neuroscreen 5,,

iPSC,BIOTIME,GPL10588,MBA_5927,ESI EP004 NP91 SM P10 D3 neuroscreen 1,,

iPSC,BIOTIME,GPL10588,MBA_5928,ESI EP004 NP91 SM P10 D3 neuroscreen 2,,

iPSC,BIOTIME,GPL10588,MBA_5929,ESI EP004 NP91 SM P10 D3 neuroscreen 3,,

iPSC,BIOTIME,GPL10588,MBA_5930,ESI EP004 NP91 SM P10 D3 neuroscreen 4,,

iPSC,BIOTIME,GPL10588,MBA_5931,ESI EP004 NP91 SM P10 D3 neuroscreen 5,,

iPSC,BIOTIME,GPL10588,MBA_5933,7PEND24 P19 D14 neuroscreen 1,,

iPSC,BIOTIME,GPL10588,MBA_5934,7PEND24 P19 D14 neuroscreen 2,,

iPSC,BIOTIME,GPL10588,MBA_5935,7PEND24 P19 D14 neuroscreen 3,,

iPSC,BIOTIME,GPL10588,MBA_5936,7PEND24 P19 D14 neuroscreen 4,,

iPSC,BIOTIME,GPL10588,MBA_5937,7PEND24 P19 D14 neuroscreen 5,,

iPSC,BIOTIME,GPL10588,MBA_5939,7SMOO32 P11 D14 neuroscreen 1,,

iPSC,BIOTIME,GPL10588,MBA_5940,7SMOO32 P11 D14 neuroscreen 2,,

iPSC,BIOTIME,GPL10588,MBA_5941,7SMOO32 P11 D14 neuroscreen 3,,

iPSC,BIOTIME,GPL10588,MBA_5942,7SMOO32 P11 D14 neuroscreen 4,,

iPSC,BIOTIME,GPL10588,MBA_5943,7SMOO32 P11 D14 neuroscreen 5,,

iPSC,BIOTIME,GPL10588,MBA_5945,EN8 P11 D14 neuroscreen 1,,

iPSC,BIOTIME,GPL10588,MBA_5946,EN8 P11 D14 neuroscreen 2,,

iPSC,BIOTIME,GPL10588,MBA_5947,EN8 P11 D14 neuroscreen 3,,

iPSC,BIOTIME,GPL10588,MBA_5948,EN8 P11 D14 neuroscreen 4,,

iPSC,BIOTIME,GPL10588,MBA_5949,EN8 P11 D14 neuroscreen 5,,

iPSC,BIOTIME,GPL10588,MBA_5950,4D20.8 P19 D14 confluence BMP4 10ng/ml + TGFb3 10ng/ml,,

iPSC,BIOTIME,GPL10588,MBA_5951,4D20.8 P19 D14 confluence BMP4 10ng/ml + TGFb3 10ng/ml,,

iPSC,BIOTIME,GPL10588,MBA_5952,4D20.8 P19 D14 MC BMP4 10ng/ml + TGFb3 10ng/ml,,

iPSC,BIOTIME,GPL10588,MBA_5953,4D20.8 P19 D14 MC BMP4 10ng/ml + TGFb3 10ng/ml,,

iPSC,BIOTIME,GPL10588,MBA_5954,SM30 P18 D14 confluence BMP4 10ng/ml + TGFb3 10ng/ml,,

iPSC,BIOTIME,GPL10588,MBA_5955,SM30 P18 D14 confluence BMP4 10ng/ml + TGFb3 10ng/ml,,

iPSC,BIOTIME,GPL10588,MBA_5956,SM30 P18 D14 MC BMP4 10ng/ml + TGFb3 10ng/ml,,

iPSC,BIOTIME,GPL10588,MBA_5957,SM30 P18 D14 MC BMP4 10ng/ml + TGFb3 10ng/ml,,

iPSC,BIOTIME,GPL10588,MBA_5958,SM30 P18 D14 MC BMP4 10ng/ml + TGFb3 10ng/ml cryo 7D post thaw in same medium,,

iPSC,BIOTIME,GPL10588,MBA_5967,SM30 P14 ctrl,,

iPSC,BIOTIME,GPL10588,MBA_5968,SM30-R14 P14 ctrl,,

iPSC,BIOTIME,GPL10588,MBA_5969,SM30-R21 P8 ctrl,,

iPSC,BIOTIME,GPL10588,MBA_5970,SM30-R26 P10 ctrl,,

iPSC,BIOTIME,GPL10588,MBA_5971,SM30-R27 P8 ctrl,,

iPSC,BIOTIME,GPL10588,MBA_5991,RAD20.16 P13 D21 Hystem BMP4 10ng/ml + TGFb3 10ng/ml,,

iPSC,BIOTIME,GPL10588,MBA_5993,SM30 P20 D7 collagen sponge BMP2 50ng/ml + TGFb3 10ng/ml +bGP 10mM,,

iPSC,BIOTIME,GPL10588,MBA_5994,SM30 P20 D7 collagen sponge BMP2 50ng/ml + TGFb3 10ng/ml +bGP 10mM,,

iPSC,BIOTIME,GPL10588,MBA_5996,MEL2 P16 D7 collagen sponge BMP2 50ng/ml + TGFb3 10ng/ml +bGP 10mM,,

iPSC,BIOTIME,GPL10588,MBA_5997,MEL2 P16 D7 collagen sponge BMP2 50ng/ml + TGFb3 10ng/ml +bGP 10mM,,

iPSC,BIOTIME,GPL10588,MBA_5998,SM30 P20 D14 collagen sponge BMP2 50ng/ml + TGFb3 10ng/ml + bglycerophosphate 10mM,,

iPSC,BIOTIME,GPL10588,MBA_5999,MEL2 P18 D14 collagen sponge BMP2 50ng/ml + TGFb3 10ng/ml + bglycerophosphate 10mM,,

iPSC,BIOTIME,GPL10588,MBA_6000,SM30 P20 D21 collagen sponge BMP2 50ng/ml + TGFb3 10ng/ml + bglycerophosphate 10mM,,

iPSC,BIOTIME,GPL10588,MBA_6001,MEL2 P16 D21 colagen sponge BMP2 50ng/ml + TGFb3 10ng/ml + bglycerophosphate 10mM,,

iPSC,BIOTIME,GPL10588,MBA_6003,ESI EP004 NP110 SM P9 D4 neuroscreen 1,,

iPSC,BIOTIME,GPL10588,MBA_6004,ESI EP004 NP110 SM P9 D4 neuroscreen 2,,

iPSC,BIOTIME,GPL10588,MBA_6005,ESI EP004 NP110 SM P9 D4 neuroscreen 3,,

iPSC,BIOTIME,GPL10588,MBA_6006,ESI EP004 NP110 SM P9 D4 neuroscreen 4,,

iPSC,BIOTIME,GPL10588,MBA_6007,ESI EP004 NP110 SM P9 D4 neuroscreen 5,,

iPSC,BIOTIME,GPL10588,MBA_6009,ESI EP004 NP111 SM P9 D11 neuroscreen 1,,

iPSC,BIOTIME,GPL10588,MBA_6010,ESI EP004 NP111 SM P9 D11 neuroscreen 2,,

iPSC,BIOTIME,GPL10588,MBA_6011,ESI EP004 NP111 SM P9 D11 neuroscreen 3,,

iPSC,BIOTIME,GPL10588,MBA_6012,ESI EP004 NP111 SM P9 D11 neuroscreen 4,,

iPSC,BIOTIME,GPL10588,MBA_6013,ESI EP004 NP111 SM P9 D11 neuroscreen 5,,

iPSC,BIOTIME,GPL10588,MBA_6015,ESI EP004 NP113 SM P10 D1 neuroscreen 1,,

iPSC,BIOTIME,GPL10588,MBA_6016,ESI EP004 NP113 SM P10 D1 neuroscreen 2,,

iPSC,BIOTIME,GPL10588,MBA_6017,ESI EP004 NP113 SM P10 D1 neuroscreen 3,,

iPSC,BIOTIME,GPL10588,MBA_6018,ESI EP004 NP113 SM P10 D1 neuroscreen 4,,

iPSC,BIOTIME,GPL10588,MBA_6019,ESI EP004 NP113 SM P10 D1 neuroscreen 5,,

iPSC,BIOTIME,GPL10588,MBA_6021,RASMO.12 P15 D10 neuroscreen 1,,

iPSC,BIOTIME,GPL10588,MBA_6022,RASMO.12 P15 D10 neuroscreen 2,,

iPSC,BIOTIME,GPL10588,MBA_6023,RASMO.12 P15 D10 neuroscreen 3,,

iPSC,BIOTIME,GPL10588,MBA_6024,RASMO.12 P15 D10 neuroscreen 4,,

iPSC,BIOTIME,GPL10588,MBA_6025,RASMO.12 P15 D10 neuroscreen 5,,

iPSC,BIOTIME,GPL10588,MBA_6027,SM30 P13 D7 neuroscreen 1,,

iPSC,BIOTIME,GPL10588,MBA_6028,SM30 P13 D7 neuroscreen 2,,

iPSC,BIOTIME,GPL10588,MBA_6029,SM30 P13 D7 neuroscreen 3,,

iPSC,BIOTIME,GPL10588,MBA_6030,SM30 P13 D7 neuroscreen 4,,

iPSC,BIOTIME,GPL10588,MBA_6031,SM30 P13 D7 neuroscreen 5,,

iPSC,BIOTIME,GPL10588,MBA_6033,ESI EP004 NP93 SM P9 D9 neuroscreen 1,,

iPSC,BIOTIME,GPL10588,MBA_6034,ESI EP004 NP93 SM P9 D9 neuroscreen 2,,

iPSC,BIOTIME,GPL10588,MBA_6035,ESI EP004 NP93 SM P9 D9 neuroscreen 3,,

iPSC,BIOTIME,GPL10588,MBA_6036,ESI EP004 NP93 SM P9 D9 neuroscreen 4,,

iPSC,BIOTIME,GPL10588,MBA_6037,ESI EP004 NP93 SM P9 D9 neuroscreen 5,,

iPSC,BIOTIME,GPL10588,MBA_6039,SK11 P12 D4 neuroscreen 1,,

iPSC,BIOTIME,GPL10588,MBA_6040,SK11 P12 D4 neuroscreen 2,,

iPSC,BIOTIME,GPL10588,MBA_6041,SK11 P12 D4 neuroscreen 3,,

iPSC,BIOTIME,GPL10588,MBA_6042,SK11 P12 D4 neuroscreen 4,,

iPSC,BIOTIME,GPL10588,MBA_6043,SK11 P12 D4 neuroscreen 5,,

iPSC,BIOTIME,GPL10588,MBA_6045,ESI EP004 NP95 EN P8 D4 neuroscreen 1,,

iPSC,BIOTIME,GPL10588,MBA_6046,ESI EP004 NP95 EN P8 D4 neuroscreen 2,,

iPSC,BIOTIME,GPL10588,MBA_6047,ESI EP004 NP95 EN P8 D4 neuroscreen 3,,

iPSC,BIOTIME,GPL10588,MBA_6048,ESI EP004 NP95 EN P8 D4 neuroscreen 4,,

iPSC,BIOTIME,GPL10588,MBA_6049,ESI EP004 NP95 EN P8 D4 neuroscreen 5,,

iPSC,BIOTIME,GPL10588,MBA_6051,RAD20.16 P13 D14 Hystem BMP4 10ng/ml + TGFb3 10ng/ml,,

iPSC,BIOTIME,GPL10588,MBA_6053,RASMO.19 P17 D14 Hystem BMP4 10ng/ml + TGFb3 10ng/ml,,

iPSC,BIOTIME,GPL10588,MBA_6054,RASMO.19 P19 D21 Hystem BMP4 10ng/ml + TGFb3 10ng/ml,,

iPSC,BIOTIME,GPL10588,MBA_6055,SM30 P20 D21 collagen sponge BMP2 50ng/ml + TGFb3 10ng/ml + bglycerophosphate 10mM,,

iPSC,BIOTIME,GPL10588,MBA_6056,MEL2 P18 D21 collagen sponge BMP2 50ng/ml + TGFb3 10ng/ml + bglycerophosphate 10mM,,

iPSC,BIOTIME,GPL10588,MBA_6058,E3 P17 pretreated fasting ctrl,,

iPSC,BIOTIME,GPL10588,MBA_6059,E3 P17 pretreated D10 Hystem condition D,,

iPSC,BIOTIME,GPL10588,MBA_6060,E3 P17 pretreated D10 Hystem condition D + cAMP,,

iPSC,BIOTIME,GPL10588,MBA_6061,E3 P16 D21 Hystem BMP7 100ng/ml + Rosiglitazone 1uM ,,

iPSC,BIOTIME,GPL10588,MBA_6062,E3 P16 D21 Hystem BMP7 100ng/ml + Rosiglitazone 1uM ,,

iPSC,BIOTIME,GPL10588,MBA_6063,E3 P16 D21 Hystem BMP4 10ng/ml + Rosiglitazone 1uM ,,

iPSC,BIOTIME,GPL10588,MBA_6064,E3 P16 D21 Hystem BMP4 10ng/ml + Rosiglitazone 1uM ,,

iPSC,BIOTIME,GPL10588,MBA_6065,E3 P17 pretreated D21 Hystem BMP7 100ng/ml + Rosiglitazone 1uM ,,

iPSC,BIOTIME,GPL10588,MBA_6066,E3 P17 pretreated D21 Hystem BMP7 100ng/ml + Rosiglitazone 1uM ,,

iPSC,BIOTIME,GPL10588,MBA_6067,E3 P17 D21 Hystem BMP7 100ng/ml + Rosiglitazone 1uM + cocktail condition C,,

iPSC,BIOTIME,GPL10588,MBA_6068,E3 P17 D21 Hystem BMP7 100ng/ml + Rosiglitazone 1uM + cocktail condition C,,

iPSC,BIOTIME,GPL10588,MBA_6070,E72 P14 pretreated fasting ctrl,,

iPSC,BIOTIME,GPL10588,MBA_6071,E72 P14 pretreated D10 Hystem condition D,,

iPSC,BIOTIME,GPL10588,MBA_6072,E72 P14 pretreated D10 Hystem condition D + cAMP,,

iPSC,BIOTIME,GPL10588,MBA_6073,E72 P13 D21 Hystem BMP7 100ng/ml + Rosiglitazone 1uM ,,

iPSC,BIOTIME,GPL10588,MBA_6074,E72 P13 D21 Hystem BMP7 100ng/ml + Rosiglitazone 1uM ,,

iPSC,BIOTIME,GPL10588,MBA_6075,E72 P13 D21 Hystem BMP4 10ng/ml + Rosiglitazone 1uM ,,

iPSC,BIOTIME,GPL10588,MBA_6076,E72 P13 D21 Hystem BMP4 10ng/ml + Rosiglitazone 1uM ,,

iPSC,BIOTIME,GPL10588,MBA_6077,E72 P14 D21 Hystem BMP7 100ng/ml + Rosiglitazone 1uM + cocktail condition C,,

iPSC,BIOTIME,GPL10588,MBA_6078,E72 P14 D21 Hystem BMP7 100ng/ml + Rosiglitazone 1uM + cocktail condition C,,

iPSC,BIOTIME,GPL10588,MBA_6079,E72 P14 pretreated D21 Hystem BMP7 100ng/ml + Rosiglitazone 1uM ,,

iPSC,BIOTIME,GPL10588,MBA_6080,E72 P14 pretreated D21 Hystem BMP7 100ng/ml + Rosiglitazone 1uM ,,

iPSC,BIOTIME,GPL10588,MBA_6082,MEL2 P17 D6 neuroscreen 1,,

iPSC,BIOTIME,GPL10588,MBA_6083,MEL2 P17 D6 neuroscreen 2,,

iPSC,BIOTIME,GPL10588,MBA_6084,MEL2 P17 D6 neuroscreen 3,,

iPSC,BIOTIME,GPL10588,MBA_6085,MEL2 P17 D6 neuroscreen 4,,

iPSC,BIOTIME,GPL10588,MBA_6086,MEL2 P17 D6 neuroscreen 5,,

iPSC,BIOTIME,GPL10588,MBA_6088,C4ELSR14 P12 D7 neuroscreen 1,,

iPSC,BIOTIME,GPL10588,MBA_6089,C4ELSR14 P12 D7 neuroscreen 2,,

iPSC,BIOTIME,GPL10588,MBA_6090,C4ELSR14 P12 D7 neuroscreen 3,,

iPSC,BIOTIME,GPL10588,MBA_6091,C4ELSR14 P12 D7 neuroscreen 4,,

iPSC,BIOTIME,GPL10588,MBA_6092,C4ELSR14 P12 D7 neuroscreen 5,,

iPSC,BIOTIME,GPL10588,MBA_6147,ESI EP001 EN04 P8 D3 neuroscreen 1,,

iPSC,BIOTIME,GPL10588,MBA_6148,ESI EP001 EN04 P8 D3 neuroscreen 2,,

iPSC,BIOTIME,GPL10588,MBA_6149,ESI EP001 EN04 P8 D3 neuroscreen 3,,

iPSC,BIOTIME,GPL10588,MBA_6150,ESI EP001 EN04 P8 D4 neuroscreen 4,,

iPSC,BIOTIME,GPL10588,MBA_6151,ESI EP001 EN04 P8 D4 neuroscreen 5,,

iPSC,BIOTIME,GPL10588,MBA_6153,ESI EP004 NP101 EN P8 D4 neuroscreen 1,,

iPSC,BIOTIME,GPL10588,MBA_6154,ESI EP004 NP101 EN P8 D4 neuroscreen 2,,

iPSC,BIOTIME,GPL10588,MBA_6155,ESI EP004 NP101 EN P8 D4 neuroscreen 3,,

iPSC,BIOTIME,GPL10588,MBA_6156,ESI EP004 NP101 EN P8 D5 neuroscreen 4,,

iPSC,BIOTIME,GPL10588,MBA_6157,ESI EP004 NP101 EN P8 D4 neuroscreen 5,,

iPSC,BIOTIME,GPL10588,MBA_6159,ESI EP004 NP80 EN P8 D4 neuroscreen 1,,

iPSC,BIOTIME,GPL10588,MBA_6160,ESI EP004 NP80 EN P8 D4 neuroscreen 2,,

iPSC,BIOTIME,GPL10588,MBA_6161,ESI EP004 NP80 EN P8 D4 neuroscreen 3,,

iPSC,BIOTIME,GPL10588,MBA_6162,ESI EP004 NP80 EN P8 D4 neuroscreen 4,,

iPSC,BIOTIME,GPL10588,MBA_6163,ESI EP004 NP80 EN P8 D4 neuroscreen 5,,

iPSC,BIOTIME,GPL10588,MBA_6165,ESI EP004 NP90 SM P9 D8 neuroscreen 1,,

iPSC,BIOTIME,GPL10588,MBA_6166,ESI EP004 NP90 SM P9 D8 neuroscreen 2,,

iPSC,BIOTIME,GPL10588,MBA_6167,ESI EP004 NP90 SM P9 D8 neuroscreen 3,,

iPSC,BIOTIME,GPL10588,MBA_6168,ESI EP004 NP90 SM P9 D8 neuroscreen 4,,

iPSC,BIOTIME,GPL10588,MBA_6169,ESI EP004 NP90 SM P9 D8 neuroscreen 5,,

iPSC,BIOTIME,GPL10588,MBA_6170,E15 P18 D10 Hystem cocktail condition 3 with dbcAMP + Rosiglitazone 1uM (pre-treated),,

iPSC,BIOTIME,GPL10588,MBA_6171,E15 P18 D10 Hystem cocktail condition 3 with dbcAMP + BMP7 100ng/ml (pre-treated),,

iPSC,BIOTIME,GPL10588,MBA_6172,E15 P18 D10 Hystem cocktail condition 3 with dbcAMP + BMP7 100ng/ml + Rosigliatzone 1uM (pre-treated),,

iPSC,BIOTIME,GPL10588,MBA_6173,E15 P18 D10 Hystem cocktail condition 3 BMP7 100ng/ml + Rosigliatzone 1uM (pre-treated),,

iPSC,BIOTIME,GPL10588,MBA_6174,E15 P18 D10 Hystem cocktail condition 3 with dbcAMP + BMP7 100ng/ml + Rosigliatzone 1uM,,

iPSC,BIOTIME,GPL10588,MBA_6175,E15 P18 D14 Hystem BMP4 10ng/ml + Rosigliatzone 1uM,,

iPSC,BIOTIME,GPL10588,MBA_6176,E15 P18 D14 Hystem BMP7 100ng/ml + Rosigliatzone 1uM,,

iPSC,BIOTIME,GPL10588,MBA_6178,EN1 P13 D14 Hystem adiposcreen 1,,

iPSC,BIOTIME,GPL10588,MBA_6179,EN1 P13 D14 Hystem adiposcreen 2,,

iPSC,BIOTIME,GPL10588,MBA_6180,EN1 P13 D10 Hystem adiposcreen 3,,

iPSC,BIOTIME,GPL10588,MBA_6181,FAILED uarray EN1 P13 D10 Hystem adiposcreen 4,,

iPSC,BIOTIME,GPL10588,MBA_6182,EN1 P13 D10 Hystem adiposcreen 5,,

iPSC,BIOTIME,GPL10588,MBA_6183,EN1 P13 D10 Hystem adiposcreen 6,,

iPSC,BIOTIME,GPL10588,MBA_6184,EN1 P13 D10 Hystem adiposcreen 7,,

iPSC,BIOTIME,GPL10588,MBA_6185,EN1 P13 D10 Hystem adiposcreen 8,,

iPSC,BIOTIME,GPL10588,MBA_6187,EN7 P13 D14 Hystem adiposcreen 1,,

iPSC,BIOTIME,GPL10588,MBA_6188,EN7 P13 D14 Hystem adiposcreen 2,,

iPSC,BIOTIME,GPL10588,MBA_6189,EN7 P13 D10 Hystem adiposcreen 3,,

iPSC,BIOTIME,GPL10588,MBA_6190,EN7 P13 D10 Hystem adiposcreen 4,,

iPSC,BIOTIME,GPL10588,MBA_6191,EN7 P13 D10 Hystem adiposcreen 5,,

iPSC,BIOTIME,GPL10588,MBA_6192,EN7 P13 D10 Hystem adiposcreen 6,,

iPSC,BIOTIME,GPL10588,MBA_6193,EN7 P13 D10 Hystem adiposcreen 7,,

iPSC,BIOTIME,GPL10588,MBA_6194,EN7 P13 D10 Hystem adiposcreen 8,,

iPSC,BIOTIME,GPL10588,MBA_6195,SM30 P20 D14 Osteo monolayer Incomplete + BMP2 50ng/ml + TGFb3 10ng/ml +bglycerophosphate 10mM with 100nM Dexamethasone,,

iPSC,BIOTIME,GPL10588,MBA_6196,SM30 P20 D14 Osteo monolayer Incomplete + BMP2 50ng/ml + TGFb3 10ng/ml +bglycerophosphate 10mM with 1nM Dexamethasone,,

iPSC,BIOTIME,GPL10588,MBA_6197,SM30 P20 D14 Osteo monolayer Incomplete + BMP2 50ng/ml +bglycerophosphate 10mM with 1nM Dexamethasone,,

iPSC,BIOTIME,GPL10588,MBA_6198,MEL2 P19 D14 Osteo monolayer Incomplete + BMP2 50ng/ml + TGFb3 10ng/ml +bglycerophosphate 10mM with 100nM Dexamethasone,,

iPSC,BIOTIME,GPL10588,MBA_6199,MEL2 P19 D14 Osteo monolayer Incomplete + BMP2 50ng/ml + TGFb3 10ng/ml +bglycerophosphate 10mM with 1nM Dexamethasone,,

iPSC,BIOTIME,GPL10588,MBA_6200,MEL2 P19 D14 Osteo monolayer Incomplete + BMP2 50ng/ml +bglycerophosphate 10mM with 1nM Dexamethasone,,

iPSC,BIOTIME,GPL10588,MBA_6201,SM30 P20 D21 collagen sp + BMP2 50ng/ml + TGFb3 10ng/ml + bGP 10mM 500000 cells,,

iPSC,BIOTIME,GPL10588,MBA_6202,MEL2 P19 D21 Collagen sp + BMP2 50ng/ml + TGFb3 10ng/ml + bGP 10mM 500000 cells,,

iPSC,BIOTIME,GPL10588,MBA_6203,T42 P21 D14 Hystem TGFb3 10ng/ml,,

iPSC,BIOTIME,GPL10588,MBA_6204,T42 P21 D14 Hystem TGFb3 10ng/ml,,

iPSC,BIOTIME,GPL10588,MBA_6205,T42 P21 D14 Hystem BMP2 50ng/ml,,

iPSC,BIOTIME,GPL10588,MBA_6206,T42 P21 D14 Hystem BMP2 50ng/ml,,

iPSC,BIOTIME,GPL10588,MBA_6207,T42 P21 D14 Hystem BMP2 50ng/ml + TGFb3 10ng/ml,,

iPSC,BIOTIME,GPL10588,MBA_6208,T42 P21 D14 Hystem BMP2 50ng/ml + TGFb3 10ng/ml,,

iPSC,BIOTIME,GPL10588,MBA_6209,T42 P21 D14 Hystem BMP4 10ng/ml,,

iPSC,BIOTIME,GPL10588,MBA_6210,T42 P21 D14 Hystem BMP4 10ng/ml,,

iPSC,BIOTIME,GPL10588,MBA_6211,T42 P21 D14 Hystem BMP4 10ng/ml + TGFb3 10ng/ml,,

iPSC,BIOTIME,GPL10588,MBA_6212,T42 P21 D14 Hystem BMP4 10ng/ml + TGFb3 10ng/ml,,

iPSC,BIOTIME,GPL10588,MBA_6213,T42 P21 D14 Hystem BMP7 100ng/ml,,

iPSC,BIOTIME,GPL10588,MBA_6214,T42 P21 D14 Hystem BMP7 100ng/ml,,

iPSC,BIOTIME,GPL10588,MBA_6215,T42 P21 D14 Hystem BMP7 100ng/ml + TGFb3 10ng/ml,,

iPSC,BIOTIME,GPL10588,MBA_6216,T42 P21 D14 Hystem BMP7 100ng/ml + TGFb3 10ng/ml,,

iPSC,BIOTIME,GPL10588,MBA_6217,T42 P20 D14 MM TGFb3 10ng/ml,,

iPSC,BIOTIME,GPL10588,MBA_6218,T42 P20 D14 MM TGFb3 10ng/ml,,

iPSC,BIOTIME,GPL10588,MBA_6220,W10 P15 D14 neuroscreen 1,,

iPSC,BIOTIME,GPL10588,MBA_6221,W10 P15 D14 neuroscreen 1,,

iPSC,BIOTIME,GPL10588,MBA_6222,W10 P15 D14 neuroscreen 2,,

iPSC,BIOTIME,GPL10588,MBA_6223,W10 P15 D14 neuroscreen 2,,

iPSC,BIOTIME,GPL10588,MBA_6224,W10 P15 D14 neuroscreen 3,,

iPSC,BIOTIME,GPL10588,MBA_6225,W10 P15 D14 neuroscreen 3,,

iPSC,BIOTIME,GPL10588,MBA_6226,W10 P15 D14 neuroscreen 4,,

iPSC,BIOTIME,GPL10588,MBA_6227,W10 P15 D14 neuroscreen 4,,

iPSC,BIOTIME,GPL10588,MBA_6228,W10 P15 D14 neuroscreen 5,,

iPSC,BIOTIME,GPL10588,MBA_6229,W10 P15 D14 neuroscreen 5,,

iPSC,BIOTIME,GPL10588,MBA_6231,Z11 P16 D14 neuroscreen 1,,

iPSC,BIOTIME,GPL10588,MBA_6232,Z11 P16 D14 neuroscreen 1,,

iPSC,BIOTIME,GPL10588,MBA_6233,Z11 P16 D14 neuroscreen 2,,

iPSC,BIOTIME,GPL10588,MBA_6234,Z11 P16 D14 neuroscreen 2,,

iPSC,BIOTIME,GPL10588,MBA_6235,Z11 P16 D14 neuroscreen 3,,

iPSC,BIOTIME,GPL10588,MBA_6236,Z11 P16 D14 neuroscreen 3,,

iPSC,BIOTIME,GPL10588,MBA_6237,Z11 P16 D14 neuroscreen 4,,

iPSC,BIOTIME,GPL10588,MBA_6238,Z11 P16 D14 neuroscreen 4,,

iPSC,BIOTIME,GPL10588,MBA_6239,Z11 P16 D14 neuroscreen 5,,

iPSC,BIOTIME,GPL10588,MBA_6240,Z11 P16 D14 neuroscreen 5,,

iPSC,BIOTIME,GPL10588,MBA_6243,U31 P15 D7 neuroscreen 1,,

iPSC,BIOTIME,GPL10588,MBA_6244,U31 P15 D7 neuroscreen 1,,

iPSC,BIOTIME,GPL10588,MBA_6245,U31 P15 D7 neuroscreen 2,,

iPSC,BIOTIME,GPL10588,MBA_6246,U31 P15 D7 neuroscreen 2,,

iPSC,BIOTIME,GPL10588,MBA_6247,U31 P15 D7 neuroscreen 3,,

iPSC,BIOTIME,GPL10588,MBA_6248,U31 P15 D7 neuroscreen 3,,

iPSC,BIOTIME,GPL10588,MBA_6249,U31 P15 D7 neuroscreen 4,,

iPSC,BIOTIME,GPL10588,MBA_6250,U31 P15 D7 neuroscreen 4,,

iPSC,BIOTIME,GPL10588,MBA_6251,U31 P15 D7 neuroscreen 5,,

iPSC,BIOTIME,GPL10588,MBA_6252,U31 P15 D7 neuroscreen 5,,

iPSC,BIOTIME,GPL10588,MBA_6255,T42 P19 D7 neuroscreen 1,,

iPSC,BIOTIME,GPL10588,MBA_6256,T42 P19 D7 neuroscreen 1,,

iPSC,BIOTIME,GPL10588,MBA_6257,T42 P19 D7 neuroscreen 2,,

iPSC,BIOTIME,GPL10588,MBA_6258,T42 P19 D7 neuroscreen 2,,

iPSC,BIOTIME,GPL10588,MBA_6259,T42 P19 D7 neuroscreen 3,,

iPSC,BIOTIME,GPL10588,MBA_6260,T42 P19 D7 neuroscreen 3,,

iPSC,BIOTIME,GPL10588,MBA_6261,T42 P19 D7 neuroscreen 4,,

iPSC,BIOTIME,GPL10588,MBA_6262,T42 P19 D7 neuroscreen 4,,

iPSC,BIOTIME,GPL10588,MBA_6263,T42 P19 D7 neuroscreen 5,,

iPSC,BIOTIME,GPL10588,MBA_6264,T42 P19 D7 neuroscreen 5,,

iPSC,BIOTIME,GPL10588,MBA_6265,Z11 P17 D14 Hystem TGFb3 10ng/ml,,

iPSC,BIOTIME,GPL10588,MBA_6266,Z11 P17 D14 Hystem TGFb3 10ng/ml,,

iPSC,BIOTIME,GPL10588,MBA_6267,Z11 P17 D14 Hystem BMP2 50ng/ml,,

iPSC,BIOTIME,GPL10588,MBA_6268,Z11 P17 D14 Hystem BMP2 50ng/ml,,

iPSC,BIOTIME,GPL10588,MBA_6269,Z11 P17 D14 Hystem BMP2 50ng/ml + TGFb3 10ng/ml,,

iPSC,BIOTIME,GPL10588,MBA_6270,Z11 P17 D14 Hystem BMP2 50ng/ml + TGFb3 10ng/ml,,

iPSC,BIOTIME,GPL10588,MBA_6271,Z11 P17 D14 Hystem BMP4 10ng/ml,,

iPSC,BIOTIME,GPL10588,MBA_6272,Z11 P17 D14 Hystem BMP4 10ng/ml,,

iPSC,BIOTIME,GPL10588,MBA_6273,Z11 P17 D14 Hystem BMP4 10ng/ml + TGFb3 10ng/ml,,

iPSC,BIOTIME,GPL10588,MBA_6274,Z11 P17 D14 Hystem BMP4 10ng/ml + TGFb3 10ng/ml,,

iPSC,BIOTIME,GPL10588,MBA_6275,Z11 P17 D14 Hystem BMP7 100ng/ml,,

iPSC,BIOTIME,GPL10588,MBA_6276,Z11 P17 D14 Hystem BMP7 100ng/ml,,

iPSC,BIOTIME,GPL10588,MBA_6277,Z11 P17 D14 Hystem BMP7 100ng/ml + TGFb3 10ng/ml,,

iPSC,BIOTIME,GPL10588,MBA_6278,Z11 P17 D14 Hystem BMP7 100ng/ml + TGFb3 10ng/ml,,

iPSC,BIOTIME,GPL10588,MBA_6279,Z11 P17 D14 MM TGFb3 10ng/ml,,

iPSC,BIOTIME,GPL10588,MBA_6280,Z11 P17 D14 MM TGFb3 10ng/ml,,

iPSC,BIOTIME,GPL10588,MBA_6283,Z11 P19 D7 neuroscreen 1,,

iPSC,BIOTIME,GPL10588,MBA_6284,Z11 P19 D7 neuroscreen 1,,

iPSC,BIOTIME,GPL10588,MBA_6285,Z11 P19 D7 neuroscreen 2,,

iPSC,BIOTIME,GPL10588,MBA_6286,Z11 P19 D7 neuroscreen 2,,

iPSC,BIOTIME,GPL10588,MBA_6287,Z11 P19 D7 neuroscreen 3,,

iPSC,BIOTIME,GPL10588,MBA_6288,Z11 P19 D7 neuroscreen 3,,

iPSC,BIOTIME,GPL10588,MBA_6289,Z11 P19 D7 neuroscreen 4,,

iPSC,BIOTIME,GPL10588,MBA_6290,Z11 P19 D7 neuroscreen 4,,

iPSC,BIOTIME,GPL10588,MBA_6291,Z11 P19 D7 neuroscreen 5,,

iPSC,BIOTIME,GPL10588,MBA_6292,Z11 P19 D7 neuroscreen 5,,

iPSC,BIOTIME,GPL10588,MBA_6293,W10 P16 D14 Hystem BMP2 50ng/ml,,

iPSC,BIOTIME,GPL10588,MBA_6294,W10 P16 D14 Hystem BMP2 50ng/ml,,

iPSC,BIOTIME,GPL10588,MBA_6295,W10 P16 D14 Hystem BMP2 50ng/ml + TGFb3 10ng/ml,,

iPSC,BIOTIME,GPL10588,MBA_6296,W10 P16 D14 Hystem BMP2 50ng/ml + TGFb3 10ng/ml,,

iPSC,BIOTIME,GPL10588,MBA_6297,W10 P16 D14 Hystem BMP4 10ng/ml,,

iPSC,BIOTIME,GPL10588,MBA_6298,W10 P16 D14 Hystem BMP4 10ng/ml,,

iPSC,BIOTIME,GPL10588,MBA_6299,W10 P16 D14 Hystem BMP4 10ng/ml + TGFb3 10ng/ml,,

iPSC,BIOTIME,GPL10588,MBA_6300,W10 P16 D14 Hystem BMP4 10ng/ml + TGFb3 10ng/ml,,

iPSC,BIOTIME,GPL10588,MBA_6301,W10 P16 D14 Hystem BMP7 100ng/ml,,

iPSC,BIOTIME,GPL10588,MBA_6302,W10 P16 D14 Hystem BMP7 100ng/ml,,

iPSC,BIOTIME,GPL10588,MBA_6303,W10 P16 D14 Hystem BMP7 100ng/ml + TGFb3 10ng/ml,,

iPSC,BIOTIME,GPL10588,MBA_6304,W10 P16 D14 Hystem BMP7 100ng/ml + TGFb3 10ng/ml,,

iPSC,BIOTIME,GPL10588,MBA_6305,W10 P17 D14 Hystem TGFb3 10ng/ml,,

iPSC,BIOTIME,GPL10588,MBA_6306,W10 P17 D14 Hystem TGFb3 10ng/ml,,

iPSC,BIOTIME,GPL10588,MBA_6307,W10 P16 D14 MM TGFb3 10ng/ml,,

iPSC,BIOTIME,GPL10588,MBA_6308,W10 P16 D14 MM TGFb3 10ng/ml,,

AC,BIOTIME,GPL10588,MBA_6309,CASMC P12 D14 Hystem TGFb3 10ng/ml,,

AC,BIOTIME,GPL10588,MBA_6310,CASMC P12 D14 Hystem TGFb3 10ng/ml,,

AC,BIOTIME,GPL10588,MBA_6311,CASMC P12 D14 Hystem BMP2 50ng/ml ,,

AC,BIOTIME,GPL10588,MBA_6312,CASMC P12 D14 Hystem BMP2 50ng/ml ,,

AC,BIOTIME,GPL10588,MBA_6313,CASMC P12 D14 Hystem BMP2 50ng/ml + TGFb3 10ng/ml,,

AC,BIOTIME,GPL10588,MBA_6314,CASMC P12 D14 Hystem BMP2 50ng/ml + TGFb3 10ng/ml,,

AC,BIOTIME,GPL10588,MBA_6315,CASMC P12 D14 Hystem BMP4 10ng/ml ,,

AC,BIOTIME,GPL10588,MBA_6316,CASMC P12 D14 Hystem BMP4 10ng/ml ,,

AC,BIOTIME,GPL10588,MBA_6317,CASMC P12 D14 Hystem BMP4 10ng/ml + TGFb3 10ng/ml,,

AC,BIOTIME,GPL10588,MBA_6318,CASMC P12 D14 Hystem BMP4 10ng/ml + TGFb3 10ng/ml,,

AC,BIOTIME,GPL10588,MBA_6319,CASMC P12 D14 Hystem BMP7 100ng/ml ,,

AC,BIOTIME,GPL10588,MBA_6320,CASMC P12 D14 Hystem BMP7 100ng/ml ,,

AC,BIOTIME,GPL10588,MBA_6321,CASMC P12 D14 Hystem BMP7 100ng/ml + TGFb3 10ng/ml,,

AC,BIOTIME,GPL10588,MBA_6322,CASMC P12 D14 Hystem BMP7 100ng/ml + TGFb3 10ng/ml,,

AC,BIOTIME,GPL10588,MBA_6323,CASMC P12 D14 MM TGFb3 10ng/ml ,,

AC,BIOTIME,GPL10588,MBA_6324,CASMC P12 D14 MM TGFb3 10ng/ml ,,

AC,BIOTIME,GPL10588,MBA_6327,CASMC P9 D14 neuroscreen 1,,

AC,BIOTIME,GPL10588,MBA_6328,CASMC P9 D14 neuroscreen 1,,

AC,BIOTIME,GPL10588,MBA_6329,CASMC P9 D14 neuroscreen 2,,

AC,BIOTIME,GPL10588,MBA_6330,CASMC P9 D14 neuroscreen 2,,

AC,BIOTIME,GPL10588,MBA_6331,CASMC P9 D14 neuroscreen 3,,

AC,BIOTIME,GPL10588,MBA_6332,CASMC P9 D14 neuroscreen 3,,

AC,BIOTIME,GPL10588,MBA_6333,CASMC P9 D14 neuroscreen 4,,

AC,BIOTIME,GPL10588,MBA_6334,CASMC P9 D14 neuroscreen 4,,

AC,BIOTIME,GPL10588,MBA_6335,CASMC P9 D14 neuroscreen 5,,

AC,BIOTIME,GPL10588,MBA_6336,CASMC P9 D14 neuroscreen 5,,

AC,BIOTIME,GPL10588,MBA_6337,CASMC P10 D14 neuroscreen 1,,

AC,BIOTIME,GPL10588,MBA_6338,CASMC P10 D14 neuroscreen 2,,

AC,BIOTIME,GPL10588,MBA_6339,CASMC P10 D14 neuroscreen 3,,

iPSC,BIOTIME,GPL10588,MBA_6340,U31 P17 D14 Hystem TGFb3 10ng/ml,,

iPSC,BIOTIME,GPL10588,MBA_6341,U31 P17 D14 Hystem TGFb3 10ng/ml,,

iPSC,BIOTIME,GPL10588,MBA_6342,U31 P17 D14 Hystem BMP2 50ng/ml ,,

iPSC,BIOTIME,GPL10588,MBA_6343,U31 P17 D14 Hystem BMP2 50ng/ml ,,

iPSC,BIOTIME,GPL10588,MBA_6344,U31 P17 D14 Hystem BMP2 50ng/ml + TGFb3 10ng/ml,,

iPSC,BIOTIME,GPL10588,MBA_6345,U31 P17 D14 Hystem BMP2 50ng/ml + TGFb3 10ng/ml,,

iPSC,BIOTIME,GPL10588,MBA_6346,U31 P17 D14 Hystem BMP4 10ng/ml ,,

iPSC,BIOTIME,GPL10588,MBA_6347,U31 P17 D14 Hystem BMP4 10ng/ml ,,

iPSC,BIOTIME,GPL10588,MBA_6348,U31 P17 D14 Hystem BMP4 10ng/ml + TGFb3 10ng/ml,,

iPSC,BIOTIME,GPL10588,MBA_6349,U31 P17 D14 Hystem BMP4 10ng/ml + TGFb3 10ng/ml,,

iPSC,BIOTIME,GPL10588,MBA_6350,U31 P17 D14 Hystem BMP7 100ng/ml ,,

iPSC,BIOTIME,GPL10588,MBA_6351,U31 P17 D14 Hystem BMP7 100ng/ml ,,

iPSC,BIOTIME,GPL10588,MBA_6352,U31 P17 D14 Hystem BMP7 100ng/ml + TGFb3 10ng/ml,,

iPSC,BIOTIME,GPL10588,MBA_6353,U31 P17 D14 Hystem BMP7 100ng/ml + TGFb3 10ng/ml,,

iPSC,BIOTIME,GPL10588,MBA_6354,U31 P17 D14 MM TGFb3 10ng/ml,,

iPSC,BIOTIME,GPL10588,MBA_6355,U31 P17 D14 MM TGFb3 10ng/ml,,

AC,BIOTIME,GPL10588,MBA_6356,MSC Caplan 1913 D0 Pellet culture (scaled with FGF2),,

AC,BIOTIME,GPL10588,MBA_6357,MSC Caplan 1913 D3 Pellet culture (scaled with FGF2),,

AC,BIOTIME,GPL10588,MBA_6358,MSC Caplan 1913 D7 Pellet culture (scaled with FGF2),,

AC,BIOTIME,GPL10588,MBA_6359,MSC Caplan 1913 D10 Pellet culture (scaled with FGF2),,

AC,BIOTIME,GPL10588,MBA_6360,MSC Caplan 1913 D14 Pellet culture (scaled with FGF2),,

AC,BIOTIME,GPL10588,MBA_6361,MSC Caplan 1913 D21 Pellet culture (scaled with FGF2),,

AC,BIOTIME,GPL10588,MBA_6362,MSC Caplan 1913 D28 Pellet culture (scaled with FGF2),,

AC,BIOTIME,GPL10588,MBA_6363,MSC Caplan 1926 D0 Pellet culture (scaled with FGF2),,

AC,BIOTIME,GPL10588,MBA_6364,MSC Caplan 1926 D3 Pellet culture (scaled with FGF2),,

AC,BIOTIME,GPL10588,MBA_6365,MSC Caplan 1926 D7 Pellet culture (scaled with FGF2),,

AC,BIOTIME,GPL10588,MBA_6366,MSC Caplan 1926 D10 Pellet culture (scaled with FGF2),,

AC,BIOTIME,GPL10588,MBA_6367,MSC Caplan 1926 D14 Pellet culture (scaled with FGF2),,

AC,BIOTIME,GPL10588,MBA_6368,MSC Caplan 1926 D21 Pellet culture (scaled with FGF2),,

AC,BIOTIME,GPL10588,MBA_6369,MSC Caplan 1926 D28 Pellet culture (scaled with FGF2),,

iPSC,BIOTIME,GPL10588,MBA_6370,NOISY microarray signal SM30 P24 ctrl from Quantum cell expansion,,

iPSC,BIOTIME,GPL10588,MBA_6371,SM30 P24 D14 MM BMP4 10ng/ml + TGFb3 10ng/ml Expanded in Quantum prior,,

iPSC,BIOTIME,GPL10588,MBA_6372,ESI #003 cl.29 P5 ctrl DM 20% FBS Derived from ESI017 on irradiated feeders same as 4D20.6 (9/6/13),,

iPSC,BIOTIME,GPL10588,MBA_6373,ESI #003 cl.29 P5 D14 MM BMP4 10ng/ml + TGFb3 10ng/ml,,

iPSC,BIOTIME,GPL10588,MBA_6374,ESI #003 cl.30 P5 ctrl DM 20% FBS (9/6/13) Derived from ESI017 on irradiated feeders same as 4D20.6 ,,

iPSC,BIOTIME,GPL10588,MBA_6375,ESI #003 cl.30 P5 D14 MM BMP4 10ng/ml + TGFb3 10ng/ml,,

iPSC,BIOTIME,GPL10588,MBA_6398,"Passed OPC1 Lot 1, 10 uL of RNA, 522 ng/uL From Asterias Nate Manley",,

iPSC,BIOTIME,GPL10588,MBA_6399,"Failed OPC1 Lot1, 10 uL of RNA, 312 ng/uL",,

iPSC,BIOTIME,GPL10588,MBA_6497,C4ELS5.6 P15 D14 Hystem adiposcreen 1,,

iPSC,BIOTIME,GPL10588,MBA_6498,C4ELS5.6 P15 D14 Hystem adiposcreen 2,,

iPSC,BIOTIME,GPL10588,MBA_6499,C4ELS5.6 P15 D10 Hystem adiposcreen 3,,

iPSC,BIOTIME,GPL10588,MBA_6500,C4ELS5.6 P15 D10 Hystem adiposcreen 4,,

iPSC,BIOTIME,GPL10588,MBA_6501,C4ELS5.6 P15 D10 Hystem adiposcreen 5,,

iPSC,BIOTIME,GPL10588,MBA_6502,C4ELS5.6 P15 D10 Hystem adiposcreen 6,,

iPSC,BIOTIME,GPL10588,MBA_6503,C4ELS5.6 P15 D10 Hystem adiposcreen 7,,

iPSC,BIOTIME,GPL10588,MBA_6504,C4ELS5.6 P15 D10 Hystem adiposcreen 8,,

iPSC,BIOTIME,GPL10588,MBA_6506,W11 P13 D14 Hystem adiposcreen 1,,

iPSC,BIOTIME,GPL10588,MBA_6507,W11 P13 D10 Hystem adiposcreen 4,,

iPSC,BIOTIME,GPL10588,MBA_6508,W11 P13 D10 Hystem adiposcreen 5,,

iPSC,BIOTIME,GPL10588,MBA_6509,W11 P13 D10 Hystem adiposcreen 6,,

iPSC,BIOTIME,GPL10588,MBA_6510,W11 P13 D10 Hystem adiposcreen 7,,

iPSC,BIOTIME,GPL10588,MBA_6511,W11 P13 D10 Hystem adiposcreen 8,,

iPSC,BIOTIME,GPL10588,MBA_6513,W10 P13 D10 Hystem adiposcreen 3,,

iPSC,BIOTIME,GPL10588,MBA_6514,W10 P13 D10 Hystem adiposcreen 4,,

iPSC,BIOTIME,GPL10588,MBA_6515,W10 P13 D10 Hystem adiposcreen 5,,

iPSC,BIOTIME,GPL10588,MBA_6516,W10 P13 D10 Hystem adiposcreen 6,,

iPSC,BIOTIME,GPL10588,MBA_6517,W10 P13 D10 Hystem adiposcreen 7,,

iPSC,BIOTIME,GPL10588,MBA_6518,W10 P13 D10 Hystem adiposcreen 8,,

iPSC,BIOTIME,GPL10588,MBA_6520,C4ELSR12 P11 D14 Hystem march adipo 7,,

iPSC,BIOTIME,GPL10588,MBA_6522,RAD20.16 P16 D14 Hystem march adipo 1,,

iPSC,BIOTIME,GPL10588,MBA_6523,RAD20.16 P16 D14 Hystem march adipo 2,,

iPSC,BIOTIME,GPL10588,MBA_6524,RAD20.16 P16 D14 Hystem march adipo 3,,

iPSC,BIOTIME,GPL10588,MBA_6525,RAD20.16 P16 D14 Hystem march adipo 4,,

iPSC,BIOTIME,GPL10588,MBA_6526,RAD20.16 P16 D14 Hystem march adipo 5,,

iPSC,BIOTIME,GPL10588,MBA_6527,RAD20.16 P16 D14 Hystem march adipo 6,,

iPSC,BIOTIME,GPL10588,MBA_6529,RAD20.24 P14 D14 Hystem march adipo new 4,,

iPSC,BIOTIME,GPL10588,MBA_6530,RAD20.24 P14 D14 Hystem march adipo new 5,,

iPSC,BIOTIME,GPL10588,MBA_6531,RAD20.24 P14 D14 Hystem march adipo new 6,,

iPSC,BIOTIME,GPL10588,MBA_6532,RAD20.24 P14 D14 Hystem march adipo new 7,,

iPSC,BIOTIME,GPL10588,MBA_6534,E72 P14 D14 Hystem march adipo 2,,

iPSC,BIOTIME,GPL10588,MBA_6535,E72 P14 D14 Hystem march adipo 5,,

iPSC,BIOTIME,GPL10588,MBA_6536,E72 P14 D14 Hystem march adipo 7,,

iPSC,BIOTIME,GPL10588,MBA_6538,J16 P13 D14 Hystem march adipo 4,,

iPSC,BIOTIME,GPL10588,MBA_6539,J16 P13 D14 Hystem march adipo 6,,

iPSC,BIOTIME,GPL10588,MBA_6540,J16 P13 D14 Hystem march adipo 7,,

iPSC,BIOTIME,GPL10588,MBA_6542,E111 P18 D14 Hystem march adipo 1,,

iPSC,BIOTIME,GPL10588,MBA_6543,E111 P18 D14 Hystem march adipo 2,,

iPSC,BIOTIME,GPL10588,MBA_6544,E111 P18 D14 Hystem march adipo 4,,

iPSC,BIOTIME,GPL10588,MBA_6545,E111 P18 D14 Hystem march adipo 6,,

iPSC,BIOTIME,GPL10588,MBA_6546,E111 P18 D14 Hystem march adipo 7,,

iPSC,BIOTIME,GPL10588,MBA_6548,CM02 P18 D14 Hystem march adipo 1,,

iPSC,BIOTIME,GPL10588,MBA_6549,CM02 P18 D14 Hystem march adipo 2,,

iPSC,BIOTIME,GPL10588,MBA_6550,CM02 P18 D14 Hystem march adipo 4,,

iPSC,BIOTIME,GPL10588,MBA_6551,CM02 P18 D14 Hystem march adipo 6,,

iPSC,BIOTIME,GPL10588,MBA_6552,CM02 P18 D14 Hystem march adipo 7,,

iPSC,BIOTIME,GPL10588,MBA_6553,SM30 P24 D14 MM BMP4 10ng/ml + TGFb3 10ng/ml,,

iPSC,BIOTIME,GPL10588,MBA_6554,SM30 P24 D14 MM BMP4 10ng/ml + TGFb3 10ng/ml,,

iPSC,BIOTIME,GPL10588,MBA_6555,SM30 P24 D21 MM BMP4 10ng/ml + TGFb3 10ng/ml T225,,

iPSC,BIOTIME,GPL10588,MBA_6556,SM30 P24 D21 MM BMP4 10ng/ml + TGFb3 10ng/ml Quantum,,

iPSC,BIOTIME,GPL10588,MBA_6557,SK11 P17 ctrl,,

iPSC,BIOTIME,GPL10588,MBA_6558,SK11 P17 D21 Hystem incomplete +Dex 100nm+ betaglycerophosphate 10mM + BMP2 50ng/ml,,

iPSC,BIOTIME,GPL10588,MBA_6559,SK11 P17 D21 Hystem incomplete +Dex 100nm+ betaglycerophosphate 10mM + BMP2 50ng/ml + TGFb3 10ng/ml,,

iPSC,BIOTIME,GPL10588,MBA_6560,SK11 P17 D21 Hystem incomplete no Dex + betaglycerophosphate 10mM + BMP2 50ng/ml ,,

iPSC,BIOTIME,GPL10588,MBA_6561,SK11 P17 D21 Hystem incomplete no Dex + betaglycerophosphate 10mM + BMP2 50ng/ml + TGFb3 10ng/ml,,

iPSC,BIOTIME,GPL10588,MBA_6562,MEL2 P18 D21 monolayer Osteo in incomplete chondro medium (100nM Dex) with betaglycerophosphate 10mM and BMP2 50ng/ml + TGFb3 10ng/ml,,

iPSC,BIOTIME,GPL10588,MBA_6563,"MEL2 P18 D21 monolayer Osteo in incomplete chondro medium with 1nM Dex, betaglycerophosphate 10mM, and BMP2 50ng/ml + TGFb3 10ng/ml",,

iPSC,BIOTIME,GPL10588,MBA_6564,"MEL2 P18 D21 monolayer Osteo in DMEM low glucose, 0.17mM ascorbate, 10 nM Dex, 10% FBS, betaglycerophosphate 10mM, BMP2 50ng/ml ",,

AC,BIOTIME,GPL10588,MBA_6565,"MSC P7 D21 monolayer Osteo in DMEM low glucose, 0.17mM ascorbate, 10 nM Dex, 10% FBS, betaglycerophosphate 10mM, BMP2 50ng/ml ",,

AC,BIOTIME,GPL10588,MBA_6566,CASMC P2 ctrl,,

iPSC,BIOTIME,GPL10588,MBA_6569,C4ELS5.1 P14 D14 Hystem march adipo 2,,

iPSC,BIOTIME,GPL10588,MBA_6570,C4ELS5.1 P14 D14 Hystem march adipo 4,,

iPSC,BIOTIME,GPL10588,MBA_6571,C4ELS5.1 P14 D14 Hystem march adipo 6,,

iPSC,BIOTIME,GPL10588,MBA_6572,C4ELS5.1 P14 D14 Hystem march adipo 7,,

iPSC,BIOTIME,GPL10588,MBA_6574,E85 P18 D14 Hystem march adipo 1,,

iPSC,BIOTIME,GPL10588,MBA_6575,E85 P18 D15 Hystem march adipo 2,,

iPSC,BIOTIME,GPL10588,MBA_6576,E85 P18 D14 Hystem march adipo 4,,

iPSC,BIOTIME,GPL10588,MBA_6577,E85 P18 D14 Hystem march adipo 6,,

iPSC,BIOTIME,GPL10588,MBA_6578,E85 P18 D14 Hystem march adipo 7,,

AC,BIOTIME,GPL10588,MBA_6579,Xgene P16 siRNA non-target pool ctrl day 2,,

AC,BIOTIME,GPL10588,MBA_6580,Xgene P16 siRNA COX7A1 Knock down pool day 2,,

AC,BIOTIME,GPL10588,MBA_6581,Xgene P18 expt 3 (6hr 2x) siRNA non-target pool D2,,

AC,BIOTIME,GPL10588,MBA_6582,Xgene P18 expt 3 (6hr 2x) siRNA COX7A1 pool D2,,

AC,BIOTIME,GPL10588,MBA_6583,Xgene P18 expt 3 (6hr 2x) siRNA non-target pool D4,,

AC,BIOTIME,GPL10588,MBA_6584,Xgene P18 expt 3 (6hr 2x) siRNA COX7A1 pool D4,,

iPSC,BIOTIME,GPL10588,MBA_6586,E68 P21 D14 Hystem march adipo 1,,

iPSC,BIOTIME,GPL10588,MBA_6587,E68 P21 D14 Hystem march adipo 2,,

iPSC,BIOTIME,GPL10588,MBA_6588,E68 P21 D14 Hystem march adipo 4,,

iPSC,BIOTIME,GPL10588,MBA_6589,E68 P21 D14 Hystem march adipo 6,,

iPSC,BIOTIME,GPL10588,MBA_6590,E68 P21 D14 Hystem march adipo 7,,

iPSC,BIOTIME,GPL10588,MBA_6592,E120 P15 D14 Hystem march adipo 1,,

iPSC,BIOTIME,GPL10588,MBA_6593,E120 P15 D15 Hystem march adipo 1,,

iPSC,BIOTIME,GPL10588,MBA_6594,E120 P15 D14 Hystem march adipo 4,,

iPSC,BIOTIME,GPL10588,MBA_6595,E120 P15 D14 Hystem march adipo 6,,

iPSC,BIOTIME,GPL10588,MBA_6596,E120 P15 D14 Hystem march adipo 7,,

iPSC,BIOTIME,GPL10588,MBA_6598,RAD20.6 P12 D14 Hystem march adipo 1,,

iPSC,BIOTIME,GPL10588,MBA_6599,RAD20.6 P12 D14 Hystem march adipo 2,,

iPSC,BIOTIME,GPL10588,MBA_6600,RAD20.6 P12 D14 Hystem march adipo 4,,

iPSC,BIOTIME,GPL10588,MBA_6601,RAD20.6 P12 D14 Hystem march adipo 6,,

iPSC,BIOTIME,GPL10588,MBA_6602,RAD20.6 P12 D14 Hystem march adipo 7,,

iPSC,BIOTIME,GPL10588,MBA_6604,E44 P20 D14 Hystem march adipo 1,,

iPSC,BIOTIME,GPL10588,MBA_6605,E44 P20 D14 Hystem march adipo 2,,

iPSC,BIOTIME,GPL10588,MBA_6606,E44 P20 D14 Hystem march adipo 4,,

iPSC,BIOTIME,GPL10588,MBA_6607,E44 P20 D14 Hystem march adipo 6,,

iPSC,BIOTIME,GPL10588,MBA_6608,E44 P20 D14 Hystem march adipo 7,,

iPSC,BIOTIME,GPL10588,MBA_6610,RAD20.5 P16 D14 Hystem march adipo 1,,

iPSC,BIOTIME,GPL10588,MBA_6611,RAD20.5 P16 D14 Hystem march adipo 2,,

iPSC,BIOTIME,GPL10588,MBA_6612,RAD20.5 P16 D14 Hystem march adipo 4,,

iPSC,BIOTIME,GPL10588,MBA_6613,RAD20.5 P16 D14 Hystem march adipo 6,,

iPSC,BIOTIME,GPL10588,MBA_6614,RAD20.5 P16 D14 Hystem march adipo 7,,

iPSC,BIOTIME,GPL10588,MBA_6616,E69 P15 D14 Hystem march adipo 1,,

iPSC,BIOTIME,GPL10588,MBA_6617,E69 P15 D14 Hystem march adipo 2,,

iPSC,BIOTIME,GPL10588,MBA_6618,E69 P15 D14 Hystem march adipo 4,,

iPSC,BIOTIME,GPL10588,MBA_6619,E69 P15 D14 Hystem march adipo 6,,

iPSC,BIOTIME,GPL10588,MBA_6620,E69 P15 D14 Hystem march adipo 7,,

iPSC,BIOTIME,GPL10588,MBA_6621,SM30 P20 D21 confluence osteo with 10% FBS,,

iPSC,BIOTIME,GPL10588,MBA_6622,MEL2 P20 D21 confluence osteo with 10% FBS,,

iPSC,BIOTIME,GPL10588,MBA_6720,C4ELS5.1 P12 D14 Chondro MM TGFb3 10ng/ml,,

AC,BIOTIME,GPL10588,MBA_6721,human fetal brown preadipocytes 20 wk Zenbio RNA-T10-CS (Lot 10914A),,

iPSC,BIOTIME,GPL10588,MBA_6723,E164 P22 D14 Hystem march adipo 1,,

iPSC,BIOTIME,GPL10588,MBA_6724,E164 P22 D14 Hystem march adipo 2,,

iPSC,BIOTIME,GPL10588,MBA_6725,E164 P22 D14 Hystem march adipo 4,,

iPSC,BIOTIME,GPL10588,MBA_6726,E164 P22 D14 Hystem march adipo 6,,

iPSC,BIOTIME,GPL10588,MBA_6727,E164 P22 D14 Hystem march adipo 7,,

iPSC,BIOTIME,GPL10588,MBA_6729,E33 P14 D14 Hystem march adipo 1,,

iPSC,BIOTIME,GPL10588,MBA_6730,E33 P14 D14 Hystem march adipo 2,,

iPSC,BIOTIME,GPL10588,MBA_6731,E33 P14 D14 Hystem march adipo 4,,

iPSC,BIOTIME,GPL10588,MBA_6732,E33 P14 D14 Hystem march adipo 6,,

iPSC,BIOTIME,GPL10588,MBA_6733,E33 P14 D14 Hystem march adipo 7,,

iPSC,BIOTIME,GPL10588,MBA_6735,EN7 P15 D7 Confluence Incomplete Chondro BMP7 200ng/ml + Rosiglitazone 1uM ,,

iPSC,BIOTIME,GPL10588,MBA_6736,EN7 P15 D7 Confluence Incomplete Chondro BMP7 200ng/ml + Rosiglitazone 1uM last day incubator set at 28C,,

iPSC,BIOTIME,GPL10588,MBA_6737,EN7 P15 D7 Confluence Incomplete Chondro BMP4 50ng/ml + Rosigliatazone 1uM ,,

iPSC,BIOTIME,GPL10588,MBA_6738,"EN7 P15 D7 Confluence Incomplete Chondro BMP4 50ng/ml + Rosiglitazone 1uM, last day CL316243 10uM for 4 hours",,

iPSC,BIOTIME,GPL10588,MBA_6739,EN7 P15 D14 Hystem Incomplete Chondro BMP4 50ng/ml + Rosigliatazone 5uM ,,

iPSC,BIOTIME,GPL10588,MBA_6740,"EN7 P15 D14 Hystem Incomplete Chondro BMP4 50ng/ml + Rosiglitazone 5uM, last day CL316243 10uM for 4 hours ",,

iPSC,BIOTIME,GPL10588,MBA_6741,EN7 P15 D14 Hystem Incomplete Chondro BMP7 200ng/ml + 5uM Rosiglitazone ,,

iPSC,BIOTIME,GPL10588,MBA_6743,EN8 P14 D7 Confluence Incomplete Chondro BMP7 200ng/ml + Rosiglitazone 1uM ,,

iPSC,BIOTIME,GPL10588,MBA_6744,EN8 P14 D7 Confluence Incomplete Chondro BMP7 200ng/ml + Rosiglitazone 1uM last day incubator set at 28C,,

iPSC,BIOTIME,GPL10588,MBA_6745,EN8 P14 D7 Confluence Incomplete Chondro BMP4 50ng/ml + Rosigliatazone 1uM 7 days ,,

iPSC,BIOTIME,GPL10588,MBA_6746,EN8 P14 D7 Confluence Incomplete Chondro BMP4 50ng/ml + Rosiglitazone 1uM last day add CL316243 10uM ,,

iPSC,BIOTIME,GPL10588,MBA_6747,EN8 P14 D14 Hystem Incomplete Chondro BMP4 50ng/ml + Rosigliatazone 5uM ,,

iPSC,BIOTIME,GPL10588,MBA_6748,"EN8 P14 D14 Hystem Incomplete Chondro BMP4 50ng/ml + Rosiglitazone 5uM, day 14 CL316243 10uM for 4 hours ",,

iPSC,BIOTIME,GPL10588,MBA_6749,EN8 P14 D14 Hystem Incomplete Chondro BMP7 200ng/ml + 5uM Rosiglitazone ,,

iPSC,BIOTIME,GPL10588,MBA_6751,EN42 P14 D7 Confluence Incomplete Chondro BMP7 200ng/ml + Rosiglitazone 1uM ,,

iPSC,BIOTIME,GPL10588,MBA_6752,"EN42 P14 D7 Confluence Incomplete Chondro BMP7 200ng/ml + Rosiglitazone 1uM, last day incubator set at 28C",,

iPSC,BIOTIME,GPL10588,MBA_6753,EN42 P14 D7 Confluence Incomplete Chondro BMP4 50ng/ml + Rosigliatazone 1uM ,,

iPSC,BIOTIME,GPL10588,MBA_6754,EN42 P14 D7 Confluence Incomplete Chondro BMP4 50ng/ml + Rosiglitazone 1uM day 6 CL316243 10nM,,

iPSC,BIOTIME,GPL10588,MBA_6755,EN42 P14 D14 Hystem Incomplete Chondro BMP4 50ng/ml + Rosigliatazone 5uM ,,

iPSC,BIOTIME,GPL10588,MBA_6756,"EN42 P14 D14 Hystem Incomplete Chondro BMP4 50ng/ml + Rosiglitazone 5uM, last day CL316243 10uM for 4 hours ",,

iPSC,BIOTIME,GPL10588,MBA_6757,EN42 P14 D14 Hystem Incomplete Chondro BMP7 200ng/ml + 5uM Rosiglitazone ,,

iPSC,BIOTIME,GPL10588,MBA_6759,C4ELSR14 P10 D14 Hystem BMP7 200ng/ml + 5uM Rosiglitazone ,,

iPSC,BIOTIME,GPL10588,MBA_6760,C4ELSR14 P10 D14 Hystem BMP4 50ng/ml + 5uM Rosiglitazone,,

iPSC,BIOTIME,GPL10588,MBA_6761,"C4ELSR14 P10 D14 Hystem BMP7 200ng/ml + 5uM Rosiglitazone, last day CL316243 10uM for 4 hours ",,

iPSC,BIOTIME,GPL10588,MBA_6762,"C4ELSR14 P10 D14 Hystem BMP4 50ng/ml + Rosiglitazone 5uM, last day CL316243 10uM for 4 hours",,

iPSC,BIOTIME,GPL10588,MBA_6763,C4ELSR14 P10 D14 Hystem BMP7 200ng/ml + Rosiglitazone 5uM last day incubator set at 28C,,

iPSC,BIOTIME,GPL10588,MBA_6764,C4ELSR14 P10 D14 Confluence Incomplete Chondro BMP7 200ng/ml + Rosiglitazone 5uM ,,

iPSC,BIOTIME,GPL10588,MBA_6765,C4ELSR14 P10 D14 Confluence Incomplete Chondro BMP4 50ng/ml + Rosiglitazone 5uM,,

iPSC,BIOTIME,GPL10588,MBA_6767,"7SMOO32 P13 D14 Hystem BMP7 200ng/ml + 5uM Rosiglitazone, last day add CL316243 10uM for 4 hours ",,

iPSC,BIOTIME,GPL10588,MBA_6768,"7SMOO32 P13 D14 Hystem BMP4 50ng/ml + Rosiglitazone 5uM, last day add CL316243 10uM for 4 hours",,

iPSC,BIOTIME,GPL10588,MBA_6769,7SMOO32 P13 D14 Hystem BMP7 200ng/ml + Rosiglitazone 5uM last day incubator set at 28C,,

iPSC,BIOTIME,GPL10588,MBA_6770,7SMOO32 P13 D14 confluence BMP7 200ng/ml + 5uM Rosiglitazone ,,

iPSC,BIOTIME,GPL10588,MBA_6771,7SMOO32 P13 D14 confluence BMP4 50ng/ml + 5uM Rosiglitazone ,,

iPSC,BIOTIME,GPL10588,MBA_6773,C4ELSR18 P10 D14 Hystem BMP4 50ng/ml + 5uM Rosiglitazone ,,

iPSC,BIOTIME,GPL10588,MBA_6774,"C4ELSR18 P10 D14 Hystem BMP7 200ng/ml + 5uM Rosiglitazone, last day CL316243 10uM for 4 hours ",,

iPSC,BIOTIME,GPL10588,MBA_6775,"C4ELSR18 P10 D14 Hystem BMP4 50ng/ml + Rosiglitazone 5uM, last day CL316243 10uM for 4 hours",,

iPSC,BIOTIME,GPL10588,MBA_6776,C4ELSR18 P10 D14 confluence Incomplete chondro BMP7 200ng/ml + Rosiglitazone 5uM ,,

iPSC,BIOTIME,GPL10588,MBA_6777,C4ELSR18 P10 D14 confluence Incomplete chondro BMP4 50ng/ml + 5uM Rosiglitazone ,,

iPSC,BIOTIME,GPL10588,MBA_6779,C4ELSR10 P10 D14 Hystem BMP7 200ng/ml + 5uM Rosiglitazone ,,

iPSC,BIOTIME,GPL10588,MBA_6780,C4ELSR10 P10 D14 Hystem BMP4 50ng/ml + 5uM Rosiglitazone,,

iPSC,BIOTIME,GPL10588,MBA_6781,"C4ELSR10 P10 D14 Hystem BMP7 200ng/ml + 5uM Rosiglitazone, last day CL316243 10uM for 4 hours ",,

iPSC,BIOTIME,GPL10588,MBA_6782,"C4ELSR10 P10 D14 Hystem BMP4 50ng/ml + Rosiglitazone 5uM, last day CL316243 10uM for 4 hours",,

iPSC,BIOTIME,GPL10588,MBA_6783,C4ELSR10 P10 D14 Hystem BMP7 200ng/ml + Rosiglitazone 5uM last day incubator set at 28C,,

iPSC,BIOTIME,GPL10588,MBA_6784,C4ELSR10 P10 D14 confluence Incomplete chondro BMP7 200ng/ml + Rosiglitazone 5uM ,,

iPSC,BIOTIME,GPL10588,MBA_6785,C4ELSR10 P10 D14 confluence Incomplete chondro BMP4 50ng/ml + 5uM Rosiglitazone,,

iPSC,BIOTIME,GPL10588,MBA_6787,C4ELS5.6 P10 D14 Hystem BMP7 200ng/ml + Rosiglitazone 5uM ,,

iPSC,BIOTIME,GPL10588,MBA_6788,C4ELS5.6 P10 D14 Hystem BMP4 50ng/ml + 5uM Rosiglitazone,,

iPSC,BIOTIME,GPL10588,MBA_6789,"C4ELS5.6 P10 D14 Hystem BMP7 200ng/ml + 5uM Rosiglitazone, last day CL316243 10uM for 4 hours ",,

iPSC,BIOTIME,GPL10588,MBA_6790,"C4ELS5.6 P10 D14 Hystem BMP4 50ng/ml + Rosiglitazone 5uM, last day CL316243 10uM for 4 hours",,

iPSC,BIOTIME,GPL10588,MBA_6791,C4ELS5.6 P10 D14 Hystem BMP7 200ng/ml + Rosiglitazone 5uM last day incubator set at 28C,,

iPSC,BIOTIME,GPL10588,MBA_6792,C4ELS5.6 P10 D14 confluence Incomplete chondro BMP7 200ng/ml + 5uM Rosiglitazone,,

iPSC,BIOTIME,GPL10588,MBA_6793,C4ELS5.6 P10 D14 confluence Incomplete chondro BMP4 50ng/ml + 5uM Rosiglitazone,,

AC,BIOTIME,GPL10588,MBA_6928,CASMC P5 Lonza new D14 Hystem BMP2 50ng/ml,,

AC,BIOTIME,GPL10588,MBA_6929,CASMC P5 Lonza new D14 Hystem BMP4 10ng/ml,,

AC,BIOTIME,GPL10588,MBA_6930,CASMC P5 Lonza new D14 Hystem BMP7 100ng/ml,,

AC,BIOTIME,GPL10588,MBA_6931,CASMC P5 Lonza new D14 Hystem TGFb3 10ng/ml,,

AC,BIOTIME,GPL10588,MBA_6932,CASMC P5 Lonza new D14 Hystem BMP4 10ng/ml + TGFb3 10ng/ml,,

AC,BIOTIME,GPL10588,MBA_6934,CASMC P7 Lifeline D14 Hystem BMP2 50ng/ml,,

AC,BIOTIME,GPL10588,MBA_6935,CASMC P7 Lifeline D14 Hystem BMP4 10ng/ml,,

AC,BIOTIME,GPL10588,MBA_6936,CASMC P7 Lifeline D14 Hystem BMP7 100ng/ml,,

AC,BIOTIME,GPL10588,MBA_6937,CASMC P7 Lifeline D14 Hystem TGFb3 10ng/ml,,

AC,BIOTIME,GPL10588,MBA_6938,CASMC P7 Lifeline D14 Hystem BMP4 10ng/ml + TGFb3 10ng/ml,,

AC,BIOTIME,GPL10588,MBA_6940,HCASMC P5 Cell applications D14 Hystem BMP2 50ng/ml,,

AC,BIOTIME,GPL10588,MBA_6941,HCASMC P5 Cell applications D14 Hystem BMP4 10ng/ml,,

AC,BIOTIME,GPL10588,MBA_6942,HCASMC P5 Cell applications D14 Hystem BMP7 100ng/ml,,

AC,BIOTIME,GPL10588,MBA_6943,HCASMC P5 Cell applications D14 Hystem TGFb3 10ng/ml,,

AC,BIOTIME,GPL10588,MBA_6944,HCASMC P5 Cell applications D14 Hystem BMP4 10ng/ml + TGFb3 10ng/ml,,

iPSC,BIOTIME,GPL10588,MBA_6946,RAPEND15 P15 D14 Hystem BMP7 200ng/ml + Rosiglitazone 5uM ,,

iPSC,BIOTIME,GPL10588,MBA_6947,RAPEND15 P15 D14 Hystem BMP4 50ng/ml + 5uM Rosiglitazone,,

iPSC,BIOTIME,GPL10588,MBA_6948,"RAPEND15 P15 D14 Hystem BMP7 200ng/ml + 5uM Rosiglitazone, last day CL316243 10uM for 4 hours ",,

iPSC,BIOTIME,GPL10588,MBA_6949,"RAPEND15 P15 D14 Hystem BMP4 50ng/ml + Rosiglitazone 5uM, last day CL316243 10uM for 4 hours",,

iPSC,BIOTIME,GPL10588,MBA_6950,"RAPEND15 P15 D14 Hystem BMP7 200ng/ml + Rosiglitazone 5uM, last day incubator set at 28C",,

iPSC,BIOTIME,GPL10588,MBA_6951,RAPEND15 P15 D14 confluence BMP7 200ng/ml + Rosiglitazone 5uM ,,

iPSC,BIOTIME,GPL10588,MBA_6952,RAPEND15 P15 D14 confluence BMP4 50ng/ml + Rosiglitazone 5uM ,,

iPSC,BIOTIME,GPL10588,MBA_6954,"C4ELSR2 P10 Hystem BMP7 200ng/ml + 5uM Rosiglitazone, last day CL316243 for 4 hours ",,

iPSC,BIOTIME,GPL10588,MBA_6955,"C4ELSR2 P10 Hystem BMP4 50ng/ml + Rosiglitazone 5uM, last day CL316243 10uM for 4 hours",,

iPSC,BIOTIME,GPL10588,MBA_6956,"C4ELSR2 P10 D14 Hystem BMP7 200ng/ml + Rosiglitazone 5uM, last day incubator set at 28C",,

iPSC,BIOTIME,GPL10588,MBA_6957,C4ELSR2 P10 D14 Confluence BMP7 200ng/ml + Rosiglitazone 5uM ,,

iPSC,BIOTIME,GPL10588,MBA_6958,C4ELSR2 P10 D14 Confluence BMP4 50ng/ml + Rosiglitazone 5uM ,,

iPSC,BIOTIME,GPL10588,MBA_6960,C4ELS5.5 P15 D14 Hystem BMP7 200ng/ml + Rosiglitazone 5uM ,,

iPSC,BIOTIME,GPL10588,MBA_6961,C4ELS5.5 P15 D14 Hystem BMP4 50ng/ml + Rosiglitazone 5uM ,,

iPSC,BIOTIME,GPL10588,MBA_6962,"C4ELS5.5 P15 D14 Hystem BMP7 200ng/ml + 5uM Rosiglitazone, last day CL316243 10uM for 4 hours ",,

iPSC,BIOTIME,GPL10588,MBA_6963,"C4ELS5.5 P15 Hystem BMP4 50ng/ml + Rosiglitazone 5uM, last day CL316243 10uM for 4 hours",,

iPSC,BIOTIME,GPL10588,MBA_6964,C4ELS5.5 P15 D14 Hystem BMP7 200ng/ml + Rosiglitazone 5uM last day incubator set at 28C,,

iPSC,BIOTIME,GPL10588,MBA_6965,C4ELS5.5 P15 D14 Confluence BMP7 200ng/ml + Rosiglitazone 5uM ,,

iPSC,BIOTIME,GPL10588,MBA_6966,C4ELS5.5 P15 D14 Confluence BMP4 50ng/ml + Rosiglitazone 5uM ,,

iPSC,BIOTIME,GPL10588,MBA_6967,C4ELS5.5 P17 ctrl,,

iPSC,BIOTIME,GPL10588,MBA_6969,J4 P10 D14 Hystem BMP7 200ng/ml + Rosiglitazone 5uM ,,

iPSC,BIOTIME,GPL10588,MBA_6970,J4 P10 D14 Hystem BMP4 50ng/ml + 5uM Rosiglitazone,,

iPSC,BIOTIME,GPL10588,MBA_6971,"J4 P10 D14 Hystem BMP7 200ng/ml + 5uM Rosiglitazone, last day CL316243 10uM for 4 hours ",,

iPSC,BIOTIME,GPL10588,MBA_6972,"J4 P10 D14 Hystem BMP4 50ng/ml + Rosiglitazone 5uM, last day CL316243 10uM for 4 hours",,

iPSC,BIOTIME,GPL10588,MBA_6973,"J4 P10 D14 Hystem D14 Hystem BMP7 200ng/ml + Rosiglitazone 5uM, last day incubator set at 28C",,

iPSC,BIOTIME,GPL10588,MBA_6974,J4 P10 D14 confluence BMP7 200ng/ml + Rosiglitazone 5uM ,,

iPSC,BIOTIME,GPL10588,MBA_6975,J4 P10 D14 confluence BMP4 50ng/ml + Rosiglitazone 5uM ,,

iPSC,BIOTIME,GPL10588,MBA_6977,C4ELSR13 P12 D14 Hystem BMP7 200ng/ml + Rosiglitazone 5uM ,,

iPSC,BIOTIME,GPL10588,MBA_6978,C4ELSR13 P12 D14 Hystem BMP4 50ng/ml + Rosiglitazone 5uM ,,

iPSC,BIOTIME,GPL10588,MBA_6979,"C4ELSR13 P12 D14 Hystem BMP7 200ng/ml + 5uM Rosiglitazone, last day CL316243 10uM for 4 hours ",,

iPSC,BIOTIME,GPL10588,MBA_6980,"C4ELSR13 P12 D14 Hystem BMP4 50ng/ml + Rosiglitazone 5uM, last day CL316243 10uM for 4 hours",,

iPSC,BIOTIME,GPL10588,MBA_6981,"C4ELSR13 P12 D14 Hystem BMP7 200ng/ml + Rosiglitazone 5uM, last day incubator set at 28C",,

iPSC,BIOTIME,GPL10588,MBA_6982,C4ELSR13 P12 D14 confluence BMP7 200ng/ml + Rosiglitazone 5uM,,

iPSC,BIOTIME,GPL10588,MBA_6983,C4ELSR13 P12 D14 confluence BMP4 50ng/ml + Rosiglitazone 5uM,,

iPSC,BIOTIME,GPL10588,MBA_6985,"C4ELS5.1 P18 D2 confluence protocol 1 patent 026 MDI: DMEM 10%FBS IBMX 1:100 (from stock 0.0115g/ml), Insulin 1:1000 (from 167mM stock), Dexamethasone 1:1000 (from 1mM stock)",,

iPSC,BIOTIME,GPL10588,MBA_6986,"C4ELS5.1 P18 D5 confluence protocol 1 patent 026 MDI: DMEM 10%FBS IBMX 1:100 (from stock 0.0115g/ml), Insulin 1:1000 (from 167mM stock), Dexamethasone 1:1000 (from 1mM stock)",,

iPSC,BIOTIME,GPL10588,MBA_6987,"C4ELS5.1 P18 D7 confluence protocol 1 patent 026 MDI: DMEM 10%FBS IBMX 1:100 (from stock 0.0115g/ml), Insulin 1:1000 (from 167mM stock), Dexamethasone 1:1000 (from 1mM stock)",,

iPSC,BIOTIME,GPL10588,MBA_6988,"C4ELS5.1 P18 D14 confluence protocol 1 patent 026 MDI: DMEM 10%FBS IBMX 1:100 (from stock 0.0115g/ml), Insulin 1:1000 (from 167mM stock), Dexamethasone 1:1000 (from 1mM stock)",,

iPSC,BIOTIME,GPL10588,MBA_6989,"C4ELS5.1 P18 D2 confluence protocol 2 patent 026 DMEM/F12 1uM bovine insulin,100nM hydrocortisone, 10ug/ml transferrin, 1nM thyronine, 1uM rosiglitazone, 33 uM biotin, 17uM pantothenic acid",,

iPSC,BIOTIME,GPL10588,MBA_6990,"C4ELS5.1 P18 D3 confluence protocol 2 patent 026 DMEM/F12 1uM bovine insulin,100nM hydrocortisone, 10ug/ml transferrin, 1nM thyronine, 1uM rosiglitazone, 33 uM biotin, 17uM pantothenic acid",,

iPSC,BIOTIME,GPL10588,MBA_6991,"C4ELS5.1 P18 D5 confluence protocol 2 patent 026 DMEM/F12 1uM bovine insulin,100nM hydrocortisone, 10ug/ml transferrin, 1nM thyronine, 1uM rosiglitazone, 33 uM biotin, 17uM pantothenic acid",,

iPSC,BIOTIME,GPL10588,MBA_6992,"C4ELS5.1 P18 D7 confluence protocol 2 patent 026 DMEM/F12 1uM bovine insulin,100nM hydrocortisone, 10ug/ml transferrin, 1nM thyronine, 1uM rosiglitazone, 33 uM biotin, 17uM pantothenic acid",,

iPSC,BIOTIME,GPL10588,MBA_6993,"C4ELS5.1 P18 D8 confluence protocol 2 patent 026 DMEM/F12 1uM bovine insulin,100nM hydrocortisone, 10ug/ml transferrin, 1nM thyronine, 1uM rosiglitazone, 33 uM biotin, 17uM pantothenic acid",,

iPSC,BIOTIME,GPL10588,MBA_6994,"C4ELS5.1 P18 D14 confluence protocol 2 patent 026 DMEM/F12 1uM bovine insulin,100nM hydrocortisone, 10ug/ml transferrin, 1nM thyronine, 1uM rosiglitazone, 33 uM biotin, 17uM pantothenic acid",,

iPSC,BIOTIME,GPL10588,MBA_6996,C4ELS5.1 P14 D14 Hystem BMP4 50ng/ml ,,

iPSC,BIOTIME,GPL10588,MBA_6997,C4ELS5.1 P14 D14 Hystem BMP4 50ng/ml + Rosiglitazone 5uM,,

iPSC,BIOTIME,GPL10588,MBA_6998,C4ELS5.1 P14 D14 Hystem BMP4 50ng/ml + Rosiglitazone 5uM + CL316243 10uM 4hrs last day,,

iPSC,BIOTIME,GPL10588,MBA_6999,C4ELS5.1 P14 D14 Hystem BMP7 200ng/ml + Rosiglitazone 5uM + CL316243 10uM 4hrs last day,,

iPSC,BIOTIME,GPL10588,MBA_7000,C4ELS5.1 P18 D14 Hystem BMP4 50ng/ml + Rosiglitazone 5uM,,

iPSC,BIOTIME,GPL10588,MBA_7001,C4ELS5.1 P18 D14 Hystem BMP4 50ng/ml + Rosiglitazone 5uM + BMP8B 100ng/ml,,

iPSC,BIOTIME,GPL10588,MBA_7002,C4ELS5.1 P18 D14 Hystem Rosiglitazone 5uM + BMP8B 100ng/ml,,

iPSC,BIOTIME,GPL10588,MBA_7003,C4ELS5.1 P18 D14 Hystem BMP8B 100ng/ml,,

iPSC,BIOTIME,GPL10588,MBA_7004,C4ELS5.1 P18 D14 Hystem BMP4 50ng/ml + Rosiglitazone 5uM + FGF21 50ng/ml last 2 days,,

iPSC,BIOTIME,GPL10588,MBA_7005,C4ELS5.1 P18 D14 Hystem BMP4 50ng/ml + Rosiglitazone 5uM + Forskolin 10uM last 4 hours,,

iPSC,BIOTIME,GPL10588,MBA_7006,C4ELS5.1 P18 D14 Hystem BMP4 50ng/ml + Rosiglitazone 5uM + CL316243 10uM 4hrs last day,,

iPSC,BIOTIME,GPL10588,MBA_7007,C4ELS5.1 P18 D21 Hystem BMP4 50ng/ml + Rosiglitazone 5uM,,

iPSC,BIOTIME,GPL10588,MBA_7008,C4ELS5.1 P18 D21 Hystem BMP4 50ng/ml + Rosiglitazone 5uM + BMP8B 100ng/ml,,

iPSC,BIOTIME,GPL10588,MBA_7009,C4ELS5.1 P18 D21 Hystem BMP4 50ng/ml + Rosiglitazone 5uM + FGF21 50ng/ml,,

iPSC,BIOTIME,GPL10588,MBA_7010,C4ELS5.1 P18 D21 Hystem BMP4 50ng/ml + Rosiglitazone 5uM + Forskolin 10uM ,,

iPSC,BIOTIME,GPL10588,MBA_7011,C4ELS5.1 P18 D21 Hystem BMP4 50ng/ml + Rosiglitazone 5uM + CL316243 10uM 4hrs last day,,

iPSC,BIOTIME,GPL10588,MBA_7012,C4ELS5.1 P14 D21 Hystem BMP4 10ng/ml,,

iPSC,BIOTIME,GPL10588,MBA_7013,C4ELS5.1 P14 D21 Hystem BMP4 50ng/ml + Rosiglitazone 5uM,,

iPSC,BIOTIME,GPL10588,MBA_7014,C4ELS5.1 P14 D21 confluent BMP4 50ng/ml + Rosiglitazone 5uM,,

iPSC,BIOTIME,GPL10588,MBA_7015,C4ELS5.1 P17 D21 Hystem BMP4 50ng/ml,,

iPSC,BIOTIME,GPL10588,MBA_7016,E3 P14 D14 Hystem BMP4 50ng/ml,,

iPSC,BIOTIME,GPL10588,MBA_7017,C4ELS5.1 P17 D14 Hystem BMP4 50ng/ml,,

iPSC,BIOTIME,GPL10588,MBA_7018,C4ELS5.1 P17 D14 Hystem BMP4 50ng/ml,,

iPSC,BIOTIME,GPL10588,MBA_7019,C4ELS5.1 P17 D14 Hystem BMP4 50ng/ml + Rosiglitazone 5uM ,,

iPSC,BIOTIME,GPL10588,MBA_7020,C4ELS5.1 P17 D14 Hystem BMP4 50ng/ml + Rosiglitazone 5uM ,,

iPSC,BIOTIME,GPL10588,MBA_7021,C4ELS5.1 P17 D21 Hystem BMP4 10ng/ml,,

iPSC,BIOTIME,GPL10588,MBA_7022,C4ELS5.1 P17 D21 Hystem BMP4 50ng/ml,,

iPSC,BIOTIME,GPL10588,MBA_7023,C4ELS5.1 P17 D21 Hystem BMP4 50ng/ml + Rosiglitazone 5uM,,

iPSC,BIOTIME,GPL10588,MBA_7024,C4ELS5.1 P17 D21 Hystem BMP4 50ng/ml + Rosiglitazone 5uM,,

iPSC,BIOTIME,GPL10588,MBA_7025,C4ELS5.1 P17 D14 Hystem Rosiglitazone 5uM ,,

iPSC,BIOTIME,GPL10588,MBA_7026,C4ELS5.1 P17 D14 Hystem Rosiglitazone 5uM ,,

iPSC,BIOTIME,GPL10588,MBA_7027,C4ELS5.1 P17 D21 Hystem Rosiglitazone 5uM ,,

iPSC,BIOTIME,GPL10588,MBA_7028,C4ELS5.1 P17 D21 Hystem Rosiglitazone 5uM ,,

iPSC,BIOTIME,GPL10588,MBA_7029,C4ELS5.1 P17 D21 MM TGFb3 10ng/ml,,

iPSC,BIOTIME,GPL10588,MBA_7030,C4ELS5.1 P17 D21 MM TGFb3 10ng/ml,,

iPSC,BIOTIME,GPL10588,MBA_7031,C4ELS5.1 P17 D21 Hystem TGFb3 10ng/ml,,

iPSC,BIOTIME,GPL10588,MBA_7032,C4ELS5.1 P17 D21 Hystem TGFb3 10ng/ml,,

iPSC,BIOTIME,GPL10588,MBA_7033,C4ELS5.1 P17 D20 confluence BMP4 50ng/ml,,

iPSC,BIOTIME,GPL10588,MBA_7034,C4ELS5.1 P17 D20 confluence BMP4 50ng/ml,,

iPSC,BIOTIME,GPL10588,MBA_7035,C4ELS5.1 P17 D20 confluence Rosiglitazone 5uM,,

iPSC,BIOTIME,GPL10588,MBA_7036,C4ELS5.1 P17 D20 confluence Rosiglitazone 5uM,,

iPSC,BIOTIME,GPL10588,MBA_7037,C4ELS5.1 P17 D20 confluence BMP4 50ng/ml + Rosiglitazone 5uM,,

iPSC,BIOTIME,GPL10588,MBA_7038,C4ELS5.1 P17 D20 confluence BMP4 50ng/ml + Rosiglitazone 5uM,,

iPSC,BIOTIME,GPL10588,MBA_7041,E3 P14 D14 Hystem BMP4 50ng/ml,,

iPSC,BIOTIME,GPL10588,MBA_7042,E3 P14 D14 Hystem Rosiglitazone 5uM,,

iPSC,BIOTIME,GPL10588,MBA_7043,E3 P14 D14 Hystem Rosiglitazone 5uM,,

iPSC,BIOTIME,GPL10588,MBA_7044,E3 P14 D14 Hystem BMP4 50ng/ml + Rosiglitazone 5uM,,

iPSC,BIOTIME,GPL10588,MBA_7045,E3 P14 D14 Hystem BMP4 50ng/ml + Rosiglitazone 5uM,,

iPSC,BIOTIME,GPL10588,MBA_7046,E3 P14 D21 Hystem BMP4 10ng/ml,,

iPSC,BIOTIME,GPL10588,MBA_7047,E3 P14 D21 Hystem BMP4 10ng/ml,,

iPSC,BIOTIME,GPL10588,MBA_7048,E3 P14 D21 Hystem BMP4 50ng/ml,,

iPSC,BIOTIME,GPL10588,MBA_7049,E3 P14 D21 Hystem BMP4 50ng/ml,,

iPSC,BIOTIME,GPL10588,MBA_7050,E3 P14 D21 Hystem Rosiglitazone 5uM,,

iPSC,BIOTIME,GPL10588,MBA_7051,E3 P14 D21 Hystem Rosiglitazone 5uM,,

iPSC,BIOTIME,GPL10588,MBA_7052,E3 P14 D21 Hystem TGFb3 10ng/ml,,

iPSC,BIOTIME,GPL10588,MBA_7053,E3 P14 D21 Hystem TGFb3 10ng/ml,,

iPSC,BIOTIME,GPL10588,MBA_7054,E3 P14 D21 Hystem BMP4 50ng/ml + Rosiglitazone 5uM,,

iPSC,BIOTIME,GPL10588,MBA_7055,E3 P14 D21 Hystem BMP4 50ng/ml + Rosiglitazone 5uM,,

iPSC,BIOTIME,GPL10588,MBA_7056,E3 P14 D21 MM TGFb3 10ng/ml,,

iPSC,BIOTIME,GPL10588,MBA_7057,E3 P14 D21 MM TGFb3 10ng/ml,,

iPSC,BIOTIME,GPL10588,MBA_7058,E3 P14 D21 confluence BMP4 50ng/ml + Rosiglitazone 5uM,,

iPSC,BIOTIME,GPL10588,MBA_7059,E3 P14 D21 confluence BMP4 50ng/ml + Rosiglitazone 5uM,,

iPSC,BIOTIME,GPL10588,MBA_7060,E3 P14 D21 confluence BMP4 50ng/ml,,

iPSC,BIOTIME,GPL10588,MBA_7061,E3 P14 D21 confluence BMP4 50ng/ml,,

iPSC,BIOTIME,GPL10588,MBA_7062,E3 P14 D21 confluence Rosiglitazone 5uM,,

iPSC,BIOTIME,GPL10588,MBA_7063,E3 P14 D21 confluence Rosiglitazone 5uM,,

AC,BIOTIME,GPL10588,MBA_7066,Human fetal brown preadipocytes Zenbio P5 D14 Hystem BMP4 50ng/ml,,

AC,BIOTIME,GPL10588,MBA_7067,Human fetal brown preadipocytes Zenbio P5 D14 Hystem BMP4 50ng/ml,,

AC,BIOTIME,GPL10588,MBA_7068,Human fetal brown preadipocytes Zenbio P5 D14 Hystem Rosiglitazone 5uM,,

AC,BIOTIME,GPL10588,MBA_7069,Human fetal brown preadipocytes Zenbio P5 D14 Hystem Rosiglitazone 5uM,,

AC,BIOTIME,GPL10588,MBA_7070,Human fetal brown preadipocytes Zenbio P5 D14 Hystem BMP4 50ng/ml + Rosiglitazone 5uM,,

AC,BIOTIME,GPL10588,MBA_7071,Human fetal brown preadipocytes Zenbio P5 D14 Hystem BMP4 50ng/ml + Rosiglitazone 5uM,,

AC,BIOTIME,GPL10588,MBA_7072,Human fetal brown preadipocytes Zenbio P5 D21 Hystem TGFb3 10ng/ml,,

AC,BIOTIME,GPL10588,MBA_7073,Human fetal brown preadipocytes Zenbio P5 D21 Hystem TGFb3 10ng/ml,,

AC,BIOTIME,GPL10588,MBA_7074,Human fetal brown preadipocytes Zenbio P5 D21 Hystem BMP4 10ng/ml,,

AC,BIOTIME,GPL10588,MBA_7075,Human fetal brown preadipocytes Zenbio P5 D21 Hystem BMP4 10ng/ml,,

AC,BIOTIME,GPL10588,MBA_7076,Human fetal brown preadipocytes Zenbio P5 D21 Hystem BMP4 50ng/ml,,

AC,BIOTIME,GPL10588,MBA_7077,Human fetal brown preadipocytes Zenbio P5 D21 Hystem BMP4 50ng/ml,,

AC,BIOTIME,GPL10588,MBA_7078,Human fetal brown preadipocytes Zenbio P5 D21 Hystem Rosiglitazone 5uM,,

AC,BIOTIME,GPL10588,MBA_7079,Human fetal brown preadipocytes Zenbio P5 D21 Hystem Rosiglitazone 5uM,,

AC,BIOTIME,GPL10588,MBA_7080,Human fetal brown preadipocytes Zenbio P5 D21 Hystem BMP4 50ng/ml + Rosiglitazone 5uM,,

AC,BIOTIME,GPL10588,MBA_7081,Human fetal brown preadipocytes Zenbio P5 D21 Hystem BMP4 50ng/ml + Rosiglitazone 5uM,,

AC,BIOTIME,GPL10588,MBA_7082,Human fetal brown preadipocytes Zenbio P5 D21 MM TGFb3 10ng/ml,,

AC,BIOTIME,GPL10588,MBA_7083,Human fetal brown preadipocytes Zenbio P5 D21 MM TGFb3 10ng/ml,,

AC,BIOTIME,GPL10588,MBA_7084,Human fetal brown preadipocytes Zenbio P5 D21 confluence BMP4 10ng/ml,,

AC,BIOTIME,GPL10588,MBA_7085,Human fetal brown preadipocytes Zenbio P5 D21 confluence BMP4 10ng/ml,,

AC,BIOTIME,GPL10588,MBA_7086,Human fetal brown preadipocytes Zenbio P5 D21 confluence Rosiglitazone 5uM,,

AC,BIOTIME,GPL10588,MBA_7087,Human fetal brown preadipocytes Zenbio P5 D21 confluence Rosiglitazone 5uM,,

AC,BIOTIME,GPL10588,MBA_7088,Human fetal brown preadipocytes Zenbio P5 D21 confluence BMP4 50ng/ml + Rosiglitazone 5uM,,

AC,BIOTIME,GPL10588,MBA_7089,Human fetal brown preadipocytes Zenbio P5 D21 confluence BMP4 50ng/ml + Rosiglitazone 5uM,,

AC,BIOTIME,GPL10588,MBA_7093,Skeletal myoblasts Zenbio P7 D14 Hystem BMP4 50ng/ml,,

AC,BIOTIME,GPL10588,MBA_7094,Skeletal myoblasts Zenbio P7 D14 Hystem BMP4 50ng/ml,,

AC,BIOTIME,GPL10588,MBA_7095,Skeletal myoblasts Zenbio P7 D14 Hystem Rosigliatazone 5uM,,

AC,BIOTIME,GPL10588,MBA_7096,Skeletal myoblasts Zenbio P7 D14 Hystem Rosigliatazone 5uM,,

AC,BIOTIME,GPL10588,MBA_7097,Skeletal myoblasts Zenbio P7 D14 Hystem BMP4 50ng/ml + Rosigliatazone 5uM,,

AC,BIOTIME,GPL10588,MBA_7098,Skeletal myoblasts Zenbio P7 D14 Hystem BMP4 50ng/ml + Rosigliatazone 5uM,,

AC,BIOTIME,GPL10588,MBA_7099,Skeletal myoblasts Zenbio P7 D21 Hystem BMP4 10ng/ml,,

AC,BIOTIME,GPL10588,MBA_7100,Skeletal myoblasts Zenbio P7 D21 Hystem BMP4 10ng/ml,,

AC,BIOTIME,GPL10588,MBA_7101,Skeletal myoblasts Zenbio P7 D21 Hystem BMP4 50ng/ml + Rosiglitazone 5uM,,

AC,BIOTIME,GPL10588,MBA_7102,Skeletal myoblasts Zenbio P7 D21 Hystem BMP4 50ng/ml + Rosiglitazone 5uM,,

AC,BIOTIME,GPL10588,MBA_7103,Skeletal myoblasts Zenbio P7 D21 Hystem BMP4 50ng/ml ,,

AC,BIOTIME,GPL10588,MBA_7104,Skeletal myoblasts Zenbio P7 D21 Hystem BMP4 50ng/ml ,,

AC,BIOTIME,GPL10588,MBA_7105,Skeletal myoblasts Zenbio P7 D21 Hystem Rosiglitazone 5uM,,

AC,BIOTIME,GPL10588,MBA_7106,Skeletal myoblasts Zenbio P7 D21 Hystem Rosiglitazone 5uM,,

AC,BIOTIME,GPL10588,MBA_7107,Skeletal myoblasts Zenbio P7 D21 confluence BMP4 50ng/ml + Rosigliatazone 5uM,,

AC,BIOTIME,GPL10588,MBA_7108,Skeletal myoblasts Zenbio P7 D21 confluence BMP4 50ng/ml + Rosigliatazone 5uM,,

AC,BIOTIME,GPL10588,MBA_7109,Skeletal myoblasts Zenbio P7 D21 confluence BMP4 50ng/ml,,

AC,BIOTIME,GPL10588,MBA_7110,Skeletal myoblasts Zenbio P7 D21confluence Rosigliatazone 5uM,,

AC,BIOTIME,GPL10588,MBA_7111,Skeletal myoblasts Zenbio P7 D21 MM TGFb3 10ng/ml,,

AC,BIOTIME,GPL10588,MBA_7112,Skeletal myoblasts Zenbio P7 D21 MM TGFb3 10ng/ml,,

iPSC,BIOTIME,GPL10588,MBA_7234,ESI NP110SM P10 D14 Hystem BMP4 50ng/ml,,

iPSC,BIOTIME,GPL10588,MBA_7235,ESI NP110SM P10 D14 Hystem BMP4 50ng/ml + rosiglitazone 5uM,,

iPSC,BIOTIME,GPL10588,MBA_7236,ESI NP110SM P10 D14 Hystem rosiglitazone 5uM,,

iPSC,BIOTIME,GPL10588,MBA_7237,ESI NP110SM P10 D14 Hystem BMP4 50ng/ml + rosiglitazone 5uM T3 2nM+ last 4 hours CL316243 10uM,,

iPSC,BIOTIME,GPL10588,MBA_7240,C4ELSR2 P12 D14 Hystem BMP4 50ng/ml + Rosiglitazone 5uM,,

iPSC,BIOTIME,GPL10588,MBA_7241,C4ELSR2 P12 D14 Hystem BMP4 50ng/ml + Rosiglitazone 5uM,,

iPSC,BIOTIME,GPL10588,MBA_7242,C4ELSR2 P12 D14 Hystem BMP4 50ng/ml ,,

iPSC,BIOTIME,GPL10588,MBA_7243,C4ELSR2 P12 D14 Hystem BMP4 50ng/ml ,,

iPSC,BIOTIME,GPL10588,MBA_7244,C4ELSR2 P12 D14 Hystem Rosiglitazone 5uM,,

iPSC,BIOTIME,GPL10588,MBA_7245,C4ELSR2 P12 D14 Hystem Rosiglitazone 5uM,,

iPSC,BIOTIME,GPL10588,MBA_7246,"SM30 P17 D14 Osteo Exosome 20140814-SM30GM, 125e6 particles Exo SM30 GM",,

iPSC,BIOTIME,GPL10588,MBA_7247,"SM30 P17 D14 Osteo Exosome 20140814-SM30IM, 750e6 particles Exo SM30 IM (incomplete chondro) ",,

iPSC,BIOTIME,GPL10588,MBA_7248,SM30 P17 D14 Osteo Incomplete medium + betaglycerophosphate + BMP2 + TGFb3 w/o exo,,

iPSC,BIOTIME,GPL10588,MBA_7250,7SMOO6 P11 D14 Hystem BMP4 50ng/ml,,

iPSC,BIOTIME,GPL10588,MBA_7251,7SMOO6 P11 D14 Hystem BMP4 50ng/ml + rosiglitazone 5uM,,

iPSC,BIOTIME,GPL10588,MBA_7252,7SMOO6 P11 D14 Hystem rosiglitazone 5uM,,

iPSC,BIOTIME,GPL10588,MBA_7253,7SMOO6 P11 D14 Hystem BMP4 50ng/ml + rosiglitazone 5uM + last 4 hours CL316243 10uM,,

iPSC,BIOTIME,GPL10588,MBA_7254,7SMOO6 P11 D14 Hystem BMP4 50ng/ml + rosiglitazone 5uM + T3 2nM + last 4 hours CL316243 10uM ,,

iPSC,BIOTIME,GPL10588,MBA_7256,C4ELSR6 P13 D14 Hystem rosiglitazone 5uM,,

iPSC,BIOTIME,GPL10588,MBA_7257,C4ELSR6 P13 D14 Hystem BMP4 50ng/ml,,

iPSC,BIOTIME,GPL10588,MBA_7258,C4ELSR6 P13 D14 Hystem BMP4 50ng/ml + rosiglitazone 5uM,,

iPSC,BIOTIME,GPL10588,MBA_7259,C4ELSR6 P13 D14 Hystem BMP4 50ng/ml + rosiglitazone 5uM + T3 2nM + last 4 hrs CL316243 10uM,,

iPSC,BIOTIME,GPL10588,MBA_7260,C4ELSR6 P13 D14 Hystem BMP4 10ng/ml + rosiglitazone 1uM + T3 2nM + last 4 hrs CL316243 10uM,,

iPSC,BIOTIME,GPL10588,MBA_7262,7SMOO12 P12 D14 Hystem BMP4 50ng/ml,,

iPSC,BIOTIME,GPL10588,MBA_7263,7SMOO12 P12 D14 Hystem BMP4 50ng/ml + rosiglitazone 5uM,,

iPSC,BIOTIME,GPL10588,MBA_7264,7SMOO12 P12 D14 Hystem rosiglitazone 5uM,,

iPSC,BIOTIME,GPL10588,MBA_7265,7SMOO12 P12 D14 Hystem BMP4 50ng/ml + rosiglitazone 5uM + last 4 hours CL316243 10uM,,

iPSC,BIOTIME,GPL10588,MBA_7266,7SMOO12 P12 D14 Hystem BMP4 50ng/ml + rosiglitazone 5uM + T3 uM + last 4 hours CL316243 10uM ,,

iPSC,BIOTIME,GPL10588,MBA_7268,EN18 P19 D14 Hystem rosiglitazone 5uM,,

iPSC,BIOTIME,GPL10588,MBA_7269,EN18 P19 D14 Hystem BMP4 50ng/ml,,

iPSC,BIOTIME,GPL10588,MBA_7270,EN18 P19 D14 Hystem BMP4 50ng/ml + rosiglitazone 5uM,,

iPSC,BIOTIME,GPL10588,MBA_7271,EN18 P19 D14 Hystem BMP4 50ng/ml + rosiglitazone 5uM T3 2nM+ last 4 hours CL316243 10uM,,

iPSC,BIOTIME,GPL10588,MBA_7273,EN55 P15 D14 Hystem rosiglitazone 5uM,,

iPSC,BIOTIME,GPL10588,MBA_7274,EN55 P15 D14 Hystem BMP4 50ng/ml,,

iPSC,BIOTIME,GPL10588,MBA_7275,EN55 P15 D14 Hystem BMP4 50ng/ml + rosiglitazone 5uM,,

iPSC,BIOTIME,GPL10588,MBA_7276,EN55 P15 D14 Hystem BMP4 50ng/ml + rosiglitazone 5uM T3 2nM+ last 4 hours CL316243 10uM,,

iPSC,BIOTIME,GPL10588,MBA_7278,Z3 P13 D14 Hystem BMP4 50ng/ml,,

iPSC,BIOTIME,GPL10588,MBA_7279,Z3 P13 D14 Hystem rosiglitazone 5uM,,

iPSC,BIOTIME,GPL10588,MBA_7280,Z3 P13 D14 Hystem BMP4 50ng/ml + rosiglitazone 5uM,,

iPSC,BIOTIME,GPL10588,MBA_7281,Z3 P13 D14 Hystem BMP4 50ng/ml + rosiglitazone 5uM + last 4 hours CL316243 10uM,,

iPSC,BIOTIME,GPL10588,MBA_7282,Z3 P13 D14 Hystem BMP4 50ng/ml + rosiglitazone 5uM + T3 2nM + last 4 hours CL316243 10uM,,

iPSC,BIOTIME,GPL10588,MBA_7284,EN26 P15 D14 Hystem rosiglitazone 5uM,,

iPSC,BIOTIME,GPL10588,MBA_7285,EN26 P15 D14 Hystem BMP4 50ng/ml,,

iPSC,BIOTIME,GPL10588,MBA_7286,EN26 P15 D14 Hystem BMP4 50ng/ml + rosiglitazone 5uM,,

iPSC,BIOTIME,GPL10588,MBA_7287,EN26 P15 D14 Hystem BMP4 50ng/ml + rosiglitazone 5uM T3 2nM+ last 4 hours CL316243 10uM,,

iPSC,BIOTIME,GPL10588,MBA_7289,EN1 P17 D14 Hystem BMP4 50ng/ml,,

iPSC,BIOTIME,GPL10588,MBA_7290,EN1 P17 D14 Hystem BMP4 50ng/ml + rosiglitazone 5uM,,

iPSC,BIOTIME,GPL10588,MBA_7291,EN1 P17 D14 Hystem rosiglitazone 5uM,,

iPSC,BIOTIME,GPL10588,MBA_7292,EN1 P17 D14 Hystem BMP4 50ng/ml + rosiglitazone 5uM T3 2nM+ last 4 hours CL316243 10uM,,

iPSC,BIOTIME,GPL10588,MBA_7294,W8 P12 D14 Hystem BMP4 50ng/ml,,

iPSC,BIOTIME,GPL10588,MBA_7295,W8 P12 D14 Hystem BMP4 50ng/ml + rosiglitazone 5uM,,

iPSC,BIOTIME,GPL10588,MBA_7296,W8 P12 D14 Hystem rosiglitazone 5uM,,

iPSC,BIOTIME,GPL10588,MBA_7297,W8 P12 D14 Hystem BMP4 50ng/ml + rosiglitazone 5uM + T3 2nM + last 4 hours CL316243 10uM,,

iPSC,BIOTIME,GPL10588,MBA_7299,7PEND20 P13 D14 Hystem BMP4 50ng/ml,,

iPSC,BIOTIME,GPL10588,MBA_7300,7PEND20 P13 D14 Hystem BMP4 50ng/ml + rosiglitazone 5uM,,

iPSC,BIOTIME,GPL10588,MBA_7301,7PEND20 P13 D14 Hystem rosiglitazone 5uM ,,

iPSC,BIOTIME,GPL10588,MBA_7302,7PEND20 P13 D14 Hystem BMP4 50ng/ml + rosiglitazone 5uM + T3 2nM + last 4 hours CL316243 10uM,,

AC,BIOTIME,GPL10588,MBA_7304,Skeletal myoblasts Zenbio P10 D21 Hystem TGFb3 10ng/ml,,

AC,BIOTIME,GPL10588,MBA_7305,Skeletal myoblasts Zenbio P10 D21 Hystem TGFb3 10ng/ml,,

AC,BIOTIME,GPL10588,MBA_7306,Skeletal myoblasts Zenbio P10 D21 Hystem BMP4 10ng/ml ,,

AC,BIOTIME,GPL10588,MBA_7307,Skeletal myoblasts Zenbio P10 D21 Hystem BMP4 10ng/ml ,,

AC,BIOTIME,GPL10588,MBA_7308,Skeletal myoblasts Zenbio P10 D21 Hystem BMP4 50ng/ml ,,

AC,BIOTIME,GPL10588,MBA_7309,Skeletal myoblasts Zenbio P10 D21 Hystem BMP4 50ng/ml ,,

AC,BIOTIME,GPL10588,MBA_7310,Skeletal myoblasts Zenbio P10 D21 Hystem Rosiglitazone 5uM,,

AC,BIOTIME,GPL10588,MBA_7311,Skeletal myoblasts Zenbio P10 D21 confluence BMP4 50ng/ml + rosigliatazone 5uM,,

iPSC,BIOTIME,GPL10588,MBA_7312,C4ELS5.1 P18 D21 Hystem TGFb3 10ng/ml,,

iPSC,BIOTIME,GPL10588,MBA_7313,C4ELS5.1 P18 D21 Hystem TGFb3 10ng/ml,,

iPSC,BIOTIME,GPL10588,MBA_7314,C4ELS5.1 P18 D21 Hystem BMP4 10ng/ml ,,

iPSC,BIOTIME,GPL10588,MBA_7315,C4ELS5.1 P18 D21 Hystem BMP4 10ng/ml ,,

iPSC,BIOTIME,GPL10588,MBA_7316,C4ELS5.1 P18 D21 Hystem BMP4 50ng/ml ,,

iPSC,BIOTIME,GPL10588,MBA_7317,C4ELS5.1 P18 D21 Hystem BMP4 50ng/ml ,,

iPSC,BIOTIME,GPL10588,MBA_7318,C4ELS5.1 P18 D21 confluence rosiglitazone 5uM,,

iPSC,BIOTIME,GPL10588,MBA_7319,C4ELS5.1 P18 D21 confluence rosiglitazone 5uM,,

iPSC,BIOTIME,GPL10588,MBA_7321,J8 P13 D14 Hystem BMP4 50ng/ml,,

iPSC,BIOTIME,GPL10588,MBA_7322,J8 P13 D14 Hystem BMP4 50ng/ml + rosiglitazone 5uM ,,

iPSC,BIOTIME,GPL10588,MBA_7323,J8 P13 D14 Hystem Rosiglitazone 5uM ,,

iPSC,BIOTIME,GPL10588,MBA_7324,J8 P13 D14 Hystem BMP4 50ng/ml + rosiglitazone 5uM + T3 2nM + last 4 hrs CL316243 10uM,,

iPSC,BIOTIME,GPL10588,MBA_7325,C4ELSR12 P16 ctrl,,

iPSC,BIOTIME,GPL10588,MBA_7326,C4ELSR12 P16 D14 Hystem BMP4 50ng/ml,,

iPSC,BIOTIME,GPL10588,MBA_7327,C4ELSR12 P16 D14 Hystem BMP4 50ng/ml + rosiglitazone 5uM,,

iPSC,BIOTIME,GPL10588,MBA_7328,C4ELSR12 P16 D14 Hystem Rosiglitazone 5uM,,

iPSC,BIOTIME,GPL10588,MBA_7329,C4ELSR12 P16 D14 Hystem BMP4 10ng/ml + rosiglitazone 1uM + T3 2nM + last 4 hrs CL316243 10uM,,

iPSC,BIOTIME,GPL10588,MBA_7330,E3 P17 D14 Hystem BMP4 50ng/ml + rosiglitazone 5uM,,

iPSC,BIOTIME,GPL10588,MBA_7331,E3 P17 D14 Hystem BMP4 50ng/ml + rosiglitazone 5uM,,

iPSC,BIOTIME,GPL10588,MBA_7332,E3 P17 D14 Hystem BMP4 50ng/ml,,

iPSC,BIOTIME,GPL10588,MBA_7333,E3 P17 D14 Hystem BMP4 50ng/ml,,

iPSC,BIOTIME,GPL10588,MBA_7334,E3 P17 D14 Hystem rosiglitazone 5uM,,

iPSC,BIOTIME,GPL10588,MBA_7335,E3 P17 D14 Hystem rosiglitazone 5uM,,

AC,BIOTIME,GPL10588,MBA_7336,Skeletal myoblasts Zenbio P10 D21 confluence BMP4 50ng/ml ,,

AC,BIOTIME,GPL10588,MBA_7337,Skeletal myoblasts Zenbio P10 D21 confluence BMP4 50ng/ml ,,

AC,BIOTIME,GPL10588,MBA_7338,Skeletal myoblasts Zenbio P10 D21 confluence rosiglitazone 5uM,,

AC,BIOTIME,GPL10588,MBA_7339,Skeletal myoblasts Zenbio P10 D21 confluence rosiglitazone 5uM,,

iPSC,BIOTIME,GPL10588,MBA_7425,E3 P14 D21 Hystem BMP4 10ng/ml,,

iPSC,BIOTIME,GPL10588,MBA_7426,E3 P17 D21 Hystem BMP4 50ng/ml,,

iPSC,BIOTIME,GPL10588,MBA_7427,E3 P16 D14 Hystem BMP4 50ng/ml ,,

iPSC,BIOTIME,GPL10588,MBA_7428,E3 P16 D14 Hystem BMP4 50ng/ml ,,

iPSC,BIOTIME,GPL10588,MBA_7429,E3 P17 D21 Hystem Rosiglitazone 5uM,,

iPSC,BIOTIME,GPL10588,MBA_7430,E3 P17 D21 Hystem Rosiglitazone 5uM,,

iPSC,BIOTIME,GPL10588,MBA_7431,E3 P16 D14 Hystem Rosiglitazone 5uM,,

iPSC,BIOTIME,GPL10588,MBA_7432,E3 P16 D14 Hystem Rosiglitazone 5uM,,

iPSC,BIOTIME,GPL10588,MBA_7433,E3 P17 D21 Hystem BMP4 50ng/ml + Rosiglitazone 5uM,,

iPSC,BIOTIME,GPL10588,MBA_7434,E3 P17 D21 Hystem BMP4 50ng/ml + Rosiglitazone 5uM,,

iPSC,BIOTIME,GPL10588,MBA_7435,E3 P16 D14 Hystem BMP4 50ng/ml + Rosiglitazone 5uM,,

iPSC,BIOTIME,GPL10588,MBA_7436,E3 P16 D14 Hystem BMP4 50ng/ml + Rosiglitazone 5uM,,

iPSC,BIOTIME,GPL10588,MBA_7437,E3 P17 D21 Hystem TGFb3 10ng/ml,,

iPSC,BIOTIME,GPL10588,MBA_7438,E3 P17 D21 Hystem TGFb3 10ng/ml,,

iPSC,BIOTIME,GPL10588,MBA_7439,E3 P16 D14 Hystem BMP4 50ng/ml + rosiglitazone 5uM + T3 2nM + last 4 hours CL316243 10uM ,,

iPSC,BIOTIME,GPL10588,MBA_7440,E3 P16 D14 Hystem BMP4 50ng/ml + rosiglitazone 5uM + T3 2nM + last 4 hours CL316243 10uM ,,

iPSC,BIOTIME,GPL10588,MBA_7441,E3 P16 D14 Hystem BMP4 10ng/ml + rosiglitazone 1uM + T3 2nM + last 4 hours CL316243 10uM ,,

iPSC,BIOTIME,GPL10588,MBA_7442,E3 P16 D14 Hystem BMP4 10ng/ml + rosiglitazone 1uM + T3 2nM + last 4 hours CL316243 10uM ,,

iPSC,BIOTIME,GPL10588,MBA_7444,E3 P16 D14 Hystem rosiglitazone 1uM + T3 2nM + last 2 days FGF21 50ng/ml + last 4 hours CL316243 10uM ,,

iPSC,BIOTIME,GPL10588,MBA_7445,E3 P17 D21 confluence BMP4 50ng/ml + Rosiglitazone 5uM,,

iPSC,BIOTIME,GPL10588,MBA_7446,E3 P17 D21 confluence BMP4 50ng/ml + Rosiglitazone 5uM,,

iPSC,BIOTIME,GPL10588,MBA_7447,E3 P17 D21 confluence BMP4 50ng/ml ,,

iPSC,BIOTIME,GPL10588,MBA_7448,E3 P17 D21 confluence BMP4 50ng/ml ,,

iPSC,BIOTIME,GPL10588,MBA_7449,E3 P17 D21 confluence Rosiglitazone 5uM,,

iPSC,BIOTIME,GPL10588,MBA_7450,E3 P17 D21 confluence Rosiglitazone 5uM,,

iPSC,BIOTIME,GPL10588,MBA_7451,E72 P13 D14 Hystem BMP4 50ng/ml,,

iPSC,BIOTIME,GPL10588,MBA_7452,E72 P13 D14 Hystem BMP4 50ng/ml,,

iPSC,BIOTIME,GPL10588,MBA_7453,E72 P13 D14 Hystem rosiglitazone 5uM,,

iPSC,BIOTIME,GPL10588,MBA_7454,E72 P13 D14 Hystem rosiglitazone 5uM,,

iPSC,BIOTIME,GPL10588,MBA_7455,E72 P13 D14 Hystem BMP4 50ng/ml + rosiglitazone 5uM,,

iPSC,BIOTIME,GPL10588,MBA_7456,E72 P13 D14 Hystem BMP4 50ng/ml + rosiglitazone 5uM,,

iPSC,BIOTIME,GPL10588,MBA_7457,E72 P13 D14 Hystem BMP4 50ng/ml + rosiglitazone 5uM + T3 2nM + last 4 hours CL316243 10uM ,,

iPSC,BIOTIME,GPL10588,MBA_7458,E72 P13 D14 Hystem TGFb3 10ng/ml,,

iPSC,BIOTIME,GPL10588,MBA_7459,E72 P13 D14 confluence rosiglitazone 5uM,,

iPSC,BIOTIME,GPL10588,MBA_7460,E72 P13 D14 confluence BMP4 50ng/ml + rosiglitazone 5uM,,

iPSC,BIOTIME,GPL10588,MBA_7461,E72 P13 D14 confluence BMP4 50ng/ml,,

iPSC,BIOTIME,GPL10588,MBA_7462,C4ELSR2 P11 ctrl,,

iPSC,BIOTIME,GPL10588,MBA_7463,C4ELSR2 P12 D21 Hystem BMP4 50ng/ml ,,

iPSC,BIOTIME,GPL10588,MBA_7464,C4ELSR2 P12 D21 Hystem BMP4 50ng/ml ,,

iPSC,BIOTIME,GPL10588,MBA_7465,C4ELSR2 P12 D21 Hystem BMP4 10ng/ml ,,

iPSC,BIOTIME,GPL10588,MBA_7466,C4ELSR2 P12 D21 Hystem BMP4 10ng/ml ,,

iPSC,BIOTIME,GPL10588,MBA_7467,C4ELSR2 P12 D21 Hystem Rosiglitazone 5uM,,

iPSC,BIOTIME,GPL10588,MBA_7468,C4ELSR2 P12 D21 Hystem Rosiglitazone 5uM,,

iPSC,BIOTIME,GPL10588,MBA_7469,C4ELSR2 P12 D21 Hystem BMP4 50ng/ml + Rosiglitazone 5uM,,

iPSC,BIOTIME,GPL10588,MBA_7470,C4ELSR2 P12 D21 Hystem BMP4 50ng/ml + Rosiglitazone 5uM,,

iPSC,BIOTIME,GPL10588,MBA_7471,C4ELSR2 P12 D21 Hystem TGFb3 10ng/ml ,,

iPSC,BIOTIME,GPL10588,MBA_7472,C4ELSR2 P12 D21 Hystem TGFb3 10ng/ml ,,

iPSC,BIOTIME,GPL10588,MBA_7473,C4ELSR2 P12 D21 MM TGFb3 10ng/ml ,,

iPSC,BIOTIME,GPL10588,MBA_7474,C4ELSR2 P12 D21 MM TGFb3 10ng/ml ,,

iPSC,BIOTIME,GPL10588,MBA_7475,C4ELSR2 P12 D21 confluence BMP4 50ng/ml + Rosiglitazone 5uM,,

iPSC,BIOTIME,GPL10588,MBA_7476,C4ELSR2 P12 D21 confluence BMP4 50ng/ml + Rosiglitazone 5uM,,

iPSC,BIOTIME,GPL10588,MBA_7477,C4ELSR2 P12 D21 confluence BMP4 50ng/ml ,,

iPSC,BIOTIME,GPL10588,MBA_7478,C4ELSR2 P12 D21 confluence BMP4 50ng/ml ,,

iPSC,BIOTIME,GPL10588,MBA_7479,C4ELSR2 P12 D21 confluence Rosiglitazone 5uM,,

iPSC,BIOTIME,GPL10588,MBA_7480,C4ELSR2 P12 D21 confluence Rosiglitazone 5uM,,

iPSC,BIOTIME,GPL10588,MBA_7481,C4ELSR2 P11 D14 Hystem Rosiglitazone 5uM,,

iPSC,BIOTIME,GPL10588,MBA_7482,C4ELSR2 P11 D14 Hystem BMP4 50ng/ml + Rosiglitazone 5uM,,

iPSC,BIOTIME,GPL10588,MBA_7483,C4ELSR2 P11 D14 Hystem BMP4 50ng/ml + Rosi T3 (Liothyronine 2nM) + last 4 hours CL316243 10uM ,,

iPSC,BIOTIME,GPL10588,MBA_7484,C4ELSR2 P11 D14 Hystem BMP4 10ng/ml + Rosiglitazone 1uM + T3 2nM + 4 hours CL316243 10uM,,

iPSC,BIOTIME,GPL10588,MBA_7485,C4ELSR2 P11 D14 Hystem Rosiglitazone 1uM + T3 2nM + last 4 hours CL316243 10uM,,

iPSC,BIOTIME,GPL10588,MBA_7486,C4ELSR2 P11 D14 Hystem BMP4 10ng/ml + Rosiglitazone 1uM + T3 2nM + last day (~16-24 hours) CL316243 10uM,,

iPSC,BIOTIME,GPL10588,MBA_7488,C4ELS5.5 P13 D14 Hystem BMP4 50ng/ml ,,

iPSC,BIOTIME,GPL10588,MBA_7489,C4ELS5.5 P13 D14 Hystem BMP4 50ng/ml ,,

iPSC,BIOTIME,GPL10588,MBA_7490,C4ELS5.5 P11 D14 Hystem BMP4 50ng/ml ,,

iPSC,BIOTIME,GPL10588,MBA_7491,C4ELS5.5 P13 D14 Hystem Rosiglitazone 5uM ,,

iPSC,BIOTIME,GPL10588,MBA_7492,C4ELS5.5 P13 D14 Hystem Rosiglitazone 5uM ,,

iPSC,BIOTIME,GPL10588,MBA_7493,C4ELS5.5 P11 D14 Hystem Rosiglitazone 5uM ,,

iPSC,BIOTIME,GPL10588,MBA_7494,C4ELS5.5 P13 D14 Hystem BMP4 50ng/ml + Rosiglitazone 5uM ,,

iPSC,BIOTIME,GPL10588,MBA_7495,C4ELS5.5 P13 D14 Hystem BMP4 50ng/ml + Rosiglitazone 5uM ,,

iPSC,BIOTIME,GPL10588,MBA_7496,C4ELS5.5 P11 D14 Hystem BMP4 50ng/ml + Rosiglitazone 5uM ,,

iPSC,BIOTIME,GPL10588,MBA_7497,C4ELS5.5 P13 D14 Hystem BMP4 50ng/ml + Rosiglitazone 5uM + T3 2nM + last 4 hours CL316243 10uM ,,

iPSC,BIOTIME,GPL10588,MBA_7498,C4ELS5.5 P13 D14 Hystem BMP4 50ng/ml + Rosiglitazone 5uM + T3 2nM + last 4 hours CL316243 10uM ,,

iPSC,BIOTIME,GPL10588,MBA_7499,C4ELS5.5 P11 D14 Hystem BMP4 50ng/ml + Rosiglitazone 5uM + T3 2nM + last 4 hours CL316243 10uM ,,

iPSC,BIOTIME,GPL10588,MBA_7500,C4ELS5.5 P13 D14 Hystem BMP4 10ng/ml + Rosiglitazone 1uM + T3 2nM + last 4 hours CL316243 10uM,,

iPSC,BIOTIME,GPL10588,MBA_7501,C4ELS5.5 P13 D14 Hystem BMP4 10ng/ml + Rosiglitazone 1uM + T3 2nM + last 4 hours CL316243 10uM,,

iPSC,BIOTIME,GPL10588,MBA_7502,C4ELS5.5 P11 D14 Hystem BMP4 10ng/ml + Rosiglitazone 1uM + T3 2nM + last 4 hours CL316243 10uM,,

iPSC,BIOTIME,GPL10588,MBA_7503,C4ELS5.5 P13 D14 Hystem Rosiglitazone 5uM + T3 2nM + last 4 hours CL316243 10uM ,,

iPSC,BIOTIME,GPL10588,MBA_7504,C4ELS5.5 P11 D14 Hystem Rosiglitazone 1uM + T3 2nM + last 4 hours CL316243 10uM,,

iPSC,BIOTIME,GPL10588,MBA_7505,C4ELS5.5 P13 D14 Hystem BMP4 10ng/ml + Rosiglitazone 1uM + T3 2nM + last 2 days FGF21 50ng/ml + last 4 hours CL316243 10uM,,

iPSC,BIOTIME,GPL10588,MBA_7506,C4ELS5.1 P17 D14 Hystem BMP4 50ng/ml ,,

iPSC,BIOTIME,GPL10588,MBA_7507,C4ELS5.1 P17 D14 Hystem BMP4 50ng/ml ,,

iPSC,BIOTIME,GPL10588,MBA_7508,C4ELS5.1 P17 D14 Hystem Rosiglitazone 5uM ,,

iPSC,BIOTIME,GPL10588,MBA_7509,C4ELS5.1 P17 D14 Hystem Rosiglitazone 5uM ,,

iPSC,BIOTIME,GPL10588,MBA_7510,C4ELS5.1 P17 D14 Hystem BMP4 50ng/ml + Rosiglitazone 5uM,,

iPSC,BIOTIME,GPL10588,MBA_7511,C4ELS5.1 P17 D14 Hystem BMP4 50ng/ml + Rosiglitazone 5uM,,

iPSC,BIOTIME,GPL10588,MBA_7512,C4ELS5.1 P17 D14 Hystem BMP4 50ng/ml + Rosiglitazone 5uM + T3 2nM last 4 hours CL316243 10uM ,,

iPSC,BIOTIME,GPL10588,MBA_7513,C4ELS5.1 P17 D14 Hystem BMP4 50ng/ml + Rosiglitazone 5uM + T3 2nM last 4 hours CL316243 10uM ,,

iPSC,BIOTIME,GPL10588,MBA_7514,C4ELS5.1 P17 D14 Hystem BMP4 10ng/ml + Rosiglitazone 1uM + T3 2nM last 4 hours CL316243 10uM ,,

iPSC,BIOTIME,GPL10588,MBA_7515,C4ELS5.1 P17 D14 Hystem BMP4 10ng/ml + Rosiglitazone 1uM + T3 2nM last 4 hours CL316243 10uM ,,

iPSC,BIOTIME,GPL10588,MBA_7517,C4ELS5.1 P17 D14 Hystem BMP4 10ng/ml + Rosiglitazone 1uM + T3 2nM + last 2 days FGF21 50ng/ml + last 4 hours CL316243 10uM ,,

iPSC,BIOTIME,GPL10588,MBA_7518,C4ELSR2 P13 D14 Hystem BMP4 50ng/ml + Rosiglitazone 5uM,,

iPSC,BIOTIME,GPL10588,MBA_7519,C4ELSR2 P13 D14 Hystem BMP4 50ng/ml + Rosiglitazone 5uM + T3 2nM + last 4 hours CL316243 10uM ,,

iPSC,BIOTIME,GPL10588,MBA_7520,C4ELSR2 P13 D14 Hystem BMP4 10ng/ml + Rosiglitazone 1uM + T3 2nM + 4 hours CL316243 10uM,,

iPSC,BIOTIME,GPL10588,MBA_7521,C4ELSR2 P13 D14 Hystem BMP4 10ng/ml + Rosiglitazone 1uM + T3 2nM + 4 hours CL316243 10uM,,

iPSC,BIOTIME,GPL10588,MBA_7522,C4ELSR2 P13 D14 Hystem Rosiglitazone 1uM + T3 2nM + 4 hours CL316243 10uM,,

iPSC,BIOTIME,GPL10588,MBA_7523,C4ELSR2 P13 D14 Hystem BMP4 10ng/ml + Rosiglitazone 1uM + T3 2nM + last 2 days FGF21 50ng/ml + last 4 hours CL316243 10uM,,

iPSC,BIOTIME,GPL10588,MBA_7524,C4ELSR2 P13 D14 Hystem BMP4 10ng/ml + Rosiglitazone 1uM + T3 2nM + last 2 days FGF21 50ng/ml + last 4 hours CL316243 10uM,,

iPSC,BIOTIME,GPL10588,MBA_7525,E75 P14 ctrl,,

iPSC,BIOTIME,GPL10588,MBA_7526,E75 P14 D14 Hystem BMP4 10ng/ml,,

iPSC,BIOTIME,GPL10588,MBA_7527,E75 P14 D14 Hystem BMP4 10ng/ml + rosiglitazone 1uM,,

iPSC,BIOTIME,GPL10588,MBA_7528,E75 P14 D14 Hystem rosiglitazone 1uM,,

iPSC,BIOTIME,GPL10588,MBA_7529,E75 P14 D14 Hystem BMP4 10ng/ml + rosiglitazone 1uM + T3 2nM + last 4 hrs CL316243 10uM,,

iPSC,BIOTIME,GPL10588,MBA_7530,E75 P14 D14 Hystem BMP4 10ng/ml + rosiglitazone 1uM + T3 2nM + last 2 days FGF21 50ng/ml + last 4 hrs CL316243 10uM,,

iPSC,BIOTIME,GPL10588,MBA_7647,SK1 P13 D14 Hystem rosiglitazone 1uM,,

iPSC,BIOTIME,GPL10588,MBA_7648,SK1 P13 D14 Hystem BMP4 10ng/ml ,,

iPSC,BIOTIME,GPL10588,MBA_7649,SK1 P13 D14 Hystem BMP4 10ng/ml + rosiglitazone 1uM,,

iPSC,BIOTIME,GPL10588,MBA_7650,SK1 P13 D14 Hystem BMP4 10ng/ml + rosiglitazone 1uM + T3 2nM + last 4 hours CL316243 10uM,,

iPSC,BIOTIME,GPL10588,MBA_7651,SK1 P13 D14 Hystem BMP4 10ng/ml + rosiglitazone 1uM + T3 2nM + last 2 days FGF21 50ng/ml+ last 4 hours CL316243 10uM,,

iPSC,BIOTIME,GPL10588,MBA_7652,SK1 P13 D14 Hystem rosiglitazone 1uM + T3 2nM + last 2 days FGF21 50ng/ml+ last 4 hours CL316243 10uM,,

iPSC,BIOTIME,GPL10588,MBA_7653,SK1 P13 D21 Hystem BMP4 10ng/ml,,

iPSC,BIOTIME,GPL10588,MBA_7654,SK5 P13 D14 Hystem rosiglitazone 1uM,,

iPSC,BIOTIME,GPL10588,MBA_7655,SK5 P13 D14 Hystem BMP4 10ng/ml + rosiglitazone 1uM ,,

iPSC,BIOTIME,GPL10588,MBA_7656,SK5 P13 D14 Hystem BMP4 10ng/ml ,,

iPSC,BIOTIME,GPL10588,MBA_7657,SK5 P13 D14 Hystem BMP4 10ng/ml + rosiglitazone 1uM + T3 2nM + last 4 hours CL316243 10uM,,

iPSC,BIOTIME,GPL10588,MBA_7658,SK5 P13 D14 Hystem BMP4 10ng/ml + rosiglitazone 1uM + T3 2nM + last 2 days FGF21 50ng/ml + last 4 hours CL316243 10uM,,

iPSC,BIOTIME,GPL10588,MBA_7659,SK5 P13 D14 Hystem rosiglitazone 1uM + T3 2nM + last 2 days FGF21 50ng/ml + last 4 hours CL316243 10uM,,

iPSC,BIOTIME,GPL10588,MBA_7660,SK17 P14 D14 Hystem rosiglitazone 1uM,,

iPSC,BIOTIME,GPL10588,MBA_7661,SK17 P14 D14 Hystem BMP4 10ng/ml ,,

iPSC,BIOTIME,GPL10588,MBA_7662,SK17 P14 D14 Hystem BMP4 10ng/ml + rosiglitazone 1uM,,

iPSC,BIOTIME,GPL10588,MBA_7663,SK17 P14 D14 Hystem BMP4 10ng/ml + rosiglitazone 1uM + T3 2ng/ml + last 4 hours CL316243 10uM,,

iPSC,BIOTIME,GPL10588,MBA_7664,SK17 P14 D14 Hystem BMP4 10ng/ml + rosiglitazone 1uM + T3 2ng/ml + last 2 days FGF21 50ng/ml + last 4 hours CL316243 10uM,,

iPSC,BIOTIME,GPL10588,MBA_7665,SK17 P14 D14 Hystem rosiglitazone 1uM + T3 2ng/ml + last 2 days FGF21 50ng/ml + last 4 hours CL316243 10uM,,

iPSC,BIOTIME,GPL10588,MBA_7666,SK17 P14 D21 Hystem BMP4 10ng/ml,,

iPSC,BIOTIME,GPL10588,MBA_7668,ESI 004 NP88 SM P12 D14 Hystem rosiglitazone 1uM,,

iPSC,BIOTIME,GPL10588,MBA_7669,ESI 004 NP88 SM P12 D14 Hystem BMP4 10ng/ml ,,

iPSC,BIOTIME,GPL10588,MBA_7670,ESI 004 NP88 SM P12 D14 Hystem BMP4 10ng/ml + rosiglitazone 1uM,,

iPSC,BIOTIME,GPL10588,MBA_7671,ESI 004 NP88 SM P12 D14 Hystem BMP4 10ng/ml + rosiglitazone 1uM + T3 2nM + last 4 hours CL316243 10uM,,

iPSC,BIOTIME,GPL10588,MBA_7672,ESI 004 NP88 SM P12 D14 Hystem BMP4 10ng/ml + rosiglitazone 1uM + T3 2nM + last 2 days FGF21 50ng/ml + last 4 hours CL316243 10uM,,

iPSC,BIOTIME,GPL10588,MBA_7673,ESI 004 NP88 SM P12 D14 Hystem rosiglitazone 1uM + T3 2nM + last 2 days FGF21 50ng/ml + last 4 hours CL316243 10uM,,

iPSC,BIOTIME,GPL10588,MBA_7674,ESI004 NP88 SM P12 D21 Hystem BMP4 10ng/ml,,

iPSC,BIOTIME,GPL10588,MBA_7676,ESI004 NP111 SM P12 D14 Hystem rosiglitazone 1uM,,

iPSC,BIOTIME,GPL10588,MBA_7677,ESI004 NP111 SM P12 D14 Hystem BMP4 10ng/ml ,,

iPSC,BIOTIME,GPL10588,MBA_7678,ESI004 NP111 SM P12 D14 Hystem BMP4 10ng/ml + rosiglitazone 1uM,,

iPSC,BIOTIME,GPL10588,MBA_7679,ESI004 NP111 SM P12 D14 Hystem BMP4 10ng/ml + rosiglitazone 1uM + T3 2ng/ml + last 4 hours CL316243 10ng/ml,,

iPSC,BIOTIME,GPL10588,MBA_7680,ESI004 NP111 SM P12 D14 Hystem BMP4 10ng/ml + rosiglitazone 1uM + T3 2ng/ml + last 2 days FGF21 50ng/ml + last 4 hours CL316243 10ng/ml,,

iPSC,BIOTIME,GPL10588,MBA_7681,ESI004 NP111 SM P12 D14 Hystem rosiglitazone 1uM + T3 2ng/ml + last 2 days FGF21 50ng/ml + last 4 hours CL316243 10ng/ml,,

iPSC,BIOTIME,GPL10588,MBA_7682,ESI004 NP111 SM P12 D21 Hystem BMP4 10ng/ml,,

iPSC,BIOTIME,GPL10588,MBA_7685,ESI RP002 EN49 P10 D14 Hystem rosiglitazone 1uM,,

iPSC,BIOTIME,GPL10588,MBA_7686,ESI RP002 EN49 P10 D14 Hystem BMP4 10ng/ml ,,

iPSC,BIOTIME,GPL10588,MBA_7687,ESI RP002 EN49 P10 D14 Hystem BMP4 10ng/ml + rosiglitazone 1uM,,

iPSC,BIOTIME,GPL10588,MBA_7688,ESI RP002 EN49 P10 D14 Hystem BMP4 10ng/ml + rosiglitazone 1uM + T3 2nM + last 4 hours CL316243 10uM,,

iPSC,BIOTIME,GPL10588,MBA_7689,ESI RP002 EN49 P10 D14 Hystem BMP4 10ng/ml + rosiglitazone 1uM + T3 2nM + last 2 days FGF21 50ng/ml + last 4 hours CL316243 10uM,,

iPSC,BIOTIME,GPL10588,MBA_7690,ESI RP002 EN49 P10 D14 Hystem rosiglitazone 1uM + T3 2nM + last 2 days FGF21 50ng/ml + last 4 hours CL316243 10uM,,

iPSC,BIOTIME,GPL10588,MBA_7691,ESI RP002 EN49 P10 D14 Hystem BMP4 10ng/ml + rosiglitazone 1uM + T3 2nM + last 4 hours CL316243 10uM,,

iPSC,BIOTIME,GPL10588,MBA_7692,ESI RP002 EN49 P10 D21 Hystem BMP4 10ng/ml,,

iPSC,BIOTIME,GPL10588,MBA_7693,ESI RP002 EN49 P10 D21 Hystem BMP4 10ng/ml,,

iPSC,BIOTIME,GPL10588,MBA_7694,ESI004 NP110 SM P12 D14 Hystem BMP4 10ng/ml + rosiglitazone 1uM + T3 2nM + last 4 hours CL316243 10uM,,

iPSC,BIOTIME,GPL10588,MBA_7695,ESI004 NP110 SM P12 D14 Hystem BMP4 10ng/ml + rosiglitazone 1uM + T3 2nM + last 4 hours CL316243 10uM,,

iPSC,BIOTIME,GPL10588,MBA_7696,ESI004 NP110 SM P12 D14 Hystem BMP4 10ng/ml + rosiglitazone 1uM + T3 2nM + last 2 days FGF21 50ng/ml + last 4 hours CL316243 10uM,,

iPSC,BIOTIME,GPL10588,MBA_7697,ESI004 NP110 SM P12 D14 Hystem BMP4 10ng/ml + rosiglitazone 1uM + T3 2nM + last 2 days FGF21 50ng/ml + last 4 hours CL316243 10uM,,

iPSC,BIOTIME,GPL10588,MBA_7698,ESI004 NP110 SM P12 D14 Hystem BMP4 10ng/ml + rosiglitazone 1uM + T3 2nM + last 4 hours CL316243 10uM at 28C,,

iPSC,BIOTIME,GPL10588,MBA_7699,ESI004 NP110 SM P12 D14 Hystem BMP4 10ng/ml + rosiglitazone 1uM + T3 2nM + last 4 hours CL316243 10uM at 28C,,

iPSC,BIOTIME,GPL10588,MBA_7700,ESI004 NP110 SM P11 D14 Hystem BMP4 50ng/ml,,

iPSC,BIOTIME,GPL10588,MBA_7701,ESI004 NP110 SM P11 D14 Hystem rosiglitazone 5uM ,,

iPSC,BIOTIME,GPL10588,MBA_7702,ESI004 NP110 SM P11 D14 Hystem BMP4 50ng/ml + rosiglitazone 5uM,,

iPSC,BIOTIME,GPL10588,MBA_7703,ESI004 NP110 SM P11 D14 Hystem BMP4 50ng/ml + rosiglitazone 5uM + T3 2nM + last 4 hours CL316243 10uM,,

iPSC,BIOTIME,GPL10588,MBA_7704,ESI004 NP110 SM P11 D14 Hystem BMP4 50ng/ml + rosiglitazone 5uM + T3 2nM + last 2 days FGF21 50ng/ml + last 4 hours CL316243 10uM,,

iPSC,BIOTIME,GPL10588,MBA_7705,ESI004 NP110 SM P11 D21 Hystem BMP4 10ng/ml,,

iPSC,BIOTIME,GPL10588,MBA_7707,C4ELS5.1 P15 D14 Hystem BMP4 10ng/ml + rosiglitazone 1uM + T3 2nM + last 4 hours CL316243 10uM,,

iPSC,BIOTIME,GPL10588,MBA_7708,C4ELS5.1 P15 D14 Hystem BMP4 10ng/ml + rosiglitazone 1uM + T3 2nM + last 4 hours CL316243 10uM,,

iPSC,BIOTIME,GPL10588,MBA_7709,C4ELS5.1 P15 D14 Hystem BMP4 10ng/ml + rosiglitazone 1uM + T3 2nM + last 4 hours CL316243 10uM,,

iPSC,BIOTIME,GPL10588,MBA_7710,C4ELS5.1 P15 D14 Hystem BMP4 10ng/ml + rosiglitazone 1uM + T3 2nM + last 2 days FGF21 50ng/ml + last 4 hours CL316243 10uM,,

iPSC,BIOTIME,GPL10588,MBA_7711,C4ELS5.1 P15 D14 Hystem BMP4 10ng/ml + rosiglitazone 1uM + T3 2nM + last 2 days FGF21 50ng/ml + last 4 hours CL316243 10uM,,

iPSC,BIOTIME,GPL10588,MBA_7712,C4ELS5.1 P15 D14 Hystem BMP4 10ng/ml + rosiglitazone 1uM + T3 2nM + last 4 hours CL316243 10uM at 28C,,

iPSC,BIOTIME,GPL10588,MBA_7713,C4ELS5.1 P15 D14 Hystem BMP4 10ng/ml + rosiglitazone 1uM + T3 2nM + last 4 hours CL316243 10uM at 28C,,

iPSC,BIOTIME,GPL10588,MBA_7714,C4ELSR2 P14 ctrl,,

iPSC,BIOTIME,GPL10588,MBA_7715,C4ELSR2 P14 D14 Hystem BMP4 10ng/ml + rosiglitazone 1uM + T3 2nM + last 4 hours CL316243 10uM,,

iPSC,BIOTIME,GPL10588,MBA_7716,C4ELSR2 P14 D14 Hystem BMP4 10ng/ml + rosiglitazone 1uM + T3 2nM + last 4 hours CL316243 10uM,,

iPSC,BIOTIME,GPL10588,MBA_7717,C4ELSR2 P14 D14 Hystem BMP4 10ng/ml + rosiglitazone 1uM + T3 2nM + last 4 hours CL316243 10uM,,

iPSC,BIOTIME,GPL10588,MBA_7718,C4ELSR2 P14 D14 Hystem BMP4 10ng/ml + rosiglitazone 1uM + T3 2nM + last 2 days FGF21 50ng/ml + last 4 hours CL316243 10uM,,

iPSC,BIOTIME,GPL10588,MBA_7719,C4ELSR2 P14 D14 Hystem BMP4 10ng/ml + rosiglitazone 1uM + T3 2nM + last 2 days FGF21 50ng/ml + last 4 hours CL316243 10uM,,

iPSC,BIOTIME,GPL10588,MBA_7720,C4ELSR2 P14 D14 Hystem BMP4 10ng/ml + rosiglitazone 1uM + T3 2nM + last 2 days FGF21 50ng/ml + last 4 hours CL316243 10uM at 28C,,

iPSC,BIOTIME,GPL10588,MBA_7721,C4ELSR2 P14 D14 Hystem BMP4 10ng/ml + rosiglitazone 1uM + T3 2nM + last 2 days FGF21 50ng/ml + last 4 hours CL316243 10uM at 28C,,

iPSC,BIOTIME,GPL10588,MBA_7723,ESI004 NPCC SM19 P5 D14 Hystem BMP4 10ng/ml + rosiglitazone 1uM + T3 2nM + last 4 hours CL316243 10uM,,

iPSC,BIOTIME,GPL10588,MBA_7724,ESI004 NPCC SM19 P5 D14 Hystem rosiglitazone 1uM + T3 2nM + last 4 hours CL316243 10uM,,

iPSC,BIOTIME,GPL10588,MBA_7725,ESI004 NPCC SM19 P5 D21 Hystem BMP4 10ng/ml ,,

iPSC,BIOTIME,GPL10588,MBA_7727,ESI004 NPCC SM31 P5 D14 Hystem BMP4 10ng/ml + rosiglitazone 1uM + T3 2nM + last 4 hours CL316243 10uM,,

iPSC,BIOTIME,GPL10588,MBA_7728,ESI004 NPCC SM31 P5 D14 Hystem rosiglitazone 1uM + T3 2nM + last 4 hours CL316243 10uM,,

iPSC,BIOTIME,GPL10588,MBA_7729,ESI004 NPCC SM31 P5 D21 Hystem BMP4 10ng/ml,,

iPSC,BIOTIME,GPL10588,MBA_7731,ESI004 NPCC SM36 P5 D14 Hystem BMP4 10ng/ml + rosiglitazone 1uM + T3 2nM + last 4 hours CL316243 10uM,,

iPSC,BIOTIME,GPL10588,MBA_7732,ESI004 NPCC SM36 P5 D14 Hystem rosiglitazone 1uM + T3 2nM + last 4 hours CL316243 10uM,,

iPSC,BIOTIME,GPL10588,MBA_7733,ESI004 NPCC SM36 P5 D21 Hystem BMP4 10ng/ml,,

iPSC,BIOTIME,GPL10588,MBA_7735,ESI004 NPCC SM28 P5 D14 Hystem BMP4 10ng/ml + rosiglitazone 1uM + T3 2nM + last 4 hours CL316243 10uM,,

iPSC,BIOTIME,GPL10588,MBA_7736,ESI004 NPCC SM28 P5 D14 Hystem rosiglitazone 1uM + T3 2nM + last 4 hours CL316243 10uM,,

iPSC,BIOTIME,GPL10588,MBA_7738,ESI004 NPCC SM40 P5 D14 Hystem BMP4 10ng/ml + rosiglitazone 1uM + T3 2nM + last 4 hours CL316243 10uM,,

iPSC,BIOTIME,GPL10588,MBA_7739,ESI004 NPCC SM40 P5 D14 Hystem rosiglitazone 1uM + T3 2nM + last 4 hours CL316243 10uM,,

iPSC,BIOTIME,GPL10588,MBA_7741,CDD4 SK22 P10 D14 Hystem BMP4 10ng/ml + rosiglitazone 1uM + T3 2nM + last 4 hours CL316243 10uM,,

iPSC,BIOTIME,GPL10588,MBA_7742,CDD4 SK22 P10 D14 Hystem rosiglitazone 1uM + T3 2nM + last 4 hours CL316243 10uM,,

iPSC,BIOTIME,GPL10588,MBA_7744,J13 P11 D14 Hystem BMP4 10ng/ml + rosiglitazone 1uM + T3 2nM + last 4 hours CL316243 10uM,,

iPSC,BIOTIME,GPL10588,MBA_7745,J13 P11 D14 Hystem rosiglitazone 1uM + T3 2nM + last 2 days FGF21 50ng/ml + last 4 hours CL316243 10uM,,

iPSC,BIOTIME,GPL10588,MBA_7746,J13 P11 D21 Hystem BMP4 10ng/ml,,

iPSC,BIOTIME,GPL10588,MBA_7747,E75 P14 D14 confluence BMP4 10ng/ml,,

iPSC,BIOTIME,GPL10588,MBA_7748,E75 P14 D14 confluence BMP4 10ng/ml + rosiglitazone 1uM,,

iPSC,BIOTIME,GPL10588,MBA_7749,E75 P14 D14 confluence rosiglitazone 1uM,,

iPSC,BIOTIME,GPL10588,MBA_7920,ABR skin sample 9wk #9579 from lower limb,,

iPSC,BIOTIME,GPL10588,MBA_7921,ABR skin sample 9wk #9578 from upper limb,,

iPSC,BIOTIME,GPL10588,MBA_7922,ABR skin sample 14wk #9580 from back,,

iPSC,BIOTIME,GPL10588,MBA_7923,ABR skin sample 14wk #9580 from back,,

AC,BIOTIME,GPL10588,MBA_7924,Upper limb 10wk #9597,,

AC,BIOTIME,GPL10588,MBA_7925,Upper limb 9wk #9596,,

AC,BIOTIME,GPL10588,MBA_7926,Lower limb 10wk #9598,,

iPSC,BIOTIME,GPL10588,MBA_7928,ABR back skin 14 wk cultured FB P0 1 #9580 ,,

AC,BIOTIME,GPL10588,MBA_7930,9595 P1 Fibroblasts lower limb 9wk,,

iPSC,BIOTIME,GPL10588,MBA_7933,RP1-SM2-21 P10 D14 Hystem BMP4 10ng/ml + rosiglitazone 1uM + T3 2nM + last 4 hours CL316243 10uM,,

iPSC,BIOTIME,GPL10588,MBA_7934,RP1-SM2-21 P10 D14 Hystem rosiglitazone 1uM + T3 2nM + last 4 hours CL316243 10uM,,

iPSC,BIOTIME,GPL10588,MBA_7935,RP1-SM2-21 P10 D21 Hystem BMP4 10ng/ml ,,

iPSC,BIOTIME,GPL10588,MBA_7937,RP1-SKEL-19 P9 D14 Hystem BMP4 10ng/ml + rosiglitazone 1uM + T3 2nM + last 4 hours CL316243 10uM,,

iPSC,BIOTIME,GPL10588,MBA_7938,RP1-SKEL-19 P9 D14 Hystem rosiglitazone 1uM + T3 2nM + last 4 hours CL316243 10uM,,

iPSC,BIOTIME,GPL10588,MBA_7939,RP1-SKEL-19 P9 D14 Hystem rosiglitazone 1uM + T3 2nM + last 2 days FGF21 50ng/ml + last 4 hours CL316243 10uM,,

iPSC,BIOTIME,GPL10588,MBA_7940,RP1-SKEL-19 P9 D21 Hystem BMP4 10ng/ml,,

iPSC,BIOTIME,GPL10588,MBA_7942,RP1-SKEL-20 P10 D14 Hystem BMP4 10ng/ml + rosiglitazone 1uM + T3 2nM + last 4 hours CL316243 10uM,,

iPSC,BIOTIME,GPL10588,MBA_7943,RP1-SKEL-20 P10 D14 Hystem rosiglitazone 1uM + T3 2nM + last 4 hours CL316243 10uM,,

iPSC,BIOTIME,GPL10588,MBA_7944,RP1-SKEL-20 P10 D21 Hystem BMP4 10ng/ml,,

iPSC,BIOTIME,GPL10588,MBA_7946,RP1-SKEL-23 P9 D14 Hystem BMP4 10ng/ml + rosiglitazone 1uM + T3 2nM + last 4 hours CL316243 10uM,,

iPSC,BIOTIME,GPL10588,MBA_7947,RP1-SKEL-23 P9 D14 Hystem rosiglitazone 1uM + T3 2nM + last 4 hours CL316243 10uM,,

iPSC,BIOTIME,GPL10588,MBA_7948,RP1-SKEL-23 P9 D21 Hystem BMP4 10ng/ml,,

iPSC,BIOTIME,GPL10588,MBA_7950,RP1-SKEL-6 P10 D14 Hystem BMP4 10ng/ml + rosiglitazone 1uM + T3 2nM + last 4 hours CL316243 10uM,,

iPSC,BIOTIME,GPL10588,MBA_7951,RP1-SKEL-6 P10 D14 Hystem rosiglitazone 1uM + T3 2nM + last 4 hours CL316243 10uM,,

iPSC,BIOTIME,GPL10588,MBA_7952,RP1-SKEL-6 P10 D21 Hystem BMP4 10ng/ml,,

iPSC,BIOTIME,GPL10588,MBA_7954,RP1-DM10-19 P10 D14 Hystem BMP4 10ng/ml + rosiglitazone 1uM + T3 2nM + last 4 hours CL316243 10uM,,

iPSC,BIOTIME,GPL10588,MBA_7955,RP1-DM10-19 P10 D14 Hystem rosiglitazone 1uM + T3 2nM + last 4 hours CL316243 10uM,,

iPSC,BIOTIME,GPL10588,MBA_7956,RP1-DM10-19 P10 D14 Hystem rosiglitazone 1uM + T3 2nM + last 2 days FGF21 50ng/ml + last 4 hours CL316243 10uM,,

iPSC,BIOTIME,GPL10588,MBA_7957,RP1-DM10-19 P10 D21 Hystem BMP4 10ng/ml,,

iPSC,BIOTIME,GPL10588,MBA_7959,RP1-SKEL-8 P10 D14 Hystem BMP4 10ng/ml + rosiglitazone 1uM + T3 2nM + last 4 hours CL316243 10uM,,

iPSC,BIOTIME,GPL10588,MBA_7960,RP1-SKEL-8 P10 D14 Hystem rosiglitazone 1uM + T3 2nM + last 4 hours CL316243 10uM,,

iPSC,BIOTIME,GPL10588,MBA_7961,RP1-SKEL-8 D21 Hystem BMP4 10ng/ml,,

iPSC,BIOTIME,GPL10588,MBA_7963,RP1-SKEL-16 P10 D14 Hystem BMP4 10ng/ml + rosiglitazone 1uM + T3 2nM + last 4 hours CL316243 10uM,,

iPSC,BIOTIME,GPL10588,MBA_7964,RP1-SKEL-16 P10 D14 Hystem rosiglitazone 1uM + T3 2nM + last 4 hours CL316243 10uM,,

iPSC,BIOTIME,GPL10588,MBA_7965,RP1-SKEL-16 P10 D21 Hystem BMP4 10ng/ml ,,

iPSC,BIOTIME,GPL10588,MBA_7967,RP1-MV2-16 P16 plastic D14 Hystem BMP4 10ng/ml + rosiglitazone 1uM + T3 2nM + last 4 hours CL316243 10uM,,

iPSC,BIOTIME,GPL10588,MBA_7968,RP1-MV2-16 P16 plastic D14 Hystem rosiglitazone 1uM + T3 2nM + last 4 hours CL316243 10uM,,

iPSC,BIOTIME,GPL10588,MBA_7969,RP1-MV2-16 P16 plastic D14 Hystem rosiglitazone 1uM + T3 2nM + last 2 days FGF21 50ng/ml + last 4 hours CL316243 10uM,,

iPSC,BIOTIME,GPL10588,MBA_7971,30-MV2-8 P9 D14 Hystem BMP4 10ng/ml + rosiglitazone 1uM + T3 2nM + last 4 hours CL316243 10uM,,

iPSC,BIOTIME,GPL10588,MBA_7972,30-MV2-8 P9 D14 Hystem BMP4 10ng/ml + rosiglitazone 1uM + T3 2nM + last day FGF21 50ng/ml + last 4 hours CL316243 10uM ,,

iPSC,BIOTIME,GPL10588,MBA_7973,30-MV2-8 P9 D14 Hystem rosiglitazone 1uM + T3 2nM + last day FGF21 50ng/ml + last 4 hours CL316243 10uM,,

iPSC,BIOTIME,GPL10588,MBA_7974,30-MV2-8 P9 D21 Hystem BMP4 10ng/ml,,

iPSC,BIOTIME,GPL10588,MBA_7976,30-MV2-24 P9 D14 Hystem BMP4 10ng/ml + rosiglitazone 1uM + T3 2nM + last 4 hours CL316243 10uM,,

iPSC,BIOTIME,GPL10588,MBA_7977,30-MV2-24 P9 D14 Hystem BMP4 10ng/ml + rosiglitazone 1uM + T3 2nM + last 4 hours CL316243 10uM and FGF21 50ng/ml,,

iPSC,BIOTIME,GPL10588,MBA_7978,30-MV2-24 P9 D14 Hystem rosiglitazone 1uM + T3 2nM + last 4 hours CL316243 10uM and FGF21 50ng/ml,,

iPSC,BIOTIME,GPL10588,MBA_7979,30-MV2-24 P9 D21 Hystem BMP4 10ng/ml ,,

iPSC,BIOTIME,GPL10588,MBA_7980,30-MV2-13 P10 ctrl,,

iPSC,BIOTIME,GPL10588,MBA_7981,30-MV2-13 P10 D14 Hystem BMP4 10ng/ml + rosiglitazone 1uM + T3 2nM + last 4 hours CL316243 10uM,,

iPSC,BIOTIME,GPL10588,MBA_7982,30-MV2-13 P10 D14 Hystem BMP4 10ng/ml + rosiglitazone 1uM + T3 2nM + last 4 hours CL316243 10uM and FGF21 50ng/ml,,

iPSC,BIOTIME,GPL10588,MBA_7983,30-MV2-13 P10 D14 Hystem rosiglitazone 1uM + T3 2nM + last 4 hours CL316243 10uM and FGF21 50ng/ml,,

iPSC,BIOTIME,GPL10588,MBA_7984,30-MV2-13 P10 D21 Hystem BMP4 10ng/ml ,,

iPSC,BIOTIME,GPL10588,MBA_7985,30-MV2-11 P9 ctrl,,

iPSC,BIOTIME,GPL10588,MBA_7986,30-MV2-11 P9 D14 Hystem BMP4 10ng/ml + rosiglitazone 1uM + T3 2nM + last 4 hours CL316243 10uM,,

iPSC,BIOTIME,GPL10588,MBA_7987,30-MV2-11 P9 D14 Hystem BMP4 10ng/ml + rosiglitazone 1uM + T3 2nM + last day FGF21 50ng/ml + last 4 hours CL316243 10uM,,

iPSC,BIOTIME,GPL10588,MBA_7988,30-MV2-11 P9 D14 Hystem rosiglitazone 1uM + T3 2nM + last day FGF21 50ng/ml + last 4 hours CL316243 10uM ,,

iPSC,BIOTIME,GPL10588,MBA_7989,30-MV2-11 P9 D21 Hystem BMP4 10ng/ml,,

iPSC,BIOTIME,GPL10588,MBA_7991,30-SKEL-7 P9 D14 Hystem BMP4 10ng/ml + rosiglitazone 1uM T3 2nM + last 4 hours CL316243 10uM,,

iPSC,BIOTIME,GPL10588,MBA_7992,30-SKEL-7 P9 D14 Hystem BMP4 10ng/ml + rosiglitazone 1uM T3 2nM + last 2 days FGF21 50ng/ml + last 4 hours CL316243 10uM,,

iPSC,BIOTIME,GPL10588,MBA_7993,30-SKEL-7 P9 D14 Hystem rosiglitazone 1uM T3 2nM + last 4 hours CL316243 10uM,,

iPSC,BIOTIME,GPL10588,MBA_7994,30-SKEL-7 P9 D21 Hystem BMP4 10/ml,,

iPSC,BIOTIME,GPL10588,MBA_7996,30-SKEL-4 P9 D14 Hystem BMP4 10ng/ml + rosiglitazone 1uM + T3 2nM + last 4 hours CL316243 10uM,,

iPSC,BIOTIME,GPL10588,MBA_7997,30-SKEL-4 P9 D14 Hystem BMP4 10ng/ml + rosiglitazone 1uM + T3 2nM + last 2 days FGF21 50ng/ml last 4 hours CL316243 10uM,,

iPSC,BIOTIME,GPL10588,MBA_7998,30-SKEL-4 P9 D14 Hystem rosiglitazone 1uM + T3 2nM + last 4 hours CL316243 10uM,,

iPSC,BIOTIME,GPL10588,MBA_8000,ESI004 EP-SK8 P11 D14 Hystem BMP4 10ng/ml + rosiglitazone 1uM + T3 2nM + last 4 hours CL316243 10uM,,

iPSC,BIOTIME,GPL10588,MBA_8001,ESI004 EP-SK8 P11 D14 Hystem rosiglitazone 1uM + T3 2nM + last 4 hours CL316243 10uM,,

iPSC,BIOTIME,GPL10588,MBA_8002,ESI004 EP-SK8 P11 D14 Hystem rosiglitazone 1uM + T3 2nM + last 2 days FGF21 50ng/ml + last 4 hours CL316243 10uM,,

iPSC,BIOTIME,GPL10588,MBA_8003,ESI004 EP SK8 P11 D21 Hystem BMP4 10ng/ml,,

iPSC,BIOTIME,GPL10588,MBA_8005,ESI004 NPCC SM29 P6 D14 Hystem BMP4 10ng/ml + rosiglitazone 1uM + T3 2nM + last 4 hours CL316243 10uM,,

iPSC,BIOTIME,GPL10588,MBA_8006,ESI004 NPCC SM29 P6 D14 Hystem rosiglitazone 1uM + T3 2nM + last 4 hours CL316243 10uM,,

iPSC,BIOTIME,GPL10588,MBA_8007,ESI004 NPCC SM29 P6 D21 Hystem BMP4 10ng/ml,,

iPSC,BIOTIME,GPL10588,MBA_8009,ESI004 NPCC SM46 P8 D14 Hystem BMP4 10ng/ml + rosiglitazone 1uM + T3 2nM + last 4 hours CL316243 10uM,,

iPSC,BIOTIME,GPL10588,MBA_8010,ESI004 NPCC SM46 P8 D14 Hystem rosiglitazone 1uM + T3 2nM + last 4 hours CL316243 10uM,,

iPSC,BIOTIME,GPL10588,MBA_8011,ESI004 NPCC SM46 P8 D21 Hystem BMP4 10ng/ml,,

iPSC,BIOTIME,GPL10588,MBA_8012,ESI004 NPCC SM46 D21 Hystem BMP4 10ng/ml,,

iPSC,BIOTIME,GPL10588,MBA_8014,ESI004 NPCC SM23 P8 D14 Hystem BMP4 10ng/ml + rosiglitazone 1uM + T3 2nM + last 4 hours CL316243 10uM,,

iPSC,BIOTIME,GPL10588,MBA_8015,ESI004 NPCC SM23 P8 D14 Hystem rosiglitazone 1uM + T3 2nM + last 4 hours CL316243 10uM,,

iPSC,BIOTIME,GPL10588,MBA_8017,ESI004 NPCC SM45 P6 D14 Hystem BMP4 10ng/ml + rosiglitazone 1uM + T3 2nM + last 4 hours CL316243 10uM,,

iPSC,BIOTIME,GPL10588,MBA_8018,ESI004 NPCC SM45 P6 D14 Hystem rosiglitazone 1uM + T3 2nM + last 4 hours CL316243 10uM,,

iPSC,BIOTIME,GPL10588,MBA_8020,30-SKEL-6 P8 D14 Hystem BMP4 10ng/ml + rosiglitazone 1uM T3 2nM + last 4 hours CL316243 10uM,,

iPSC,BIOTIME,GPL10588,MBA_8021,30-SKEL-6 P8 D14 Hystem BMP4 10ng/ml + rosiglitazone 1uM T3 2nM + last 2 days FGF21 50ng/ml + last 4 hours CL316243 10uM,,

iPSC,BIOTIME,GPL10588,MBA_8022,30-SKEL-6 P8 D14 Hystem rosiglitazone 1uM T3 2nM + last 4 hours CL316243 10uM,,

iPSC,BIOTIME,GPL10588,MBA_8024,ESI004 NP110 SM P17 D14 Hystem BMP4 10ng/ml + rosiglitazone 1uM + T3 2nM + last 4 hours CL316243 10uM,,

AC,BIOTIME,GPL10588,MBA_8027,Fetal brown preadipocytes (Zenbio) P8 D14 Hystem BMP4 10ng/ml + rosiglitazone 1uM + T3 2nM + last 4 hours CL316243 10uM,,

AC,BIOTIME,GPL10588,MBA_8028,Fetal brown preadipocytes (Zenbio) P8 D14 Hystem BMP4 10ng/ml + rosiglitazone 1uM + T3 2nM + last 4 hours CL316243 10uM,,

AC,BIOTIME,GPL10588,MBA_8029,Fetal brown preadipocytes (Zenbio) P8 D14 Hystem BMP4 10ng/ml + rosiglitazone 1uM + T3 2nM + last 4 hours CL316243 10uM,,

AC,BIOTIME,GPL10588,MBA_8033,Fetal brown preadipocytes (Zenbio) P8 D14 Hystem BMP4 10ng/ml + rosiglitazone 1uM + T3 2nM + 2 days FGF21 50ng/ml + last 4 hours CL316243 10uM,,

AC,BIOTIME,GPL10588,MBA_8034,Fetal brown preadipocytes (Zenbio) P8 D14 Hystem BMP4 10ng/ml + rosiglitazone 1uM + T3 2nM + 2 days FGF21 50ng/ml + last 4 hours CL316243 10uM,,

AC,BIOTIME,GPL10588,MBA_8035,Fetal brown preadipocytes (Zenbio) P8 D14 Hystem rosiglitazone 1uM + T3 2nM + last 4 hours CL316243 10uM at 28C,,

AC,BIOTIME,GPL10588,MBA_8036,Fetal brown preadipocytes (Zenbio) P8 D14 Hystem rosiglitazone 1uM + T3 2nM + last 4 hours CL316243 10uM at 28C,,

iPSC,BIOTIME,GPL10588,MBA_8038,ESI004 NP88 SM P15 D14 Hystem BMP4 10ng/ml + rosiglitazone 1uM + T3 2nM + last 4 hours CL316243 10uM,,

iPSC,BIOTIME,GPL10588,MBA_8039,ESI004 NP88 SM P15 D14 Hystem BMP4 10ng/ml + rosiglitazone 1uM + T3 2nM + last 4 hours CL316243 10uM,,

iPSC,BIOTIME,GPL10588,MBA_8040,ESI004 NP88 SM P15 D14 Hystem BMP4 10ng/ml + rosiglitazone 1uM + T3 2nM + last 4 hours CL316243 10uM,,

iPSC,BIOTIME,GPL10588,MBA_8044,ESI004 NP88 SM P15 D14 Hystem BMP4 10ng/ml + rosiglitazone 1uM + T3 2nM + last 2 days FGF21 50ng/ml + last 4 hours CL316243 10uM,,

iPSC,BIOTIME,GPL10588,MBA_8045,ESI004 NP88 SM P15 D14 Hystem BMP4 10ng/ml + rosiglitazone 1uM + T3 2nM + last 2 days FGF21 50ng/ml + last 4 hours CL316243 10uM,,

iPSC,BIOTIME,GPL10588,MBA_8046,ESI004 NP88 SM P15 D14 Hystem BMP4 10ng/ml + rosiglitazone 1uM + T3 2nM + last 4 hours CL316243 10uM at 28C,,

iPSC,BIOTIME,GPL10588,MBA_8047,ESI004 NP88 SM P15 D14 Hystem BMP4 50ng/ml + rosiglitazone 5uM + T3 2nM + last 4 hours CL316243 10uM at 28C,,

iPSC,BIOTIME,GPL10588,MBA_8048,ESI004 NPCC SM28 P6 D12 Hystem BMP4 10ng/ml,,

iPSC,BIOTIME,GPL10588,MBA_8049,ESI004 NPCC SM40 P5 D21 Hystem BMP4 10ng/ml,,

iPSC,BIOTIME,GPL10588,MBA_8050,ESI004 NPCC SM23 P5 D21 Hystem BMP4 10ng/ml,,

iPSC,BIOTIME,GPL10588,MBA_8051,ESI004 NPCC SM27 P5 ctrl,,

iPSC,BIOTIME,GPL10588,MBA_8053,ESI004 NP88 SM P12 D4 Hystem BMP4 10ng/ml + rosiglitazone 1uM + T3 2nM + last 4 hours CL316243 10uM time course,,

iPSC,BIOTIME,GPL10588,MBA_8054,ESI004 NP88 SM P12 D4 Hystem rosiglitazone 1uM + T3 2nM + last 4 hours CL316243 10uM time course,,

iPSC,BIOTIME,GPL10588,MBA_8055,ESI004 NP88 SM P12 D7 Hystem BMP4 10ng/ml + rosiglitazone 1uM + T3 2nM + last 4 hours CL316243 10uM,,

iPSC,BIOTIME,GPL10588,MBA_8056,ESI004 NP88 SM P12 D7 Hystem rosiglitazone 1uM + T3 2nM + last 4 hours CL316243 10uM,,

iPSC,BIOTIME,GPL10588,MBA_8057,ESI004 NP88 SM P12 D10 Hystem BMP4 10ng/ml + rosiglitazone 1uM + T3 2nM + last 4 hours CL316243 10uM,,

iPSC,BIOTIME,GPL10588,MBA_8058,ESI004 NP88 SM P12 D10 Hystem rosiglitazone 1uM + T3 2nM + last 4 hours CL316243 10uM,,

iPSC,BIOTIME,GPL10588,MBA_8059,ESI004 NP88 SM P12 D14 Hystem BMP4 10ng/ml + rosiglitazone 1uM + T3 2nM + last 4 hours CL316243 10uM,,

iPSC,BIOTIME,GPL10588,MBA_8061,ESI004 NPCC SM19 P8 ctrl,,

iPSC,BIOTIME,GPL10588,MBA_8062,ESI004 NPCC SM19 P8 D4 Hystem BMP4 10ng/ml + rosiglitazone 1uM + T3 2nM + last 4 hours CL316243 10uM,,

iPSC,BIOTIME,GPL10588,MBA_8063,ESI004 NPCC SM19 P8 D4 Hystem rosiglitazone 1uM + T3 2nM + last 4 hours CL316243 10uM,,

iPSC,BIOTIME,GPL10588,MBA_8064,ESI004 NPCC SM19 P8 D7 Hystem BMP4 10ng/ml + rosiglitazone 1uM + T3 2nM + last 4 hours CL316243 10uM,,

iPSC,BIOTIME,GPL10588,MBA_8065,ESI004 NPCC SM19 P8 D7 Hystem rosiglitazone 1uM + T3 2nM + last 4 hours CL316243 10uM,,

iPSC,BIOTIME,GPL10588,MBA_8066,ESI004 NPCC SM19 P8 D10 Hystem BMP4 10ng/ml + rosiglitazone 1uM + T3 2nM + last 4 hours CL316243 10uM,,

iPSC,BIOTIME,GPL10588,MBA_8067,ESI004 NPCC SM19 P8 D10 Hystem rosiglitazone 1uM + T3 2nM + last 4 hours CL316243 10uM,,

iPSC,BIOTIME,GPL10588,MBA_8068,ESI004 NPCC SM19 P8 D14 Hystem BMP4 10ng/ml + rosiglitazone 1uM + T3 2nM + last 4 hours CL316243 10uM,,

iPSC,BIOTIME,GPL10588,MBA_8069,ESI004 NPCC SM19 P8 D14 Hystem rosiglitazone 1uM + T3 2nM + last 4 hours CL316243 10uM,,

iPSC,BIOTIME,GPL10588,MBA_8071,ESI004 NP110 SM P12 D4 Hystem BMP4 10ng/ml + rosiglitazone 1uM + T3 2nM + last 4 hours CL316243 10uM,,

iPSC,BIOTIME,GPL10588,MBA_8072,ESI004 NP110 SM P12 D4 Hystem rosiglitazone 1uM + T3 2nM + last 4 hours CL316243 10uM,,

iPSC,BIOTIME,GPL10588,MBA_8073,ESI004 NP110 SM P12 D7 Hystem BMP4 10ng/ml + rosiglitazone 1uM + T3 2nM + last 4 hours CL316243 10uM,,

iPSC,BIOTIME,GPL10588,MBA_8074,ESI004 NP110 SM P12 D7 Hystem rosiglitazone 1uM + T3 2nM + last 4 hours CL316243 10uM,,

iPSC,BIOTIME,GPL10588,MBA_8075,ESI004 NP110 SM P12 D10 Hystem BMP4 10ng/ml + rosiglitazone 1uM + T3 2nM + last 4 hours CL316243 10uM,,

iPSC,BIOTIME,GPL10588,MBA_8076,ESI004 NP110 SM P12 D10 Hystem rosiglitazone 1uM + T3 2nM + last 4 hours CL316243 10uM,,

iPSC,BIOTIME,GPL10588,MBA_8077,ESI004 NP110 SM P12 D14 Hystem BMP4 10ng/ml + rosiglitazone 1uM + T3 2nM + last 4 hours CL316243 10uM,,

iPSC,BIOTIME,GPL10588,MBA_8079,ESI004 NP110 SM P12 D14 Hystem BMP7 100ng/ml + rosiglitazone 1uM + T3 2nM + last 4 hours CL316243 10uM,,

iPSC,BIOTIME,GPL10588,MBA_8080,ESI004 NP110 SM P12 D14 confluence BMP4 10ng/ml + rosiglitazone 1uM + T3 2nM + last 4 hours CL316243 10uM,,

iPSC,BIOTIME,GPL10588,MBA_8081,ESI004 NP110 SM P12 D14 confluence rosiglitazone 1uM + T3 2nM + last 4 hours CL316243 10uM,,

iPSC,BIOTIME,GPL10588,MBA_8082,ESI004 NP110 SM P12 D14 confluence BMP4 10ng/ml + rosiglitazone 1uM + T3 2nM Day9 remove BMP4 + last 4 hours CL316243 10uM,,

iPSC,BIOTIME,GPL10588,MBA_8083,ESI004 NP110 SM P12 D14 confluence rosiglitazone 1uM + T3 2nM + Day9 add BMP4 + last 4 hours CL316243 10uM ,,

iPSC,BIOTIME,GPL10588,MBA_8084,ESI004 NP110 SM P27 D14 Hystem BMP4 10ng/ml + rosiglitazone 1uM + T3 2nM + last 4 hours CL316243 10uM,,

iPSC,BIOTIME,GPL10588,MBA_8085,ESI004 NP110 SM P27 D14 Hystem rosiglitazone 1uM + T3 2nM + last 4 hours CL316243 10uM,,

iPSC,BIOTIME,GPL10588,MBA_8086,ESI004 NP110 SM P17 D14 Hystem BMP4 10ng/ml + rosiglitazone 1uM + T3 2nM + last 4 hours CL316243 10uM,,

iPSC,BIOTIME,GPL10588,MBA_8087,ESI004 NP110 SM P17 D14 Hystem rosiglitazone 1uM + T3 2nM + last 4 hours CL316243 10uM,,

iPSC,BIOTIME,GPL10588,MBA_8088,ESI004 NP110 SM P17 D14 Hystem rosiglitazone 1uM + T3 2nM + last 4 hours CL316243 10uM,,

iPSC,BIOTIME,GPL10588,MBA_8089,ESI004 NP110 SM P17 D14 Hystem rosiglitazone 1uM + T3 2nM + last 4 hours CL316243 10uM,,

AC,BIOTIME,GPL10588,MBA_8091,Subcutaneous preadipocytes (Zenbio) P6 D14 Hystem BMP4 10ng/ml + rosiglitazone 1uM + T3 2nM + last 4 hours CL316243 10uM,,

AC,BIOTIME,GPL10588,MBA_8092,Subcutaneous preadipocytes (Zenbio) P6 D14 Hystem BMP4 10ng/ml + rosiglitazone 1uM + T3 2nM + last 4 hours CL316243 10uM,,

AC,BIOTIME,GPL10588,MBA_8093,Subcutaneous preadipocytes (Zenbio) P6 D14 Hystem BMP4 10ng/ml + rosiglitazone 1uM + T3 2nM + last 4 hours CL316243 10uM,,

AC,BIOTIME,GPL10588,MBA_8097,Subcutaneous preadipocytes (Zenbio) P6 D14 Hystem BMP4 10ng/ml + rosiglitazone 1uM + T3 2nM + 2 days FGF21 50ng/ml + last 4 hours CL316243 10uM,,

AC,BIOTIME,GPL10588,MBA_8098,Subcutaneous preadipocytes (Zenbio) P6 D14 Hystem BMP4 10ng/ml + rosiglitazone 1uM + T3 2nM + 2 days FGF21 50ng/ml + last 4 hours CL316243 10uM,,

AC,BIOTIME,GPL10588,MBA_8099,Subcutaneous preadipocytes (Zenbio) P6 D14 Hystem BMP4 10ng/ml + rosiglitazone 1uM + T3 2nM + last 4 hours CL316243 10uM at 28C,,

AC,BIOTIME,GPL10588,MBA_8100,Subcutaneous preadipocytes (Zenbio) P6 D14 Hystem BMP4 10ng/ml + rosiglitazone 1uM + T3 2nM + last 4 hours CL316243 10uM at 28C,,

iPSC,BIOTIME,GPL10588,MBA_8102,E3 P13 D14 Hystem BMP4 10ng/ml + rosiglitazone 1uM + T3 2nM + last 4 hours CL316243 10uM,,

iPSC,BIOTIME,GPL10588,MBA_8103,E3 P13 D14 Hystem BMP4 10ng/ml + rosiglitazone 1uM + T3 2nM + last 4 hours CL316243 10uM,,

iPSC,BIOTIME,GPL10588,MBA_8104,E3 P13 D14 Hystem BMP4 10ng/ml + rosiglitazone 1uM + T3 2nM + last 4 hours CL316243 10uM,,

iPSC,BIOTIME,GPL10588,MBA_8108,E3 P13 D14 Hystem BMP4 10ng/ml + rosiglitazone 1uM + T3 2nM + last 2 days FGF21 50ng/ml + last 4 hours CL316243 10uM,,

iPSC,BIOTIME,GPL10588,MBA_8109,E3 P13 D14 Hystem BMP4 10ng/ml + rosiglitazone 1uM + T3 2nM + last 2 days FGF21 50ng/ml + last 4 hours CL316243 10uM,,

iPSC,BIOTIME,GPL10588,MBA_8110,E3 P13 D14 Hystem BMP4 10ng/ml + rosiglitazone 1uM + T3 2nM + last 4 hours CL316243 10uM at 28C,,

iPSC,BIOTIME,GPL10588,MBA_8111,E3 P13 D14 Hystem BMP4 10ng/ml + rosiglitazone 1uM + T3 2nM + last 4 hours CL316243 10uM at 28C,,

iPSC,BIOTIME,GPL10588,MBA_8113,E85 P14 D14 Hystem BMP4 10ng/ml + rosiglitazone 1uM + T3 2nM + last 4 hours CL316243 10uM,,

iPSC,BIOTIME,GPL10588,MBA_8114,E85 P14 D14 Hystem BMP4 10ng/ml + rosiglitazone 1uM + T3 2nM + last 4 hours CL316243 10uM,,

iPSC,BIOTIME,GPL10588,MBA_8115,E85 P14 D14 Hystem BMP4 10ng/ml + rosiglitazone 1uM + T3 2nM + last 4 hours CL316243 10uM,,

iPSC,BIOTIME,GPL10588,MBA_8119,E85 P14 D14 Hystem BMP4 10ng/ml + rosiglitazone 1uM + T3 2nM + last 2 days FGF21 50ng/ml + last 4 hours CL316243 10uM,,

iPSC,BIOTIME,GPL10588,MBA_8120,E85 P14 D14 Hystem BMP4 10ng/ml + rosiglitazone 1uM + T3 2nM + last 2 days FGF21 50ng/ml + last 4 hours CL316243 10uM,,

iPSC,BIOTIME,GPL10588,MBA_8121,E85 P14 D14 Hystem BMP4 10ng/ml + rosiglitazone 1uM + T3 2nM + last 4 hours CL316243 10uM at 28C,,

iPSC,BIOTIME,GPL10588,MBA_8122,E85 P14 D14 Hystem BMP4 10ng/ml + rosiglitazone 1uM + T3 2nM + last 4 hours CL316243 10uM at 28C,,

iPSC,BIOTIME,GPL10588,MBA_8124,SK1 P15 D14 Hystem BMP4 10ng/ml + rosiglitazone 1uM + T3 2nM + last 4 hours CL316243 10uM,,

iPSC,BIOTIME,GPL10588,MBA_8125,SK1 P15 D14 Hystem rosiglitazone 1uM + T3 2nM + last 4 hours CL316243 10uM,,

iPSC,BIOTIME,GPL10588,MBA_8126,SK1 P15 D14 Hystem BMP4 10ng/ml + rosiglitazone 1uM + T3 2nM + last day CL316243 10uM,,

iPSC,BIOTIME,GPL10588,MBA_8127,SK1 P15 D14 Hystem rosiglitazone 1uM + T3 2nM + last day CL316243 10uM,,

iPSC,BIOTIME,GPL10588,MBA_8128,SK1 P15 D14 Hystem rosiglitazone 1uM + T3 2nM + last 3 days CL316243 10uM,,

iPSC,BIOTIME,GPL10588,MBA_8129,SK1 P15 D14 confluence BMP4 10ng/ml + rosiglitazone 1uM + T3 2nM + last 4hours CL316243 10uM,,

iPSC,BIOTIME,GPL10588,MBA_8130,SK1 P15 D14 confluence rosiglitazone 1uM + T3 2nM + last day CL316243 10uM,,

iPSC,BIOTIME,GPL10588,MBA_8132,SK5 P14 D14 Hystem BMP4 10ng/ml + rosiglitazone 1uM + T3 2nM + last 4 hours CL316243 10uM,,

iPSC,BIOTIME,GPL10588,MBA_8133,SK5 P14 D14 Hystem rosiglitazone 1uM + T3 2nM + last 4 hours CL316243 10uM,,

iPSC,BIOTIME,GPL10588,MBA_8134,SK5 P14 D14 Hystem BMP4 10ng/ml + rosiglitazone 1uM + T3 2nM + last day CL316243 10uM,,

iPSC,BIOTIME,GPL10588,MBA_8135,SK5 P14 D14 Hystem rosiglitazone 1uM + T3 2nM + last day CL316243 10uM,,

iPSC,BIOTIME,GPL10588,MBA_8136,SK5 P14 D14 Hystem rosiglitazone 1uM + T3 2nM + last 2 days CL316243 10uM,,

iPSC,BIOTIME,GPL10588,MBA_8138,SK11 P16 D14 Hystem BMP4 10ng/ml + rosiglitazone 1uM + T3 2nM + last 4 hours CL316243 10uM,,

iPSC,BIOTIME,GPL10588,MBA_8139,SK11 P16 D14 Hystem rosiglitazone 1uM + T3 2nM + last 4 hours CL316243 10uM,,

iPSC,BIOTIME,GPL10588,MBA_8140,SK11 P16 D14 Hystem BMP4 10ng/ml + rosiglitazone 1uM + T3 2nM + last day CL316243 10uM,,

iPSC,BIOTIME,GPL10588,MBA_8141,SK11 P16 D14 Hystem rosiglitazone 1uM + T3 2nM + last day CL316243 10uM,,

iPSC,BIOTIME,GPL10588,MBA_8142,SK11 P15 D14 Hystem rosiglitazone 1uM + T3 2nM + last 3 days CL316243 10uM,,

iPSC,BIOTIME,GPL10588,MBA_8143,SK11 P16 D14 confluence BMP4 10ng/ml + rosiglitazone 1uM + T3 2nM + last day CL316243 10uM,,

iPSC,BIOTIME,GPL10588,MBA_8144,SK11 P16 D14 confluence rosiglitazone 1uM + T3 2nM + last 4 hours CL316243 10uM,,

AC,BIOTIME,GPL10588,MBA_8206,HS-1 P6 D14 Hystem incomplete (basal) only,,

AC,BIOTIME,GPL10588,MBA_8207,HS-1 P6 D14 Hystem BMP4 10ng/ml,,

AC,BIOTIME,GPL10588,MBA_8208,HS-1 P6 D14 Hystem rosiglitazone 1uM + T3 2nM + last 4 hours CL316243 10uM,,

AC,BIOTIME,GPL10588,MBA_8209,HS-1 P6 D21 Hystem BMP4 10ng/ml,,

AC,BIOTIME,GPL10588,MBA_8211,FB ABR #9578 Upper limb 9wk P5 D14 Hystem incomplete (basal) only,,

AC,BIOTIME,GPL10588,MBA_8212,FB ABR #9578 Upper limb 9wk P5 D14 Hystem BMP4 10ng/ml,,

AC,BIOTIME,GPL10588,MBA_8213,FB ABR #9578 Upper limb 9wk P5 D14 Hystem rosiglitazone 1uM + T3 2nM + last 4 hours CL316243 10uM,,

AC,BIOTIME,GPL10588,MBA_8214,FB ABR #9578 upper limb 9wk P5 D21 Hystem BMP4 10ng/ml,,

AC,BIOTIME,GPL10588,MBA_8216,MDW-1 P6 D14 Hystem incomplete (basal) only,,

AC,BIOTIME,GPL10588,MBA_8217,MDW-1 P6 D14 Hystem BMP4 10ng/ml,,

AC,BIOTIME,GPL10588,MBA_8218,MDW-1 P6 D14 Hystem rosiglitazone 1uM + T3 2nM + last 4 hours CL316243 10uM,,

AC,BIOTIME,GPL10588,MBA_8219,MDW-1 P6 D21 Hystem BMP4 10ng/ml,,

iPSC,BIOTIME,GPL10588,MBA_8220,RP1-MV2-16 P16 plastic D21 Hystem BMP4 10ng/ml,,

iPSC,BIOTIME,GPL10588,MBA_8221,SK5 D14 confluence BMP4 10ng/ml + rosiglitazone 1uM + T3 2nM + last 4 hours CL316243 10uM,,

iPSC,BIOTIME,GPL10588,MBA_8222,SK5 D14 confluence rosiglitazone 1uM + T3 2nM + last 4 hours CL316243 10uM,,

iPSC,BIOTIME,GPL10588,MBA_8224,ESI004 NP110 SM P15 D14 confluent BMP7 100ng/ml + rosiglitazone 1uM + T3 2nM + last 4 hours CL316243 10uM,,

iPSC,BIOTIME,GPL10588,MBA_8225,ESI004 NP110 SM P15 D14 confluent BMP7 100ng/ml + rosiglitazone 1uM + T3 2nM + last 4 hours CL316243 10uM,,

iPSC,BIOTIME,GPL10588,MBA_8226,ESI004 NP110 SM P15 D14 confluent BMP4 10ng/ml + rosiglitazone 1uM + T3 2nM + last 4 hours CL316243 10uM,,

iPSC,BIOTIME,GPL10588,MBA_8227,ESI004 NP110 SM P15 D14 confluent BMP4 10ng/ml + rosiglitazone 1uM + T3 2nM + last 4 hours CL316243 10uM,,

iPSC,BIOTIME,GPL10588,MBA_8228,ESI004 NP110 SM P15 D14 confluent rosiglitazone 1uM + T3 2nM + last 4 hours CL316243 10uM,,

iPSC,BIOTIME,GPL10588,MBA_8229,ESI004 NP110 SM P15 D14 confluent rosiglitazone 1uM + T3 2nM + last 4 hours CL316243 10uM,,

iPSC,BIOTIME,GPL10588,MBA_8231,ESI004 NP110 SM P21 D14 confluence BMP4 10ng/ml + rosiglitazone 1uM + T3 2nM + last 4 hours CL316243 10uM,,

iPSC,BIOTIME,GPL10588,MBA_8232,ESI004 NP110 SM P21 D14 confluence BMP4 10ng/ml + rosiglitazone 1uM + T3 2nM + last 4 hours CL316243 10uM,,

iPSC,BIOTIME,GPL10588,MBA_8233,ESI004 NP110 SM P20 D14 confluence BMP4 10ng/ml + rosiglitazone 1uM + T3 2nM + last 4 hours CL316243 10uM,,

iPSC,BIOTIME,GPL10588,MBA_8234,ESI004 NP110 SM P20 D14 confluence rosiglitazone 1uM + T3 2nM + last 4 hours CL316243 10uM,,

iPSC,BIOTIME,GPL10588,MBA_8236,ESI004 NP110 SM P32 D14 Hystem BMP4 10ng/ml + rosiglitazone 1uM + T3 2nM + last 4 hours CL316243 10uM,,

iPSC,BIOTIME,GPL10588,MBA_8238,ESI004 NP110 SM P32 D21 Hystem BMP4 10ng/ml + rosiglitazone 1uM + T3 2nM + last 4 hours CL316243 10uM,,

iPSC,BIOTIME,GPL10588,MBA_8241,ESI004 NP88 SM P14 D14 Hystem BMP4 10ng/ml + rosiglitazone 1uM + T3 2nM + last 4 hours CL316243 10uM,,

iPSC,BIOTIME,GPL10588,MBA_8242,ESI004 NP88 SM P14 D14 Hystem BMP4 10ng/ml + rosiglitazone 1uM + T3 2nM + last 4 hours CL316243 10uM,,

iPSC,BIOTIME,GPL10588,MBA_8243,ESI004 NP88 SM P15 D14 confluence BMP4 10ng/ml + rosiglitazone 1uM + T3 2nM + last 4 hours CL316243 10uM,,

iPSC,BIOTIME,GPL10588,MBA_8244,ESI004 NP88 SM P15 D14 confluence rosiglitazone 1uM + T3 2nM + last 4 hours CL316243 10uM,,

iPSC,BIOTIME,GPL10588,MBA_8249,E3 P12 D14 confluence BMP4 10ng/ml + rosiglitazone 1uM + T3 2nM + last 4 hours CL316243 10uM ,,

iPSC,BIOTIME,GPL10588,MBA_8250,E3 P12 D14 confluence BMP4 10ng/ml + rosiglitazone 1uM + T3 2nM + last 4 hours CL316243 10uM ,,

iPSC,BIOTIME,GPL10588,MBA_8252,4D20.9 P15 D14 Hystem BMP4 10ng/ml + rosiglitazone 1uM + T3 2nM + last 4 hours CL316243 10uM,,

iPSC,BIOTIME,GPL10588,MBA_8253,4D20.9 P15 D14 Hystem BMP4 10ng/ml + rosiglitazone 1uM + T3 2nM + last 4 hours CL316243 10uM,,

iPSC,BIOTIME,GPL10588,MBA_8254,4D20.9 P15 D14 Hystem BMP4 10ng/ml + rosiglitazone 1uM + T3 2nM + last 4 hours CL316243 10uM,,

iPSC,BIOTIME,GPL10588,MBA_8255,4D20.9 P15 D14 Hystem rosiglitazone 1uM + T3 2nM + last 4 hours CL316243 10uM,,

iPSC,BIOTIME,GPL10588,MBA_8256,4D20.9 P15 D14 Hystem rosiglitazone 1uM + T3 2nM + last 4 hours CL316243 10uM,,

iPSC,BIOTIME,GPL10588,MBA_8257,4D20.9 P15 D14 Hystem rosiglitazone 1uM + T3 2nM + last 4 hours CL316243 10uM,,

iPSC,BIOTIME,GPL10588,MBA_8258,4D20.9 P15 D14 Hystem rosiglitazone 1uM + T3 2nM + last 4 hours CL316243 10uM at 28C,,

iPSC,BIOTIME,GPL10588,MBA_8259,4D20.9 P15 D14 Hystem rosiglitazone 1uM + T3 2nM + last 4 hours CL316243 10uM at 28C,,

iPSC,BIOTIME,GPL10588,MBA_8260,4D209 P14 D14 Hystem BMP4 10ng/ml + rosiglitazone 1uM + T3 2nM + last 2 days FGF21 50ng/ml + last 4 hours CL316243 10uM,,

iPSC,BIOTIME,GPL10588,MBA_8261,4D209 P14 D14 Hystem BMP4 10ng/ml + rosiglitazone 1uM + T3 2nM + last 2 days FGF21 50ng/ml + last 4 hours CL316243 10uM,,

AC,BIOTIME,GPL10588,MBA_8263,Fetal brown preadipocytes (Zenbio) P6 D14 confluence BMP7 100ng/ml + rosiglitazone 1uM + T3 2nM + last 4 hours CL316243 10uM,,

AC,BIOTIME,GPL10588,MBA_8264,Fetal brown preadipocytes (Zenbio) P6 D14 confluence BMP7 100ng/ml + rosiglitazone 1uM + T3 2nM + last 4 hours CL316243 10uM,,

AC,BIOTIME,GPL10588,MBA_8265,Fetal brown preadipocytes (Zenbio) P6 D14 confluence BMP4 10ng/ml + rosiglitazone 1uM + T3 2nM + last 4 hours CL316243 10uM,,

AC,BIOTIME,GPL10588,MBA_8266,Fetal brown preadipocytes (Zenbio) P6 D14 confluence BMP4 10ng/ml + rosiglitazone 1uM + T3 2nM + last 4 hours CL316243 10uM,,

AC,BIOTIME,GPL10588,MBA_8267,Fetal brown preadipocytes (Zenbio) P6 D14 confluence rosiglitazone 1uM + T3 2nM + last 4 hours CL316243 10uM,,

AC,BIOTIME,GPL10588,MBA_8268,Fetal brown preadipocytes (Zenbio) P6 D14 confluence rosiglitazone 1uM + T3 2nM + last 4 hours CL316243 10uM,,

AC,BIOTIME,GPL10588,MBA_8269,Fetal brown preadipocytes P7 ctrl,,

AC,BIOTIME,GPL10588,MBA_8270,Fetal brown preadipocytes (Zenbio) P7 D14 confluence BMP4 10ng/ml + rosiglitazone 1uM + T3 2nM + last 4 hours CL316243 10uM,,

AC,BIOTIME,GPL10588,MBA_8271,Fetal brown preadipocytes (Zenbio) P7 D14 confluence BMP4 10ng/ml + rosiglitazone 1uM + T3 2nM + last 4 hours CL316243 10uM,,

AC,BIOTIME,GPL10588,MBA_8273,Subcutaneous preadipocytes (Zenbio) P5 D14 confluence BMP7 100ng/ml + rosiglitazone 1uM + T3 2nM + last 4 hours CL316243 10uM,,

AC,BIOTIME,GPL10588,MBA_8274,Subcutaneous preadipocytes (Zenbio) P5 D14 confluence BMP7 100ng/ml + rosiglitazone 1uM + T3 2nM + last 4 hours CL316243 10uM,,

AC,BIOTIME,GPL10588,MBA_8275,Subcutaneous preadipocytes (Zenbio) P5 D14 confluence BMP4 10ng/ml + rosiglitazone 1uM + T3 2nM + last 4 hours CL316243 10uM,,

AC,BIOTIME,GPL10588,MBA_8276,Subcutaneous preadipocytes (Zenbio) P5 D14 confluence BMP4 10ng/ml + rosiglitazone 1uM + T3 2nM + last 4 hours CL316243 10uM,,

AC,BIOTIME,GPL10588,MBA_8277,Subcutaneous preadipocytes (Zenbio) P5 D14 confluence rosiglitazone 1uM + T3 2nM + last 4 hours CL316243 10uM,,

AC,BIOTIME,GPL10588,MBA_8278,Subcutaneous preadipocytes (Zenbio) P5 D14 confluence rosiglitazone 1uM + T3 2nM + last 4 hours CL316243 10uM,,

iPSC,BIOTIME,GPL10588,MBA_8279,E85 P17 D14 confluence BMP4 10ng/ml + rosiglitazone 1uM + T3 2nM + last 4 hours CL316243 10uM,,

iPSC,BIOTIME,GPL10588,MBA_8280,E85 P17 D14 confluence rosiglitazone 1uM + T3 2nM + last 4 hours CL316243 10uM,,

iPSC,BIOTIME,GPL10588,MBA_8365,E3 P14 D14 confluence BMP4 10ng/ml + rosiglitazone 1uM + T3 2nM + last 4 hours CL316243 10uM ,,

iPSC,BIOTIME,GPL10588,MBA_8366,E3 P14 D14 confluence rosiglitazone 1uM + T3 2nM + last 4 hours CL316243 10uM ,,

iPSC,BIOTIME,GPL10588,MBA_8367,C4ELSR2 P17 D14 Hystem BMP4 10ng/ml + rosiglitazone 1uM + T3 2nM + last 4 hours CL316243 10uM at 28C,,

iPSC,BIOTIME,GPL10588,MBA_8368,C4ELSR2 P17 D14 Hystem BMP4 10ng/ml + rosiglitazone 1uM + T3 2nM + last 4 hours CL316243 10uM at 28C,,

iPSC,BIOTIME,GPL10588,MBA_8369,C4ELSR2 P17 D14 Hystem rosiglitazone 1uM + T3 2nM + last 4 hours CL316243 10uM,,

iPSC,BIOTIME,GPL10588,MBA_8370,C4ELSR2 P17 D14 Hystem rosiglitazone 1uM + T3 2nM + last 4 hours CL316243 10uM,,

iPSC,BIOTIME,GPL10588,MBA_8371,C4ELSR2 P17 D14 confluence BMP4 10ng/ml + rosiglitazone 1uM + T3 2nM + last 4 hours CL316243 10uM ,,

iPSC,BIOTIME,GPL10588,MBA_8372,C4ELSR2 P17 D14 confluence rosiglitazone 1uM + T3 2nM + last 4 hours CL316243 10uM ,,

iPSC,BIOTIME,GPL10588,MBA_8374,C4ELS5.1 P17 D14 confluence BMP4 10ng/ml rosiglitazone 1uM + T3 2nM + last 4 hours CL316243 10uM,,

iPSC,BIOTIME,GPL10588,MBA_8375,C4ELS5.1 P17 D14 confluence rosiglitazone 1uM + T3 2nM + last 4 hours CL316243 10uM,,

iPSC,BIOTIME,GPL10588,MBA_8376,ESI004 NP110 SM P9 D14 confluence BMP4 10ng/ml + rosiglitazone 1uM + T3 2nM + last 4 hours CL316243 10uM,,

iPSC,BIOTIME,GPL10588,MBA_8377,ESI004 NP110 SM P9 D14 confluence rosiglitazone 1uM + T3 2nM + last 4 hours CL316243 10uM,,

iPSC,BIOTIME,GPL10588,MBA_8378,ESI004 NP110 SM P11 D14 confluence BMP4 10ng/ml + rosiglitazone 1uM + T3 2nM + last 4 hours CL316243 10uM,,

iPSC,BIOTIME,GPL10588,MBA_8379,ESI004 NP110 SM P11 D14 confluence rosiglitazone 1uM + T3 2nM + last 4 hours CL316243 10uM,,

AC,BIOTIME,GPL10588,MBA_8380,Fetal brown preadipocytes (Zenbio) P6 D14 confluence BMP4 10ng/ml + rosiglitazone 1uM + T3 2nM + last 4 hours CL316243 10uM,,

AC,BIOTIME,GPL10588,MBA_8381,Fetal brown preadipocytes (Zenbio) P6 D14 confluence rosiglitazone 1uM + T3 2nM + last 4 hours CL316243 10uM,,

AC,BIOTIME,GPL10588,MBA_8382,Fetal brown preadipocytes (Zenbio) P5 D14 confluence BMP4 10ng/ml + rosiglitazone 1uM + T3 2nM + last 4 hours CL316243 10uM,,

AC,BIOTIME,GPL10588,MBA_8383,Fetal brown preadipocytes (Zenbio) P5 D14 confluence rosiglitazone 1uM + T3 2nM + last 4 hours CL316243 10uM,,

AC,BIOTIME,GPL10588,MBA_8384,Subcutaneous preadipocytes (Zenbio) P7 ctrl,,

AC,BIOTIME,GPL10588,MBA_8385,Subcutaneous preadipocytes (Zenbio) P7 D14 confluence BMP4 10ng/ml + rosiglitazone 1uM + T3 2nM + last 4 hours CL316243 10uM,,

AC,BIOTIME,GPL10588,MBA_8386,Subcutaneous preadipocytes (Zenbio) P7 D14 confluence rosiglitazone 1uM + T3 2nM + last 4 hours CL316243 10uM,,

AC,BIOTIME,GPL10588,MBA_8389,CASMC P6 (Lonza lot 0000200212) D14 Hystem BMP4 10ng/ml + rosiglitazone 1uM + T3 2nM + last 4 hours CL316243 10uM,,

AC,BIOTIME,GPL10588,MBA_8390,CASMC P6 (Lonza lot 0000200212) D14 Hystem BMP4 10ng/ml + rosiglitazone 1uM + T3 2nM + last 4 hours CL316243 10uM,,

AC,BIOTIME,GPL10588,MBA_8393,CASMC P6 (lot 0000200212) D14 Hystem BMP4 10ng/ml + rosiglitazone 1uM + T3 2nM + last 2 days FGF21 50ng/ml + last 4 hours CL316243 10uM,,

AC,BIOTIME,GPL10588,MBA_8394,CASMC P6 (lot 0000200212) D14 Hystem BMP4 10ng/ml + rosiglitazone 1uM + T3 2nM + last 2 days FGF21 50ng/ml + last 4 hours CL316243 10uM,,

AC,BIOTIME,GPL10588,MBA_8395,CASMC P6 (lot 0000200212) D14 Hystem BMP4 10ng/ml + rosiglitazone 1uM + T3 2nM + last 4 hours CL316243 10uM at 28C,,

AC,BIOTIME,GPL10588,MBA_8396,CASMC P6 (lot 0000200212) D14 Hystem BMP4 10ng/ml + rosiglitazone 1uM + T3 2nM + last 4 hours CL316243 10uM at 28C,,

AC,BIOTIME,GPL10588,MBA_8399,CASMC P4 (Lonza Lot#0000289727) Hystem BMP4 10ng/ml + rosiglitazone 1uM + T3 2nM + last 4 hours CL316243 10uM,,

AC,BIOTIME,GPL10588,MBA_8400,CASMC P4 (Lonza Lot#0000289727) Hystem BMP4 10ng/ml + rosiglitazone 1uM + T3 2nM + last 4 hours CL316243 10uM,,

AC,BIOTIME,GPL10588,MBA_8403,CASMC P4 (Lonza Lot#0000289727) D14 Hystem BMP4 10ng/ml + rosiglitazone 1uM + T3 2nM + last 2 days FGF21 50ng/ml + last 4 hours CL316243 10uM,,

AC,BIOTIME,GPL10588,MBA_8404,CASMC P4 (Lonza Lot#0000289727) D14 Hystem BMP4 10ng/ml + rosiglitazone 1uM + T3 2nM + last 2 days FGF21 50ng/ml + last 4 hours CL316243 10uM,,

AC,BIOTIME,GPL10588,MBA_8405,CASMC P4 (Lonza Lot#0000289727) D14 Hystem BMP4 10ng/ml + rosiglitazone 1uM + T3 2nM + last 4 hours CL316243 10uM at 28C,,

AC,BIOTIME,GPL10588,MBA_8406,CASMC P4 (Lonza Lot#0000289727) D14 Hystem BMP4 10ng/ml + rosiglitazone 1uM + T3 2nM + last 4 hours CL316243 10uM at 28C,,

AC,BIOTIME,GPL10588,MBA_8409,Omental Preadipocyte Zenbio (Lot SLOM-9) P9 Hystem BMP4 10ng/ml + rosiglitazone 1uM + T3 2nM + last 4 hours CL316243 10uM,,

AC,BIOTIME,GPL10588,MBA_8410,Omental Preadipocyte Zenbio (Lot SLOM-9) P9 Hystem BMP4 10ng/ml + rosiglitazone 1uM + T3 2nM + last 4 hours CL316243 10uM,,

AC,BIOTIME,GPL10588,MBA_8413,Omental Preadipocyte Zenbio (Lot SLOM-9) P9 Hystem BMP4 10ng/ml + rosiglitazone 1uM + T3 2nM + last 2 days FGF21 50ng/ml last 4 hours CL316243 10uM,,

AC,BIOTIME,GPL10588,MBA_8414,Omental Preadipocyte Zenbio (Lot SLOM-9) P9 Hystem BMP4 10ng/ml + rosiglitazone 1uM + T3 2nM + last 2 days FGF21 50ng/ml last 4 hours CL316243 10uM,,

AC,BIOTIME,GPL10588,MBA_8415,Omental Preadipocyte Zenbio (Lot SLOM-9) P9 Hystem BMP4 10ng/ml + rosiglitazone 1uM + T3 2nM + last 4 hours CL316243 10uM at 28C,,

AC,BIOTIME,GPL10588,MBA_8416,Omental Preadipocyte Zenbio (Lot SLOM-9) P9 Hystem BMP4 10ng/ml + rosiglitazone 1uM + T3 2nM + last 4 hours CL316243 10uM at 28C,,

iPSC,BIOTIME,GPL10588,MBA_8417,RP1-SKEL-8 P9 D14 Hystem BMP4 10ng/ml + rosiglitazone 1uM + T3 2nM + last 4 hours CL316243 10uM ,,

iPSC,BIOTIME,GPL10588,MBA_8418,RP1-SKEL-8 P9 D14 MM BMP4 10ng/ml + rosiglitazone 1uM + T3 2nM + last 4 hours CL316243 10uM ,,

iPSC,BIOTIME,GPL10588,MBA_8419,RP1-SKEL-8 P9 D14 confluence BMP4 10ng/ml + rosiglitazone 1uM + T3 2nM + last 4 hours CL316243 10uM ,,

iPSC,BIOTIME,GPL10588,MBA_8421,SK25 P10 D14 Hystem BMP4 10ng/ml + rosiglitazone 1uM + T3 2nM + last 4 hours CL316243 10uM,,

iPSC,BIOTIME,GPL10588,MBA_8422,SK25 P10 D14 Hystem rosiglitazone 1uM + T3 2nM + last 4 hours CL316243 10uM,,

iPSC,BIOTIME,GPL10588,MBA_8423,SK25 P10 D14 confluence rosiglitazone 1uM + T3 2nM + last 4 hours CL316243 10uM,,

iPSC,BIOTIME,GPL10588,MBA_8425,SK32 P9 D14 Hystem BMP4 10ng/ml + rosiglitazone 1uM + T3 2nM + last 4 hours CL316243 10uM,,

iPSC,BIOTIME,GPL10588,MBA_8426,SK32 P9 D14 Hystem rosiglitazone 1uM + T3 2nM + last 4 hours CL316243 10uM,,

iPSC,BIOTIME,GPL10588,MBA_8427,SK49 P10 ctrl,,

iPSC,BIOTIME,GPL10588,MBA_8428,SK49 P10 D14 Hystem BMP4 10ng/ml + rosiglitazone 1uM + T3 2nM + last 4 hours CL316243 10uM,,

iPSC,BIOTIME,GPL10588,MBA_8429,SK49 P10 D14 Hystem rosiglitazone 1uM + T3 2nM + last 4 hours CL316243 10uM,,

iPSC,BIOTIME,GPL10588,MBA_8430,SK49 P10 D14 confluence BMP4 10ng/ml + rosiglitazone 1uM + T3 2nM + last 4 hours CL316243 10uM,,

iPSC,BIOTIME,GPL10588,MBA_8431,SK49 P10 D14 confluence rosiglitazone 1uM + T3 2nM + last 4 hours CL316243 10uM,,

iPSC,BIOTIME,GPL10588,MBA_8433,SK20 P10 D14 Hystem BMP4 10ng/ml + rosiglitazone 1uM + T3 2nM + last 4 hours CL316243 10uM,,

iPSC,BIOTIME,GPL10588,MBA_8434,SK20 P10 D14 Hystem rosiglitazone 1uM + T3 2nM + last 4 hours CL316243 10uM,,

AC,BIOTIME,GPL10588,MBA_8437,Subcutaneous preadipocytes Zenbio (Lot SLOO36) P6 D14 Hystem BMP4 10ng/ml + rosiglitazone 1uM + T3 2nM + last 4 hours CL316243 10uM,,

AC,BIOTIME,GPL10588,MBA_8438,Subcutaneous preadipocytes Zenbio (Lot SLOO36) P6 D14 Hystem BMP4 10ng/ml + rosiglitazone 1uM + T3 2nM + last 4 hours CL316243 10uM,,

AC,BIOTIME,GPL10588,MBA_8441,Subcutaneous preadipocytes Zenbio (Lot SLOO36) P6 D14 Hystem BMP4 10ng/ml + rosiglitazone 1uM + T3 2nM + last 2 days FGF21 50ng/ml last 4 hours CL316243 10uM,,

AC,BIOTIME,GPL10588,MBA_8442,Subcutaneous preadipocytes Zenbio (Lot SLOO36) P6 D14 Hystem BMP4 10ng/ml + rosiglitazone 1uM + T3 2nM + last 2 days FGF21 50ng/ml last 4 hours CL316243 10uM,,

AC,BIOTIME,GPL10588,MBA_8443,Subcutaneous preadipocytes Zenbio (Lot SLOO36) P6 D14 Hystem BMP4 10ng/ml + rosiglitazone 1uM + T3 2nM + last 4 hours CL316243 10uM at 28C,,

AC,BIOTIME,GPL10588,MBA_8444,Subcutaneous preadipocytes Zenbio (Lot SLOO36) P6 D14 Hystem BMP4 10ng/ml + rosiglitazone 1uM + T3 2nM + last 4 hours CL316243 10uM at 28C,,

AC,BIOTIME,GPL10588,MBA_8447,Omental Preadipocyte Zenbio (Lot SLOM-18) P9 D14 Hystem BMP4 10ng/ml + rosiglitazone 1uM + T3 2nM + last 4 hours CL316243 10uM,,

AC,BIOTIME,GPL10588,MBA_8448,Omental Preadipocyte Zenbio (Lot SLOM-18) P9 D14 Hystem BMP4 10ng/ml + rosiglitazone 1uM + T3 2nM + last 4 hours CL316243 10uM,,

AC,BIOTIME,GPL10588,MBA_8451,Omental Preadipocyte Zenbio (Lot SLOM-18) P9 D14 Hystem BMP4 10ng/ml + rosiglitazone 1uM + T3 2nM + last 2 days FGF21 50ng/ml + last 4 hours CL316243 10uM,,

AC,BIOTIME,GPL10588,MBA_8452,Omental Preadipocyte Zenbio (Lot SLOM-18) P9 D14 Hystem BMP4 10ng/ml + rosiglitazone 1uM + T3 2nM + last 2 days FGF21 50ng/ml + last 4 hours CL316243 10uM,,

AC,BIOTIME,GPL10588,MBA_8453,Omental Preadipocyte Zenbio (Lot SLOM-18) P9 D14 Hystem BMP4 10ng/ml + rosiglitazone 1uM + T3 2nM + last 4 hours CL316243 10uM at 28C,,

AC,BIOTIME,GPL10588,MBA_8454,Omental Preadipocyte Zenbio (Lot SLOM-18) P9 D14 Hystem BMP4 10ng/ml + rosiglitazone 1uM + T3 2nM + last 4 hours CL316243 10uM at 28C,,

AC,BIOTIME,GPL10588,MBA_8455,Subcutaneous preadipocytes (Zenbio) P5 D14 confluence BMP4 10ng/ml + rosiglitazone 1uM + T3 2nM + last 4 hours CL316243 10uM,,

AC,BIOTIME,GPL10588,MBA_8456,Subcutaneous preadipocytes (Zenbio) P5 D14 confluence rosiglitazone 1uM + T3 2nM + last 4 hours CL316243 10uM,,

iPSC,BIOTIME,GPL10588,MBA_8457,SK20 P10 D14 confluence BMP4 10ng/ml + rosiglitazone 1uM + T3 2nM + last 4 hours CL316243 10uM,,

iPSC,BIOTIME,GPL10588,MBA_8458,SK20 P10 D14 confluence rosiglitazone 1uM + T3 2nM + last 4 hours CL316243 10uM,,

iPSC,BIOTIME,GPL10588,MBA_8459,SK8 P10 D14 confluence BMP4 10ng/ml + rosiglitazone 1uM + T3 2nM + last 4 hours CL316243 10uM,,

iPSC,BIOTIME,GPL10588,MBA_8460,SK8 P10 D14 confluence rosiglitazone 1uM + T3 2nM + last 4 hours CL316243 10uM,,

iPSC,BIOTIME,GPL10588,MBA_8461,SK8 P10 D14 Hystem BMP4 10ng/ml + rosiglitazone 1uM + T3 2nM + last 4 hours CL316243 10uM,,

iPSC,BIOTIME,GPL10588,MBA_8462,SK8 P10 D14 Hystem rosiglitazone 1uM + T3 2nM + last 4 hours CL316243 10uM,,

iPSC,BIOTIME,GPL10588,MBA_8464,ESI004 NP77 EN P8 D14 Hystem BMP4 10ng/ml +rosiglitazone 1uM + T3 2nM + last 4 hours CL316243 10uM,,

iPSC,BIOTIME,GPL10588,MBA_8465,ESI004 NP77 EN P8 D14 Hystem rosiglitazone 1uM + T3 2nM + last 4 hours CL316243 10uM,,

iPSC,BIOTIME,GPL10588,MBA_8466,ESI004 NP77 EN P8 D14 confluence BMP4 10ng/ml +rosiglitazone 1uM + T3 2nM + last 4 hours CL316243 10uM,,

iPSC,BIOTIME,GPL10588,MBA_8467,ESI004 NP77 EN P8 D14 confluence rosiglitazone 1uM + T3 2nM + last 4 hours CL316243 10uM,,

iPSC,BIOTIME,GPL10588,MBA_8469,ESI004 NP78 EN P7 D14 Hystem BMP4 10ng/ml +rosiglitazone 1uM + T3 2nM + last 4 hours CL316243 10uM,,

iPSC,BIOTIME,GPL10588,MBA_8470,ESI004 NP78 EN P7 D14 Hystem rosiglitazone 1uM + T3 2nM + last 4 hours CL316243 10uM,,

iPSC,BIOTIME,GPL10588,MBA_8471,ESI004 NP78 EN P7 D14 confluence BMP4 10ng/ml +rosiglitazone 1uM + T3 2nM + last 4 hours CL316243 10uM,,

iPSC,BIOTIME,GPL10588,MBA_8472,ESI004 NP78 EN P7 D14 confluence rosiglitazone 1uM + T3 2nM + last 4 hours CL316243 10uM,,

iPSC,BIOTIME,GPL10588,MBA_8474,ESI004 NP80 EN P8 D14 Hystem BMP4 10ng/ml +rosiglitazone 1uM + T3 2nM + last 4 hours CL316243 10uM,,

iPSC,BIOTIME,GPL10588,MBA_8475,ESI004 NP80 EN P8 D14 Hystem rosiglitazone 1uM + T3 2nM + last 4 hours CL316243 10uM,,

iPSC,BIOTIME,GPL10588,MBA_8476,ESI004 NP80 EN P8 D14 confluence BMP4 10ng/ml +rosiglitazone 1uM + T3 2nM + last 4 hours CL316243 10uM,,

iPSC,BIOTIME,GPL10588,MBA_8477,ESI004 NP80 EN P8 D14 confluence rosiglitazone 1uM + T3 2nM + last 4 hours CL316243 10uM,,

iPSC,BIOTIME,GPL10588,MBA_8479,ESI004 EN63 P10 D14 Hystem BMP4 10ng/ml +rosiglitazone 1uM + T3 2nM + last 4 hours CL316243 10uM,,

iPSC,BIOTIME,GPL10588,MBA_8480,ESI004 EN63 P10 D14 Hystem rosiglitazone 1uM + T3 2nM + last 4 hours CL316243 10uM,,

iPSC,BIOTIME,GPL10588,MBA_8481,ESI004 EN63 P10 D14 confluence BMP4 10ng/ml +rosiglitazone 1uM + T3 2nM + last 4 hours CL316243 10uM,,

iPSC,BIOTIME,GPL10588,MBA_8482,ESI004 EN63 P10 D14 confluence rosiglitazone 1uM + T3 2nM + last 4 hours CL316243 10uM,,

iPSC,BIOTIME,GPL10588,MBA_8484,ESI004 NP92 SM P8 D14 Hystem BMP4 10ng/ml +rosiglitazone 1uM + T3 2nM + last 4 hours CL316243 10uM,,

iPSC,BIOTIME,GPL10588,MBA_8485,ESI004 NP92 SM P8 D14 Hystem rosiglitazone 1uM + T3 2nM + last 4 hours CL316243 10uM,,

iPSC,BIOTIME,GPL10588,MBA_8486,ESI004 NP92 SM P8 D14 confluence BMP4 10ng/ml +rosiglitazone 1uM + T3 2nM + last 4 hours CL316243 10uM,,

iPSC,BIOTIME,GPL10588,MBA_8487,ESI004 NP92 SM P8 D14 confluence rosiglitazone 1uM + T3 2nM + last 4 hours CL316243 10uM,,

AC,BIOTIME,GPL10588,MBA_8495,hMSC (PromoCell) P7 D14 Hystem BMP4 10ng/ml + rosiglitazone 1uM + T3 2nM + last 4 hours CL316243 10uM,,

AC,BIOTIME,GPL10588,MBA_8496,hMSC (PromoCell) P7 D14 Hystem BMP4 10ng/ml + rosiglitazone 1uM + T3 2nM + last 4 hours CL316243 10uM,,

AC,BIOTIME,GPL10588,MBA_8497,hMSC (PromoCell) P7 D14 Hystem BMP4 10ng/ml + rosiglitazone 1uM + T3 2nM + last 4 hours CL316243 10uM,,

AC,BIOTIME,GPL10588,MBA_8500,hMSC (PromoCell) P7 D14 Hystem BMP4 10ng/ml + rosiglitazone 1uM + T3 2nM + last 2 days FGF21 50ng/ml + last 4 hours CL316243 10uM,,

AC,BIOTIME,GPL10588,MBA_8501,hMSC (PromoCell) P7 D14 Hystem BMP4 10ng/ml + rosiglitazone 1uM + T3 2nM + last 2 days FGF21 50ng/ml + last 4 hours CL316243 10uM,,

AC,BIOTIME,GPL10588,MBA_8504,hMSC (Lonza) P10 D14 Hystem BMP4 10ng/ml + rosiglitazone 1uM + T3 2nM + last 4 hours CL316243 10uM,,

AC,BIOTIME,GPL10588,MBA_8505,hMSC (Lonza) P10 D14 Hystem BMP4 10ng/ml + rosiglitazone 1uM + T3 2nM + last 4 hours CL316243 10uM,,

AC,BIOTIME,GPL10588,MBA_8508,hMSC (Lonza) P10 D14 Hystem BMP4 10ng/ml + rosiglitazone 1uM + T3 2nM + last 2 days FGF21 50ng/ml + last 4 hours CL316243 10uM,,

AC,BIOTIME,GPL10588,MBA_8509,hMSC (Lonza) P10 D14 Hystem BMP4 10ng/ml + rosiglitazone 1uM + T3 2nM + last 2 days FGF21 50ng/ml + last 4 hours CL316243 10uM,,

iPSC,BIOTIME,GPL10588,MBA_8512,C4ELS5.1 P14 D14 confluence BMP4 10ng/ml + rosiglitazone 1uM + T3 2nM + last 4 hours CL316243 10uM ,,

iPSC,BIOTIME,GPL10588,MBA_8513,C4ELS5.1 P14 D14 confluence BMP4 10ng/ml + rosiglitazone 1uM + T3 2nM + last 4 hours CL316243 10uM ,,

iPSC,BIOTIME,GPL10588,MBA_8514,C4ELS5.1 P14 D14 confluence rosiglitazone 1uM + T3 2nM + last 4 hours CL316243 10uM ,,

iPSC,BIOTIME,GPL10588,MBA_8515,C4ELS5.1 P14 D14 confluence rosiglitazone 1uM + T3 2nM + last 4 hours CL316243 10uM ,,

iPSC,BIOTIME,GPL10588,MBA_8517,ESI004 NP59 EN P11 D14 Hystem BMP4 10ng/ml + rosiglitazone 1uM + T3 2nM + last 4 hours CL316243 10uM,,

iPSC,BIOTIME,GPL10588,MBA_8518,ESI004 NP59 EN P11 D14 Hystem rosiglitazone 1uM + T3 2nM + last 4 hours CL316243 10uM,,

iPSC,BIOTIME,GPL10588,MBA_8519,ESI004 NP59 EN P11 D14 confluence BMP4 10ng/ml + rosiglitazone 1uM + T3 2nM + last 4 hours CL316243 10uM,,

iPSC,BIOTIME,GPL10588,MBA_8520,ESI004 NP59 EN P11 D14 confluence rosiglitazone 1uM + T3 2nM + last 4 hours CL316243 10uM,,

iPSC,BIOTIME,GPL10588,MBA_8522,ESI004 NP62 SM P11 D14 confluence BMP4 10ng/ml + rosiglitazone 1uM + T3 2nM + last 4 hours CL316243 10uM,,

iPSC,BIOTIME,GPL10588,MBA_8523,ESI004 NP62 SM P11 D14 confluence rosiglitazone 1uM + T3 2nM + last 4 hours CL316243 10uM,,

iPSC,BIOTIME,GPL10588,MBA_8524,ESI004 NP62 SM P11 D14 Hystem BMP4 10ng/ml + rosiglitazone 1uM + T3 2nM + last 4 hours CL316243 10uM,,

iPSC,BIOTIME,GPL10588,MBA_8525,ESI004 NP62 SM P11 D14 Hystem rosiglitazone 1uM + T3 2nM + last 4 hours CL316243 10uM,,

iPSC,BIOTIME,GPL10588,MBA_8527,ESI004 NP91 SM P8 D14 Hystem BMP4 10ng/ml + rosiglitazone 1uM + T3 2nM + last 4 hours CL316243 10uM,,

iPSC,BIOTIME,GPL10588,MBA_8528,ESI004 NP91 SM P8 D14 Hystem rosiglitazone 1uM + T3 2nM + last 4 hours CL316243 10uM,,

iPSC,BIOTIME,GPL10588,MBA_8529,ESI004 NP91 SM P8 D14 confluence BMP4 10ng/ml +rosiglitazone 1uM + T3 2nM + last 4 hours CL316243 10uM,,

iPSC,BIOTIME,GPL10588,MBA_8530,ESI004 NP91 SM P8 D14 confluence rosiglitazone 1uM + T3 2nM + last 4 hours CL316243 10uM,,

iPSC,BIOTIME,GPL10588,MBA_8532,ESI004 NP93 SM P9 D14 Hystem BMP4 10ng/ml + rosiglitazone 1uM + T3 2nM + last 4 hours CL316243 10uM,,

iPSC,BIOTIME,GPL10588,MBA_8533,ESI004 NP93 SM P9 D14 Hystem rosiglitazone 1uM + T3 2nM + last 4 hours CL316243 10uM,,

iPSC,BIOTIME,GPL10588,MBA_8534,ESI004 NP93 SM P9 D14 confluence BMP4 10ng/ml + rosiglitazone 1uM + T3 2nM + last 4 hours CL316243 10uM,,

iPSC,BIOTIME,GPL10588,MBA_8535,ESI004 NP93 SM P9 D14 confluence rosiglitazone 1uM + T3 2nM + last 4 hours CL316243 10uM,,

iPSC,BIOTIME,GPL10588,MBA_8537,ESI004 NP95 EN P8 D14 Hystem BMP4 10ng/ml + rosiglitazone 1uM + T3 2nM + last 4 hours CL316243 10uM,,

iPSC,BIOTIME,GPL10588,MBA_8538,ESI004 NP95 EN P8 D14 Hystem rosiglitazone 1uM + T3 2nM + last 4 hours CL316243 10uM,,

iPSC,BIOTIME,GPL10588,MBA_8539,ESI004 NP95 EN P8 D14 confluence BMP4 10ng/ml + rosiglitazone 1uM + T3 2nM + last 4 hours CL316243 10uM,,

iPSC,BIOTIME,GPL10588,MBA_8540,ESI004 NP95 EN P8 D14 confluence rosiglitazone 1uM + T3 2nM + last 4 hours CL316243 10uM,,

iPSC,BIOTIME,GPL10588,MBA_8542,ESI004 NP113 SM P10 D14 Hystem BMP4 10ng/ml + rosiglitazone 1uM + T3 2nM + last 4 hours CL316243 10uM,,

iPSC,BIOTIME,GPL10588,MBA_8543,ESI004 NP113 SM P10 D14 Hystem rosiglitazone 1uM + T3 2nM + last 4 hours CL316243 10uM,,

iPSC,BIOTIME,GPL10588,MBA_8544,ESI004 NP113 SM P10 D14 confluence BMP4 10ng/ml + rosiglitazone 1uM + T3 2nM + last 4 hours CL316243 10uM,,

iPSC,BIOTIME,GPL10588,MBA_8545,ESI004 NP113 SM P10 D14 confluence rosiglitazone 1uM + T3 2nM + last 4 hours CL316243 10uM,,

iPSC,BIOTIME,GPL10588,MBA_8547,SK60 P11 D14 Hystem BMP4 10ng/ml + rosiglitazone 1uM + T3 2nM + last 4 hours CL316243 10uM,,

iPSC,BIOTIME,GPL10588,MBA_8548,SK60 P11 D14 Hystem rosiglitazone 1uM + T3 2nM + last 4 hours CL316243 10uM,,

iPSC,BIOTIME,GPL10588,MBA_8549,SK60 P11 D14 confluence BMP4 10ng/ml + rosiglitazone 1uM + T3 2nM + last 4 hours CL316243 10uM,,

iPSC,BIOTIME,GPL10588,MBA_8550,SK60 P11 D14 confluence rosiglitazone 1uM + T3 2nM + last 4 hours CL316243 10uM,,

AC,BIOTIME,GPL10588,MBA_8553,Subcutaneous preadipocytes Zenbio (Lot SLOO54) P6 D14 Hystem BMP4 10ng/ml + rosiglitazone 1uM + T3 2nM + last 4 hours CL316243 10uM,,

AC,BIOTIME,GPL10588,MBA_8554,Subcutaneous preadipocytes Zenbio (Lot SLOO54) P6 D14 Hystem BMP4 10ng/ml + rosiglitazone 1uM + T3 2nM + last 4 hours CL316243 10uM,,

AC,BIOTIME,GPL10588,MBA_8557,Subcutaneous preadipocytes Zenbio (Lot SLOO54) P6 D14 Hystem BMP4 10ng/ml + rosiglitazone 1uM + T3 2nM + last 2 days FGF21 50ng/ml last 4 hours CL316243 10uM,,

AC,BIOTIME,GPL10588,MBA_8558,Subcutaneous preadipocytes Zenbio (Lot SLOO54) P6 D14 Hystem BMP4 10ng/ml + rosiglitazone 1uM + T3 2nM + last 2 days FGF21 50ng/ml last 4 hours CL316243 10uM,,

AC,BIOTIME,GPL10588,MBA_8559,Subcutaneous preadipocytes Zenbio (Lot SLOO54) P6 D14 Hystem BMP4 10ng/ml + rosiglitazone 1uM + T3 2nM + last 4 hours CL316243 10uM at 28C,,

AC,BIOTIME,GPL10588,MBA_8560,Subcutaneous preadipocytes Zenbio (Lot SLOO54) P6 D14 Hystem BMP4 10ng/ml + rosiglitazone 1uM + T3 2nM + last 4 hours CL316243 10uM at 28C,,

iPSC,BIOTIME,GPL10588,MBA_8562,MEL2 P17 D14 Hystem BMP4 10ng/ml + rosiglitazone 1uM + T3 2nM + last 4 hours CL316243 10uM,,

iPSC,BIOTIME,GPL10588,MBA_8563,MEL2 P17 D14 Hystem rosiglitazone 1uM + T3 2nM + last 4 hours CL316243 10uM,,

iPSC,BIOTIME,GPL10588,MBA_8564,MEL2 P17 D14 confluence BMP4 10ng/ml + rosiglitazone 1uM + T3 2nM + last 4 hours CL316243 10uM,,

iPSC,BIOTIME,GPL10588,MBA_8565,MEL2 P17 D14 confluence rosiglitazone 1uM + T3 2nM + last 4 hours CL316243 10uM,,

AC,BIOTIME,GPL10588,MBA_8566,MDW-1 (Cl.1) NP1-3.14 P5 ctrl,,

AC,BIOTIME,GPL10588,MBA_8567,MDW-1 (Cl.1) NP1-3.14 P5 D14 confluence BMP4 10ng/ml + rosiglitazone 1uM + T3 2nM + last 4 hours CL316243 10uM,,

AC,BIOTIME,GPL10588,MBA_8568,MDW-1 (Cl.1) NP1-3.14 P5 D14 confluence rosiglitazone 1uM + T3 2nM + last 4 hours CL316243 10uM,,

AC,BIOTIME,GPL10588,MBA_8569,MDW-1 (Cl.1) NP1-3.21 P5 ctrl,,

AC,BIOTIME,GPL10588,MBA_8570,MDW-1 (Cl.1) NP1-3.21 P5 D14 confluence BMP4 10ng/ml + rosiglitazone 1uM + T3 2nM + last 4 hours CL316243 10uM,,

AC,BIOTIME,GPL10588,MBA_8571,MDW-1 (Cl.1) NP1-3.21 P5 D14 confluence rosiglitazone 1uM + T3 2nM + last 4 hours CL316243 10uM,,

AC,BIOTIME,GPL10588,MBA_8572,MDW-1 (Cl.1) NP1-1.1 P5 ctrl,,

AC,BIOTIME,GPL10588,MBA_8573,MDW-1 (Cl.1) NP1-1.1 P5 D14 confluence BMP4 10ng/ml + rosiglitazone 1uM + T3 2nM + last 4 hours CL316243 10uM,,

AC,BIOTIME,GPL10588,MBA_8574,MDW-1 (Cl.1) NP1-1.1 P5 D14 confluence rosiglitazone 1uM + T3 2nM + last 4 hours CL316243 10uM,,

AC,BIOTIME,GPL10588,MBA_8575,MDW-1 (Cl.1) NP1-3.22 P5 ctrl,,

AC,BIOTIME,GPL10588,MBA_8576,MDW-1 (Cl.1) NP1-3.22 P5 D14 confluence rosiglitazone 1uM + T3 2nM + last 4 hours CL316243 10uM,,

AC,BIOTIME,GPL10588,MBA_8577,MDW-1 (Cl.1) NP1-1.4 P5 ctrl,,

AC,BIOTIME,GPL10588,MBA_8578,MDW-1 (Cl.1) NP1-1.4 P5 D14 confluence rosiglitazone 1uM + T3 2nM + last 4 hours CL316243 10uM,,

AC,BIOTIME,GPL10588,MBA_8579,MDW-1 (Cl.1) NP1-3.5 P5 ctrl,,

AC,BIOTIME,GPL10588,MBA_8580,MDW-1 (Cl.1) NP1-3.5 P5 D14 confluence BMP4 10ng/ml + rosiglitazone 1uM + T3 2nM + last 4 hours CL316243 10uM,,

AC,BIOTIME,GPL10588,MBA_8581,MDW-1 (Cl.1) NP1-3.5 P5 D14 confluence rosiglitazone 1uM + T3 2nM + last 4 hours CL316243 10uM,,

AC,BIOTIME,GPL10588,MBA_8582,MDW-1 (Cl.1) NP1-3.12 P5 ctrl,,

AC,BIOTIME,GPL10588,MBA_8583,MDW-1 (Cl.1) NP1-3.12 P5 D14 confluence BMP4 10ng/ml + rosiglitazone 1uM + T3 2nM + last 4 hours CL316243 10uM,,

AC,BIOTIME,GPL10588,MBA_8584,MDW-1 (Cl.1) NP1-3.12 P5 D14 confluence rosiglitazone 1uM + T3 2nM + last 4 hours CL316243 10uM,,

AC,BIOTIME,GPL10588,MBA_8585,MDW-1 (Cl.1) NP1-3.7 P5 ctrl,,

AC,BIOTIME,GPL10588,MBA_8586,MDW-1 (Cl.1) NP1-3.7 P5 D14 confluence BMP4 10ng/ml + rosiglitazone 1uM + T3 2nM + last 4 hours CL316243 10uM,,

AC,BIOTIME,GPL10588,MBA_8587,MDW-1 (Cl.1) NP1-3.7 P5 D14 confluence rosiglitazone 1uM + T3 2nM + last 4 hours CL316243 10uM,,

AC,BIOTIME,GPL10588,MBA_8588,MDW-1 (Cl.1) NP1-1.7 P5 ctrl,,

AC,BIOTIME,GPL10588,MBA_8589,MDW-1 (Cl.1) NP1-1.7 P5 D14 confluence BMP4 10ng/ml + rosiglitazone 1uM + T3 2nM + last 4 hours CL316243 10uM,,

AC,BIOTIME,GPL10588,MBA_8590,MDW-1 (Cl.1) NP1-1.7 P5 D14 confluence rosiglitazone 1uM + T3 2nM + last 4 hours CL316243 10uM,,

AC,BIOTIME,GPL10588,MBA_8591,MDW-1 (Cl.1) NP1-3.8 P5 ctrl,,

AC,BIOTIME,GPL10588,MBA_8592,MDW-1 (Cl.1) NP1-3.8 P5 D14 confluence BMP4 10ng/ml + rosiglitazone 1uM + T3 2nM + last 4 hours CL316243 10uM,,

AC,BIOTIME,GPL10588,MBA_8593,MDW-1 (Cl.1) NP1-3.8 P5 D14 confluence rosiglitazone 1uM + T3 2nM + last 4 hours CL316243 10uM,,

AC,BIOTIME,GPL10588,MBA_8594,MDW-1 (Cl.1) NP1-1.10 P5 ctrl,,

AC,BIOTIME,GPL10588,MBA_8595,MDW-1 (Cl.1) NP1-1.10 P5 D14 confluence BMP4 10ng/ml + rosiglitazone 1uM + T3 2nM + last 4 hours CL316243 10uM,,

AC,BIOTIME,GPL10588,MBA_8596,MDW-1 (Cl.1) NP1-1.10 P5 D14 confluence rosiglitazone 1uM + T3 2nM + last 4 hours CL316243 10uM,,

AC,BIOTIME,GPL10588,MBA_8597,MDW-1 (Cl.1) NP1-1.24 P5 ctrl,,

AC,BIOTIME,GPL10588,MBA_8598,MDW-1 (Cl.1) NP1-1.24 P5 D14 confluence BMP4 10ng/ml + rosiglitazone 1uM + T3 2nM + last 4 hours CL316243 10uM,,

AC,BIOTIME,GPL10588,MBA_8599,MDW-1 (Cl.1) NP1-1.24 P5 D14 confluence rosiglitazone 1uM + T3 2nM + last 4 hours CL316243 10uM,,

AC,BIOTIME,GPL10588,MBA_8600,MDW-1 (Cl.1) NP1-3.1 P5 ctrl,,

AC,BIOTIME,GPL10588,MBA_8601,MDW-1 (Cl.1) NP1-3.1 P5 D14 confluence BMP4 10ng/ml + rosiglitazone 1uM + T3 2nM + last 4 hours CL316243 10uM,,

AC,BIOTIME,GPL10588,MBA_8602,MDW-1 (Cl.1) NP1-3.1 P5 D14 confluence rosiglitazone 1uM + T3 2nM + last 4 hours CL316243 10uM,,

AC,BIOTIME,GPL10588,MBA_8603,MDW-1 (Cl.1) NP1-3.15 P5 ctrl,,

AC,BIOTIME,GPL10588,MBA_8604,MDW-1 (Cl.1) NP1-3.15 P5 D14 confluence BMP4 10ng/ml + rosiglitazone 1uM + T3 2nM + last 4 hours CL316243 10uM,,

AC,BIOTIME,GPL10588,MBA_8605,MDW-1 (Cl.1) NP1-3.15 P5 D14 confluence rosiglitazone 1uM + T3 2nM + last 4 hours CL316243 10uM,,

AC,BIOTIME,GPL10588,MBA_8606,MDW-1 (Cl.1) NP1-1.11 P5 ctrl,,

AC,BIOTIME,GPL10588,MBA_8607,MDW-1 (Cl.1) NP1-1.11 P5 D14 confluence BMP4 10ng/ml + rosiglitazone 1uM + T3 2nM + last 4 hours CL316243 10uM,,

AC,BIOTIME,GPL10588,MBA_8608,MDW-1 (Cl.1) NP1-1.11 P5 D14 confluence rosiglitazone 1uM + T3 2nM + last 4 hours CL316243 10uM,,

AC,BIOTIME,GPL10588,MBA_8609,MDW-1 (Cl.1) NP1-1.8 P5 ctrl,,

AC,BIOTIME,GPL10588,MBA_8610,MDW-1 (Cl.1) NP1-1.8 P5 D14 confluence BMP4 10ng/ml + rosiglitazone 1uM + T3 2nM + last 4 hours CL316243 10uM,,

AC,BIOTIME,GPL10588,MBA_8611,MDW-1 (Cl.1) NP1-1.8 P5 D14 confluence rosiglitazone 1uM + T3 2nM + last 4 hours CL316243 10uM,,

AC,BIOTIME,GPL10588,MBA_8612,MDW-1 (Cl.1) NP1-3.10 P5 ctrl,,

AC,BIOTIME,GPL10588,MBA_8613,MDW-1 (Cl.1) NP1-3.10 P5 D14 confluence BMP4 10ng/ml + rosiglitazone 1uM + T3 2nM + last 4 hours CL316243 10uM,,

AC,BIOTIME,GPL10588,MBA_8614,MDW-1 (Cl.1) NP1-3.10 P5 D14 confluence rosiglitazone 1uM + T3 2nM + last 4 hours CL316243 10uM,,

AC,BIOTIME,GPL10588,MBA_8615,MDW-1 (Cl.1) NP1-3.18 P5 ctrl,,

AC,BIOTIME,GPL10588,MBA_8616,MDW-1 (Cl.1) NP1-3.18 P5 D14 confluence BMP4 10ng/ml + rosiglitazone 1uM + T3 2nM + last 4 hours CL316243 10uM,,

AC,BIOTIME,GPL10588,MBA_8617,MDW-1 (Cl.1) NP1-3.18 P5 D14 confluence rosiglitazone 1uM + T3 2nM + last 4 hours CL316243 10uM,,

AC,BIOTIME,GPL10588,MBA_8618,MDW-1 (Cl.1) NP1-2.30 P5 ctrl,,

AC,BIOTIME,GPL10588,MBA_8619,MDW-1 (Cl.1) NP1-2.30 P5 D14 confluence BMP4 10ng/ml + rosiglitazone 1uM + T3 2nM + last 4 hours CL316243 10uM,,

AC,BIOTIME,GPL10588,MBA_8620,MDW-1 (Cl.1) NP1-2.30 P5 D14 confluence rosiglitazone 1uM + T3 2nM + last 4 hours CL316243 10uM,,

AC,BIOTIME,GPL10588,MBA_8621,MDW-1 (Cl.1) NP1-2.29 P5 ctrl,,

AC,BIOTIME,GPL10588,MBA_8622,MDW-1 (Cl.1) NP1-2.29 P5 D14 confluence BMP4 10ng/ml + rosiglitazone 1uM + T3 2nM + last 4 hours CL316243 10uM,,

AC,BIOTIME,GPL10588,MBA_8623,MDW-1 (Cl.1) NP1-2.29 P5 D14 confluence rosiglitazone 1uM + T3 2nM + last 4 hours CL316243 10uM,,

AC,BIOTIME,GPL10588,MBA_8624,MDW-1 (Cl.1) NP1-2.31 P5 ctrl,,

AC,BIOTIME,GPL10588,MBA_8625,MDW-1 (Cl.1) NP1-2.31 P5 D14 confluence BMP4 10ng/ml + rosiglitazone 1uM + T3 2nM + last 4 hours CL316243 10uM,,

AC,BIOTIME,GPL10588,MBA_8626,MDW-1 (Cl.1) NP1-2.31 P5 D14 confluence rosiglitazone 1uM + T3 2nM + last 4 hours CL316243 10uM,,

AC,BIOTIME,GPL10588,MBA_8627,MDW-1 (Cl.1) NP1-2.32 P5 ctrl,,

AC,BIOTIME,GPL10588,MBA_8628,MDW-1 (Cl.1) NP1-2.32 P5 D14 confluence BMP4 10ng/ml + rosiglitazone 1uM + T3 2nM + last 4 hours CL316243 10uM,,

AC,BIOTIME,GPL10588,MBA_8629,MDW-1 (Cl.1) NP1-2.32 P5 D14 confluence rosiglitazone 1uM + T3 2nM + last 4 hours CL316243 10uM,,

AC,BIOTIME,GPL10588,MBA_8630,MDW-1 (Cl.1) NP1-2.2 P5 ctrl,,

AC,BIOTIME,GPL10588,MBA_8631,MDW-1 (Cl.1) NP1-2.2 P5 D14 confluence BMP4 10ng/ml + rosiglitazone 1uM + T3 2nM + last 4 hours CL316243 10uM,,

AC,BIOTIME,GPL10588,MBA_8632,MDW-1 (Cl.1) NP1-2.2 P5 D14 confluence rosiglitazone 1uM + T3 2nM + last 4 hours CL316243 10uM,,

AC,BIOTIME,GPL10588,MBA_8633,MDW-1 (Cl.1) NP1-2.4 P5 ctrl,,

AC,BIOTIME,GPL10588,MBA_8634,MDW-1 (Cl.1) NP1-2.4 P5 D14 confluence BMP4 10ng/ml + rosiglitazone 1uM + T3 2nM + last 4 hours CL316243 10uM,,

AC,BIOTIME,GPL10588,MBA_8635,MDW-1 (Cl.1) NP1-2.4 P5 D14 confluence rosiglitazone 1uM + T3 2nM + last 4 hours CL316243 10uM,,

AC,BIOTIME,GPL10588,MBA_8636,MDW-1 (Cl.1) NP1-2.45 P5 ctrl,,

AC,BIOTIME,GPL10588,MBA_8637,MDW-1 (Cl.1) NP1-2.45 P5 D14 confluence BMP4 10ng/ml + rosiglitazone 1uM + T3 2nM + last 4 hours CL316243 10uM,,

AC,BIOTIME,GPL10588,MBA_8638,MDW-1 (Cl.1) NP1-2.45 P5 D14 confluence rosiglitazone 1uM + T3 2nM + last 4 hours CL316243 10uM,,

AC,BIOTIME,GPL10588,MBA_8639,MDW-1 (Cl.1) NP1-2.3 P5 ctrl,,

AC,BIOTIME,GPL10588,MBA_8640,MDW-1 (Cl.1) NP1-2.3 P5 D14 confluence BMP4 10ng/ml + rosiglitazone 1uM + T3 2nM + last 4 hours CL316243 10uM,,

AC,BIOTIME,GPL10588,MBA_8641,MDW-1 (Cl.1) NP1-2.3 P5 D14 confluence rosiglitazone 1uM + T3 2nM + last 4 hours CL316243 10uM,,

AC,BIOTIME,GPL10588,MBA_8642,MDW-1 (Cl.1) NP1-2.16 P5 ctrl,,

AC,BIOTIME,GPL10588,MBA_8643,MDW-1 (Cl.1) NP1-2.16 P5 D14 confluence BMP4 10ng/ml + rosiglitazone 1uM + T3 2nM + last 4 hours CL316243 10uM,,

AC,BIOTIME,GPL10588,MBA_8644,MDW-1 (Cl.1) NP1-2.16 P5 D14 confluence rosiglitazone 1uM + T3 2nM + last 4 hours CL316243 10uM,,

AC,BIOTIME,GPL10588,MBA_8645,MDW-1 (Cl.1) NP1-2.8 P5 ctrl,,

AC,BIOTIME,GPL10588,MBA_8646,MDW-1 (Cl.1) NP1-2.8 P5 D14 confluence BMP4 10ng/ml + rosiglitazone 1uM + T3 2nM + last 4 hours CL316243 10uM,,

AC,BIOTIME,GPL10588,MBA_8647,MDW-1 (Cl.1) NP1-2.8 P5 D14 confluence rosiglitazone 1uM + T3 2nM + last 4 hours CL316243 10uM,,

AC,BIOTIME,GPL10588,MBA_8648,MDW-1 (Cl.1) NP1-2.11 P5 ctrl,,

AC,BIOTIME,GPL10588,MBA_8649,MDW-1 (Cl.1) NP1-2.11 P5 D14 confluence BMP4 10ng/ml + rosiglitazone 1uM + T3 2nM + last 4 hours CL316243 10uM,,

AC,BIOTIME,GPL10588,MBA_8650,MDW-1 (Cl.1) NP1-2.11 P5 D14 confluence rosiglitazone 1uM + T3 2nM + last 4 hours CL316243 10uM,,

AC,BIOTIME,GPL10588,MBA_8651,MDW-1 (Cl.1) NP1-2.10 P5 ctrl,,

AC,BIOTIME,GPL10588,MBA_8652,MDW-1 (Cl.1) NP1-2.10 P5 D14 confluence BMP4 10ng/ml + rosiglitazone 1uM + T3 2nM + last 4 hours CL316243 10uM,,

AC,BIOTIME,GPL10588,MBA_8653,MDW-1 (Cl.1) NP1-2.10 P5 D14 confluence rosiglitazone 1uM + T3 2nM + last 4 hours CL316243 10uM,,

AC,BIOTIME,GPL10588,MBA_8654,MDW-1 (Cl.1) NP1-1.38 P5 ctrl,,

AC,BIOTIME,GPL10588,MBA_8655,MDW-1 (Cl.1) NP1-1.38 P5 D14 confluence BMP4 10ng/ml + rosiglitazone 1uM + T3 2nM + last 4 hours CL316243 10uM,,

AC,BIOTIME,GPL10588,MBA_8656,MDW-1 (Cl.1) NP1-1.38 P5 D14 confluence rosiglitazone 1uM + T3 2nM + last 4 hours CL316243 10uM,,

AC,BIOTIME,GPL10588,MBA_8657,MDW-1 (Cl.1) NP1-2.21 P5 ctrl,,

AC,BIOTIME,GPL10588,MBA_8658,MDW-1 (Cl.1) NP1-2.21 P5 D14 confluence BMP4 10ng/ml + rosiglitazone 1uM + T3 2nM + last 4 hours CL316243 10uM,,

AC,BIOTIME,GPL10588,MBA_8659,MDW-1 (Cl.1) NP1-2.21 P5 D14 confluence rosiglitazone 1uM + T3 2nM + last 4 hours CL316243 10uM,,

AC,BIOTIME,GPL10588,MBA_8660,MDW-1 (Cl.1) NP1-2.25 P5 ctrl,,

AC,BIOTIME,GPL10588,MBA_8661,MDW-1 (Cl.1) NP1-2.25 P5 D14 confluence BMP4 10ng/ml + rosiglitazone 1uM + T3 2nM + last 4 hours CL316243 10uM,,

AC,BIOTIME,GPL10588,MBA_8662,MDW-1 (Cl.1) NP1-2.25 P5 D14 confluence rosiglitazone 1uM + T3 2nM + last 4 hours CL316243 10uM,,

AC,BIOTIME,GPL10588,MBA_8663,MDW-1 (Cl.1) NP1-1.32 P5 ctrl,,

AC,BIOTIME,GPL10588,MBA_8664,MDW-1 (Cl.1) NP1-1.32 P5 D14 confluence BMP4 10ng/ml + rosiglitazone 1uM + T3 2nM + last 4 hours CL316243 10uM,,

AC,BIOTIME,GPL10588,MBA_8665,MDW-1 (Cl.1) NP1-1.32 P5 D14 confluence rosiglitazone 1uM + T3 2nM + last 4 hours CL316243 10uM,,

AC,BIOTIME,GPL10588,MBA_8666,MDW-1 (Cl.1) NP1-2.34 P5 ctrl,,

AC,BIOTIME,GPL10588,MBA_8667,MDW-1 (Cl.1) NP1-2.34 P5 D14 confluence BMP4 10ng/ml + rosiglitazone 1uM + T3 2nM + last 4 hours CL316243 10uM,,

AC,BIOTIME,GPL10588,MBA_8668,MDW-1 (Cl.1) NP1-2.34 P5 D14 confluence rosiglitazone 1uM + T3 2nM + last 4 hours CL316243 10uM,,

AC,BIOTIME,GPL10588,MBA_8669,MDW-1 (Cl.1) NP1-2.37 P5 ctrl,,

AC,BIOTIME,GPL10588,MBA_8670,MDW-1 (Cl.1) NP1-2.37 P5 D14 confluence BMP4 10ng/ml + rosiglitazone 1uM + T3 2nM + last 4 hours CL316243 10uM,,

AC,BIOTIME,GPL10588,MBA_8671,MDW-1 (Cl.1) NP1-2.37 P5 D14 confluence rosiglitazone 1uM + T3 2nM + last 4 hours CL316243 10uM,,

AC,BIOTIME,GPL10588,MBA_8672,MDW-1 (Cl.1) NP1-3.25 P5 ctrl,,

AC,BIOTIME,GPL10588,MBA_8673,MDW-1 (Cl.1) NP1-3.25 P5 D14 confluence BMP4 10ng/ml + rosiglitazone 1uM + T3 2nM + last 4 hours CL316243 10uM,,

AC,BIOTIME,GPL10588,MBA_8674,MDW-1 (Cl.1) NP1-3.25 P5 D14 confluence rosiglitazone 1uM + T3 2nM + last 4 hours CL316243 10uM,,

AC,BIOTIME,GPL10588,MBA_8675,MDW-1 (Cl.1) NP1-2.26 P5 ctrl,,

AC,BIOTIME,GPL10588,MBA_8676,MDW-1 (Cl.1) NP1-2.26 P5 D14 confluence BMP4 10ng/ml + rosiglitazone 1uM + T3 2nM + last 4 hours CL316243 10uM,,

AC,BIOTIME,GPL10588,MBA_8677,MDW-1 (Cl.1) NP1-2.26 P5 D14 confluence rosiglitazone 1uM + T3 2nM + last 4 hours CL316243 10uM,,

AC,BIOTIME,GPL10588,MBA_8678,MDW-1 (Cl.1) NP1-2.12 P5 ctrl,,

AC,BIOTIME,GPL10588,MBA_8679,MDW-1 (Cl.1) NP1-2.12 P5 D14 confluence BMP4 10ng/ml + rosiglitazone 1uM + T3 2nM + last 4 hours CL316243 10uM,,

AC,BIOTIME,GPL10588,MBA_8680,MDW-1 (Cl.1) NP1-2.12 P5 D14 confluence rosiglitazone 1uM + T3 2nM + last 4 hours CL316243 10uM,,

AC,BIOTIME,GPL10588,MBA_8681,MDW-1 (Cl.1) NP1-1.29 P5 ctrl,,

AC,BIOTIME,GPL10588,MBA_8682,MDW-1 (Cl.1) NP1-1.29 P5 D14 confluence BMP4 10ng/ml + rosiglitazone 1uM + T3 2nM + last 4 hours CL316243 10uM,,

AC,BIOTIME,GPL10588,MBA_8683,MDW-1 (Cl.1) NP1-1.29 P5 D14 confluence rosiglitazone 1uM + T3 2nM + last 4 hours CL316243 10uM,,

AC,BIOTIME,GPL10588,MBA_8684,MDW-1 (Cl.1) NP1-2.41 P5 ctrl,,

AC,BIOTIME,GPL10588,MBA_8685,MDW-1 (Cl.1) NP1-2.41 P6 D14 confluence BMP4 10ng/ml + rosiglitazone 1uM + T3 2nM + last 4 hours CL316243 10uM,,

AC,BIOTIME,GPL10588,MBA_8686,MDW-1 (Cl.1) NP1-2.41 P6 D14 confluence rosiglitazone 1uM + T3 2nM + last 4 hours CL316243 10uM,,

AC,BIOTIME,GPL10588,MBA_8687,MDW-1 (Cl.1) NP1-1.15 P5 ctrl,,

AC,BIOTIME,GPL10588,MBA_8688,MDW-1 (Cl.1) NP1-1.15 P5 D14 confluence BMP4 10ng/ml + rosiglitazone 1uM + T3 2nM + last 4 hours CL316243 10uM,,

AC,BIOTIME,GPL10588,MBA_8689,MDW-1 (Cl.1) NP1-3.13 P5 ctrl,,

AC,BIOTIME,GPL10588,MBA_8690,MDW-1 (Cl.1) NP1-3.13 P5 D14 confluence BMP4 10ng/ml + rosiglitazone 1uM + T3 2nM + last 4 hours CL316243 10uM,,

AC,BIOTIME,GPL10588,MBA_8691,MDW-1 (Cl.1) NP1-1.13 P5 ctrl,,

AC,BIOTIME,GPL10588,MBA_8692,MDW-1 (Cl.1) NP1-1.13 P5 D14 confluence rosiglitazone 1uM + T3 2nM + last 4 hours CL316243 10uM,,

AC,BIOTIME,GPL10588,MBA_8693,MDW-1 (Cl.1) NP1-3.30 P6 ctrl,,

AC,BIOTIME,GPL10588,MBA_8694,MDW-1 (Cl.1) NP1-1.33 P6 ctrl,,

AC,BIOTIME,GPL10588,MBA_8695,MDW-1 (Cl.1) NP1-2.33 P6 ctrl,,

AC,BIOTIME,GPL10588,MBA_8696,MDW-1 (Cl.1) NP1-3.3 P5 ctrl,,

AC,BIOTIME,GPL10588,MBA_8697,MDW-1 (Cl.1) NP1-3.20 P5 ctrl,,

AC,BIOTIME,GPL10588,MBA_8698,MDW-1 (Cl.1) NP1-3.37 P5 ctrl,,

AC,BIOTIME,GPL10588,MBA_8699,MDW-1 (Cl.1) NP1-1.44 P5 ctrl,,

AC,BIOTIME,GPL10588,MBA_8700,MDW-1 (Cl.1) NP1-2.40 P5 ctrl,,

AC,BIOTIME,GPL10588,MBA_8701,MDW-1 (Cl.1) NP1-2.42 P5 ctrl,,

AC,BIOTIME,GPL10588,MBA_8702,MDW-1 (Cl.1) NP1-1.36 P5 ctrl,,

AC,BIOTIME,GPL10588,MBA_8703,MDW-1 (Cl.1) NP1-2.17 P5 ctrl,,

AC,BIOTIME,GPL10588,MBA_8704,MDW-1 (Cl.1) NP1-1.40 P6 ctrl,,

AC,BIOTIME,GPL10588,MBA_8705,MDW-1 (Cl.1) NP1-2.42 P6 ctrl,,

AC,BIOTIME,GPL10588,MBA_8706,MDW-1 (Cl.1) NP1-3.15 P7 ctrl,,

AC,BIOTIME,GPL10588,MBA_8707,HBVSMC (SciceCell lot# 4706) P7 ctrl (20wk fetal male),,

AC,BIOTIME,GPL10588,MBA_8708,HBVSMC (SciceCell lot# 4706) P7 ctrl,,

AC,BIOTIME,GPL10588,MBA_8709,HBVSMC (SciceCell lot# 4706) P7 D14 Hystem BMP4 10ng/ml + rosiglitazone 1uM + T3 2nM + last 4 hours CL316243 10uM,,

AC,BIOTIME,GPL10588,MBA_8710,HBVSMC (SciceCell lot# 4706) P7 D14 Hystem BMP4 10ng/ml + rosiglitazone 1uM + T3 2nM + last 4 hours CL316243 10uM,,

AC,BIOTIME,GPL10588,MBA_8711,HBVSMC (SciceCell lot# 4706) P7 D14 Hystem rosiglitazone 1uM + T3 2nM + last 4 hours CL316243 10uM,,

AC,BIOTIME,GPL10588,MBA_8712,HBVSMC (SciceCell lot# 4706) P7 D14 Hystem rosiglitazone 1uM + T3 2nM + last 4 hours CL316243 10uM,,

AC,BIOTIME,GPL10588,MBA_8713,HBVSMC (SciceCell lot# 4706) P7 D14 Hystem BMP4 10ng/ml + rosiglitazone 1uM + T3 2nM + last 2 days FGF21 50ng/ml + last 4 hours CL316243 10uM,,

AC,BIOTIME,GPL10588,MBA_8714,HBVSMC (SciceCell lot# 4706) P7 D14 Hystem BMP4 10ng/ml + rosiglitazone 1uM + T3 2nM + last 2 days FGF21 50ng/ml + last 4 hours CL316243 10uM,,

AC,BIOTIME,GPL10588,MBA_8715,HBVSMC (SciceCell lot# 4706) P7 D14 Hystem BMP4 10ng/ml + rosiglitazone 1uM + T3 2nM + last 4 hours CL316243 10uM at 28C,,

AC,BIOTIME,GPL10588,MBA_8716,HBVSMC (SciceCell lot# 4706) P7 D14 Hystem BMP4 10ng/ml + rosiglitazone 1uM + T3 2nM + last 4 hours CL316243 10uM at 28C,,

AC,BIOTIME,GPL10588,MBA_8717,HBVSMC (SciceCell lot# 4762) P7 ctrl,,

AC,BIOTIME,GPL10588,MBA_8718,HBVSMC (SciceCell lot# 4762) P7 ctrl,,

AC,BIOTIME,GPL10588,MBA_8719,HBVSMC (SciceCell lot# 4762) P5 D14 Hystem BMP4 10ng/ml + rosiglitazone 1uM + T3 2nM + last 4 hours CL316243 10uM,,

AC,BIOTIME,GPL10588,MBA_8720,HBVSMC (SciceCell lot# 4762) P5 D14 Hystem BMP4 10ng/ml + rosiglitazone 1uM + T3 2nM + last 4 hours CL316243 10uM,,

AC,BIOTIME,GPL10588,MBA_8721,HBVSMC (SciceCell lot# 4762) P5 D14 Hystem rosiglitazone 1uM + T3 2nM + last 4 hours CL316243 10uM,,

AC,BIOTIME,GPL10588,MBA_8722,HBVSMC (SciceCell lot# 4762) P5 D14 Hystem rosiglitazone 1uM + T3 2nM + last 4 hours CL316243 10uM,,

AC,BIOTIME,GPL10588,MBA_8723,HBVSMC (ScienCell lot# 4762) P5 D14 Hystem BMP4 10ng/ml + rosiglitazone 1uM + T3 2nM + last 2 days FGF21 50ng/ml + last 4 hours CL316243 10uM,,

AC,BIOTIME,GPL10588,MBA_8724,HBVSMC (ScienCell lot# 4762) P5 D14 Hystem BMP4 10ng/ml + rosiglitazone 1uM + T3 2nM + last 2 days FGF21 50ng/ml + last 4 hours CL316243 10uM,,

AC,BIOTIME,GPL10588,MBA_8725,HBVSMC (ScienCell lot# 4762) P5 D14 Hystem BMP4 10ng/ml + rosiglitazone 1uM + T3 2nM + last 4 hours CL316243 10uM at 28C,,

AC,BIOTIME,GPL10588,MBA_8726,HBVSMC (ScienCell lot# 4762) P5 D14 Hystem BMP4 10ng/ml + rosiglitazone 1uM + T3 2nM + last 4 hours CL316243 10uM at 28C,,

AC,BIOTIME,GPL10588,MBA_8727,Human skin fibroblasts ATCC CRL-1497 2.5 week neonate P5 ctrl ,,

AC,BIOTIME,GPL10588,MBA_8728,Human skin fibroblasts ATCC CRL-1497 2.5 week neonate P5 ctrl ,,

AC,BIOTIME,GPL10588,MBA_8729,Fetal Brown P9 ctrl,,

AC,BIOTIME,GPL10588,MBA_8730,Fetal Brown P9 ctrl,,

AC,BIOTIME,GPL10588,MBA_8731,PC-1 FB P6 ctrl,,

AC,BIOTIME,GPL10588,MBA_8732,PC-1 FBs P6 ctrl,,

AC,BIOTIME,GPL10588,MBA_8733,BH-2 FB P9 ctrl,,

AC,BIOTIME,GPL10588,MBA_8734,BH-2 FB P9 ctrl,,

AC,BIOTIME,GPL10588,MBA_8735,JM-1 FB P5 ctrl,,

AC,BIOTIME,GPL10588,MBA_8736,JM-1 FB P5 ctrl,,

AC,BIOTIME,GPL10588,MBA_8737,TH-1 FB P5 ctrl,,

AC,BIOTIME,GPL10588,MBA_8738,TH-1 FB P5 ctrl,,

AC,BIOTIME,GPL10588,MBA_8739,CAW-1 FB P7 ctrl,,

AC,BIOTIME,GPL10588,MBA_8740,CAW-1 FB P7 ctrl,,

AC,BIOTIME,GPL10588,MBA_8741,MDW-1 FB P6 ctrl,,

AC,BIOTIME,GPL10588,MBA_8742,MDW-1 FB P6 ctrl,,

AC,BIOTIME,GPL10588,MBA_8743,MDW-1 FB P6 D14 Hystem BMP4 10ng/ml + rosiglitazone 1uM + T3 2nM + last 4 hours CL316243 10uM,,

AC,BIOTIME,GPL10588,MBA_8744,MDW-1 FB P6 D14 Hystem BMP4 10ng/ml + rosiglitazone 1uM + T3 2nM + last 4 hours CL316243 10uM,,

AC,BIOTIME,GPL10588,MBA_8745,MDW-1 FB P6 D14 Hystem rosiglitazone 1uM + T3 2nM + last 4 hours CL316243 10uM,,

AC,BIOTIME,GPL10588,MBA_8746,MDW-1 FB P6 D14 Hystem rosiglitazone 1uM + T3 2nM + last 4 hours CL316243 10uM,,

AC,BIOTIME,GPL10588,MBA_8747,MDW-1 FB P6 D14 Hystem BMP4 10ng/ml + rosiglitazone 1uM + T3 2nM + last 2 days FGF21 50ng/ml + last 4 hours CL316243 10uM,,

AC,BIOTIME,GPL10588,MBA_8748,MDW-1 FB P6 D14 Hystem BMP4 10ng/ml + rosiglitazone 1uM + T3 2nM + last 2 days FGF21 50ng/ml + last 4 hours CL316243 10uM,,

AC,BIOTIME,GPL10588,MBA_8749,MDW-1 FB P6 D14 Hystem BMP4 10ng/ml + rosiglitazone 1uM + T3 2nM + last 4 hours CL316243 10uM at 28C,,

AC,BIOTIME,GPL10588,MBA_8750,MDW-1 FB P6 D14 Hystem BMP4 10ng/ml + rosiglitazone 1uM + T3 2nM + last 4 hours CL316243 10uM at 28C,,

AC,BIOTIME,GPL10588,MBA_8751,Limb skin FB (ABR 9726) 16 wks P2 ctrl ,,

AC,BIOTIME,GPL10588,MBA_8752,Limb skin FB (ABR 9726) 16 wks P2 ctrl,,

AC,BIOTIME,GPL10588,MBA_8753,Normal human arm skin fibroblast 48 yr (Coriell GM05879 C) P11ctrl,,

AC,BIOTIME,GPL10588,MBA_8754,Normal human arm skin fibroblast 25 yr (Coriell GM03651 G) P13 ctrl,,

AC,BIOTIME,GPL10588,MBA_8755,Normal human arm skin fibroblast 70 yr (Coriell GM01681 B) P15 ctrl,,

AC,BIOTIME,GPL10588,MBA_8756,Normal human arm skin fibroblast 50 yr (Coriell GM23251 A) P+2 ctrl,,

AC,BIOTIME,GPL10588,MBA_8757,Normal human arm skin fibroblast 50 yr (Coriell GM23251 A) P+2 ctrl,,

AC,BIOTIME,GPL10588,MBA_8758,Normal human arm skin fibroblast 44 yr (Coriell GM23249 A) P+1 ctrl,,

AC,BIOTIME,GPL10588,MBA_8759,Normal human arm skin fibroblast 71 yr (Coriell GM01680 B) P17 ctrl,,

AC,BIOTIME,GPL10588,MBA_8760,Normal human arm skin fibroblast 13 yr (Coriell GM01651 F) P14 ctrl,,

AC,BIOTIME,GPL10588,MBA_8761,Normal human arm skin fibroblast 39 yr (Coriell GM01717 A) P12,,

AC,BIOTIME,GPL10588,MBA_8762,Normal human arm skin fibroblast 39 yr (Coriell GM01717 A) P12,,

AC,BIOTIME,GPL10588,MBA_8763,Normal human arm skin fibroblast 19 yr (Coriell GM03377 E) P15,,

AC,BIOTIME,GPL10588,MBA_8764,Normal human arm skin fibroblast 19 yr (Coriell GM03377 E) P15,,

AC,BIOTIME,GPL10588,MBA_8765,Normal human arm skin fibroblast 11 yr (Coriell GM01582 B) P14,,

AC,BIOTIME,GPL10588,MBA_8766,Normal human arm skin fibroblast 11 yr (Coriell GM01582 B) P14,,

AC,BIOTIME,GPL10588,MBA_8769,Normal human arm fibroblast 37 yr (Coriell GM01650 D) P16,,

AC,BIOTIME,GPL10588,MBA_8770,Normal human arm fibroblast 37 yr (Coriell GM01650 D) P16,,

AC,BIOTIME,GPL10588,MBA_8771,JM-1 cl23 iPS P9,,

AC,BIOTIME,GPL10588,MBA_8772,JM-1 cl23 iPS P9,,

AC,BIOTIME,GPL10588,MBA_8775,HAoSMC (aortic smooth muscle PromoCell) lot 3992005 P7 ctrl,,

AC,BIOTIME,GPL10588,MBA_8776,HAoSMC (aortic smooth muscle PromoCell) lot 3992005 P7 ctrl,,

AC,BIOTIME,GPL10588,MBA_8777,HAoSMC (aortic smooth muscle PromoCell) lot 3992005 P7 D14 Hystem BMP4 10ng/ml + rosiglitazone 1uM + T3 2nM + last 4 hours CL316243 10uM,,

AC,BIOTIME,GPL10588,MBA_8778,HAoSMC (aortic smooth muscle PromoCell) lot 3992005 P7 D14 Hystem BMP4 10ng/ml + rosiglitazone 1uM + T3 2nM + last 4 hours CL316243 10uM,,

AC,BIOTIME,GPL10588,MBA_8779,HAoSMC (aortic smooth muscle PromoCell) lot 3992005 P7 D14 Hystem rosiglitazone 1uM + T3 2nM + last 4 hours CL316243 10uM,,

AC,BIOTIME,GPL10588,MBA_8780,HAoSMC (aortic smooth muscle PromoCell) lot 3992005 P7 D14 Hystem rosiglitazone 1uM + T3 2nM + last 4 hours CL316243 10uM,,

AC,BIOTIME,GPL10588,MBA_8781,HAoSMC (aortic smooth muscle PromoCell) lot 3992005 P7 D14 Hystem BMP4 10ng/ml + rosiglitazone 1uM + T3 2nM + last 2 days FGF21 50ng/ml + last 4 hours CL316243 10uM,,

AC,BIOTIME,GPL10588,MBA_8782,HAoSMC (aortic smooth muscle PromoCell) lot 3992005 P7 D14 Hystem BMP4 10ng/ml + rosiglitazone 1uM + T3 2nM + last 2 days FGF21 50ng/ml + last 4 hours CL316243 10uM,,

AC,BIOTIME,GPL10588,MBA_8783,HAoSMC (aortic smooth muscle PromoCell) lot 3992005 P7 D14 Hystem BMP4 10ng/ml + rosiglitazone 1uM + T3 2nM + last 4 hours CL316243 10uM at 28C,,

AC,BIOTIME,GPL10588,MBA_8784,HAoSMC (aortic smooth muscle PromoCell) lot 3992005 P7 D14 Hystem BMP4 10ng/ml + rosiglitazone 1uM + T3 2nM + last 4 hours CL316243 10uM at 28C,,

iPSC,BIOTIME,GPL10588,MBA_8785,30-MV2-6 P8 for active motif,,

AC,BIOTIME,GPL10588,MBA_8786,NHEK-neo ctrl P3,,

AC,BIOTIME,GPL10588,MBA_8787,HUVEC P9 ctrl (active motif related) ,,

iPSC,BIOTIME,GPL10588,MBA_8788,SM30 ReCyte,,

iPSC,BIOTIME,GPL10588,MBA_8789,SM28 ReCyte,,

AC,BIOTIME,GPL10588,MBA_8790,R14 ReCyte,,

AC,BIOTIME,GPL10588,MBA_8791,R21 ReCyte,,

AC,BIOTIME,GPL10588,MBA_8792,R26 ReCyte,,

AC,BIOTIME,GPL10588,MBA_8793,R27 ReCyte,,

iPSC,BIOTIME,GPL10588,MBA_8794,DIFF1-A1 ReCyte,,

iPSC,BIOTIME,GPL10588,MBA_8795,DIFF1-A4 ReCyte,,

iPSC,BIOTIME,GPL10588,MBA_8796,DIFF1-B4 ReCyte,,

iPSC,BIOTIME,GPL10588,MBA_8797,DIFF28C2-4B2 ReCyte,,

AC,BIOTIME,GPL10588,MBA_8798,HAoSMC (PromoCell lot 40020012.2) P8 ctrl,,

AC,BIOTIME,GPL10588,MBA_8799,HAoSMC (PromoCell lot 40020012.2) P8 ctrl,,

AC,BIOTIME,GPL10588,MBA_8800,HAoSMC (PromoCell lot 40020012.2) P8 D14 Hystem BMP4 10ng/ml + rosiglitazone 1uM + T3 2nM + last 4 hours CL316243 10uM,,

AC,BIOTIME,GPL10588,MBA_8801,HAoSMC (PromoCell lot 40020012.2) P8 D14 Hystem BMP4 10ng/ml + rosiglitazone 1uM + T3 2nM + last 4 hours CL316243 10uM,,

AC,BIOTIME,GPL10588,MBA_8802,HAoSMC (PromoCell lot 40020012.2) P8 D14 Hystem rosiglitazone 1uM + T3 2nM + last 4 hours CL316243 10uM,,

AC,BIOTIME,GPL10588,MBA_8803,HAoSMC (PromoCell lot 40020012.2) P8 D14 Hystem rosiglitazone 1uM + T3 2nM + last 4 hours CL316243 10uM,,

AC,BIOTIME,GPL10588,MBA_8804,HAoSMC (PromoCell lot 40020012.2) P8 D14 D14 Hystem BMP4 10ng/ml + rosiglitazone 1uM + T3 2nM + last 2 days FGF21 50ng/ml + last 4 hours CL316243 10uM,,

AC,BIOTIME,GPL10588,MBA_8805,HAoSMC (PromoCell lot 40020012.2) P8 D14 D14 Hystem BMP4 10ng/ml + rosiglitazone 1uM + T3 2nM + last 2 days FGF21 50ng/ml + last 4 hours CL316243 10uM,,

AC,BIOTIME,GPL10588,MBA_8806,HAoSMC (PromoCell lot 40020012.2) P8 D14 Hystem BMP4 10ng/ml + rosiglitazone 1uM + T3 2nM + last 4 hours CL316243 10uM at 28C,,

AC,BIOTIME,GPL10588,MBA_8807,HAoSMC (PromoCell lot 40020012.2) P8 D14 Hystem BMP4 10ng/ml + rosiglitazone 1uM + T3 2nM + last 4 hours CL316243 10uM at 28C,,

AC,BIOTIME,GPL10588,MBA_8808,NHBE P4 ctrl,,

AC,BIOTIME,GPL10588,MBA_8809,NHBE P4 ctrl,,

AC,BIOTIME,GPL10588,MBA_8816,HAEC (Lonza human aortic endothelial cells) P6 ctrl,,

AC,BIOTIME,GPL10588,MBA_8817,HAEC (Lonza human aortic endothelial cells) P6 ctrl,,

AC,BIOTIME,GPL10588,MBA_8820,BH-2 (cl.3) iPS P4 ,,

AC,BIOTIME,GPL10588,MBA_8821,BH-2 (cl.3) iPS P4 ,,

AC,BIOTIME,GPL10588,MBA_8822,MDW-1 iPS cl.1 P6,,

AC,BIOTIME,GPL10588,MBA_8823,MDW-1 iPS cl.1 P6,,

AC,BIOTIME,GPL10588,MBA_8824,PC-1 iPS (cl.9) P3 ,,

AC,BIOTIME,GPL10588,MBA_8825,PC-1 iPS (cl.9) P3 ,,

AC,BIOTIME,GPL10588,MBA_8826,Fetal Brown P9 D14 confluence rosiglitazone 1uM + T3 2nM + last 4 hours CL316243 10uM (active motif related),,

AC,BIOTIME,GPL10588,MBA_8827,Fetal Brown P9 D14 confluence rosiglitazone 1uM + T3 2nM + last 4 hours CL316243 10uM (active motif related),,

iPSC,BIOTIME,GPL10588,MBA_8828,W10 P12 D14 confl BMP4 10ng/ml + TGFb3 10ng/ml (active motif related error in prep for RNA need MM) ,,

iPSC,BIOTIME,GPL10588,MBA_8829,W10 P12 D14 confl BMP4 10ng/ml + TGFb3 10ng/ml (active motif related error in prep for RNA need MM) ,,

iPSC,BIOTIME,GPL10588,MBA_8830,ESI004 NP110 SM P12 D14 confluence rosiglitazone 1uM + T3 2nM + last 4 hours CL316243 10uM (active motif related),,

iPSC,BIOTIME,GPL10588,MBA_8831,ESI004 NP110 SM P12 D14 confluence rosiglitazone 1uM + T3 2nM + last 4 hours CL316243 10uM (active motif related),,

AC,BIOTIME,GPL10588,MBA_8832,MDW-1 cl1 NP2 EN50 P7 ctrl,,

AC,BIOTIME,GPL10588,MBA_8833,MDW-1 cl1 NP2 EN50 P7 D14 confluence BMP4 10ng/ml + rosiglitazone 1uM + T3 2nM + last 4 hours CL316243 10uM,,

AC,BIOTIME,GPL10588,MBA_8834,MDW-1 cl1 NP2 EN50 P7 D14 confluence rosiglitazone 1uM + T3 2nM + last 4 hours CL316243 10uM,,

AC,BIOTIME,GPL10588,MBA_8835,MDW-1 cl1NP2 EN50 P7 D14 confluence BMP4 10ng/ml,,

AC,BIOTIME,GPL10588,MBA_8836,MDW1-1 cl1 NP2 EN89 P6 ctrl,,

AC,BIOTIME,GPL10588,MBA_8837,MDW-1 cl1 NP2 EN89 P6 D14 confluence BMP4 10ng/ml + rosiglitazone 1uM + T3 2nM + last 4 hours CL316243 10uM,,

AC,BIOTIME,GPL10588,MBA_8838,MDW-1 cl1 NP2 EN89 P6 D14 confluence rosiglitazone 1uM + T3 2nM + last 4 hours CL316243 10uM,,

AC,BIOTIME,GPL10588,MBA_8839,MDW-1 cl 1 NP2 EN89 P6 D14 confluence BMP4 10ng/ml ,,

AC,BIOTIME,GPL10588,MBA_40_2,NHOST Normal human osteoblasts Lonza,,

iPSC,BIOTIME,GPL10588,MBA_7483_2, re-prepped at U Minn from plate_109 C4ELSR2 P11 D14 Hystem BMP4 50ng/ml + Rosi T3 (Liothyronine 2nM) + last 4 hours CL316243 10uM ,,

iPSC,BIOTIME,GPL10588,MBA_7529_2,E75 P14 D14 Hystem BMP4 10ng/ml + rosiglitazone 1uM + T3 2nM + last 4 hrs CL316243 10uM,,

iPSC,BIOTIME,GPL10588,MBA_7722_2,ESI004 NPCC SM19 P5 ctrl,,

iPSC,BIOTIME,GPL10588,MBA_7726_2,ESI004 NPCC SM31 P5 ctrl,,

iPSC,BIOTIME,GPL10588,MBA_7727_2,ESI004 NPCC SM31 P5 D14 Hystem BMP4 10ng/ml + rosiglitazone 1uM + T3 2nM + last 4 hours CL316243 10uM,,

iPSC,BIOTIME,GPL10588,MBA_7730_2,ESI004 NPCC SM36 P5 ctrl,,

iPSC,BIOTIME,GPL10588,MBA_7731_2,ESI004 NPCC SM36 P5 D14 Hystem BMP4 10ng/ml + rosiglitazone 1uM + T3 2nM + last 4 hours CL316243 10uM,,

iPSC,BIOTIME,GPL10588,MBA_7734_2,ESI004 NPCC SM28 P5 ctrl,,

iPSC,BIOTIME,GPL10588,MBA_7735_2,ESI004 NPCC SM28 P5 D14 Hystem BMP4 10ng/ml + rosiglitazone 1uM + T3 2nM + last 4 hours CL316243 10uM,,

iPSC,BIOTIME,GPL10588,MBA_7737_2,ESI004 NPCC SM40 P5 ctrl,,

iPSC,BIOTIME,GPL10588,MBA_7738_2,ESI004 NPCC SM40 P5 D14 Hystem BMP4 10ng/ml + rosiglitazone 1uM + T3 2nM + last 4 hours CL316243 10uM,,

iPSC,BIOTIME,GPL10588,MBA_7933_2,RP1-SM2-21 P10 D14 Hystem BMP4 10ng/ml + rosiglitazone 1uM + T3 2nM + last 4 hours CL316243 10uM,,

iPSC,BIOTIME,GPL10588,MBA_7936_2,RP1-SKEL-19 P9 ctrl,,

iPSC,BIOTIME,GPL10588,MBA_7937_2,RP1-SKEL-19 P9 D14 Hystem BMP4 10ng/ml + rosiglitazone 1uM + T3 2nM + last 4 hours CL316243 10uM,,

iPSC,BIOTIME,GPL10588,MBA_7942_2,RP1-SKEL-20 P10 D14 Hystem BMP4 10ng/ml + rosiglitazone 1uM + T3 2nM + last 4 hours CL316243 10uM,,

iPSC,BIOTIME,GPL10588,MBA_7946_2,RP1-SKEL-23 P9 D14 Hystem BMP4 10ng/ml + rosiglitazone 1uM + T3 2nM + last 4 hours CL316243 10uM,,

iPSC,BIOTIME,GPL10588,MBA_7949_2,RP1-SKEL-6 P10,,

iPSC,BIOTIME,GPL10588,MBA_7950_2,RP1-SKEL-6 P10 D14 Hystem BMP4 10ng/ml + rosiglitazone 1uM + T3 2nM + last 4 hours CL316243 10uM,,

iPSC,BIOTIME,GPL10588,MBA_7953_2,RP1-DM10-19 P10 ctrl,,

iPSC,BIOTIME,GPL10588,MBA_7954_2,RP1-DM10-19 P10 D14 Hystem BMP4 10ng/ml + rosiglitazone 1uM + T3 2nM + last 4 hours CL316243 10uM,,

iPSC,BIOTIME,GPL10588,MBA_7958_2,RP1-SKEL-8 P10 ctrl,,

iPSC,BIOTIME,GPL10588,MBA_7959_2,RP1-SKEL-8 P10 D14 Hystem BMP4 10ng/ml + rosiglitazone 1uM + T3 2nM + last 4 hours CL316243 10uM,,

iPSC,BIOTIME,GPL10588,MBA_7962_2,RP1-SKEL-16 P10 ctrl,,

iPSC,BIOTIME,GPL10588,MBA_7963_2,RP1-SKEL-16 P10 D14 Hystem BMP4 10ng/ml + rosiglitazone 1uM + T3 2nM + last 4 hours CL316243 10uM,,

iPSC,BIOTIME,GPL10588,MBA_7967_2,RP1-MV2-16 P16 (plastic) D14 Hystem BMP4 10ng/ml + rosiglitazone 1uM + T3 2nM + last 4 hours CL316243 10uM,,

iPSC,BIOTIME,GPL10588,MBA_8004_2,ESI004 NPCC SM29 P6 ctrl,,

iPSC,BIOTIME,GPL10588,MBA_8005_2,ESI004 NPCC SM29 P6 D14 Hystem BMP4 10ng/ml + rosiglitazone 1uM + T3 2nM + last 4 hours CL316243 10uM,,

iPSC,BIOTIME,GPL10588,MBA_8008_2,ESI004 NPCC SM46 P7 ctrl,,

iPSC,BIOTIME,GPL10588,MBA_8009_2,ESI004 NPCC SM46 P8 D14 Hystem BMP4 10ng/ml + rosiglitazone 1uM + T3 2nM + last 4 hours CL316243 10uM,,

iPSC,BIOTIME,GPL10588,MBA_8013_2,ESI004 NPCC SM23 P8 ctrl,,

iPSC,BIOTIME,GPL10588,MBA_8014_2,ESI004 NPCC SM23 P8 D14 Hystem BMP4 10ng/ml + rosiglitazone 1uM + T3 2nM + last 4 hours CL316243 10uM,,

iPSC,BIOTIME,GPL10588,MBA_8016_2,ESI004 NPCC SM45 P6 ctrl,,

iPSC,BIOTIME,GPL10588,MBA_8017_2,ESI004 NPCC SM45 P6 D14 Hystem BMP4 10ng/ml + rosiglitazone 1uM + T3 2nM + last 4 hours CL316243 10uM,,

iPSC,BIOTIME,GPL10588,MBA_8019_2,30-SKEL-6 P8 ctrl,,

iPSC,BIOTIME,GPL10588,MBA_8020_2,30-SKEL-6 P8 D14 Hystem BMP4 10ng/ml + rosiglitazone 1uM T3 2nM + last 4 hours CL316243 10uM,,

iPSC,BIOTIME,GPL10588,MBA_8123_2,SK1 P15 ctrl,,

iPSC,BIOTIME,GPL10588,MBA_8138_2,SK11 P16 D14 Hystem BMP4 10ng/ml + rosiglitazone 1uM + T3 2nM + last 4 hours CL316243 10uM,,

ASC,E-MEXP-3289,A-MEXP-2072,E-MEXP-3289_1,DMSO20 adipo 1 week_2,,

ASC,E-MEXP-3289,A-MEXP-2072,E-MEXP-3289_13,TPV20 adipo 1 week,,

ASC,E-MEXP-3289,A-MEXP-2072,E-MEXP-3289_3,TPV20 adipo 1 week_2,,

ASC,E-MEXP-3289,A-MEXP-2072,E-MEXP-3289_5,TPV20 hMSC_2,,

ASC,E-MEXP-3289,A-MEXP-2072,E-MEXP-3289_2,DMSO20 hMSC_2,,

ASC,E-MEXP-3289,A-MEXP-2072,E-MEXP-3289_12,DMSO20 hMSC,,

ASC,E-MEXP-3289,A-MEXP-2072,E-MEXP-3289_6,TPV20 hMSC_2,,

ASC,E-MEXP-3289,A-MEXP-2072,E-MEXP-3289_15,TPV20 hMSC,,

IPSC,E-MTAB-1040,A-MEXP-2072,E-MTAB-1040_24,GU9569i-cont2_P6+13,,

IPSC,E-MTAB-1040,A-MEXP-2072,E-MTAB-1040_26,GU9572i-cont2_P9+12,,

IPSC,E-MTAB-1040,A-MEXP-2072,E-MTAB-1040_21,GU9565i-cont1_P8+8,,

IPSC,E-MTAB-1040,A-MEXP-2072,E-MTAB-1040_28,GU9569i-cont1_p5+12&17,,

IPSC,E-MTAB-1040,A-MEXP-2072,E-MTAB-1040_25,GU9572i-cont1_P9+20,,

IPSC,E-MTAB-1040,A-MEXP-2072,E-MTAB-1040_22,GU9565i-cont2_P7+11,,

IPSC,E-MTAB-1040,A-MEXP-2072,E-MTAB-1040_20,GU9563i-cont1_P12+16,,

IPSC,E-MTAB-1040,A-MEXP-2072,E-MTAB-1040_23,GU9565i-cont2_P7+15,,

IPSC,E-MTAB-1040,A-MEXP-2072,E-MTAB-1040_27,GU9572i-cont3_P3+16,,

ESC,E-MTAB-1647,GPL6947,E-MTAB-1647_8,H1 on Matrigel (2),,

ESC,E-MTAB-1647,GPL6947,E-MTAB-1647_4,H1 on 1112BM (2),,

ESC,E-MTAB-1647,GPL6947,E-MTAB-1647_0,H1 on 293BM (1),,

ESC,E-MTAB-1647,GPL6947,E-MTAB-1647_7,H1 on Matrigel (1),,

ESC,E-MTAB-1647,GPL6947,E-MTAB-1647_3,H1 on 1112BM (1),,

ESC,E-MTAB-1647,GPL6947,E-MTAB-1647_2,H1 on 293BM (3),,

ESC,E-MTAB-1647,GPL6947,E-MTAB-1647_9,H1 on Matrigel (3),,

ESC,E-MTAB-1647,GPL6947,E-MTAB-1647_5,H1 on 1112BM (3),,

ESC,E-MTAB-1647,GPL6947,E-MTAB-1647_6,H1 on 1112BM (4),,

ESC,E-MTAB-1647,GPL6947,E-MTAB-1647_1,H1 on 293BM (2),,

ESC,E-MTAB-1647,GPL6947,E-MTAB-1647_10,H1 on Matrigel (4),,

ESC,E-MTAB-1653,GPL6947,E-MTAB-1653_26,Undiff-G3-3,,

ESC,E-MTAB-1653,GPL6947,E-MTAB-1653_3,Undiff-G4-1,,

ESC,E-MTAB-1653,GPL6947,E-MTAB-1653_53,T24-WCE-2,,

ESC,E-MTAB-1653,GPL6947,E-MTAB-1653_15,Undiff-G4-2,,

ESC,E-MTAB-1653,GPL6947,E-MTAB-1653_41,TE12-G2-4,,

ESC,E-MTAB-1653,GPL6947,E-MTAB-1653_5,TE12-G2-1,,

ESC,E-MTAB-1653,GPL6947,E-MTAB-1653_38,Undiff-G3-4,,

ESC,E-MTAB-1653,GPL6947,E-MTAB-1653_39,Undiff-G4-4,,

ESC,E-MTAB-1653,GPL6947,E-MTAB-1653_14,Undiff-G3-2,,

ESC,E-MTAB-1653,GPL6947,E-MTAB-1653_2,Undiff-G3-1,,

ESC,E-MTAB-1653,GPL6947,E-MTAB-1653_29,TE12-G2-3,,

ESC,E-MTAB-1653,GPL6947,E-MTAB-1653_59,T24-WCE-4,,

ESC,E-MTAB-1653,GPL6947,E-MTAB-1653_28,TE12-G1-2,,

ESC,E-MTAB-1653,GPL6947,E-MTAB-1653_40,TE12-G1-4,,

ESC,E-MTAB-1653,GPL6947,E-MTAB-1653_4,TE12-G1-1,,

ESC,E-MTAB-1653,GPL6947,E-MTAB-1653_27,Undiff-G4-3,,

ESC,E-MTAB-1653,GPL6947,E-MTAB-1653_56,T24-WCE-3,,

ESC,E-MTAB-1653,GPL6947,E-MTAB-1653_16,TE12-G1-3,,

ESC,E-MTAB-1653,GPL6947,E-MTAB-1653_17,TE12-G2-2,,

ESC,E-MTAB-1653,GPL6947,E-MTAB-1653_50,T24-WCE-1,,

ASC,E-MTAB-3576,A-MEXP-2072,E-MTAB-3576_2,CD34+ CTRL_c,,

ESC,E-MTAB-3825,A-MEXP-2072,E-MTAB-3825_3,H9_2,,

ESC,E-MTAB-3825,A-MEXP-2072,E-MTAB-3825_1,FES22_EB,,

ESC,E-MTAB-3825,A-MEXP-2072,E-MTAB-3825_2,H9_1,,

ESC,E-MTAB-3825,A-MEXP-2072,E-MTAB-3825_4,H9_EB,,

ESC,E-MTAB-3825,A-MEXP-2072,E-MTAB-3825_0,FES22,,

IPSC,E-MTAB-3825,A-MEXP-2072,E-MTAB-3825_11,T14F_EB_2,,

IPSC,E-MTAB-3825,A-MEXP-2072,E-MTAB-3825_45,T55F_iPS_1,,

IPSC,E-MTAB-3825,A-MEXP-2072,E-MTAB-3825_12,T14F_iPS_1,,

IPSC,E-MTAB-3825,A-MEXP-2072,E-MTAB-3825_47,T55F_iPS_2,,

IPSC,E-MTAB-3825,A-MEXP-2072,E-MTAB-3825_36,T53F_iPS_1L,,

IPSC,E-MTAB-3825,A-MEXP-2072,E-MTAB-3825_13,T14F_iPS_1L,,

IPSC,E-MTAB-3825,A-MEXP-2072,E-MTAB-3825_26,T42F_iPS_2L,,

IPSC,E-MTAB-3825,A-MEXP-2072,E-MTAB-3825_35,T53F_iPS_1,,

IPSC,E-MTAB-3825,A-MEXP-2072,E-MTAB-3825_24,T42F_iPS_1,,

IPSC,E-MTAB-3825,A-MEXP-2072,E-MTAB-3825_37,T53F_iPS_2,,

IPSC,E-MTAB-3825,A-MEXP-2072,E-MTAB-3825_14,T14F_iPS_2,,

IPSC,E-MTAB-3825,A-MEXP-2072,E-MTAB-3825_44,T55F_EB_2,,

IPSC,E-MTAB-3825,A-MEXP-2072,E-MTAB-3825_23,T42F_EB_1,,

IPSC,E-MTAB-3825,A-MEXP-2072,E-MTAB-3825_46,T55F_iPS_1L,,

IPSC,E-MTAB-3825,A-MEXP-2072,E-MTAB-3825_34,T53F_EB_2,,

IPSC,E-MTAB-3825,A-MEXP-2072,E-MTAB-3825_25,T42F_iPS_2,,

IPSC,E-MTAB-4057,A-MEXP-2072,E-MTAB-4057_129,HPSI1113i-qolg_1,,

IPSC,E-MTAB-4057,A-MEXP-2072,E-MTAB-4057_127,HPSI0114i-posc_1,,

IPSC,E-MTAB-4057,A-MEXP-2072,E-MTAB-4057_95,HPSI1013i-jufd_1,,

IPSC,E-MTAB-4057,A-MEXP-2072,E-MTAB-4057_23,HPSI0314i-hoik_1,,

IPSC,E-MTAB-4057,A-MEXP-2072,E-MTAB-4057_60,HPSI1113i-dons_3,,

IPSC,E-MTAB-4057,A-MEXP-2072,E-MTAB-4057_84,HPSI0114i-iisa_2,,

IPSC,E-MTAB-4057,A-MEXP-2072,E-MTAB-4057_107,HPSI1213i-nusw_2,,

IPSC,E-MTAB-4057,A-MEXP-2072,E-MTAB-4057_72,HPSI1113i-hayt_3,,

IPSC,E-MTAB-4057,A-MEXP-2072,E-MTAB-4057_133,HPSI1113i-qorq_2,,

IPSC,E-MTAB-4057,A-MEXP-2072,E-MTAB-4057_55,HPSI0913i-diku_2,,

IPSC,E-MTAB-4057,A-MEXP-2072,E-MTAB-4057_122,HPSI1013i-pamv_1,,

IPSC,E-MTAB-4057,A-MEXP-2072,E-MTAB-4057_109,HPSI1213i-nusw_3,,

IPSC,E-MTAB-4057,A-MEXP-2072,E-MTAB-4057_96,HPSI0114i-kolf_2,,

IPSC,E-MTAB-4057,A-MEXP-2072,E-MTAB-4057_62,HPSI0913i-eika_1,,

IPSC,E-MTAB-4057,A-MEXP-2072,E-MTAB-4057_88,HPSI1013i-jogf_2,,

IPSC,E-MTAB-4057,A-MEXP-2072,E-MTAB-4057_112,HPSI1113i-oaaz_2,,

IPSC,E-MTAB-4057,A-MEXP-2072,E-MTAB-4057_9,HPSI0214i-feec_2,,

IPSC,E-MTAB-4057,A-MEXP-2072,E-MTAB-4057_118,HPSI1213i-pahc_6,,

IPSC,E-MTAB-4057,A-MEXP-2072,E-MTAB-4057_37,HPSI0114i-vass_1,,

IPSC,E-MTAB-4057,A-MEXP-2072,E-MTAB-4057_106,HPSI1213i-nekd_2,,

IPSC,E-MTAB-4057,A-MEXP-2072,E-MTAB-4057_22,HPSI0314i-hoik_3,,

IPSC,E-MTAB-4057,A-MEXP-2072,E-MTAB-4057_20,HPSI0214i-heth_1,,

IPSC,E-MTAB-4057,A-MEXP-2072,E-MTAB-4057_155,HPSI1013i-wopl_1,,

IPSC,E-MTAB-4057,A-MEXP-2072,E-MTAB-4057_147,HPSI1113i-wetu_1,,

IPSC,E-MTAB-4057,A-MEXP-2072,E-MTAB-4057_79,HPSI1013i-hiaf_1,,

IPSC,E-MTAB-4057,A-MEXP-2072,E-MTAB-4057_49,HPSI1113i-bima_2,,

IPSC,E-MTAB-4057,A-MEXP-2072,E-MTAB-4057_156,HPSI1013i-wuye_1,,

IPSC,E-MTAB-4057,A-MEXP-2072,E-MTAB-4057_66,HPSI1113i-eofe_3,,

IPSC,E-MTAB-4057,A-MEXP-2072,E-MTAB-4057_135,HPSI0114i-rozh_3,,

IPSC,E-MTAB-4057,A-MEXP-2072,E-MTAB-4057_120,HPSI1013i-pamv_2,,

IPSC,E-MTAB-4057,A-MEXP-2072,E-MTAB-4057_140,HPSI0314i-sojd_2,,

IPSC,E-MTAB-4057,A-MEXP-2072,E-MTAB-4057_81,HPSI1113i-ieki_2,,

IPSC,E-MTAB-4057,A-MEXP-2072,E-MTAB-4057_164,HPSI0114i-zapk_2,,

IPSC,E-MTAB-4057,A-MEXP-2072,E-MTAB-4057_3,HPSI0214i-bute_2,,

IPSC,E-MTAB-4057,A-MEXP-2072,E-MTAB-4057_142,HPSI1113i-uofv_1,,

IPSC,E-MTAB-4057,A-MEXP-2072,E-MTAB-4057_2,HPSI0314i-bubh_3,,

IPSC,E-MTAB-4057,A-MEXP-2072,E-MTAB-4057_29,HPSI0214i-pelm_2,,

IPSC,E-MTAB-4057,A-MEXP-2072,E-MTAB-4057_137,HPSI0114i-rozh_4,,

IPSC,E-MTAB-4057,A-MEXP-2072,E-MTAB-4057_98,HPSI0114i-kolf_3,,

IPSC,E-MTAB-4057,A-MEXP-2072,E-MTAB-4057_24,HPSI0214i-kehc_2,,

IPSC,E-MTAB-4057,A-MEXP-2072,E-MTAB-4057_123,HPSI1113i-podx_2,,

IPSC,E-MTAB-4057,A-MEXP-2072,E-MTAB-4057_13,HPSI0214i-fiau_3,,

IPSC,E-MTAB-4057,A-MEXP-2072,E-MTAB-4057_38,HPSI0214i-wibj_1,,

IPSC,E-MTAB-4057,A-MEXP-2072,E-MTAB-4057_134,HPSI1113i-qorq_1,,

IPSC,E-MTAB-4057,A-MEXP-2072,E-MTAB-4057_28,HPSI0214i-pelm_3,,

IPSC,E-MTAB-4057,A-MEXP-2072,E-MTAB-4057_160,HPSI1213i-xuja_3,,

IPSC,E-MTAB-4057,A-MEXP-2072,E-MTAB-4057_31,HPSI0314i-qaqx_1,,

IPSC,E-MTAB-4057,A-MEXP-2072,E-MTAB-4057_65,HPSI0114i-eipl_1,,

IPSC,E-MTAB-4057,A-MEXP-2072,E-MTAB-4057_19,HPSI0214i-heja_2,,

IPSC,E-MTAB-4057,A-MEXP-2072,E-MTAB-4057_117,HPSI1213i-pahc_5,,

IPSC,E-MTAB-4057,A-MEXP-2072,E-MTAB-4057_150,HPSI1013i-woci_3,,

IPSC,E-MTAB-4057,A-MEXP-2072,E-MTAB-4057_136,HPSI0114i-rozh_5,,

IPSC,E-MTAB-4057,A-MEXP-2072,E-MTAB-4057_116,HPSI0114i-oevr_3,,

IPSC,E-MTAB-4057,A-MEXP-2072,E-MTAB-4057_100,HPSI0114i-lexy_2,,

IPSC,E-MTAB-4057,A-MEXP-2072,E-MTAB-4057_46,HPSI1213i-babk_3,,

IPSC,E-MTAB-4057,A-MEXP-2072,E-MTAB-4057_80,HPSI1013i-hiaf_2,,

IPSC,E-MTAB-4057,A-MEXP-2072,E-MTAB-4057_145,HPSI0114i-vabj_2,,

IPSC,E-MTAB-4057,A-MEXP-2072,E-MTAB-4057_128,HPSI0114i-posc_2,,

IPSC,E-MTAB-4057,A-MEXP-2072,E-MTAB-4057_21,HPSI0314i-hoik_2,,

IPSC,E-MTAB-4057,A-MEXP-2072,E-MTAB-4057_113,HPSI0913i-oapg_4,,

IPSC,E-MTAB-4057,A-MEXP-2072,E-MTAB-4057_4,HPSI0314i-cuhk_2,,

IPSC,E-MTAB-4057,A-MEXP-2072,E-MTAB-4057_32,HPSI0314i-qonc_1,,

IPSC,E-MTAB-4057,A-MEXP-2072,E-MTAB-4057_67,HPSI1113i-eofe_1,,

IPSC,E-MTAB-4057,A-MEXP-2072,E-MTAB-4057_149,HPSI1113i-wetu_2,,

IPSC,E-MTAB-4057,A-MEXP-2072,E-MTAB-4057_26,HPSI0913i-lise_3,,

IPSC,E-MTAB-4057,A-MEXP-2072,E-MTAB-4057_16,HPSI0114i-fikt_1,,

IPSC,E-MTAB-4057,A-MEXP-2072,E-MTAB-4057_73,HPSI1113i-hayt_1,,

IPSC,E-MTAB-4057,A-MEXP-2072,E-MTAB-4057_7,HPSI0214i-eiwy_1,,

IPSC,E-MTAB-4057,A-MEXP-2072,E-MTAB-4057_97,HPSI0114i-kolf_1,,

IPSC,E-MTAB-4057,A-MEXP-2072,E-MTAB-4057_64,HPSI0114i-eipl_2,,

IPSC,E-MTAB-4057,A-MEXP-2072,E-MTAB-4057_11,HPSI0214i-fiau_1,,

IPSC,E-MTAB-4057,A-MEXP-2072,E-MTAB-4057_59,HPSI1113i-dons_2,,

IPSC,E-MTAB-4057,A-MEXP-2072,E-MTAB-4057_33,HPSI0314i-qonc_2,,

IPSC,E-MTAB-4057,A-MEXP-2072,E-MTAB-4057_10,HPSI0214i-feec_3,,

IPSC,E-MTAB-4057,A-MEXP-2072,E-MTAB-4057_146,HPSI0114i-vabj_1,,

IPSC,E-MTAB-4057,A-MEXP-2072,E-MTAB-4057_161,HPSI1213i-xuja_1,,

IPSC,E-MTAB-4057,A-MEXP-2072,E-MTAB-4057_34,HPSI0214i-rayr_1,,

IPSC,E-MTAB-4057,A-MEXP-2072,E-MTAB-4057_40,HPSI0314i-xugn_1,,

IPSC,E-MTAB-4057,A-MEXP-2072,E-MTAB-4057_47,HPSI0114i-bezi_3,,

IPSC,E-MTAB-4057,A-MEXP-2072,E-MTAB-4057_82,HPSI1113i-ieki_1,,

IPSC,E-MTAB-4057,A-MEXP-2072,E-MTAB-4057_115,HPSI0114i-oevr_2,,

IPSC,E-MTAB-4057,A-MEXP-2072,E-MTAB-4057_50,HPSI1113i-bima_3,,

IPSC,E-MTAB-4057,A-MEXP-2072,E-MTAB-4057_131,HPSI1113i-qolg_3,,

IPSC,E-MTAB-4057,A-MEXP-2072,E-MTAB-4057_85,HPSI0114i-iisa_3,,

IPSC,E-MTAB-4057,A-MEXP-2072,E-MTAB-4057_91,HPSI0114i-joxm_1,,

IPSC,E-MTAB-4057,A-MEXP-2072,E-MTAB-4057_144,HPSI0114i-vabj_3,,

IPSC,E-MTAB-4057,A-MEXP-2072,E-MTAB-4057_125,HPSI1113i-podx_1,,

IPSC,E-MTAB-4057,A-MEXP-2072,E-MTAB-4057_70,HPSI1013i-garx_1,,

IPSC,E-MTAB-4057,A-MEXP-2072,E-MTAB-4057_165,HPSI0114i-zapk_3,,

IPSC,E-MTAB-4057,A-MEXP-2072,E-MTAB-4057_74,HPSI1113i-hayt_2,,

IPSC,E-MTAB-4057,A-MEXP-2072,E-MTAB-4057_75,HPSI1213i-hehd_2,,

IPSC,E-MTAB-4057,A-MEXP-2072,E-MTAB-4057_41,HPSI1013i-yemz_3,,

IPSC,E-MTAB-4057,A-MEXP-2072,E-MTAB-4057_101,HPSI0114i-lexy_3,,

IPSC,E-MTAB-4057,A-MEXP-2072,E-MTAB-4057_78,HPSI1013i-hiaf_3,,

IPSC,E-MTAB-4057,A-MEXP-2072,E-MTAB-4057_56,HPSI0913i-diku_1,,

IPSC,E-MTAB-4057,A-MEXP-2072,E-MTAB-4057_138,HPSI1013i-sebz_3,,

IPSC,E-MTAB-4057,A-MEXP-2072,E-MTAB-4057_0,HPSI0314i-bubh_2,,

IPSC,E-MTAB-4057,A-MEXP-2072,E-MTAB-4057_102,HPSI0114i-lexy_1,,

IPSC,E-MTAB-4057,A-MEXP-2072,E-MTAB-4057_139,HPSI1013i-sebz_1,,

IPSC,E-MTAB-4057,A-MEXP-2072,E-MTAB-4057_68,HPSI1113i-eofe_2,,

IPSC,E-MTAB-4057,A-MEXP-2072,E-MTAB-4057_58,HPSI1113i-dons_1,,

IPSC,E-MTAB-4057,A-MEXP-2072,E-MTAB-4057_8,HPSI0314i-fafq_1,,

IPSC,E-MTAB-4057,A-MEXP-2072,E-MTAB-4057_42,HPSI1013i-yemz_2,,

IPSC,E-MTAB-4057,A-MEXP-2072,E-MTAB-4057_69,HPSI1013i-garx_3,,

IPSC,E-MTAB-4057,A-MEXP-2072,E-MTAB-4057_103,HPSI0913i-lise_2,,

IPSC,E-MTAB-4057,A-MEXP-2072,E-MTAB-4057_167,HPSI0114i-zoxy_1,,

IPSC,E-MTAB-4057,A-MEXP-2072,E-MTAB-4057_89,HPSI1013i-jogf_3,,

IPSC,E-MTAB-4057,A-MEXP-2072,E-MTAB-4057_94,HPSI1013i-jufd_2,,

IPSC,E-MTAB-4057,A-MEXP-2072,E-MTAB-4057_1,HPSI0314i-bubh_1,,

IPSC,E-MTAB-4057,A-MEXP-2072,E-MTAB-4057_51,HPSI1113i-bima_1,,

IPSC,E-MTAB-4057,A-MEXP-2072,E-MTAB-4057_153,HPSI1013i-wopl_2,,

IPSC,E-MTAB-4057,A-MEXP-2072,E-MTAB-4057_45,HPSI1213i-babk_1,,

IPSC,E-MTAB-4057,A-MEXP-2072,E-MTAB-4057_15,HPSI0114i-fikt_3,,

IPSC,E-MTAB-4057,A-MEXP-2072,E-MTAB-4057_148,HPSI1113i-wetu_3,,

IPSC,E-MTAB-4057,A-MEXP-2072,E-MTAB-4057_17,HPSI0214i-heja_3,,

IPSC,E-MTAB-4057,A-MEXP-2072,E-MTAB-4057_53,HPSI1013i-cups_3,,

IPSC,E-MTAB-4057,A-MEXP-2072,E-MTAB-4057_152,HPSI1013i-woci_1,,

IPSC,E-MTAB-4057,A-MEXP-2072,E-MTAB-4057_5,HPSI0314i-cuhk_1,,

IPSC,E-MTAB-4057,A-MEXP-2072,E-MTAB-4057_124,HPSI1113i-podx_3,,

IPSC,E-MTAB-4057,A-MEXP-2072,E-MTAB-4057_76,HPSI1213i-hehd_3,,

IPSC,E-MTAB-4057,A-MEXP-2072,E-MTAB-4057_48,HPSI0114i-bezi_1,,

IPSC,E-MTAB-4057,A-MEXP-2072,E-MTAB-4057_99,HPSI1013i-kuxp_1,,

IPSC,E-MTAB-4057,A-MEXP-2072,E-MTAB-4057_126,HPSI0114i-posc_3,,

IPSC,E-MTAB-4057,A-MEXP-2072,E-MTAB-4057_93,HPSI1013i-jufd_3,,

IPSC,E-MTAB-4057,A-MEXP-2072,E-MTAB-4057_141,HPSI1113i-uofv_2,,

IPSC,E-MTAB-4057,A-MEXP-2072,E-MTAB-4057_104,HPSI1213i-nekd_3,,

IPSC,E-MTAB-4057,A-MEXP-2072,E-MTAB-4057_111,HPSI1113i-oaaz_3,,

IPSC,E-MTAB-4057,A-MEXP-2072,E-MTAB-4057_121,HPSI1013i-pamv_3,,

IPSC,E-MTAB-4057,A-MEXP-2072,E-MTAB-4057_27,HPSI0913i-lise_1,,

IPSC,E-MTAB-4057,A-MEXP-2072,E-MTAB-4057_163,HPSI0114i-zapk_1,,

IPSC,E-MTAB-4057,A-MEXP-2072,E-MTAB-4057_52,HPSI1013i-cups_1,,

IPSC,E-MTAB-4057,A-MEXP-2072,E-MTAB-4057_43,HPSI1013i-yemz_1,,

IPSC,E-MTAB-4057,A-MEXP-2072,E-MTAB-4057_30,HPSI0214i-pelm_1,,

IPSC,E-MTAB-4057,A-MEXP-2072,E-MTAB-4057_132,HPSI1113i-qorq_3,,

IPSC,E-MTAB-4057,A-MEXP-2072,E-MTAB-4057_71,HPSI1013i-garx_2,,

IPSC,E-MTAB-4057,A-MEXP-2072,E-MTAB-4057_35,HPSI0314i-sojd_3,,

IPSC,E-MTAB-4057,A-MEXP-2072,E-MTAB-4057_108,HPSI1213i-nusw_1,,

IPSC,E-MTAB-4057,A-MEXP-2072,E-MTAB-4057_158,HPSI1013i-wuye_3,,

IPSC,E-MTAB-4057,A-MEXP-2072,E-MTAB-4057_14,HPSI0114i-fikt_2,,

IPSC,E-MTAB-4057,A-MEXP-2072,E-MTAB-4057_61,HPSI0913i-eika_2,,

IPSC,E-MTAB-4057,A-MEXP-2072,E-MTAB-4057_105,HPSI1213i-nekd_1,,

IPSC,E-MTAB-4057,A-MEXP-2072,E-MTAB-4057_168,HPSI0114i-zoxy_3,,

IPSC,E-MTAB-4057,A-MEXP-2072,E-MTAB-4057_151,HPSI1013i-woci_2,,

IPSC,E-MTAB-4057,A-MEXP-2072,E-MTAB-4057_159,HPSI0314i-xugn_2,,

IPSC,E-MTAB-4057,A-MEXP-2072,E-MTAB-4057_130,HPSI1113i-qolg_2,,

IPSC,E-MTAB-4057,A-MEXP-2072,E-MTAB-4057_92,HPSI0114i-joxm_2,,

IPSC,E-MTAB-4057,A-MEXP-2072,E-MTAB-4057_154,HPSI1013i-wopl_3,,

IPSC,E-MTAB-4057,A-MEXP-2072,E-MTAB-4057_54,HPSI1013i-cups_2,,

IPSC,E-MTAB-4057,A-MEXP-2072,E-MTAB-4057_110,HPSI1113i-oaaz_1,,

IPSC,E-MTAB-4057,A-MEXP-2072,E-MTAB-4057_162,HPSI1213i-xuja_2,,

IPSC,E-MTAB-4057,A-MEXP-2072,E-MTAB-4057_18,HPSI0214i-heja_1,,

IPSC,E-MTAB-4057,A-MEXP-2072,E-MTAB-4057_157,HPSI1013i-wuye_2,,

IPSC,E-MTAB-4057,A-MEXP-2072,E-MTAB-4057_83,HPSI1113i-ieki_3,,

IPSC,E-MTAB-4057,A-MEXP-2072,E-MTAB-4057_6,HPSI0314i-cuhk_3,,

IPSC,E-MTAB-4057,A-MEXP-2072,E-MTAB-4057_39,HPSI0214i-wibj_2,,

IPSC,E-MTAB-4057,A-MEXP-2072,E-MTAB-4057_12,HPSI0214i-fiau_2,,

IPSC,E-MTAB-4057,A-MEXP-2072,E-MTAB-4057_114,HPSI0913i-oapg_5,,

IPSC,E-MTAB-4057,A-MEXP-2072,E-MTAB-4057_77,HPSI1213i-hehd_1,,

IPSC,E-MTAB-4057,A-MEXP-2072,E-MTAB-4057_143,HPSI1113i-uofv_3,,

IPSC,E-MTAB-4057,A-MEXP-2072,E-MTAB-4057_25,HPSI0214i-kucg_2,,

IPSC,E-MTAB-4057,A-MEXP-2072,E-MTAB-4057_44,HPSI1213i-babk_2,,

IPSC,E-MTAB-4057,A-MEXP-2072,E-MTAB-4057_87,HPSI1013i-jogf_1,,

IPSC,E-MTAB-4057,A-MEXP-2072,E-MTAB-4057_166,HPSI0114i-zoxy_2,,

IPSC,E-MTAB-4057,A-MEXP-2072,E-MTAB-4057_63,HPSI0114i-eipl_3,,

IPSC,E-MTAB-4057,A-MEXP-2072,E-MTAB-4057_119,HPSI1213i-pahc_4,,

IPSC,E-MTAB-4057,A-MEXP-2072,E-MTAB-4057_57,HPSI0913i-diku_3,,

IPSC,E-MTAB-4057,A-MEXP-2072,E-MTAB-4057_90,HPSI0114i-joxm_3,,

IPSC,E-MTAB-4057,A-MEXP-2072,E-MTAB-4057_86,HPSI0114i-iisa_1,,

IPSC,E-MTAB-4057,A-MEXP-2072,E-MTAB-4057_36,HPSI0314i-sojd_1,,

ESC,E-MTAB-817,GPL6947,E-MTAB-817_7,hES thaw A,,

ESC,E-MTAB-817,GPL6947,E-MTAB-817_3,hES thaw B,,

ESC,E-MTAB-817,GPL6947,E-MTAB-817_2,hES thaw B,,

ESC,E-MTAB-817,GPL6947,E-MTAB-817_4,hES thaw C,,

ESC,E-MTAB-817,GPL6947,E-MTAB-817_5,hES thaw C,,

ESC,E-MTAB-817,GPL6947,E-MTAB-817_6,hES thaw A,,

ASC,E-TABM-978,GPL6947,E-TABM-978_59,OX273 HSC,,

ASC,E-TABM-978,GPL6947,E-TABM-978_44,OX263 HSC,,

ASC,E-TABM-978,GPL6947,E-TABM-978_39,OX219 HSC,,

ASC,E-TABM-978,GPL6947,E-TABM-978_54,OX269 HSC,,

ASC,E-TABM-978,GPL6947,E-TABM-978_49,OX266 HSC,,

IPSC,GSE17579,GPL6947,GSM438022,hiPSC_undiff_rep1,,

IPSC,GSE17579,GPL6947,GSM438034,hiPSC_undiff_rep3,,

IPSC,GSE17579,GPL6947,GSM438026,hiPSC_undiff_rep2,,

ASC,GSE21973,GPL6947,GSM546366,HSC rep2,,

ASC,GSE21973,GPL6947,GSM546367,HSC rep3,,

ASC,GSE21973,GPL6947,GSM546365,HSC rep1,,

ASC,GSE22651,GPL6947,GSM561962,SC31-MSC_B,,

ASC,GSE22651,GPL6947,GSM561963,SC41-MSC_A,,

ASC,GSE22651,GPL6947,GSM561961,SC31-MSC_A,,

ASC,GSE22651,GPL6947,GSM561964,SC41-MSC_B,,

ESC,GSE22651,GPL6947,GSM561950,Mel1_P52,,

ESC,GSE22651,GPL6947,GSM561909,BG02_B,,

ESC,GSE22651,GPL6947,GSM561928,HES-2_A,,

ESC,GSE22651,GPL6947,GSM561958,Pel_B,,

ESC,GSE22651,GPL6947,GSM561924,H9_A1,,

ESC,GSE22651,GPL6947,GSM561925,H9_A2,,

ESC,GSE22651,GPL6947,GSM561937,HSF6_B,,

ESC,GSE22651,GPL6947,GSM561907,BG01_B,,

ESC,GSE22651,GPL6947,GSM561911,BG03_B,,

ESC,GSE22651,GPL6947,GSM561951,Miz4_A,,

ESC,GSE22651,GPL6947,GSM561908,BG02_A,,

ESC,GSE22651,GPL6947,GSM561949,Mel1_P51,,

ESC,GSE22651,GPL6947,GSM561936,HSF6_A,,

ESC,GSE22651,GPL6947,GSM561952,Miz4_B,,

ESC,GSE22651,GPL6947,GSM561957,Pel_A,,

ESC,GSE22651,GPL6947,GSM561910,BG03_A,,

ESC,GSE22651,GPL6947,GSM561906,BG01_A,,

ESC,GSE22651,GPL6947,GSM561929,HES-2_B,,

IPSC,GSE22651,GPL6947,GSM561917,Gottesfeld_3816.5_2,,

IPSC,GSE22651,GPL6947,GSM561921,Gottesfeld_4078.1B3_2,,

IPSC,GSE22651,GPL6947,GSM561945,IPS-J1-12-BJ1-iPS12,,

IPSC,GSE22651,GPL6947,GSM561918,Gottesfeld_4078.1A2_1,,

IPSC,GSE22651,GPL6947,GSM561930,hFib2-Ips5_A,,

IPSC,GSE22651,GPL6947,GSM561954,MR90C2P28,,

IPSC,GSE22651,GPL6947,GSM561914,ES4CL2_A,,

IPSC,GSE22651,GPL6947,GSM561953,MR90C2P27,,

IPSC,GSE22651,GPL6947,GSM561931,hFib2-Ips5_B,,

IPSC,GSE22651,GPL6947,GSM561923,Gottesfeld_8.2A4R_2,,

IPSC,GSE22651,GPL6947,GSM561919,Gottesfeld_4078.1A2_2,,

IPSC,GSE22651,GPL6947,GSM561944,IPS-F-5-hFib2-Ips5,,

IPSC,GSE22651,GPL6947,GSM561920,Gottesfeld_4078.1B3_1,,

IPSC,GSE22651,GPL6947,GSM561922,Gottesfeld_8.2A4R_1,,

IPSC,GSE22651,GPL6947,GSM561916,Gottesfeld_3816.5_1,,

IPSC,GSE22651,GPL6947,GSM561946,IPSY2-1MSC-Ips1,,

IPSC,GSE22651,GPL6947,GSM561915,ES4CL2_B,,

IPSC,GSE22792,GPL6947,GSM563505,OSK+PRDM14 hiPSC_rep2,,

IPSC,GSE22792,GPL6947,GSM563504,OSK+PRDM14 hiPSC_rep1,,

IPSC,GSE22792,GPL6947,GSM563507,OSKC+NFRKB hiPSC_rep2,,

IPSC,GSE22792,GPL6947,GSM563506,OSKC+NFRKB hiPSC_rep1,,

IPSC,GSE22792,GPL6947,GSM563508,OSKC+PRDM14 hiPSC_rep1,,

IPSC,GSE22792,GPL6947,GSM563509,OSKC+PRDM14 hiPSC_rep2,,

IPSC,GSE22792,GPL6947,GSM563502,OSK+NFRKB hiPSC_rep1,,

IPSC,GSE22792,GPL6947,GSM563503,OSK+NFRKB hiPSC_rep2,,

AC,GSE23649,GPL6947,GSM580269,DCD9B,,

AC,GSE23649,GPL6947,GSM580262,DCD6A,,

AC,GSE23649,GPL6947,GSM580260,DCD5A,,

AC,GSE23649,GPL6947,GSM580253,LD11B,,

AC,GSE23649,GPL6947,GSM580241,LD4B,,

AC,GSE23649,GPL6947,GSM580242,LD5A,,

AC,GSE23649,GPL6947,GSM580254,DCD1A,,

AC,GSE23649,GPL6947,GSM580247,LD8B,,

AC,GSE23649,GPL6947,GSM580236,LD1A,,

AC,GSE23649,GPL6947,GSM580264,DCD7A,,

AC,GSE23649,GPL6947,GSM580263,DCD6B,,

AC,GSE23649,GPL6947,GSM580267,DCD8B,,

AC,GSE23649,GPL6947,GSM580255,DCD1B,,

AC,GSE23649,GPL6947,GSM580304,CONT3,,

AC,GSE23649,GPL6947,GSM580265,DCD7B,,

AC,GSE23649,GPL6947,GSM580245,LD6B,,

AC,GSE23649,GPL6947,GSM580240,LD4A,,

AC,GSE23649,GPL6947,GSM580246,LD8A,,

AC,GSE23649,GPL6947,GSM580266,DCD8A,,

AC,GSE23649,GPL6947,GSM580239,LD3B,,

AC,GSE23649,GPL6947,GSM580303,CONT2,,

AC,GSE23649,GPL6947,GSM580256,DCD3A,,

AC,GSE23649,GPL6947,GSM580249,LD9B,,

AC,GSE23649,GPL6947,GSM580250,LD10A,,

AC,GSE23649,GPL6947,GSM580302,CONT1,,

AC,GSE23649,GPL6947,GSM580251,LD10B,,

AC,GSE23649,GPL6947,GSM580248,LD9A,,

AC,GSE23649,GPL6947,GSM580238,LD3A,,

AC,GSE23649,GPL6947,GSM580259,DCD4B,,

AC,GSE23649,GPL6947,GSM580244,LD6A,,

AC,GSE23649,GPL6947,GSM580258,DCD4A,,

AC,GSE23649,GPL6947,GSM580257,DCD3B,,

AC,GSE23649,GPL6947,GSM580261,DCD5B,,

AC,GSE23649,GPL6947,GSM580243,LD5B,,

AC,GSE23649,GPL6947,GSM580268,DCD9A,,

AC,GSE23649,GPL6947,GSM580252,LD11A,,

AC,GSE23649,GPL6947,GSM580237,LD1B,,

ESC,GSE25046,GPL6947,GSM615029,ES_2 [mRNA],,

ESC,GSE25046,GPL6947,GSM615030,ES_3 [mRNA],,

ESC,GSE25046,GPL6947,GSM615028,ES_1 [mRNA],,

ESC,GSE25046,GPL6947,GSM615031,ES_4 [mRNA],,

AC,GSE26852,GPL6947,GSM661130,Normal Muscle individual 7,,

AC,GSE26852,GPL6947,GSM661125,Normal Muscle individual 3,,

AC,GSE26852,GPL6947,GSM661128,Normal Muscle individual 6,,

AC,GSE26852,GPL6947,GSM661126,Normal Muscle individual 4,,

AC,GSE26852,GPL6947,GSM661124,Normal Muscle individual 2,,

AC,GSE26852,GPL6947,GSM661123,Normal Muscle individual 1,,

AC,GSE26852,GPL6947,GSM661127,Normal Muscle individual 5,,

ESC,GSE28633,GPL6947,GSM709609,Human embryonic stem cell - replicate 2,,

ESC,GSE28633,GPL6947,GSM709610,Human embryonic stem cell - replicate 3,,

ESC,GSE28633,GPL6947,GSM709608,Human embryonic stem cell - replicate 1,,

ASC,GSE29105,GPL6947,GSM720943,BA_2,,

ASC,GSE29105,GPL6947,GSM720947,BB_3,,

ASC,GSE29105,GPL6947,GSM720939,AA_1,,

ASC,GSE29105,GPL6947,GSM720944,BA_3,,

ASC,GSE29105,GPL6947,GSM720946,BB_2,,

ASC,GSE29105,GPL6947,GSM720940,AA_2,,

ASC,GSE29105,GPL6947,GSM720945,BB_1,,

ASC,GSE29105,GPL6947,GSM720941,AA_3,,

ASC,GSE29105,GPL6947,GSM720942,BA_1,,

AC,GSE29221,GPL6947,GSM722674,Non-diabetic 7,,

AC,GSE29221,GPL6947,GSM722675,Non-diabetic 8,,

AC,GSE29221,GPL6947,GSM722673,Non-diabetic 6,,

AC,GSE29221,GPL6947,GSM722679,Non-diabetic 12,,

AC,GSE29221,GPL6947,GSM722668,Non-diabetic 1,,

AC,GSE29221,GPL6947,GSM722671,Non-diabetic 4,,

AC,GSE29221,GPL6947,GSM722678,Non-diabetic 11,,

AC,GSE29221,GPL6947,GSM722676,Non-diabetic 9,,

AC,GSE29221,GPL6947,GSM722672,Non-diabetic 5,,

AC,GSE29221,GPL6947,GSM722677,Non-diabetic 10,,

AC,GSE29221,GPL6947,GSM722670,Non-diabetic 3,,

AC,GSE29221,GPL6947,GSM722669,Non-diabetic 2,,

ESC,GSE29784,GPL6947,GSM738205,Embryonic stem cells Rep1,,

ESC,GSE29784,GPL6947,GSM738206,Embryonic stem cells Rep2,,

ASC,GSE30029,GPL6947,GSM743177,normal 2008 247 CD34+,,

ASC,GSE30029,GPL6947,GSM743190,normal 2009 150 CD34+,,

ASC,GSE30029,GPL6947,GSM743144,normal 2009 281 CD34+,,

ASC,GSE30029,GPL6947,GSM743093,normal 2009 062 CD34+,,

ASC,GSE30029,GPL6947,GSM743156,normal 2008 253 CD34+,,

ASC,GSE30029,GPL6947,GSM743110,normal 2008 022 CD34+,,

ASC,GSE30029,GPL6947,GSM743124,normal BM HC6 CD34+,,

ASC,GSE30029,GPL6947,GSM743147,normal 2009 118 CD34+,,

ASC,GSE30029,GPL6947,GSM743151,normal 2009 007 CD34+,,

ASC,GSE30029,GPL6947,GSM743091,normal 2009 085 CD34+,,

ASC,GSE30029,GPL6947,GSM743121,normal 2008 256 CD34+,,

ASC,GSE30029,GPL6947,GSM743113,normal 2008 248 CD34+,,

ASC,GSE30029,GPL6947,GSM743150,normal 2008 252 CD34+,,

ASC,GSE30029,GPL6947,GSM743129,normal BM HC3 CD34+,,

ASC,GSE30029,GPL6947,GSM743123,normal 2008 254 CD34+,,

ASC,GSE30029,GPL6947,GSM743153,normal HC7 CD34+,,

ASC,GSE30029,GPL6947,GSM743095,normal 2009 096 CD34+,,

ASC,GSE30029,GPL6947,GSM743167,normal 2009 037 CD34+,,

ASC,GSE30029,GPL6947,GSM743080,normal BM HC5 CD34+,,

ASC,GSE30029,GPL6947,GSM743154,normal HC9 CD34+,,

ASC,GSE30029,GPL6947,GSM743149,normal 2008 251 CD34+,,

ASC,GSE30029,GPL6947,GSM743181,normal HC2 CD34+,,

ASC,GSE30029,GPL6947,GSM743145,normal HC1 CD34+,,

ASC,GSE30029,GPL6947,GSM743074,normal 2008 250 CD34+,,

ASC,GSE30029,GPL6947,GSM743180,normal 2009 297 CD34+,,

ASC,GSE30029,GPL6947,GSM743096,normal 2008 249 CD34+,,

ASC,GSE30029,GPL6947,GSM743155,normal 2008 057 CD34+,,

ASC,GSE30029,GPL6947,GSM743157,normal HC8 CD34+,,

ASC,GSE30029,GPL6947,GSM743100,normal 2008 255 CD34+,,

ASC,GSE30029,GPL6947,GSM743148,normal 2009 005 CD34+,,

ASC,GSE30029,GPL6947,GSM743119,normal BM HC4 CD34+,,

ESC,GSE30596,GPL10558,GSM758985,HUESC6 rep1,,

ESC,GSE30596,GPL10558,GSM758986,HUESC6 rep2,,

ESC,GSE30652,GPL6947,GSM760056,ES_SIVF011_passage28_____Male_273.743.1.3,,

ESC,GSE30652,GPL6947,GSM760018,ES_SIVF041_passage17_____Female_282.752.1.1,,

ESC,GSE30652,GPL6947,GSM760036,ES_WA09_passage51_____Female_87.302.2.1,,

ESC,GSE30652,GPL6947,GSM759997,ES_SIVF001_passage18_____Female_112.396.1.1,,

ESC,GSE30652,GPL6947,GSM760048,ES_MIZ4_passage53_____Male_37.147.3.1,,

ESC,GSE30652,GPL6947,GSM759993,ES_MEL4_passage35_____Female_127.378.1.1,,

ESC,GSE30652,GPL6947,GSM760041,ES_HES4_passage56_____Male_34.116.2.1,,

ESC,GSE30652,GPL6947,GSM759976,ES_CSC14_passage26_____Female_197.524.1.1,,

ESC,GSE30652,GPL6947,GSM760004,ES_SIVF007_passage23_____Female_115.356.2.1,,

ESC,GSE30652,GPL6947,GSM760002,ES_SIVF006_passage24_____Female_114.355.2.1,,

ESC,GSE30652,GPL6947,GSM760057,ES_SIVF014_passage42_____Male_295.773.1.2,,

ESC,GSE30652,GPL6947,GSM759990,ES_MEL2_passage13_____Female_36.131.2.1,,

ESC,GSE30652,GPL6947,GSM759973,ES_CM8_passage12_____Female_241.619.1.2,,

ESC,GSE30652,GPL6947,GSM759980,ES_ESI053_passage37_____Female_30.105.4.1,,

ESC,GSE30652,GPL6947,GSM760010,ES_SIVF020_passage52_____Female_119.350.2.1,,

ESC,GSE30652,GPL6947,GSM760069,ES_SIVF045_passage13_____Male_286.756.1.1,,

ESC,GSE30652,GPL6947,GSM760067,ES_SIVF043_passage12_____Male_284.754.1.1,,

ESC,GSE30652,GPL6947,GSM760021,ES_SIVF047_passage10_____Female_288.758.1.1,,

ESC,GSE30652,GPL6947,GSM760044,ES_HUES16_passage21_____Male_70.233.1.1,,

ESC,GSE30652,GPL6947,GSM760017,ES_SIVF040_passage17_____Female_281.751.1.1,,

ESC,GSE30652,GPL6947,GSM759989,ES_HES3_passage59_____Female_33.118.2.1,,

ESC,GSE30652,GPL6947,GSM760049,ES_MIZ4_passage53_____Male_37.148.2.1,,

ESC,GSE30652,GPL6947,GSM760043,ES_HES4_passage60_____Male_34.121.2.1,,

ESC,GSE30652,GPL6947,GSM759972,ES_CM7_passage19_____Female_243.621.2.2,,

ESC,GSE30652,GPL6947,GSM760050,ES_MIZ4_passage62_____Male_37.156.2.1,,

ESC,GSE30652,GPL6947,GSM760007,ES_SIVF018_passage72_____Female_293.770.1.1,,

ESC,GSE30652,GPL6947,GSM759983,ES_HES2_passage28_____Female_32.273.3.1,,

ESC,GSE30652,GPL6947,GSM760011,ES_SIVF020_passage82_____Female_289.762.1.1,,

ESC,GSE30652,GPL6947,GSM760051,ES_MIZ4_passage63_____Male_37.151.3.1,,

ESC,GSE30652,GPL6947,GSM760008,ES_SIVF019_passage67_____Female_118.776.1.2,,

ESC,GSE30652,GPL6947,GSM759969,ES_CM1_passage16_____Female_246.624.2.1,,

ESC,GSE30652,GPL6947,GSM759966,ES_CM11_passage17_____Female_240.617.1.2,,

ESC,GSE30652,GPL6947,GSM759968,ES_CM14_passage87_____Female_239.615.1.2,,

ESC,GSE30652,GPL6947,GSM759991,ES_MEL2_passage15_____Female_36.133.2.1,,

ESC,GSE30652,GPL6947,GSM760064,ES_SIVF022_passage48_____Male_121.768.1.1,,

ESC,GSE30652,GPL6947,GSM760001,ES_SIVF006_passage22_____Female_114.344.2.1,,

ESC,GSE30652,GPL6947,GSM759978,ES_ESI035_passage35_____Female_27.102.3.1,,

ESC,GSE30652,GPL6947,GSM760035,ES_WA09_passage46_____Female_87.144.2.1,,

ESC,GSE30652,GPL6947,GSM760126,ES.parthenote_LLC6_passage16_____Female_43.172.1.1,,

ESC,GSE30652,GPL6947,GSM760042,ES_HES4_passage57_____Male_34.117.2.1,,

ESC,GSE30652,GPL6947,GSM760015,ES_SIVF028_passage28_____Female_276.746.1.1,,

ESC,GSE30652,GPL6947,GSM759974,ES_CM8_passage25_____Female_241.618.1.2,,

ESC,GSE30652,GPL6947,GSM760054,ES_SIVF002_passage50_____Male_290.763.1.1,,

ESC,GSE30652,GPL6947,GSM760037,ES_BG02_passage46_____Male_54.215.1.1,,

ESC,GSE30652,GPL6947,GSM760027,ES_WA07_passage35_____Female_47.176.1.6,,

ESC,GSE30652,GPL6947,GSM760014,ES_SIVF027_passage31_____Female_297.777.1.2,,

ESC,GSE30652,GPL6947,GSM760066,ES_SIVF026_passage30_____Male_298.778.1.3,,

ESC,GSE30652,GPL6947,GSM760127,ES.parthenote_LLC6_passage45_____Female_43.904.1.1,,

ESC,GSE30652,GPL6947,GSM760006,ES_SIVF018_passage44_____Female_117.347.2.1,,

ESC,GSE30652,GPL6947,GSM760059,ES_SIVF017_passage43_____Male_116.346.3.1,,

ESC,GSE30652,GPL6947,GSM760065,ES_SIVF025_passage35_____Male_296.775.1.3,,

ESC,GSE30652,GPL6947,GSM760125,ES.parthenote_LLC2_passage24_____Female_42.171.1.7,,

ESC,GSE30652,GPL6947,GSM759994,ES_MEL4_passage36_____Female_127.379.1.1,,

ESC,GSE30652,GPL6947,GSM760128,ES.parthenote_LLC7_passage16_____Female_44.173.1.6,,

ESC,GSE30652,GPL6947,GSM759982,ES_HES2_passage105_____Female_32.135.2.1,,

ESC,GSE30652,GPL6947,GSM759987,ES_HES3_passage31_____Female_33.276.2.1,,

ESC,GSE30652,GPL6947,GSM759988,ES_HES3_passage54_____Female_33.112.2.1,,

ESC,GSE30652,GPL6947,GSM760034,ES_WA09_passage44_____Female_87.453.1.2,,

ESC,GSE30652,GPL6947,GSM759971,ES_CM6_passage19_____Female_244.622.1.2,,

ESC,GSE30652,GPL6947,GSM760026,ES_UC06_passage71_____Female_39.157.3.1,,

ESC,GSE30652,GPL6947,GSM760016,ES_SIVF029_passage28_____Female_277.747.1.1,,

ESC,GSE30652,GPL6947,GSM760012,ES_SIVF024_passage15_____Female_294.772.1.2,,

ESC,GSE30652,GPL6947,GSM760053,ES_SIVF002_passage17_____Male_136.398.1.1,,

ESC,GSE30652,GPL6947,GSM760060,ES_SIVF017_passage67_____Male_292.769.1.1,,

ESC,GSE30652,GPL6947,GSM760129,ES.parthenote_LLC8_passage18_____Female_45.174.1.7,,

ESC,GSE30652,GPL6947,GSM760030,ES_WA09_passage39_____Female_87.138.2.1,,

ESC,GSE30652,GPL6947,GSM760023,ES_UC06_passage61_____Female_39.158.2.1,,

ESC,GSE30652,GPL6947,GSM759970,ES_CM2_passage17_____Female_245.623.1.2,,

ESC,GSE30652,GPL6947,GSM760040,ES_FES22_passage44_____Male_23.93.2.1,,

ESC,GSE30652,GPL6947,GSM760070,ES_SIVF048_passage13_____Male_278.748.1.1,,

ESC,GSE30652,GPL6947,GSM760009,ES_SIVF019_passage81_____Female_118.761.1.1,,

ESC,GSE30652,GPL6947,GSM760029,ES_WA09_passage39_____Female_87.142.2.1,,

ESC,GSE30652,GPL6947,GSM760005,ES_SIVF016_passage19_____Female_275.745.1.1,,

ESC,GSE30652,GPL6947,GSM759998,ES_SIVF001_passage45_____Female_112.342.2.1,,

ESC,GSE30652,GPL6947,GSM759975,ES_CSC14C1_passage26_____Female_202.550.1.2,,

ESC,GSE30652,GPL6947,GSM760045,ES_MEL1_passage51_____Male_35.122.2.1,,

ESC,GSE30652,GPL6947,GSM759986,ES_HES2_passage92_____Female_32.110.2.1,,

ESC,GSE30652,GPL6947,GSM759965,ES_BG03_passage55_____Female_124.372.1.1,,

ESC,GSE30652,GPL6947,GSM759984,ES_HES2_passage55_____Female_32.114.2.1,,

ESC,GSE30652,GPL6947,GSM760039,ES_FES22_passage44_____Male_23.92.2.1,,

ESC,GSE30652,GPL6947,GSM760052,ES_MIZ4_passage65_____Male_37.157.3.1,,

ESC,GSE30652,GPL6947,GSM760024,ES_UC06_passage61_____Female_39.159.2.1,,

ESC,GSE30652,GPL6947,GSM759977,ES_ESI017_passage24_____Female_26.101.2.1,,

ESC,GSE30652,GPL6947,GSM759992,ES_MEL3_passage34_____Female_126.376.1.1,,

ESC,GSE30652,GPL6947,GSM760061,ES_SIVF021_passage19_____Male_120.351.2.1,,

ESC,GSE30652,GPL6947,GSM760020,ES_SIVF046_passage11_____Female_287.757.1.1,,

ESC,GSE30652,GPL6947,GSM759995,ES_MIZ6_passage27_____Female_38.161.2.1,,

ESC,GSE30652,GPL6947,GSM760058,ES_SIVF015_passage18_____Male_274.744.1.1,,

ESC,GSE30652,GPL6947,GSM759979,ES_ESI051_passage37_____Female_29.104.2.1,,

ESC,GSE30652,GPL6947,GSM760033,ES_WA09_passage43_____Female_87.521.1.2,,

ESC,GSE30652,GPL6947,GSM759999,ES_SIVF003_passage22_____Female_113.343.2.1,,

ESC,GSE30652,GPL6947,GSM760038,ES_ESI049_passage38_____Male_28.103.3.1,,

ESC,GSE30652,GPL6947,GSM760000,ES_SIVF004_passage43_____Female_291.766.1.1,,

ESC,GSE30652,GPL6947,GSM759996,ES_MIZ6_passage27_____Female_38.162.3.1,,

ESC,GSE30652,GPL6947,GSM760071,ES_SIVF050_passage9_____Male_280.750.1.1,,

ESC,GSE30652,GPL6947,GSM760055,ES_SIVF005_passage44_____Male_123.767.1.1,,

ESC,GSE30652,GPL6947,GSM760062,ES_SIVF021_passage49_____Male_120.764.1.1,,

ESC,GSE30652,GPL6947,GSM760025,ES_UC06_passage70_____Female_39.156.2.1,,

ESC,GSE30652,GPL6947,GSM760130,ES.parthenote_LLC9_passage20_____Female_46.175.1.6,,

ESC,GSE30652,GPL6947,GSM760046,ES_MEL1_passage59_____Male_35.125.2.1,,

ESC,GSE30652,GPL6947,GSM759981,ES_FES30_passage26_____Female_22.91.2.1,,

ESC,GSE30652,GPL6947,GSM759967,ES_CM14_passage21_____Female_239.616.1.2,,

ESC,GSE30652,GPL6947,GSM760019,ES_SIVF042_passage13_____Female_283.753.1.1,,

ESC,GSE30652,GPL6947,GSM760047,ES_MEL1_passage59_____Male_35.128.2.1,,

ESC,GSE30652,GPL6947,GSM760124,ES.parthenote_LLC15_passage45_____Female_317.905.1.4,,

ESC,GSE30652,GPL6947,GSM760063,ES_SIVF022_passage19_____Male_121.352.2.1,,

ESC,GSE30652,GPL6947,GSM760003,ES_SIVF007_passage21_____Female_115.345.2.1,,

ESC,GSE30652,GPL6947,GSM759985,ES_HES2_passage91_____Female_32.275.2.1,,

ESC,GSE30652,GPL6947,GSM760032,ES_WA09_passage41_____Female_87.304.7.4,,

ESC,GSE30652,GPL6947,GSM760028,ES_WA07_passage56_____Female_47.177.1.6,,

ESC,GSE30652,GPL6947,GSM760068,ES_SIVF044_passage13_____Male_285.755.1.1,,

ESC,GSE30652,GPL6947,GSM760031,ES_WA09_passage40_____Female_87.137.2.1,,

ESC,GSE30652,GPL6947,GSM760022,ES_SIVF049_passage9_____Female_279.749.1.1,,

ESC,GSE30652,GPL6947,GSM760013,ES_SIVF024_passage28_____Female_294.774.1.2,,

IPSC,GSE30652,GPL6947,GSM760099,iPS_C14_passage32_____Male_310.889.1.1,,

IPSC,GSE30652,GPL6947,GSM760119,iPS_HDF51IPS3_passage34_____Female_155.645.1.1,,

IPSC,GSE30652,GPL6947,GSM760076,iPS_HDF51IPS1_passage6_____Female_153.437.2.1,,

IPSC,GSE30652,GPL6947,GSM760072,iPS_HDF51IPS10_passage6_____Female_161.445.1.2,,

IPSC,GSE30652,GPL6947,GSM760122,iPS_HDF51IPS7_passage33_____Female_158.648.1.2,,

IPSC,GSE30652,GPL6947,GSM760115,iPS_HDF51IPS12_passage33_____Female_163.651.1.1,,

IPSC,GSE30652,GPL6947,GSM760089,iPS_HDF51IPS3_passage12_____Female_155.490.2.1,,

IPSC,GSE30652,GPL6947,GSM760081,iPS_HDF51IPS7_passage5_____Female_158.442.1.2,,

IPSC,GSE30652,GPL6947,GSM760121,iPS_HDF51IPS6_passage33_____Female_157.647.1.1,,

IPSC,GSE30652,GPL6947,GSM760080,iPS_HDF51IPS6_passage5_____Female_157.441.1.2,,

IPSC,GSE30652,GPL6947,GSM760100,iPS_IPS-Foreskin-CL1_passage18_____Male_130.384.1.1,,

IPSC,GSE30652,GPL6947,GSM760116,iPS_HDF51IPS13_passage34_____Female_164.652.1.3,,

IPSC,GSE30652,GPL6947,GSM760082,iPS_HDF51IPS9_passage7_____Female_160.473.1.2,,

IPSC,GSE30652,GPL6947,GSM760092,iPS_HDF51IPS7_passage11_____Female_158.493.2.1,,

IPSC,GSE30652,GPL6947,GSM760104,iPS_GOTTESFELD4078.1A2_passage27_____Male_302.782.1.1,,

IPSC,GSE30652,GPL6947,GSM760088,iPS_HDF51IPS2_passage12_____Female_154.489.1.1,,

IPSC,GSE30652,GPL6947,GSM760093,iPS_HDF51IPS9_passage14_____Female_160.500.2.1,,

IPSC,GSE30652,GPL6947,GSM760084,iPS_HDF51IPS11_passage14_____Female_162.501.2.1,,

IPSC,GSE30652,GPL6947,GSM760086,iPS_HDF51IPS13_passage14_____Female_164.502.1.1,,

IPSC,GSE30652,GPL6947,GSM760097,iPS_IPS-IMR90C4_passage27_____Female_135.394.1.1,,

IPSC,GSE30652,GPL6947,GSM760106,iPS_HFIB2IPS5_passage69_____Male_85.420.1.1,,

IPSC,GSE30652,GPL6947,GSM760078,iPS_HDF51IPS3_passage6_____Female_155.439.1.2,,

IPSC,GSE30652,GPL6947,GSM760087,iPS_HDF51IPS1_passage12_____Female_153.488.2.1,,

IPSC,GSE30652,GPL6947,GSM760090,iPS_HDF51IPS5_passage12_____Female_156.491.2.1,,

IPSC,GSE30652,GPL6947,GSM760105,iPS_GOTTESFELD4078.1B3_passage20_____Male_301.781.1.1,,

IPSC,GSE30652,GPL6947,GSM760079,iPS_HDF51IPS5_passage6_____Female_156.440.1.2,,

IPSC,GSE30652,GPL6947,GSM760123,iPS_HDF51IPS9_passage33_____Female_160.649.1.1,,

IPSC,GSE30652,GPL6947,GSM760107,iPS_K4AMKIPS4F1_passage15_____Male_138.401.1.1,,

IPSC,GSE30652,GPL6947,GSM760112,iPS_TH160OCT4SOX2BTHAL_passage38_____Male_311.890.1.1,,

IPSC,GSE30652,GPL6947,GSM760118,iPS_HDF51IPS2_passage34_____Female_154.644.1.1,,

IPSC,GSE30652,GPL6947,GSM760120,iPS_HDF51IPS5_passage34_____Female_156.646.1.1,,

IPSC,GSE30652,GPL6947,GSM760109,iPS_NSC253I_passage23_____Male_314.893.1.1,,

IPSC,GSE30652,GPL6947,GSM760102,iPS_IPS-Foreskin-CL3_passage19_____Male_132.389.2.1,,

IPSC,GSE30652,GPL6947,GSM760075,iPS_HDF51IPS13_passage8_____Female_164.471.1.1,,

IPSC,GSE30652,GPL6947,GSM760113,iPS_HDF51IPS10_passage37_____Female_161.900.1.3,,

IPSC,GSE30652,GPL6947,GSM760103,iPS_IPS-Foreskin-CL4_passage18_____Male_133.390.2.1,,

IPSC,GSE30652,GPL6947,GSM760073,iPS_HDF51IPS11_passage8_____Female_162.475.1.1,,

IPSC,GSE30652,GPL6947,GSM760108,iPS_KIPS4F8_passage27_____Male_250.668.1.1,,

IPSC,GSE30652,GPL6947,GSM760098,iPS_KIPS4F1_passage32_____Female_249.667.1.1,,

IPSC,GSE30652,GPL6947,GSM760083,iPS_HDF51IPS10_passage12_____Female_161.495.2.1,,

IPSC,GSE30652,GPL6947,GSM760101,iPS_IPS-Foreskin-CL2_passage17_____Male_131.387.2.1,,

IPSC,GSE30652,GPL6947,GSM760095,iPS_GOTTESFELD8.2A4R_passage28_____Female_299.779.1.1,,

IPSC,GSE30652,GPL6947,GSM760085,iPS_HDF51IPS12_passage12_____Female_163.497.2.1,,

IPSC,GSE30652,GPL6947,GSM760074,iPS_HDF51IPS12_passage6_____Female_163.447.1.2,,

IPSC,GSE30652,GPL6947,GSM760114,iPS_HDF51IPS11_passage33_____Female_162.650.1.2,,

IPSC,GSE30652,GPL6947,GSM760110,iPS_TH124SOX2BTHAL_passage34_____Male_309.888.2.1,,

IPSC,GSE30652,GPL6947,GSM760077,iPS_HDF51IPS2_passage6_____Female_154.438.1.1,,

IPSC,GSE30652,GPL6947,GSM760094,iPS_GOTTESFELD3816.5_passage14_____Female_300.780.1.1,,

IPSC,GSE30652,GPL6947,GSM760111,iPS_TH152OCT4BTHAL_passage30_____Male_308.887.1.1,,

IPSC,GSE30652,GPL6947,GSM760096,iPS_IPS-IMR90C2_passage27_____Female_134.392.1.1,,

IPSC,GSE30652,GPL6947,GSM760091,iPS_HDF51IPS6_passage11_____Female_157.492.2.1,,

IPSC,GSE30652,GPL6947,GSM760117,iPS_HDF51IPS1_passage25_____Female_153.894.1.1,,

ESC,GSE31845,GPL6947,GSM789843,Human Embryonic Stem Cell Line (H9) [gene expr],,

IPSC,GSE31845,GPL6947,GSM789840,EN13 iPSC Line (EH6A) [gene expr],,

IPSC,GSE31845,GPL6947,GSM789839,EN13 iPSC Line (EH6) [gene expr],,

IPSC,GSE31845,GPL6947,GSM789842,IMR90-4 iPSC Line [gene expr],,

IPSC,GSE31845,GPL6947,GSM789837,EN13 iPSC Line (EH2) [gene expr],,

IPSC,GSE31845,GPL6947,GSM789836,EN13 iPSC Line (EH1) [gene expr],,

IPSC,GSE31845,GPL6947,GSM789838,EN13 iPSC Line (EH3) [gene expr],,

IPSC,GSE31845,GPL6947,GSM789841,EN13 iPSC Line (B2) [gene expr],,

IPSC,GSE32581,GPL10558,GSM807456,HiPS28-23,,

IPSC,GSE32581,GPL10558,GSM807450,HiPS20-1,,

IPSC,GSE32581,GPL10558,GSM807452,HiPS20-9,,

IPSC,GSE32581,GPL10558,GSM807454,HiPS24-2,,

IPSC,GSE32581,GPL10558,GSM807458,HiPS28-27,,

ESC,GSE34912,GPL10558,GSM857441,H1_bmp4_ct3_d2,,

ESC,GSE34912,GPL10558,GSM857443,H1_bmp4_ct5_d4,,

ESC,GSE34912,GPL10558,GSM857446,H1_bmp4_ct8_d7,,

ESC,GSE34912,GPL10558,GSM857439,H1_bmp4_ct1_d0,,

ESC,GSE34912,GPL10558,GSM857444,H1_bmp4_ct6_d5,,

ESC,GSE34912,GPL10558,GSM857442,H1_bmp4_ct4_d3,,

ESC,GSE34912,GPL10558,GSM857445,H1_bmp4_ct7_d6,,

ESC,GSE34912,GPL10558,GSM857440,H1_bmp4_ct2_d1,,

ESC,GSE34918,GPL10558,GSM857497,H1P rep2,,

ESC,GSE34918,GPL10558,GSM857496,H1P rep1,,

ESC,GSE34920,GPL10558,GSM857506,EF1a-control_2,,

ESC,GSE34920,GPL10558,GSM857505,EF1a-control_1,,

ESC,GSE35028,GPL6947,GSM860997,human Embryonic Stem Cell,,

IPSC,GSE35028,GPL6947,GSM861000,nonviral Cord Blood iPSC clone 6.13,,

IPSC,GSE35028,GPL6947,GSM861003,nonviral Keratinocyte iPSC clone 3,,

IPSC,GSE35028,GPL6947,GSM861004,nonviral Fetal fibroblast iPSC clone 1,,

IPSC,GSE35028,GPL6947,GSM861005,nonviral Fetal fibroblast iPSC clone 6,,

IPSC,GSE35028,GPL6947,GSM861002,nonviral Keratinocyte iPSC clone 1,,

IPSC,GSE35028,GPL6947,GSM861001,nonviral Cord Blood iPSC clone 19.11,,

IPSC,GSE35028,GPL6947,GSM860998,nonviral Cord Blood iPSC clone 6.2,,

IPSC,GSE35028,GPL6947,GSM860999,nonviral Cord Blood iPSC clone 6.11,,

IPSC,GSE35347,GPL10558,GSM866576,iPS1 rep1,,

IPSC,GSE35347,GPL10558,GSM866588,iPS2 rep4,,

IPSC,GSE35347,GPL10558,GSM866582,iPS2 rep1,,

IPSC,GSE35347,GPL10558,GSM866578,iPS1 rep3,,

IPSC,GSE35347,GPL10558,GSM866590,iPS2 rep6,,

IPSC,GSE35347,GPL10558,GSM866577,iPS1 rep2,,

IPSC,GSE35347,GPL10558,GSM866583,iPS2 rep2,,

IPSC,GSE35347,GPL10558,GSM866581,iPS1 rep6,,

IPSC,GSE35347,GPL10558,GSM866589,iPS2 rep5,,

IPSC,GSE35347,GPL10558,GSM866584,iPS2 rep3,,

IPSC,GSE35347,GPL10558,GSM866580,iPS1 rep5,,

IPSC,GSE35347,GPL10558,GSM866579,iPS1 rep4,,

ASC,GSE35390,GPL10558,GSM867237,OB1 P20,,

ASC,GSE35390,GPL10558,GSM867236,OB2 P9,,

ASC,GSE35390,GPL10558,GSM867235,OB1 P9,,

ASC,GSE35390,GPL10558,GSM867242,NSC_2,,

ASC,GSE35390,GPL10558,GSM867243,NSC_3,,

ASC,GSE35390,GPL10558,GSM867241,NSC_1,,

ASC,GSE35390,GPL10558,GSM867238,OB2 P20,,

ASC,GSE35390,GPL10558,GSM867239,OB1 P25,,

ASC,GSE35390,GPL10558,GSM867240,OB2 P22,,

AC,GSE35399,GPL10558,GSM867377,Stromal-cells-022,,

AC,GSE35399,GPL10558,GSM867425,Stromal-cells-125,,

AC,GSE35399,GPL10558,GSM867359,Stromal-cells-007,,

AC,GSE35399,GPL10558,GSM867410,Stromal-cells-100,,

AC,GSE35399,GPL10558,GSM867387,Stromal-cells-050,,

AC,GSE35399,GPL10558,GSM867403,Stromal-cells-071,,

AC,GSE35399,GPL10558,GSM867365,Stromal-cells-010,,

AC,GSE35399,GPL10558,GSM867415,Stromal-cells-110,,

AC,GSE35399,GPL10558,GSM867371,Stromal-cells-019,,

AC,GSE35399,GPL10558,GSM867393,Stromal-cells-056,,

ESC,GSE35911,GPL10558,GSM877297,H1P99C,,

ESC,GSE35911,GPL10558,GSM877295,H1P99B,,

ESC,GSE35911,GPL10558,GSM877283,H1P99A,,

IPSC,GSE35911,GPL10558,GSM877296,iPSIMR90Col1,,

IPSC,GSE35911,GPL10558,GSM877298,iPSIMR90Col9,,

IPSC,GSE35911,GPL10558,GSM877284,iPSIMR90Col10,,

AC,GSE36192,GPL6947,GSM882584,UMARY-1403-CRBLM,,

AC,GSE36192,GPL6947,GSM882752,SH-95-27-CRBLM,,

AC,GSE36192,GPL6947,GSM883136,UMARY-1672-FCTX,,

AC,GSE36192,GPL6947,GSM882463,UMARY-4786-CRBLM,,

AC,GSE36192,GPL6947,GSM882841,028-08-CRBLM,,

AC,GSE36192,GPL6947,GSM882681,UMARY-5082-CRBLM,,

AC,GSE36192,GPL6947,GSM883094,UMARY-5123-FCTX,,

AC,GSE36192,GPL6947,GSM883120,MIAMI-3839-FCTX,,

AC,GSE36192,GPL6947,GSM883051,UMARY-305-FCTX,,

AC,GSE36192,GPL6947,GSM882876,UMARY-5125-CRBLM,,

AC,GSE36192,GPL6947,GSM882874,010-10-CRBLM,,

AC,GSE36192,GPL6947,GSM883204,SH-92-14-FCTX,,

AC,GSE36192,GPL6947,GSM882666,UMARY-1363-CRBLM,,

AC,GSE36192,GPL6947,GSM882808,002-10-CRBLM,,

AC,GSE36192,GPL6947,GSM882905,BLSA-2020-FCTX,,

AC,GSE36192,GPL6947,GSM882586,UMARY-1323-CRBLM,,

AC,GSE36192,GPL6947,GSM883274,001-10-FCTX,,

AC,GSE36192,GPL6947,GSM882456,UMARY-4636-CRBLM,,

AC,GSE36192,GPL6947,GSM882630,UMARY-1347-CRBLM,,

AC,GSE36192,GPL6947,GSM883270,033-08-FCTX,,

AC,GSE36192,GPL6947,GSM883068,UMARY-1500-FCTX,,

AC,GSE36192,GPL6947,GSM882689,UMARY-4546-CRBLM,,

AC,GSE36192,GPL6947,GSM882537,UMARY-1795-CRBLM,,

AC,GSE36192,GPL6947,GSM882470,UMARY-4924-CRBLM,,

AC,GSE36192,GPL6947,GSM883325,007-09-FCTX,,

AC,GSE36192,GPL6947,GSM882763,SH-00-49-CRBLM,,

AC,GSE36192,GPL6947,GSM883164,SH-04-08-FCTX,,

AC,GSE36192,GPL6947,GSM882484,BLSA-2037-CRBLM,,

AC,GSE36192,GPL6947,GSM883012,UMARY-4789-FCTX,,

AC,GSE36192,GPL6947,GSM882764,SH-01-14-CRBLM,,

AC,GSE36192,GPL6947,GSM882550,UMARY-880-CRBLM,,

AC,GSE36192,GPL6947,GSM882483,BLSA-1961-CRBLM,,

AC,GSE36192,GPL6947,GSM883157,UMARY-1744-FCTX,,

AC,GSE36192,GPL6947,GSM882631,UMARY-1675-CRBLM,,

AC,GSE36192,GPL6947,GSM882992,UMARY-1713-FCTX,,

AC,GSE36192,GPL6947,GSM882564,UMARY-1935-CRBLM,,

AC,GSE36192,GPL6947,GSM882951,JHU-993-FCTX,,

AC,GSE36192,GPL6947,GSM882545,UMARY-1847-CRBLM,,

AC,GSE36192,GPL6947,GSM882548,UMARY-1866-CRBLM,,

AC,GSE36192,GPL6947,GSM882527,UMARY-1226-CRBLM,,

AC,GSE36192,GPL6947,GSM883081,UMARY-675-FCTX,,

AC,GSE36192,GPL6947,GSM882790,033-09-CRBLM,,

AC,GSE36192,GPL6947,GSM882481,BLSA-1805-CRBLM,,

AC,GSE36192,GPL6947,GSM883333,UMARY-4669-FCTX,,

AC,GSE36192,GPL6947,GSM882932,UMARY-5087-FCTX,,

AC,GSE36192,GPL6947,GSM882818,004-06-CRBLM,,

AC,GSE36192,GPL6947,GSM883211,MIAMI-3296-FCTX,,

AC,GSE36192,GPL6947,GSM882800,001-06-CRBLM,,

AC,GSE36192,GPL6947,GSM882832,012-08-CRBLM,,

AC,GSE36192,GPL6947,GSM882693,UMARY-5173-CRBLM,,

AC,GSE36192,GPL6947,GSM882778,SH-96-35-CRBLM,,

AC,GSE36192,GPL6947,GSM883024,UMARY-1274-FCTX,,

AC,GSE36192,GPL6947,GSM882735,SH-99-31-CRBLM,,

AC,GSE36192,GPL6947,GSM882934,BLSA-1579-FCTX,,

AC,GSE36192,GPL6947,GSM883124,UMARY-4643-FCTX,,

AC,GSE36192,GPL6947,GSM883197,SH-03-28-FCTX,,

AC,GSE36192,GPL6947,GSM883287,019-08-FCTX,,

AC,GSE36192,GPL6947,GSM882927,UMARY-4924-FCTX,,

AC,GSE36192,GPL6947,GSM882644,MIAMI-3651-CRBLM,,

AC,GSE36192,GPL6947,GSM882812,018-08-CRBLM,,

AC,GSE36192,GPL6947,GSM883312,017-08-FCTX,,

AC,GSE36192,GPL6947,GSM882847,023-09-CRBLM,,

AC,GSE36192,GPL6947,GSM883240,SH-98-19-FCTX,,

AC,GSE36192,GPL6947,GSM883183,SH-97-37-FCTX,,

AC,GSE36192,GPL6947,GSM883091,UMARY-1378-FCTX,,

AC,GSE36192,GPL6947,GSM883004,UMARY-1862-FCTX,,

AC,GSE36192,GPL6947,GSM882637,UMARY-5123-CRBLM,,

AC,GSE36192,GPL6947,GSM882882,SH-96-22-CRBLM,,

AC,GSE36192,GPL6947,GSM883261,011-09-FCTX,,

AC,GSE36192,GPL6947,GSM883025,UMARY-878-FCTX,,

AC,GSE36192,GPL6947,GSM882900,UMARY-1849-FCTX,,

AC,GSE36192,GPL6947,GSM883129,UMARY-662-FCTX,,

AC,GSE36192,GPL6947,GSM883328,015-09-FCTX,,

AC,GSE36192,GPL6947,GSM883174,SH-07-46-FCTX,,

AC,GSE36192,GPL6947,GSM883054,UMARY-671-FCTX,,

AC,GSE36192,GPL6947,GSM882466,UMARY-4842-CRBLM,,

AC,GSE36192,GPL6947,GSM882568,UMARY-878-CRBLM,,

AC,GSE36192,GPL6947,GSM882909,UMARY-4543-FCTX,,

AC,GSE36192,GPL6947,GSM882839,018-06-CRBLM,,

AC,GSE36192,GPL6947,GSM882655,UMARY-1461-CRBLM,,

AC,GSE36192,GPL6947,GSM882853,029-09-CRBLM,,

AC,GSE36192,GPL6947,GSM882492,JHU-713-CRBLM,,

AC,GSE36192,GPL6947,GSM883300,032-09-FCTX,,

AC,GSE36192,GPL6947,GSM883303,007-10-FCTX,,

AC,GSE36192,GPL6947,GSM882894,UMARY-1326-FCTX,,

AC,GSE36192,GPL6947,GSM883222,SH-02-06-FCTX,,

AC,GSE36192,GPL6947,GSM882864,034-09-CRBLM,,

AC,GSE36192,GPL6947,GSM883049,UMARY-4592-FCTX,,

AC,GSE36192,GPL6947,GSM882973,UMARY-1936-FCTX,,

AC,GSE36192,GPL6947,GSM882824,016-09-CRBLM,,

AC,GSE36192,GPL6947,GSM883327,006-10-FCTX,,

AC,GSE36192,GPL6947,GSM882924,UMARY-4903-FCTX,,

AC,GSE36192,GPL6947,GSM883205,SH-97-19-FCTX,,

AC,GSE36192,GPL6947,GSM883057,UMARY-1101-FCTX,,

AC,GSE36192,GPL6947,GSM882953,JHU-995-FCTX,,

AC,GSE36192,GPL6947,GSM883122,MIAMI-3772-FCTX,,

AC,GSE36192,GPL6947,GSM882590,UMARY-3-CRBLM,,

AC,GSE36192,GPL6947,GSM882663,MIAMI-3839-CRBLM,,

AC,GSE36192,GPL6947,GSM882880,SH-97-14-CRBLM,,

AC,GSE36192,GPL6947,GSM882514,UMARY-1917-CRBLM,,

AC,GSE36192,GPL6947,GSM882861,006-08-CRBLM,,

AC,GSE36192,GPL6947,GSM882786,UMARY-1607-CRBLM,,

AC,GSE36192,GPL6947,GSM882727,SH-96-13-CRBLM,,

AC,GSE36192,GPL6947,GSM882922,UMARY-4841-FCTX,,

AC,GSE36192,GPL6947,GSM882670,UMARY-1111-CRBLM,,

AC,GSE36192,GPL6947,GSM882482,BLSA-1924-CRBLM,,

AC,GSE36192,GPL6947,GSM883016,UMARY-813-FCTX,,

AC,GSE36192,GPL6947,GSM882572,UMARY-1789-CRBLM,,

AC,GSE36192,GPL6947,GSM882917,UMARY-4727-FCTX,,

AC,GSE36192,GPL6947,GSM882623,UMARY-1266-CRBLM,,

AC,GSE36192,GPL6947,GSM882528,UMARY-1230-CRBLM,,

AC,GSE36192,GPL6947,GSM882645,UMARY-1027-CRBLM,,

AC,GSE36192,GPL6947,GSM883255,006-09-FCTX,,

AC,GSE36192,GPL6947,GSM883317,038-08-FCTX,,

AC,GSE36192,GPL6947,GSM883214,UMARY-4735-FCTX,,

AC,GSE36192,GPL6947,GSM882994,UMARY-1792-FCTX,,

AC,GSE36192,GPL6947,GSM882838,020-08-CRBLM,,

AC,GSE36192,GPL6947,GSM882635,MIAMI-3747-CRBLM,,

AC,GSE36192,GPL6947,GSM882834,038-08-CRBLM,,

AC,GSE36192,GPL6947,GSM883144,UMARY-510-FCTX,,

AC,GSE36192,GPL6947,GSM882444,BLSA-1883-CRBLM,,

AC,GSE36192,GPL6947,GSM882692,UMARY-455-CRBLM,,

AC,GSE36192,GPL6947,GSM882993,UMARY-1715-FCTX,,

AC,GSE36192,GPL6947,GSM882872,024-08-CRBLM,,

AC,GSE36192,GPL6947,GSM882554,UMARY-4789-CRBLM,,

AC,GSE36192,GPL6947,GSM883151,UMARY-105-FCTX,,

AC,GSE36192,GPL6947,GSM882810,034-07-CRBLM,,

AC,GSE36192,GPL6947,GSM882985,UMARY-1226-FCTX,,

AC,GSE36192,GPL6947,GSM882461,UMARY-4729-CRBLM,,

AC,GSE36192,GPL6947,GSM883221,SH-01-14-FCTX,,

AC,GSE36192,GPL6947,GSM882443,UMARY-1849-CRBLM,,

AC,GSE36192,GPL6947,GSM882749,SH-05-36-CRBLM,,

AC,GSE36192,GPL6947,GSM882617,UMARY-257-CRBLM,,

AC,GSE36192,GPL6947,GSM883206,SH-05-36-FCTX,,

AC,GSE36192,GPL6947,GSM882881,SH-07-63-CRBLM,,

AC,GSE36192,GPL6947,GSM883155,UMARY-4722-FCTX,,

AC,GSE36192,GPL6947,GSM883046,UMARY-1818-FCTX,,

AC,GSE36192,GPL6947,GSM882504,UMARY-1571-CRBLM,,

AC,GSE36192,GPL6947,GSM883286,021-08-FCTX,,

AC,GSE36192,GPL6947,GSM882711,SH-96-32-CRBLM,,

AC,GSE36192,GPL6947,GSM883237,SH-97-09-FCTX,,

AC,GSE36192,GPL6947,GSM883066,UMARY-1013-FCTX,,

AC,GSE36192,GPL6947,GSM882857,031-08-CRBLM,,

AC,GSE36192,GPL6947,GSM883156,UMARY-634-FCTX,,

AC,GSE36192,GPL6947,GSM883224,SH-04-19-FCTX,,

AC,GSE36192,GPL6947,GSM882936,BLSA-1603-FCTX,,

AC,GSE36192,GPL6947,GSM882860,021-09-CRBLM,,

AC,GSE36192,GPL6947,GSM882547,UMARY-1865-CRBLM,,

AC,GSE36192,GPL6947,GSM882627,UMARY-1455-CRBLM,,

AC,GSE36192,GPL6947,GSM882817,025-08-CRBLM,,

AC,GSE36192,GPL6947,GSM883250,039-06-FCTX,,

AC,GSE36192,GPL6947,GSM882580,UMARY-388-CRBLM,,

AC,GSE36192,GPL6947,GSM882952,JHU-994-FCTX,,

AC,GSE36192,GPL6947,GSM882941,BLSA-2037-FCTX,,

AC,GSE36192,GPL6947,GSM882544,BLSA-1838-CRBLM,,

AC,GSE36192,GPL6947,GSM882797,031-09-CRBLM,,

AC,GSE36192,GPL6947,GSM883039,MIAMI-3799-FCTX,,

AC,GSE36192,GPL6947,GSM883190,SH-95-21-FCTX,,

AC,GSE36192,GPL6947,GSM882541,UMARY-1037-CRBLM,,

AC,GSE36192,GPL6947,GSM882990,UMARY-1570-FCTX,,

AC,GSE36192,GPL6947,GSM883041,UMARY-1403-FCTX,,

AC,GSE36192,GPL6947,GSM882509,UMARY-1614-CRBLM,,

AC,GSE36192,GPL6947,GSM882636,UMARY-4287-CRBLM,,

AC,GSE36192,GPL6947,GSM882522,UMARY-1133-CRBLM,,

AC,GSE36192,GPL6947,GSM882620,UMARY-689-CRBLM,,

AC,GSE36192,GPL6947,GSM883189,SH-97-53-FCTX,,

AC,GSE36192,GPL6947,GSM882898,BLSA-1839-FCTX,,

AC,GSE36192,GPL6947,GSM882598,UMARY-4976-CRBLM,,

AC,GSE36192,GPL6947,GSM882609,UMARY-1013-CRBLM,,

AC,GSE36192,GPL6947,GSM883209,SH-95-27-FCTX,,

AC,GSE36192,GPL6947,GSM883056,UMARY-165-FCTX,,

AC,GSE36192,GPL6947,GSM883185,SH-01-37-FCTX,,

AC,GSE36192,GPL6947,GSM882592,UMARY-4592-CRBLM,,

AC,GSE36192,GPL6947,GSM882518,UMARY-1078-CRBLM,,

AC,GSE36192,GPL6947,GSM883320,002-08-FCTX,,

AC,GSE36192,GPL6947,GSM883149,UMARY-455-FCTX,,

AC,GSE36192,GPL6947,GSM883137,UMARY-1539-FCTX,,

AC,GSE36192,GPL6947,GSM882966,UMARY-1614-FCTX,,

AC,GSE36192,GPL6947,GSM883045,UMARY-143-FCTX,,

AC,GSE36192,GPL6947,GSM882757,UMARY-4735-CRBLM,,

AC,GSE36192,GPL6947,GSM882503,UMARY-1568-CRBLM,,

AC,GSE36192,GPL6947,GSM883263,027-09-FCTX,,

AC,GSE36192,GPL6947,GSM882955,UMARY-1502-FCTX,,

AC,GSE36192,GPL6947,GSM882928,UMARY-5024-FCTX,,

AC,GSE36192,GPL6947,GSM882599,UMARY-165-CRBLM,,

AC,GSE36192,GPL6947,GSM883069,UMARY-26-FCTX,,

AC,GSE36192,GPL6947,GSM882967,UMARY-1648-FCTX,,

AC,GSE36192,GPL6947,GSM882784,SH-99-02-CRBLM,,

AC,GSE36192,GPL6947,GSM883028,UMARY-5116-FCTX,,

AC,GSE36192,GPL6947,GSM882964,UMARY-1611-FCTX,,

AC,GSE36192,GPL6947,GSM882916,UMARY-4726-FCTX,,

AC,GSE36192,GPL6947,GSM882896,UMARY-1465-FCTX,,

AC,GSE36192,GPL6947,GSM883212,UMARY-1113-FCTX,,

AC,GSE36192,GPL6947,GSM883172,SH-03-63-FCTX,,

AC,GSE36192,GPL6947,GSM882954,UMARY-1454-FCTX,,

AC,GSE36192,GPL6947,GSM882902,UMARY-1907-FCTX,,

AC,GSE36192,GPL6947,GSM882582,MIAMI-3799-CRBLM,,

AC,GSE36192,GPL6947,GSM882699,UMARY-634-CRBLM,,

AC,GSE36192,GPL6947,GSM883338,SH-96-22-FCTX,,

AC,GSE36192,GPL6947,GSM882552,UMARY-4263-CRBLM,,

AC,GSE36192,GPL6947,GSM883065,UMARY-5171-FCTX,,

AC,GSE36192,GPL6947,GSM882915,UMARY-4725-FCTX,,

AC,GSE36192,GPL6947,GSM882878,UMARY-1794-CRBLM,,

AC,GSE36192,GPL6947,GSM882947,JHU-710-FCTX,,

AC,GSE36192,GPL6947,GSM883316,029-09-FCTX,,

AC,GSE36192,GPL6947,GSM882559,UMARY-871-CRBLM,,

AC,GSE36192,GPL6947,GSM883167,SH-94-35-FCTX,,

AC,GSE36192,GPL6947,GSM882792,003-08-CRBLM,,

AC,GSE36192,GPL6947,GSM882486,BLSA-2102-CRBLM,,

AC,GSE36192,GPL6947,GSM882920,UMARY-4786-FCTX,,

AC,GSE36192,GPL6947,GSM883061,UMARY-1024-FCTX,,

AC,GSE36192,GPL6947,GSM882781,SH-97-17-CRBLM,,

AC,GSE36192,GPL6947,GSM883242,SH-99-54-FCTX,,

AC,GSE36192,GPL6947,GSM882579,UMARY-288-CRBLM,,

AC,GSE36192,GPL6947,GSM882911,UMARY-4593-FCTX,,

AC,GSE36192,GPL6947,GSM883244,UMARY-4781-FCTX,,

AC,GSE36192,GPL6947,GSM883030,UMARY-1135-FCTX,,

AC,GSE36192,GPL6947,GSM882989,UMARY-1498-FCTX,,

AC,GSE36192,GPL6947,GSM882512,UMARY-1710-CRBLM,,

AC,GSE36192,GPL6947,GSM883182,SH-99-44-FCTX,,

AC,GSE36192,GPL6947,GSM882616,UMARY-1743-CRBLM,,

AC,GSE36192,GPL6947,GSM882604,UMARY-1024-CRBLM,,

AC,GSE36192,GPL6947,GSM882771,SH-07-28-CRBLM,,

AC,GSE36192,GPL6947,GSM883294,018-08-FCTX,,

AC,GSE36192,GPL6947,GSM882987,UMARY-1486-FCTX,,

AC,GSE36192,GPL6947,GSM883202,SH-06-66-FCTX,,

AC,GSE36192,GPL6947,GSM883009,BLSA-2066-FCTX,,

AC,GSE36192,GPL6947,GSM882501,UMARY-1544-CRBLM,,

AC,GSE36192,GPL6947,GSM882733,SH-95-21-CRBLM,,

AC,GSE36192,GPL6947,GSM882531,UMARY-1498-CRBLM,,

AC,GSE36192,GPL6947,GSM882594,UMARY-305-CRBLM,,

AC,GSE36192,GPL6947,GSM882975,UMARY-1076-FCTX,,

AC,GSE36192,GPL6947,GSM883058,UMARY-1790-FCTX,,

AC,GSE36192,GPL6947,GSM882912,UMARY-4598-FCTX,,

AC,GSE36192,GPL6947,GSM882608,UMARY-5171-CRBLM,,

AC,GSE36192,GPL6947,GSM882825,002-08-CRBLM,,

AC,GSE36192,GPL6947,GSM882562,BLSA-1889-CRBLM,,

AC,GSE36192,GPL6947,GSM883285,009-09-FCTX,,

AC,GSE36192,GPL6947,GSM883233,SH-95-34-FCTX,,

AC,GSE36192,GPL6947,GSM882664,MIAMI-3410-CRBLM,,

AC,GSE36192,GPL6947,GSM882843,020-07-CRBLM,,

AC,GSE36192,GPL6947,GSM882587,UMARY-497-CRBLM,,

AC,GSE36192,GPL6947,GSM882625,UMARY-240-CRBLM,,

AC,GSE36192,GPL6947,GSM883093,UMARY-4287-FCTX,,

AC,GSE36192,GPL6947,GSM882597,UMARY-671-CRBLM,,

AC,GSE36192,GPL6947,GSM882737,SH-04-05-CRBLM,,

AC,GSE36192,GPL6947,GSM882495,JHU-994-CRBLM,,

AC,GSE36192,GPL6947,GSM882855,021-05-CRBLM,,

AC,GSE36192,GPL6947,GSM882981,UMARY-1136-FCTX,,

AC,GSE36192,GPL6947,GSM882708,SH-03-66-CRBLM,,

AC,GSE36192,GPL6947,GSM882502,UMARY-1545-CRBLM,,

AC,GSE36192,GPL6947,GSM882508,UMARY-1612-CRBLM,,

AC,GSE36192,GPL6947,GSM882717,SH-07-46-CRBLM,,

AC,GSE36192,GPL6947,GSM882713,SH-96-44-CRBLM,,

AC,GSE36192,GPL6947,GSM883102,UMARY-1027-FCTX,,

AC,GSE36192,GPL6947,GSM882950,JHU-719-FCTX,,

AC,GSE36192,GPL6947,GSM883279,020-07-FCTX,,

AC,GSE36192,GPL6947,GSM883163,SH-98-23-FCTX,,

AC,GSE36192,GPL6947,GSM883152,UMARY-814-FCTX,,

AC,GSE36192,GPL6947,GSM883105,UMARY-164-FCTX,,

AC,GSE36192,GPL6947,GSM883278,001-06-FCTX,,

AC,GSE36192,GPL6947,GSM882668,UMARY-1423-CRBLM,,

AC,GSE36192,GPL6947,GSM882575,UMARY-1859-CRBLM,,

AC,GSE36192,GPL6947,GSM883146,UMARY-4546-FCTX,,

AC,GSE36192,GPL6947,GSM882459,UMARY-4726-CRBLM,,

AC,GSE36192,GPL6947,GSM883191,SH-03-15-FCTX,,

AC,GSE36192,GPL6947,GSM882676,UMARY-1185-CRBLM,,

AC,GSE36192,GPL6947,GSM882445,UMARY-1907-CRBLM,,

AC,GSE36192,GPL6947,GSM883281,014-08-FCTX,,

AC,GSE36192,GPL6947,GSM882698,UMARY-4722-CRBLM,,

AC,GSE36192,GPL6947,GSM883150,UMARY-5173-FCTX,,

AC,GSE36192,GPL6947,GSM883038,UMARY-1831-FCTX,,

AC,GSE36192,GPL6947,GSM882540,UMARY-1028-CRBLM,,

AC,GSE36192,GPL6947,GSM882433,UMARY-1134-CRBLM,,

AC,GSE36192,GPL6947,GSM882854,035-06-CRBLM,,

AC,GSE36192,GPL6947,GSM882615,UMARY-1861-CRBLM,,

AC,GSE36192,GPL6947,GSM883231,SH-08-44-FCTX,,

AC,GSE36192,GPL6947,GSM882674,UMARY-4638-CRBLM,,

AC,GSE36192,GPL6947,GSM882798,017-08-CRBLM,,

AC,GSE36192,GPL6947,GSM882715,SH-03-63-CRBLM,,

AC,GSE36192,GPL6947,GSM883032,UMARY-1859-FCTX,,

AC,GSE36192,GPL6947,GSM882724,SH-01-46-CRBLM,,

AC,GSE36192,GPL6947,GSM882700,UMARY-1744-CRBLM,,

AC,GSE36192,GPL6947,GSM882705,UMARY-55-CRBLM,,

AC,GSE36192,GPL6947,GSM883276,004-08-FCTX,,

AC,GSE36192,GPL6947,GSM882435,UMARY-1209-CRBLM,,

AC,GSE36192,GPL6947,GSM883268,026-07-FCTX,,

AC,GSE36192,GPL6947,GSM883003,UMARY-1847-FCTX,,

AC,GSE36192,GPL6947,GSM882918,UMARY-4729-FCTX,,

AC,GSE36192,GPL6947,GSM882718,SH-06-25-CRBLM,,

AC,GSE36192,GPL6947,GSM883117,MIAMI-4022-FCTX,,

AC,GSE36192,GPL6947,GSM883272,021-05-FCTX,,

AC,GSE36192,GPL6947,GSM882827,006-10-CRBLM,,

AC,GSE36192,GPL6947,GSM882906,BLSA-2069-FCTX,,

AC,GSE36192,GPL6947,GSM882761,MIAMI-3828-CRBLM,,

AC,GSE36192,GPL6947,GSM882750,SH-96-38-CRBLM,,

AC,GSE36192,GPL6947,GSM882549,UMARY-1867-CRBLM,,

AC,GSE36192,GPL6947,GSM883158,UMARY-4670-FCTX,,

AC,GSE36192,GPL6947,GSM883123,UMARY-1363-FCTX,,

AC,GSE36192,GPL6947,GSM882455,UMARY-4598-CRBLM,,

AC,GSE36192,GPL6947,GSM882933,BLSA-1556-FCTX,,

AC,GSE36192,GPL6947,GSM882948,JHU-712-FCTX,,

AC,GSE36192,GPL6947,GSM883096,UMARY-1379-FCTX,,

AC,GSE36192,GPL6947,GSM882436,UMARY-1260-CRBLM,,

AC,GSE36192,GPL6947,GSM883176,SH-02-08-FCTX,,

AC,GSE36192,GPL6947,GSM883090,MIAMI-2841-FCTX,,

AC,GSE36192,GPL6947,GSM883177,SH-92-05-FCTX,,

AC,GSE36192,GPL6947,GSM883013,UMARY-5028-FCTX,,

AC,GSE36192,GPL6947,GSM882478,BLSA-1595-CRBLM,,

AC,GSE36192,GPL6947,GSM883147,UMARY-4898-FCTX,,

AC,GSE36192,GPL6947,GSM883313,016-08-FCTX,,

AC,GSE36192,GPL6947,GSM882467,UMARY-4903-CRBLM,,

AC,GSE36192,GPL6947,GSM882956,UMARY-1535-FCTX,,

AC,GSE36192,GPL6947,GSM883125,UMARY-1423-FCTX,,

AC,GSE36192,GPL6947,GSM882870,039-06-CRBLM,,

AC,GSE36192,GPL6947,GSM883210,UMARY-818-FCTX,,

AC,GSE36192,GPL6947,GSM882996,UMARY-1797-FCTX,,

AC,GSE36192,GPL6947,GSM882723,SH-05-10-CRBLM,,

AC,GSE36192,GPL6947,GSM882957,UMARY-1540-FCTX,,

AC,GSE36192,GPL6947,GSM883097,UMARY-5179-FCTX,,

AC,GSE36192,GPL6947,GSM883079,UMARY-1841-FCTX,,

AC,GSE36192,GPL6947,GSM883266,010-09-FCTX,,

AC,GSE36192,GPL6947,GSM883001,UMARY-1830-FCTX,,

AC,GSE36192,GPL6947,GSM882968,UMARY-1652-FCTX,,

AC,GSE36192,GPL6947,GSM882646,UMARY-1609-CRBLM,,

AC,GSE36192,GPL6947,GSM882772,SH-07-37-CRBLM,,

AC,GSE36192,GPL6947,GSM882734,SH-03-15-CRBLM,,

AC,GSE36192,GPL6947,GSM882938,BLSA-1805-FCTX,,

AC,GSE36192,GPL6947,GSM882814,007-08-CRBLM,,

AC,GSE36192,GPL6947,GSM882661,MIAMI-3860-CRBLM,,

AC,GSE36192,GPL6947,GSM883229,SH-07-37-FCTX,,

AC,GSE36192,GPL6947,GSM882683,UMARY-1573-CRBLM,,

AC,GSE36192,GPL6947,GSM882867,026-07-CRBLM,,

AC,GSE36192,GPL6947,GSM882888,JHU-384-FCTX,,

AC,GSE36192,GPL6947,GSM883213,UMARY-72-FCTX,,

AC,GSE36192,GPL6947,GSM882438,UMARY-142-CRBLM,,

AC,GSE36192,GPL6947,GSM882588,UMARY-143-CRBLM,,

AC,GSE36192,GPL6947,GSM882449,BLSA-2069-CRBLM,,

AC,GSE36192,GPL6947,GSM882659,MIAMI-3278-CRBLM,,

AC,GSE36192,GPL6947,GSM882740,SH-03-28-CRBLM,,

AC,GSE36192,GPL6947,GSM882440,UMARY-1583-CRBLM,,

AC,GSE36192,GPL6947,GSM882852,018-09-CRBLM,,

AC,GSE36192,GPL6947,GSM882765,SH-02-06-CRBLM,,

AC,GSE36192,GPL6947,GSM882828,034-08-CRBLM,,

AC,GSE36192,GPL6947,GSM882473,UMARY-5079-CRBLM,,

AC,GSE36192,GPL6947,GSM882450,UMARY-4590-CRBLM,,

AC,GSE36192,GPL6947,GSM882561,BLSA-1802-CRBLM,,

AC,GSE36192,GPL6947,GSM882619,UMARY-260-CRBLM,,

AC,GSE36192,GPL6947,GSM883296,012-09-FCTX,,

AC,GSE36192,GPL6947,GSM883112,UMARY-1461-FCTX,,

AC,GSE36192,GPL6947,GSM883175,SH-06-25-FCTX,,

AC,GSE36192,GPL6947,GSM882869,012-10-CRBLM,,

AC,GSE36192,GPL6947,GSM883203,SH-01-31-FCTX,,

AC,GSE36192,GPL6947,GSM883119,MIAMI-3751-FCTX,,

AC,GSE36192,GPL6947,GSM882469,UMARY-4916-CRBLM,,

AC,GSE36192,GPL6947,GSM882499,UMARY-1535-CRBLM,,

AC,GSE36192,GPL6947,GSM882669,UMARY-1908-CRBLM,,

AC,GSE36192,GPL6947,GSM882744,SH-05-16-CRBLM,,

AC,GSE36192,GPL6947,GSM882686,UMARY-794-CRBLM,,

AC,GSE36192,GPL6947,GSM883036,UMARY-288-FCTX,,

AC,GSE36192,GPL6947,GSM883173,SH-98-32-FCTX,,

AC,GSE36192,GPL6947,GSM883007,UMARY-1867-FCTX,,

AC,GSE36192,GPL6947,GSM883035,UMARY-1365-FCTX,,

AC,GSE36192,GPL6947,GSM882557,UMARY-604-CRBLM,,

AC,GSE36192,GPL6947,GSM883160,UMARY-1674-FCTX,,

AC,GSE36192,GPL6947,GSM882639,UMARY-1379-CRBLM,,

AC,GSE36192,GPL6947,GSM883239,SH-97-46-FCTX,,

AC,GSE36192,GPL6947,GSM882687,UMARY-510-CRBLM,,

AC,GSE36192,GPL6947,GSM882930,UMARY-5079-FCTX,,

AC,GSE36192,GPL6947,GSM882500,UMARY-1540-CRBLM,,

AC,GSE36192,GPL6947,GSM882691,UMARY-1569-CRBLM,,

AC,GSE36192,GPL6947,GSM882773,SH-08-04-CRBLM,,

AC,GSE36192,GPL6947,GSM882633,MIAMI-2841-CRBLM,,

AC,GSE36192,GPL6947,GSM882523,UMARY-1136-CRBLM,,

AC,GSE36192,GPL6947,GSM883252,025-08-FCTX,,

AC,GSE36192,GPL6947,GSM883083,UMARY-5120-FCTX,,

AC,GSE36192,GPL6947,GSM882883,SH-03-11-CRBLM,,

AC,GSE36192,GPL6947,GSM882738,SH-07-18-CRBLM,,

AC,GSE36192,GPL6947,GSM883116,MIAMI-3278-FCTX,,

AC,GSE36192,GPL6947,GSM882462,UMARY-4782-CRBLM,,

AC,GSE36192,GPL6947,GSM882601,UMARY-1790-CRBLM,,

AC,GSE36192,GPL6947,GSM883217,MIAMI-3797-FCTX,,

AC,GSE36192,GPL6947,GSM882742,SH-99-50-CRBLM,,

AC,GSE36192,GPL6947,GSM883142,UMARY-1322-FCTX,,

AC,GSE36192,GPL6947,GSM883072,UMARY-1861-FCTX,,

AC,GSE36192,GPL6947,GSM882884,SH-97-02-CRBLM,,

AC,GSE36192,GPL6947,GSM883232,SH-95-02-FCTX,,

AC,GSE36192,GPL6947,GSM883082,UMARY-240-FCTX,,

AC,GSE36192,GPL6947,GSM883130,UMARY-4724-FCTX,,

AC,GSE36192,GPL6947,GSM882755,UMARY-1113-CRBLM,,

AC,GSE36192,GPL6947,GSM883002,BLSA-1838-FCTX,,

AC,GSE36192,GPL6947,GSM882694,UMARY-105-CRBLM,,

AC,GSE36192,GPL6947,GSM882768,SH-04-21-CRBLM,,

AC,GSE36192,GPL6947,GSM883044,UMARY-497-FCTX,,

AC,GSE36192,GPL6947,GSM882558,UMARY-813-CRBLM,,

AC,GSE36192,GPL6947,GSM883006,UMARY-1866-FCTX,,

AC,GSE36192,GPL6947,GSM883085,UMARY-1464-FCTX,,

AC,GSE36192,GPL6947,GSM882780,SH-97-09-CRBLM,,

AC,GSE36192,GPL6947,GSM883087,UMARY-1347-FCTX,,

AC,GSE36192,GPL6947,GSM883181,SH-01-46-FCTX,,

AC,GSE36192,GPL6947,GSM882760,MIAMI-3797-CRBLM,,

AC,GSE36192,GPL6947,GSM883180,SH-05-10-FCTX,,

AC,GSE36192,GPL6947,GSM882974,UMARY-1065-FCTX,,

AC,GSE36192,GPL6947,GSM882629,UMARY-1497-CRBLM,,

AC,GSE36192,GPL6947,GSM883293,034-08-FCTX,,

AC,GSE36192,GPL6947,GSM883326,038-09-FCTX,,

AC,GSE36192,GPL6947,GSM882553,UMARY-4788-CRBLM,,

AC,GSE36192,GPL6947,GSM882794,036-08-CRBLM,,

AC,GSE36192,GPL6947,GSM882489,JHU-705-CRBLM,,

AC,GSE36192,GPL6947,GSM883301,026-09-FCTX,,

AC,GSE36192,GPL6947,GSM882910,UMARY-4545-FCTX,,

AC,GSE36192,GPL6947,GSM882556,UMARY-602-CRBLM,,

AC,GSE36192,GPL6947,GSM882732,SH-97-53-CRBLM,,

AC,GSE36192,GPL6947,GSM882892,UMARY-1209-FCTX,,

AC,GSE36192,GPL6947,GSM882714,SH-06-21-CRBLM,,

AC,GSE36192,GPL6947,GSM882914,UMARY-4640-FCTX,,

AC,GSE36192,GPL6947,GSM882555,UMARY-5028-CRBLM,,

AC,GSE36192,GPL6947,GSM882569,UMARY-1441-CRBLM,,

AC,GSE36192,GPL6947,GSM883118,MIAMI-3860-FCTX,,

AC,GSE36192,GPL6947,GSM882685,UMARY-1322-CRBLM,,

AC,GSE36192,GPL6947,GSM882743,SH-00-34-CRBLM,,

AC,GSE36192,GPL6947,GSM882448,BLSA-2020-CRBLM,,

AC,GSE36192,GPL6947,GSM883258,018-09-FCTX,,

AC,GSE36192,GPL6947,GSM882988,UMARY-1496-FCTX,,

AC,GSE36192,GPL6947,GSM882441,BLSA-1839-CRBLM,,

AC,GSE36192,GPL6947,GSM882889,UMARY-1105-FCTX,,

AC,GSE36192,GPL6947,GSM882719,SH-02-08-CRBLM,,

AC,GSE36192,GPL6947,GSM882505,UMARY-1578-CRBLM,,

AC,GSE36192,GPL6947,GSM882776,SH-95-34-CRBLM,,

AC,GSE36192,GPL6947,GSM883339,SH-03-11-FCTX,,

AC,GSE36192,GPL6947,GSM882618,UMARY-290-CRBLM,,

AC,GSE36192,GPL6947,GSM882606,UMARY-772-CRBLM,,

AC,GSE36192,GPL6947,GSM883332,UMARY-5125-FCTX,,

AC,GSE36192,GPL6947,GSM883034,UMARY-1428-FCTX,,

AC,GSE36192,GPL6947,GSM883143,UMARY-794-FCTX,,

AC,GSE36192,GPL6947,GSM882498,UMARY-1502-CRBLM,,

AC,GSE36192,GPL6947,GSM882519,UMARY-1079-CRBLM,,

AC,GSE36192,GPL6947,GSM882566,UMARY-1668-CRBLM,,

AC,GSE36192,GPL6947,GSM882595,UMARY-630-CRBLM,,

AC,GSE36192,GPL6947,GSM882995,UMARY-1795-FCTX,,

AC,GSE36192,GPL6947,GSM882673,UMARY-4724-CRBLM,,

AC,GSE36192,GPL6947,GSM882657,MIAMI-3643-CRBLM,,

AC,GSE36192,GPL6947,GSM882653,MIAMI-3228-CRBLM,,

AC,GSE36192,GPL6947,GSM882730,SH-92-32-CRBLM,,

AC,GSE36192,GPL6947,GSM882679,UMARY-1672-CRBLM,,

AC,GSE36192,GPL6947,GSM883153,UMARY-4906-FCTX,,

AC,GSE36192,GPL6947,GSM883184,SH-96-13-FCTX,,

AC,GSE36192,GPL6947,GSM883092,MIAMI-3747-FCTX,,

AC,GSE36192,GPL6947,GSM883291,007-06-FCTX,,

AC,GSE36192,GPL6947,GSM883199,SH-99-50-FCTX,,

AC,GSE36192,GPL6947,GSM882904,BLSA-2000-FCTX,,

AC,GSE36192,GPL6947,GSM883262,016-09-FCTX,,

AC,GSE36192,GPL6947,GSM883323,034-07-FCTX,,

AC,GSE36192,GPL6947,GSM883319,017-09-FCTX,,

AC,GSE36192,GPL6947,GSM882642,UMARY-1406-CRBLM,,

AC,GSE36192,GPL6947,GSM883168,SH-96-32-FCTX,,

AC,GSE36192,GPL6947,GSM882959,UMARY-1545-FCTX,,

AC,GSE36192,GPL6947,GSM882665,MIAMI-3772-CRBLM,,

AC,GSE36192,GPL6947,GSM883178,SH-03-17-FCTX,,

AC,GSE36192,GPL6947,GSM882581,UMARY-1831-CRBLM,,

AC,GSE36192,GPL6947,GSM882907,UMARY-4590-FCTX,,

AC,GSE36192,GPL6947,GSM882567,UMARY-1274-CRBLM,,

AC,GSE36192,GPL6947,GSM882842,014-09-CRBLM,,

AC,GSE36192,GPL6947,GSM883315,004-10-FCTX,,

AC,GSE36192,GPL6947,GSM882971,UMARY-1909-FCTX,,

AC,GSE36192,GPL6947,GSM882551,BLSA-2066-CRBLM,,

AC,GSE36192,GPL6947,GSM882823,001-10-CRBLM,,

AC,GSE36192,GPL6947,GSM883113,MIAMI-3216-FCTX,,

AC,GSE36192,GPL6947,GSM882891,UMARY-4549-FCTX,,

AC,GSE36192,GPL6947,GSM882515,UMARY-1936-CRBLM,,

AC,GSE36192,GPL6947,GSM883073,UMARY-1743-FCTX,,

AC,GSE36192,GPL6947,GSM882458,UMARY-4725-CRBLM,,

AC,GSE36192,GPL6947,GSM882831,034-06-CRBLM,,

AC,GSE36192,GPL6947,GSM883115,UMARY-5117-FCTX,,

AC,GSE36192,GPL6947,GSM882728,SH-01-37-CRBLM,,

AC,GSE36192,GPL6947,GSM882707,SH-04-08-CRBLM,,

AC,GSE36192,GPL6947,GSM883282,028-08-FCTX,,

AC,GSE36192,GPL6947,GSM882791,024-09-CRBLM,,

AC,GSE36192,GPL6947,GSM883336,SH-97-14-FCTX,,

AC,GSE36192,GPL6947,GSM882675,UMARY-1599-CRBLM,,

AC,GSE36192,GPL6947,GSM883192,SH-99-31-FCTX,,

AC,GSE36192,GPL6947,GSM882978,UMARY-1104-FCTX,,

AC,GSE36192,GPL6947,GSM882736,SH-03-50-CRBLM,,

AC,GSE36192,GPL6947,GSM882538,UMARY-1797-CRBLM,,

AC,GSE36192,GPL6947,GSM883100,MIAMI-2112-FCTX,,

AC,GSE36192,GPL6947,GSM883243,UMARY-1607-FCTX,,

AC,GSE36192,GPL6947,GSM882976,UMARY-1078-FCTX,,

AC,GSE36192,GPL6947,GSM882963,UMARY-1584-FCTX,,

AC,GSE36192,GPL6947,GSM882946,JHU-705-FCTX,,

AC,GSE36192,GPL6947,GSM882999,UMARY-1037-FCTX,,

AC,GSE36192,GPL6947,GSM882885,SH-00-38-CRBLM,,

AC,GSE36192,GPL6947,GSM882662,MIAMI-3751-CRBLM,,

AC,GSE36192,GPL6947,GSM882897,UMARY-1583-FCTX,,

AC,GSE36192,GPL6947,GSM882962,UMARY-1578-FCTX,,

AC,GSE36192,GPL6947,GSM883241,SH-99-02-FCTX,,

AC,GSE36192,GPL6947,GSM883000,UMARY-1827-FCTX,,

AC,GSE36192,GPL6947,GSM882506,UMARY-1584-CRBLM,,

AC,GSE36192,GPL6947,GSM883031,UMARY-1038-FCTX,,

AC,GSE36192,GPL6947,GSM882939,BLSA-1924-FCTX,,

AC,GSE36192,GPL6947,GSM882931,UMARY-5081-FCTX,,

AC,GSE36192,GPL6947,GSM882641,UMARY-1277-CRBLM,,

AC,GSE36192,GPL6947,GSM883037,UMARY-388-FCTX,,

AC,GSE36192,GPL6947,GSM882731,SH-99-29-CRBLM,,

AC,GSE36192,GPL6947,GSM882826,007-06-CRBLM,,

AC,GSE36192,GPL6947,GSM882960,UMARY-1568-FCTX,,

AC,GSE36192,GPL6947,GSM882753,UMARY-818-CRBLM,,

AC,GSE36192,GPL6947,GSM883331,025-09-FCTX,,

AC,GSE36192,GPL6947,GSM882511,UMARY-1652-CRBLM,,

AC,GSE36192,GPL6947,GSM883264,033-09-FCTX,,

AC,GSE36192,GPL6947,GSM883342,UMARY-177-FCTX,,

AC,GSE36192,GPL6947,GSM883070,UMARY-251-FCTX,,

AC,GSE36192,GPL6947,GSM882574,UMARY-1038-CRBLM,,

AC,GSE36192,GPL6947,GSM882539,UMARY-1825-CRBLM,,

AC,GSE36192,GPL6947,GSM882877,UMARY-4669-CRBLM,,

AC,GSE36192,GPL6947,GSM883267,011-10-FCTX,,

AC,GSE36192,GPL6947,GSM882865,023-08-CRBLM,,

AC,GSE36192,GPL6947,GSM882672,UMARY-662-CRBLM,,

AC,GSE36192,GPL6947,GSM883226,SH-06-05-FCTX,,

AC,GSE36192,GPL6947,GSM882621,UMARY-1442-CRBLM,,

AC,GSE36192,GPL6947,GSM882596,UMARY-229-CRBLM,,

AC,GSE36192,GPL6947,GSM883114,MIAMI-3643-FCTX,,

AC,GSE36192,GPL6947,GSM882751,SH-95-26-CRBLM,,

AC,GSE36192,GPL6947,GSM883107,UMARY-5119-FCTX,,

AC,GSE36192,GPL6947,GSM882903,UMARY-1940-FCTX,,

AC,GSE36192,GPL6947,GSM882767,SH-04-19-CRBLM,,

AC,GSE36192,GPL6947,GSM882815,007-09-CRBLM,,

AC,GSE36192,GPL6947,GSM883019,BLSA-1802-FCTX,,

AC,GSE36192,GPL6947,GSM883318,030-09-FCTX,,

AC,GSE36192,GPL6947,GSM882788,UMARY-5077-CRBLM,,

AC,GSE36192,GPL6947,GSM882741,SH-07-04-CRBLM,,

AC,GSE36192,GPL6947,GSM882442,BLSA-1840-CRBLM,,

AC,GSE36192,GPL6947,GSM882697,UMARY-4591-CRBLM,,

AC,GSE36192,GPL6947,GSM883023,UMARY-933-FCTX,,

AC,GSE36192,GPL6947,GSM883271,024-09-FCTX,,

AC,GSE36192,GPL6947,GSM882809,022-06-CRBLM,,

AC,GSE36192,GPL6947,GSM883304,023-08-FCTX,,

AC,GSE36192,GPL6947,GSM882703,UMARY-1674-CRBLM,,

AC,GSE36192,GPL6947,GSM882583,MIAMI-3231-CRBLM,,

AC,GSE36192,GPL6947,GSM883292,012-08-FCTX,,

AC,GSE36192,GPL6947,GSM882532,UMARY-1570-CRBLM,,

AC,GSE36192,GPL6947,GSM882628,UMARY-1464-CRBLM,,

AC,GSE36192,GPL6947,GSM882610,UMARY-1213-CRBLM,,

AC,GSE36192,GPL6947,GSM883238,SH-97-17-FCTX,,

AC,GSE36192,GPL6947,GSM882701,UMARY-4670-CRBLM,,

AC,GSE36192,GPL6947,GSM883148,UMARY-1569-FCTX,,

AC,GSE36192,GPL6947,GSM883021,UMARY-1064-FCTX,,

AC,GSE36192,GPL6947,GSM883017,UMARY-871-FCTX,,

AC,GSE36192,GPL6947,GSM882970,UMARY-1710-FCTX,,

AC,GSE36192,GPL6947,GSM882806,010-09-CRBLM,,

AC,GSE36192,GPL6947,GSM883014,UMARY-602-FCTX,,

AC,GSE36192,GPL6947,GSM882837,037-08-CRBLM,,

AC,GSE36192,GPL6947,GSM883284,005-09-FCTX,,

AC,GSE36192,GPL6947,GSM882488,BLSA-827-CRBLM,,

AC,GSE36192,GPL6947,GSM882546,UMARY-1862-CRBLM,,

AC,GSE36192,GPL6947,GSM882651,UMARY-5085-CRBLM,,

AC,GSE36192,GPL6947,GSM882634,UMARY-1378-CRBLM,,

AC,GSE36192,GPL6947,GSM882961,UMARY-1571-FCTX,,

AC,GSE36192,GPL6947,GSM883159,UMARY-544-FCTX,,

AC,GSE36192,GPL6947,GSM882758,MIAMI-1931-CRBLM,,

AC,GSE36192,GPL6947,GSM883128,UMARY-4848-FCTX,,

AC,GSE36192,GPL6947,GSM882709,SH-96-30-CRBLM,,

AC,GSE36192,GPL6947,GSM883104,UMARY-1864-FCTX,,

AC,GSE36192,GPL6947,GSM882982,UMARY-1158-FCTX,,

AC,GSE36192,GPL6947,GSM883088,UMARY-1675-FCTX,,

AC,GSE36192,GPL6947,GSM883283,012-10-FCTX,,

AC,GSE36192,GPL6947,GSM882901,BLSA-1883-FCTX,,

AC,GSE36192,GPL6947,GSM882807,011-09-CRBLM,,

AC,GSE36192,GPL6947,GSM883246,036-09-FCTX,,

AC,GSE36192,GPL6947,GSM883302,026-08-FCTX,,

AC,GSE36192,GPL6947,GSM883071,UMARY-650-FCTX,,

AC,GSE36192,GPL6947,GSM883334,UMARY-1794-FCTX,,

AC,GSE36192,GPL6947,GSM882464,UMARY-914-CRBLM,,

AC,GSE36192,GPL6947,GSM882799,025-09-CRBLM,,

AC,GSE36192,GPL6947,GSM882762,MIAMI-4042-CRBLM,,

AC,GSE36192,GPL6947,GSM882465,UMARY-4841-CRBLM,,

AC,GSE36192,GPL6947,GSM882890,UMARY-1134-FCTX,,

AC,GSE36192,GPL6947,GSM882706,SH-98-23-CRBLM,,

AC,GSE36192,GPL6947,GSM882474,UMARY-5081-CRBLM,,

AC,GSE36192,GPL6947,GSM882475,UMARY-5087-CRBLM,,

AC,GSE36192,GPL6947,GSM882926,UMARY-4916-FCTX,,

AC,GSE36192,GPL6947,GSM882593,UMARY-4228-CRBLM,,

AC,GSE36192,GPL6947,GSM883005,UMARY-1865-FCTX,,

AC,GSE36192,GPL6947,GSM882721,SH-03-17-CRBLM,,

AC,GSE36192,GPL6947,GSM882804,026-08-CRBLM,,

AC,GSE36192,GPL6947,GSM882602,UMARY-5089-CRBLM,,

AC,GSE36192,GPL6947,GSM883111,MIAMI-2852-FCTX,,

AC,GSE36192,GPL6947,GSM882510,UMARY-1648-CRBLM,,

AC,GSE36192,GPL6947,GSM882632,UMARY-1429-CRBLM,,

AC,GSE36192,GPL6947,GSM883018,UMARY-879-FCTX,,

AC,GSE36192,GPL6947,GSM882529,UMARY-1486-CRBLM,,

AC,GSE36192,GPL6947,GSM883064,UMARY-4728-FCTX,,

AC,GSE36192,GPL6947,GSM883311,010-10-FCTX,,

AC,GSE36192,GPL6947,GSM883135,UMARY-5111-FCTX,,

AC,GSE36192,GPL6947,GSM882451,UMARY-4540-CRBLM,,

AC,GSE36192,GPL6947,GSM882787,UMARY-4781-CRBLM,,

AC,GSE36192,GPL6947,GSM882471,UMARY-5024-CRBLM,,

AC,GSE36192,GPL6947,GSM883196,SH-07-72-FCTX,,

AC,GSE36192,GPL6947,GSM882446,UMARY-1940-CRBLM,,

AC,GSE36192,GPL6947,GSM883089,UMARY-1429-FCTX,,

AC,GSE36192,GPL6947,GSM883166,SH-96-30-FCTX,,

AC,GSE36192,GPL6947,GSM883288,018-07-FCTX,,

AC,GSE36192,GPL6947,GSM883170,SH-96-44-FCTX,,

AC,GSE36192,GPL6947,GSM883223,SH-02-12-FCTX,,

AC,GSE36192,GPL6947,GSM882605,UMARY-1843-CRBLM,,

AC,GSE36192,GPL6947,GSM883060,UMARY-433-FCTX,,

AC,GSE36192,GPL6947,GSM882530,UMARY-1496-CRBLM,,

AC,GSE36192,GPL6947,GSM883277,018-05-FCTX,,

AC,GSE36192,GPL6947,GSM883140,UMARY-1573-FCTX,,

AC,GSE36192,GPL6947,GSM883043,UMARY-1323-FCTX,,

AC,GSE36192,GPL6947,GSM882643,MIAMI-2112-CRBLM,,

AC,GSE36192,GPL6947,GSM882520,UMARY-1104-CRBLM,,

AC,GSE36192,GPL6947,GSM883225,SH-04-21-FCTX,,
[truncated: 425,665 more chars]
